# Facile, Reversible Hydrogen Activation by Low-Coordinate Magnesium Oxide Complexes

**DOI:** 10.1021/jacs.4c16041

**Published:** 2025-01-29

**Authors:** Samuel Thompson, Stuart Burnett, Rochelle Ferns, Tanja van Mourik, Aidan P. McKay, Alexandra M. Z. Slawin, David B. Cordes, Andreas Stasch

**Affiliations:** EaStCHEM School of Chemistry, University of St Andrews, North Haugh, St Andrews KY16 9ST, U.K.

## Abstract

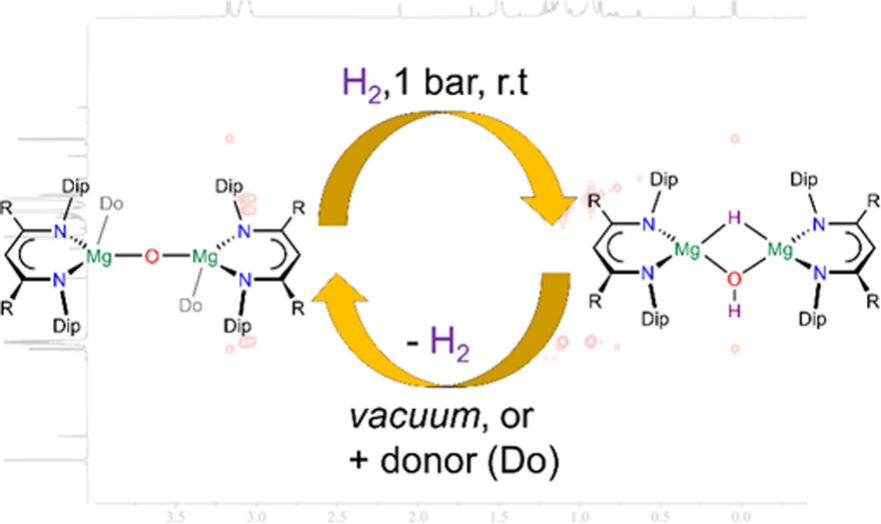

New approaches to achieve facile and reversible dihydrogen activation are of importance for synthesis, catalysis, and hydrogen storage. Here we show that low-coordinate magnesium oxide complexes [{(^RDip^nacnac)Mg}_2_(μ-O)] **1**, with ^RDip^nacnac = HC(RCNDip)_2_, Dip = 2,6-*i*Pr_2_C_6_H_3_, R = Me (**1a**), Et (**1b**), *i*Pr (**1c**), readily react with dihydrogen under mild conditions to afford mixed hydride-hydroxide complexes [{(^RDip^nacnac)Mg}_2_(μ-H)(μ-OH)] **4**. Dehydrogenation of complexes **4** is strongly dependent on remote ligand substitution and can be achieved by simple vacuum-degassing of **4c** (R = *i*Pr) to regain **1c**. Donor addition to complexes **4** also releases hydrogen and affords donor adducts of magnesium oxide complexes. Computational studies suggest that the hydrogen activation mechanism involves nucleophilic attack of an oxide lone pair at a weakly bound H_2_···Mg complex in an S_N_2-like manner that induces a heterolytic dihydrogen cleavage to yield an MgOH and an MgH unit. Alternative synthetic routes into complex **4b** from a magnesium hydride complex have been investigated and the ability of complexes **1** or **4** to act as catalysts for the hydrogenation of 1,1-diphenylethene (DPE) has been tested.

## Introduction

Hydrogen has a key role as a feedstock in industrial chemical production^1^ and as an energy carrier.^2^ Understanding and applying the activation of the dihydrogen molecule, including related elementary reaction steps such as the addition and elimination of hydrogen in various hydrogenation and dehydrogenation reactions,^3,4^ are crucial for the development of hydrogenation processes that are ubiquitous in the production of bulk commodities and fine chemicals.^1,5^ Furthermore, reversible hydrogen uptake is of interest for hydrogen storage applications.^6^ Hydrogen activation is dominated by transition metal complexes and materials,^3,4^ but over the last 20 years, new approaches for hydrogen activation with well-defined main group element compounds under mild conditions have been discovered and include compound classes such as heavier group 14 element alkyne analogues,^7,8^ frustrated Lewis pairs (FLPs),^9−11^ stable reactive carbenes^12^ and related compound classes from the *p*-block.^13−16^ These greatly advanced our understanding of (reversible) hydrogen splitting and introduced new approaches to stoichiometric and catalytic hydrogenations.^10,11,14^ The mechanisms of these facile activation reactions are typically described by synergistic orbital interactions of suitable symmetry and energy. In these, the HOMO of dihydrogen (σ-bond) donates to an empty orbital of the *p*-block reagent molecule (e.g., the LUMO) and the reagent donates electron density (e.g., from the HOMO) to the LUMO of dihydrogen (σ*-orbital). Both combinations of orbital interactions weaken the H–H bond and simultaneously strengthen newly formed element-hydrogen bonds. Hydrogen activation by well-defined alkaline earth metal complexes under mild conditions^16−19^ is comparably rare and has, for example, been achieved by few selected reactive organometallic and hydride complexes,^16−19^ and by some unusually bonded low-oxidation state species.^17,20−25^ This progress has also translated to selected alkaline earth metal complexes being employed as hydrogenation catalysts, for example using their highly polar metal ligand bonds^26,27^ or using metal–ligand cooperativity^28,29^ via redox-noninnocent pincer ligands.^30−32^

The alkaline earth metals are named after their basic oxides and their metal ions are coordinated to oxygen-based anions in nature.^33^ Ionic, rock salt MgO in its various forms has found a wide range of applications,^34,35^ and has been used as a solid catalyst support^36^ and in nanoparticle form to catalyze or enable a variety of organic bond forming reactions.^37^ When treated with dihydrogen, the gas is predominantly physisorbed on the MgO surface, but this also leads to heterolytic dissociation on few reactive, likely three-coordinate acid (Mg) and base (O) sites on the surface.^38,39^ This shows the significant increase in reactivity of exposed low-coordinate ion sites although it has been suggested that low coordination alone is not responsible for the large increase in reactivity.^40^ Despite the ubiquitous nature of metal–oxygen interactions in alkaline earth metal chemistry, well-defined oxide complexes with low-coordinate oxide centers are rare.^41^ For magnesium, few examples have been afforded by reactions of low-oxidation state species^42−44^ with nitrous oxide (N_2_O),^45−50^ or in one instance by reacting a magnesium hydride complex with N_2_O via an intermediate MgOH complex.^51^ These examples typically require access to a suitable precursor species and a robust and sterically demanding ligand to control the product aggregation state. In addition, complexes with magnesium oxide fragments in higher coordination numbers have been characterized where the oxide fragment often originated from adventitious impurities such as water or air, or by degradation reactions of oxygen-containing solvents or reagents.^52,53^ In this work we describe the facile and reversible hydrogen activation by magnesium oxide complexes and the relationship between magnesium hydride, hydroxide, and oxide species.

## Results and Discussion

### Synthesis and Characterization

**Scheme 1:**
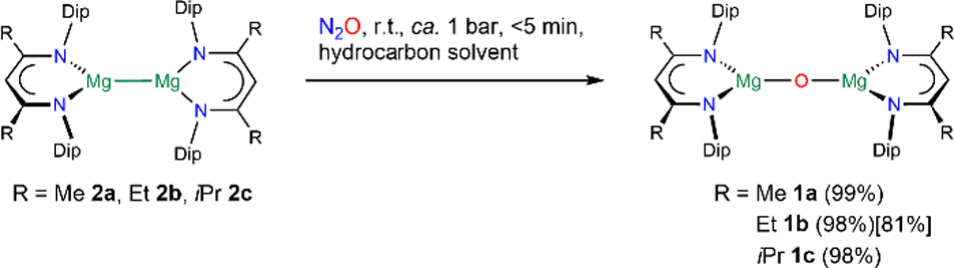
Synthesis of Magnesium Oxide Complexes 1

The well-defined, low-coordinate magnesium oxide complexes [{(^RDip^nacnac)Mg}_2_(μ-O)] **1**, with ^RDip^nacnac = HC(RCNDip)_2_, R = Me (**1a**),^45^ Et (**1b**), *i*Pr (**1c**),^47^ Dip = 2,6-*i*Pr_2_C_6_H_3_, were prepared from the rapid reaction of the corresponding dimagnesium(I) complexes^42−44,47,54^ [{(^RDip^nacnac)Mg}_2_] **2** (R = Me **2a**, Et **2b**, *i*Pr **2c**) with N_2_O in hydrocarbon solvents such as benzene, toluene or *n*-hexane (Scheme 1). The new Mg^I^ complex [{(^EtDip^nacnac)Mg}_2_] **2b** (R = Et) has been prepared in an analogous manner to those with R = Me, *i*Pr and characterized, see the Supporting Information (1.2.1) for full details.

**Figure 1:**
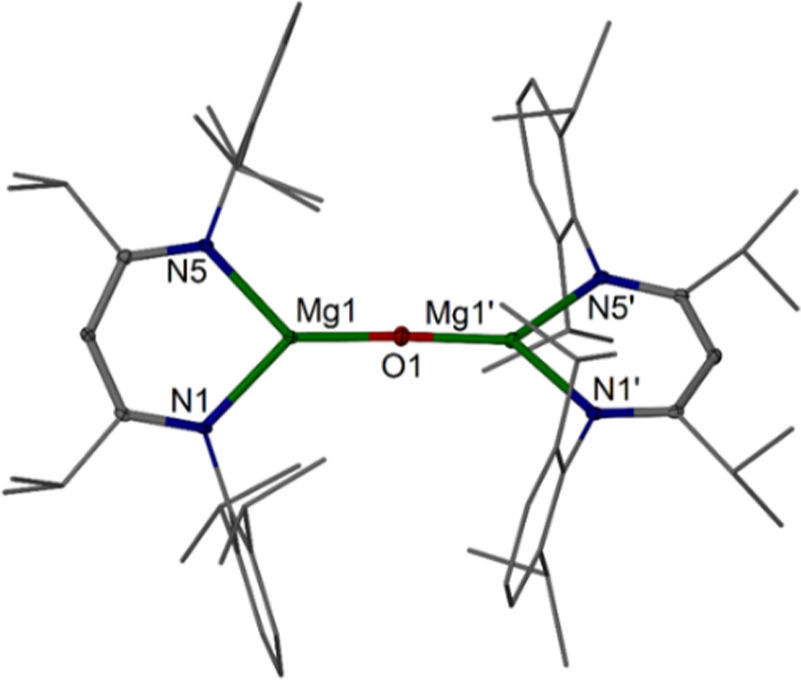
Molecular structure of [{(^iPrDip^nacnac)Mg}_2_(μ-O)] **1c** (30% thermal ellipsoids, partial wireframe). Hydrogen atoms are omitted. Selected bond lengths (Å) and angle (deg): Mg1–O1 1.8042(7), Mg1–N1 2.0512(19), Mg1–N5 2.0410(18); Mg1–O1–Mg1′ 177.28(16).

The molecular structure of a donor-free oxide complex, [{(^iPrDip^nacnac)Mg}_2_(μ-O)] **1c**, was determined by single crystal X-ray diffraction (Figure 1) and shows a near-linear (177.28(16)°) low-coordinate Mg–O–Mg unit with Mg–O distances (1.8042(7) Å) similar to those found for few related donor adducts,^45,47^ a partial molecular structure,^47^ and from a DFT computational study (1.805 Å for **1c**).^55^ Complexes **2b** (Figure S180) and **2c** (Figure S181) were also structurally characterized and the geometries are highly comparable to those of **2a**.^45,47^ Handling of complexes **1** requires thoroughly dry conditions and the tendency of **1** to readily react with traces of moisture, or with other substances, to form magnesium hydroxide complexes [{(^RDip^nacnac)Mg(μ-OH)}_2_] **3** (R = Me **3a**, Et **3b**, *i*Pr **3c**), vide infra, has been noted before,^45,47^ and gives a hint of the high reactivity of these species. Complexes **1** formed rapidly in near quantitative in situ yields showing only minute traces of hydroxide complexes **3** when suitably dried nitrous oxide was used. Isolated **1b** was afforded in 81% yield with 7% **3b** on a small scale. Complex **1b** is stable in the solid state under argon and only decomposes above 280 °C. A solution of **1b** in deuterated benzene can be heated at 100 °C for 87 h without showing significant decomposition.

**Scheme 2:**
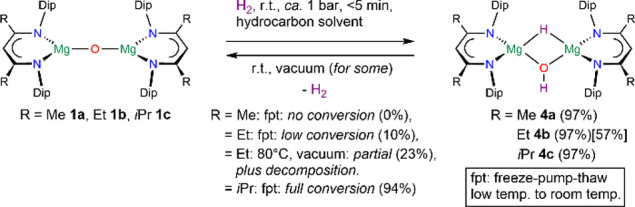
Reversible Hydrogen Activation by Magnesium Oxide Complexes 1

Treating complexes [{(^RDip^nacnac)Mg}_2_(μ-O)] **1**, with ca. 1 bar of hydrogen gas at room temperature in hydrocarbon solvents such as deuterated benzene showed the rapid consumption of **1** and clean formation of the hydrogen addition product [{(^RDip^nacnac)Mg}_2_(μ-H)(μ-OH)] **4** (R = Me **4a**, Et **4b**, *i*Pr **4c**) (Scheme 2) when followed by ^1^H NMR spectroscopy.

**Figure 2:**
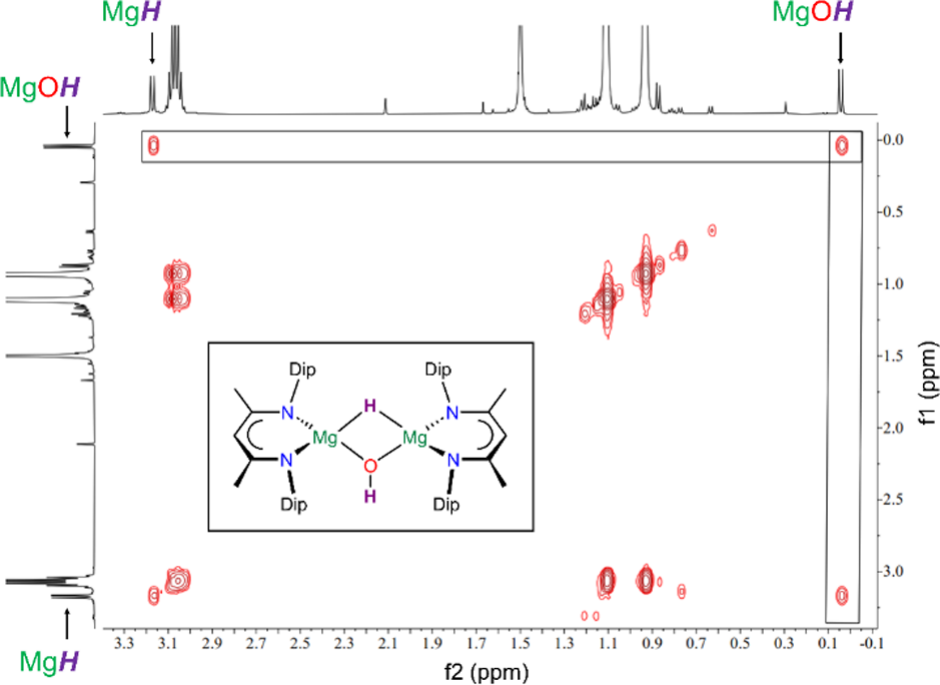
Excerpt of the ^1^H,^1^H COSY NMR spectrum (499.9 MHz, C_6_D_6_, 298 K) of [{(^MeDip^nacnac)Mg}_2_(μ-H)(μ-OH)] **4a** showing the coupling between the MgO*H* and Mg*H* hydrogen atoms.

This was evidenced by the characteristic appearance of two doublets (^3^*J*_HH_ = 8.0 Hz for **1a**) at δ 0.04 ppm (MgO*H*, **1a**) and δ 3.17 ppm (Mg*H*, **1a**), that show no association with carbon resonances in 2D NMR spectra, as can be seen in the excerpt of the ^1^H,^1^H COSY NMR spectrum (Figure 2). Equally, the reaction of **1b** with D_2_ rapidly formed [{(^EtDip^nacnac)Mg}_2_(μ-D)(μ-OD)], **4b-***d*_**2**_, and the ^2^H NMR spectrum shows a very broad resonance around ca. δ −0.2 ppm (MgO*D*) and a singlet at δ 3.24 ppm (Mg*D*), highlighting that there is no resolved coupling between the two deuterium nuclei (Figures S94 and S96). Conducting the reaction of **1b** with hydrogen strictly held at −30 °C for 30 min in *n*-hexane followed by low-temperature degassing resulted in only low conversion to **4b** and largely unreacted **1b**. Complexes **4** could be prepared in situ in essentially quantitative yields and only traces of decomposition to hydroxide complexes **3** were found due to the high sensitivity of the oxide complexes to moisture. Complex **4b** could be precipitated in 57% yield on a small scale and complex **4c** was found to be difficult to isolate due to its properties, vide infra.

**Figure 3:**
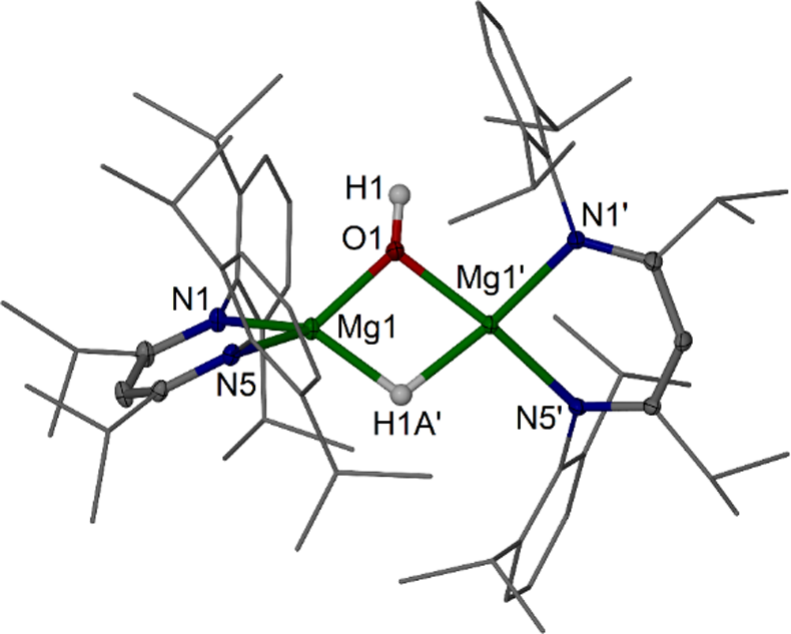
Molecular structure of [{(^iPrDip^nacnac)Mg}_2_(μ-H)(μ-OH)] **4c** (30% thermal ellipsoids, partial wireframe). Hydrogen atoms except H1 and H1A′, symmetry-disordered H/OH, and a second independent molecule are omitted for clarity. Selected bond lengths (Å) and angle (deg) of molecule 1: Mg1–O1 1.891(4), Mg1’–O1 1.955(4), Mg1–N1 2.0670(16), Mg1–N5 2.0653(16), Mg1–H1A 1.87(5), Mg1′–H1A 1.82(5), Mg1····Mg1 2.9294(12); Mg1–O1–Mg1′ 99.20(13).

Complexes **4a**, **4b** and **4c** could be structurally characterized (Figure 3 for **4c**, Figure S188 for **4a**, Figure S189 for **4b**) and in all cases show half molecules of **4** in their asymmetric units (**4c** provided two independent halves). The H and OH positions in **4a** are disordered by symmetry and two OH positions were found in close proximity in molecules of **4b** and **4c**. The Mg–OH and Mg–H bond lengths are unremarkable, but it is worth noting that their distances are of similar magnitude (ca. 1.9 Å). All molecules **4** show arrangements with bridging hydride and hydroxide ions between two distorted tetrahedral Mg centers, but the orientations of the β-diketiminate ligands, as judged by the relative twisting of two N···C(H)···N ligand planes (see the SI, Table S2), differ significantly. Complex **4a** shows a coplanar ligand arrangement whereas complexes **4b** (plane twisting angle 81.6°) and **4c** (88.0°, 89.6°, see Figure 3) show an approximate orthogonal arrangement of the ligand planes. The small changes in the remote ligand substituents in the series **4a** < **4b** < **4c** lead to noticeably more steric crowding around the central Mg_2_(μ-H)(μ-OH)-core (Figures S191 and S192). This situation is similar to that in the coplanar ligand arrangement in [{(^MeDip^nacnac)Mg(μ-H)}_2_]^56^ compared with the almost orthogonal (73.3°) ligand arrangement in [{(^tBuDip^nacnac)Mg(μ-H)}_2_]^57^ that was noted previously, due to the steric increase in the backbone substituents in the latter example.

After repeatedly applying freeze–pump–thaw degassing cycles to samples of complexes **4** in deuterated benzene (Scheme 2), complex **4a** showed no significant change (Figure S123). Degassing of complex **4b** afforded low conversion to the dehydrogenated oxide complex **1b** (Figure S124). Therefore, all solvent was removed from a sample of **4b**, and the residue was subjected to 8 h at 80 °C under vacuum. After redissolving, some 23% conversion to oxide **1b** was observed alongside unreacted **4b** (30%) and significant decomposition to dihydroxide [{(^EtDip^nacnac)Mg(μ-OH)}_2_] **3b** and proligand ^EtDip^nacnacH according to ^1^H NMR spectroscopy (Figure S125). The fraction of reformed **1b** could again be rapidly converted with H_2_ to **4b**. Adding hydrogen gas (ca. 1 bar) to a sample of **4b-***d*_**2**_ in deuterated benzene at room temperature slowly afforded resonances for the formation of **4b** in the NMR spectra, as well as showing very low levels of formation of HD alongside (Figures S128 and S129). Complex **4c**, however, was found to be unstable in solution when no hydrogen atmosphere was present and could be dehydrogenated in very high yield to oxide complex **1c** by repeatedly applying freeze–pump–thaw degassing cycles to the sample (Figures S126 and S127). Combining solutions of [{(^EtDip^nacnac)Mg}_2_(μ-H)(μ-OH)] **4b** and [{(^iPrDip^nacnac)Mg}_2_(μ-O)] **1c** in deuterated benzene did not lead to hydrogen scrambling (i.e., to form some **1b** and **4c**) at room temperature or 100 °C (Figure S105), which is in line with **4c** dehydrogenating more easily. The reaction between degassed solutions of **1b** and **4c** could not be performed due to the instability of **4c** in the absence of dihydrogen. Overall, these experiments have demonstrated the facile reversibility of this reaction and the strong influence of the backbone substitution on the reverse reaction.

There are a number of recently reported related low-coordinate early main group metal oxide complexes that demonstrated high reactivity. Anionic aluminum oxide and aluminum imide complexes derived from a potassium aluminyl species were found to slowly activate hydrogen at room temperature (oxide) or elevated temperature (imide), respectively, but no reversibility was reported.^58,59^ The former reaction also displaced THF from the Al center and afforded an aluminum(III) hydride-hydroxide species. Adding [2.2.2]cryptand to a related potassium complex of an anionic alumoxane at low temperature led to the intramolecular C–H addition of a ligand methyl group across the Al=O bond.^60^ Similarly, treatment of the aluminum(I) complexes [(^MeDip^nacnac)Al] and [(^tBuDip^nacnac)Al] with N_2_O led, depending on conditions,^61^ to a variety of aluminum(III) products, including the dimeric oxide [{(^MeDip^nacnac)AlO}_2_],^62^ hydroxide and hyponitrite complexes, and products of ligand C–H activation or solvent C–O cleavage reactions. This also provided a high-yielding C–H activation derivative of the putative monomeric alumoxane intermediate [(^tBuDip^nacnac)Al=O].^61^ Reaction of the related magnesium(0) complex [{(^tBuDipep^nacnac)MgNa}_2_] (Dipep = 2,6-(3-C_5_H_11_)-C_6_H_3_) with N_2_O provided a product mixture including a C–H activation product of the putative oxide intermediate [{(^tBuDipep^nacnac)MgONa}_2_] similar to those observed for the related aluminum species.^50^

**Scheme 3:**
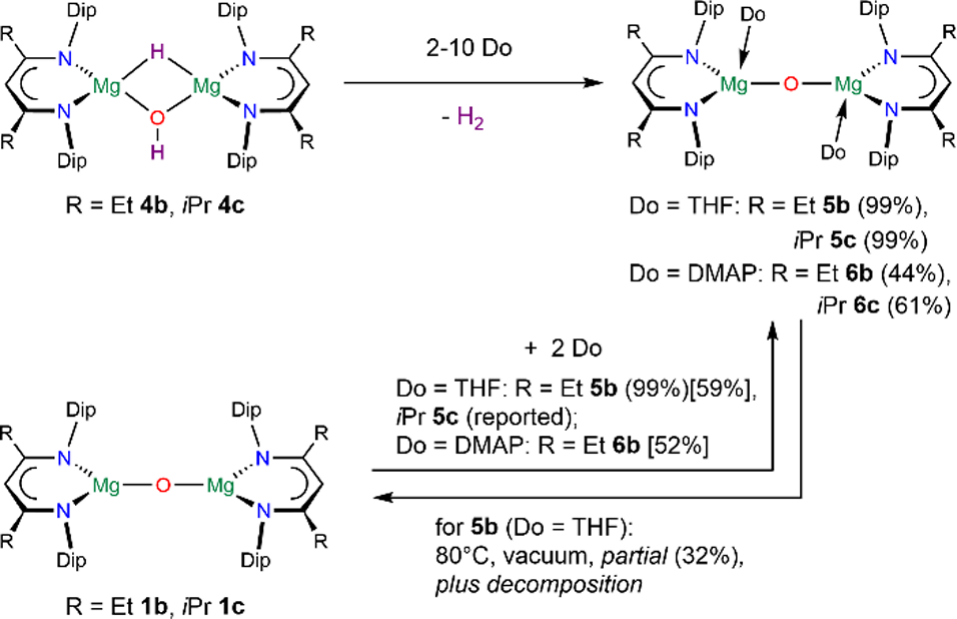
Donor-Induced Dehydrogenation of Complex **4b-c**

We treated [{(^RDip^nacnac)Mg}_2_(μ-H)(μ-OH)] **4b** and **4c** with the donors (Do) THF and DMAP (4-dimethylaminopyridine) in deuterated benzene (Scheme 3) and followed the progress of the reactions by ^1^H NMR spectroscopy (Figures S131–137). Upon THF addition, a gradual conversion of **4b** and **4c** to [{(^RDip^nacnac)Mg(THF)}_2_(μ-O)] **5b** and **5c** and hydrogen gas was observed when using between 2 and 10 equiv of THF. Slightly more THF is needed to dehydrogenate **4b** compared with **4c**, in accordance with the easier dehydrogenation of the latter. Complexes [{(^RDip^nacnac)Mg(THF)}_2_(μ-O)] **5b** and **5c** could be crystallized from these mixtures and were also independently obtained by addition of THF to oxide complexes **1b** or **1c** (Scheme 3).

**Figure 4:**
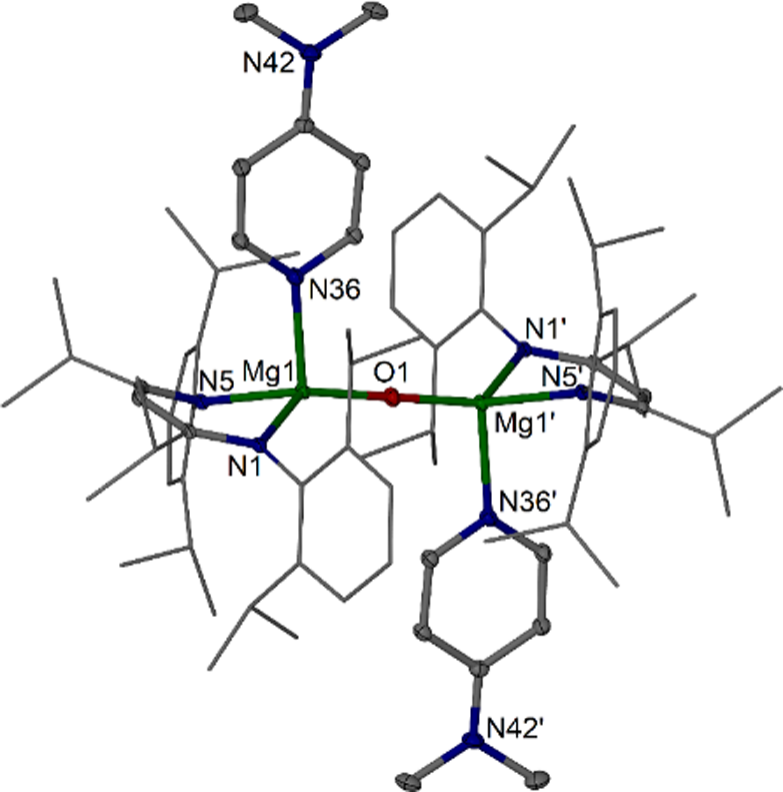
Molecular structure of [{(^iPrDip^nacnac)Mg(DMAP)}_2_(μ-O)] **6c** (30% thermal ellipsoids, partial wireframe). Hydrogen atoms are omitted for clarity. Selected bond lengths (Å) and angle (deg): Mg1–O1 1.8348(5), Mg1–N1 2.1375(14), Mg1–N5 2.0951(14), Mg1–N36 2.1798(14), Mg1····Mg1′ 3.6695(10); Mg1–O1–Mg1′ 180.00(2).

The molecular structure of **5b** is similar to those of **5a**(45) and **5c**.^47^ The stronger donor DMAP required fewer equivalents of donor addition to effect dehydrogenation and formed [{(^RDip^nacnac)Mg(DMAP)}_2_(μ-O)] **6b** and **6c** in an analogous manner (Scheme 3), but the DMAP reactions were also accompanied by more byproduct formation. Similar to the THF adduct complexes, **6b** and **6c** were easily afforded by adding DMAP to the uncoordinated oxides **1b** and **1c**, respectively, and DMAP could also displace THF in **5b** to afford **6b**. The molecular structure of **6c** (Figure 4) shows the expected features with a linear Mg–O–Mg unit and is overall comparable to those of **5a**–**5c** with only a slight elongation of the Mg–O bonds compared to those of **1c**.

Placing a sample of [{(^EtDip^nacnac)Mg(THF)}_2_(μ-O)] **5b** under vacuum at 60 °C for 4 h showed that the coordinating THF ligands could be removed forming 32% of **1b**, alongside significant decomposition (Figure S138). When substoichiometric quantities of THF were titrated into a solution of uncoordinated oxide **1b**, only one set of NMR resonances for the β-diketiminate ligands was observed, with a gradual shift of the ligand backbone resonance, which suggests the rapid interchange of THF ligands between Mg centers under these conditions (Figure S111).

**Scheme 4:**
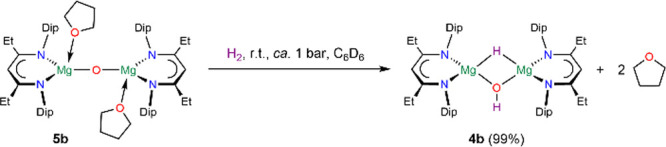
Hydrogenation of Complex **5b**

The reaction of isolated crystallized [{(^EtDip^nacnac)Mg(THF)}_2_(μ-O)] **5b** in deuterated benzene with hydrogen showed to our surprise that, according to ^1^H NMR spectroscopy, [{(^EtDip^nacnac)Mg}_2_(μ-H)(μ-OH)] **4b** and uncoordinated THF were formed (Scheme 4 and Figure S120). In one instance, crystals of **4b** were afforded from the sample. This is in line with our observation that an excess of THF is required to drive the dehydrogenation of complexes **4** to completion. In contrast, [{(^EtDip^nacnac)Mg(DMAP)}_2_(μ-O)] **6b** did not react with hydrogen in deuterated benzene to **4b** at room temperature or elevated temperatures, but the latter induced slow decomposition (Figure S122). This suggests that the lability of the THF ligands in **5b** allows the hydrogenation to take place, likely via [{(^EtDip^nacnac)Mg}(μ-O){Mg(THF)(^EtDip^nacnac)}].

**Scheme 5:**
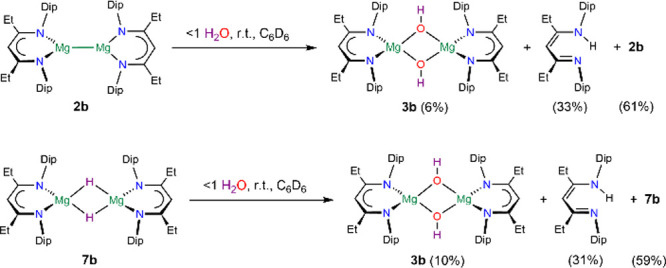
Hydrolysis of **2b** and **7b**

The reversibility of the system [{(^RDip^nacnac)Mg}_2_(μ-O)] **1** plus hydrogen to [{(^RDip^nacnac)Mg}_2_(μ-H)(μ-OH)] **4** emphasizes the possibility of accessing this system via an alternative synthesis of **4**. In principle, complexes **4** could be generated by the addition of one molecule of water to the magnesium(I) complexes [{(^RDip^nacnac)Mg}_2_] **2**. To test if this is viable, we studied the reaction of [{(^EtDip^nacnac)Mg}_2_] **2b** with substoichiometric amounts of water in a dilute solution in deuterated benzene followed by ^1^H NMR spectroscopy at room temperature (Scheme 5 and Figure S161). These reactions, however, did not show resonances for significant quantities of **4b** and the converted fraction of **2b** (39%) largely transformed to the symmetric dihydroxide **3b** (6%) and proligand ^EtDip^nacnacH (33%). Although we did not study low-temperature approaches, the absence of any **4b** makes a high-yielding synthesis via this method unlikely. Exploring other alternative routes to **4**, we have prepared the new hydride complex [{(^EtDip^nacnac)Mg(μ-H)}_2_] **7b** (see the SI, 1.2.2), in analogy to syntheses of **7a** and **7c**,^47,56,57^ as a suitable starting material and investigated its reaction with substoichiometric amounts of water at room temperature (Scheme 5 and Figure S162). Again, the converted fraction of **7b** (41%) forms dihydroxide **3b** (10%) and proligand ^EtDip^nacnacH (31%) but no significant quantities of **4b**. The reason for this selectivity, i.e., not forming significant quantities of **4b**, is currently not known, but could involve a more rapid hydrolysis reaction of intermediate **4b** compared with the hydrolysis rates of **2b** or **7b**, respectively. It is worth pointing out that solutions of **4b** do not dismutate to hydroxide **3b** and hydride **7b** under the conditions of the hydrolysis experiments. Alternatively, despite the dilute solution of water in benzene used in the experiment, the water molecules could be present and reacting as hydrogen-bonded water dimers,^63^ rather than water monomers, leading directly to **3b** and/or ^EtDip^nacnacH.

**Scheme 6:**
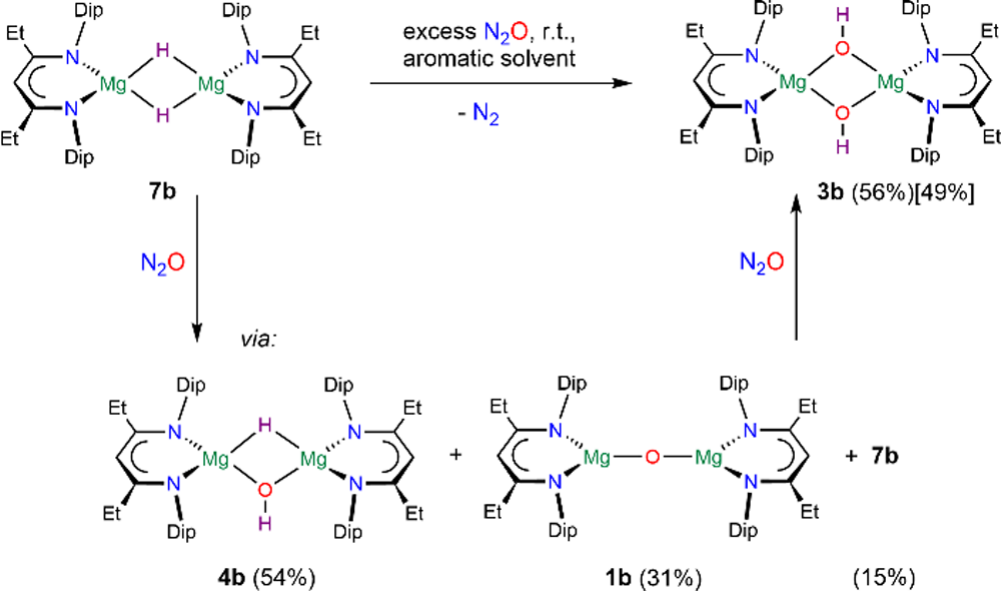
Synthesis of **3b** via **4b**

Hydroxide complexes of β-diketiminate alkaline earth metals and related species have been afforded via controlled hydrolysis experiments before.^41,64−68^ Previously, the Okuda group reacted the terminal magnesium (aluminum) hydride complex [(Me_3_TACD·Al*i*Bu_3_)MgH] (Me_3_TACD)H = 1,4,7-trimethyl-1,4,7,10-tetraazacyclododecane) with N_2_O and isolated the linear oxide complex [{(Me_3_TACD·Al*i*Bu_3_)Mg}_2_O] in good yield that was suggested to have formed by oxidation of the terminal hydride to the terminal hydroxide species as an intermediate and further reaction between hydride and hydroxide to afford the oxide product via hydrogen elimination.^51^ Oxide complex [{(Me_3_TACD·Al*i*Bu_3_)Mg}_2_O] was found to be unreactive toward excess N_2_O or hydrogen, however.^51^ We have reacted [{(^EtDip^nacnac)Mg(μ-H)}_2_] **7b** with small, likely substoichiometric quantities of N_2_O at room temperature followed by ^1^H NMR spectroscopy and could observe the partial conversion of **7b** to **4b** and even some **1b** (Figure S144). The mixture converted with further N_2_O in situ to afford dihydroxide **3b** (56%) as the main product (Scheme 6). Although the latter method is a promising avenue to access **4b**, reactions of oxides **1** with H_2_ have been superior for the preparation of complexes **4** when oxides are available. The dihydroxide **3b** could be synthesized on a preparative scale from hydride **7b** with N_2_O in 49% crystallized yield (Scheme 6).

**Figure 5:**
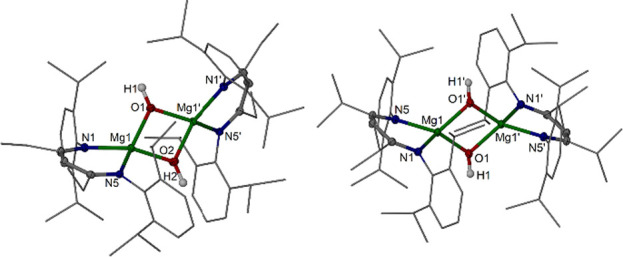
Molecular structure of [{(^EtDip^nacnac)Mg(μ-OH)}_2_] **3b**, left, and [{(^iPrDip^nacnac)Mg(μ-OH)}_2_] **3c**, right (30% thermal ellipsoids, partial wireframe). Hydrogen atoms except OH hydrogens, solvent molecules, and minor components of disorder are omitted for clarity. Selected bond lengths (Å) and angles (deg): **3b**: Mg1–O1 1.9293(14), Mg1–O2 1.9392(14), Mg1–N1 2.0744(16), Mg1–N5 2.0581(15), Mg1····Mg1′ 2.9751(11); Mg1–O1–Mg1′ 100.89(10), Mg1–O2–Mg1′ 100.18(10). **3c:** Mg1–O1 1.9613(11), Mg1–O1′ 1.9688(11), Mg1–N1 2.0946(12), Mg1–N5 2.1102(12), Mg1····Mg1′ 3.0188(9); Mg1–O1–Mg1′ 100.37(5).

The molecular structure of the donor adduct [{(^EtDip^nacnac)Mg(DMAP)(μ-OH)}_2_] **8b** shows a typical coplanar ligand arrangement for a complex with β-diketiminate ligands and five-coordinate Mg centers. Complexes **3b** and **3c** show well-resolved, sharp ^1^H NMR resonances for the ligand hydrogen atoms and typical upfield (δ −0.50 ppm for **3b**) resonances for the Mg(μ-O*H*) units. The well-resolved NMR resonances for **3b** and **3c** suggest that both coplanar and orthogonal ligand arrangements are accessible in solution. Rapid interconversion of various isomers is a typical property for these types of ionic complexes with highly flexible metal–ligand interactions. Thus, the two molecular structures of **3b** and **3c** can be viewed as snapshots of solution behavior. It may be surprising that slightly bulkier **3c** was afforded in the coplanar solid-state geometry whereas **3b** was afforded with the twisted (orthogonal) ligand orientation that allows shorter metal–ligand interactions and therefore likely represents a more (energetically) relaxed structure (Figure 5). The preference for an orthogonal ligand orientation may be slightly higher for bulkier **3c** compared with **3b**, when the results from the analysis of isomer geometries and energies of complex **4c** from DFT computational studies, vide infra, are considered.

**Scheme 7:**
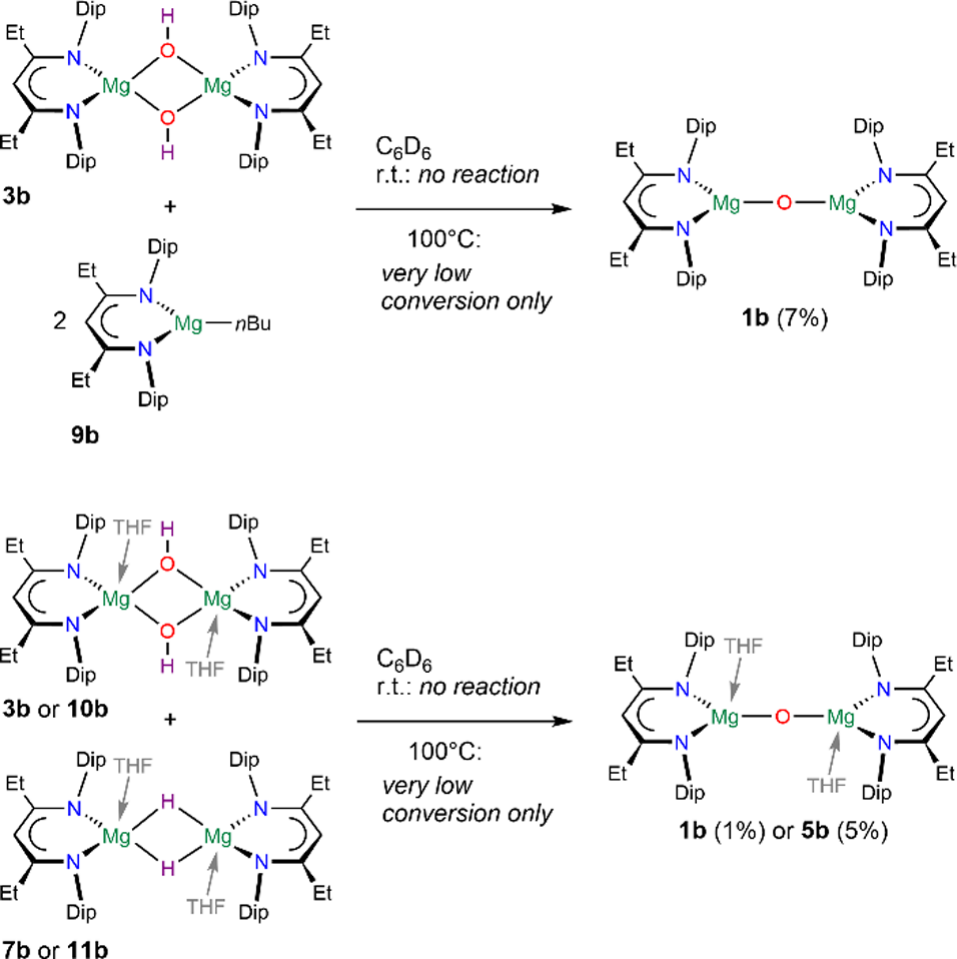
Alternative Synthetic Routes to Oxide Complexes **1b** and **5b**

To access alternative routes into oxides **1**, and in relation to Okuda’s oxide synthesis,^51^ we studied the reaction of [{(^EtDip^nacnac)Mg(μ-OH)}_2_] **3b** with the butyl complex [(^EtDip^nacnac)Mg*n*Bu] **9b** in deuterated benzene (Scheme 7 and Figures S150 and S151). The solid-state molecular structure of [(^EtDip^nacnac)Mg*n*Bu] **9b** was found to be monomeric (Figure S182), c.f. monomeric [(^iPrDip^nacnac)Mg*n*Bu],^47^ whereas a dimeric species was characterized in the solid state for [{(^MeDip^nacnac)Mg*n*Bu}_2_].^69^ However, no reaction between **3b** and **9b** was observed at room temperature or at 80 °C. At 100 °C, the complexes remained largely unreacted but showed low conversion (7%) to the oxide **1b** at 100 °C after 48 h (Scheme 7). Reacting [{(^EtDip^nacnac)Mg(μ-OH)}_2_] **3b** with [{(^EtDip^nacnac)Mg(μ-H)}_2_] **7b** in deuterated benzene similarly showed no reaction at room temperature or at 80 °C, i.e., no hydrogen elimination to [{(^EtDip^nacnac)Mg}_2_(μ-O)] **1b** or no ligand scrambling to [{(^EtDip^nacnac)Mg}_2_(μ-H)(μ-OH)] **4b**. At 100 °C, however, some trace conversion to the oxide **1b** after 105 h was observed (Scheme 7 and Figures S152 and S153). This also hints that complexes **7b** and **3b** do not dissociate into monomeric species under these conditions to a significant extent, which would be expected to form an oxide complex more readily in relation to Okuda’s oxide synthesis.^51^

It is expected that both the aggregation state and reactivity of MgH and MgOH complexes is highly dependent on ligand sterics and metal coordination number; the latter can be modulated by donor molecules, see for example the donor-induced dehydrogenation of complexes **4b** and **4c** in Scheme 3. Adding THF to a mixture of **3b**, to afford [{(^EtDip^nacnac)Mg(THF)(μ-OH)}_2_] **10b** in situ, and **7b** to afford [{(^EtDip^nacnac)Mg(THF)(μ-H)}_2_] **11b** in situ, showed no formation of oxide complex **5b** at room temperature, nor formation of **4b** or a possible donor adduct (Scheme 7). Heating the mixture of **10b** and **11b** to 100 °C, however, slowly afforded resonances for the oxide complex **5b** (ca. 5% after 63 h) albeit with some decomposition products alongside (Figures S152 and S153). A few crystals of **5b** could be obtained from this procedure. A similar study with the stronger donor DMAP instead of THF, between hydride complex [(^EtDip^nacnac)Mg(DMAP)H] **12b** of monomeric structure (Figure S186), c.f. related [(^tBuDip^nacnac)Mg(DMAP)H],^57^ and hydroxide complex [{(^EtDip^nacnac)Mg(DMAP)(μ-OH)}_2_] **8b** (Figure S198), showed low levels of conversion at 60 °C to afford some [{(^EtDip^nacnac)Mg(DMAP)}_2_(μ-O)] **6b** (ca. 6% after 23 h) but largely formed a product mixture suggesting decomposition (Figures S159 and S160). Previously, the related reaction of [{(^MeDip^nacnac)Ca(THF)(μ-OH)}_2_] with [{(^MeDip^nacnac)Ca(THF)(μ-H)}_2_] at 85 °C in benzene overnight did not afford a calcium oxide complex or an anion-scrambled complex [{(^MeDip^nacnac)Ca}_2_(μ-H)(μ-OH)] and the starting materials were found to be unchanged.^66^ Thus, these high-temperature approaches can provide low levels of magnesium oxide products but have not been deemed convenient as an alternative route for this ligand framework, especially when considering that oxide complex products are expected to show high reactivity.

**Scheme 8:**
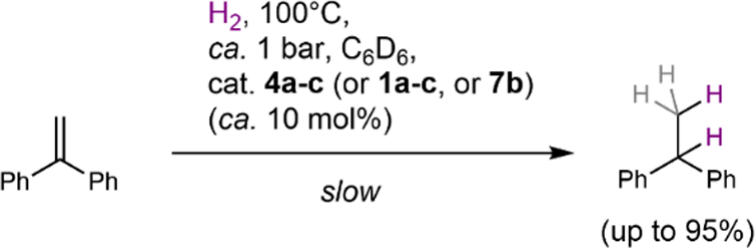
Catalytic Hydrogenation of DPE

Because the magnesium oxide (**1**)/hydride-hydroxide (**4**) system was found to be capable of reversibly activating and binding hydrogen, we were interested in the possibility of catalytic substrate hydrogenations. As an initial proof-of-concept reaction, we used ca. 10 mol % of the oxide complexes **1a**–**c** to access **4a**–**c** via reaction with hydrogen to study the catalytic hydrogenation of 1,1-diphenylethene (DPE) with hydrogen (ca. 1 bar) heated at 100 °C (Scheme 8) in deuterated benzene. This showed slow conversion of DPE to 1,1-diphenylethane (Scheme 8) under these conditions hinting at a promising approach to hydrogenation catalysis. Qualitative rates decrease **4a** > **4b** > **4c** and shows that the less bulky system is more competent in hydrogenations for steric reasons which is opposed to the trend for dehydrogenation of **4**, a process likely induced by sterics, vide infra. Although conversion rates are slow (47% yield after 221 h, 95% yield after 700 h, turnover frequency of 9.7 × 10^–3^ h^–1^ for **4a**), an initial experiment with a slightly higher hydrogen pressure indicated a significant pressure dependence and improvement (Figure S165). For comparison, we carried out DPE hydrogenation using catalytic amounts of magnesium hydride complex **7b** using the same conditions (ca. 1 bar, 100 °C) and found a broadly similar activity compared with complexes **4**. Reactions of **7b** with DPE in the absence of hydrogen gas, or with insufficient hydrogen pressure, showed the formation of a main hydromagnesiation (hydromagnesation) intermediate, [(^EtDip^nacnac)Mg{CPh_2_CH_3_}], as suggested by NMR spectroscopy (Figures S170–S177). When **4b** and DPE were reacted stoichiometrically in the absence of hydrogen gas, no formation of a hydromagnesiation product was observed, but instead, significant amounts of oxide complex **1b** and only trace amounts of 1,1-diphenylethane had formed (Figure S167). These experiments indicate that the DPE hydrogenation pathway may be mechanistically different between reactions catalyzed by complexes **4** and hydride complex **7b**, and that both MgOH and MgH moieties may be involved in hydrogenation by the former complexes. When the catalyst activity is considered, calcium-based examples are known as hydrogenation catalysts for this substrate and several show a higher activity for this reaction.^26,32,70−76^

### Computational Studies

**Figure 6:**
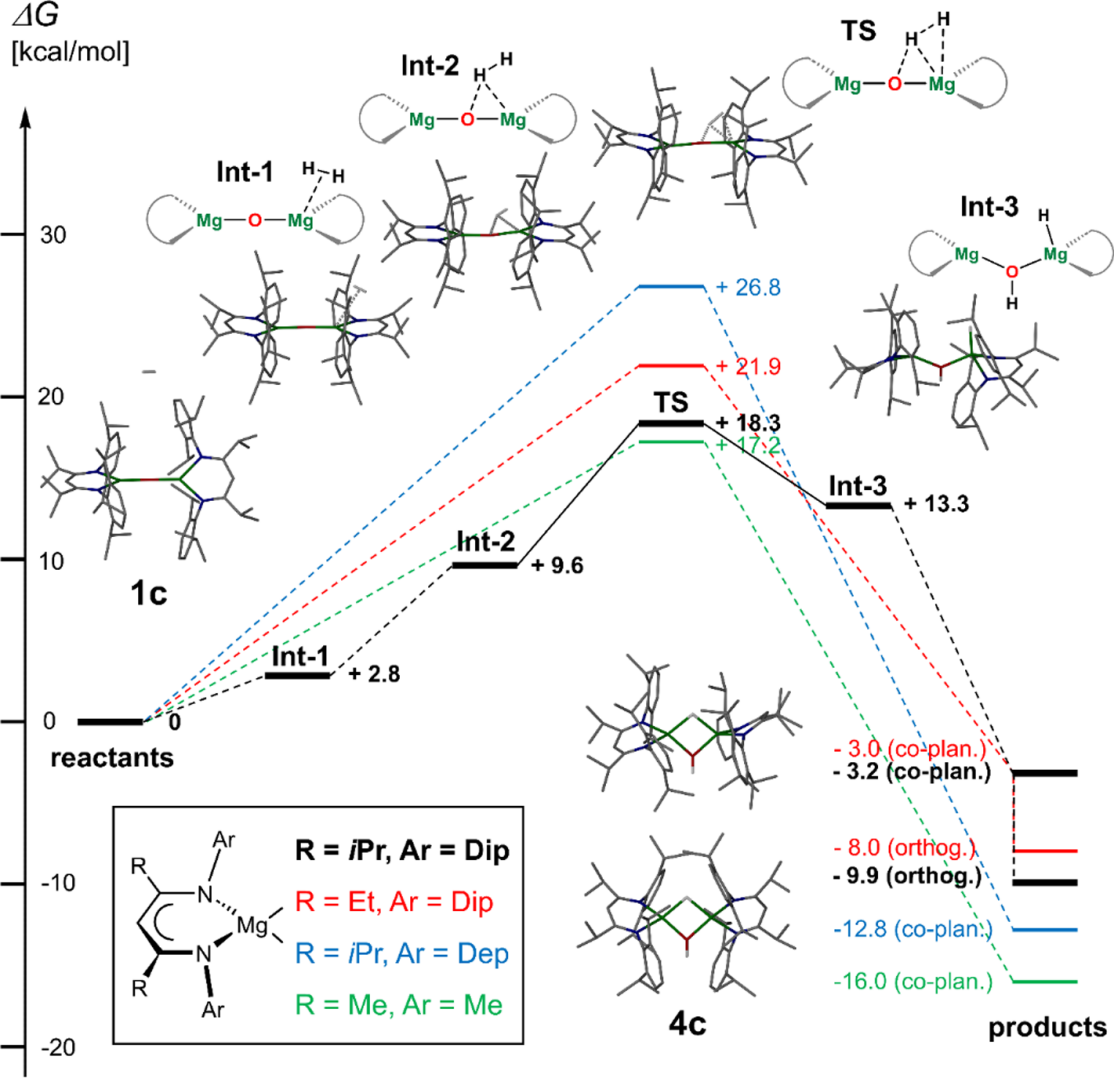
Energy profile (Δ*G* in kcal/mol) for the reaction of [{(^RAr^nacnac)Mg}_2_(μ-O)] with hydrogen computed at the M06-D3_PCM(benzene)_/def2-TZVP//M06-L-D3/def2-SVP level of theory. Dashed lines represent unconnected compounds on the energetic path of the reaction profile whereas solid lines represent a connected path on the reaction coordinate via a transition state.

To shed light on the mechanistic key step and reversibility of the hydrogen activation reaction we have performed DFT computational studies for the reactions of **1b** and **1c** with H_2_ to form **4b** and **4c**, respectively, at the M06-D3_PCM(benzene)_/def2-TZVP//M06-L-D3/def2-SVP level of theory, plus for two model complexes with smaller substituents on the ligands, i.e., of [{(^iPrDep^nacnac)Mg}_2_(μ-O)] (Dep = 2,6-diethylphenyl) and [{(^MeMe^nacnac)Mg}_2_(μ-O)]. The reversible system with the bulkiest ligand (**1c** to **4c**) was studied in most detail and the computed energy profile for the proposed mechanistic pathway is shown in Figure 6, including key energy data (transition states, products) for the other ligand modifications. For the reaction of **1c** with dihydrogen (bold black lines), two endergonic, weak dihydrogen adducts of **1c** (**Int-1**, **Int-2**) could be identified. The transition state (**TS**) for the heterolytic dihydrogen cleavage at the low-coordinate Mg–O interaction is with +18.3 kcal/mol low enough to support the facile reaction at room temperature. The newly formed terminal Mg–H and bridging Mg–OH–Mg moieties in **Int-3**, plus two similar isomers **Int-4** and **Int-5** (Figure S202), can undergo minor ionic rearrangement to yield product **4c**. Using strain analysis, where we considered changes to the magnesium complex geometries minus the dihydrogen fragment for **Int-2**, **TS** and **Int-3**, shows, as expected, that very little rearrangement energy is required from **Int-2** to **TS** (1.8 kcal/mol). In line with the dihydrogen cleavage and the associated changes in geometry, a large energy change is, however, required to account for changes from **TS** to **Int-3** (30.8 kcal/mol). The isomer of **4c** with orthogonal ligand arrangement, c.f. the molecular structure in Figure 3, is exergonic by −9.9 kcal/mol compared to the starting materials and −6.7 kcal/mol lower in energy than **4c** with an approximate coplanar ligand set. Inspecting the mean bond lengths between orthogonal **4c** and coplanar **4c** (Figure S205), shows that the orthogonal geometry allows slightly shorter metal–ligand interactions and thus offers more electrostatic stabilization as a likely reason for this preference and is a direct result of the employed ligand bulk. The formation of complex **4c** is only exergonic by −9.9 kcal/mol and the reverse reaction (dehydrogenation) thus shows an activation barrier of +28.2 kcal/mol, but only +21.5 kcal/mol from its coplanar isomer, which is accessible at room temperature based on considerations around the X-ray structures of the closely related hydroxides **3b** and **3c** (Figure 5) and their NMR spectra.

The hydrogenation of **1b** (red lines) shows a similar overall profile but with slightly higher barriers for the hydrogenation (+21.9 kcal/mol) and dehydrogenation (+29.9 kcal/mol) reaction, which supports the trend of the more challenging experimental dehydrogenation of **1b** compared with **1c**. Slightly lowering the steric profile by changing the aryl substituents from Dip to Dep, i.e., using [{(^iPrDep^nacnac)Mg}_2_(μ-O)] for the hydrogenation (blue lines), shows a higher energy transition state (+26.8 kcal/mol) and formation of a more exergonic product that would not allow a facile reverse reaction (barrier for dehydrogenation: +39.6 kcal/mol).

Of further note is the preference for the product geometry with coplanar ligand arrangement for [{(^iPrDep^nacnac)Mg}_2_(μ-H)(μ-OH)] which we could not optimize in the orthogonal geometry, whereas the preferred structures for **4b** and **4c** show lower-lying orthogonal isomers, as were found in their solid-state structures, vide supra. Here it is worth remembering that compound **4a** with a coplanar solid-state structure did not easily dehydrogenate. Computing the hydrogenation for sterically unencumbered [{(^MeMe^nacnac)Mg}_2_(μ-O)]^55^ (green lines), i.e. only hosting methyl groups on the nitrogen atoms and backbone positions, shows a relatively low hydrogenation barrier (+17.2 kcal/mol) but a highly exergonic product and thus a high barrier for dehydrogenation (+33.2 kcal/mol). These computations show that the steric profile relating to bulk and shape strongly influences the thermodynamics and kinetics of the system. It appears that, for the studied β-diketiminate systems, the steric bulk is required to destabilize the product and can help lowering the barrier for dehydrogenation, which requires significant distortion. This may be linked to the ease of distorting the product isomer with coplanar β-diketiminate ligand planes to the isomer with approximate orthogonal arrangements of ligand planes (−5.0 kcal/mol for **4b**, −6.7 kcal/mol for **4c**).

The previously mentioned study from Okuda and co-workers suggested that their monomeric magnesium hydride complex and the corresponding monomeric magnesium hydroxide complex can combine to a bridging oxide complex plus dihydrogen.^51^ Thus, the possibility of the dehydrogenation reaction occurring via monomeric complexes was computationally studied for **4c**. This shows, however, that +38.6 kcal/mol are required to dissociate **4c** into monomeric [(^iPrDip^nacnac)MgH] and [(^iPrDip^nacnac)MgOH], which is significantly higher than the activation barrier for dehydrogenation via the determined pathway and was thus deemed unfavorable. That reactions via monomeric complex entities are unlikely in this case can also be inferred from the reaction of **3b** with **7b**, which did not lead to significant amounts of **1b** (Scheme 5).

**Figure 7:**
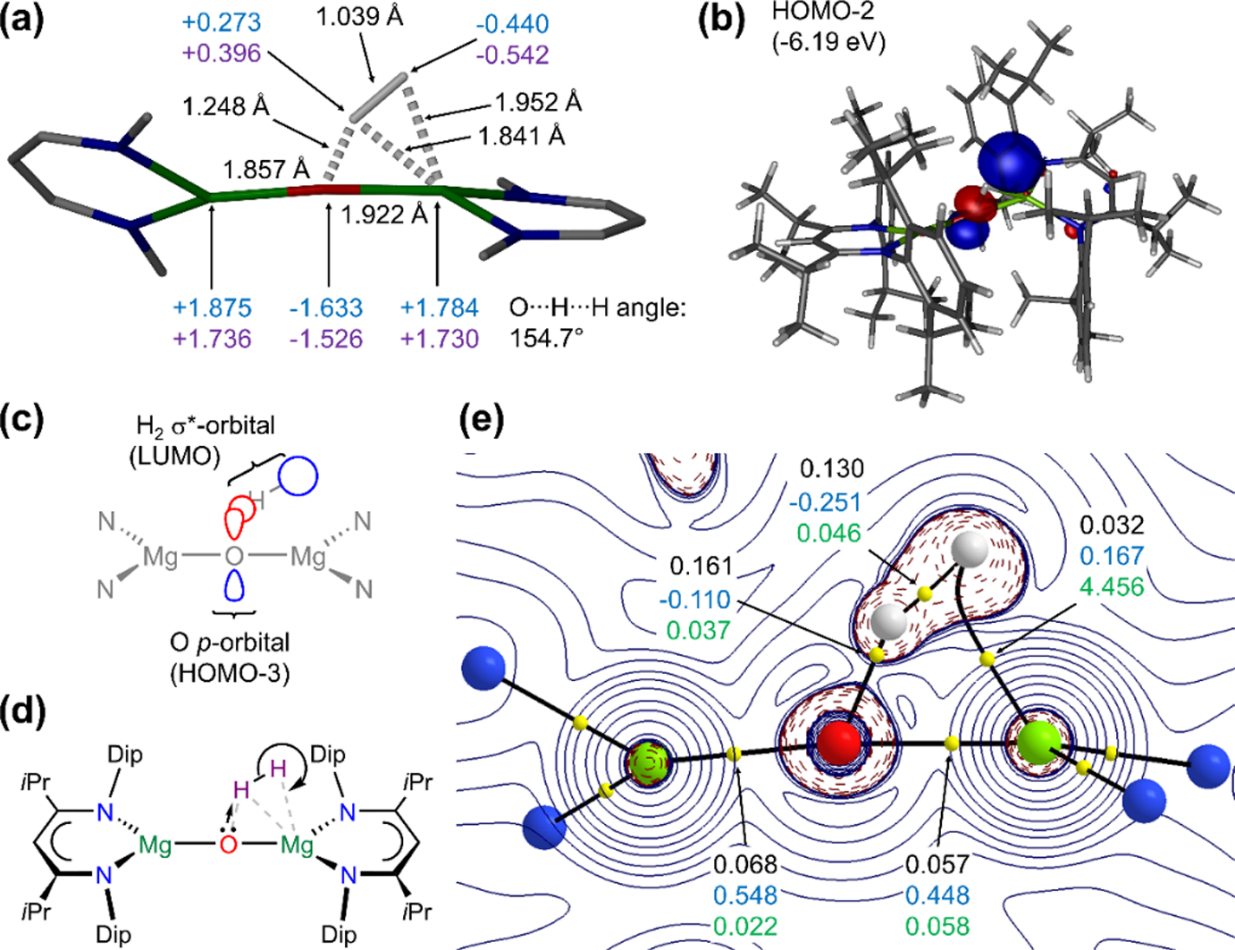
Computational analysis and interpretation of the transition state of [{(^iPrDip^nacnac)Mg}_2_(μ-O)-H_2_] (Mg green, O red, N blue, C dark gray/black, H light gray): (a) bond lengths (black) and calculated charges (NPA: top, in blue, QTAIM: bottom, in purple) of the core of the transition state; (b) HOMO-2 (isovalue 0.06), the HOMO and HOMO–1 are ligand-based; (c) simplified orbital sketch of the HOMO-2 shown in (b); (d) simplified arrow-pushing drawing of the S_N_2-like heterolytic hydrogen-cleavage; (e) QTAIM contour plots of the Laplacian of electron density (solid lines positive, dashed lines negative) through the Mg–O–H_2_ plane showing only the core atoms, bond paths (black) and bond critical points (yellow). Values for the electron density, ρ(*r*) (top, in black, in e/bohr^3^), Laplacian, ∇^2^ρ(*r*) (middle, in blue, e/bohr^5^), and bond ellipticity, ε (bottom, in green), are given for selected bond critical points.

An analysis of the transition state of the reaction (Figure 7) displays the key interatomic distances and calculated charges (a), the key orbital interaction in the HOMO–2 (b), and an interpretation of the HOMO–2 (c). The geometry of the transition state is close to that of the free oxide **1c** with close association of a H_2_ unit with an elongated (+38%) H–H bond that sits end-on bound at the oxide and side-on coordinated to an Mg center (a). The orbital interaction and sketch (b,c) suggest overlap of a high-lying oxide *p*-orbital lone pair, previously identified as the HOMO–3 in the computed oxide species **1c**,^55^ with the σ*-orbital (LUMO) of the dihydrogen molecule. The largest orbital lobe (blue in (b) and (c)) is located near spherically around the terminal hydrogen atom that holds significant anionic charge (a) and is forming the Mg^2+^-coordinated hydride ion. The hydrogen atom with the short contact to the oxide center carries significant positive charge to form the hydroxide proton. This can be summarized as an S_N_2-type nucleophilic attack of an oxide lone pair on the dihydrogen unit heterolytically cleaving the H–H bond and forming a hydride ion as a leaving group in the coordination sphere of an Mg^2+^ center (d). Additionally, it can be viewed as a deprotonation of H_2_ by a strong oxide base. Figure 7e shows the Laplacian of the electron density (∇^2^ρ(*r*)) from QTAIM analysis, including bond paths and relevant bond critical points (bcps) with selected values. The H_2_ unit is in an area of negative Laplacian (e) and the anionic charge accumulation is also apparent from the overall charge in the H_2_ unit (−0.167 from NPA, −0.146 from QTAIM, (a)). The bcps between H and O and between both H atoms also show a high electron density. Both these bcps are closest to the more positively charged hydrogen atom showing the polarization toward the heterolytic cleavage. Only the terminal hydrogen atom shows a bond path to the coordinating Mg center. This bond path is quite bent with a large bond ellipticity at the associated bcp, whereas other bcps show low bond ellipticities. A study of the noncovalent interactions in the transition state (Figure S209) agrees qualitatively well with the QTAIM analysis.

Overall, the mechanistic aspects of the heterolytic dihydrogen cleavage by complexes **1** show similarities to those found for FLPs^10,11,77^ and in some alkaline earth metal complexes with polar ligands.^17,19,26^ Heterolytic dihydrogen cleavage can also be achieved by electrophilic transition metals,^3,4^ albeit without assistance from *d*-orbitals in the presented case. A complex with largely ionic metal–ligand interactions allows for comparably facile geometric reorganization to facilitate the reverse reaction that requires bringing proton and hydride into close proximity. We have shown that the latter process is strongly influenced by the supporting ligands sphere. The facile dehydrogenation of **4c** competes with the most competent examples in FLP chemistry which require longer periods at room temperature for dehydrogenation.^10,11,77,78^

## Conclusions

We have presented the facile and rapid hydrogenation of well-defined, low-coordinate magnesium oxide complexes to mixed hydride-hydroxide complexes, [{(^RDip^nacnac)Mg}_2_(μ-H)(μ-OH)] **4** (R = Me **4a**, Et **4b**, *i*Pr **4c**), using molecular dihydrogen at room temperature under atmospheric pressure. Mechanistically, an oxide lone pair nucleophilicly attacks a H_2_···Mg σ-complex in an S_N_2-like manner that induces a heterolytic dihydrogen cleavage by adding electron density to the σ* H_2_-orbital forming a hydride ion coordinating to Mg on the distant hydrogen and adding a proton to the oxide. This is followed by ionic rearrangement to the final product geometries, which, for the bulkiest examples studied, is only mildly exergonic. The reaction was found to be reversible under mild conditions for **4c**, and likely proceeds via the reverse of the discussed mechanism, i.e., by distorting the ionic structure until the H^+^ and H^–^ units are in close proximity, and not via free monomers. Dehydrogenation of complexes **4** could also be induced by adding donor molecules such as THF or DMAP. This latter process likely enables dehydrogenation by more facile complex reorganization and weaker coordination bonds. Thus, both physical and chemical methods have been employed to dehydrogenate **4c**. Alternative syntheses to mixed hydride-hydroxide and oxide complexes have been explored and show that other routes are possible, for example by reacting magnesium hydride complexes with nitrous oxide, but that dihydrogen addition to well-defined oxide complexes is currently the preferred method for this system.

We have found significant differences in dehydrogenations of complexes **4**, despite only small modifications in a remote part of the ligand; complex **4a** showed no room temperature dehydrogenation whereas **4c** underwent full dehydrogenation. This demonstrates the large influence of minor changes to the ligand environment. The required distortion to effect dehydrogenation appears to be broadly linked to the preference for a complex geometry with an orthogonal ligand plane orientation governed by sterics, in contrast to a complex with coplanar ligand arrangement. The ligand bulk and shape in example **4c** appear to keep the complex in a relatively high-lying energetic coordination geometry but is still unencumbered enough not to lock the complex in a fixed position that prevents reorganization toward the transition state geometry and allows facile dehydrogenation. This is supported by flexible ionic metal–ligand interactions in these *s*-block metal complexes that are generally easy to distort. This situation is broadly reminiscent of the entatic state concept in (metallo)enzymes that ensures relatively low activation energies are realized for catalyzed reactions from the respective resting states of the (metallo)enzymes, or indeed, as found in some coordination compounds.^79^ Once again, the modification of the fine steric balance of sterically demanding alkaline earth metal complexes has led to significant changes in product stability and reactivity. Low-coordinate oxide complexes should be able to (reversibly) activate many other inert chemical bonds and provide a new approach to (hydrogenation) catalysis.

## Data Availability

The research data (NMR spectroscopy, DFT computational studies) supporting this publication can be accessed at 10.17630/41208338-2fc7-4bb9-8074-4d9b783cfdab

## Supporting Information Available

Experimental section, NMR spectra, X-ray crystallography, and DFT computational studies (PDF)
