# Supplementary material for: Facile, Reversible Hydrogen Activation by Low-Coordinate Magnesium Oxide Complexes
Source: J Am Chem Soc. 2025 Jan 29;147(6):5247–57. doi: 10.1021/jacs.4c16041 (PMC11826877; doi:10.1021/jacs.4c16041)
Supplement: Supplementary file 1 — ja4c16041_si_001.pdf [file ja4c16041_si_001.pdf]

Supporting Information

for

Facile, reversible hydrogen activation by low-coordinate magnesium oxide complexes

Samuel Thompson, Stuart Burnett, Rochelle Ferns, Tanja van Mourik, Aidan P. McKay, Alexandra M. Z. Slawin, David B. Cordes, and Andreas Stasch*

EaStCHEM School of Chemistry, University of St Andrews, North Haugh, St Andrews, KY16 9ST, United Kingdom.

Table of Contents

1	Experimental Section	1
2	NMR spectroscopy	32
3	X-ray crystallography	210
4	DFT Computational studies	240
5	References	316

1 Experimental Section

1.1 General considerations

All manipulations were conducted using standard Schlenk and glove box techniques under a dry argon or dinitrogen atmosphere. Benzene, toluene, tetrahydrofuran, *n*-hexane, cyclohexane, and diethyl ether were either dried and distilled under inert gas over lithium aluminium hydride and stored over molecular sieves, or taken from an MBraun solvent purification system and degassed before use.^{1}H, ^{2}H, and ^{13}C{^{1}H} NMR spectra were recorded on a Bruker AVII 400, Bruker AV III 500 or Bruker AVIII-HD 700 spectrometers in deuterated benzene and were referenced to the residual ^{1}H or ^{13}C{^{1}H} NMR resonances of the solvent used. Chemical shifts are given in ppm. Abbreviations: s = singlet, d = doublet, t = triplet, q = quartet, sept = septet, br = broad, vbr = very broad and m = multiplet. Many small-scale reactions were carried out in 5 mm (mostly 7-inch length) NMR tubes with a J Young top. Many of these small-scale reactions are described in the NMR section (Section 2) in the form of stacked NMR spectra with some experimental context in the figure captions. IR spectra were obtained on a Shimadzu IR Affinity spectrometer with ATR attachment. Melting points were determined using a Gallenkamp melting point apparatus in sealed glass capillaries under argon and are uncorrected. Elemental analyses on some selected compounds were performed by the Elemental Analysis Service at London Metropolitan University. Proligand ^{EtDip}nacnacH[1] and complexes [{(^{MeDip}nacnac)Mg}$_2$(μ-O)] **1a**,[2] [{(^{MeMes}nacnac)Mg}$_2$],[3] [{(^{iPrDip}nacnac)Mg}$_2$(μ-O)] **1c**, [{(^{iPrDip}nacnac)Mg}$_2$] **2c**, and [{(^{iPrDip}nacnac)Mg(THF)}$_2$(μ-O)] **5c**[4] have been synthesised and described before. Complexes with the ^{MeDip}nacnac ligand have compound numbers ending with **a**, with the ^{EtDip}nacnac ligand ending with **b**, and with the ^{iPrDip}nacnac ligand ending with **c**.

1.1.1 Comments around ligand choice and solubility properties of the complexes

The majority of investigations were conducted with the (^{EtDip}nacnac)Mg (**b** complexes) and (^{iPrDip}nacnac)Mg (**c** complexes) complex fragments that are described in the next sections. For small-scale *in-situ* reactions in 5 mm J Young NMR tubes followed by (^{1}H) NMR spectroscopy, we have predominantly chosen the (^{EtDip}nacnac)Mg fragment to ensure good solubility of all reaction constituents and thus allow representative analysis by NMR spectroscopy. Some (relative) solubility trends are given below.

[{(^{EtDip}nacnac)Mg}$_2$(μ-O)] **1b** has been found to be highly to extremely soluble in all employed hydrocarbon solvents and it could not be crystallised from toluene, benzene, *n*-hexane, *n*-pentane, or cyclohexane so far. At high concentrations at room temperature, compound **1b** could not be precipitated, but at low temperatures (e.g. freezer, −40°C) the complex could be partially precipitated from some solvents as a fine white powder.

[{(^{EtDip}nacnac)Mg}$_2$(μ-H)(μ-OH)] **4b** is less soluble compared to the respective oxide precursor **1b** in C_6D_6. After the addition of dihydrogen to **1b**, a colourless solution remains and upon standing overnight, complex **4b** crystallised out. Complex **4b** is also less soluble in *n*-hexane. Dissolving the oxide complex **1b** in *n*-hexane and adding H_2, produced a white precipitate of **4b** after shaking plus a saturated colourless solution of the product in *n*-hexane.

[{(^{iPrDip}nacnac)Mg}$_2$(μ-O)] **1c** is only poorly soluble in C_6D_6. After the addition of N_2O to [{(^{iPrDip}nacnac)Mg}$_2$] **2c**, **1c** immediately formed as a white precipitate that was found to be sparingly soluble at higher temperatures (approx. 15 mg could be dissolved in 0.6 mL of C_6D_6 at ca. 100°C).

[{(^{iPrDip}nacnac)Mg}$_2$(μ-H)(μ-OH)] **4c** is highly soluble in C_6D_6. After the addition of H_2 to the respective oxide complex **1c**, the solubility of the system increased, and the complex did not crystallise from the solution at room temperature.

1.1.2 General Procedure for Drying Gases

The extreme moisture sensitivity of the oxide complexes **1** and the tendency to form hydroxide complexes had been mentioned previously.[2,4] Thus, it was very important to work with dried gases. Gases (N_2O and H_2) were handled at near atmospheric pressure with relatively low flow from standard gas cylinders.

The following set-up and procedure was followed to dry gases at atmospheric pressure over molecular sieves: A J Young flask (500 mL volume) had molecular sieves added (3 Å, ca. 2-3 cm from the bottom, pre-dried overnight at ca. 130°C for at least 18 hours) and was connected to the Schlenk line (two-bank manifold vacuum/dinitrogen gas).[5] The flask was heated (to ca. 300°C) with a heat gun whilst under full vacuum until a constant vacuum reading was reached before allowing it to cool to room temperature and carefully refilling it with pre-dried dinitrogen (ca. 1 bar). This process was repeated two more times. After the last cycle of heating the flask under vacuum, the flask was first allowed to cool to room temperature, before being cooled to −80°C (cold bath to immerse more than half of the flask) and then sealed, i.e., the flask was not refilled with pre-dried dinitrogen. The tubing to the sealed flask was then filled with pre-dried dinitrogen (ca. 1 bar). The flask and the cylinder of desired gas were then connected via tubing and a gas-connector T-piece to one tubing outlet on the Schlenk line. This tubing setup can be evacuated and filled with dinitrogen gas (cycle, multiple times). The tubing system was then opened to the nitrogen system of the Schlenk line, and the nitrogen flow was stopped. The gas cylinder was now opened at a low flow and pressure, allowing the gas to flow through the tubing and out through the bubbler of the Schlenk line set-up (ensuring that pressurised gas is not flowing into a closed apparatus!). The J Young flask was now slowly and carefully opened, allowing the flask to fill with ca. 1 bar of gas before being sealed. The gas cylinder was then closed, the tubing evacuated and refilled with pre-dried dinitrogen (ca. 1 bar). The flask was allowed to warm

to room temperature (pressure change to approximately up to 1.2-1.5 bar). The gas was allowed to stand in the flask over the molecular sieves overnight (for ca. 18 hours at room temperature) before being used the next day. An evacuated tubing set-up between the flask containing the gas, the evacuated and sealed reaction flask or NMR tube, all connected via tubing and a gas-connector T-piece to one Schlenk line tap, allowed the dried gas to fill the reaction flask or NMR tube at approximately atmospheric pressure. For that, the tap to the Schlenk line was closed, the tubing and reaction flask/NMR tube were held under vacuum, and the pressure was equilibrated by opening the flask with the dry gas to the evacuated reaction flask/NMR tube and tubing volume. The receiving tube/flask under vacuum can be externally cooled (for example, NMR tubes for hydrogen reactions had their bottom half cooled to add enough gas). After brief equilibration, all flasks were closed. Depending on the size of the reaction flask or NMR tube and evacuated tubing volume, the combined volume may be below atmospheric pressure, the equilibration may not backfill these volumes to 1 atmosphere, and a brief topping up with dinitrogen gas from the Schlenk line may be needed (giving a H_2/N_2 mixture). Heating the sealed NMR tubes or flasks will create more than 1 bar of pressure (consider $pV = nRT$), but the gas volumes are small, and a proportion of the gas reacts. The number of equivalents of (reagent) gas may be estimated from the gas phase volume in the reaction flask or NMR tube. Typically, some excess of a reagent gas is in practice needed to reach the completion of a reaction. Some small-scale reactions may require a second filling of gas.

For the alkene hydrogenation, the NMR tubes were filled with hydrogen gas as described above where the storage flask was filled at approximately −80°C and the NMR tube was cooled to −196°C when the gas was equilibrated into the NMR tube. To estimate the pressure for this filling method, the released gas compared to atmospheric pressure was estimated as ca. 0.2-0.3 mL for a tube that has approximately 2.6 mL gas phase above the solvent level; this suggested an approximate pressure of 1.1 bar. The gas volume of 2.6 mL suggested approximately 0.10-0.12 mmol hydrogen gas, which was approximately 2-3 equivalents only based on the alkene, and some will be consumed during the reaction.

Note on heating NMR tubes: For some reactions, the solutions in NMR tubes were heated, e.g. to 100°C. Only the solvent level, and not the gas phase above, was heated, but the internal pressure will increase with heating. Assuming the whole tube would be heated to 100°C, an internal pressure of up to 1.25 could be reached (from 1 bar, or more if some cooling was applied to the tube while filling and sufficient pressure was available via the storage gas flask).

Please note (caution!): When cooling a flask or tube for filling with a gas, ensure that the flask is kept above the boiling point of the respective gas (e.g. nitrous oxide boiling point = −89°C, dihydrogen boiling point = −259°C) to avoid condensing the gas into the flask or tube that is to be sealed - risk

of explosion upon (minimal) warming! Similarly, be aware of the physical properties of argon (boiling point = −186°C, melting point = −189°C) if using this inert gas instead of dinitrogen.

1.2 Syntheses

1.2.1 Synthesis of [{(^{EtDip}nacnac)Mg}$_2$] 2b and precursors

These complexes have been prepared by analogy to previously reported related derivatives.[3,4,6]

[(^{EtDip}nacnac)Mg(OEt$_2$)I]

A solution of iodomethane (2.28 mL, 36.6 mmol, 1.15 equiv.) in diethyl ether (ca. 15 mL) was added over 20 minutes with stirring to magnesium powder (1.16 g, 47.7 mmol, 1.50 equiv., activated by the addition of one small iodine crystal) in diethyl ether (ca. 15 mL). The suspension was stirred and heated under reflux until most of the magnesium powder was consumed (ca. 1 hour). The resultant mixture was allowed to cool to room temperature and then added to a −30°C solution of ^{EtDip}nacnacH (14.2 g, 31.8 mmol, 1.00 equiv.) in diethyl ether (ca. 15 mL). The solution was allowed to warm to room temperature and stirred for one hour, during which the formation of a white precipitate was observed. This was then allowed to settle, filtered, and the solid dried *in vacuo* (ca. 10 minutes, room temperature) to yield [(^{EtDip}nacnac)Mg(OEt$_2$)I] as a white powder. Concentration of the supernatant solution (to ca. 5 mL) and storage at −40°C yielded further crops of [(^{EtDip}nacnac)Mg(OEt$_2$)I]. Colourless crystals of [(^{EtDip}nacnac)Mg(OEt$_2$)I] suitable for X-ray crystallographic analysis were grown from a concentrated benzene solution. Yield: 15.0 g (70%); ^{1}H NMR (499.9 MHz, C_6D_6, 298 K) δ 0.54 (br, 6H, OCH$_2$C*H*$_3$), 0.98 (br, 6H, NCCH$_2$C*H*$_3$), 1.14-1.47 (m, 24H, Ar-*o*-CH(C*H*$_3$)$_2$), 2.05 (br, 4H, NCC*H*$_2$CH$_3$), 3.35 (br, 8H, Ar-*o*-C*H*(CH$_3$)$_2$), 3.41-3.81 (vbr, 4H, OC*H*$_2$CH$_3$), 5.04 (s, 1H, NCC*H*CN), 7.10-7.16 (m, 6H, Ar-*H*). ^{13}C{^{1}H} NMR (125.7 MHz, C_6D_6, 298 K) δ 13.2 (OCH$_2$*C*H$_3$), 13.4 (NCCH$_2$*C*H$_3$), 24.9 (Ar-*o*-CH(*C*H$_3$)$_2$), 26.3 (br, Ar-*o*-CH(*C*H$_3$)$_2$) 28.2 (NC*C*H$_2$CH$_3$), 65.9 (O*C*H$_2$CH$_3$), 90.3 (NC*C*HCN), 124.3 (Ar-*C*), 125.6 (Ar-*C*), 143.3 (br, Ar-*C*), 144.6 (Ar-*C*), 175.0 (N*C*CH*C*N).

[{(^{EtDip}nacnac)Mg(μ-I)}$_2$]

The diethyl ether ligand can decoordinate from [(^{EtDip}nacnac)Mg(OEt$_2$)I] and form [{(^{EtDip}nacnac)Mg(μ-I)}$_2$], and a synthetic procedure and spectroscopic data are therefore included. To a colourless solution of [(^{EtDip}nacnac)Mg(OEt$_2$)I] (1.06 g, 1.58 mmol) in toluene (ca. 15 mL), all volatiles were removed *in vacuo* to give a white residue which was dried *in vacuo* (ca. 20 minutes, ca. 60°C). The white residue was washed with toluene (ca. 10 mL) to afford a white precipitate. This was then allowed to settle, filtered, and the solid dried *in vacuo* (ca. 10 minutes, ca. 60°C) to yield [{(^{EtDip}nacnac)Mg(μ-I)}$_2$] as a white powder. Concentration of the supernatant solution (to ca. 5 mL) and storage at −40°C yielded further crops of [{(^{EtDip}nacnac)Mg(μ-I)}$_2$]. Colourless crystals of [{(^{EtDip}nacnac)Mg(μ-I)}$_2$] suitable for X-ray crystallographic analysis were grown from a

concentrated benzene solution. Yield = 0.85 g (90%); ^{1}H NMR (499.9 MHz, C_6D_6, 298 K) δ 0.86 (t, J_{HH} = 7.4 Hz, 12H, NCCH_2C*H*$_3$), 1.05 (d, J_{HH} = 6.8 Hz, 24H, Ar-*o*-CH(C*H*$_3$)$_2$), 1.16 (d, J_{HH} = 6.8 Hz, 24H, Ar-*o*-CH(C*H*$_3$)$_2$), 1.92 (q, J_{HH} = 7.5 Hz, 8H, NCC*H*$_2$CH$_3$), 3.26 (sept, J_{HH} = 6.7 Hz, 8H, Ar-*o*-C*H*(CH$_3$)$_2$), 4.93 (s, 2H, NCC*H*CN), 7.06–7.21 (m, 12H, Ar-*H*). ^{13}C{^{1}H} NMR (125.7 MHz, C_6D_6, 298 K) δ 13.0 (NCCH$_2$*C*H$_3$), 25.0 (Ar-*o*-CH(*C*H$_3$)$_2$), 26.3 (Ar-*o*-CH(*C*H$_3$)$_2$), 28.3 (Ar-*o*-*C*H(CH$_3$)$_2$), 28.6 (NC*C*H$_2$CH$_3$), 89.6 (NC*C*HCN), 124.3 (Ar-*C*), 126.1 (Ar-*C*), 143.3 (Ar-*C*), 143.5 (Ar-*C*), 174.4 (N*C*CHC*N*).

[{(^{EtDip}nacnac)Mg}$_2$] 2b

A colourless solution of [(^{EtDip}nacnac)Mg(OEt$_2$)I] (1.01 g, 1.51 mmol, 1.00 equiv.) in toluene (ca. 20 mL) was stirred vigorously over a potassium mirror (590 mg, 15.1 mmol, 10.0 equiv.) for ca. 16 hours at room temperature. The resulting yellow solution was then allowed to settle, filtered, and the solution concentrated to ca. 10 mL, and stored at −40°C for 48 hours, affording [{(^{EtDip}nacnac)Mg}$_2$] **2b** as a pale-yellow crystalline solid. Concentration of the supernatant solution (to ca. 5 mL) and storage at −40°C yielded further crops of [{(^{EtDip}nacnac)Mg}$_2$] **2b**. Yellow crystals of [{(^{EtDip}nacnac)Mg}$_2$] **2b** suitable for X-ray crystallographic analysis were grown from a concentrated *n*-hexane solution. During the preparation and isolation of [{(^{EtDip}nacnac)Mg}$_2$] **2b**, a small amount (ca. 3%) of [{(^{EtDip}nacnac)Mg(μ-OH)}$_2$] **3b** (^{1}H NMR resonance of Mg(μ-OH)$_2$: δ −0.50 ppm) was formed in a later crop from exposure to moisture/air. Yield: 601 mg (42%); M.p.: >300°C. ^{1}H NMR (499.9 MHz, C_6D_6, 298 K) δ 0.90 (t, J_{HH} = 7.5 Hz, 12H, NCCH$_2$C*H*$_3$), 1.00 (d, J_{HH} = 6.9 Hz, 24H, Ar-*o*-CH(C*H*$_3$)$_2$), 1.21 (d, J_{HH} = 6.8 Hz, 24H, Ar-*o*-CH(C*H*$_3$)$_2$), 1.94 (q, J_{HH} = 7.5 Hz, 8H, NCC*H*$_2$CH$_3$), 3.14 (sept, J_{HH} = 6.9 Hz, 8H, Ar-*o*-C*H*(CH$_3$)$_2$), 4.91 (s, 2H, NCC*H*CN), 7.05-7.11 (m, 12H, Ar-*H*). ^{13}C{^{1}H} NMR (125.7 MHz, C_6D_6, 298 K) δ 13.4 (NCCH$_2$*C*H$_3$), 24.2 (Ar-*o*-CH(*C*H$_3$)$_2$), 25.7 (Ar-*o*-CH(*C*H$_3$)$_2$), 28.3 (Ar-*o*-*C*H(CH$_3$)$_2$), 29.0 (NC*C*H$_2$CH$_3$), 90.8 (NC*C*HCN), 123.9 (Ar-*C*), 125.2 (Ar-*C*), 142.6 (Ar-*C*), 145.0 (Ar-*C*), 172.6 (N*C*CHC*N*). Elemental analysis: calculated for $C_{62}H_{90}Mg_2N_4$ **2b**: C 79.22; H 9.65; N 5.96%; found C 78.64; H 9.51; N 5.66%. IR (ATR), ν~/cm^{-1}: 2959m, 1528m, 1460m, 1414s, 1312m, 1260m, 1165m, 1070m, 791m, 764m.

1.2.2 Synthesis of [{(^{EtDip}nacnac)Mg(μ-H)}$_2$] 7b and precursors

These complexes have been prepared in analogy to previously reported related derivatives.[3,4,7]

[(^{EtDip}nacnac)Mg*n*Bu] 9b

To a colourless solution of ^{EtDip}nacnacH (2.34 g, 5.23 mmol, 1.0 equiv.) at 0°C in toluene (20 mL), Mg(*n*Bu)$_2$ (1 M in heptane, 7.84 mL, 7.84 mmol, 1.5 equiv.) was added dropwise. The solution was allowed to warm to room temperature and stirred overnight (ca. 18 hours). The solution was then concentrated *in vacuo* (to ca. 10 mL) and placed at −40°C overnight. This initially afforded a colourless crystalline solid of [(^{EtDip}nacnac)Mg(μ-O*n*Bu)}$_2$] likely due to using a solution of Mg(*n*Bu)$_2$ that likely contained significant amounts of oxidised "(*n*Bu)Mg(O*n*Bu)". The remaining solution afforded a colourless crystalline solid of [(^{EtDip}nacnac)Mg*n*Bu] **9b**. Concentration of the supernatant solution (to ca. 5 mL) and storage at −40°C yielded further crops of [(^{EtDip}nacnac)Mg*n*Bu] **9b**. Colourless crystals of both [(^{EtDip}nacnac)Mg(μ-O*n*Bu)}$_2$] and [(^{EtDip}nacnac)Mg*n*Bu] **9b** suitable for X-ray crystallographic analysis were grown from a concentrated toluene solution. Yield ([(^{EtDip}nacnac)Mg*n*Bu] **9b**): 1.60 g (58%); ^{1}H NMR (500.1 MHz, C_6D_6, 295 K) δ −0.25 (t, J_{HH} = 7.9 Hz, 2H, Mg-C*H*$_2$CH$_2$CH$_2$CH$_3$), 0.82 (t, J_{HH} = 7.3 Hz, 3H, Mg-CH$_2$CH$_2$CH$_2$C*H*$_3$), 0.94–1.04 (m, 8H, Mg-CH$_2$CH$_2$C*H*$_2$CH$_3$ & NCCH$_2$C*H*$_3$), 1.17 (d, J_{HH} = 6.9 Hz, 12H, Ar-*o*-CH(C*H*$_3$)$_2$), 1.26-1.36 (m, 14H, Ar-*o*-CH(C*H*$_3$)$_2$ & Mg-CH$_2$C*H*$_2$CH$_2$CH$_3$), 2.10 (q, J_{HH} = 7.6 Hz, 4H, NCC*H*$_2$CH$_3$), 3.18 (sept, J_{HH} = 6.9 Hz, 4H, Ar-*o*-C*H*(CH$_3$)$_2$), 5.09 (s, 1H, NCC*H*CN), 7.11 (s, 6H, Ar-*H*). ^{13}C{^{1}H} NMR (125.8 MHz, C_6D_6, 295 K) δ 6.2 (Mg-*C*H$_2$CH$_2$CH$_2$CH$_3$), 13.1 (NCCH$_2$*C*H$_3$), 14.4 (Mg-CH$_2$CH$_2$CH$_2$*C*H$_3$), 23.4 (Ar-*o*-CH(*C*H$_3$)$_2$), 24.9 (Ar-*o*-CH(*C*H$_3$)$_2$), 28.5 (Ar-*o*-*C*H(CH$_3$)$_2$), 28.8 (NC*C*H$_2$CH$_3$), 31.0 (Mg-CH$_2$CH$_2$*C*H$_2$CH$_3$), 31.2 (Mg-CH$_2$*C*H$_2$CH$_2$CH$_3$), 90.3 (NC*C*HCN), 124.1 (Ar-*C*), 125.9 (Ar-*C*), 142.1 (Ar-*C*), 143.3 (Ar-*C*), 174.1 (N*C*CH*C*N). Elemental analysis: calculated for $C_{35}H_{54}MgN_2$ **9b**: C 79.75; H 10.33; 5.31%; found C 77.73; H 10.38; N 4.92%. IR (ATR), ν~/cm^{-1}: 2959m, 1526m, 1414s, 1375s, 1312s, 1261m, 1069m, 571m, 509m, 428m.

Yield of [(^{EtDip}nacnac)Mg(μ-O*n*Bu)}$_2$]: 500 mg (18%); ^{1}H NMR (500.1 MHz, C_6D_6, 298 K) δ 0.38 (d, J_{HH} = 6.8 Hz, 12H, Ar-*o*-CH(C*H*$_3$)$_2$), 0.92 (t, J_{HH} = 7.5 Hz, 12H, NCCH$_2$C*H*$_3$), 0.97 (t, J_{HH} = 7.4 Hz, Mg-OCH$_2$CH$_2$CH$_2$C*H*$_3$), 1.10 (d, J_{HH} = 6.6 Hz, 12H, Ar-*o*-CH(C*H*$_3$)$_2$), 1.20 (d, J_{HH} = 6.8 Hz, 12H, Ar-*o*-CH(C*H*$_3$)$_2$), 1.22-1.41 (m, 4H, Mg-OCH$_2$CH$_2$C*H*$_2$CH$_3$), 1.44 (d, J_{HH} = 6.9 Hz, 12H, Ar-*o*-CH(C*H*$_3$)$_2$), 1.52-1.62 (m, 4H, Mg-OCH$_2$C*H*$_2$CH$_2$CH$_3$), 1.68-1.79 (m, 4H, NCC*H*$_2$CH$_3$), 1.86-1.97 (m, 4H, NCC*H*$_2$CH$_3$), 3.01 (sept, J_{HH} = 6.8 Hz, 4H, Ar-*o*-C*H*(CH$_3$)$_2$), 3.46 (sept, J_{HH} = 6.7 Hz, 4H, Ar-*o*-C*H*(CH$_3$)$_2$) 3.60 (t, J_{HH} = 8.6 Hz, 4H, Mg-OC*H*$_2$CH$_2$CH$_2$CH$_3$), 4.95 (s, 2H, NCC*H*CN), 7.06-7.22 (m, 12H, Ar-*H*). ^{13}C{^{1}H} NMR (125.8 MHz, C_6D_6, 298 K) δ 13.3 (NCCH$_2$*C*H$_3$), 14.8 (Mg-OCH$_2$CH$_2$CH$_2$*C*H$_3$), 19.7 (Mg-OCH$_2$CH$_2$*C*H$_2$CH$_3$), 23.6 (Ar-*o*-CH(*C*H$_3$)$_2$), 24.2 (Ar-*o*-CH(*C*H$_3$)$_2$), 25.7 (Ar-*o*-CH(*C*H$_3$)$_2$), 27.9 (Ar-*o*-*C*H(CH$_3$)$_2$), 28.2 (NC*C*H$_2$CH$_3$), 29.2

(Ar-*o*-*C*H(CH_3)$_2$), 38.4 (Mg-OCH_2*C*$H_2CH_2CH_3$), 64.2 (Mg-O*C*$H_2CH_2CH_2CH_3$), 89.5 (NC*C*HCN), 123.7 (Ar-*C*), 124.0 (Ar-*C*), 125.5 (Ar-*C*), 142.2 (Ar-*C*), 144.3 (Ar-*C*), 146.9 (Ar-*C*), 173.5 (N*C*CH*C*N).

[(^{EtDip}nacnac)Mg(THF)*n*Bu]

To a colourless solution of [(^{EtDip}nacnac)Mg*n*Bu] **9b** (227.1 mg, 430.8 μmol, 1.0 equiv.) in *n*-hexane (10 mL), was added THF (42 μL, 0.52 mmol, 1.2 equiv.) at room temperature. The solution was stirred for 30 minutes before being concentrated *in vacuo* (to ca. 5 mL) and placed at −40°C overnight which afforded a colourless crystalline crop of [(^{EtDip}nacnac)Mg(THF)*n*Bu]. Concentration of the supernatant solution (to ca. 2 mL) and storage at −40°C yielded further crops of [(^{EtDip}nacnac)Mg(THF)*n*Bu]. Yield: 200.1 mg (78%); ^{1}H NMR (499.9 MHz, C_6D_6, 298 K) δ −0.36 (t, J_{HH} = 8.2 Hz, 2H, Mg-C$H_2$$CH_2CH_2CH_3$), 0.93 (t, J_{HH} = 7.3 Hz, 3H, Mg-$CH_2CH_2CH_2$CH_3), 1.00 (t, J_{HH} = 7.5 Hz, 6H, NCCH_2CH_3), 1.20-1.27 (m, 14H, Ar-*o*-CH(CH_3)$_2$ & Mg-CH_2CH_2C$H_2$$CH_3$), 1.27-1.34 (m, 16H, Ar-*o*-CH(CH_3)$_2$ & CH_2(CH_2)$_{THF}$), 1.41-1.51 (m, 2H, Mg-CH_2C$H_2$$CH_2CH_3$), 2.05 (q, J_{HH} = 7.5 Hz, 4H, NCC$H_2$$CH_3$), 3.29 (sept, J_{HH} = 7.0 Hz, 4H, Ar-*o*-C*H*(CH_3)$_2$), 3.69-3.73 (m, 4H, O(CH_2)$_{THF}$), 4.94 (s, 1H, NCC*H*CN), 7.17-7.19 (m, 6H, Ar-*H*). ^{13}C{^{1}H} NMR (125.7 MHz, C_6D_6, 298 K) δ 6.1 (Mg-*C*$H_2CH_2CH_2CH_3$), 13.7 (NCCH_2*C*H_3), 14.5 (Mg-$CH_2CH_2CH_2$*C*H_3), 24.6 (Ar-*o*-CH(*C*H_3)$_2$), 25.4 (CH_2(*C*H_2)$_{THF}$), 25.4 (Ar-*o*-CH(*C*H_3)$_2$), 28.2 (Ar-*o*-*C*H(CH_3)$_2$), 28.8 (NC*C*H_2CH_3), 32.0 (Mg-CH_2CH_2*C*H_2CH_3), 32.7 (Mg-CH_2*C*$H_2CH_2CH_3$), 69.9 (O(*C*H_2)$_{THF}$), 89.7 (NC*C*HCN), 123.9 (Ar-*C*), 125.1 (Ar-*C*), 142.7 (Ar-*C*), 145.5 (Ar-*C*), 173.1 (N*C*CH*C*N).

[{(^{EtDip}nacnac)Mg(μ-H)}$_2$] 7b

Method 1 (*in-situ*): To a colourless solution of [(^{EtDip}nacnac)Mg*n*Bu] **9b** (21.9 mg, 41.5 μmol, 1.0 equiv.) in C_6D_6 (0.6 mL) in a J Young NMR tube, was added phenylsilane (5.6 μL, 45 μmol, 1.1 equiv.) at room temperature. The reaction was monitored by ^{1}H NMR spectroscopy over time. After heating at 60°C for five hours, near full conversion (ca. 92%) of [(^{EtDip}nacnac)Mg*n*Bu] **9b** to [{(^{EtDip}nacnac)Mg(μ-H)}$_2$] **7b** was observed via ^{1}H NMR spectroscopy.

Method 2 (isolated): To a colourless solution of [(^{EtDip}nacnac)Mg*n*Bu] **9b** (506.0 mg, 959.9 μmol, 1.0 equiv.) in *n*-hexane (10 mL), was added a solution of phenylsilane (130 μL, 1.06 mmol, 1.1 equiv.) in *n*-hexane (2 mL) at room temperature. The solution was stirred and heated for five hours at 60°C, during which the formation of a white precipitate was observed. This was then allowed to settle, filtered, and the solid dried *in vacuo* (10 minutes, room temperature) to yield [{(^{EtDip}nacnac)Mg(μ-H)}$_2$] **7b** as a white powder. Concentration of the supernatant solution (ca. 5 mL) and storage at −40°C yielded further crops of [{(^{EtDip}nacnac)Mg(μ-H)}$_2$] **7b**. Colourless crystals suitable for X-ray crystallographic analysis were grown from a concentrated benzene

solution. Yield: 152.0 mg (34%); M.p.: 121-123°C (melting and decomposition from a white solid to a brown oil). ^{1}H NMR (500.1 MHz, C_6D_6, 298 K) δ 0.91 (t, J_{HH} = 7.5 Hz, 12H, NCCH$_2$CH_3), 0.98 (d, J_{HH} = 6.9 Hz, 24H, Ar-*o*-CH(CH_3)$_2$), 1.14 (d, J_{HH} = 6.9 Hz, 24H, Ar-*o*-CH(CH_3)$_2$), 1.88 (q, J_{HH} = 7.5 Hz, 8H, NCCH_2CH$_3$), 3.08 (sept, J_{HH} = 6.8 Hz, 8H, Ar-*o*-C*H*(CH$_3$)$_2$), 4.03 (s, 2H, Mg-*H*), 4.97 (s, 2H, NCC*H*CN), 7.01-7.12 (m, 12H, Ar-*H*). ^{13}C{^{1}H} NMR (125.8 MHz, C_6D_6, 298 K) δ 13.2 (NCCH$_2$*C*H$_3$), 23.8 (Ar-*o*-CH(*C*H$_3$)$_2$), 25.2 (Ar-*o*-CH(*C*H$_3$)$_2$), 28.4 (Ar-*o*-*C*H(CH$_3$)$_2$), 28.8 (NC*C*H$_2$CH$_3$), 89.4 (NC*C*HCN), 124.3 (Ar-*C*), 125.4 (Ar-*C*), 142.6 (Ar-*C*), 145.5 (Ar-*C*), 174.0 (N*C*CH*C*N).

[{(^{EtDip}nacnac)Mg(THF)(μ-H)}$_2$] 11b

To a colourless solution of isolated [{(^{EtDip}nacnac)Mg(μ-H)}$_2$] **7b** (15.3 mg, 16.2 μmol, 1.0 equiv.) in C_6D_6 (0.6 mL) in a J Young NMR tube was added THF (2.6 μL, 32 μmol, 2.0 equiv.) at room temperature. ^{1}H NMR spectroscopy was recorded as soon as possible. Immediate and complete conversion of [{(^{EtDip}nacnac)Mg(μ-H)}$_2$] **7b** to [{(^{EtDip}nacnac)Mg(THF)(μ-H)}$_2$] **11b** was observed by ^{1}H NMR spectroscopy. ^{1}H NMR (700.0 MHz, C_6D_6, 295 K) δ 1.04 (br, 12H, NCCH$_2$CH_3), 1.11-1.16 (m, 8H, CH$_2$(CH_2)$_{THF}$), 1.21 (br, 24H, Ar-*o*-CH(CH_3)$_2$), 1.37 (br, 24H, Ar-*o*-CH(CH_3)$_2$), 2.04 (br, 8H, NCCH_2CH$_3$), 3.24 (br, 8H, O(CH_2)$_{THF}$), 3.49 (br, 8H, Ar-*o*-C*H*(CH$_3$)$_2$), 4.10 (br, 2H, Mg-*H*), 5.00 (s, 2H, NCC*H*CN), 7.01-7.15 (m, 12H, Ar-*H*). ^{1}H NMR (499.9 MHz, C_6D_6, 344 K) δ 1.01 (t, J_{HH} = 7.5 Hz, 12H, NCCH$_2$CH_3), 1.18 (d, J_{HH} = 6.8 Hz, 48H, Ar-*o*-CH(CH_3)$_2$), 1.30-1.36 (m, 8H, CH$_2$(CH_2)$_{THF}$), 2.02 (q, J_{HH} = 7.4 Hz, 8H, NCCH_2CH$_3$), 3.29 (br, 8H, Ar-*o*-C*H*(CH$_3$)$_2$), 3.37-3.46 (m, 8H, O(CH_2)$_{THF}$), 4.08 (s, 2H, Mg-*H*), 4.98 (s, 2H, NCC*H*CN), 7.10 (s, 12H, Ar-*H*). ^{13}C{^{1}H} NMR (176.0 MHz, C_6D_6, 295 K) δ 13.5 (NCCH$_2$*C*H$_3$), 24.6 (Ar-*o*-CH(*C*H$_3$)$_2$), 25.1 (CH$_2$(*C*H$_2$)$_{THF}$), 25.4 (Ar-*o*-CH(*C*H$_3$)$_2$), 28.1 (Ar-*o*-*C*H(CH$_3$)$_2$), 28.8 (NC*C*H$_2$CH$_3$), 68.7 (O(*C*H$_2$)$_{THF}$), 89.2 (br, NC*C*HCN), 124.1 (Ar-*C*), 125.1 (Ar-*C*), 143.1 (Ar-*C*), 146.4 (br, Ar-*C*), 173.3 (N*C*CH*C*N).

[(^{EtDip}nacnac)Mg(DMAP)H] 12b

To a colourless solution of isolated [{(^{EtDip}nacnac)Mg(μ-H)}$_2$] **7b** (13.2 mg, 14.0 μmol, 1.0 equiv.) in C_6D_6 (0.6 mL) in a J Young NMR tube was added DMAP (3.6 mg, 29 μmol, 2.1 equiv.) at room temperature. ^{1}H NMR spectroscopy was recorded as soon as possible. Immediate and complete conversion of [{(^{EtDip}nacnac)Mg(μ-H)}$_2$] **7b** to [{(^{EtDip}nacnac)Mg(DMAP)H] **12b** at room temperature was observed via ^{1}H NMR spectroscopy. Upon heating at 60°C for ca. five minutes, some decomposition of [{(^{EtDip}nacnac)Mg(DMAP)H] **12b** was observed. ^{1}H NMR (499.9 MHz, C_6D_6, 298 K) δ 0.89 (vbr, 6H, Ar-*o*-CH(CH_3)$_2$), 1.16 (t, J_{HH} = 7.5 Hz, 6H, NCCH$_2$CH_3), 1.29 (br, 12H, Ar-*o*-CH(CH_3)$_2$), 1.59 (vbr, 6H, Ar-*o*-CH(CH_3)$_2$), 1.96 (s, 6H, N(CH_3)$_2$), 2.23 (q, J_{HH} = 7.5 Hz, 4H, NCCH_2CH$_3$), 3.35 (vbr, 4H, Ar-*o*-C*H*(CH$_3$)$_2$), 4.65 (s, 1H, Mg-*H*), 5.12 (s, 1H, NCC*H*CN), 5.73

(d, J_{HH} = 6.1 Hz, 2H, Ar_{DMAP}-*H*), 7.14-7.22 (m, 6H, Ar-*H*), 8.18 (d, J_{HH} = 6.1 Hz, 2H, Ar_{DMAP}-*H*). $^{13}C\{^1H\}$ NMR (125.7 MHz, C_6D_6, 298 K) δ 13.8 ($NCCH_2CH_3$), 24.5 (br, Ar-*o*-$CH(CH_3)_2$), 25.4 (br, Ar-*o*-$CH(CH_3)_2$), 28.4 (br, Ar-*o*-$CH(CH_3)_2$), 29.0 ($NCCH_2CH_3$), 38.1 ($N(CH_3)_2$), 89.1 (NC*C*HCN), 106.4 (Ar_{DMAP}-*C*), 124.0 (Ar-*C*), 124.9 (Ar-*C*), 143.2 (br, Ar-*C*), 145.6 (Ar-*C*), 149.2 (Ar_{DMAP}-*C*), 155.1 (Ar_{DMAP}-*C*), 173.1 (N*C*CH*C*N).

1.2.3 Synthesis of magnesium oxide complexes and hydrogen activation experiments

[{(^{MeDip}nacnac)Mg}$_2$(μ-O)] **1a**[2] and [{(^{iPrDip}nacnac)Mg}$_2$(μ-O)] **1c**[4] have been reported previously. For further synthetic work and transformations, once [{(^{RDip}nacnac)Mg}$_2$(μ-O)] **1** (R = Me **1a**, Et **1b**, *i*Pr **1c**) was synthesised *in-situ*, the solution was degassed via three freeze-pump-thaw cycles before being purged with pre-dried dinitrogen (ca. 1 bar) to remove any excess nitrous oxide from the solution. Where necessary for further transformations or synthetic work, once [{(^{RDip}nacnac)Mg}$_2$(μ-H)(μ-OH)] **4** (R = Me **4a**, Et **4b**, *i*Pr **4c**) was synthesised *in-situ*, the solution was degassed via three freeze-pump-thaw cycles before being purged with pre-dried dinitrogen (ca. 1 bar) to remove any excess dihydrogen from the solution. For R = *i*Pr, however, this was not possible due to the ease of dehydrogenation. The masses and number of moles of compounds generated *in-situ* have been given for their quantitative formation if this was indicated according to NMR spectroscopy.

[{(^{MeDip}nacnac)Mg}$_2$(μ-O)] 1a

A yellow solution of [{(^{MeDip}nacnac)Mg}$_2$] **2a** (18.0 mg, 20.4 μmol) in C_6D_6 (0.5 mL) in a J Young NMR tube was cooled to −70°C. The headspace was evacuated before refilling with pre-dried nitrous oxide (ca. 1 bar) and sealing the tube. The solution was allowed to warm to room temperature and the J Young NMR tube was shaken vigorously (ca. 10 seconds), during which an immediate colour change from yellow to colourless was observed. ^{1}H NMR spectroscopy was recorded as soon as possible. Immediate and complete conversion of [{(^{MeDip}nacnac)Mg}$_2$] **2a** to [{(^{MeDip}nacnac)Mg}$_2$(μ-O)] **1a** was observed via ^{1}H NMR spectroscopy at room temperature. ^{1}H NMR (499.9 MHz, C_6D_6, 298 K) δ 0.95 (d, J_{HH} = 6.9 Hz, 24H, Ar-*o*-CH(CH_3)$_2$), 1.17 (d, J_{HH} = 6.9 Hz, 24H, Ar-*o*-CH(CH_3)$_2$), 1.53 (s, 12H, NCCH_3), 2.99 (sept, J_{HH} = 6.9 Hz, 8H, Ar-*o*-C*H*(CH$_3$)$_2$), 4.83 (s, 2H, NCC*H*CN), 7.02-7.16 (m, 12H, Ar-*H*). ^{13}C{^{1}H} NMR (125.7 MHz, C_6D_6, 298 K) δ 22.8 (Ar-*o*-CH(*C*H$_3$)$_2$), 24.0 (NC*C*H$_3$), 25.0 (Ar-*o*-CH(*C*H$_3$)$_2$), 28.6 (Ar-*o*-*C*H(CH$_3$)$_2$), 94.6 (NC*C*HCN), 123.9 (Ar-*C*), 125.2 (Ar-*C*), 141.8 (Ar-*C*), 145.3 (Ar-*C*), 169.2 (N*C*CH*C*N).

[{(^{EtDip}nacnac)Mg}$_2$(μ-O)] 1b

Method 1 (*in-situ*): A yellow solution of [{(^{EtDip}nacnac)Mg}$_2$] **2b** (10.0 mg, 10.6 μmol) in C_6D_6 (0.5 mL) in a J Young NMR tube was cooled to −70°C. The headspace was evacuated before refilling with pre-dried nitrous oxide (ca. 1 bar) and sealing the tube. The solution was allowed to warm to room temperature and the J Young NMR tube was shaken vigorously (ca. 10 seconds), during which an immediate colour change from yellow to colourless was observed. ^{1}H NMR spectroscopy was

recorded as soon as possible. Immediate and complete conversion of [{(^{EtDip}nacnac)Mg}$_2$] **2b** to [{(^{EtDip}nacnac)Mg}$_2$(μ-O)] **1b** was observed via ^{1}H NMR spectroscopy at room temperature. During the preparation of *in-situ* [{(^{EtDip}nacnac)Mg}$_2$(μ-O)] **2b** a small amount (ca. 2%) of [{(^{EtDip}nacnac)Mg(μ-OH)}$_2$] **3b** (^{1}H NMR resonance of Mg(μ-OH)$_2$: δ −0.50 ppm) was formed.

Method 2 (isolated): A yellow solution of [{(^{EtDip}nacnac)Mg}$_2$] **2b** (163.4 mg, 173.8 μmol) in *n*-hexane (10 mL) in a J Young flask (250 mL) was cooled to −70°C. The headspace was evacuated before refilling with pre-dried nitrous oxide (ca. 1 bar) and sealing the flask. The solution was allowed to warm to room temperature and the flask was shaken vigorously (ca. 10 seconds), during which an immediate colour change from yellow to colourless was observed. All volatiles were subsequently removed *in vacuo* to give [{(^{EtDip}nacnac)Mg}$_2$(μ-O)] **1b** as a white powder. During the preparation and isolation of [{(^{EtDip}nacnac)Mg}$_2$(μ-O)] **1b** a small amount of [{(^{EtDip}nacnac)Mg(μ-OH)}$_2$] **3b** (^{1}H NMR resonance of Mg(μ-OH)$_2$: δ −0.50 ppm) was formed (ca. 7%). Yield: 134.0 mg (81%); M.p.: 282-284°C (decomposition and melting from a white solid to a yellowish oil). Note: compound **1b** is stable when heated in deuterated benzene followed by ^{1}H NMR spectroscopy and spectra are virtually unchanged after heating for 24 h, and after 4 days (with negligible decomposition that could not be reliably integrated against). ^{1}H NMR (499.9 MHz, C_6D_6, 298 K) δ 0.90 (t, J_{HH} = 7.5 Hz, 12H, NCCH$_2$C*H*$_3$), 0.97 (d, J_{HH} = 6.9 Hz, 24H, Ar-*o*-CH(C*H*$_3$)$_2$), 1.19 (d, J_{HH} = 6.9 Hz, 24H, Ar-*o*-CH(C*H*$_3$)$_2$), 1.94 (q, J_{HH} = 7.6 Hz, 8H, NCC*H*$_2$CH$_3$), 3.03 (sept, J_{HH} = 6.9 Hz, 8H, Ar-*o*-C*H*(CH$_3$)$_2$), 4.93 (s, 2H, NCC*H*CN), 7.00-7.15 (m, 12H, Ar-*H*). ^{13}C{^{1}H} NMR (125.7 MHz, C_6D_6, 298 K) δ 13.2 (NCCH$_2$*C*H$_3$), 22.6 (Ar-*o*-CH(*C*H$_3$)$_2$), 25.3 (Ar-*o*-CH(*C*H$_3$)$_2$), 28.6 (Ar-*o*-*C*H(CH$_3$)$_2$), 29.0 (NC*C*H$_2$CH$_3$), 90.0 (NC*C*HCN), 123.9 (Ar-*C*), 125.2 (Ar-*C*), 142.1 (Ar-*C*), 144.8 (Ar-*C*), 174.0 (N*C*CHC*N*). Elemental analysis: calculated for $C_{62}H_{90}Mg_2N_4O$ **1b**: C 77.89; H 9.49; 5.86%; found C 76.62; H 9.44; N 5.75%. IR (ATR), v~/cm^{-1}: 2959w, 1520m, 1418s, 1373w, 1308m, 1260w, 1069w, 694w, 528w, 434w.

[{^{MeDip}nacnac)Mg}$_2$(μ-H)(μ-OH)] 4a

A colourless *in-situ* prepared solution of [{(^{MeDip}nacnac)Mg}$_2$(μ-O)] **1a** (18.4 mg, 20.4 μmol) in C_6D_6 (0.5 mL) in a J Young NMR tube was cooled to −196°C. The headspace was evacuated before refilling with pre-dried dihydrogen (ca. 1 bar) and sealing the tube. The solution was allowed to warm to room temperature and the J Young tube was shaken vigorously (ca. 10 seconds). ^{1}H NMR spectroscopy was recorded as soon as possible. Immediate and complete conversion of [{(^{MeDip}nacnac)Mg}$_2$(μ-O)] **1a** to [{(^{MeDip}nacnac)Mg}$_2$(μ-H)(μ-OH)] **4a** was observed via ^{1}H NMR spectroscopy at room temperature. After approximately five minutes, a white precipitate was noted to form in the J Young NMR tube. This was assumed to be [{(^{MeDip}nacnac)Mg}$_2$(μ-H)(μ-OH)] **4a**. During the preparation of *in-situ* [{(^{MeDip}nacnac)Mg}$_2$(μ-H)(μ-OH)] **4a**, a small amount only (ca. 3%)

of [{(^{MeDip}nacnac)Mg(μ-OH)}$_2$] **3a** (^{1}H NMR resonance of Mg(μ-OH)$_2$: *δ* −0.43 ppm) was detected. Colourless crystals of **4a** suitable for X-ray crystallographic analysis were grown from the C_6D_6 solution. ^{1}H NMR (499.9 MHz, C_6D_6, 298 K) *δ* 0.04 (d, J_{HH} = 7.9 Hz, 1H, Mg-O*H*), 0.93 (d, J_{HH} = 6.9 Hz, 24H, Ar-*o*-CH(C*H*$_3$)$_2$), 1.11 (d, J_{HH} = 6.9 Hz, 24H, Ar-*o*-CH(C*H*$_3$)$_2$), 1.50 (s, 12H, NCC*H*$_3$), 3.07 (sept, J_{HH} = 6.8 Hz, 8H, Ar-*o*-C*H*(CH$_3$)$_2$), 3.17 (d, J_{HH} = 8.0 Hz, 1H, Mg-*H*), 4.83 (s, 2H, NCC*H*CN), 6.96-7.13 (m, 12H, Ar-*H*). ^{13}C{^{1}H} (125.7 MHz, C_6D_6, 298K) NMR *δ* 24.0 (Ar-*o*-CH(*C*H$_3$)$_2$), 24.5 (NC*C*H$_3$), 24.7 (Ar-*o*-CH(*C*H$_3$)$_2$), 28.4 (Ar-*o*-*C*H(CH$_3$)$_2$), 95.1 (NC*C*HCN), 124.1 (Ar-*C*), 125.4 (Ar-*C*), 142.2 (Ar-*C*), 146.5 (Ar-*C*), 169.0 (N*C*CHC*N*).

[{(^{MeDip}nacnac)Mg}$_2$(μ-OD)(μ-D)] 4a-*d*$_2$

A colourless *in-situ* prepared solution of [{(^{MeDip}nacnac)Mg}$_2$(μ-O)] **1a** (10.7 mg, 11.9 μmol) in C_6D_6 (0.5 mL) in a J Young NMR tube was cooled to −196°C. The headspace was evacuated before refilling with pre-dried dideuterium (ca. 1 bar) and sealing the tube. The solution was allowed to warm to room temperature and shaken vigorously (ca. 10 seconds). ^{1}H and ^{2}H NMR spectra were recorded as soon as possible. Immediate and complete conversion of [{(^{MeDip}nacnac)Mg}$_2$(μ-O)] **1a** to [{(^{MeDip}nacnac)Mg}$_2$(μ-OD)(μ-D)] **4a-*d*$_2$** was observed via ^{1}H and ^{2}H NMR spectroscopy at room temperature. ^{1}H NMR (700.0 MHz, C_6D_6, 295 K) *δ* 0.93 (d, J_{HH} = 7.0 Hz, 24H, Ar-*o*-CH(C*H*$_3$)$_2$), 1.11 (d, J_{HH} = 6.9 Hz, 24H, Ar-*o*-CH(C*H*$_3$)$_2$), 1.50 (s, 12H, NCC*H*$_3$), 3.07 (sept, J_{HH} = 7.0 Hz, 8H, Ar-*o*-C*H*(CH$_3$)$_2$), 4.83 (s, 2H, NCC*H*CN), 6.99-7.11 (m, 12H, Ar-*H*). ^{13}C{^{1}H} NMR (176.0 MHz, C_6D_6, 295 K) *δ* 24.0 (Ar-*o*-CH(*C*H$_3$)$_2$), 24.5 (NC*C*H$_3$), 24.7 (Ar-*o*-CH(*C*H$_3$)$_2$), 28.4 (Ar-*o*-*C*H(CH$_3$)$_2$), 95.1 (NC*C*HCN), 124.1 (Ar-*C*), 125.4 (Ar-*C*), 142.2 (Ar-*C*), 146.5 (Ar-*C*), 169.0 (N*C*CHC*N*). ^{2}H NMR (107.5 MHz, C_6D_6, 295 K) *δ* 0.03 (br, 1D, Mg-O*D*), 3.20 (s, 1D, Mg-*D*).

[{(^{EtDip}nacnac)Mg}$_2$(μ-H)(μ-OH)] 4b

Method 1 (*in-situ*): A colourless *in-situ* prepared solution of [{(^{EtDip}nacnac)Mg}$_2$(μ-O)] **1b** (10.0 mg, 10.5 μmol) in C_6D_6 (0.5 mL) in a J Young NMR tube was cooled to −196°C. The headspace was evacuated before refilling with pre-dried dihydrogen (ca. 1 bar) and sealing the tube. The solution was allowed to warm to room temperature and the J Young tube was shaken vigorously (ca. 10 seconds). ^{1}H NMR spectroscopy was recorded as soon as possible. Immediate and complete conversion of [{(^{EtDip}nacnac)Mg}$_2$(μ-O)] **1b** to [{(^{EtDip}nacnac)Mg}$_2$(μ-H)(μ-OH)] **4b** was observed via ^{1}H NMR spectroscopy at room temperature. During the preparation of *in-situ* [{(^{EtDip}nacnac)Mg}$_2$(μ-H)(μ-OH)] **4b**, a small amount of [{(^{EtDip}nacnac)Mg(μ-OH)}$_2$] **3b** (^{1}H NMR resonance of Mg(μ-OH)$_2$: *δ* −0.50 ppm) was formed (ca. 5%).

Method 2 (isolated): A colourless *in-situ* prepared solution of [{(^{EtDip}nacnac)Mg}$_2$(μ-O)] **1b** (174.7 mg, 182.7 μmol) in *n*-hexane (10 mL) in a J Young flask (250 mL) was cooled to −196°C.

The headspace was evacuated before refilling with pre-dried dihydrogen (ca. 1 bar) and sealing the flask. The solution was allowed to warm to room temperature and the flask was shaken vigorously (for ca. 10 seconds) during which the formation of a white precipitate was observed. The mixture was then stirred for 30 minutes at room temperature, before allowing the precipitate to settle. This was then filtered and the solid dried *in vacuo* (ca. 10 minutes, room temperature) to yield [{(^{EtDip}nacnac)Mg}$_2$(μ-H)(μ-OH)] **4b** as a white powder. Concentration of the supernatant solution (to ca. 5 mL) and storage at −40°C yielded further crops of [{(^{EtDip}nacnac)Mg}$_2$(μ-H)(μ-OH)] **4b**. During the preparation and isolation of [{(^{EtDip}nacnac)Mg}$_2$(μ-H)(μ-OH)] **4b**, a small amount of [{(^{EtDip}nacnac)Mg(μ-OH)}$_2$] **3b** (^{1}H NMR resonance of Mg(μ-OH)$_2$: δ −0.50 ppm) was formed (ca. 7%). Colourless crystals of **4b** suitable for X-ray crystallographic analysis were grown from a concentrated C_6D_6 solution. Yield: 100.0 mg (57%); M.p.: Decomposition between 138-140°C from a white solid to a brown oil. ^{1}H NMR (500.1 MHz, C_6D_6, 298 K) δ −0.13 (d, J_{HH} = 8.1 Hz, 1H, Mg-O*H*), 0.87–0.97 (m, 36H, NCCH$_2$C*H*$_3$ & Ar-*o*-CH(C*H*$_3$)$_2$), 1.14 (d, J_{HH} = 6.8 Hz, 24H, Ar-*o*-CH(C*H*$_3$)$_2$), 1.90 (q, J_{HH} = 7.5 Hz, 8H, NCC*H*$_2$CH$_3$), 3.10 (sept, J_{HH} = 6.9 Hz, 8H, Ar-*o*-C*H*(CH$_3$)$_2$), 3.21 (d, J_{HH} = 8.1 Hz, 1H, Mg-*H*), 4.95 (s, 2H, NCC*H*CN), 6.99-7.09 (m, 12H, Ar-*H*). ^{13}C{^{1}H} NMR (125.8 MHz, C_6D_6, 295 K) δ 13.3 (NCCH$_2$*C*H$_3$), 24.0 (Ar-*o*-CH(*C*H$_3$)$_2$), 24.9 (Ar-*o*-CH(*C*H$_3$)$_2$), 28.2 (Ar-*o*-*C*H(CH$_3$)$_2$), 28.7 (NC*C*H$_2$CH$_3$), 89.3 (NC*C*HCN), 124.0 (Ar-*C*), 125.3 (Ar-*C*), 142.5 (Ar-*C*), 145.8 (Ar-*C*), 173.5 (N*C*CH*C*N). Elemental analysis: calculated for $C_{62}H_{92}Mg_2N_4O$ **4b**: C 77.73; H 9.68; N 5.85%; found C 77.56; H 9.17; N 5.41%. IR (ATR), $\tilde{v}$/cm^{-1}: 2961m, 1522m, 1458m, 1418s, 1371m, 1310m, 1260m, 1069m, 691m, 525w.

Low temperature hydrogenation attempt

A colourless *in-situ* prepared solution of [{(^{EtDip}nacnac)Mg}$_2$(μ-O)] **1b** (10.0 mg, 10.5 μmol) in *n*-hexane (0.5 mL) was cooled to −30°C in a J Young NMR tube. The headspace was evacuated before refilling with pre-dried dihydrogen (ca. 1 bar) and sealing the tube. The solution was held at −30°C for 30 minutes before freeze-pump-thawing three times (freezing at −196°C and thawing at −30°C) under vacuum. All volatiles were subsequently removed *in vacuo* at −30°C to afford a white residue. The white residue was redissolved in C_6D_6 (0.6 mL) and ^{1}H NMR spectroscopy was recorded. Only a trace amount of [{(^{EtDip}nacnac)Mg}$_2$(μ-H)(μ-OH)] **4b** (ca. <4%) was observed to have formed, with the majority being unreacted [{(^{EtDip}nacnac)Mg}$_2$(μ-O)] **1b** (ca. 59%), alongside [{(^{EtDip}nacnac)Mg(μ-OH)}$_2$] **3b** (ca. 29%) and ^{EtDip}nacnacH (ca. 8%) from decomposition. Although significant decomposition had occurred, likely due to the prolonged handling at low temperatures and numerous vacuum and filling cycles, the absence of significant amounts of **4b** suggests that no significant conversion occurred at −30°C.

[{(^{EtDip}nacnac)Mg}$_2$(μ-OD)(μ-D)] 4b-*d*$_2$

A colourless *in-situ* prepared solution of [{(^{EtDip}nacnac)Mg}$_2$(μ-O)] **1b** (10.2 mg, 10.7 μmol) in C_6D_6 (0.5 mL) in a J Young NMR tube was cooled to −196°C. The headspace was evacuated before refilling with pre-dried dideuterium (ca. 1 bar) and sealing the tube. The solution was allowed to warm to room temperature and shaken vigorously (ca. 10 seconds). ^{1}H and ^{2}H NMR spectra were recorded as soon as possible. Immediate and complete conversion of [{(^{EtDip}nacnac)Mg}$_2$(μ-O)] **1b** to [{(^{EtDip}nacnac)Mg}$_2$(μ-OD)(μ-D)] **4b-*d*$_2$** was observed via ^{1}H and ^{2}H NMR spectroscopy at room temperature. ^{1}H NMR (700.0 MHz, C_6D_6, 295 K) δ 0.92 (t, J_{HH} = 7.5 Hz, 12H, NCCH$_2$C*H*$_3$), 0.95 (d, J_{HH} = 6.9 Hz, 24H, Ar-*o*-CH(C*H*$_3$)$_2$), 1.14 (d, J_{HH} = 6.9, 24H, Ar-*o*-CH(C*H*$_3$)$_2$), 1.90 (q, J_{HH} = 7.5 Hz, 8H, NCC*H*$_2$CH$_3$), 3.10 (sept, J_{HH} = 6.9 Hz, 8H, Ar-*o*-C*H*(CH$_3$)$_2$), 4.95 (s, 2H, NCC*H*CN), 7.03–7.11 (m, 12H, Ar-*H*). ^{2}H NMR (107.5 MHz, C_6D_6, 295 K) δ −0.2 (vbr, 1D, Mg-O*D*), 3.24 (s, 1D, Mg-*D*). ^{13}C{^{1}H} NMR (176.0 MHz, C_6D_6, 295 K) δ 13.3 (NCCH$_2$*C*H$_3$), 24.0 (Ar-*o*-CH(*C*H$_3$)$_2$), 24.9 (Ar-*o*-CH(*C*H$_3$)$_2$), 28.2 (NC*C*H$_2$CH$_3$), 28.7 (Ar-*o*-*C*H(CH$_3$)$_2$), 89.3 (NC*C*HCN), 124.0 (Ar-*C*), 125.3 (Ar-*C*), 142.5 (Ar-*C*), 145.8 (Ar-*C*), 173.5 (N*C*CH*C*N).

[{(^{iPrDip}nacnac)Mg}$_2$(μ-H)(μ-OH)] 4c

A colourless *in-situ* prepared solution of [{(^{iPrDip}nacnac)Mg}$_2$(μ-O)] **1c** (10.0 mg, 9.88 μmol) in C_6D_6 (0.5 mL) in a J Young NMR tube was cooled to −196°C. The headspace was evacuated before refilling with pre-dried dihydrogen (ca. 1 bar) and sealing the tube. The solution was allowed to warm to room temperature and shaken vigorously (ca. 10 seconds). ^{1}H NMR spectroscopy was recorded as soon as possible. Immediate and complete conversion of [{(^{iPrDip}nacnac)Mg}$_2$(μ-O)] **1c** to [{(^{iPrDip}nacnac)Mg}$_2$(μ-H)(μ-OH)] **4c** was observed via ^{1}H NMR spectroscopy at room temperature. During the preparation of *in-situ* [{(^{iPrDip}nacnac)Mg}$_2$(μ-H)(μ-OH)] **4c**, a small amount of [{(^{iPrDip}nacnac)Mg(μ-OH)}$_2$] **3c** (^{1}H NMR resonance of Mg(μ-OH)$_2$: δ −0.53 ppm) was formed (ca. 3%). Colourless crystals suitable for X-ray crystallographic analysis were grown from a concentrated benzene solution under a hydrogen/nitrogen atmosphere. Note that this complex was difficult to isolate due to the ease of dehydrogenation. ^{1}H NMR (499.9 MHz, C_6D_6, 283 K) δ −0.30 (d, J_{HH} = 8.3 Hz, 1H, Mg-O*H*), 0.92-0.99 (m, 48H, NCCH(C*H*$_3$)$_2$ & Ar-*o*-CH(C*H*$_3$)$_2$), 1.23 (d, J_{HH} = 6.8 Hz, 24H, Ar-*o*-CH(C*H*$_3$)$_2$), 2.44 (sept, J_{HH} = 6.4 Hz, 4H, NCC*H*(CH$_3$)$_2$), 3.14 (sept, J_{HH} = 7.0 Hz, 8H, Ar-*o*-C*H*(CH$_3$)$_2$), 3.27 (d, J_{HH} = 8.3 Hz, 1H, Mg-*H*), 4.86 (s, 2H, NCC*H*CN), 7.03-7.10 (m, 12H, Ar-*H*). ^{13}C{^{1}H} NMR (125.7 MHz, C_6D_6, 323 K) δ 23.5 (Ar-*o*-CH(*C*H$_3$)$_2$), 24.3 (Ar-*o*-CH(*C*H$_3$)$_2$), 26.0 (NCCH(*C*H$_3$)$_2$) 27.8 (Ar-*o*-*C*H(CH$_3$)$_2$), 31.8 (NC*C*H(CH$_3$)$_2$), 85.4 (NC*C*HCN), 124.1 (Ar-*C*), 125.1 (Ar-*C*), 143.2 (Ar-*C*), 145.2 (Ar-*C*), 178.9 (N*C*CH*C*N).

Competitive hydrogenation attempt

A colourless *in-situ* prepared solution of [{(^{EtDip}nacnac)Mg}$_2$(μ-O)] **1b** (7.9 mg, 8.4 μmol, 1.0 equiv.) and [{(^{iPrDip}nacnac)Mg}$_2$(μ-O)] **1c** (9.0 mg, 9.0 μmol, 1.1 equiv.) in C_6D_6 (0.6 mL) in a J Young NMR tube was cooled to −196°C. The headspace was evacuated before refilling with pre-dried dihydrogen (ca. 1 bar) and sealing the tube. The solution was allowed to warm to room temperature and shaken vigorously (ca. 10 seconds). ^{1}H NMR spectroscopy was recorded as soon as possible (Figure S104). Immediate and near complete conversion of [{(^{EtDip}nacnac)Mg}$_2$(μ-O)] **1b** and [{(^{iPrDip}nacnac)Mg}$_2$(μ-O)] **1c** to [{(^{EtDip}nacnac)Mg}$_2$(μ-H)(μ-OH)] **4b** and [{(^{iPrDip}nacnac)Mg}$_2$(μ-H)(μ-OH)] **4c**, respectively, was observed via ^{1}H NMR spectroscopy. After the addition of dihydrogen (ca. 1 bar), a white precipitate was noted at the bottom of the J Young NMR tube. This was assumed to be [{(^{iPrDip}nacnac)Mg}$_2$(μ-O)] **1c**, which was found to be less soluble in C_6D_6, relative to [{(^{EtDip}nacnac)Mg}$_2$(μ-O)] **1b**. Thus, no significant difference in the rate of activation of dihydrogen between [{(^{EtDip}nacnac)Mg}$_2$(μ-O)] **1b** and [{(^{iPrDip}nacnac)Mg}$_2$(μ-O)] **1c** could be practically determined from this experiment.

[{(^{EtDip}nacnac)Mg}$_2$(μ-H)(μ-OH)] 4b and [{(^{iPrDip}nacnac)Mg}$_2$(μ-O)] 1c exchange attempt

To a colourless solution of isolated [{(^{EtDip}nacnac)Mg}$_2$(μ-H)(μ-OH)] **4b** (11.2 mg, 11.7 μmol, 1.0 equiv.) in C_6D_6 (0.4 mL) in a J Young NMR tube, was added a colourless *in-situ*-prepared (and degassed) solution of [{(^{iPrDip}nacnac)Mg}$_2$(μ-O)] **1c** (14.1 mg, 13.9 μmol, 1.2 equiv.) in C_6D_6 (0.4 mL) at room temperature. The reaction was monitored by ^{1}H NMR spectroscopy (Figure S105) over time and was additionally heated at 100°C for 45 hours. The mixture largely stayed as **4b** and **1c** which is in line with **4c** dehydrogenating more easily. The formation of only a small amount (ca. 9%) of [{(^{iPrDip}nacnac)Mg}$_2$(μ-H)(μ-OH)] **4c** (^{1}H NMR resonance of Mg-O*H*: δ −0.30 ppm) was observed via ^{1}H NMR spectroscopy. Please note that the related experiment of treating [{(^{iPrDip}nacnac)Mg}$_2$(μ-H)(μ-OH)] **4c** with [{(^{EtDip}nacnac)Mg}$_2$(μ-O)] **1b** could not be performed due to the instability of **4c** in the absence of dihydrogen.

[{(^{EtDip}nacnac)Mg(THF)}$_2$(μ-O)] 5b

Method 1 (*in-situ*): To a colourless *in-situ* prepared solution of [{(^{EtDip}nacnac)Mg}$_2$(μ-O)] **1b** (19.5 mg, 20.4 μmol, 1.0 equiv.) in C_6D_6 (0.5 mL) in a J Young NMR tube was added THF (3.4 μL, 10.2 μmol, 0.5 equiv., from a 3 M THF stock solution in C_6D_6) at room temperature. ^{1}H NMR spectroscopy was recorded as soon as possible. Further 0.5 equivalents of THF were added and the reaction was monitored by ^{1}H NMR spectroscopy over time. Upon additions of THF to [{(^{EtDip}nacnac)Mg}$_2$(μ-O)] **1b**, the gradual formation of [{(^{EtDip}nacnac)Mg(THF)}$_2$(μ-O)] **5b** was observed. After 1 equivalent of THF was added to [{(^{EtDip}nacnac)Mg}$_2$(μ-O)] **1b**, colourless crystals

appeared to form in the J Young NMR tube. This was assumed to be [{(^{EtDip}nacnac)Mg(THF)}$_2$(μ-O)] **5b**. During the sub-stoichiometric additions of THF to [{(^{EtDip}nacnac)Mg}$_2$(μ-O)] **1b** only one set of resonances for the β-diketiminate ligands was observed, with a gradual shift of the ligand backbone resonance.

Method 2 (isolated): To a colourless *in-situ* prepared solution of [{(^{EtDip}nacnac)Mg}$_2$(μ-O)] **1b** (103.0 mg, 107.7 μmol, 1.0 equiv.) in *n*-hexane (10 mL), was added THF (100 μL, 1.23 mmol, 11.4 equiv.) at room temperature. The solution was stirred for 10 minutes, concentrated *in vacuo* (to ca. 5 mL), and placed at −40°C overnight which afforded a colourless crystalline crop of [{(^{EtDip}nacnac)Mg(THF)}$_2$(μ-O)] **5b**. Concentration of the supernatant solution and storage at −40°C yielded further crops of [{(^{EtDip}nacnac)Mg(THF)}$_2$(μ-O)] **5b**. During the preparation and isolation of [{(^{EtDip}nacnac)Mg(THF)}$_2$(μ-O)] **5b**, a small amount (ca. 5%) of [{(^{EtDip}nacnac)Mg(μ-OH)}$_2$] **3b** (^{1}H NMR resonance of Mg(μ-OH)$_2$: δ −0.50 ppm) was formed. Colourless crystals suitable for X-ray crystallographic analysis were grown from a concentrated benzene solution. Yield: 70.3 mg (59%); ^{1}H NMR (500.1 MHz, C_6D_6, 298 K) δ 0.98 (t, J_{HH} = 7.5 Hz, 12H, NCCH$_2$CH_3), 1.12 (br, 24H, Ar-*o*-CH(CH_3)$_2$), 1.23 (d, J_{HH} = 6.8 Hz, 24H, Ar-*o*-CH(CH_3)$_2$), 1.28 (br, 8H, CH$_2$(CH_2)$_{THF}$), 1.98 (q, J_{HH} = 7.5 Hz, 8H, NCCH_2CH$_3$), 3.20 (br, 8H, Ar-*o*-C*H*(CH$_3$)$_2$), 3.46 (br, 8H, O(CH_2)$_{THF}$), 4.88 (s, 2H, NCC*H*CN), 7.07-7.14 (m, 12H, Ar-*H*). ^{13}C{^{1}H} NMR (125.8 MHz, C_6D_6, 298 K) δ 13.6 (NCCH$_2$*C*H$_3$), 24.2 (Ar-*o*-CH(*C*H$_3$)$_2$), 25.4 (CH$_2$(*C*H$_2$)$_{THF}$), 25.5 (Ar-*o*-CH(*C*H$_3$)$_2$), 28.1 (Ar-*o*-*C*H(CH$_3$)$_2$), 29.0 (NC*C*H$_2$CH$_3$), 69.3 (O(*C*H$_2$)$_{THF}$), 89.6 (NC*C*HCN), 123.7 (Ar-*C*), 124.6 (Ar-*C*), 142.6 (Ar-*C*), 146.4 (Ar-*C*), 173.0 (N*C*CH*C*N). Elemental analysis: calculated for $C_{70}H_{106}Mg_2N_4O_3$ **5b**: C 76.42; H 9.71; N 5.09%; found C 75.87; H 9.51; N 4.90%. IR (ATR), v~/cm^{-1}: 2959w, 1528m, 1422s, 1310m, 1260m, 1070w, 793w, 756w, 482w, 447m, 420m.

Stability study of **5b**: A colourless *in-situ* prepared solution of [{(^{EtDip}nacnac)Mg(THF)}$_2$(μ-O)] **5b** (22.4 mg, 20.4 μmol) in C_6D_6 (0.5 mL) in a J Young NMR tube was heated at 80°C for 20 hours and then 100°C for 198 hours respectively. The reaction was monitored by ^{1}H NMR spectroscopy over time. Upon heating, complex [{(^{EtDip}nacnac)Mg(THF)}$_2$(μ-O)] **5b** slowly decomposed, for example, after 24 hours, ca. 12% of **5b** was consumed and after 198 hours, ca. 42% of **5b** was consumed.

[{(^{EtDip}nacnac)Mg(DMAP)}$_2$(μ-O)] 6b

To a colourless solution of [{(^{EtDip}nacnac)Mg}$_2$(μ-O)] **1b** (111.0 mg, 118.1 μmol, 1.0 equiv.) in benzene (10 mL), a solution of DMAP (30.3 mg, 248.0 μmol, 2.1 equiv.) in benzene (5 mL) was added at room temperature. The solution was stirred for 10 minutes, concentrated *in vacuo* (to ca. 10 mL), and placed at 6°C overnight which afforded a colourless crystalline crop of [{(^{EtDip}nacnac)Mg(DMAP)}$_2$(μ-O)] **6b** as the major product (ca. 52%) in a compound mixture that had co-crystallised with [{(^{EtDip}nacnac)Mg(DMAP)(μ-OH)}$_2$] **8b** (16%) and other unidentifiable

nacnac-containing compounds. [{(^{EtDip}nacnac)Mg(DMAP)}$_2$(μ-O)] **6b** could not be purified from the compound mixture. At room temperature, the ^{1}H NMR spectrum shows many broad resonances. High-temperature NMR spectroscopy was thwarted as the mixture started to decompose at elevated temperatures in solution. ^{1}H NMR (499.9 MHz, C_6D_6, 298 K) δ 1.09 (t, J_{HH} = 7.5 Hz, 12H, NCCH$_2$C*H*$_3$), 1.21 (vbr, 48H, Ar-*o*-CH(CH$_3$)$_2$), 2.04 (q, J_{HH} = 7.5 Hz, 12H), 2.28 (s, 16H, N(C*H*$_3$)$_3$), 2.96 (br, 4H, Ar-*o*-C*H*(CH$_3$)$_2$), 3.52 (br, 4H, Ar-*o*-C*H*(CH$_3$)$_2$), 5.04 (s, 2H, NCC*H*CN), 6.04 (br, 4H, Ar$_{DMAP}$-*H*), 7.11-7.26 (m, 12H, Ar-*H*), 8.46 (vbr, 4H, Ar$_{DMAP}$-*H*). ^{13}C{^{1}H} NMR (125.7 MHz, C_6D_6, 298 K) δ 13.9 (NCCH$_2$*C*H$_3$), 23.8 (vbr, Ar-*o*-CH(*C*H$_3$)$_2$), 24.2 (vbr, Ar-*o*-CH(*C*H$_3$)$_2$), 27.3 (vbr, Ar-*o*-*C*H(CH$_3$)$_2$), 28.0 (vbr, Ar-*o*-*C*H(CH$_3$)$_2$), 28.9 (NC*C*H$_2$CH$_3$), 38.4 (N(*C*H$_3$)$_2$), 89.2 (NC*C*HCN), 106.2 (vbr, Ar$_{DMAP}$-*C*), 123.9 (Ar-*C*), 124.1 (Ar-*C*), 142.4 (Ar-*C*), 148.2 (Ar$_{DMAP}$-*C*), 148.5 (Ar-*C*), 154.6 (vbr, Ar$_{DMAP}$-*C*), 172.4 (N*C*CH*C*N).

Reaction of [{(^{EtDip}nacnac)Mg(THF)}$_2$(μ-O)] 5b with DMAP

To a colourless solution of [{(^{EtDip}nacnac)Mg(THF)}$_2$(μ-O)] **5b** (13.5 mg, 12.3 μmol, 1.0 equiv.) in C_6D_6 (0.5 mL) in a J Young NMR tube was added DMAP (18.0 μL, 3.0 μmol, 0.25 equiv., from a 0.165 M DMAP stock solution in C_6D_6) at room temperature. The reaction was monitored by ^{1}H NMR spectroscopy over time. Further 0.25 equivalents of DMAP were added until all starting material was consumed. Upon additions of DMAP to [{(^{EtDip}nacnac)Mg(THF)}$_2$(μ-O)] **5b** the formation of [{(^{EtDip}nacnac)Mg(DMAP)}$_2$(μ-O)] **6b** (ca. 72%) and resonances for uncoordinated THF were observed along with the formation of a small amount (ca. 10%) of [{(^{EtDip}nacnac)Mg(DMAP)(μ-OH)}$_2$] **8b** and other nacac-containing reaction products.

Hydrogenation attempts of donor adducts 5b, 5c and 6b

5b: A colourless solution of isolated crystalline [{(^{EtDip}nacnac)Mg(THF)}$_2$(μ-O)] **5b** (15.0 mg, 13.6 μmol) in C_6D_6 (0.6 mL) in a J Young NMR tube was cooled to −196°C. The headspace was evacuated before refilling with pre-dried dihydrogen (ca. 1 bar) and sealing the tube. The solution was allowed to warm to room temperature and the J Young tube was shaken vigorously (ca. 10 seconds). ^{1}H NMR spectroscopy was recorded as soon as possible (Figure S120). Immediate and complete conversion of [{(^{EtDip}nacnac)Mg(THF)}$_2$(μ-O)] **5b** to [{(^{EtDip}nacnac)Mg}$_2$(μ-H)(μ-OH)] **4b** and resonances for uncoordinated THF were observed via ^{1}H NMR spectroscopy at room temperature. To this colourless *in-situ*-prepared solution of [{(^{EtDip}nacnac)Mg}$_2$(μ-H)(μ-OH)] **4b** (13.0 mg, 13.6 μmol, 1.0 equiv.) in C_6D_6 (0.6 mL) in the J Young NMR tube, was added an excess of THF (17.8 μL, 218 μmol, 16.0 equiv.). ^{1}H NMR spectroscopy was recorded as soon as possible. Immediate and complete conversion of [{(^{EtDip}nacnac)Mg}$_2$(μ-H)(μ-OH)] **4b** to [{(^{EtDip}nacnac)Mg(THF)}$_2$(μ-O)] **5b** was observed via ^{1}H

NMR spectroscopy at room temperature. During the experiment, a small amount (ca. 5%) of [{(^{EtDip}nacnac)Mg(μ-OH)}$_2$] **3b** (^{1}H NMR resonance of Mg(μ-OH)$_2$: δ −0.50 ppm) was observed.

5c: A colourless solution of isolated, crystallised [{(^{iPrDip}nacnac)Mg(THF)}$_2$(μ-O)] **5c** (11.0 mg, 9.51 μmol) in C_6D_6 (0.5 mL) in a J Young NMR tube was cooled to −196°C. The headspace was evacuated before refilling with pre-dried dihydrogen (ca. 1 bar) and sealing the tube. The solution was allowed to warm to room temperature and the J Young NMR tube was shaken vigorously (for ca. 10 seconds). ^{1}H NMR spectroscopy was recorded as soon as possible (Figure S121). Immediate and complete conversion of [{(^{iPrDip}nacnac)Mg(THF)}$_2$(μ-O)] **5c** to [{(^{iPrDip}nacnac)Mg}$_2$(μ-H)(μ-OH)] **4c** and resonances of uncoordinated THF were observed via ^{1}H NMR spectroscopy at room temperature. During the experiment, a small amount (ca. 5%) of [{(^{iPrDip}nacnac)Mg(μ-OH)}$_2$] **3c** (^{1}H NMR resonance of Mg(μ-OH)$_2$: δ −0.55 ppm) was observed.

6b: A colourless solution of impure* [{(^{EtDip}nacnac)Mg(DMAP)}$_2$(μ-O)] **6b** (15.0 mg, 12.5 μmol) in C_6D_6 (0.6 mL) in a J Young NMR tube was cooled to −196°C. The headspace was evacuated before refilling with pre-dried dihydrogen (ca. 1 bar) and sealing the tube. The solution was allowed to warm to room temperature and shaken vigorously (ca. 10 seconds). The reaction was monitored by ^{1}H NMR spectroscopy over time (Figure S122) and heated at 100°C for 18 hours, in which predominantly decomposition was observed. No quantity of [{(^{EtDip}nacnac)Mg}$_2$(μ-H)(μ-OH)] **4b** was detected at any stage. [*Due to the difficulty of isolating pure crystalline [{(^{EtDip}nacnac)Mg(DMAP)}$_2$(μ-O)] **6b**, this sample contained a mixture of **6b** (52%), a small amount of [{(^{EtDip}nacnac)Mg(DMAP)(μ-OH)}$_2$] **8b** (16%), as well as small amounts of other unidentifiable nacnac-containing compounds (31%). The sample was nevertheless used to qualitatively test the reactivity with hydrogen].

1.2.4 Dehydrogenation experiments

Dehydrogenation attempts for compounds 4 using vacuum

4a: A colourless *in-situ* prepared solution of [{(^{MeDip}nacnac)Mg}$_2$(μ-H)(μ-OH)] **4a** (12.0 mg, 13.3 μmol) in C_6D_6 (0.5 mL) in a J Young NMR tube was freeze-pump-thaw-degassed three times (one cycle of degassing) under vacuum before refilling the tube with pre-dried dinitrogen (ca. 1 bar). The reaction was monitored by ^{1}H NMR spectroscopy (Figure S123) before the tube was freeze-pump-thaw-degassed three times more. [{(^{MeDip}nacnac)Mg}$_2$(μ-H)(μ-OH)] **4a** was shown to be stable towards degassing under these conditions. No evidence of the formation of [{(^{MeDip}nacnac)Mg}$_2$(μ-O)] **1a** was observed via ^{1}H NMR spectroscopy, as indicated by the absence of the resonance backbone-C*H* at 4.83 ppm. During the experiment, a small amount (ca. 9%) of [{(^{MeDip}nacnac)Mg(μ-OH)}$_2$] **3a** (^{1}H NMR resonance of Mg(μ-OH)$_2$: δ −0.43 ppm) was observed to form.

4b: A colourless *in-situ* prepared solution of [{(^{EtDip}nacnac)Mg}$_2$(μ-H)(μ-OH)] **4b** (10.0 mg, 10.4 μmol) in C_6D_6 (0.5 mL) in a J Young NMR tube was freeze-pump-thaw-degassed three times (one cycle of degassing) under vacuum before being refilled the tube with pre-dried dinitrogen (ca. 1 bar). The reaction was monitored by ^{1}H NMR spectroscopy (Figure S124) before the tube was freeze-pump-thawed three times more. [{(^{EtDip}nacnac)Mg}$_2$(μ-H)(μ-OH)] **4b** was found to be moderately stable towards degassing as only a small amount (ca. 10%) of [{(^{EtDip}nacnac)Mg}$_2$(μ-O)] **1b** (^{1}H NMR resonance of backbone-C*H*: δ 4.93 ppm) was observed to form via ^{1}H NMR spectroscopy.

To a colourless *in-situ*-prepared solution of [{(^{EtDip}nacnac)Mg}$_2$(μ-H)(μ-OH)] **4b** (18.0 mg, 18.8 μmol) in C_6D_6 (0.6 mL) in a J Young NMR tube, all volatiles were removed *in vacuo* to give a white residue. The white residue was heated for eight hours at 80°C *in vacuo* (1.5-3.0 × 10^{-2} mbar) and then redissolved in C_6D_6 (0.6 mL). The regeneration of [{(^{EtDip}nacnac)Mg}$_2$(μ-O)] **1b** (ca. 23%, ^{1}H NMR resonance of backbone-C*H*: δ 4.93 ppm) was observed via ^{1}H NMR spectroscopy (Figure S125). Unreacted [{(^{EtDip}nacnac)Mg}$_2$(μ-H)(μ-OH)] **4b** (ca. 30%, ^{1}H NMR resonance of backbone-C*H*: δ 4.95 ppm) was also observed. Additionally, the formation of [{(^{EtDip}nacnac)Mg(μ-OH)}$_2$] **3b** (ca. 26%, ^{1}H NMR resonance of Mg(μ-OH)$_2$: δ −0.50 ppm) and ^{EtDip}nacnacH (ca. 21%, ^{1}H NMR resonance of backbone-C*H*: δ 5.07 ppm) were observed by ^{1}H NMR spectroscopy, indicative of decomposition. The J Young NMR tube was then cooled to −70°C. The headspace was evacuated before refilling with pre-dried dihydrogen (ca. 1 bar) and sealing the tube. The solution was allowed to warm to room temperature and shaken vigorously (ca. 10 seconds). ^{1}H

NMR spectroscopy was recorded as soon as possible. The earlier presence of [{(^{EtDip}nacnac)Mg}$_2$(μ-O)] **1b** was confirmed via ^{1}H NMR spectroscopy as the resonance of the backbone-C*H* of [{(^{EtDip}nacnac)Mg}$_2$(μ-O)] **1b** was consumed to re-form [{(^{EtDip}nacnac)Mg}$_2$(μ-H)(μ-OH)] **4b**.

4c: A colourless *in-situ* prepared solution of [{(^{iPrDip}nacnac)Mg}$_2$(μ-H)(μ-OH)] **4c** (10.0 mg, 9.86 μmol) in C_6D_6 (0.5 mL) in a J Young NMR tube was freeze-pump-thaw-degassed three times (one cycle of degassing) under vacuum before being refilled the tube with pre-dried dinitrogen (ca. 1 bar). The reaction was monitored by ^{1}H NMR spectroscopy (Figures S126 and S127). After one cycle of degassing, a significant amount of conversion (ca. 27%) of [{(^{iPrDip}nacnac)Mg}$_2$(μ-H)(μ-OH)] **4c** to [{(^{iPrDip}nacnac)Mg}$_2$(μ-O)] **1c** was observed. The solution was then degassed three more times and near full conversion (ca. 94%) of [{(^{iPrDip}nacnac)Mg}$_2$(μ-H)(μ-OH)] **4c** to [{(^{iPrDip}nacnac)Mg}$_2$(μ-O)] **1c** was observed by ^{1}H NMR spectroscopy.

H_2 exchange of [{(^{EtDip}nacnac)Mg}$_2$(μ-OD)(μ-D)] 4b-*d*$_2$

A colourless *in-situ*-prepared solution of [{(^{EtDip}nacnac)Mg}$_2$(μ-OD)(μ-D)] **4b-*d*$_2$** (10.3 mg, 10.7 μmol) in C_6D_6 (0.5 mL) in a J Young NMR tube was cooled to −196°C. The headspace was evacuated before refilling with pre-dried dihydrogen (ca. 1 bar) and sealing the tube. The solution was allowed to warm to room temperature and shaken vigorously (ca. 10 seconds). The reaction was monitored by ^{1}H NMR spectroscopy over time (Figures S128 and S129). The gradual consumption of [{(^{EtDip}nacnac)Mg}$_2$(μ-OD)(μ-D)] **4b-*d*$_2$** and formation of [{(^{EtDip}nacnac)Mg}$_2$(μ-H)(μ-OH)] **4b** (ca. 39% conversion) after 1001 hours (ca. 42 days) at room temperature was observed. In addition, the slow formation of small quantities of hydrogen deuteride, HD, was observed.

Exchange attempt of [{(^{EtDip}nacnac)Mg}$_2$(μ-H)(μ-OH)] 4b using [{(^{MeMes}nacnac)Mg}$_2$]

To a colourless solution of [{(^{EtDip}nacnac)Mg}$_2$(μ-H)(μ-OH)] **4b** (9.9 mg, 10.3 μmol, 1.0 equiv.) in C_6D_6 (0.6 mL) in a J Young NMR tube, was added [{(^{MeMes}nacnac)Mg}$_2$] (8.9 mg, 12.4 μmol, 1.2 equiv.) at room temperature. The reaction was monitored by ^{1}H NMR spectroscopy (Figure S130) over time and heated at 100°C for 46 hours. A small amount of [{(^{EtDip}nacnac)Mg}$_2$(μ-O)] **1b** (ca. 29%) was observed to form along with other unidentifiable nacac-containing compounds.

Dehydrogenation experiments using donor additions

4b and THF

To a colourless *in-situ* prepared solution of [{(^{EtDip}nacnac)Mg}$_2$(μ-H)(μ-OH)] **4b** (20.6 mg, 21.5 μmol, 1.0 equiv.) in C_6D_6 (0.6 mL) in a J Young NMR tube, was added THF (8.8 μL, 10.8 μmol, 0.5 equiv., from a 1.22 M THF stock solution in C_6D_6) at room temperature. The reaction was monitored by ^{1}H NMR spectroscopy (Figures S131 and S132) over time. Further 0.5 equivalents of THF were added until all starting material was consumed. Before the titration, a white precipitate was noted at the bottom of the J Young NMR tube. This was assumed to be [{(^{EtDip}nacnac)Mg}$_2$(μ-H)(μ-OH)] **4b** that had precipitated from solution at room temperature. A small amount of a white precipitate was present during the titration until approximately 10 equivalents of THF were added. Upon additions of THF to [{(^{EtDip}nacnac)Mg}$_2$(μ-H)(μ-OH)] **4b**, the gradual formation of [{(^{EtDip}nacnac)Mg(THF)}$_2$(μ-O)] **5b** was observed.

4c and THF

To a colourless *in-situ* prepared solution of [{(^{iPrDip}nacnac)Mg}$_2$(μ-H)(μ-OH)] **4c** (9.5 mg, 9.4 μmol, 1.0 equiv.) in C_6D_6 (0.6 mL) in a J Young NMR tube was added THF (16.0 μL, 2.4 μmol, 0.25 equiv., from a 0.15 M THF stock solution in C_6D_6) at room temperature. The reaction was monitored by ^{1}H NMR spectroscopy (Figures S133 and S134) over time. Further 0.25 equivalents of THF were added until all starting material was consumed. Upon additions of THF to [{(^{iPrDip}nacnac)Mg}$_2$(μ-H)(μ-OH)] **4c**, the formation of [{(^{iPrDip}nacnac)Mg(THF)}$_2$(μ-O)] **5c** was observed. After five equivalents of THF were added to the *in-situ* prepared solution of [{(^{iPrDip}nacnac)Mg}$_2$(μ-H)(μ-OH)] **4c** at room temperature, all [{(^{iPrDip}nacnac)Mg}$_2$(μ-H)(μ-OH)] **4c** had been observed to be consumed to form [{(^{iPrDip}nacnac)Mg(THF)}$_2$(μ-O)] **5c**.

4b and DMAP

To a colourless *in-situ* prepared solution of [{(^{EtDip}nacnac)Mg}$_2$(μ-H)(μ-OH)] **4b** (17.7 mg, 18.5 μmol, 1.0 equiv.) in C_6D_6 (0.6 mL) in a J Young NMR tube, was added DMAP (28 μL, 4.63 μmol, 0.25 equiv., from a 0.165 M DMAP stock solution in C_6D_6) at room temperature. The reaction was monitored by ^{1}H NMR spectroscopy over time (Figures S135 and S136). Further 0.25 equivalents of DMAP were added until all starting material was consumed. Upon additions of DMAP to [{(^{EtDip}nacnac)Mg}$_2$(μ-H)(μ-OH)] **4b**, the formation of [{(^{EtDip}nacnac)Mg(DMAP)}$_2$(μ-O)] **6b** as the major product was observed (ca. 43%). Before the titration, a white precipitate was noted at the bottom of the J Young NMR tube. This was assumed to be [{(^{EtDip}nacnac)Mg}$_2$(μ-H)(μ-OH)] **4b** that had precipitated out of solution at room temperature. During the titration, the formation of

[{(^{EtDip}nacnac)Mg(DMAP)(μ-OH)}$_2$] **8b** (ca. 32%, ^{1}H NMR resonance of Mg(μ-OH)$_2$: δ −0.92 ppm) and other nacac-containing reaction products were observed.

4c and DMAP

To a colourless *in-situ* prepared solution of [{(^{iPrDip}nacnac)Mg}$_2$(μ-H)(μ-OH)] **4c** (10.0 mg, 9.9 μmol, 1.0 equiv.) in C_6D_6 (0.6 mL) in a J Young NMR tube, was added DMAP (2.5 mg, 20 μmol, 2.1 equiv.) at room temperature. ^{1}H NMR spectroscopy was recorded as soon as possible. Immediate partial conversion of [{(^{iPrDip}nacnac)Mg}$_2$(μ-H)(μ-OH)] **4c** to [{(^{iPrDip}nacnac)Mg(DMAP)}$_2$(μ-O)] **6c** as the major product (ca. 61%) was observed. During the experiment, the formation of [{(^{iPrDip}nacnac)Mg(DMAP)(μ-OH)}$_2$] **8c** (ca. 13%, ^{1}H NMR resonance of Mg(μ-OH)$_2$: δ −0.70 ppm) and other nacac-containing reaction products were observed (Figures S137).

Attempt of donor solvent removal from 5b

To a colourless solution of isolated [{(^{EtDip}nacnac)Mg(THF)}$_2$(μ-O)] **5b** (15.0 mg, 13.6 μmol) in C_6D_6 (0.5 mL) in a J Young NMR tube, all volatiles were removed *in vacuo* to give a white residue. The white residue was heated for four hours at 60°C *in vacuo* (1.5-3.0 × 10^{-2} mbar) and then redissolved in C_6D_6 (0.6 mL). Regeneration of [{(^{EtDip}nacnac)Mg}$_2$(μ-O)] **1b** (ca. 32%) was observed via ^{1}H NMR spectroscopy (Figure S138). Additionally, the formation of [{(^{EtDip}nacnac)Mg(μ-OH)}$_2$] **3b** (ca. 52%, ^{1}H NMR resonance of Mg(μ-OH)$_2$: δ −0.50 ppm) and ^{EtDip}nacnacH (ca. 16%, resonance ^{1}H NMR resonance of backbone-C*H*: δ 5.07 ppm) were detected by ^{1}H NMR spectroscopy, indicating significant decomposition alongside THF removal.

1.2.5 Synthesis and reactions of magnesium hydroxide complexes

[{(^{EtDip}nacnac)Mg(μ-OH)}$_2$] 3b

Method 1 (*in-situ*): A colourless solution of [{(^{EtDip}nacnac)Mg(μ-H)}$_2$] **7b** (10.0 mg, 10.6 μmol) in C_6D_6 (0.5 mL) in a J Young NMR tube was cooled to −70°C. The headspace was evacuated before refilling with pre-dried nitrous oxide (ca. 1 bar) and sealing the tube (one cycle of nitrous oxide only). The solution was allowed to warm to room temperature and the J Young tube was shaken vigorously (ca. 10 seconds). ^{1}H NMR spectroscopy was recorded as soon as possible. Formation of [{(^{EtDip}nacnac)Mg}$_2$(μ-H)(μ-OH)] **4b** (ca. 54%) and [{(^{EtDip}nacnac)Mg}$_2$(μ-O)] **1b** (ca. 31%) were observed by ^{1}H NMR spectroscopy as the major intermediates, in addition to other minor nacac-containing reaction products. The solution was re-cooled to −70°C, and the headspace was evacuated before refilling with pre-dried nitrous oxide (ca. 1 bar) and sealing the tube (second cycle of nitrous oxide). The solution was allowed to warm to room temperature and the J Young tube was shaken vigorously (ca. 10 seconds). ^{1}H NMR spectroscopy was recorded as soon as possible. Formation of [{(^{EtDip}nacnac)Mg(μ-OH)}$_2$] **3b** was observed by ^{1}H NMR spectroscopy (^{1}H NMR resonance of Mg(μ-OH)$_2$: δ −0.50 ppm) as the major product (ca. 56%).

Method 2 (isolated): A colourless solution of [{(^{EtDip}nacnac)Mg(μ-H)}$_2$] **7b** (110.0 mg, 116.8 μmol) in toluene (15 mL) in a J Young flask (250 mL) was cooled to −70°C. The headspace was evacuated before refilling with pre-dried nitrous oxide (ca. 1 bar) and sealing the flask. This was then allowed to warm to room temperature, shaken vigorously (ca. 10 seconds) and stirred for one hour. All volatiles were subsequently removed *in vacuo* and the residue was extracted with *n*-hexane (10 mL), filtered, concentrated *in vacuo* (to ca. 5 mL), and stored at −40°C overnight to give a white precipitate. The precipitate was filtered and the solid dried *in vacuo* (ca. 10 minutes, room temperature) yielding [{(^{EtDip}nacnac)Mg(μ-OH)}$_2$] **3b** as a white powder. Concentration of the supernatant solution (ca. 1 mL) and storage at −40°C yielded further crops of [{(^{EtDip}nacnac)Mg(μ-OH)}$_2$] **3b**. Crystals suitable for X-ray crystallographic analysis were grown from a concentrated benzene solution. Yield: 55.5 mg (49%); ^{1}H NMR (500.1 MHz, C_6D_6, 298 K) δ −0.50 (s, 2H, Mg-O*H*), 0.89 (d, J_{HH} = 6.9 Hz, 24H, Ar-*o*-CH(C*H*$_3$)$_2$), 0.94 (t, J_{HH} = 7.5 Hz, 12H, NCCH$_2$C*H*$_3$), 1.13 (d, J_{HH} = 6.8 Hz, 24H, Ar-*o*-CH(C*H*$_3$)$_2$), 1.88 (q, J_{HH} = 7.5 Hz, 8H, NCC*H*$_2$CH$_3$), 3.09 (sept, J_{HH} = 6.8 Hz, 8H, Ar-*o*-C*H*(CH$_3$)$_2$), 4.95 (s, 2H, NCC*H*CN), 7.01–7.13 (m, 12H, Ar-*H*). ^{13}C{^{1}H} NMR (125.8 MHz, C_6D_6, 298 K) δ 13.4 (NCCH$_2$*C*H$_3$), 24.1 (Ar-*o*-CH(*C*H$_3$)$_2$), 24.8 (Ar-*o*-CH(*C*H$_3$)$_2$), 28.1 (Ar-*o*-*C*H(CH$_3$)$_2$), 28.7 (NC*C*H$_2$CH$_3$), 89.1 (NC*C*HCN), 124.0 (Ar-*C*), 125.2 (Ar-*C*), 142.4 (Ar-*C*), 146.4 (Ar-*C*), 173.4 (N*C*CHC*N*).

[{(^{iPrDip}nacnac)Mg(μ-OH)}$_2$] 3c

A colourless, *in-situ* prepared solution of [{(^{iPrDip}nacnac)Mg}$_2$(μ-O)] **1c** (12.0 mg, 11.9 μmol) in C_6D_6 (0.6 mL) in a J Young NMR tube, still containing some residual N_2O (not degassed), was cooled to −196°C. The headspace was evacuated before refilling with pre-dried dihydrogen (ca. 1 bar) and sealing the tube. The solution was allowed to warm to room temperature and shaken vigorously (ca. 10 seconds). ^{1}H NMR spectroscopy was recorded as soon as possible. Immediate and complete conversion of **1c** to [{(^{iPrDip}nacnac)Mg(μ-OH)}$_2$] **3c** at room temperature was observed via ^{1}H NMR spectroscopy. Separately, a long crystallisation attempt of [{(^{iPrDip}nacnac)Mg}$_2$(μ-H)(μ-OH)] **4c** from a concentrated benzene solution yielded few colourless crystals of [{(^{iPrDip}nacnac)Mg(μ-OH)}$_2$] **3c** suitable for X-ray crystallographic analysis. ^{1}H NMR (400.1 MHz, C_6D_6, 295 K) δ −0.54 (s, 2H, Mg-(O*H*)$_2$), 0.86 (d, J_{HH} = 6.8 Hz, 24H, Ar-*o*-CH(C*H*$_3$)$_2$), 0.98 (d, J_{HH} = 6.7 Hz, 24H, NCCH(C*H*$_3$)$_2$), 1.21 (d, J_{HH} = 6.8 Hz, 24H, Ar-*o*-CH(C*H*$_3$)$_2$), 2.41 (sept, J_{HH} = 6.9 Hz, 4H, NCC*H*(CH$_3$)$_2$), 3.11 (sept, J_{HH} = 6.7 Hz, 8H, Ar-*o*-C*H*(CH$_3$)$_2$), 4.86 (s, 2H, NCC*H*CN), 7.01-7.15 (m, 12H, Ar-*H*). ^{13}C{^{1}H} NMR (100.6 MHz, C_6D_6, 295 K) δ 23.5 (NCCH(*C*H$_3$)$_2$), 24.4 (Ar-*o*-CH(*C*H$_3$)$_2$), 25.7 (Ar-*o*-CH(*C*H$_3$)$_2$), 27.7 (Ar-*o*-*C*H(CH$_3$)$_2$), 31.5 (NC*C*H(CH$_3$)$_2$), 85.1 (NC*C*HCN), 124.1 (Ar-*C*), 125.1 (Ar-*C*), 142.9 (Ar-*C*), 145.7 (Ar-*C*), 178.4 (N*C*CH*C*N).

Reaction of [{(^{EtDip}nacnac)Mg(μ-OH)}$_2$] 3b with [(^{EtDip}nacnac)Mg*n*Bu] 9b

To a colourless solution of [{(^{EtDip}nacnac)Mg(μ-OH)}$_2$] **3b** (20.0 mg, 20.5 μmol, 1.0 equiv.) in C_6D_6 (0.6 mL) in a J Young NMR tube was added [(^{EtDip}nacnac)Mg*n*Bu] **9b** (21.6 mg, 41.1 μmol, 2.0 equiv.) at room temperature. The reaction was heated at 100°C for 48 hours and progress was monitored by ^{1}H NMR spectroscopy over time. A minor amount (ca. 7%) of [{(^{EtDip}nacnac)Mg}$_2$(μ-O)] **1b** was observed to have formed. To test if the reaction can be accelerated by THF addition, THF (13.4 μL, 164 μmol, 8.0 equiv.) was added to the J Young NMR tube at room temperature. The reaction was monitored by ^{1}H NMR spectroscopy over time and was heated at 80°C for 36 hours. A minor amount (ca. 9% total) of [{(^{EtDip}nacnac)Mg(THF)}$_2$(μ-O)] **5b** was observed to form. Colourless crystals suitable for X-ray crystallographic analysis were grown from the concentrated C_6D_6 solution and were analysed as [{(^{EtDip}nacnac)Mg(THF)}$_2$(μ-O)] **5b**.

Reaction of [{(^{EtDip}nacnac)Mg(μ-OH)}$_2$] 3b with [{(^{EtDip}nacnac)Mg(μ-H)}$_2$] 7b

To a colourless solution of isolated [{(^{EtDip}nacnac)Mg(μ-OH)}$_2$] **3b** (22.0 mg, 22.6 μmol, 1.0 equiv.) in C_6D_6 (0.6 mL) in a J Young NMR tube, was added [{(^{EtDip}nacnac)Mg(μ-H)}$_2$] **7b** (23.4 mg, 24.8 μmol, 1.1 equiv.) at room temperature. The reaction was monitored by ^{1}H NMR spectroscopy over time and was heated at 100°C for 105 hours. After the addition of [{(^{EtDip}nacnac)Mg(μ-H)}$_2$] **7b** to the J Young NMR tube, a white precipitate was noted at the bottom of the J Young NMR tube.

This was assumed to be [{(^{EtDip}nacnac)Mg(μ-H)}$_2$] **7b** that had not yet dissolved into the solution. A trace amount (ca. 1%) of [{(^{EtDip}nacnac)Mg}$_2$(μ-O)] **1b** was observed to have formed. To test if the reaction can be accelerated by THF addition, THF (18.4 μL, 227 μmol, 10.0 equiv.) was added to the J Young NMR tube (forming [{(^{EtDip}nacnac)Mg(THF)(μ-OH)}$_2$] **10b** and [{(^{EtDip}nacnac)Mg(THF)(μ-H)}$_2$] **11b**) at room temperature. The reaction was heated at 100°C for 63 hours and was monitored by ^{1}H NMR spectroscopy over time. A small amount (ca. 5% total) of [{(^{EtDip}nacnac)Mg(THF)}$_2$(μ-O)] **5b** was observed to form. A few colourless crystals of [{(^{EtDip}nacnac)Mg(THF)}$_2$(μ-O)] **5b** suitable for X-ray crystallographic analysis were grown from the concentrated C_6D_6 solution.

[{(^{EtDip}nacnac)Mg(DMAP)(μ-OH)}$_2$] 8b

To a colourless solution of isolated [{(^{EtDip}nacnac)Mg(μ-OH)}$_2$] **3b** (12.2 mg, 12.5 μmol, 1.0 equiv.) in C_6D_6 (0.6 mL) in a J Young NMR tube was added DMAP (3.1 mg, 25 μmol, 2.0 equiv.) at room temperature. ^{1}H NMR spectroscopy was recorded as soon as possible. ^{1}H NMR spectroscopy showed immediate and complete conversion from [{(^{EtDip}nacnac)Mg(μ-OH)}$_2$] **3b** to [{(^{EtDip}nacnac)Mg(DMAP)(μ-OH)}$_2$] **8b** at room temperature. Colourless crystals suitable for X-ray crystallographic analysis were grown from a concentrated benzene solution. ^{1}H NMR (499.9 MHz, C_6D_6, 298 K) δ −0.95 (s, 2H, Mg-O*H*), 0.96 (d, J_{HH} = 6.9 Hz, 24H, Ar-*o*-CH(C*H*$_3$)$_2$), 1.08 (t, J_{HH} = 7.5 Hz, 12H, NCCH$_2$C*H*$_3$), 1.15 (d, J_{HH} = 6.9 Hz, 24H, Ar-*o*-CH(C*H*$_3$)$_2$), 2.03 (q, J_{HH} = 7.5 Hz, 8H, NCC*H*$_2$CH$_3$), 2.23 (s, 12H, N(C*H*$_3$)$_2$), 3.13 (sept, J_{HH} = 6.9 Hz, 8H, Ar-*o*-C*H*(CH$_3$)$_2$), 5.06 (s, 2H, NCC*H*CN), 6.09-6.14 (m, 4H, Ar$_{DMAP}$-*H*), 7.12-7.16 (m, 12H, Ar-*H*), 8.22-8.27 (m, 4H, Ar$_{DMAP}$-*H*). ^{13}C{^{1}H} NMR (125.7 MHz, C_6D_6, 298 K) δ 13.6 (NCCH$_2$*C*H$_3$), 24.8 (Ar-*o*-CH(*C*H$_3$)$_2$), 25.7 (Ar-*o*-CH(*C*H$_3$)$_2$), 28.1 (Ar-*o*-*C*H(CH$_3$)$_2$), 28.9 (NC*C*H$_2$CH$_3$), 38.3 (N(*C*H$_3$)$_2$), 88.5 (NC*C*HCN), 106.5 (Ar$_{DMAP}$-*C*), 124.1 (Ar-*C*), 124.2 (Ar-*C*), 142.4 (Ar-*C*), 148.2 (Ar-*C*), 150.6 (Ar$_{DMAP}$-*C*), 154.2 (Ar$_{DMAP}$-*C*), 172.2 (N*C*CH*C*N).

Reaction of [{(^{EtDip}nacnac)Mg(DMAP)(μ-OH)}$_2$] 8b with [{(^{EtDip}nacnac)Mg(DMAP)H] 12b

To a colourless *in-situ* prepared solution of [{(^{EtDip}nacnac)Mg(DMAP)(μ-OH)}$_2$] **8b** (15.2 mg, 12.5 μmol, 1.0 equiv.) in C_6D_6 (0.4 mL) was added a colourless *in-situ* prepared solution of [{(^{EtDip}nacnac)Mg(DMAP)H] **12b** (15.6 mg, 26.3 μmol, 2.1 equiv.) in C_6D_6 (0.4 mL) in a J Young NMR tube at room temperature. The reaction was monitored by ^{1}H NMR spectroscopy over time and heated at 60°C for 23 hours, during which the solution colour changed from colourless to brown. Consumption of the starting substrates was observed via ^{1}H NMR spectroscopy to yield a small amount (ca. 6%) of [{(^{EtDip}nacnac)Mg(DMAP)}$_2$(*μ*-O)] **6b**, in addition to unidentifiable resonances for a mixture of nacac-containing compounds.

1.2.6 Hydrolysis experiments

Hydrolysis of [{(^{EtDip}nacnac)Mg}$_2$] 2b

To a yellow solution of [{(^{EtDip}nacnac)Mg}$_2$] **2b** (10.7 mg, 11.4 μmol, 1.0 equiv.) in C_6D_6 (0.4 mL) in a J Young NMR tube, was added a sub-stoichiometric amount of water (597 μL, 3.8 μmol, 0.3 equiv., from a 6.4 mM stock solution of water in C_6D_6) at room temperature. The reaction was monitored by ^{1}H NMR spectroscopy over time (Figure S161). Upon the initial addition of sub-stoichiometric amounts of water, the formation of [{(^{EtDip}nacnac)Mg(μ-OH)}$_2$] **3b** (ca. 4%, ^{1}H NMR resonance of Mg(μ-OH)$_2$: δ −0.50 ppm) and ^{EtDip}nacnacH (ca. 23%, ^{1}H NMR resonance of backbone-C*H*: δ 5.07 ppm) were observed via ^{1}H NMR spectroscopy for the converted fraction. Further addition of water (597 μL, 3.8 μmol, 0.3 equiv., from the same stock solution in C_6D_6) was added to the solution at room temperature and further formation of [{(^{EtDip}nacnac)Mg(μ-OH)}$_2$] **3b** (ca. 6% total) and ^{EtDip}nacnacH (ca. 33% total) were observed via ^{1}H NMR spectroscopy. During the experiment, no formation of [{(^{EtDip}nacnac)Mg}$_2$(μ-H)(μ-OH)] **4b** was observed.

Hydrolysis of [{(^{EtDip}nacnac)Mg(μ-H)}$_2$] 7b

To a colourless solution of isolated [{(^{EtDip}nacnac)Mg(μ-H)}$_2$] **7b** (20.6 mg, 21.9 μmol, 1.0 equiv.) in C_6D_6 (0.4mL) in a J Young NMR tube, was added a sub-stoichiometric amount of water (686 μL, 4.37 μmol, 0.2 equiv., from a 6.4 mM water stock solution in C_6D_6) at room temperature. The reaction was monitored by ^{1}H NMR spectroscopy over time (Figure S162). Upon the initial addition of sub-stoichiometric amounts of water, the formation of [{(^{EtDip}nacnac)Mg(μ-OH)}$_2$] **3b** (ca. 5%, ^{1}H NMR resonance of Mg(μ-OH)$_2$: δ −0.50 ppm) was observed by ^{1}H NMR spectroscopy for the converted fraction. A second addition of a sub-stoichiometric amount of water (1.71 mL, 10.9 μmol, 0.5 equiv., from the same stock solution in C_6D_6) was added to the solution at room temperature and further formation of [{(^{EtDip}nacnac)Mg(μ-OH)}$_2$] **3b** (ca. 10% total) and ^{EtDip}nacnacH (ca. 31% total, ^{1}H NMR resonance of backbone-C*H*: δ 5.07 ppm) were observed via ^{1}H NMR spectroscopy. After the final addition of the sub-stoichiometric amount of water (1.03 mL, 6.56 μmol, 0.3 equiv., from the same stock solution in C_6D_6) was added to the solution at room temperature, the full consumption of [{(^{EtDip}nacnac)Mg(μ-H)}$_2$] **7b** to form [{(^{EtDip}nacnac)Mg(μ-OH)}$_2$] **3b** (ca. 10% total) and ^{EtDip}nacnacH (ca. 90% total) was observed via ^{1}H NMR spectroscopy. During the experiment, no formation of [{(^{EtDip}nacnac)Mg}$_2$(μ-H)(μ-OH)] **4b** was observed.

1.2.7 Hydrogenation experiments of DPE

1,1-diphenylethylene (DPE) was used as an example alkene to test the low-pressure hydrogenation.

Control reaction (no catalyst)

A colourless solution of DPE (4.0 µL, 23 µmol) in C_6D_6 (0.6 mL) in a J Young NMR tube was cooled to −196°C. The headspace was evacuated before refilling with pre-dried dihydrogen (ca. 1 bar) and sealing the tube. The solution was allowed to warm to room temperature and shaken vigorously (ca. 10 seconds). The reaction was monitored by ^{1}H NMR spectroscopy over time and was heated at 100°C for 288 hours. No formation of 1,1-diphenylethane was observed.

Hydrogenation of DPE using catalytic 4a

To a colourless *in-situ* prepared solution of [{(^{MeDip}nacnac)Mg}$_2$(µ-H)(µ-OH)] **4a** (16.5 mg, 18.3 µmol, 14.0 mol%) in C_6D_6 (0.6 mL) in a J Young NMR tube, was added DPE (23.1 µL, 131 µmol, 7.1 equiv.) at room temperature. The solution was then cooled to −196°C, the headspace was evacuated before refilling with pre-dried dihydrogen (ca. 1 bar) and sealing the tube. The reaction was monitored by ^{1}H NMR spectroscopy over time and was heated at 100°C for 700 hours. After heating the solution for 456 hours, the J Young NMR tube was re-filled with pre-dried dihydrogen (ca. 1 bar). During the experiment, the near quantitative consumption of DPE and the formation of 1,1-diphenylethane was observed. Yield = 95%. Turnover number (TON) = 6.82. Turnover frequency $9.7 \cdot 10^{-3}$ h^{-1}. Please note that the reaction was stopped after 700 hours, but the catalyst system was not inactive yet, so further turnover could have been generated. During the reaction, [{(^{MeDip}nacnac)Mg}$_2$(µ-H)(µ-OH)] **4a** was found to be the dominant nacnac-containing product, i.e., the resting state in the mixture. 1,1-diphenylethane ^{1}H NMR (499.9 MHz, C_6D_6, 298 K) δ 1.46 (d, J_{HH} = 7.3 Hz, 3H, C*H*$_3$CCH(Ph)$_2$), 3.93 (q, J_{HH} = 7.2 Hz, 1H, CH$_3$CC*H*(Ph)$_2$), 7.02-7.15 (m, 10H, Ar-*H*).

Hydrogenation of DPE using catalytic 4a and a higher H_2 pressure

To a colourless *in-situ* prepared solution of [{(^{MeDip}nacnac)Mg}$_2$(µ-H)(µ-OH)] **4a** (15.2 mg, 16.9 µmol, 12.6 mol%) in C_6D_6 (0.6 mL) in a J Young NMR tube, was added DPE (23.6 µL, 133.8 µmol, 7.9 equiv.) at room temperature. The solution was then cooled to −196°C, the headspace was evacuated before refilling with pre-dried dihydrogen with an estimated pressure of ca. 2(-3) bar (achieved via a different cooling regime for the storage flask) and sealing the tube (the exact pressure could not be determined). The reaction was monitored by ^{1}H NMR spectroscopy over time and was heated at 100°C for 237 hours. During the experiment, the consumption of DPE and the formation of 1,1-diphenylethane was observed. Yield = 99%. TON = 7.84. TOF = $3.3 \cdot 10^{-2}$ h^{-1}. Please note that the

reaction was stopped at this state of conversion, but the catalyst system was not inactive yet, so further turnover could have been generated. During the reaction, [{(^{MeDip}nacnac)Mg}$_2$(μ-H)(μ-OH)] **4a** was found to be the dominant nacnac-containing product, i.e., the resting state in the mixture. Although the H_2 pressure was only estimated, the experiment showed that hydrogenation is strongly dependent on gas pressure and that the reaction can go to completion. Please note that all other reported hydrogenation experiments used ca. 1 bar gas pressure as described in 1.1.2.

Hydrogenation of DPE using catalytic 4b

To a colourless *in-situ* prepared solution of [{(^{EtDip}nacnac)Mg}$_2$(μ-H)(μ-OH)] **4b** (14.3 mg, 14.9 μmol, 15.2 mol%) in C_6D_6 (0.6 mL) in a J Young NMR tube, was added DPE (17.4 μL, 98.5 μmol, 6.6 equiv.) at room temperature. The solution was then cooled to −196°C, the headspace was evacuated before refilling with pre-dried dihydrogen (ca. 1 bar) and sealing the tube. The reaction was monitored by ^{1}H NMR spectroscopy over time and was heated at 100°C for 700 hours. After heating the solution for 496 hours, the J Young NMR tube was re-filled with pre-dried dihydrogen (ca. 1 bar). During the experiment, the partial consumption of DPE and the formation of 1,1-diphenylethane was observed. Yield = 28%. TON = 1.82. TOF = $2.6 \cdot 10^{-3}$ h^{-1}. Please note that the reaction was stopped after 700 hours, but the catalyst system was not inactive yet, so further turnover could have been generated. During the reaction, [{(^{EtDip}nacnac)Mg}$_2$(μ-H)(μ-OH)] **4b** was found to be the dominant nacnac-containing product, i.e., the resting state in the mixture.

Stoichiometric reaction of DPE with 4b

To a colourless solution of isolated [{(^{EtDip}nacnac)Mg}$_2$(μ-H)(μ-OH)] **4b** (10.1 mg, 10.5 μmol, 1.0 equiv.) in C_6D_6 (0.5 mL) in a J Young NMR tube, was added DPE (7.4 μL, 42 μmol, 4.0 equiv.) at room temperature. The reaction was monitored by ^{1}H NMR spectroscopy over time and was heated at 100°C for 93 hours. During the experiment, the consumption of [{(^{EtDip}nacnac)Mg}$_2$(μ-H)(μ-OH)] **4b** and the formation of [{(^{EtDip}nacnac)Mg}$_2$(μ-O)] **1b**, in addition to a trace amount of 1,1-diphenylethane was observed by ^{1}H NMR spectroscopy. The formation of [{(^{EtDip}nacnac)Mg(μ-OH)}$_2$] **3b** from decomposition was also observed.

Hydrogenation of DPE using catalytic 4c

To a colourless *in-situ* prepared solution of [{(^{iPrDip}nacnac)Mg}$_2$(μ-H)(μ-OH)] **4c** (15.1 mg, 14.9 μmol, 14.9 mol%) in C_6D_6 (0.6 mL) in a J Young NMR tube, was added DPE (17.6 μL, 99.8 μmol, 6.71 equiv.) at room temperature. The solution was then cooled to −196°C, the headspace was evacuated before refilling with pre-dried dihydrogen (ca. 1 bar) and sealing the tube. The reaction was monitored by ^{1}H NMR spectroscopy over time and was heated at 100°C for 700 hours. After

heating the solution for 20, 40 and 439 hours respectively, the J Young NMR tube was re-filled with pre-dried dihydrogen (ca. 1 bar). During the experiment, the partial consumption of DPE and the formation of 1,1-diphenylethane was observed. Yield = 9%. TON = 0.61. TOF = $8.7 \cdot 10^{-4}$ h^{-1}. Please note that the reaction was stopped after 700 hours (~1 month), but the catalyst system was not inactive yet, so further turnover could have been generated. The reaction for this ligand is, however, very slow, and therefore it was impractical to continue to a higher yield and turnover. The hydrogenation catalysis experiments using **4a**, **4b**, and **4c** as catalysts, respectively, show the large influence of the backbone substitution and ligand sterics for this reaction. During the reaction, [{(^{iPrDip}nacnac)Mg}$_2$(μ-H)(μ-OH)] **4c** was found to be the dominant nacnac-containing product, i.e., the resting state in the mixture.

Hydrogenation of DPE using catalytic 7b

To a colourless solution of [{(^{EtDip}nacnac)Mg(μ-H)}$_2$] **7b** (10.0 mg, 10.6 μmol, 15.9 mol%) in C_6D_6 (0.6 mL) in a J Young NMR tube, was added DPE (11.8 μL, 66.9 μmol, 6.30 equiv.) at room temperature. The solution was then cooled to −196°C, the headspace was evacuated before refilling with pre-dried dihydrogen (ca. 1 bar) and sealing the tube. Upon the initial addition of DPE and dihydrogen to the J Young NMR tube, a white precipitate was observed. This was assumed to be [{(^{EtDip}nacnac)Mg(μ-H)}$_2$] **7b**. The reaction was monitored by ^{1}H NMR spectroscopy over time and was heated at 100°C for 700 hours. During the experiment, the partial consumption of DPE and the formation of 1,1-diphenylethane was observed. Yield = 61%. TON = 3.85. TOF = $5.5 \cdot 10^{-3}$ h^{-1}. Please note that the reaction was stopped after 700 hours, but the catalyst system was not inactive yet, so further turnover could have been generated. During the reaction, [{(^{EtDip}nacnac)Mg(μ-H)}$_2$] **7b** was found to be the dominant nacnac-containing product, i.e., the resting state in the mixture.

Hydrogenation of DPE using catalytic 7b using a lower H_2 amount/pressure

To a colourless solution of [{(^{EtDip}nacnac)Mg(μ-H)}$_2$] **7b** (12.5 mg, 13.3 μmol, 7.0 mol%) in C_6D_6 (0.6 mL) in a J Young NMR tube, was added DPE (33.6 μL, 190 μmol, 14.3 equiv.) at room temperature. The solution was then cooled to −40°C, the headspace was evacuated before refilling with pre-dried dihydrogen and sealing the tube – this filling procedure led to less hydrogen being added to the tube compared to the experimental procedure reported above. The reaction was monitored by ^{1}H NMR spectroscopy (Figure S170) over time and was heated at 100°C for 88 hours. ^{1}H NMR spectroscopy showed the major product (ca. 63%) to be [(^{EtDip}nacnac)Mg{CPh_2CH_3}] (see next experiment, backbone-C*H* resonance at 5.11 ppm and Mg-CPh_2CH_3 resonance at 1.22 ppm) in addition to unreacted [{(^{EtDip}nacnac)Mg(μ-H)}$_2$] **7b** (ca. 2%) other nacac-containing reaction products (ca. 26%). In addition, ^{1}H NMR spectroscopy indicates the formation of small quantities of

a complex likely to be isomeric [(^{EtDip}nacnac)Mg{CH_2CHPh_2}] (ca. 3%, backbone-C*H* resonance at 5.06 ppm, Mg-CH_2CHPh_2 resonance at 3.77 ppm and CH_2CHPh_2 resonance at 0.43 ppm), plus small quantities of 1,1-diphenylethane (ca. 6%, C*H*$_3$ resonance at 1.46 ppm and CPh$_2$*H* resonance at 4.93 ppm) during the experiment. This experiment showed that predominantly slow hydromagnesiation of DPE occurs under these conditions.

Stoichiometric reaction of DPE with 7b

To a colourless solution of [{(^{EtDip}nacnac)Mg(μ-H)}$_2$] **7b** (17.8 mg, 18.9 μmol, 1.0 equiv.) in C_6D_6 (0.6 mL) in a J Young NMR tube was added DPE (9.2 μL, 52 μmol, 2.8 equiv.) at room temperature. The reaction was monitored by ^{1}H NMR spectroscopy (Figure S176) and was heated at 100°C for 882 hours. ^{1}H NMR spectroscopy showed the major product (ca. 39%) to be presumed [(^{EtDip}nacnac)Mg{CPh_2CH_3}] (backbone-C*H* resonance at 5.11 ppm and Mg-CPh$_2$C*H*$_3$ resonance at 1.22 ppm). The formation of [(^{EtDip}nacnac)Mg{CH_2CHPh_2}] (ca. 12%, backbone-C*H* resonance at 5.06 ppm, Mg-CH$_2$C*H*Ph$_2$ resonance at 3.77 ppm and C*H*$_2$CHPh$_2$ resonance at 0.43 ppm), in addition to unreacted [{(^{EtDip}nacnac)Mg(μ-H)}$_2$] (ca. 9%) and other nacac-containing reaction products (ca. 14%) was observed by ^{1}H NMR spectroscopy. In addition, the formation of 1,1-diphenylethane (ca. 25%, C*H*$_3$ resonance at 1.46 ppm and CPh$_2$*H* resonance at 4.93 ppm) was observed by ^{1}H NMR spectroscopy during the experiment after prolonged time. This reaction provided a less clean formation of [(^{EtDip}nacnac)Mg{CPh_2CH_3}] compared with the experiment reported above that used a small amount of added hydrogen gas. So far, the main product, [(^{EtDip}nacnac)Mg{CPh_2CH_3}], could not be isolated or purified by crystallisation. The combination of NMR data from this and the previous experiment reported above, and 2D NMR spectra, were analysed to elucidate the connectivity (Figures S170-S177). Data for [(^{EtDip}nacnac)Mg{CPh_2CH_3}]: ^{1}H NMR (499.9 MHz, C_6D_6, 298 K) δ 0.91-0.99 (m, 18H, NCCH$_2$C*H*$_3$ & Ar-*o*-CH(C*H*$_3$)$_2$), 1.08 (d, J_{HH} = 6.8 Hz, 12H, Ar-*o*-CH(C*H*$_3$)$_2$), 1.22 (s, 3H, Mg-CPh$_2$C*H*$_3$), 2.05 (q, J_{HH} = 7.5 Hz, 4H, NCC*H*$_2$CH$_3$), 3.05 (sept, J_{HH} = 6.5 Hz, 4H, Ar-*o*-CH(C*H*$_3$)$_2$), 5.11 (s, 1H, NCC*H*CN), 6.81-6.89 (m, 6H, Ph-*H*), 6.97-7.03 (m, 4H, Ph-*H*) 7.04-7.14 (m, 6H, Ar-*H*). ^{13}C{^{1}H} NMR (125.7 MHz, C_6D_6, 298 K) δ 13.1 (NCCH$_2$*C*H$_3$), 24.2 (Ar-*o*-CH(*C*H$_3$)$_2$), 24.5 (Ar-*o*-CH(*C*H$_3$)$_2$), 27.0 (Mg-CPh$_2$*C*H$_3$), 28.7 (NC*C*H$_2$CH$_3$), 28.8 (Ar-*o*-*C*H(CH$_3$)$_2$), 45.9 (Mg-*C*Ph$_2$CH$_3$), 90.1 (NC*C*HCN), 121.9 (Ph-*C*), 124.5 (Ar-*C*), 126.2 (Ar-*C*), 127.4 (Ph-*C*), 129.0 (Ph-*C*), 142.0 (Ar-*C*), 142.3 (Ar-*C*), 144.0 (Ar-*C*), 155.8 (Ph-*C*), 175.2 (N*C*CHC*N*).

2 NMR spectroscopy

2.1 General considerations

NMR spectra were recorded in deuterated benzene; further details are given in the figure captions. Chemical shifts are given in ppm. In some samples, resonances of residual solvent and/or silicone grease (as impurities) may be present; for example silicone grease (literature values: ^{1}H: 0.29 ppm, ^{13}C{^{1}H}: 1.38 ppm), toluene (^{1}H: 2.11, 7.02, 7.13 ppm, ^{13}C{^{1}H}: 21.10, 125.68, 128.56, 129.33, 137.91 ppm), and *n*-hexane (^{1}H: 0.89, 1.24 ppm, ^{13}C{^{1}H}: 14.32, 23.04, 31.96 ppm).[8] [{(^{iPrDip}nacnac)Mg}$_2$(μ-O)] **1c** and [{(^{iPrDip}nacnac)Mg(THF)}$_2$(μ-O)] **5c** have been reported previously but are included here for completeness and comparison.[4] [{(^{MeDip}nacnac)Mg}$_2$(μ-O)] **1a** has been reported previously, but no images of NMR spectra were reported.[2] The order of spectra broadly follows the order of the experimental section.

2.2 Synthesis of $[\{(^{EtDip}nacnac)Mg\}_2]$ 2b and precursors

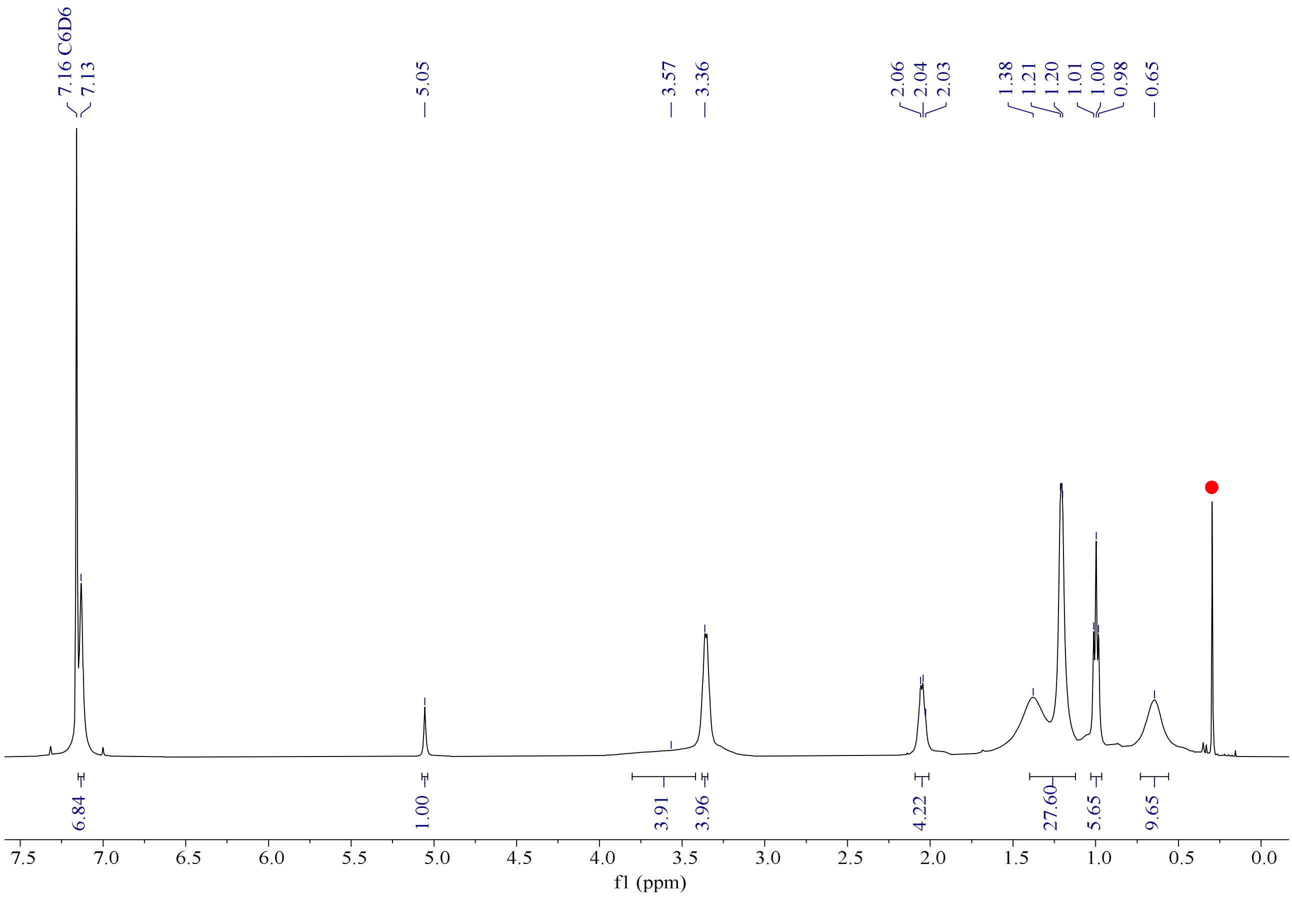

Figure S1. ^{1}H NMR spectrum (499.9 MHz, C_6D_6, 298 K) of isolated $[(^{EtDip}nacnac)Mg(OEt_2)I]$. The red circle denotes the resonance associated with silicone grease.

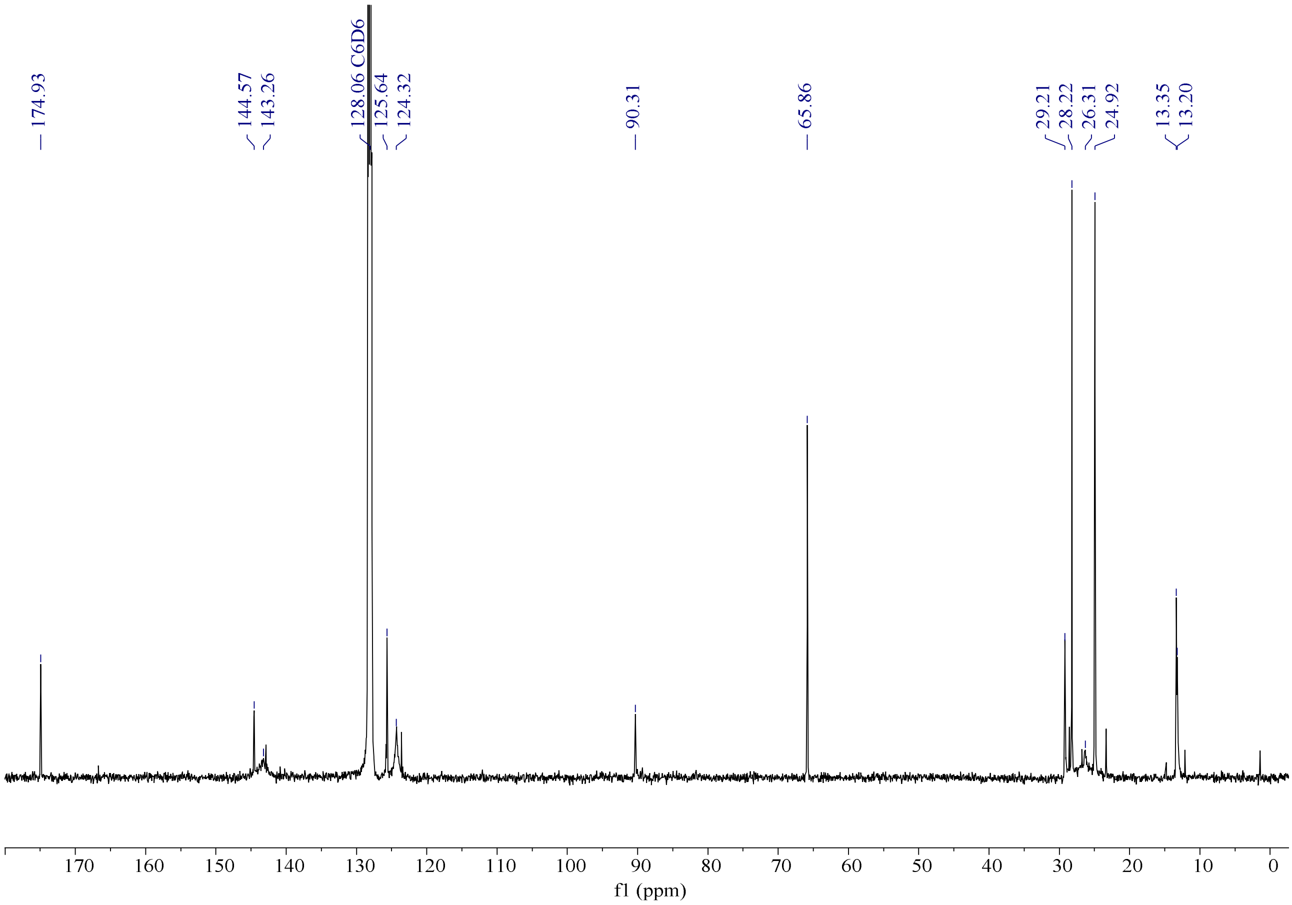

Figure S2. $^{13}C\{^1H\}$ NMR spectrum (125.7 MHz, C_6D_6, 298 K) of isolated [(^{EtDip}nacnac)Mg(OEt_2)I].

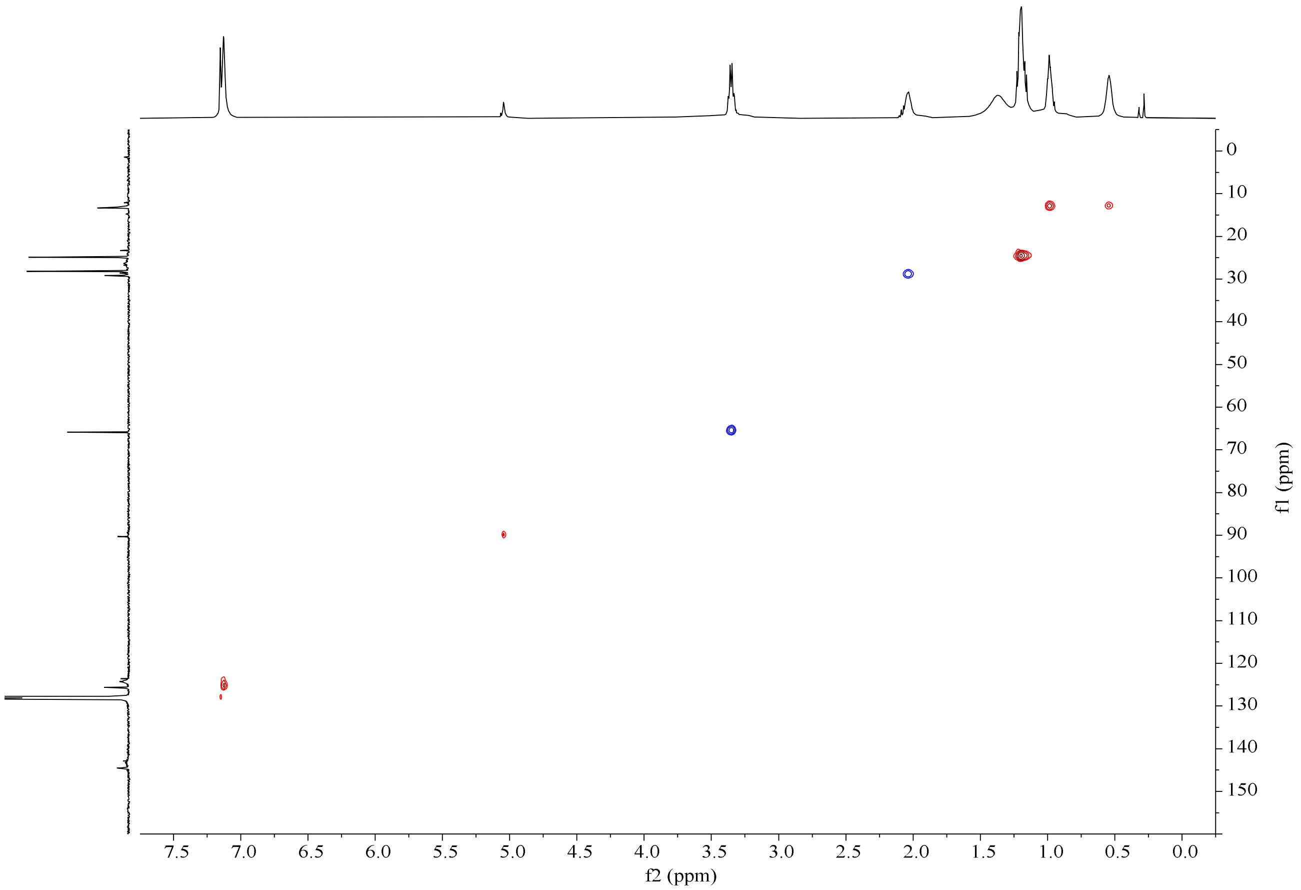

Figure S3. ^{1}H-^{13}C HSQC NMR spectrum of isolated [(^{EtDip}nacnac)Mg(OEt_2)I].

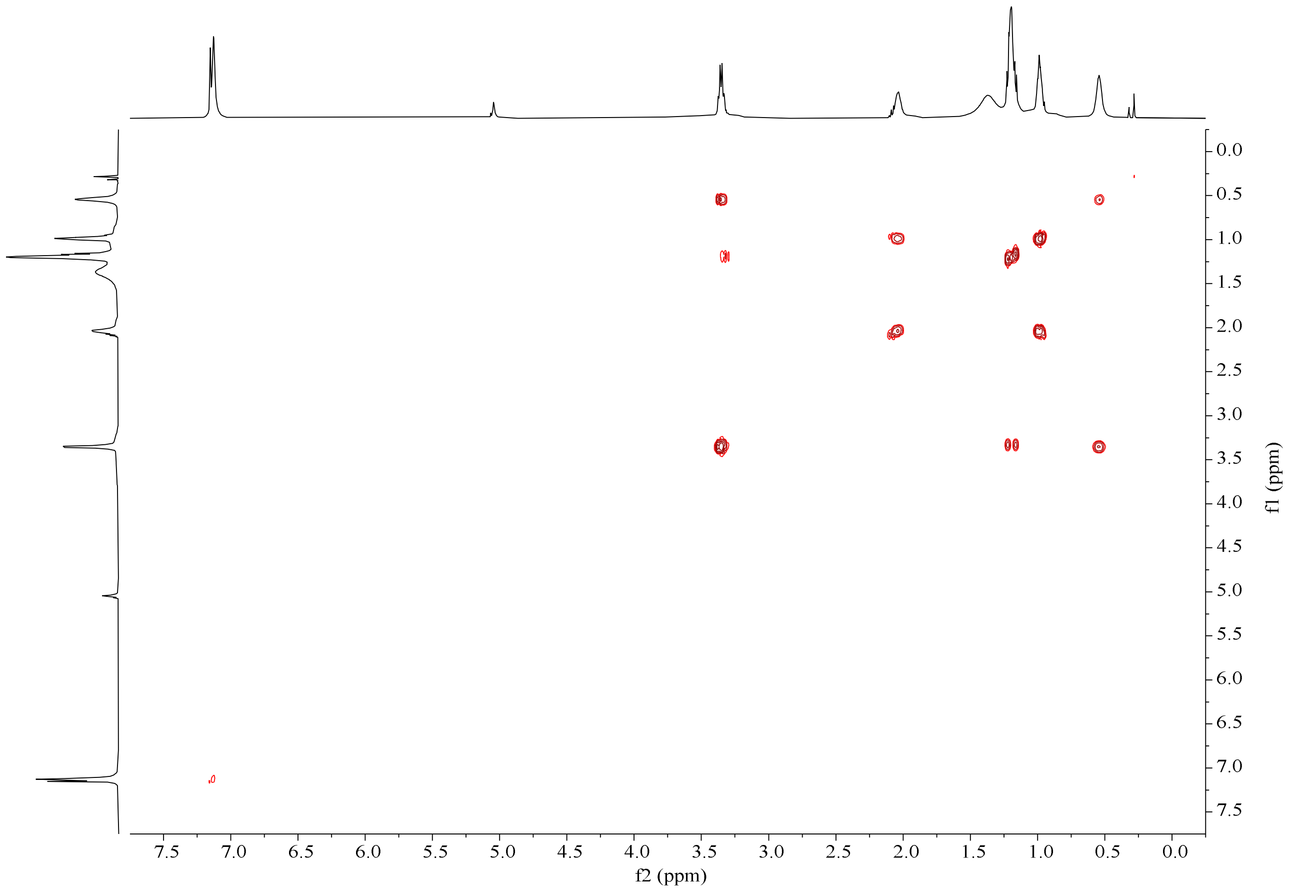

Figure S4. ^{1}H-^{1}H COSY NMR spectrum of isolated [(^{EtDip}nacnac)Mg(OEt_2)I].

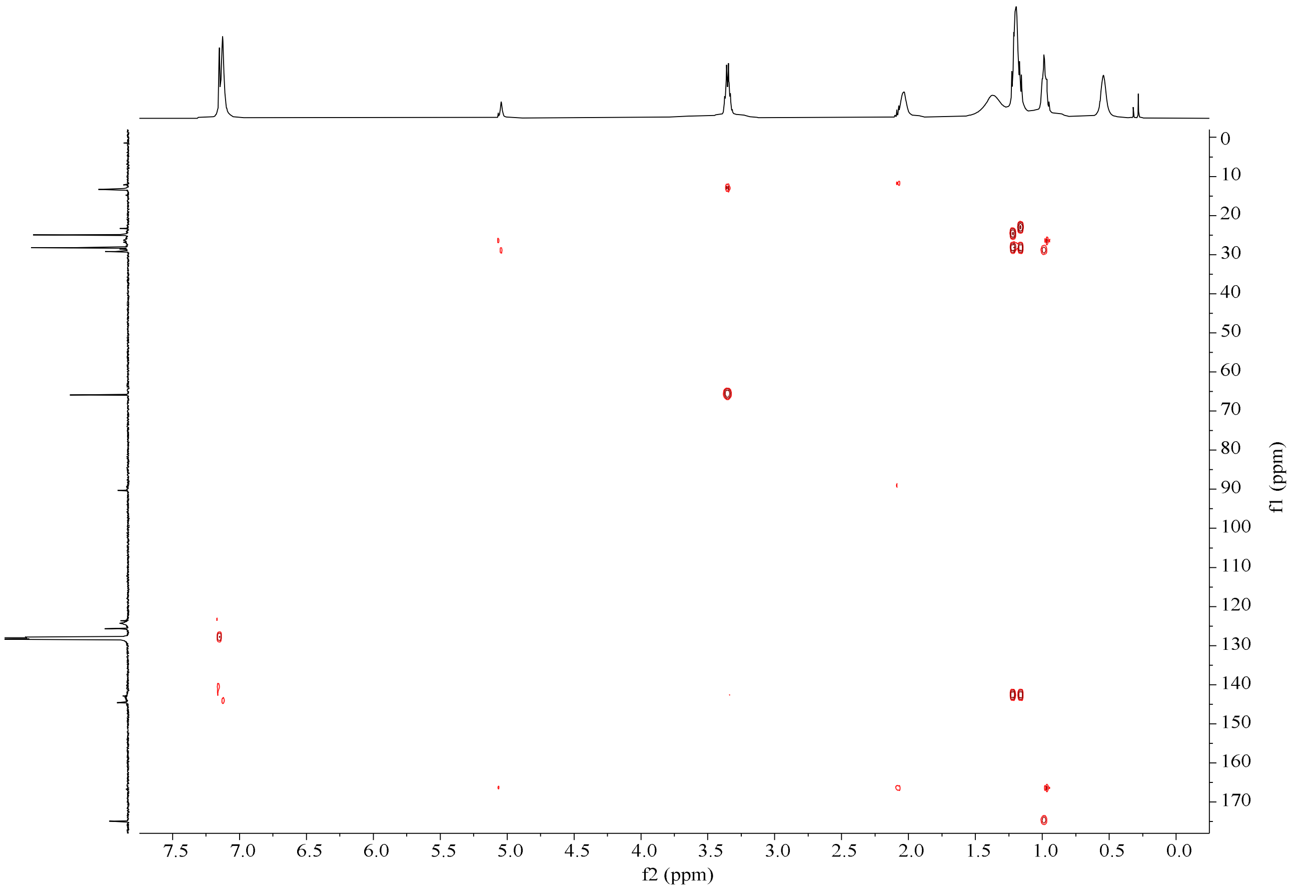

Figure S5. ^{1}H-^{13}C HMBC NMR spectrum of isolated [(^{EtDip}nacnac)Mg(OEt_2)I].

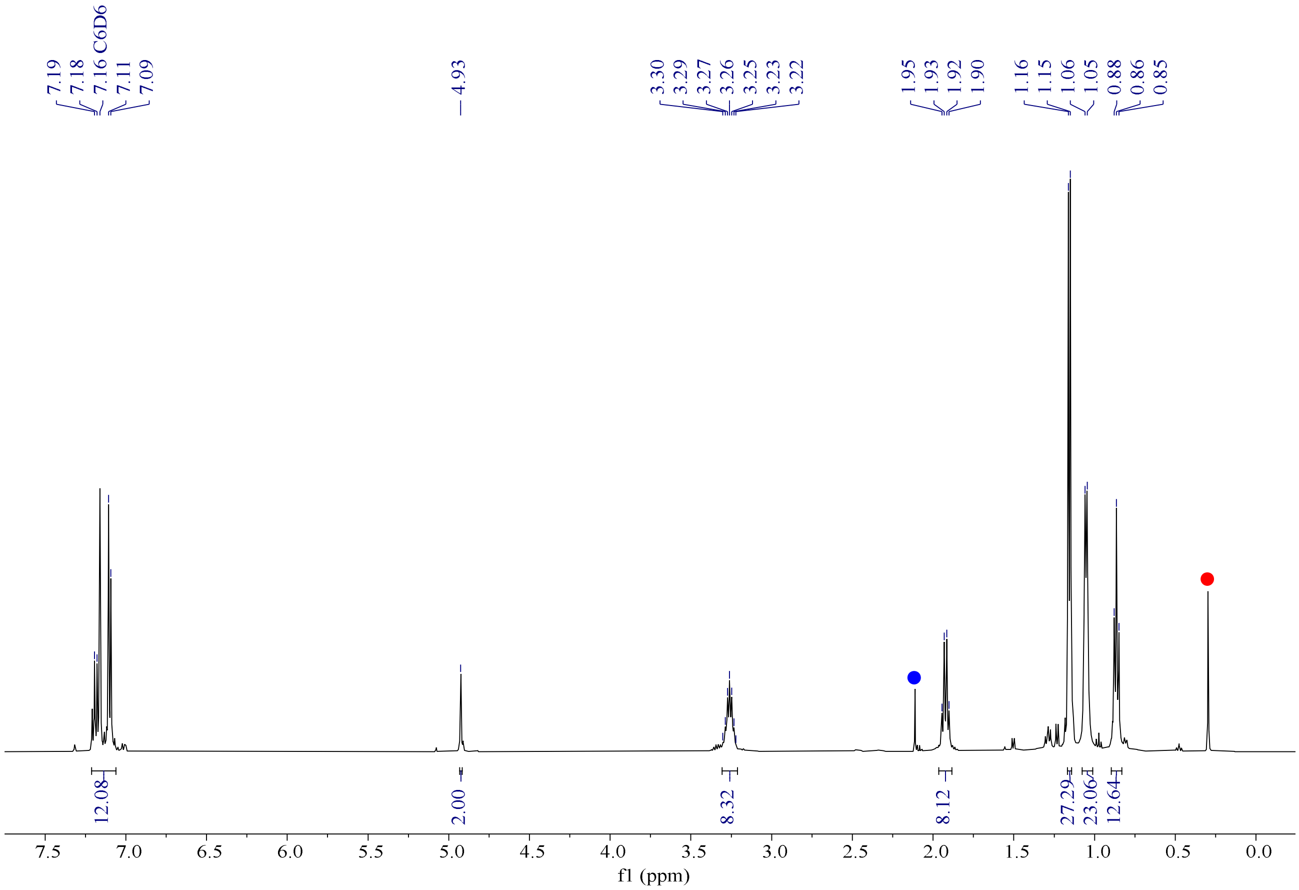

Figure S6. ^{1}H NMR spectrum (499.9 MHz, C_6D_6, 298 K) of isolated [{(^{EtDip}nacnac)Mg(μ-I)}$_2$]. The red circle denotes the resonance associated with silicone grease. The blue circle denotes the resonance associated with toluene (Ph-CH_3).

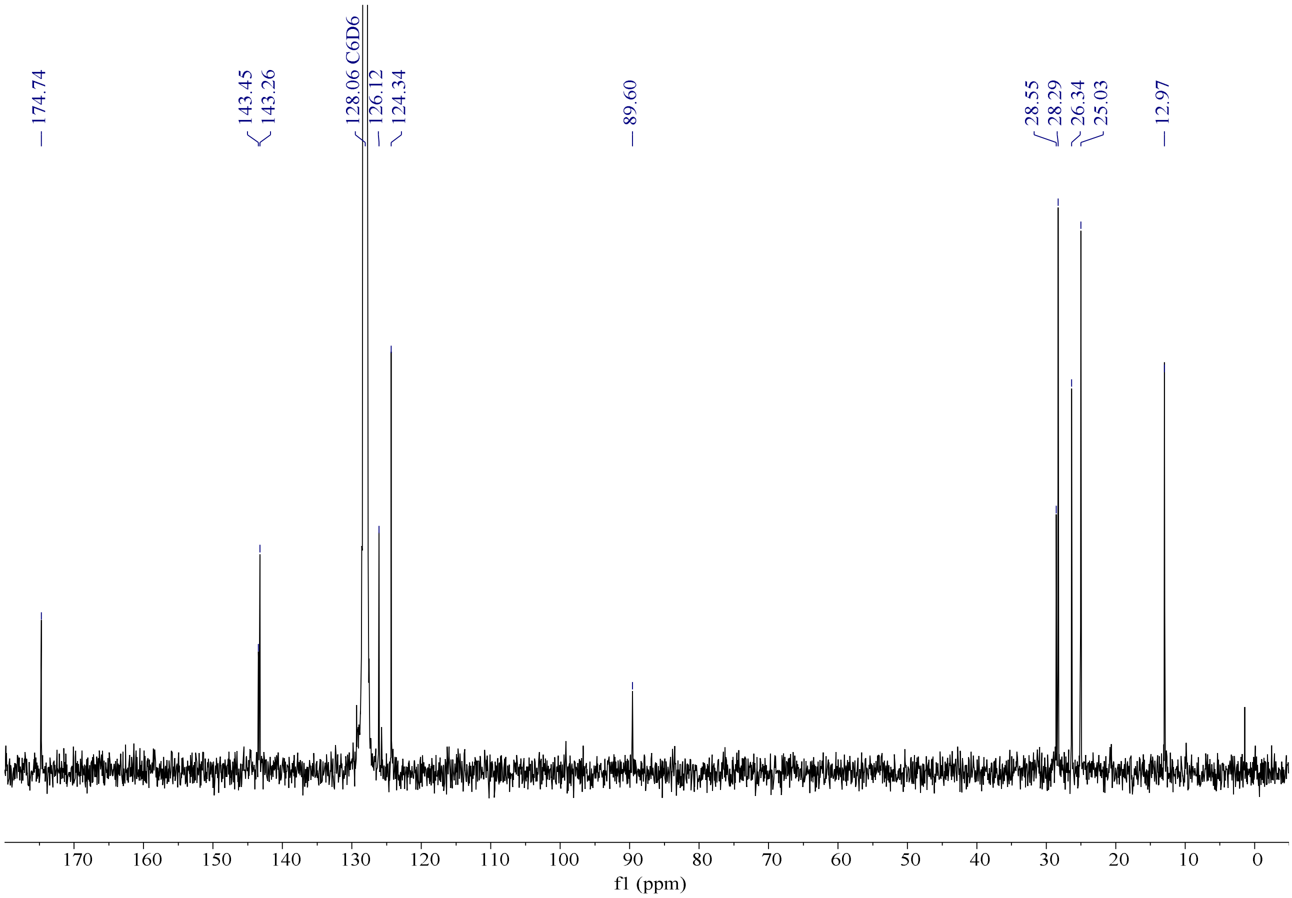

Figure S7. $^{13}C\{^1H\}$ NMR spectrum (125.7 MHz, C_6D_6, 298 K) of isolated $[\{(^{EtDip}nacnac)Mg(\mu\text{-}I)\}_2]$.

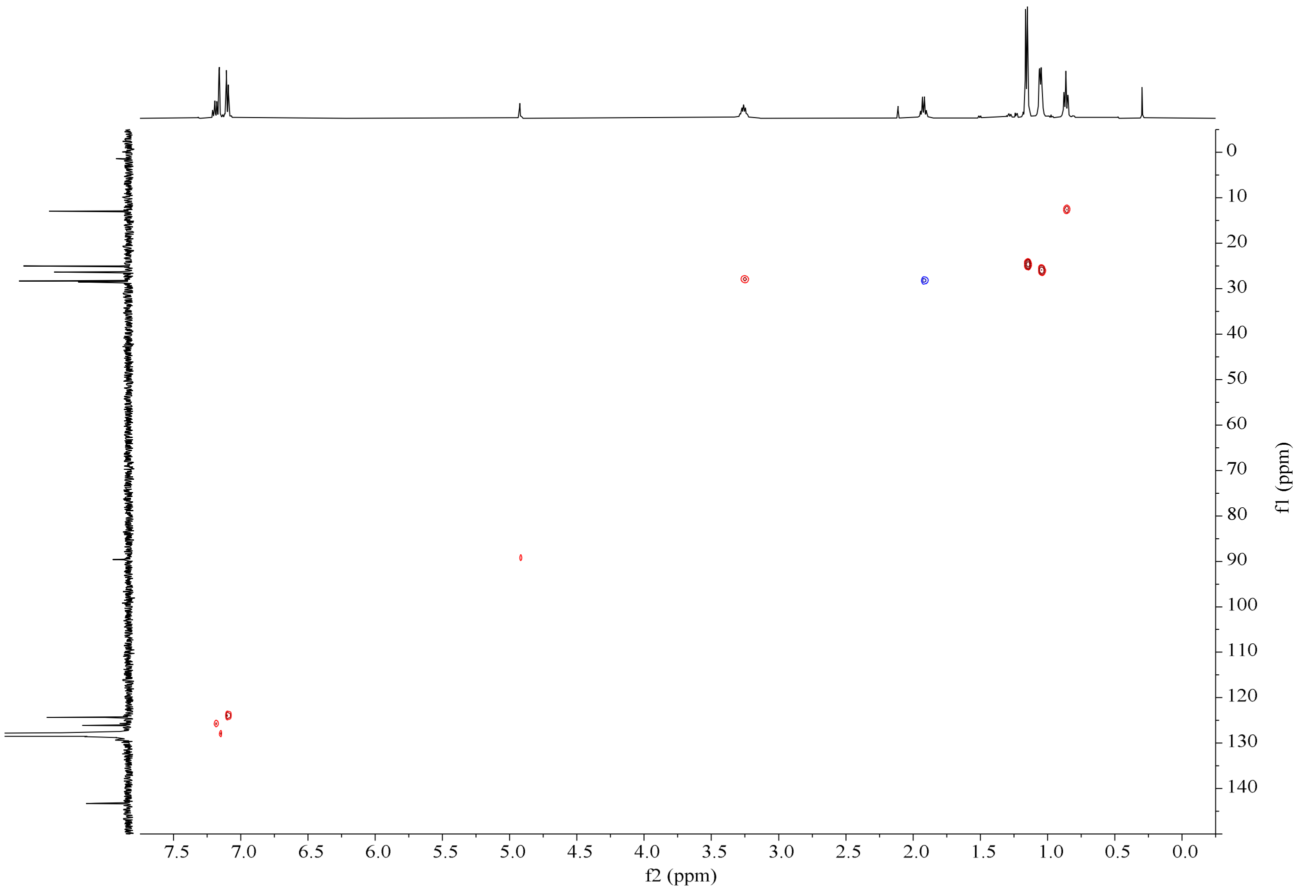

Figure S8. ^{1}H-^{13}C HSQC NMR spectrum of isolated [{(^{EtDip}nacnac)Mg(μ-I)}$_2$].

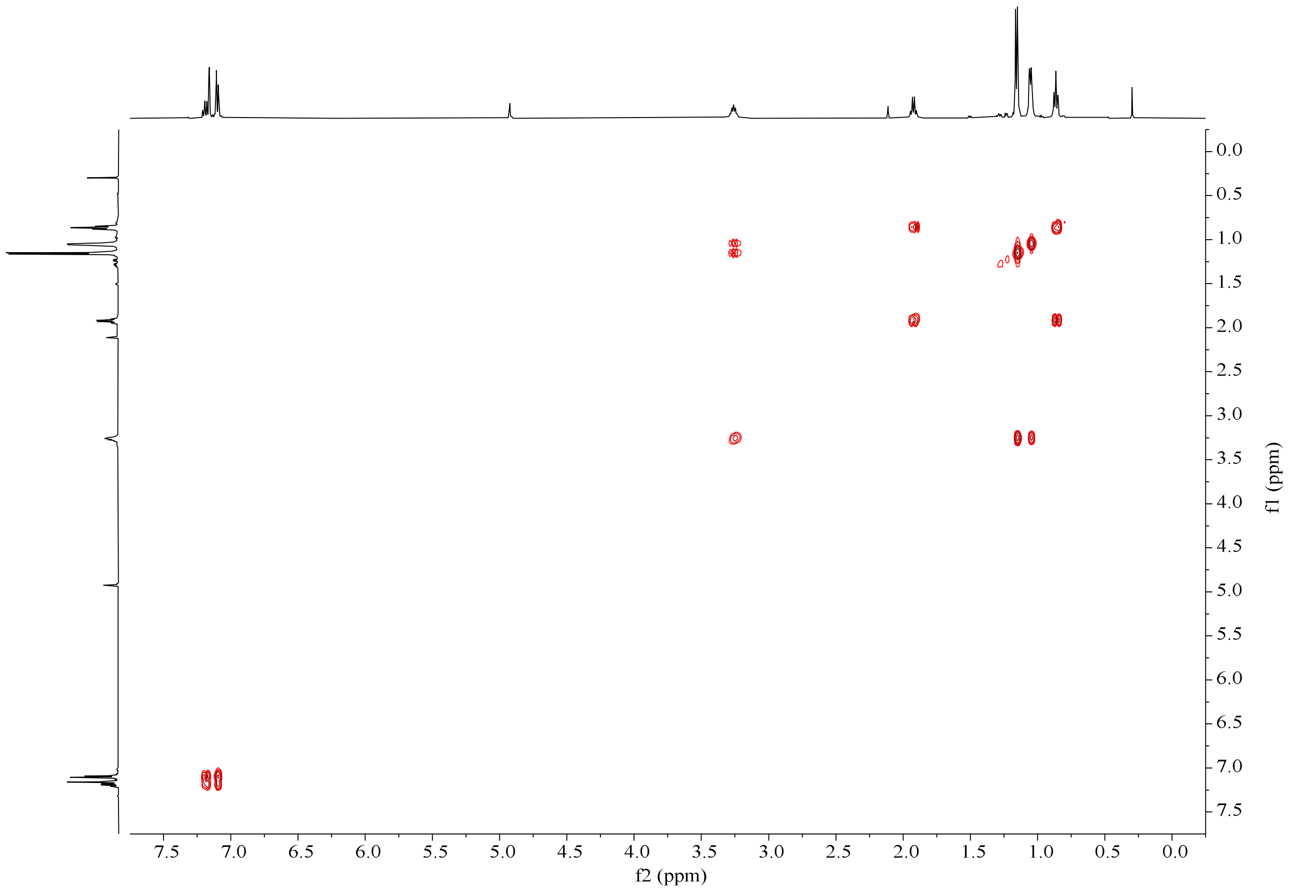

Figure S9. ^1H-^1H COSY NMR spectrum of isolated [{(^{EtDip}nacnac)Mg(μ-I)}$_2$].

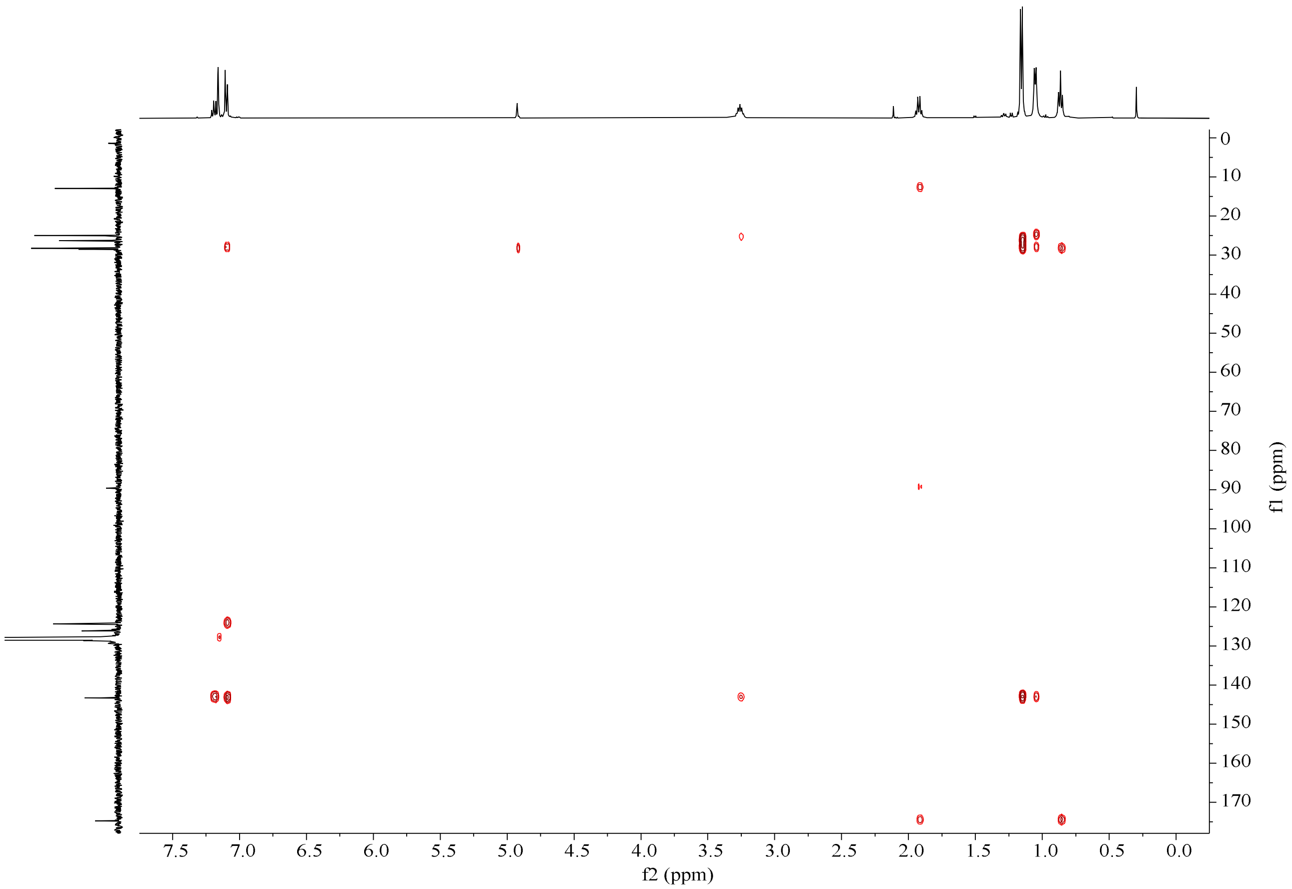

Figure S10. ^{1}H-^{13}C HMBC NMR spectrum of isolated [{(^{EtDip}nacnac)Mg(μ-I)}$_2$].

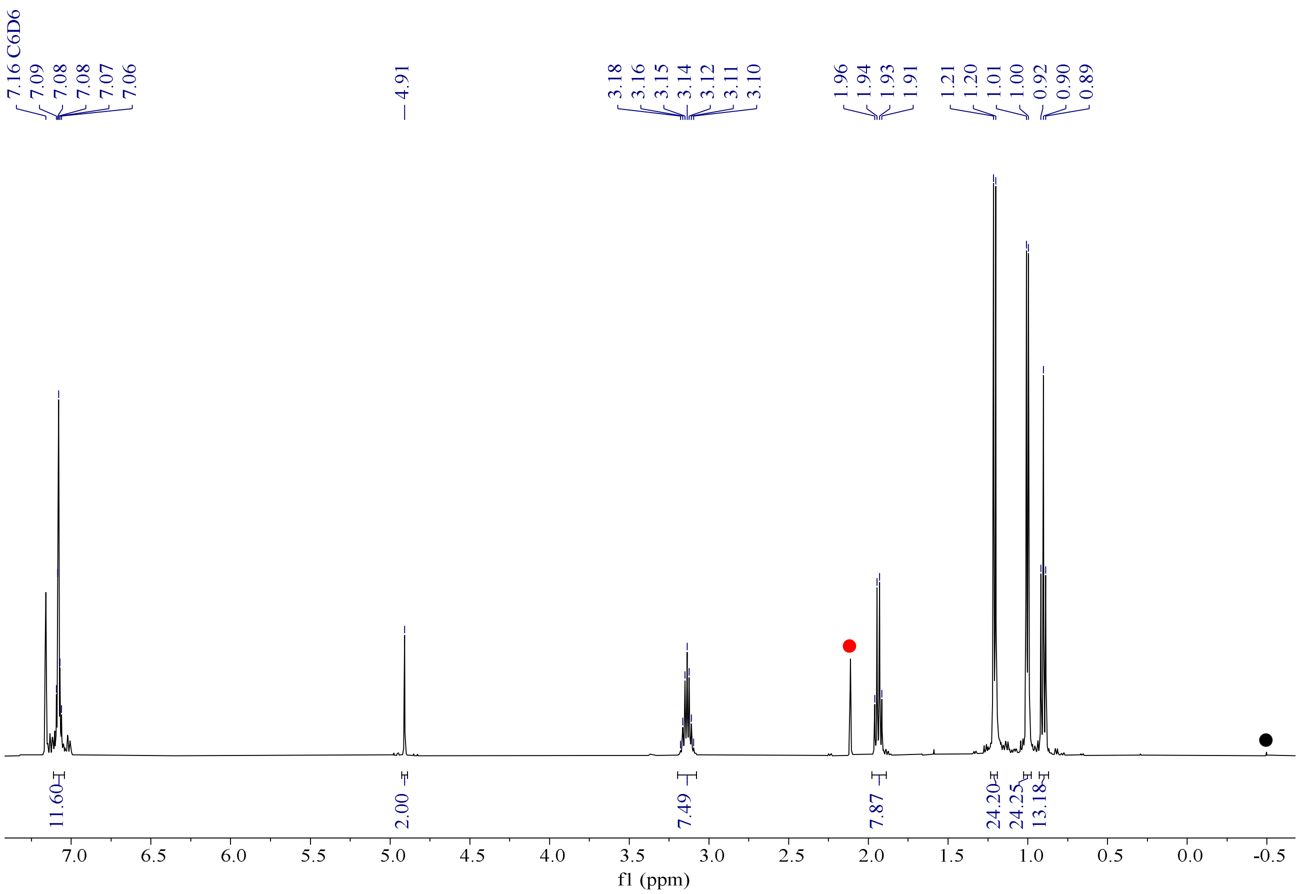

Figure S11. ^{1}H NMR spectrum (499.9 MHz, C_6D_6, 298 K) of isolated [{(^{EtDip}nacnac)Mg}$_2$] **2b**. The red circle denotes the resonance associated with toluene (Ph-CH_3). During the preparation and isolation of [{(^{EtDip}nacnac)Mg}$_2$] **2b**, a very small amount (ca. 3%) of [{(^{EtDip}nacnac)Mg(μ-OH)}$_2$] **3b** (^{1}H NMR resonance of Mg(μ-OH)$_2$: δ −0.50 ppm) was formed likely from moisture/air. The black circle denotes the resonance associated with Mg(μ-OH)$_2$ of [{(^{EtDip}nacnac)Mg(μ-OH)}$_2$] **3b**.

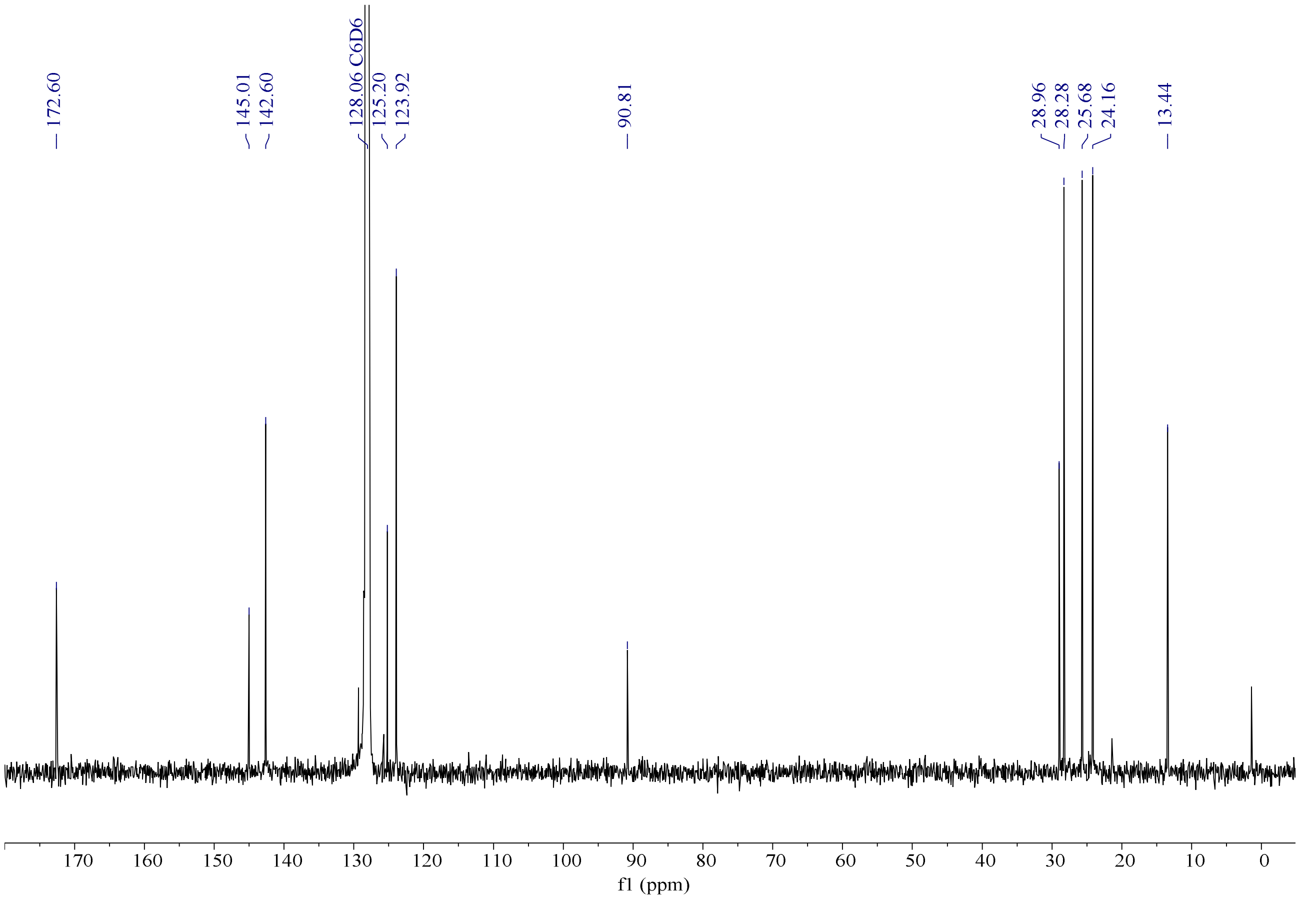

Figure S12. $^{13}C\{^{1}H\}$ NMR spectrum (125.7 MHz, C_6D_6, 298 K) of isolated [{(^{EtDip}nacnac)Mg}$_2$] **2b**.

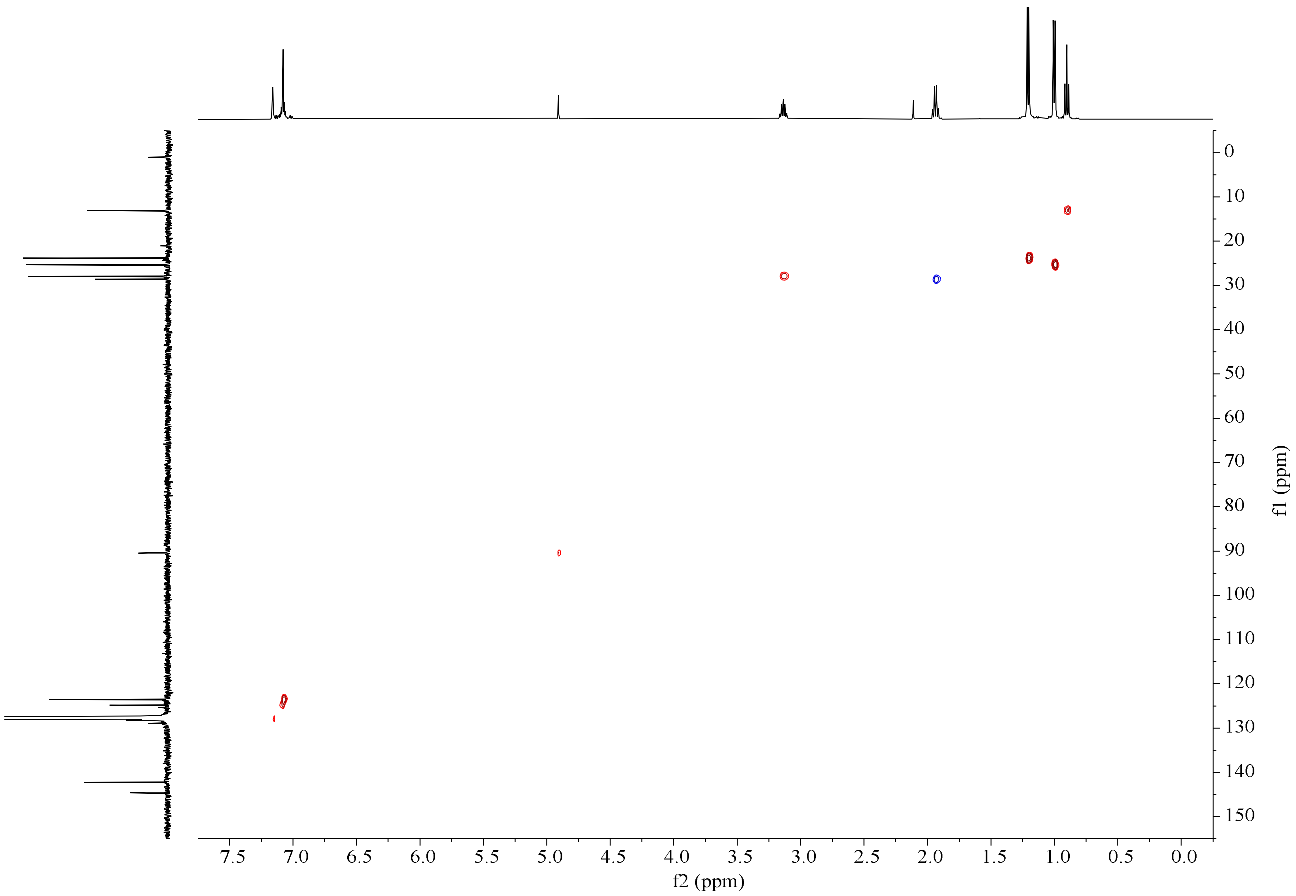

Figure S13. ^{1}H-^{13}C HSQC NMR spectrum of isolated [{(^{EtDip}nacnac)Mg}$_2$] **2b**.

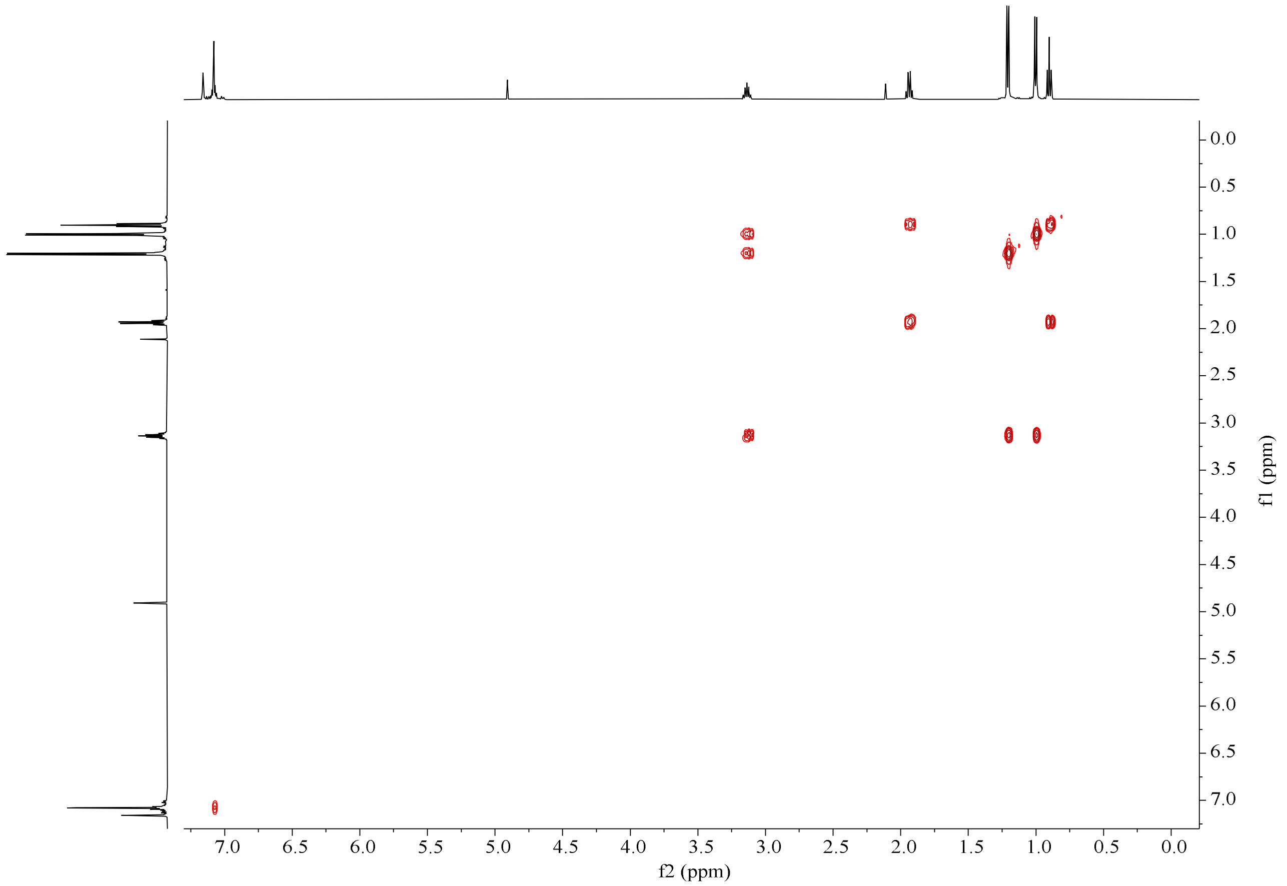

Figure S14. ^{1}H-^{1}H COSY NMR spectrum of isolated [{(^{EtDip}nacnac)Mg}$_2$] **2b**.

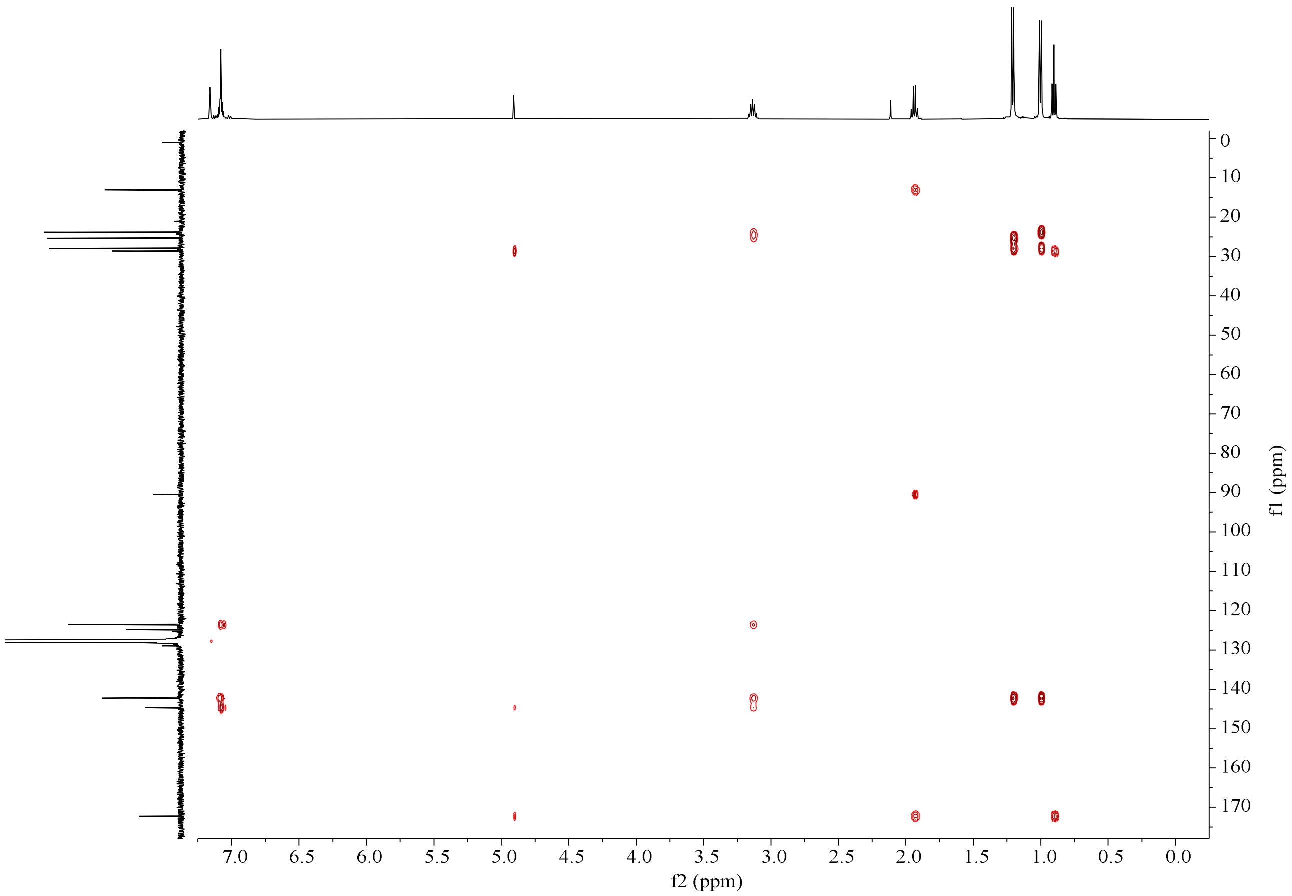

Figure S15. ^{1}H-^{13}C HMBC NMR spectrum of isolated [{(^{EtDip}nacnac)Mg}$_2$] **2b**.

2.3 Synthesis of [{(^{EtDip}nacnac)Mg(μ-H)}$_2$] 7b and precursors

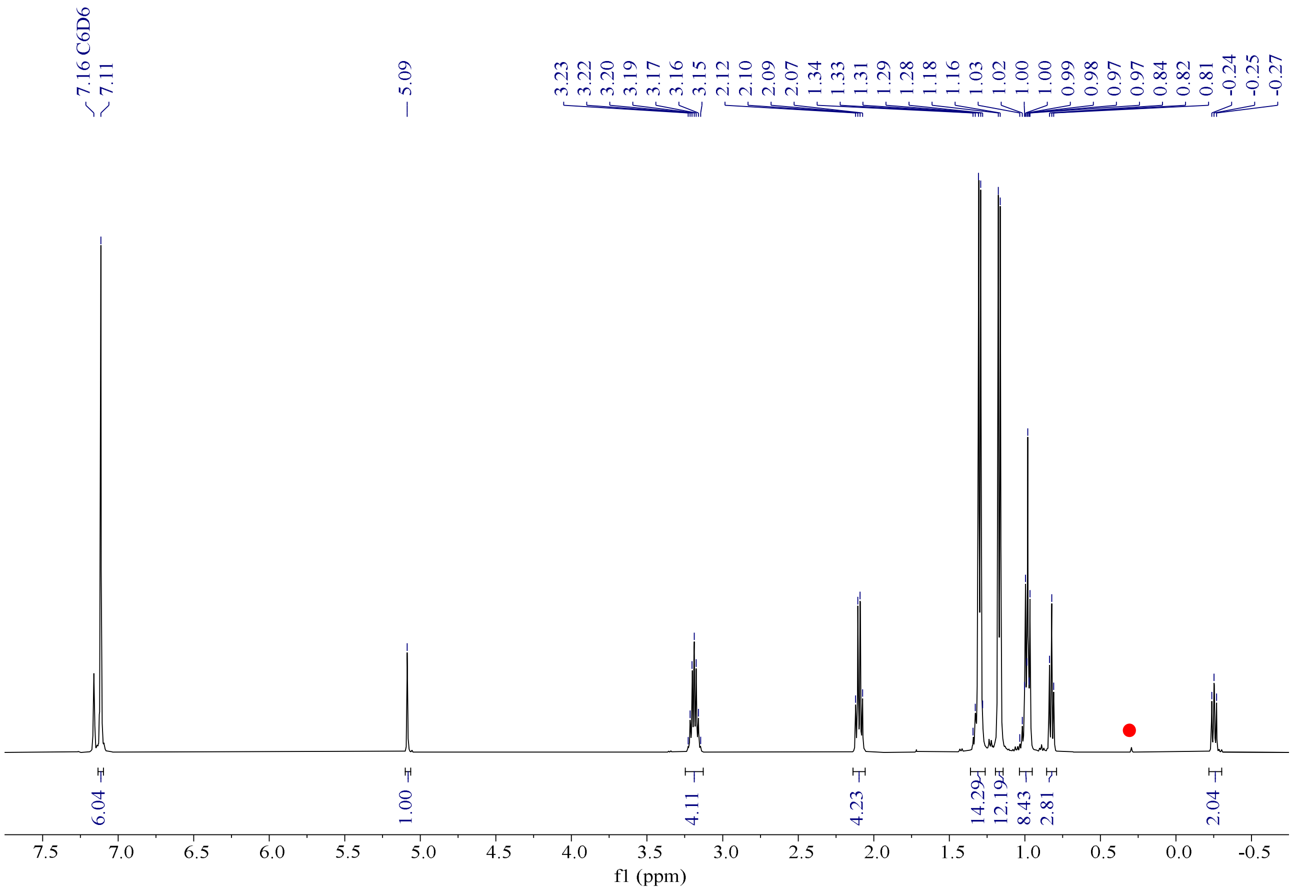

Figure S16. ^{1}H NMR spectrum (500.1 MHz, C_6D_6, 295 K) of isolated [(^{EtDip}nacnac)Mg(*n*Bu)] **9b**. The red circle denotes the resonance associated with silicone grease.

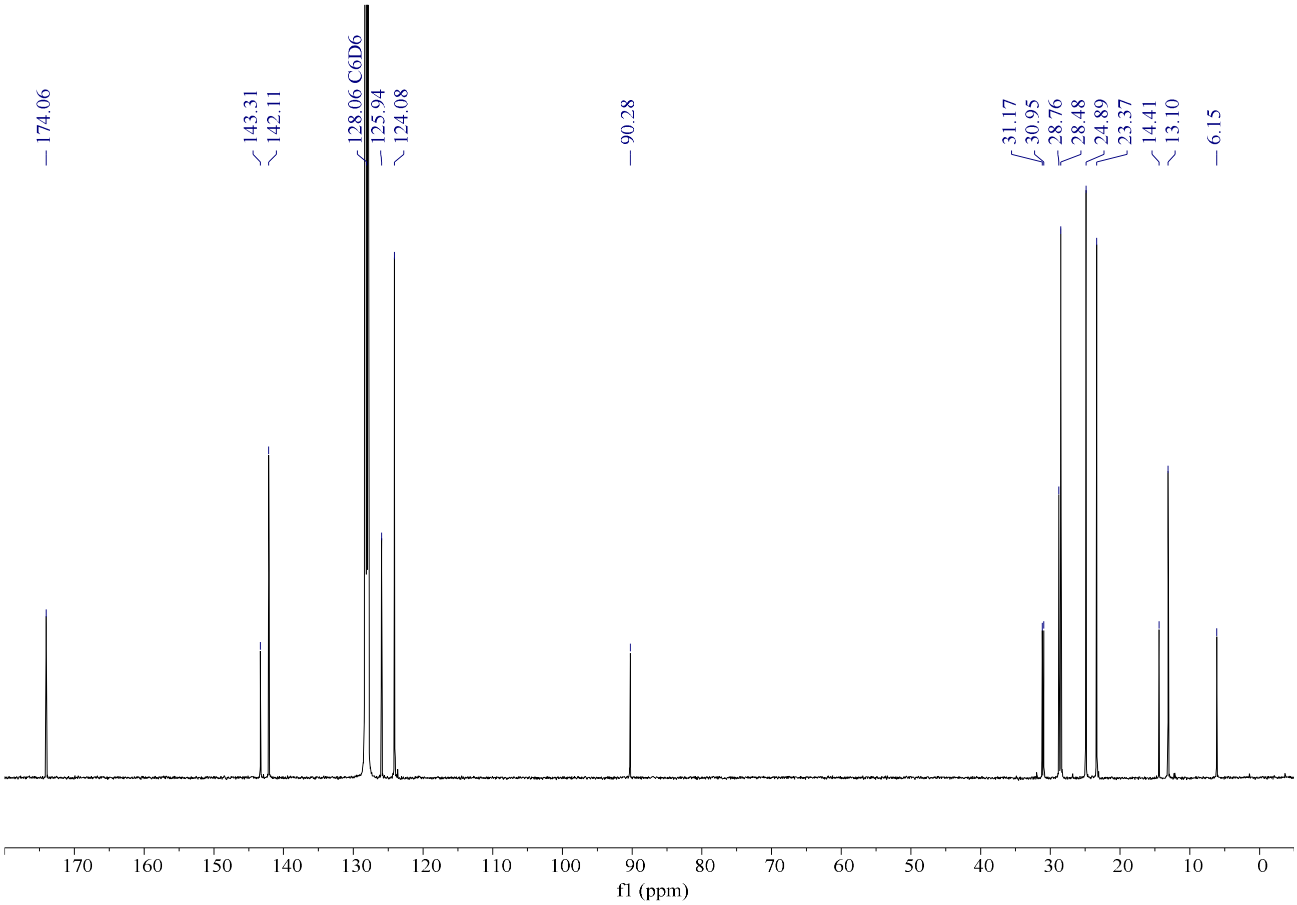

Figure S17. $^{13}C\{^{1}H\}$ NMR spectrum (125.8 MHz, C_6D_6, 295 K) of isolated [(^{EtDip}nacnac)Mg*n*Bu] **9b**.

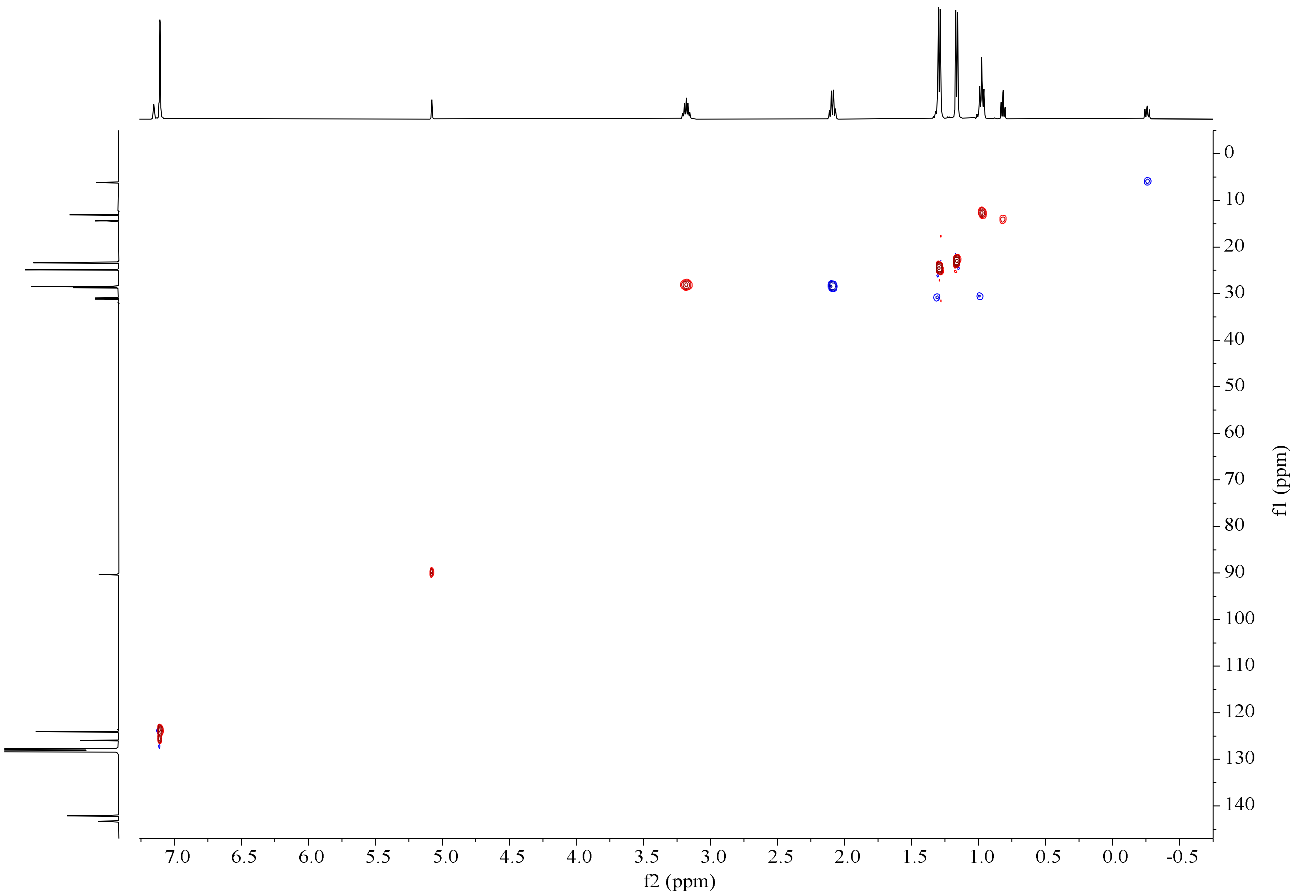

Figure S18. ^{1}H-^{13}C HSQC NMR spectrum of isolated [(^{EtDip}nacnac)Mg*n*Bu] **9b**.

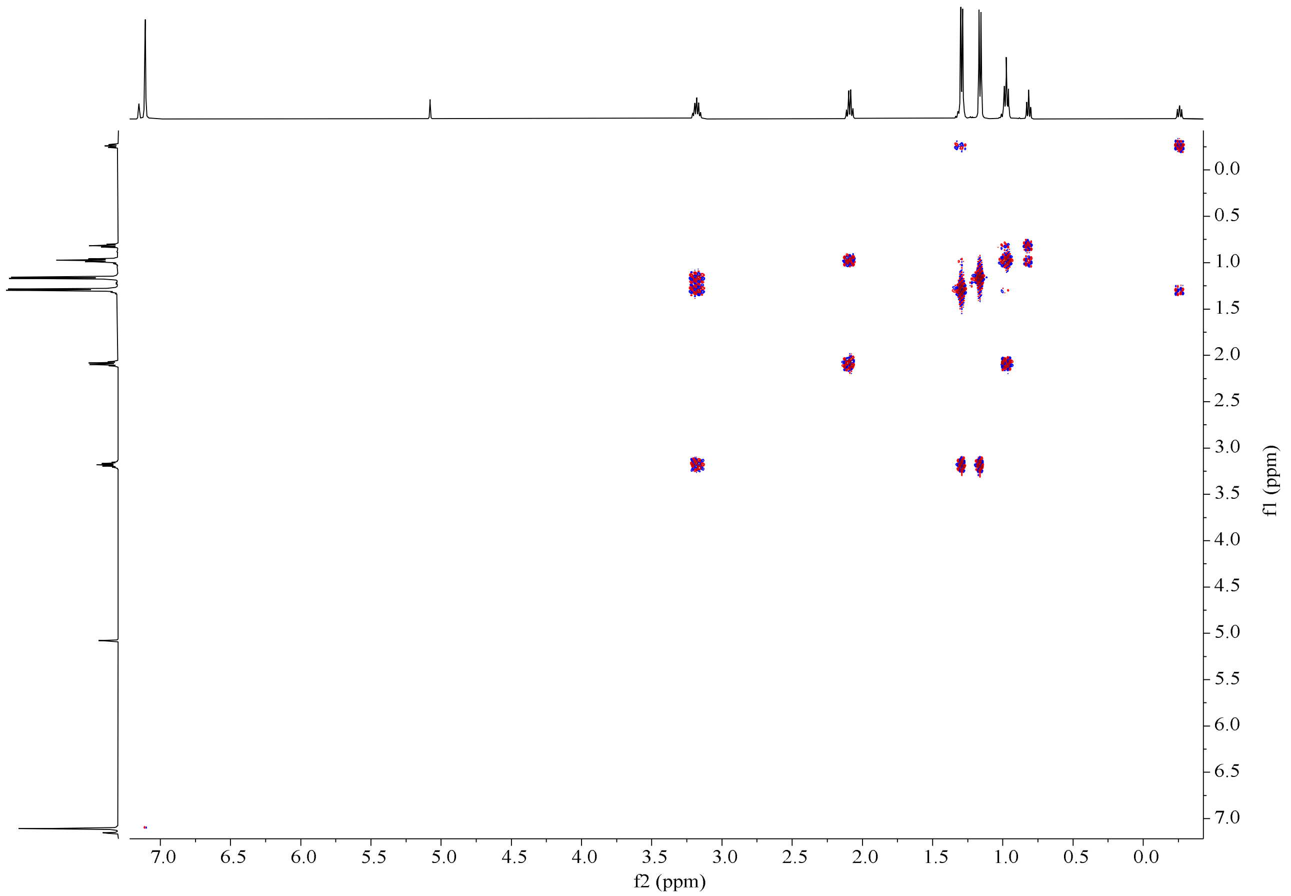

Figure S19. ^{1}H-^{1}H COSY NMR spectrum of isolated [(^{EtDip}nacnac)Mg*n*Bu] **9b**.

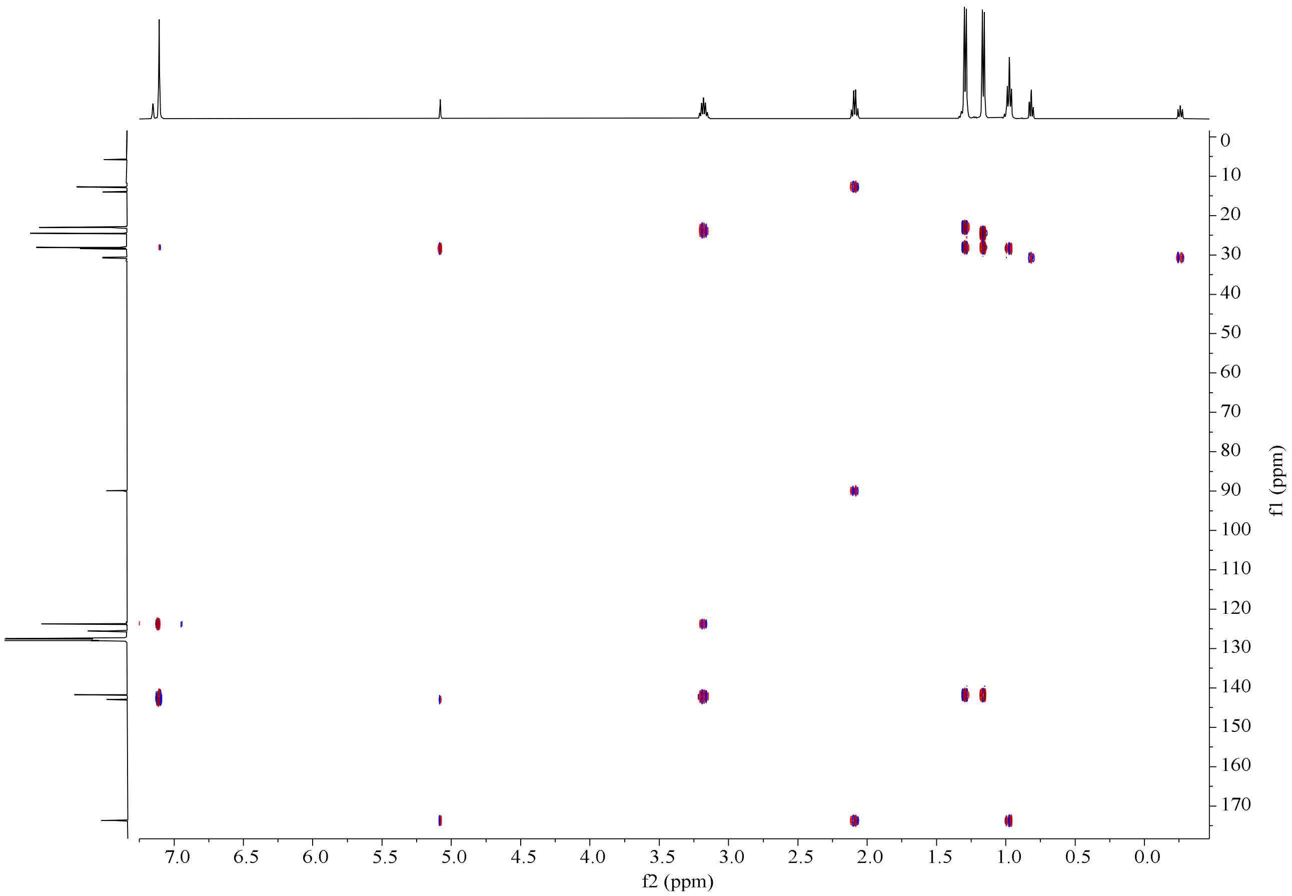

Figure S20. ^1H-^{13}C HMBC NMR spectrum of isolated [(^{EtDip}nacnac)Mg*n*Bu] **9b**.

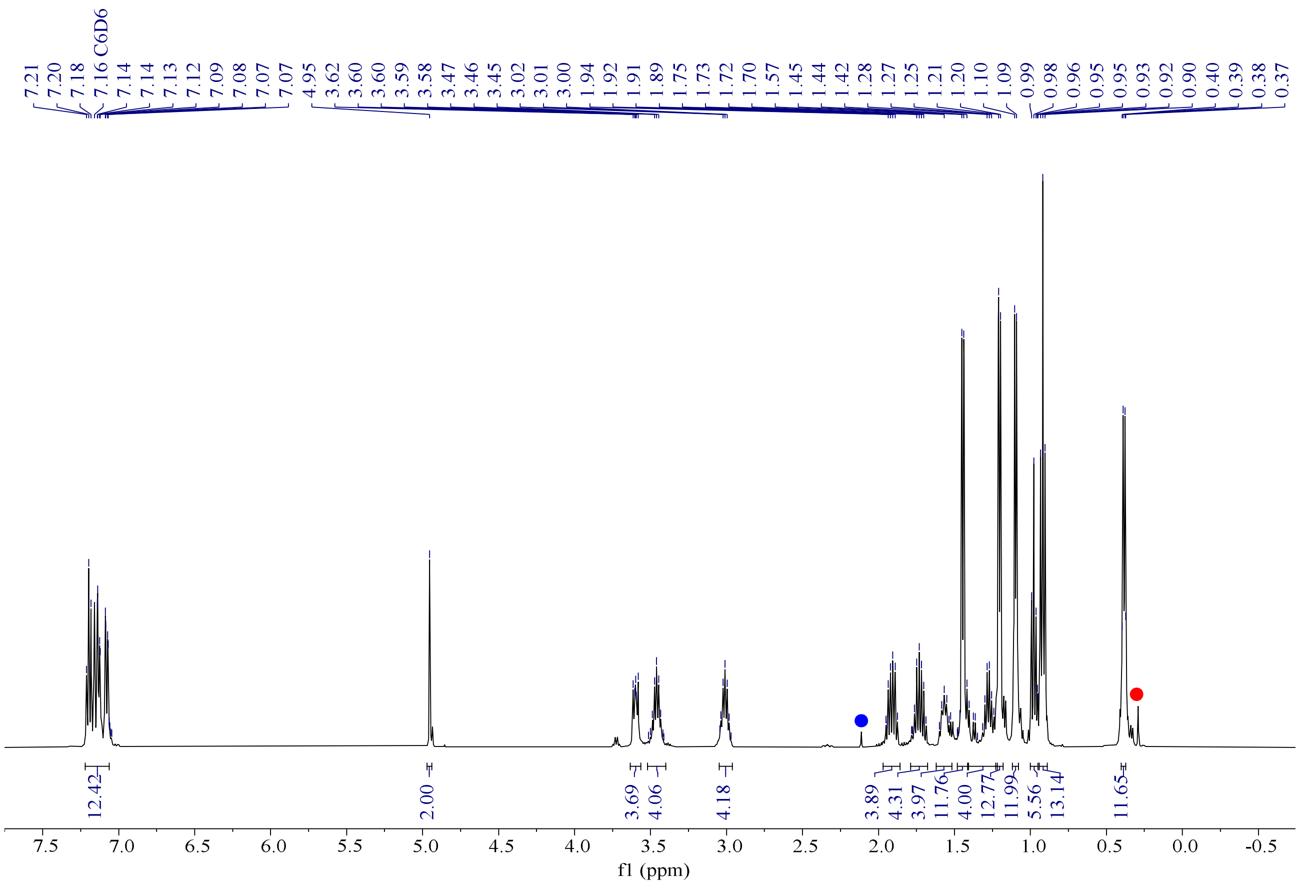

Figure S21. ^{1}H NMR spectrum (500.1 MHz, C_6D_6, 298 K) of isolated [(^{EtDip}nacnac)Mg(μ-O*n*Bu)}$_2$]. The red circle denotes the resonance associated with silicone grease. The blue circle denotes the resonance associated with toluene (Ph-CH_3).

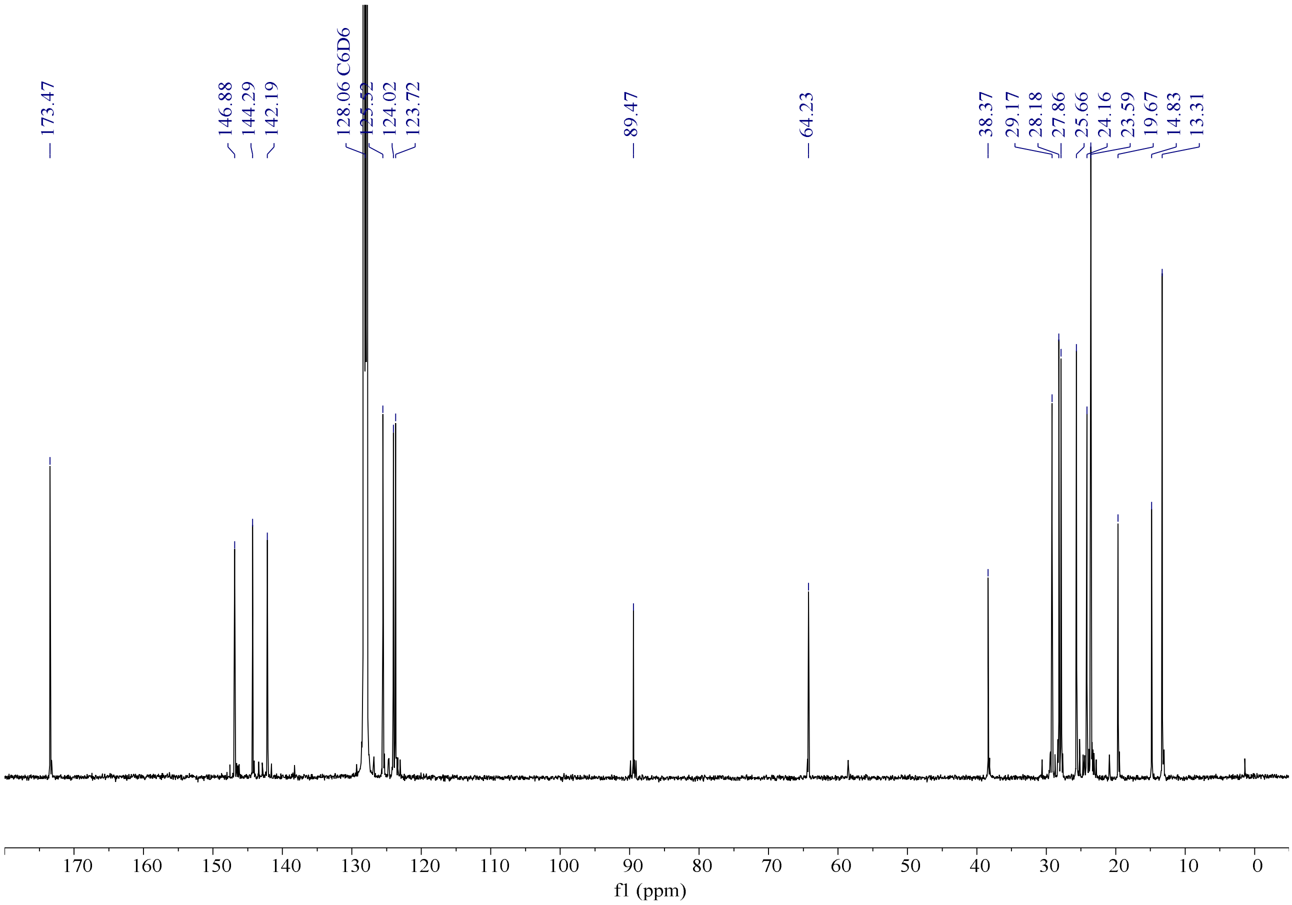

Figure S22. $^{13}C\{^{1}H\}$ NMR spectrum (125.8 MHz, C_6D_6, 298 K) of isolated $[(^{EtDip}nacnac)Mg(\mu\text{-O}n\text{Bu})\}_2]$.

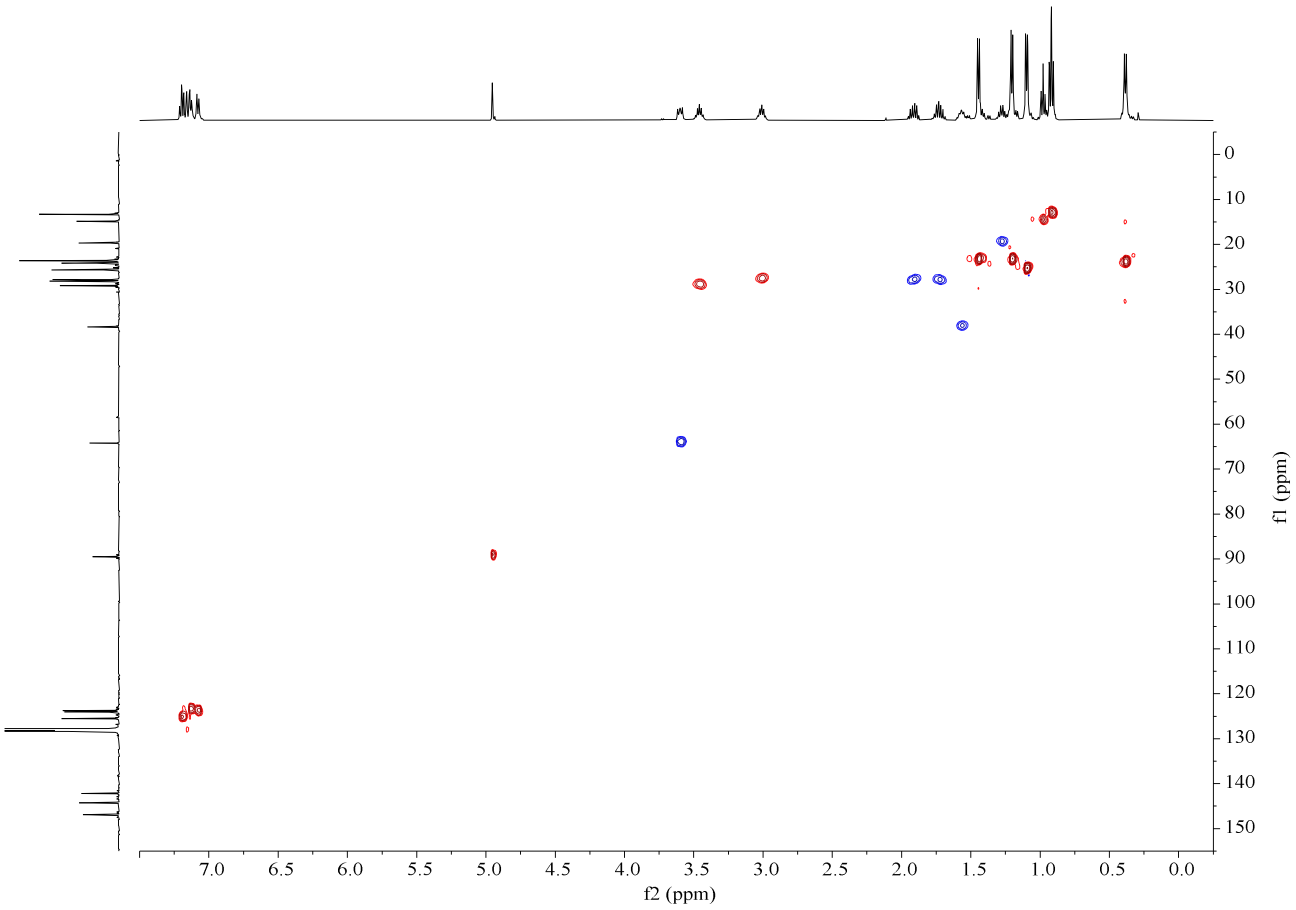

Figure S23. ^{1}H-^{13}C HSQC NMR spectrum of isolated [(^{EtDip}nacnac)Mg(μ-O*n*Bu)}$_2$].

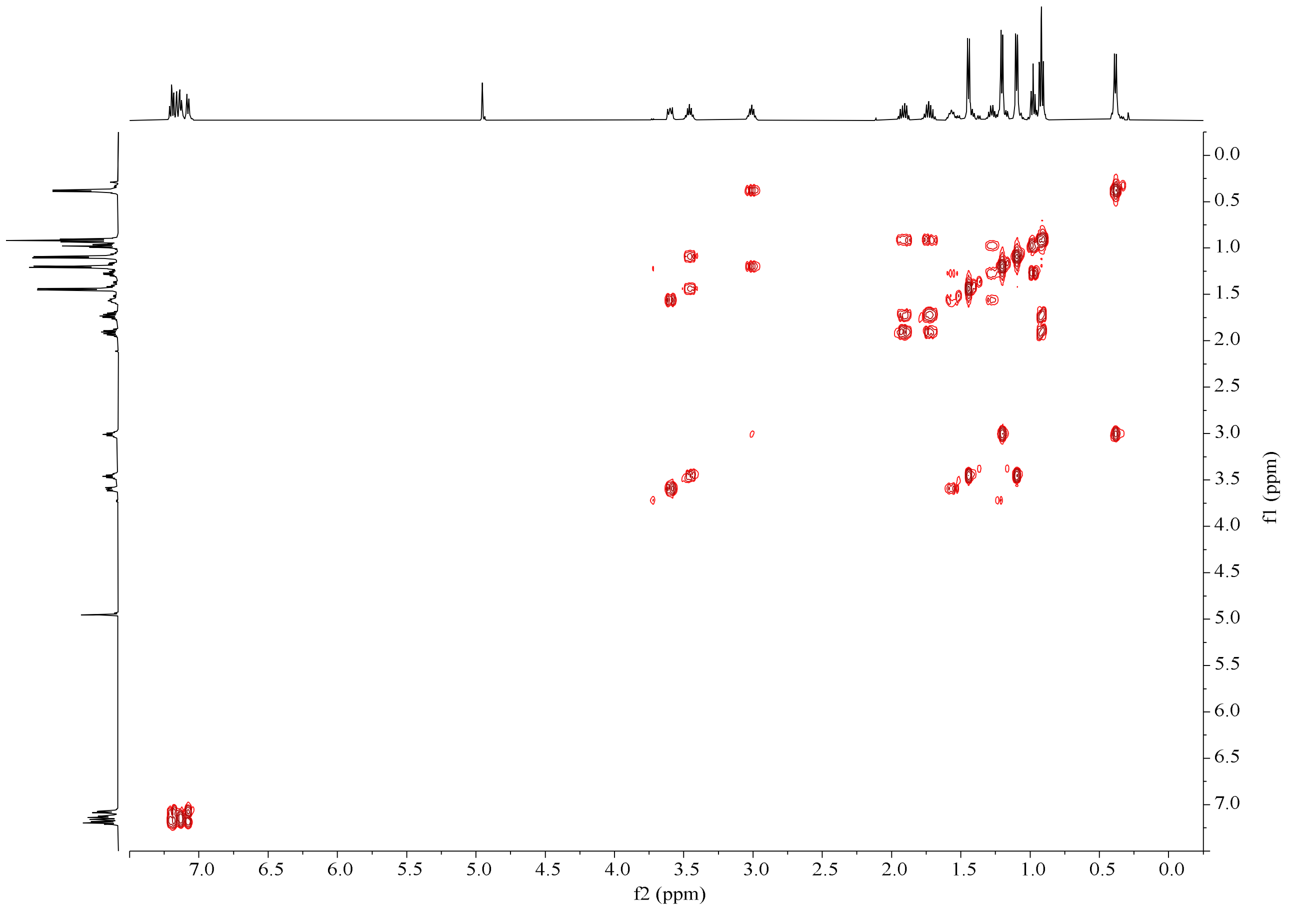

Figure S24. ^{1}H-^{1}H COSY NMR spectrum of isolated [(^{EtDip}nacnac)Mg(μ-O*n*Bu)}$_2$].

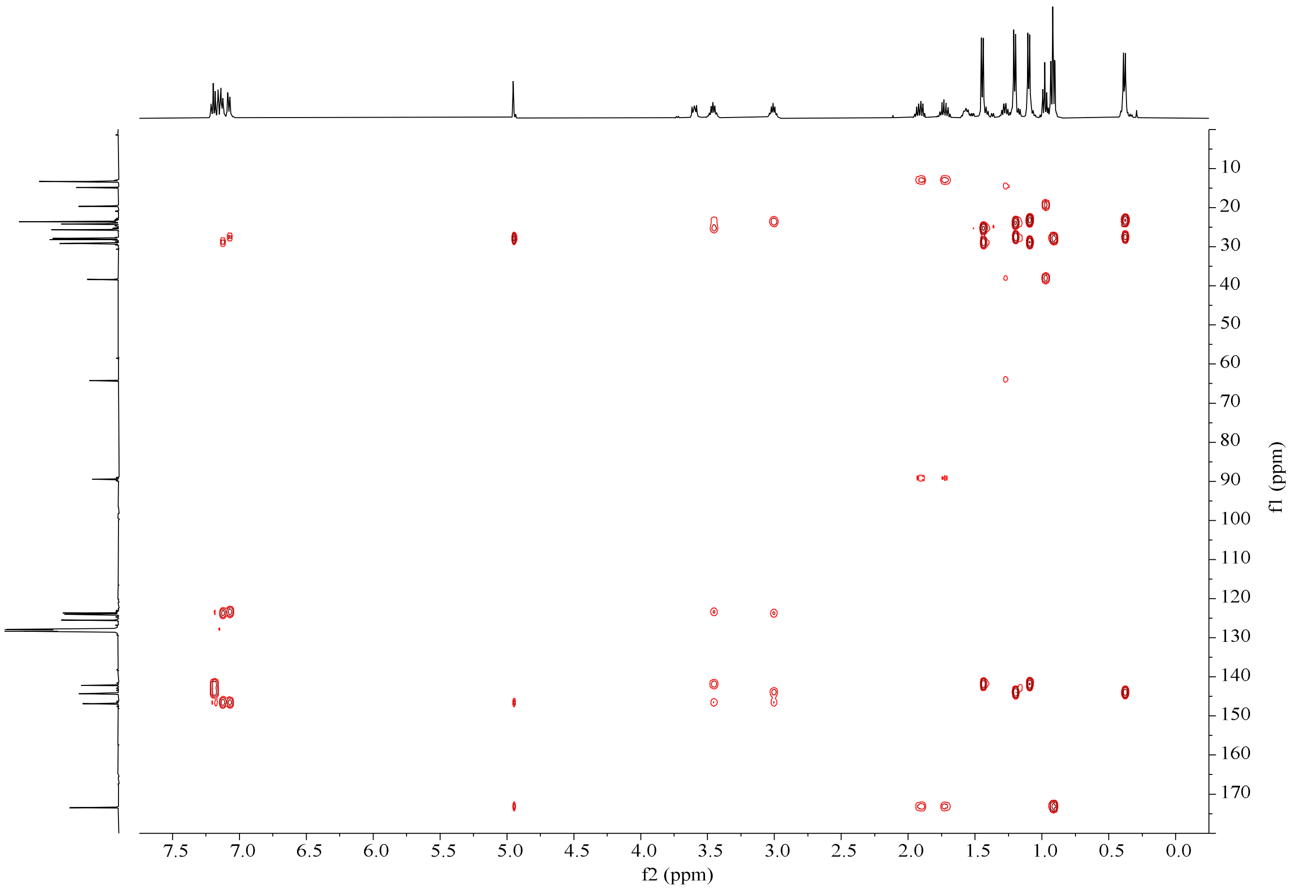

Figure S25. ^{1}H-^{13}C HMBC NMR spectrum of isolated [(^{EtDip}nacnac)Mg(μ-O*n*Bu)}$_2$].

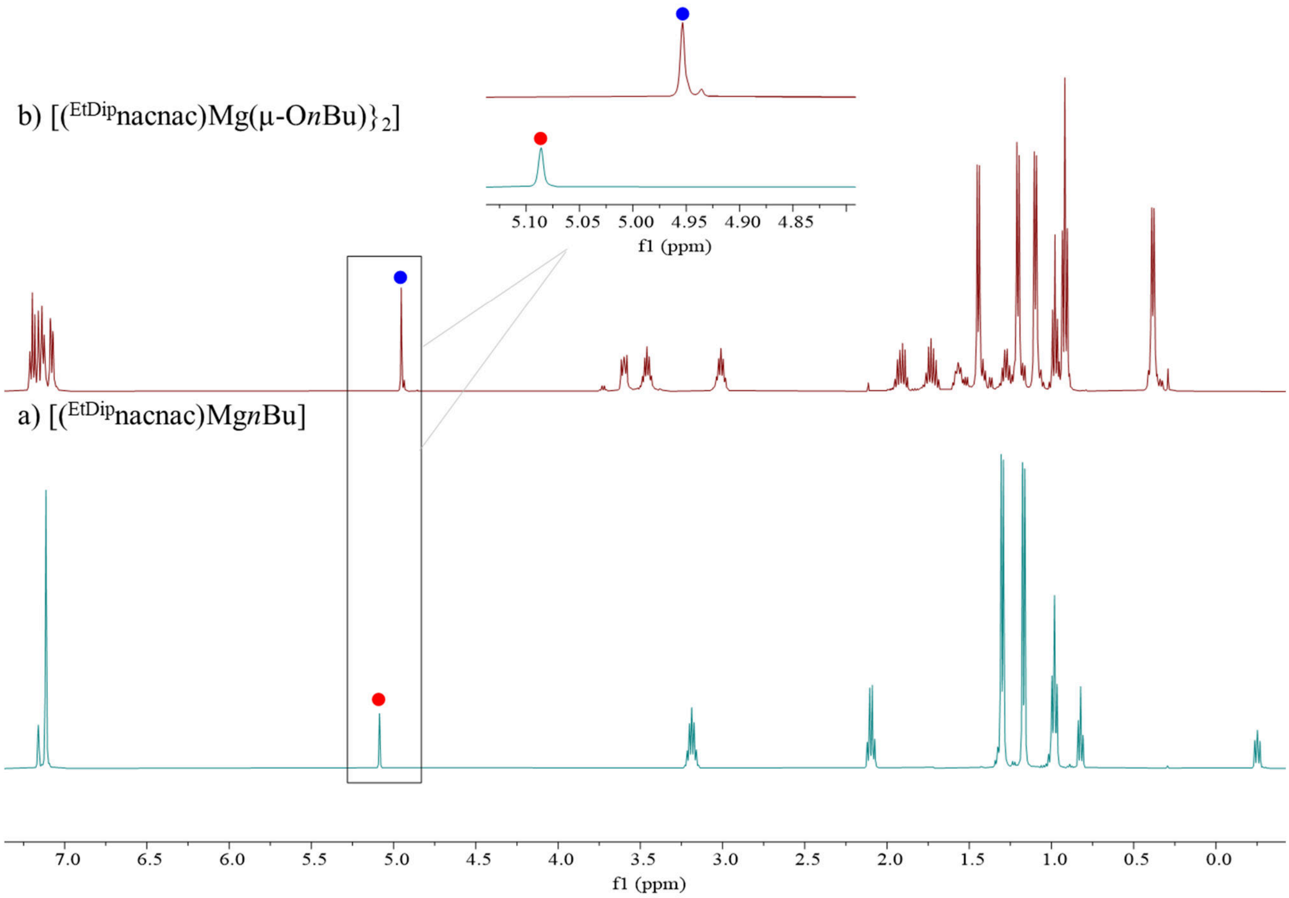

Figure S26. Stacked ^{1}H NMR spectra of isolated [(^{EtDip}nacnac)Mg*n*Bu] **9b** and isolated [(^{EtDip}nacnac)Mg(μ-O*n*Bu)}$_2$]. The red circle denotes the resonance associated with the backbone-C*H* of [(^{EtDip}nacnac)Mg*n*Bu] **9b**. The blue circle denotes the resonance associated with the backbone-C*H* of [(^{EtDip}nacnac)Mg(μ-O*n*Bu)}$_2$]. Spectrum a): 500.1 MHz, C_6D_6, 295 K. Spectrum b): 500.1 MHz, C_6D_6, 298 K.

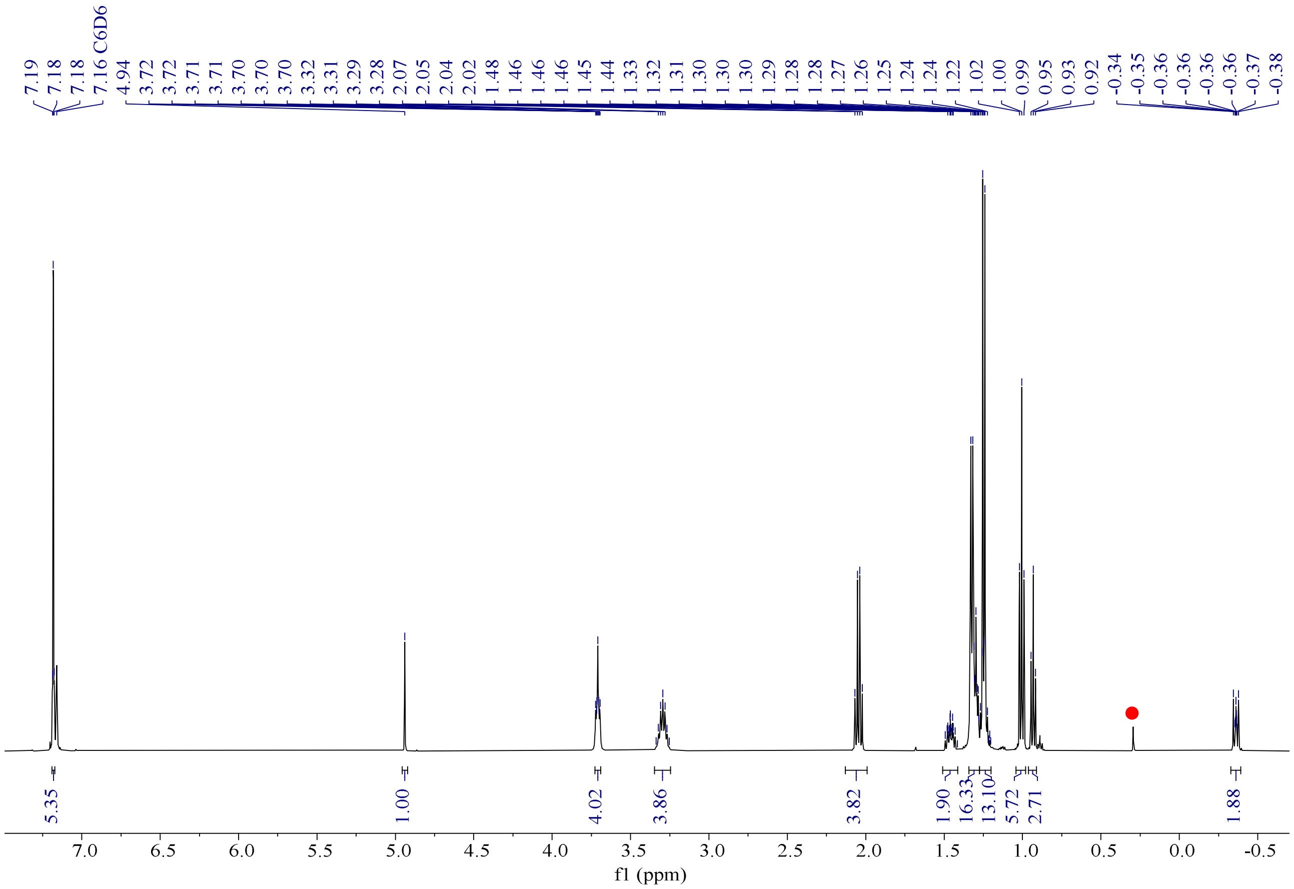

Figure S27. ^{1}H NMR spectrum (499.9 MHz, C_6D_6, 298 K) of isolated [(^{EtDip}nacnac)Mg(THF)*n*Bu]. The red circle denotes the resonance associated with silicone grease.

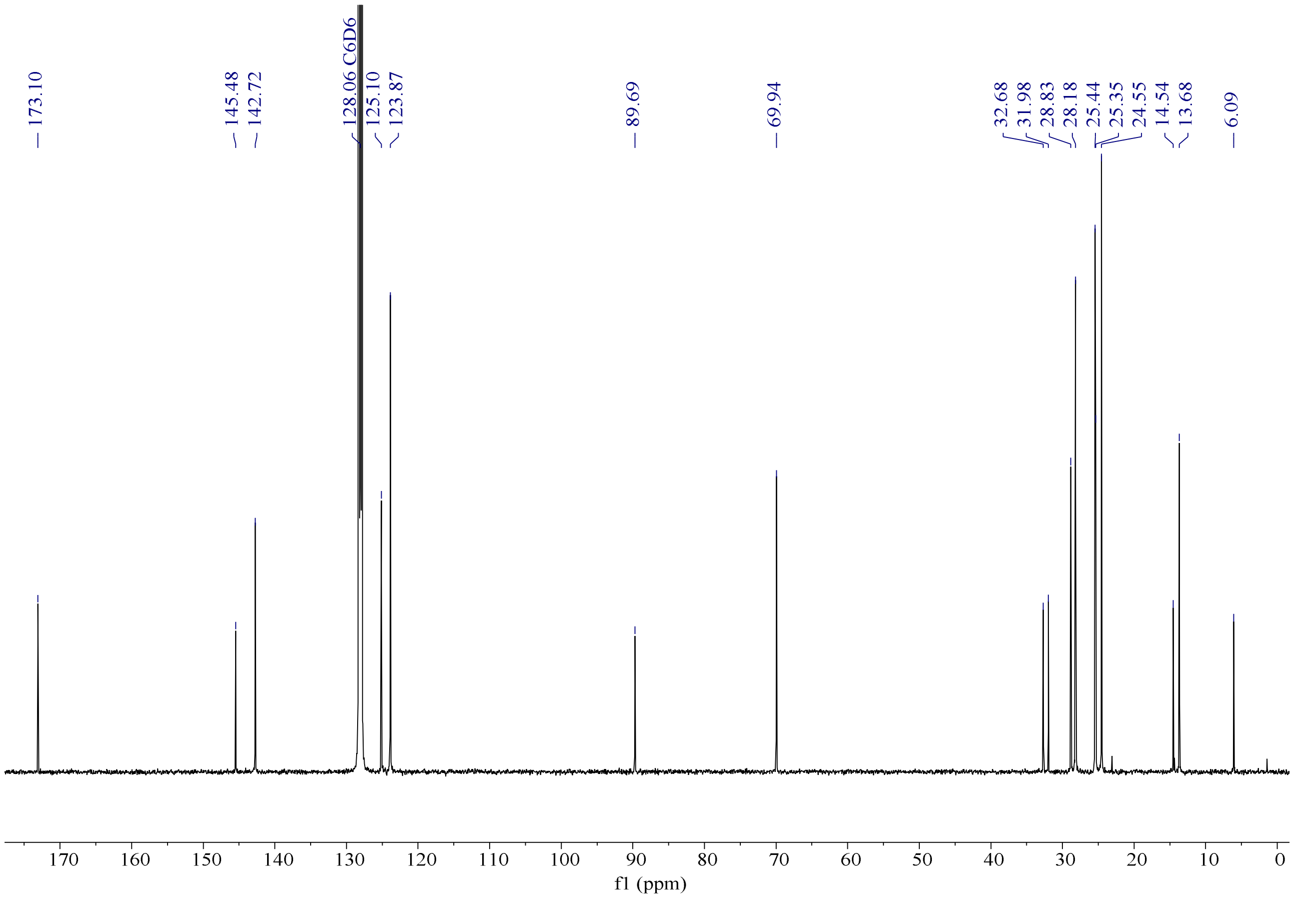

Figure S28. $^{13}C\{^{1}H\}$ NMR spectrum (125.7 MHz, C_6D_6, 298 K) of isolated [(^{EtDip}nacnac)Mg(THF)*n*Bu].

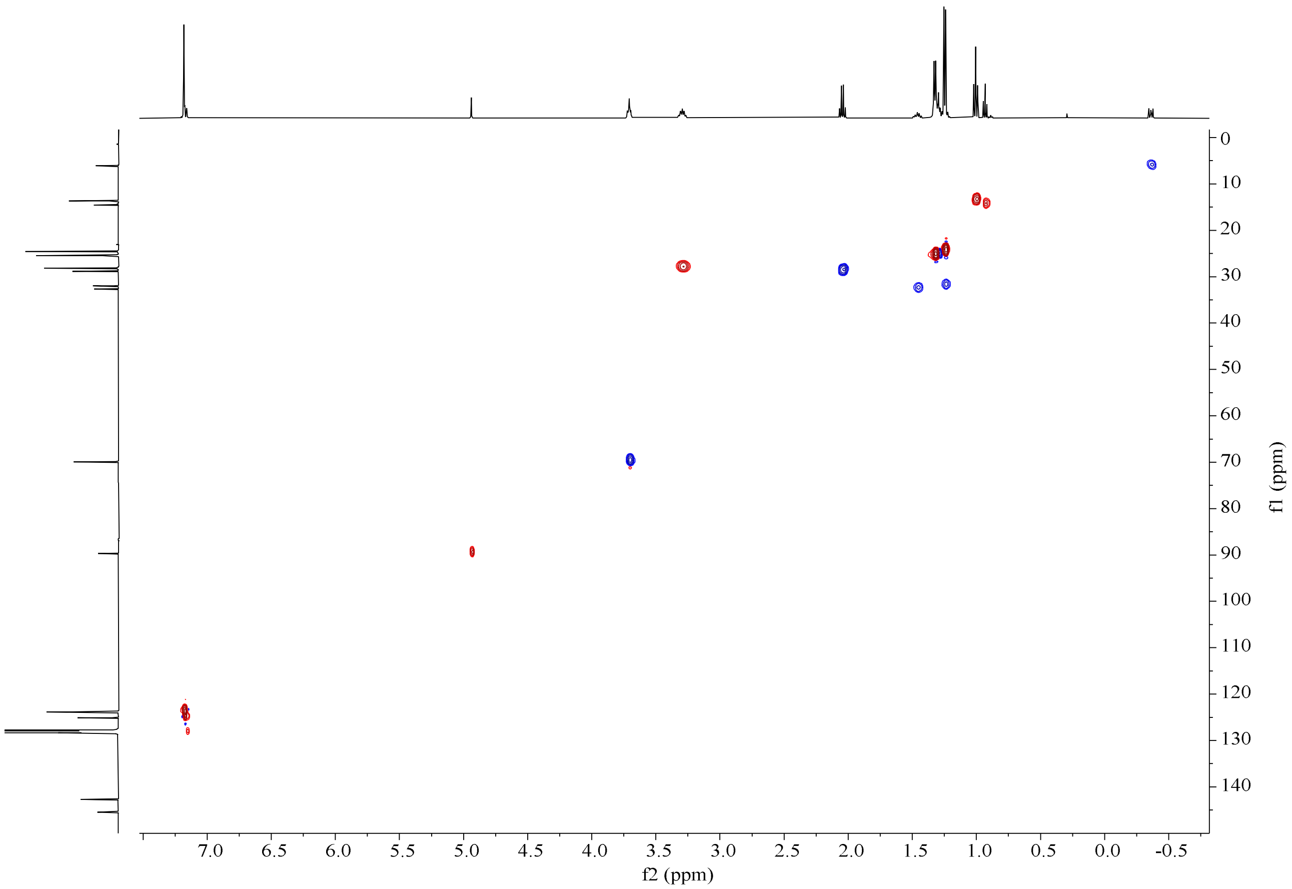

Figure S29. ^{1}H-^{13}C HSQC NMR spectrum of isolated [(^{EtDip}nacnac)Mg(THF)*n*Bu].

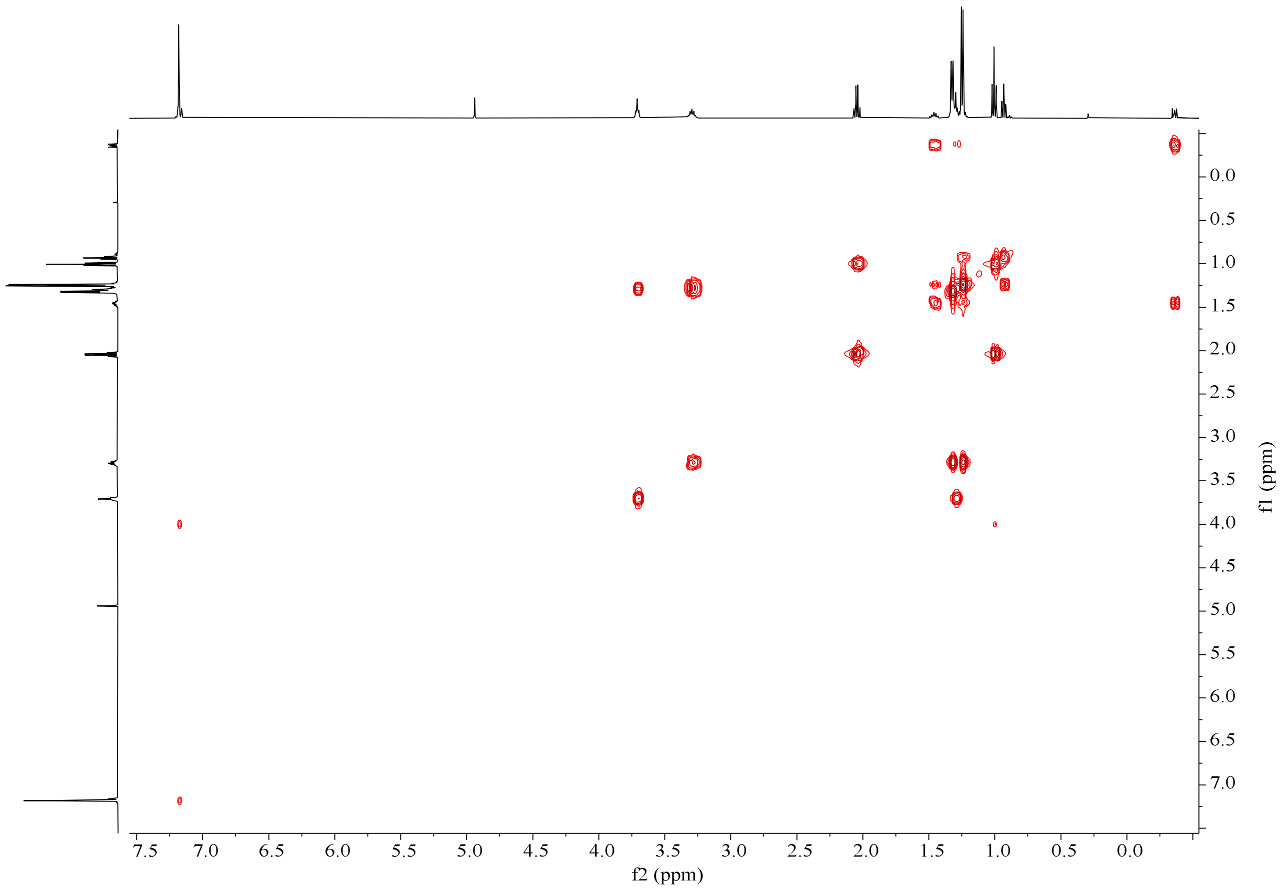

Figure S30. ^{1}H-^{1}H COSY NMR spectrum of isolated [(^{EtDip}nacnac)Mg(THF)*n*Bu].

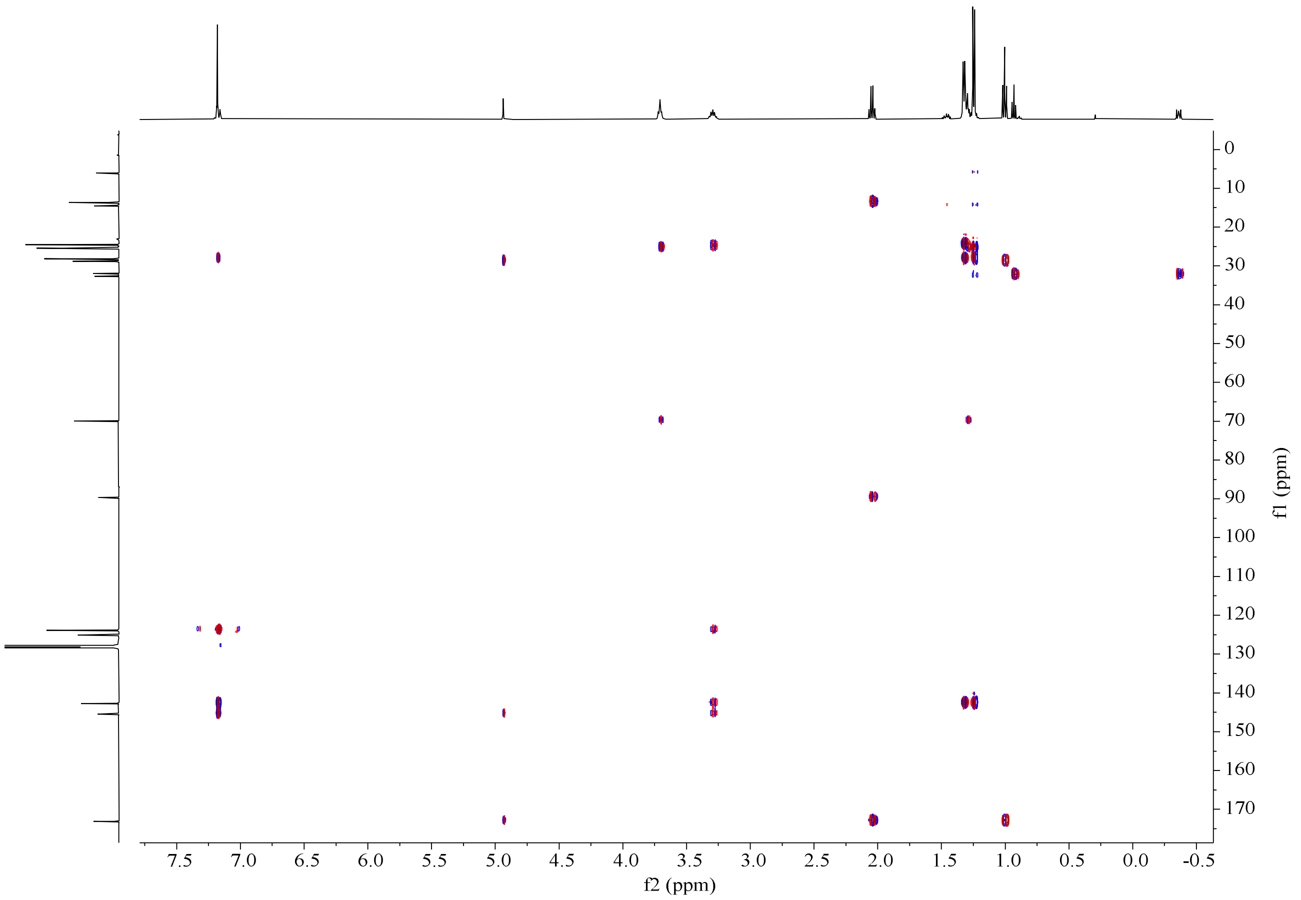

Figure S31. ^{1}H-^{13}C HMBC NMR spectrum of isolated [(^{EtDip}nacnac)Mg(THF)*n*Bu].

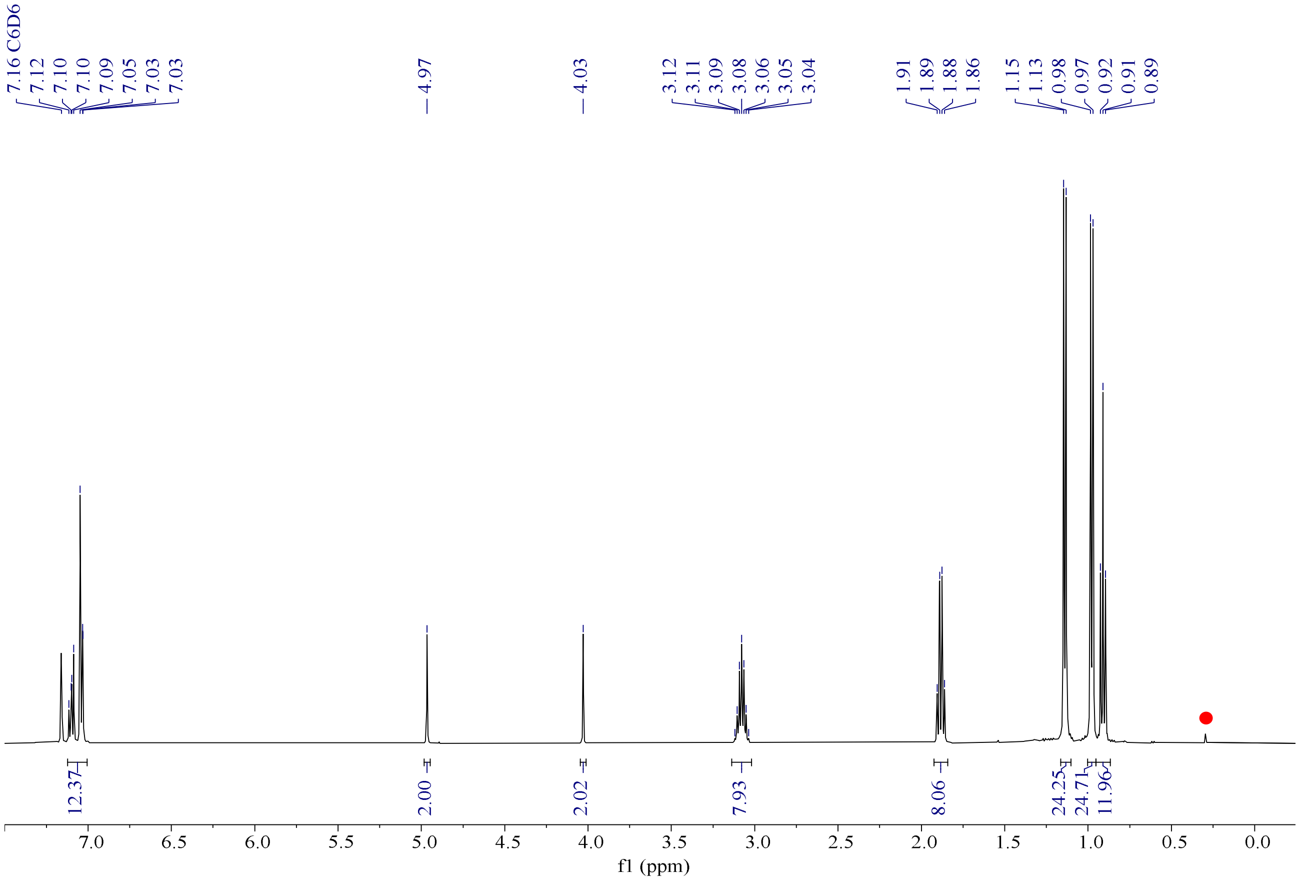

Figure S32. ^{1}H NMR spectrum (500.1 MHz, C_6D_6, 298 K) of isolated [{(^{EtDip}nacnac)Mg(μ-H)}$_2$] **7b**. The red circle denotes the resonance associated with silicone grease.

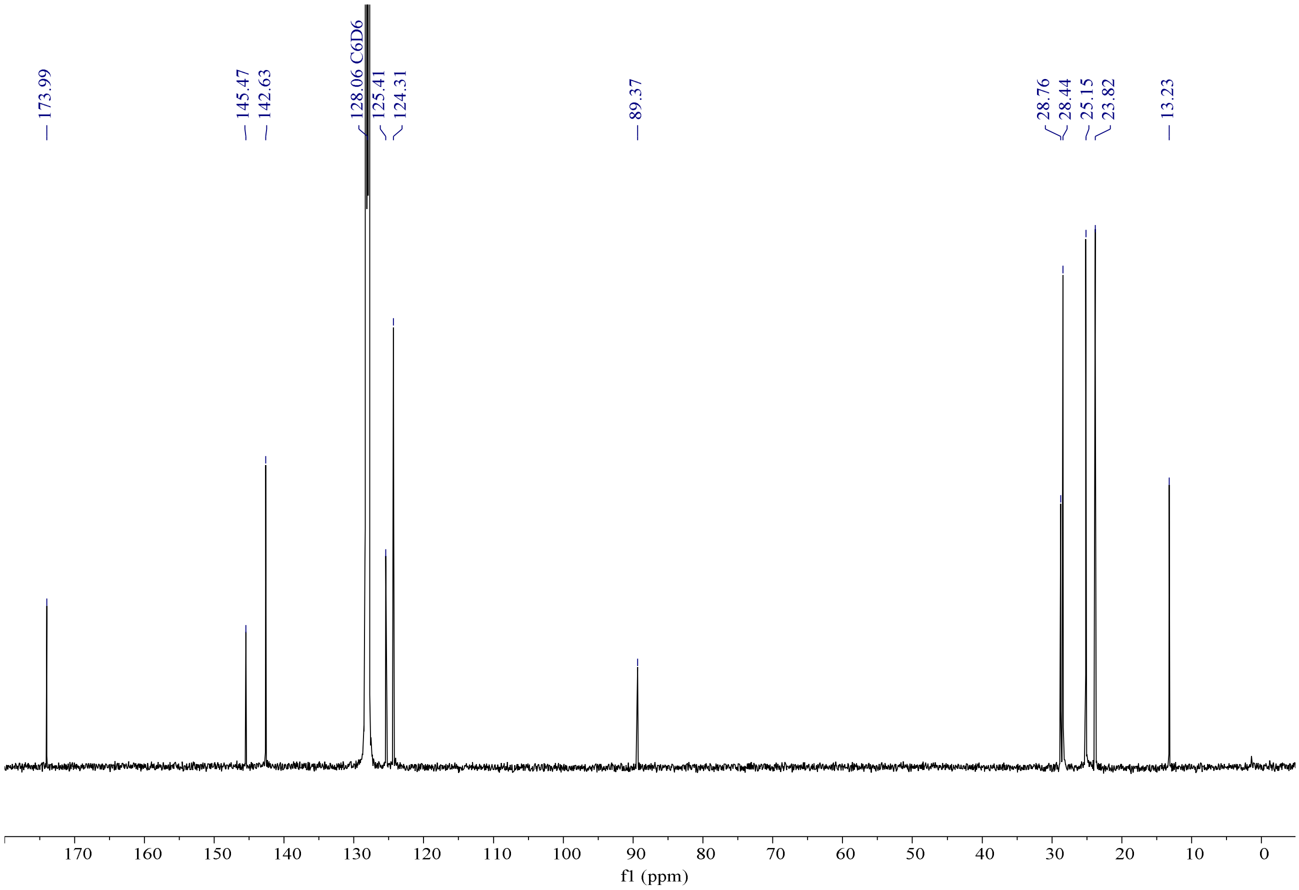

Figure S33. $^{13}C\{^{1}H\}$ NMR spectrum (125.8 MHz, C_6D_6, 298 K) of isolated [{(^{EtDip}nacnac)Mg(μ-H)}$_2$] **7b**.

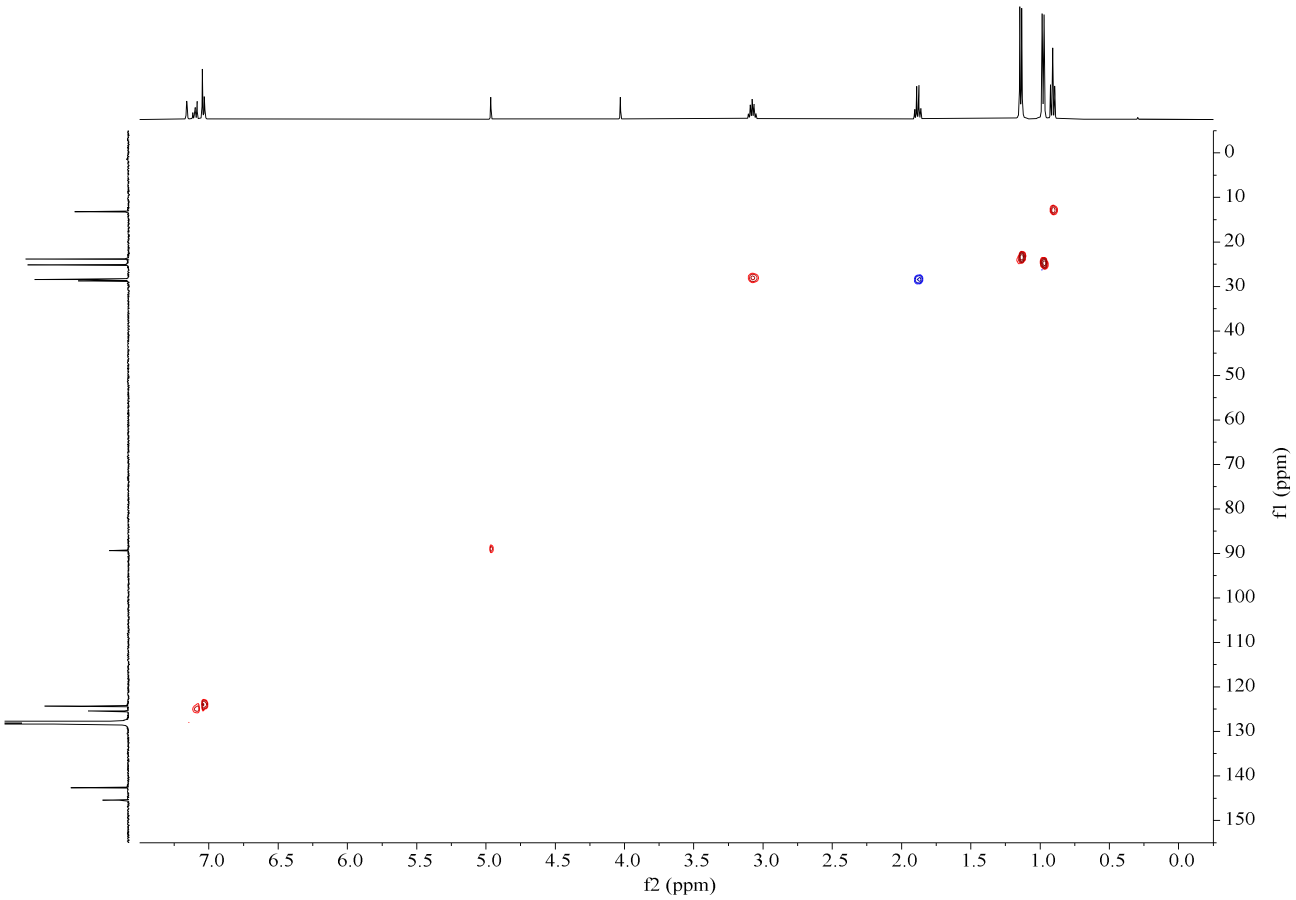

Figure S34. ^{1}H-^{13}C HSQC NMR spectrum of isolated [{(^{EtDip}nacnac)Mg(μ-H)}$_2$] **7b**.

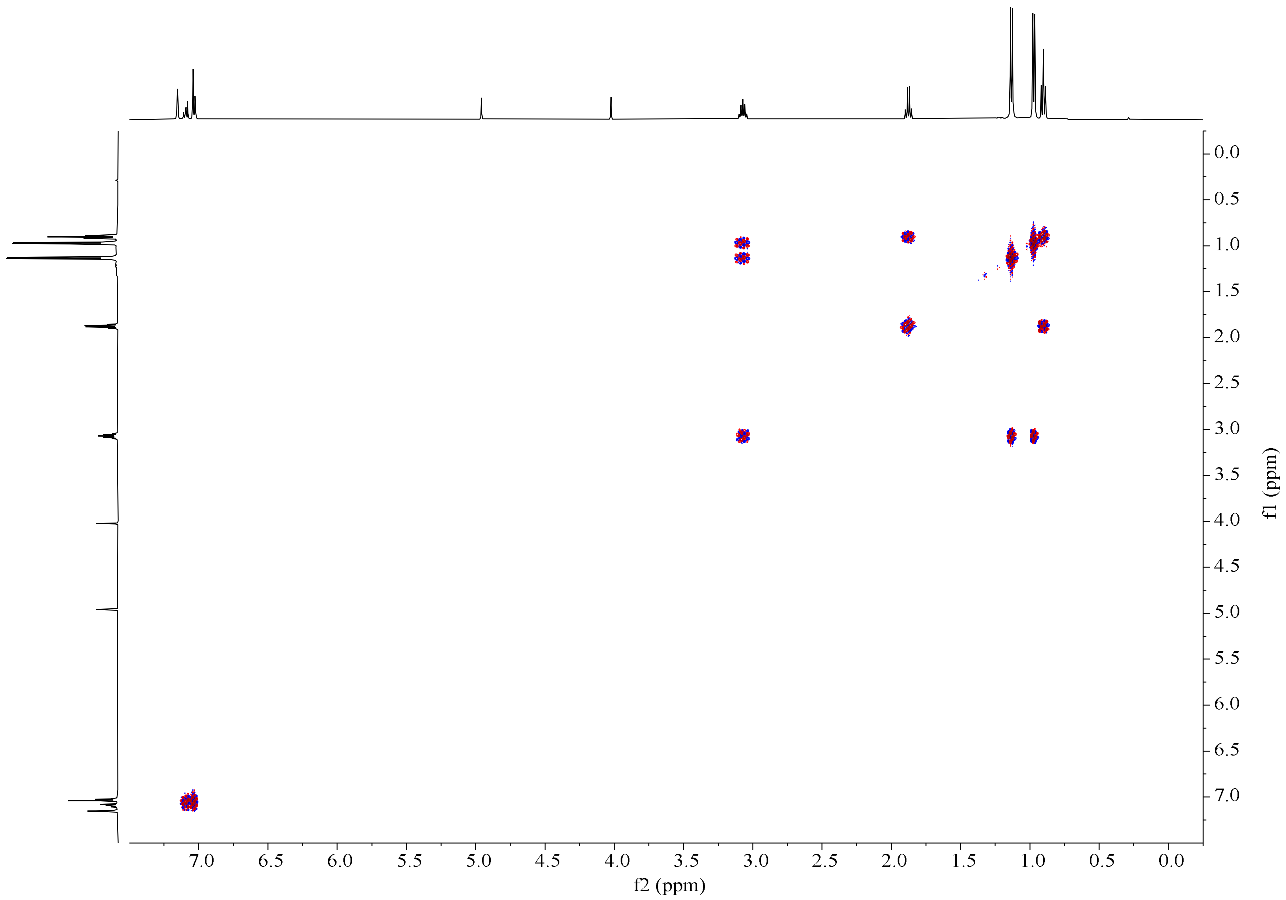

Figure S35. ^{1}H-^{1}H COSY NMR spectrum of isolated [{(^{EtDip}nacnac)Mg(μ-H)}$_2$] **7b**.

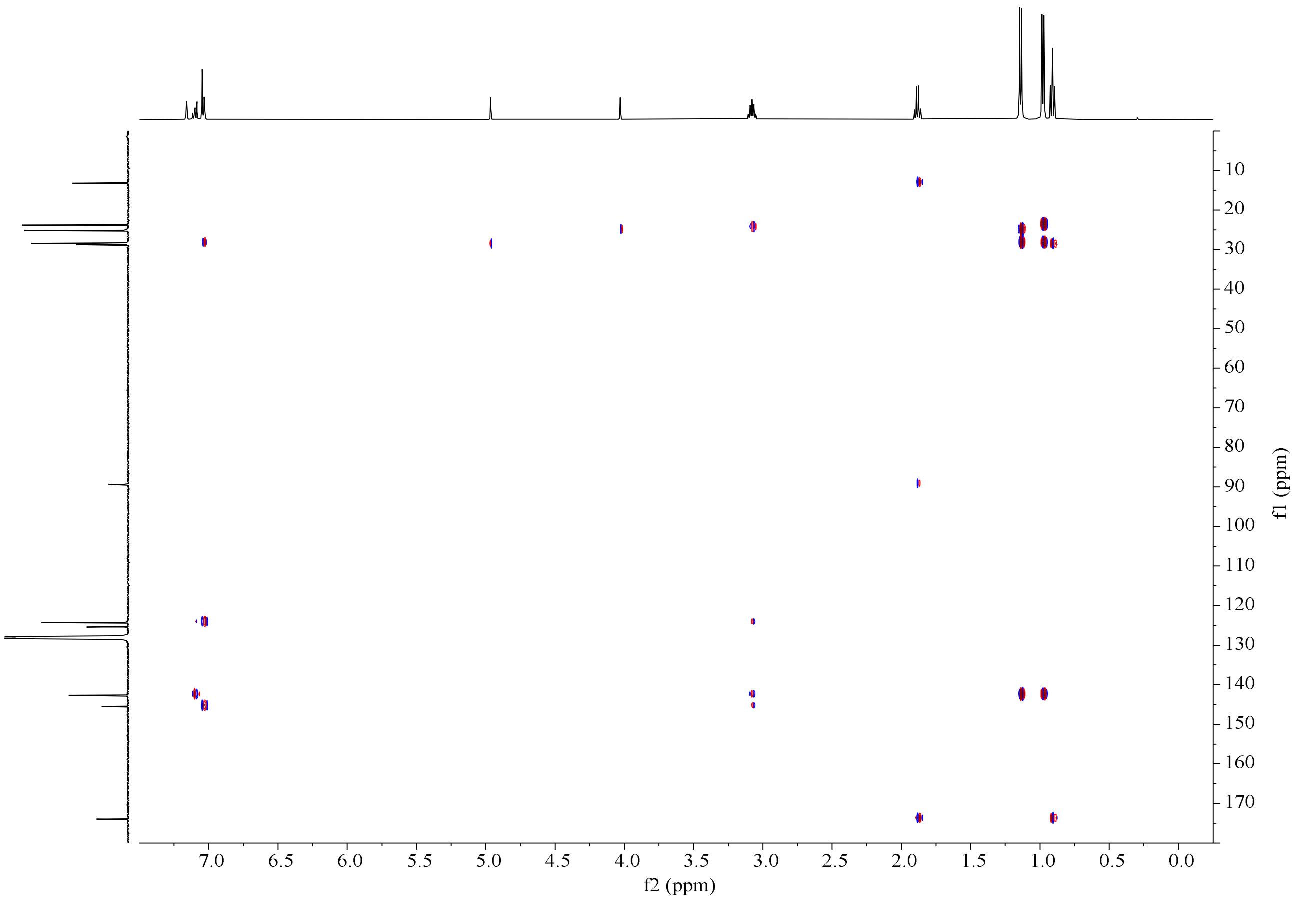

Figure S36. ^{1}H-^{13}C HMBC NMR spectrum of isolated [{(^{EtDip}nacnac)Mg(μ-H)}$_2$] **7b**.

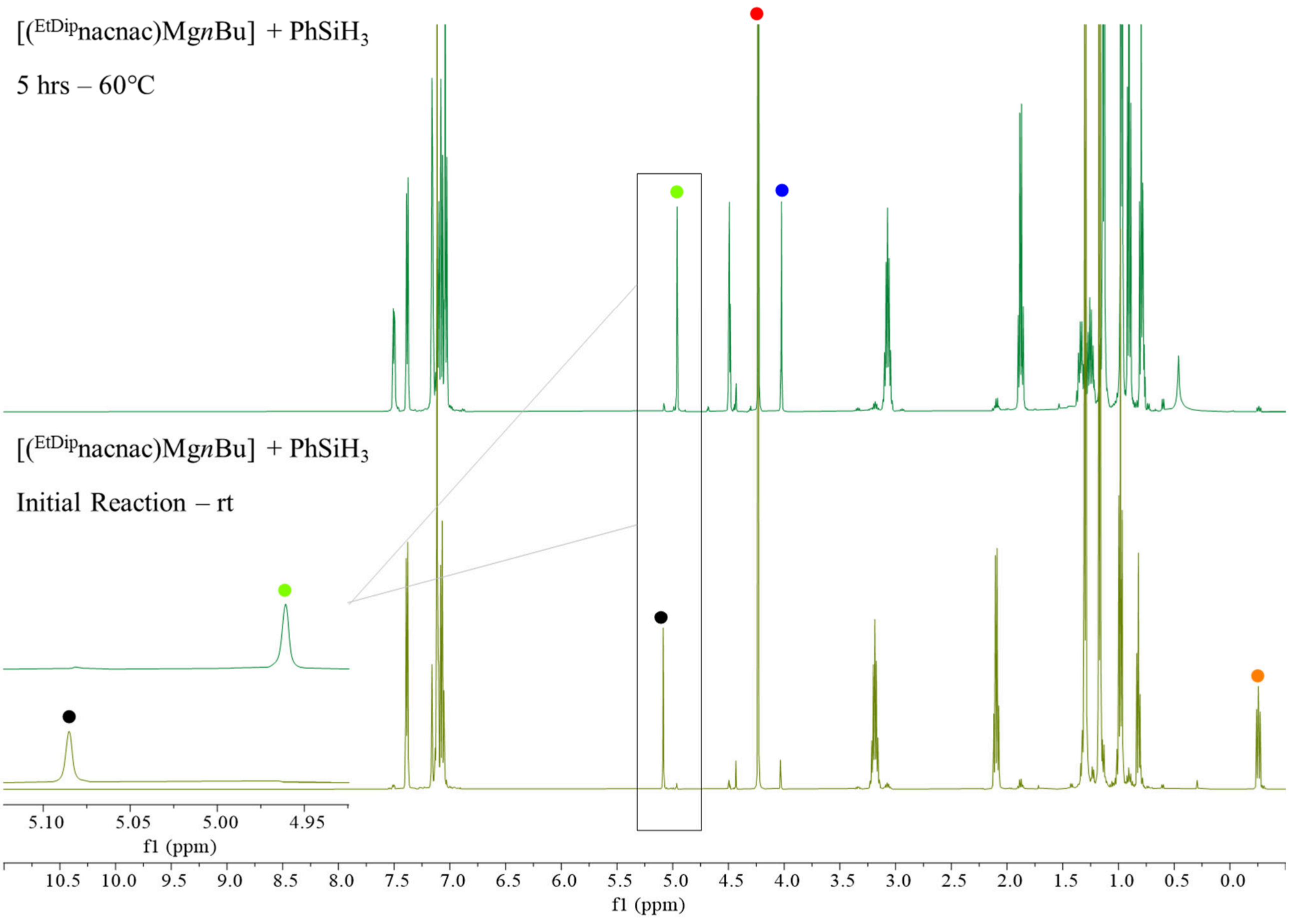

Figure S37. Stacked ^{1}H NMR spectra (500.1 MHz, C_6D_6, 298 K) of the reaction of a colourless solution of [(^{EtDip}nacnac)Mg*n*Bu] **9b** (21.9 mg, 41.5 µmol, 1.0 equiv.) with phenylsilane (5.6 µL, 45 µmol, 1.1 equiv.) in C_6D_6 (0.6 mL) in a J Young NMR tube. The black circle denotes the resonance associated with the backbone-C*H* of [(^{EtDip}nacnac)Mg*n*Bu] **9b**. The orange circle denotes the resonance associated with Mg-$CH_2C_3H_7$ of [(^{EtDip}nacnac)Mg*n*Bu] **9b**. The blue circle denotes the resonance associated with Mg-*H* of [{(^{EtDip}nacnac)Mg(µ-H)}$_2$] **7b**. The green circle denotes the resonance associated with the backbone-C*H* of [{(^{EtDip}nacnac)Mg(µ-H)}$_2$] **7b**. The red circle denotes the resonance associated with $PhSiH_3$.

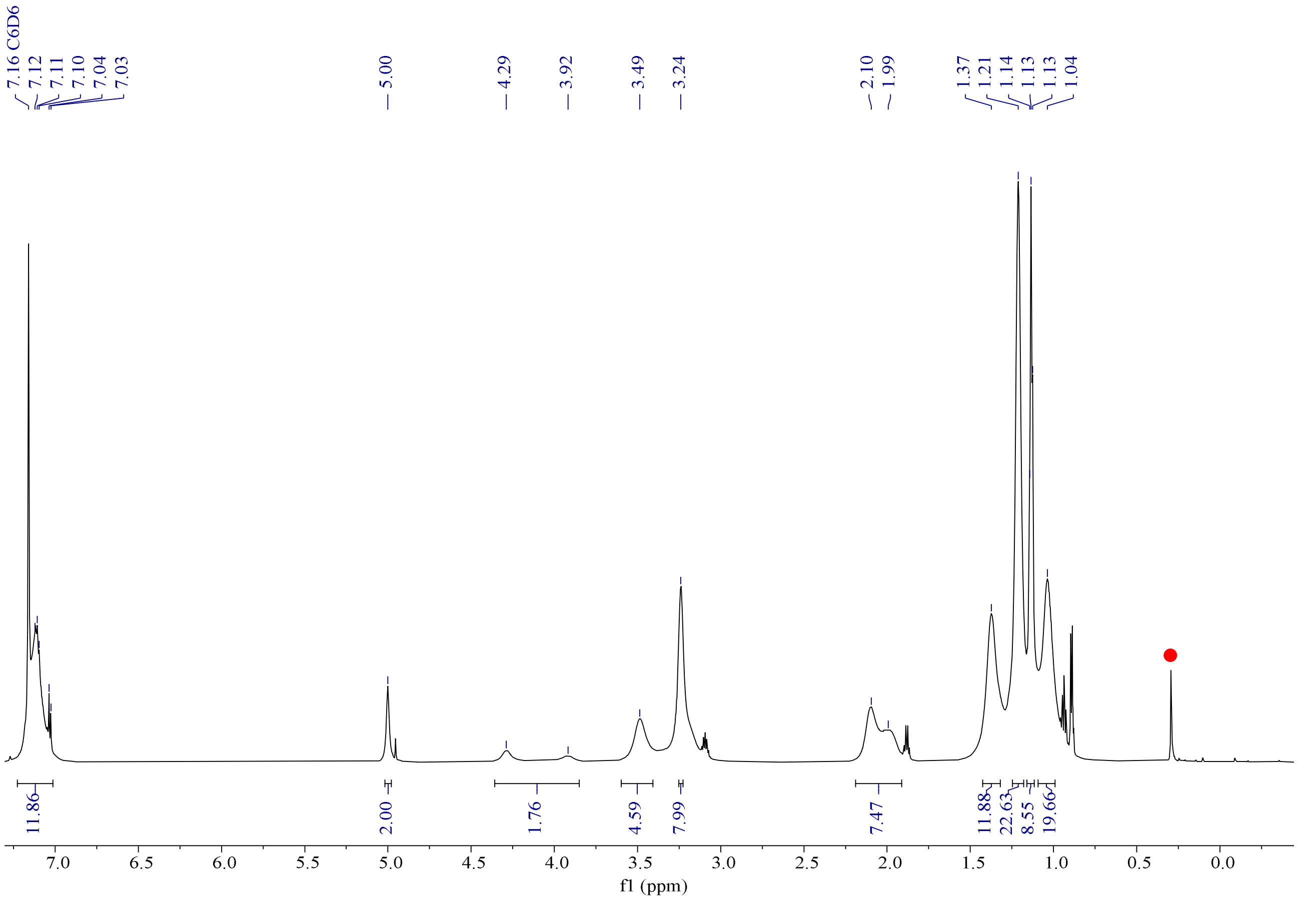

Figure S38. ^{1}H NMR spectrum (700.0 MHz, C_6D_6, 295 K) of *in-situ* generated [{(^{EtDip}nacnac)Mg(THF)(μ-H)}$_2$] **11b**. The red circle denotes the resonance associated with silicone grease.

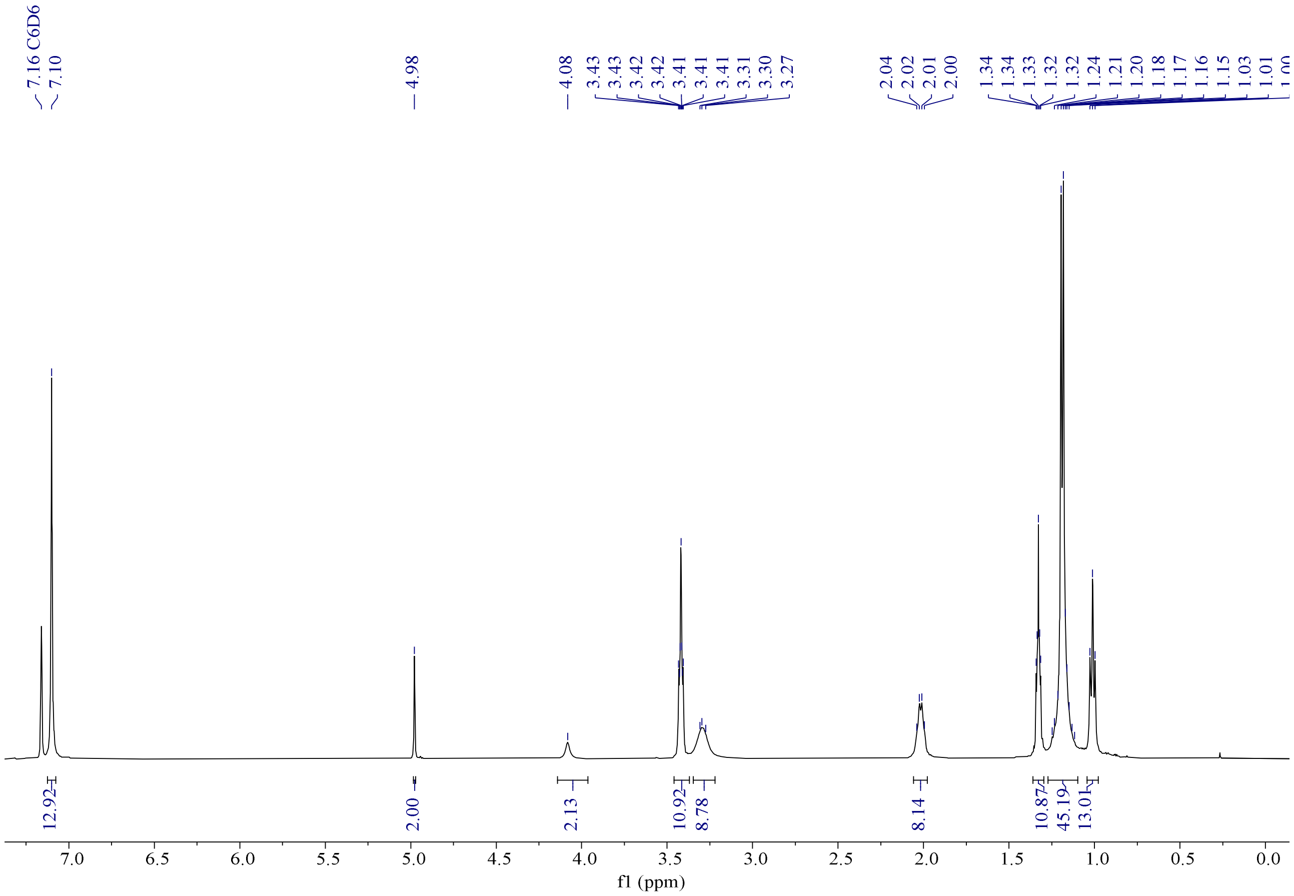

Figure S39. ^{1}H NMR spectrum (499.9 MHz, C_6D_6, 344 K) of *in-situ* generated [{(^{EtDip}nacnac)Mg(THF)(μ-H)}$_2$] **11b**.

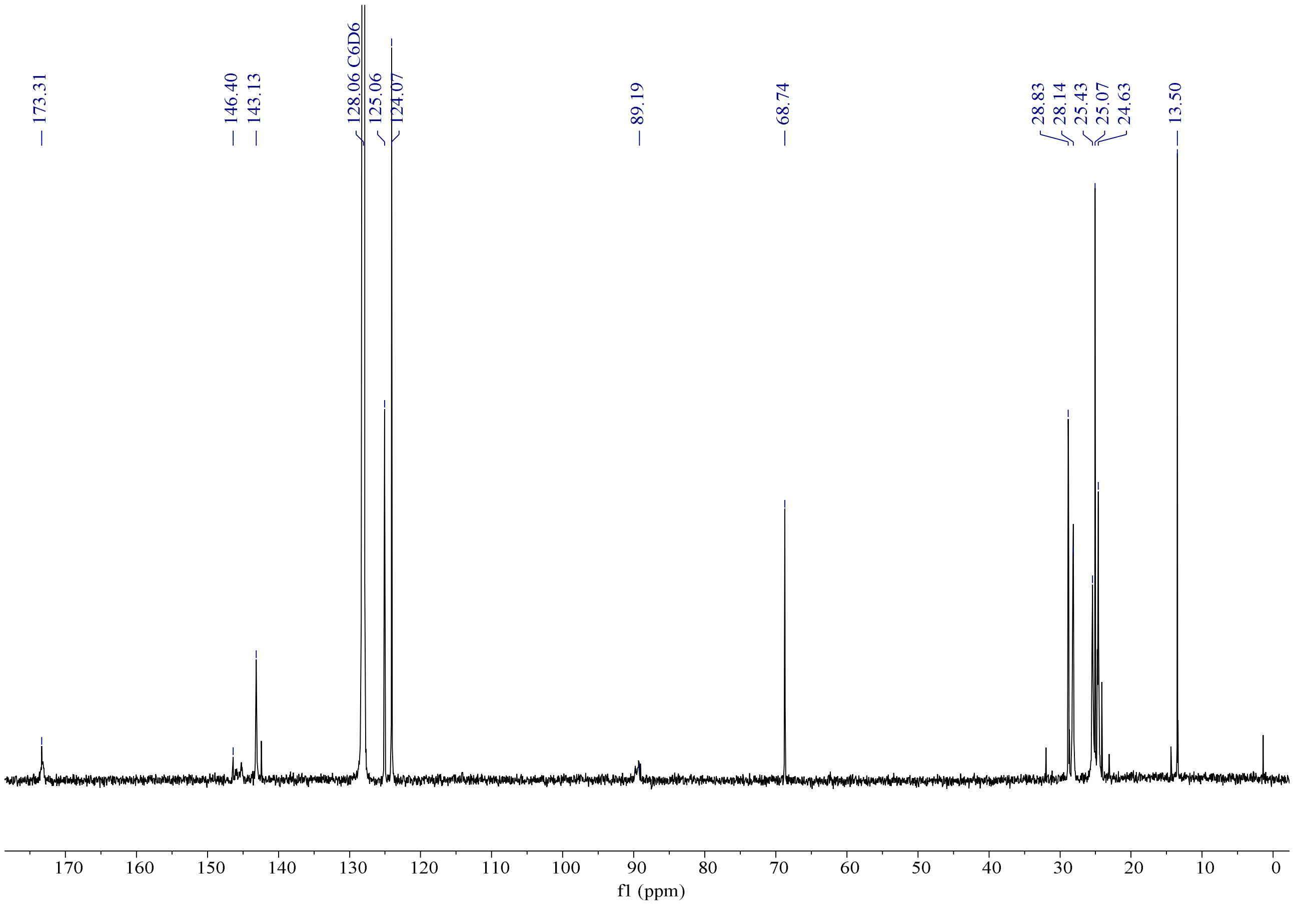

Figure S40. $^{13}C\{^{1}H\}$ NMR spectrum (176.0 MHz, C_6D_6, 295 K) of *in-situ* generated $[\{(^{EtDip}nacnac)Mg(THF)(\mu\text{-}H)\}_2]$ **11b**.

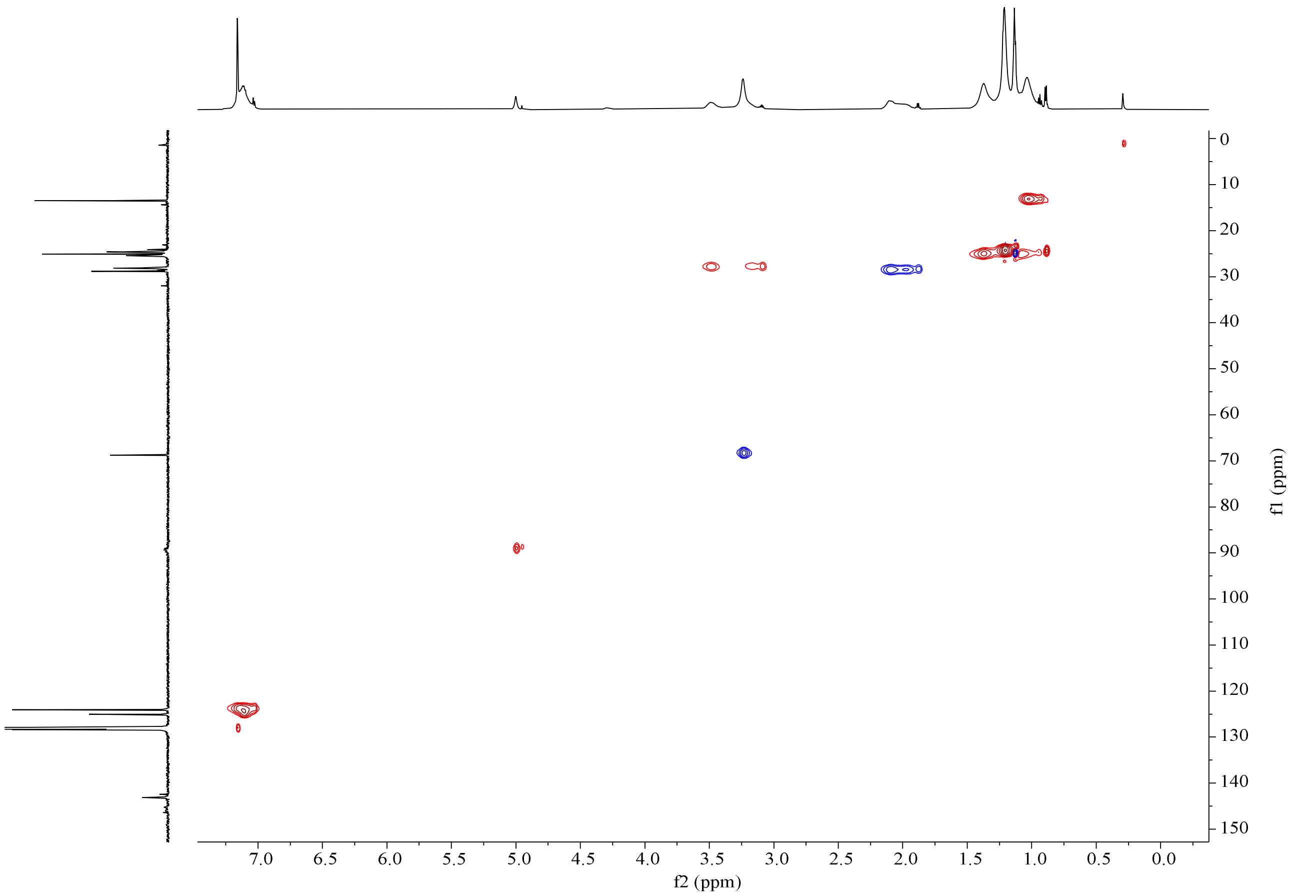

Figure S41. ^{1}H-^{13}C HSQC NMR spectrum of *in-situ* generated [{(^{EtDip}nacnac)Mg(THF)(μ-H)}$_2$] **11b**.

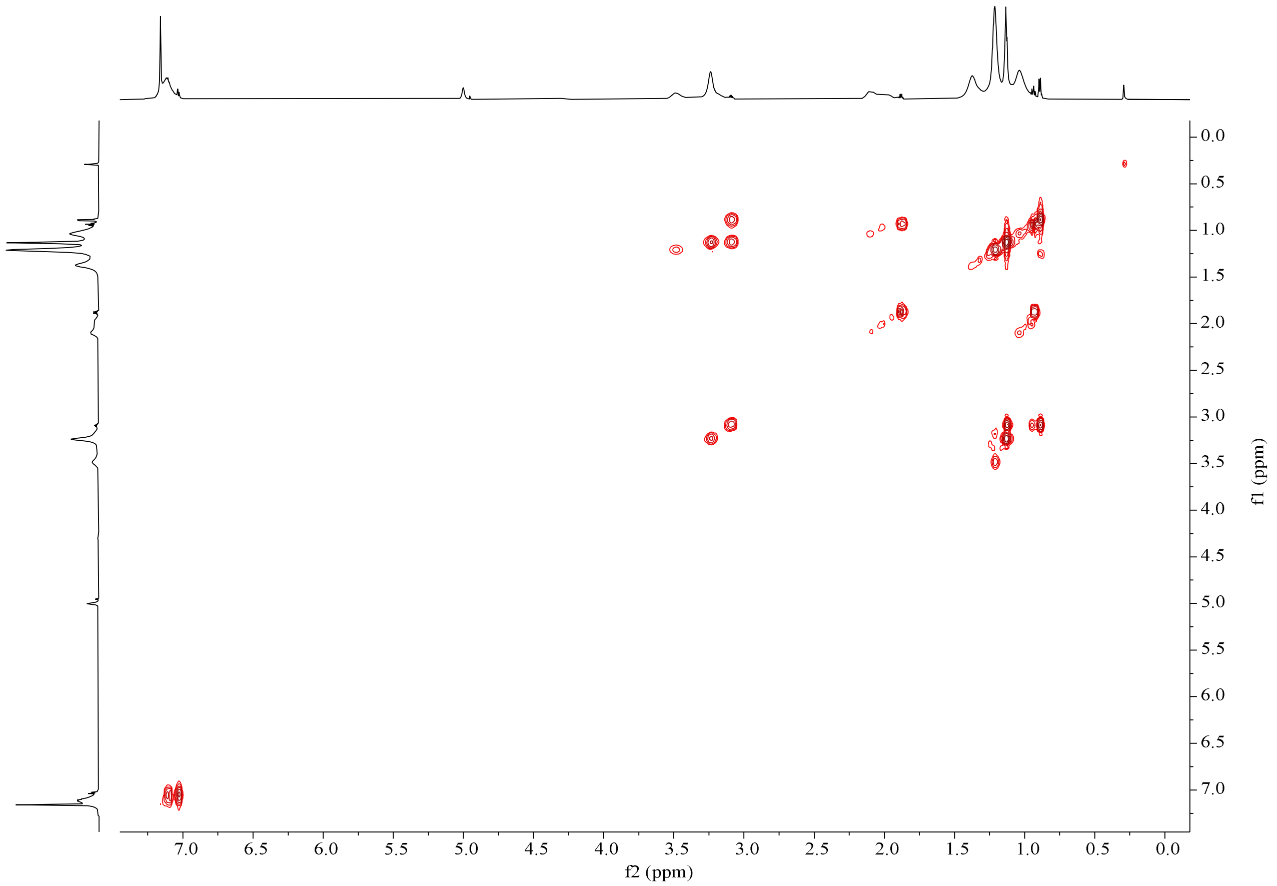

Figure S42. ^{1}H-^{1}H COSY NMR spectrum of *in-situ* generated [{(^{EtDip}nacnac)Mg(THF)(μ-H)}$_2$] **11b**.

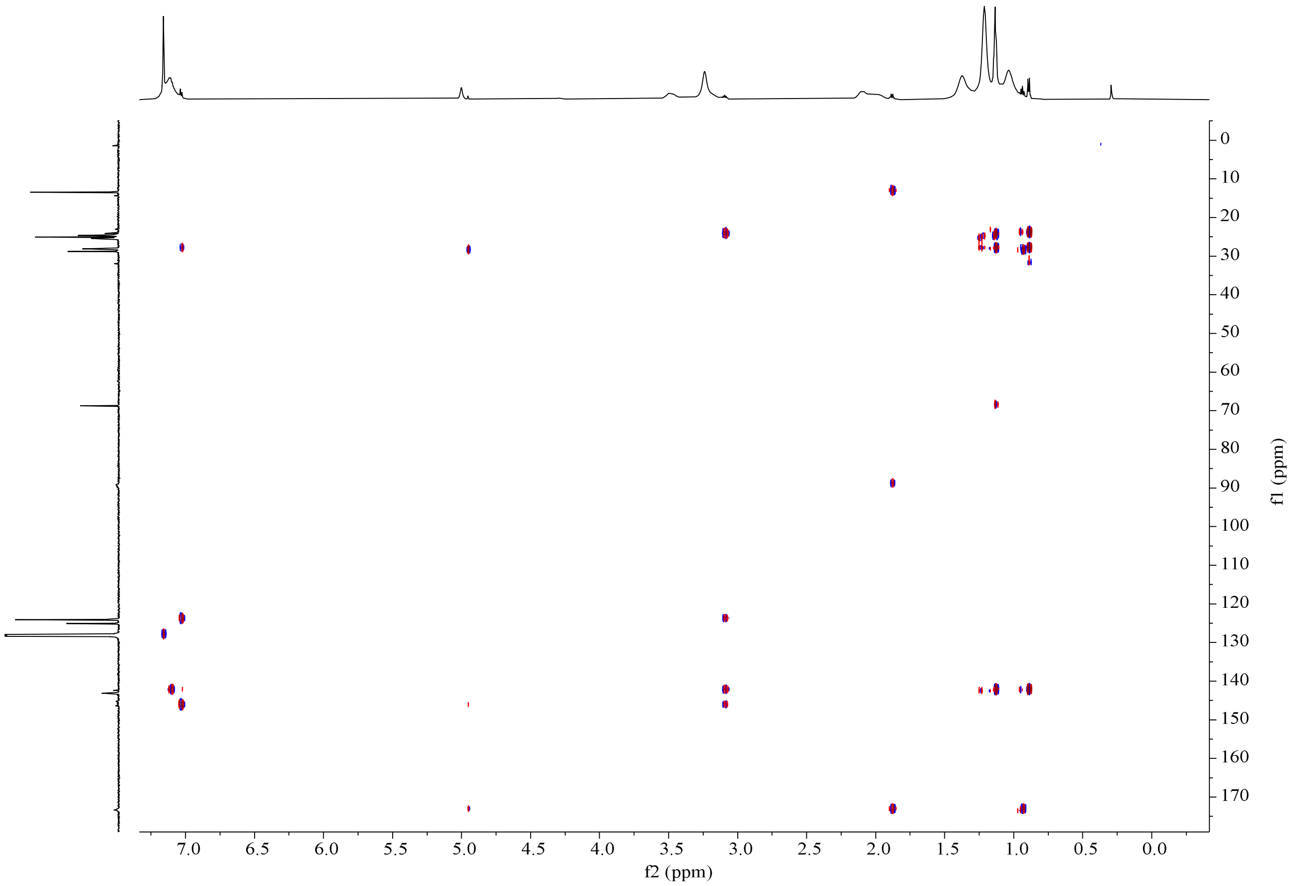

Figure S43. ^{1}H-^{13}C HMBC NMR spectrum of *in-situ* generated [{(^{EtDip}nacnac)Mg(THF)(μ-H)}$_2$] **11b**.

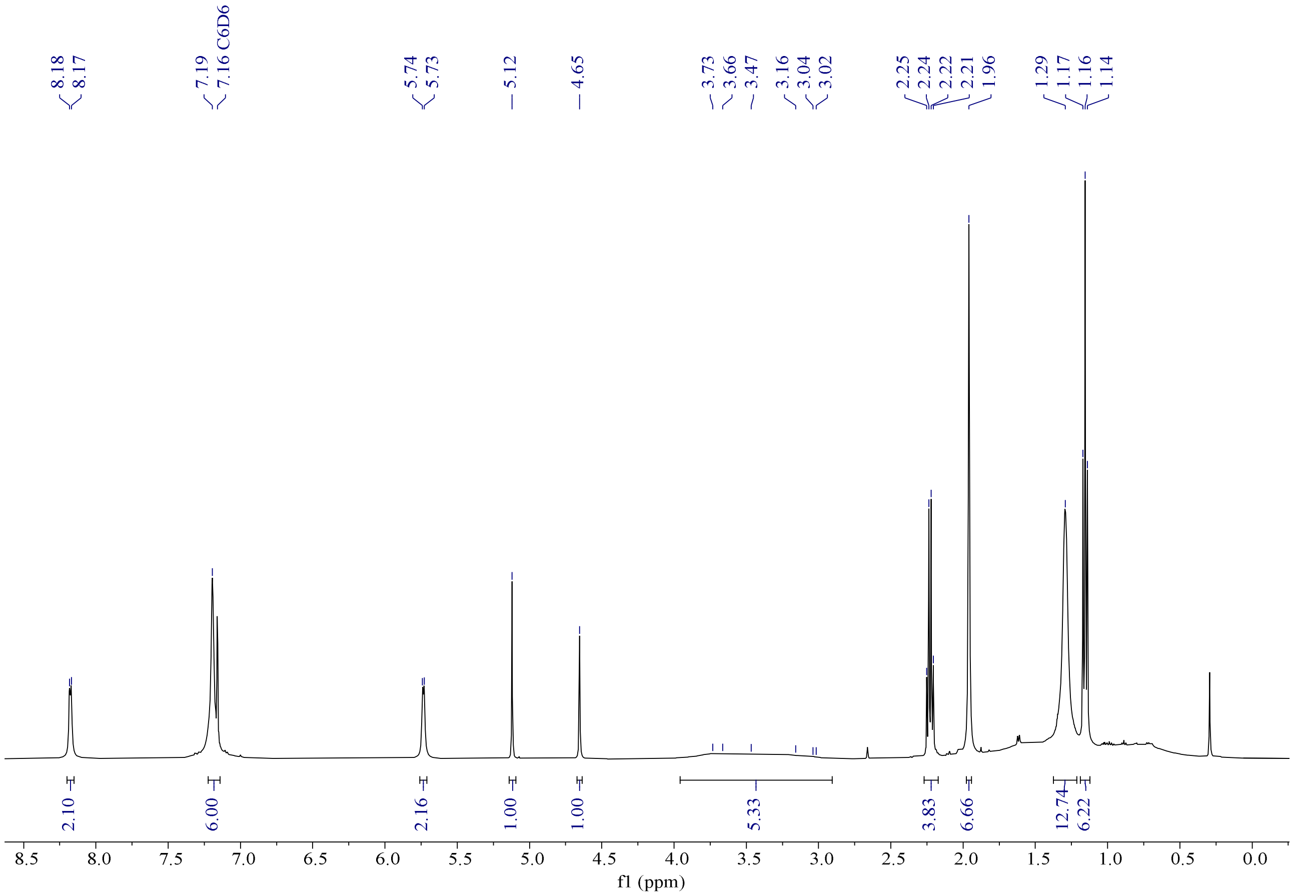

Figure S44. ^{1}H NMR spectrum (499.9 MHz, C_6D_6, 298 K) of *in-situ* generated [(^{EtDip}nacnac)Mg(DMAP)H] **12b**.

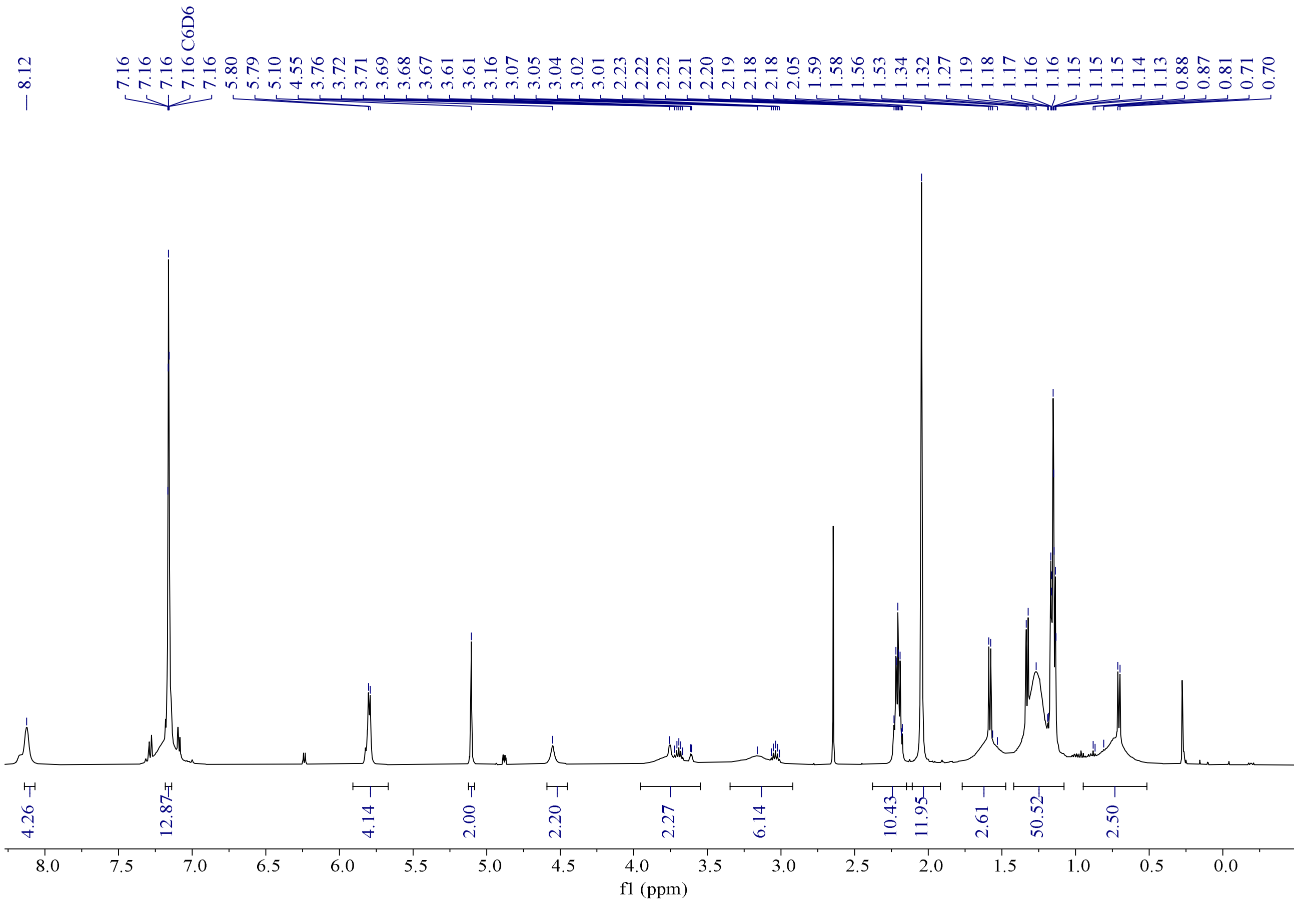

Figure S45. ^{1}H NMR spectrum (499.9 MHz, C_6D_6, 334 K) of *in-situ* generated [(^{EtDip}nacnac)Mg(DMAP)H] **12b**.

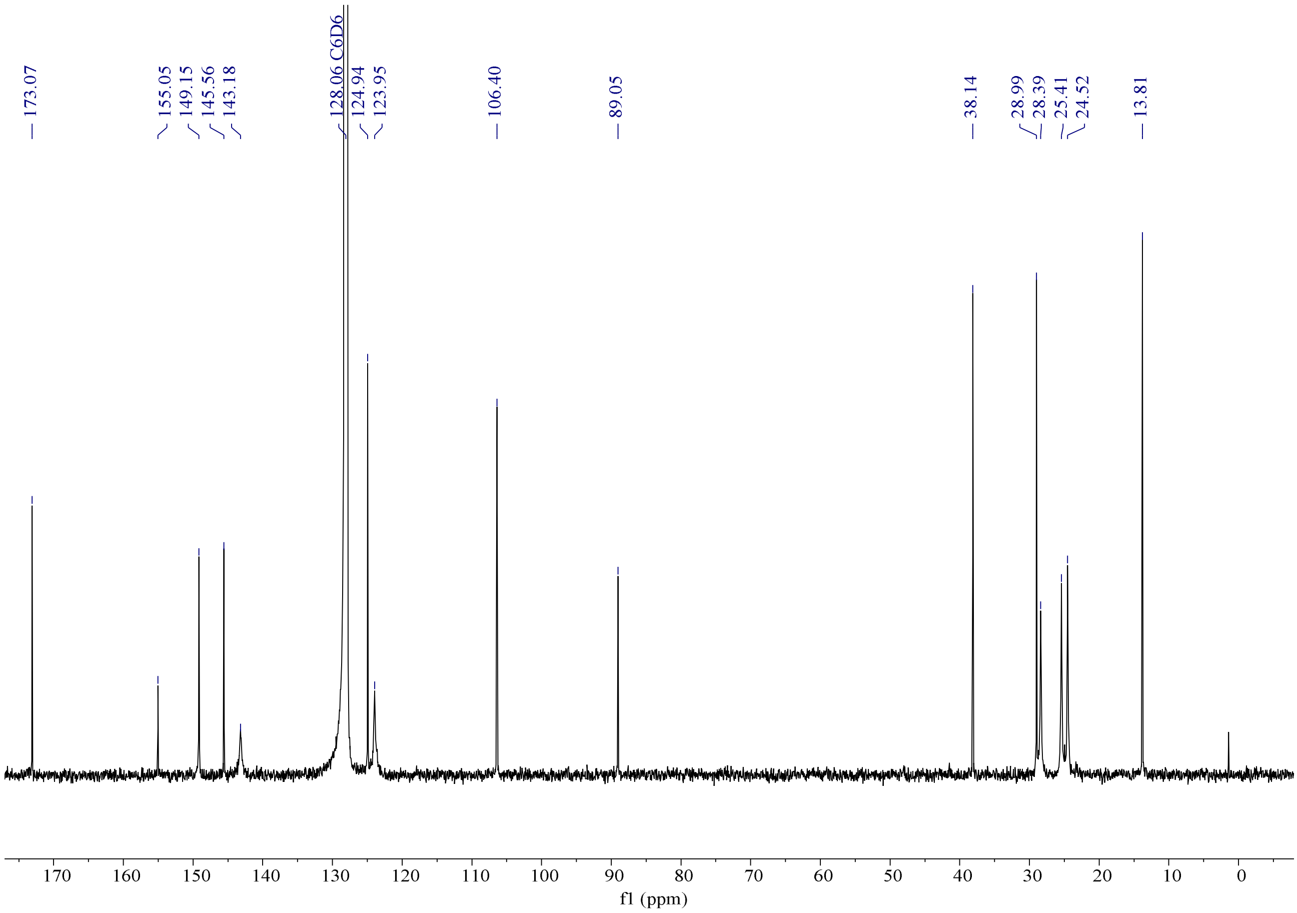

Figure S46. $^{13}C\{^{1}H\}$ NMR spectrum (125.7 MHz, C_6D_6, 298 K) of *in-situ* generated [(^{EtDip}nacnac)Mg(DMAP)H] **12b**.

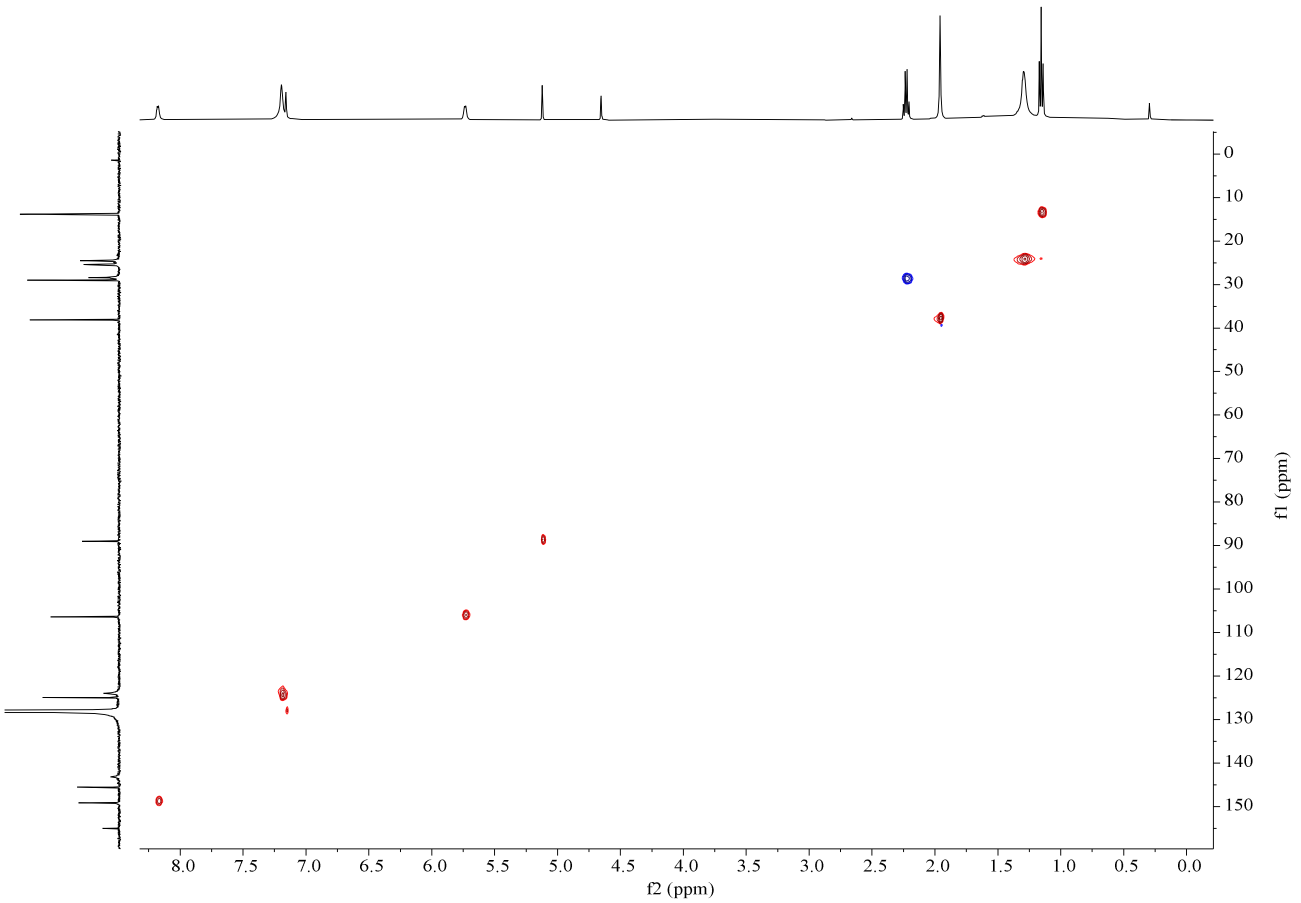

Figure S47. ^{1}H-^{13}C HSQC NMR spectrum of *in-situ* generated [(^{EtDip}nacnac)Mg(DMAP)H] **12b**.

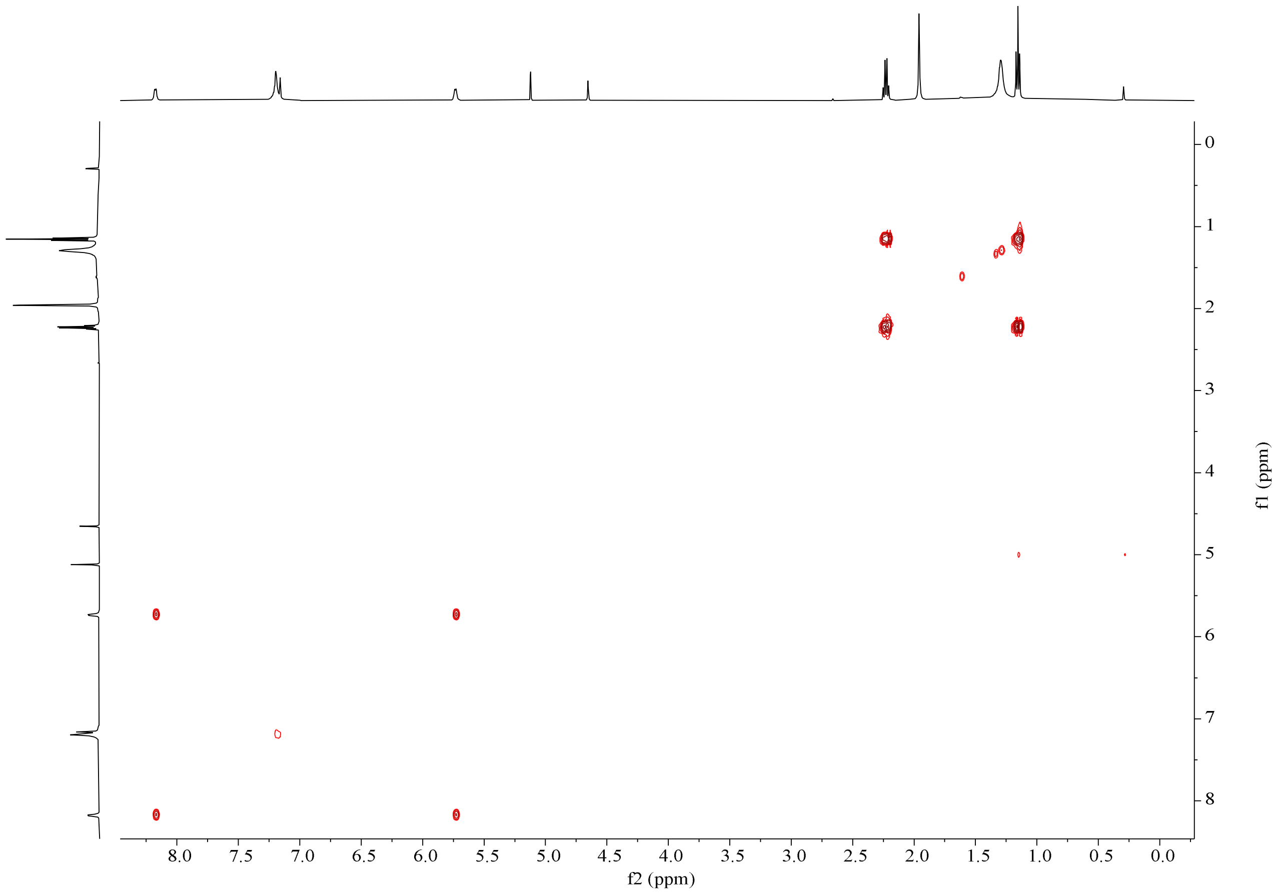

Figure S48. ^{1}H-^{1}H COSY NMR spectrum of *in-situ* generated [(^{EtDip}nacnac)Mg(DMAP)H] **12b**.

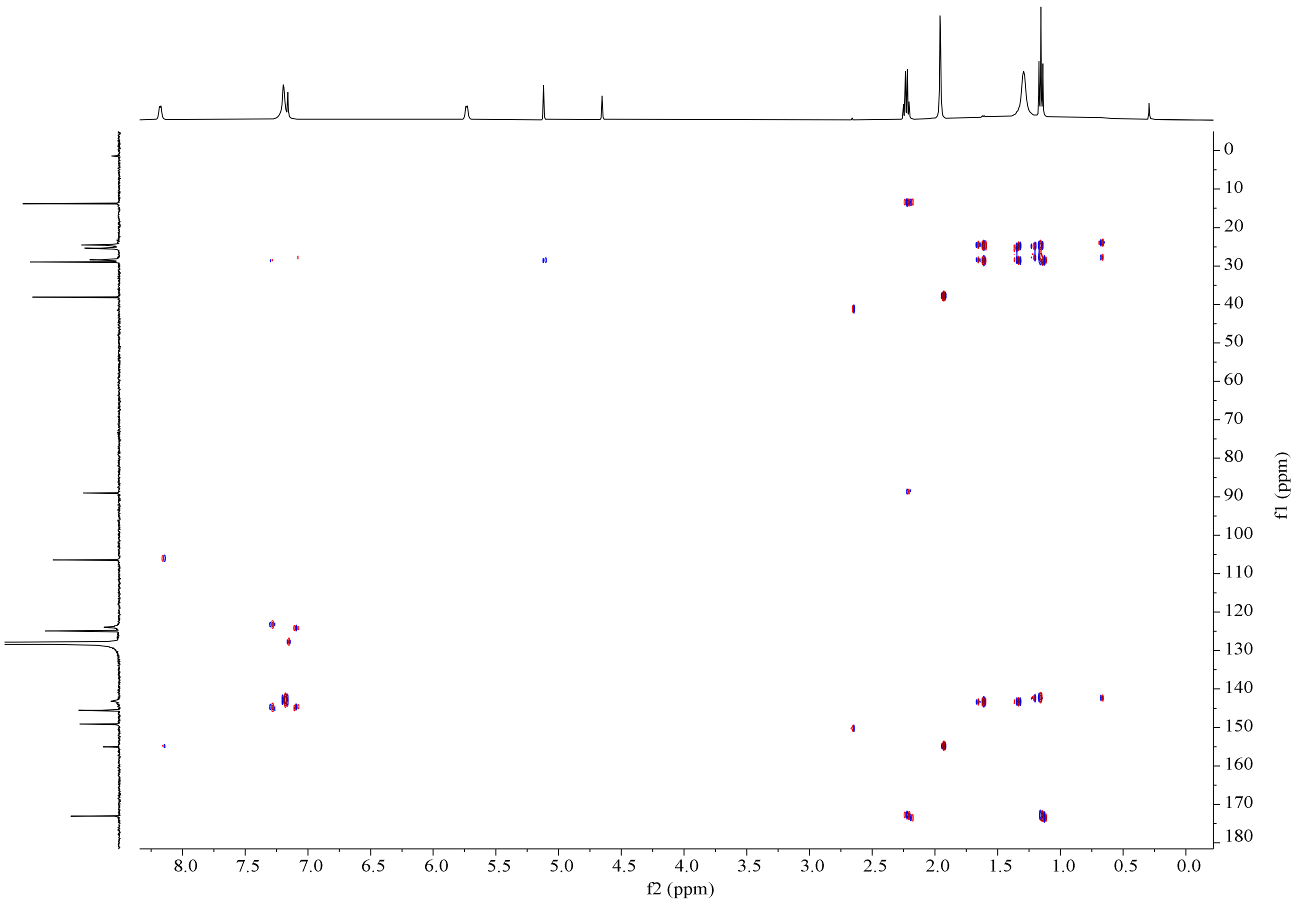

Figure S49. ^{1}H-^{13}C HMBC NMR spectrum of *in-situ* generated [(^{EtDip}nacnac)Mg(DMAP)H] **12b**.

2.4 Synthesis of magnesium oxide complexes and hydrogen activation experiments

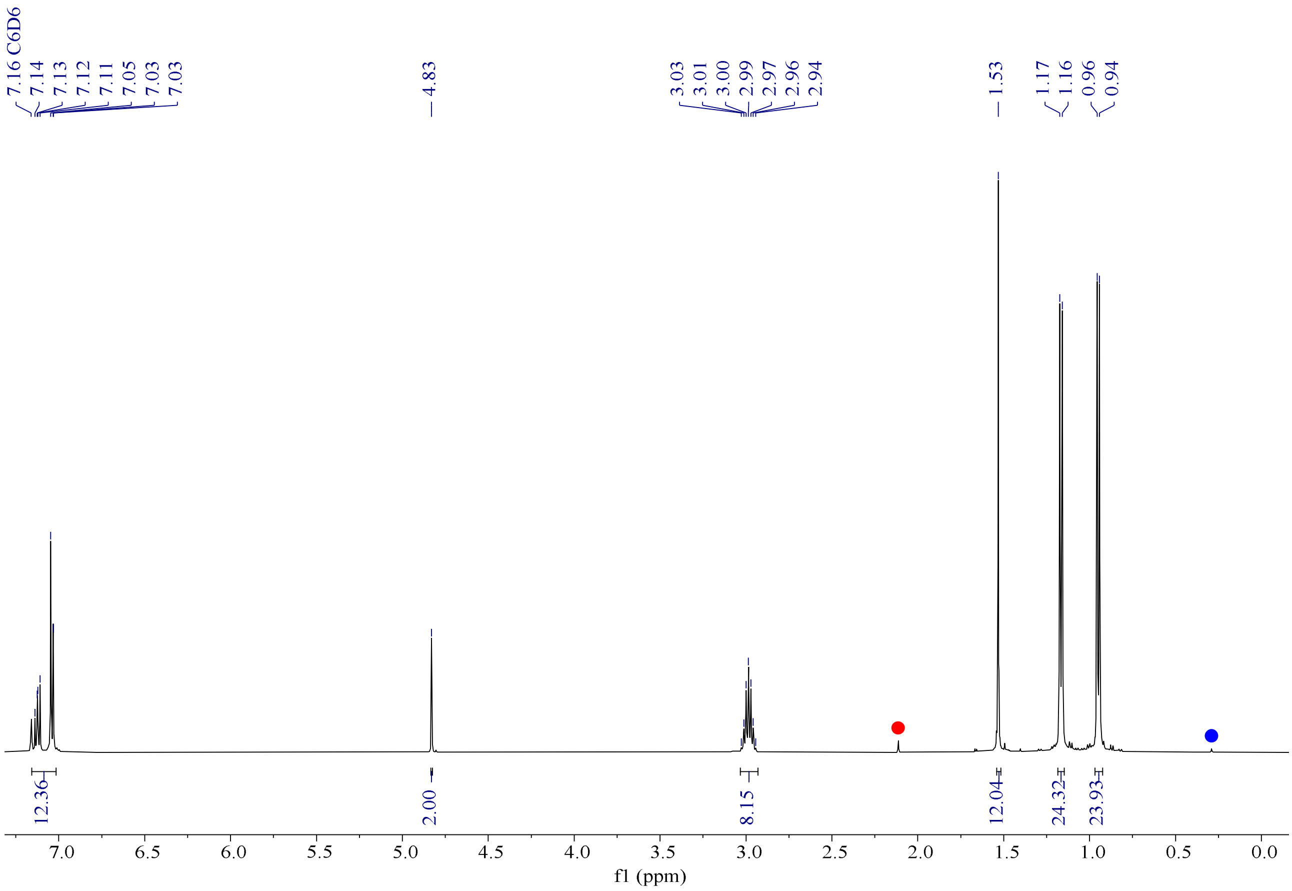

Figure S50. ^{1}H NMR spectrum (499.9 MHz, C_6D_6, 298 K) of *in-situ* generated $[\{(^{MeDip}nacnac)Mg\}_2(\mu\text{-}O)]$ **1a**. The blue circle denotes the resonance associated with silicone grease. The red circle denotes the resonance associated with toluene (Ph-CH_3).

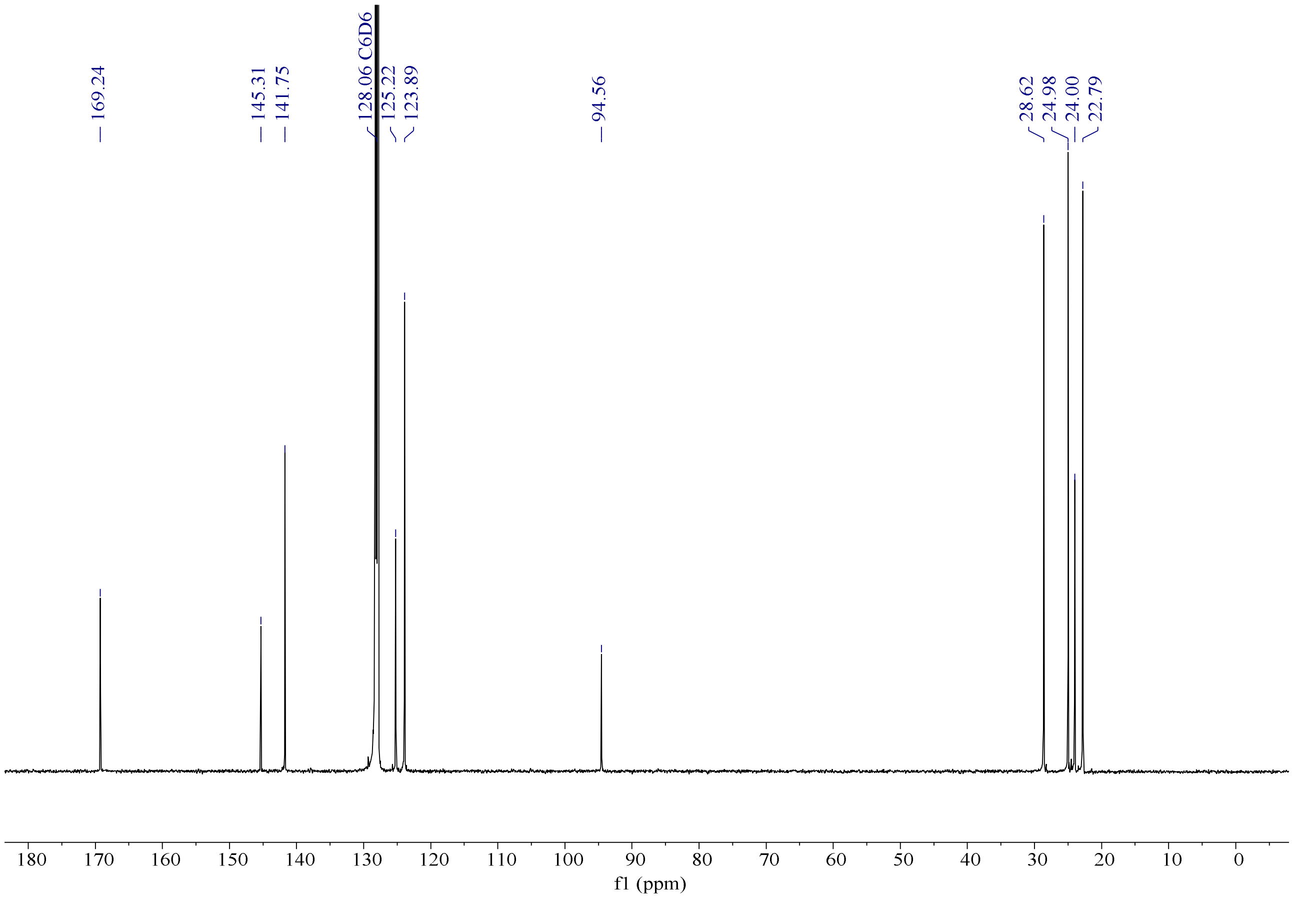

Figure S51. $^{13}C\{^{1}H\}$ NMR spectrum (125.7 MHz, C_6D_6, 298 K) of *in-situ* generated $[\{(^{MeDip}nacnac)Mg\}_2(\mu\text{-}O)]$ **1a.**

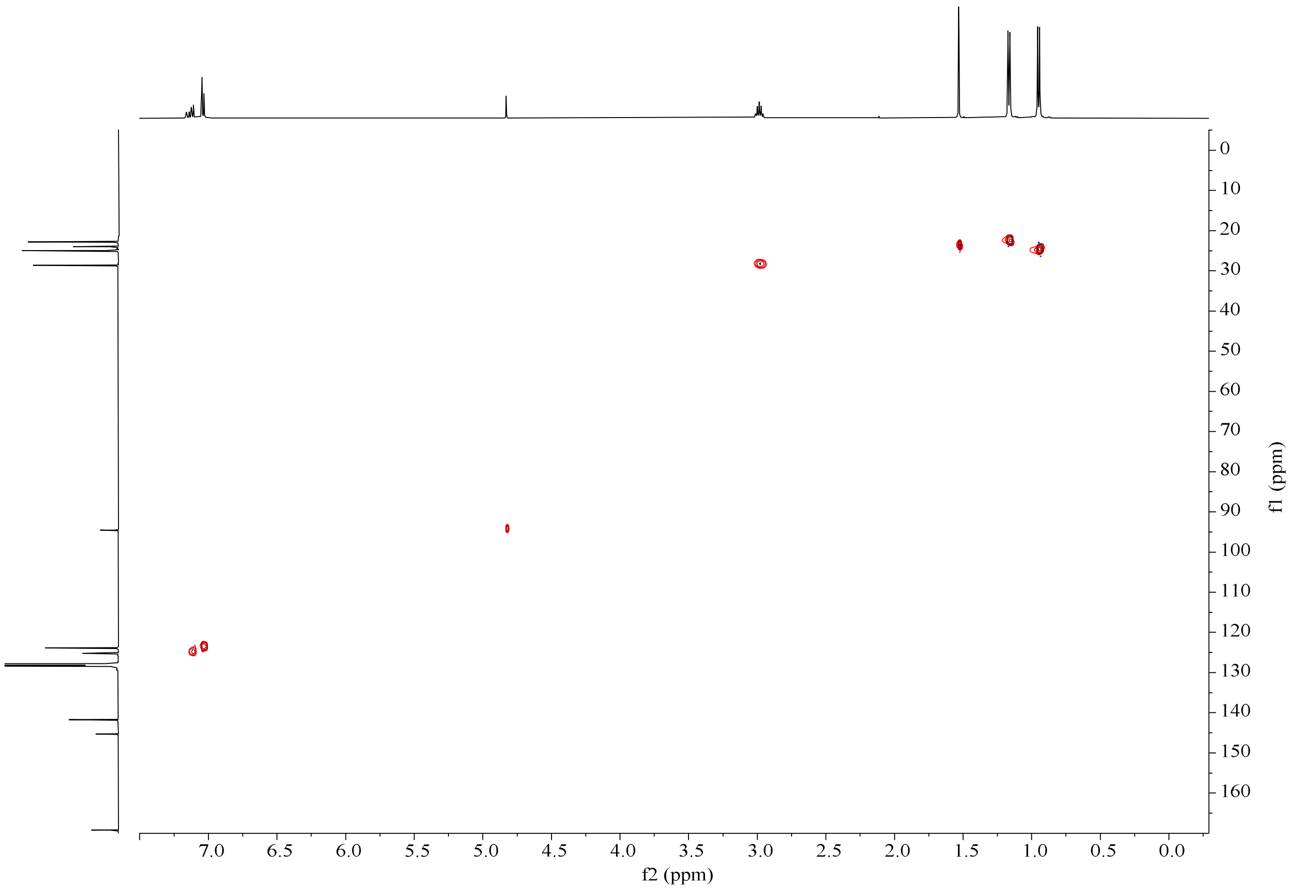

Figure S52. ^{1}H-^{13}C HSQC NMR spectrum of *in-situ* generated [{(^{MeDip}nacnac)Mg}$_2$(μ-O)] **1a**.

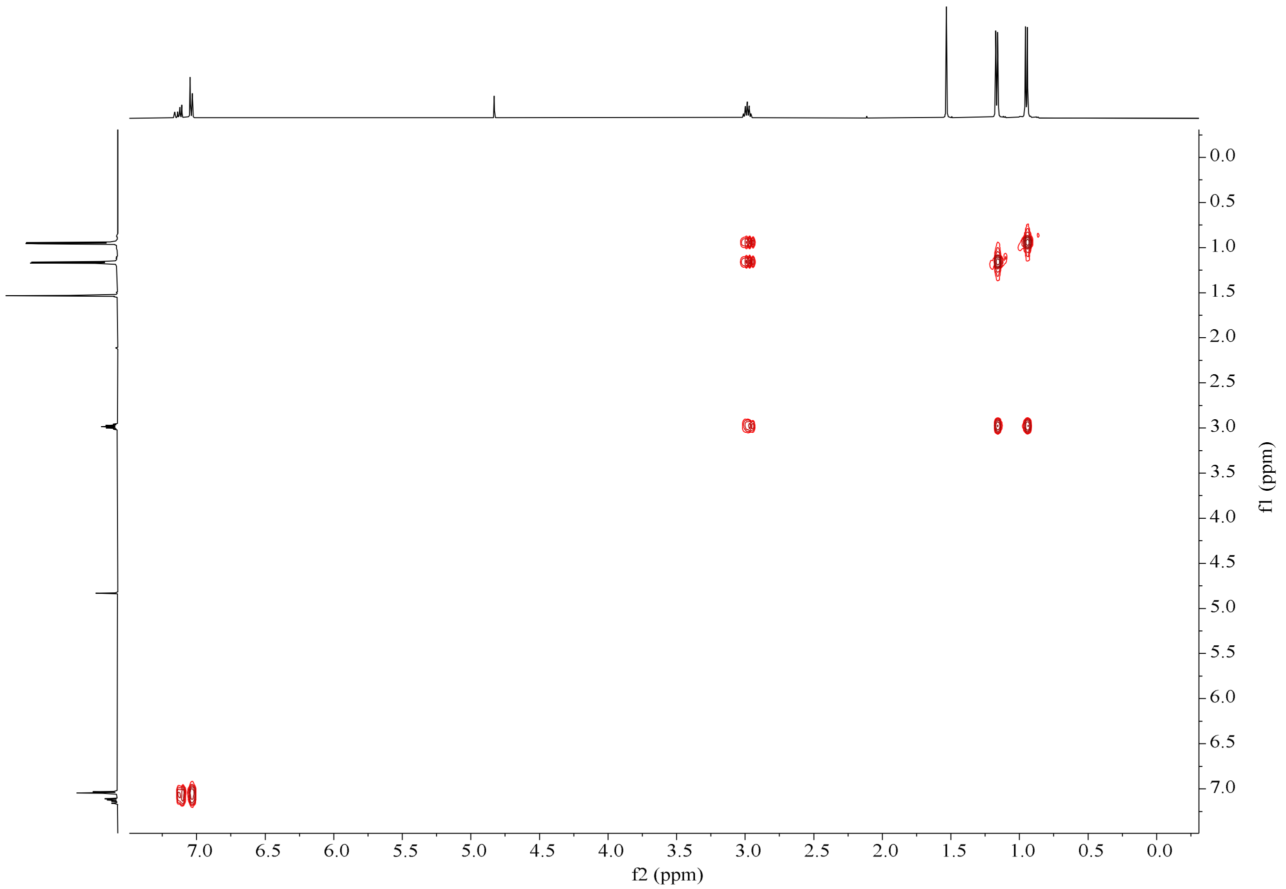

Figure S53. ^{1}H-^{1}H COSY NMR spectrum of *in-situ* generated [{(^{MeDip}nacnac)Mg}$_2$(μ-O)] **1a**.

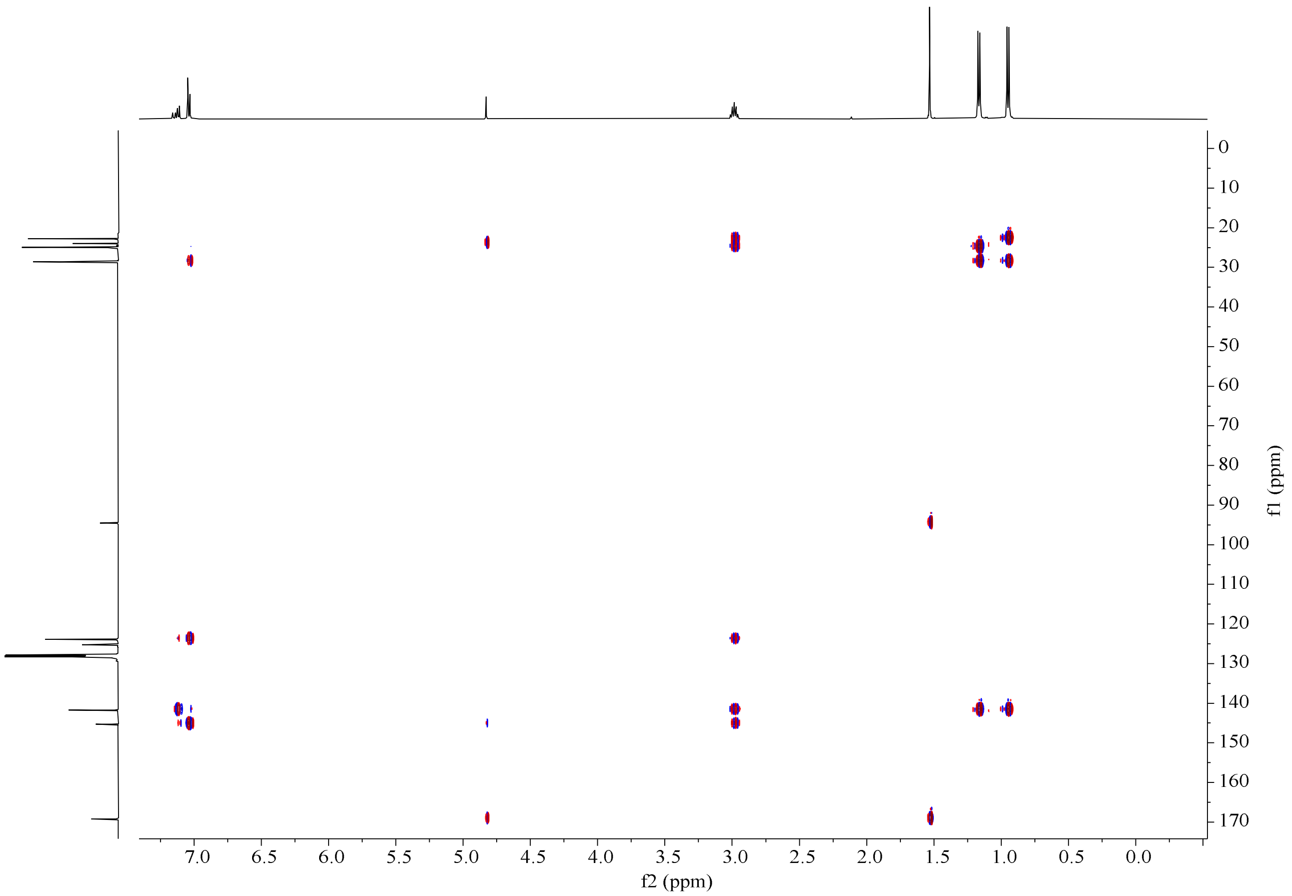

Figure S54. ^{1}H-^{13}C HMBC NMR spectrum of *in-situ* generated [{(^{MeDip}nacnac)Mg}$_2$(μ-O)] **1a**.

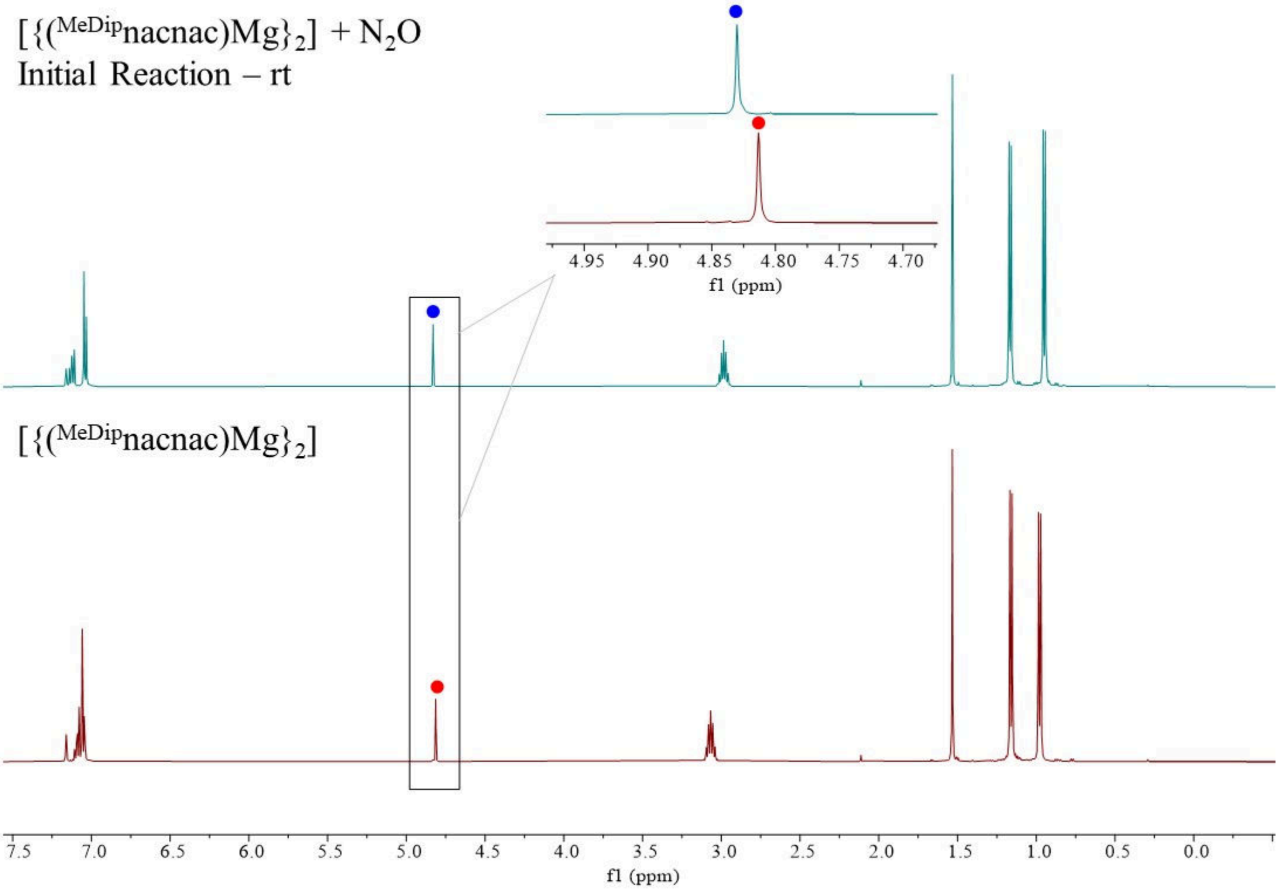

Figure S55. Stacked ^{1}H NMR spectra (499.9 MHz, C_6D_6, 298 K) of the reaction of a yellow solution of [{(^{MeDip}nacnac)Mg}$_2$] **2a** (18.0 mg, 20.4 μmol) and nitrous oxide (ca. 1 bar) in C_6D_6 (0.6 mL) at room temperature (rt) in a J Young NMR tube. The red circle denotes the resonance associated with the backbone-C*H* of [{(^{MeDip}nacnac)Mg}$_2$] **2a**. The blue circle denotes the resonance associated with the backbone-C*H* of [{(^{MeDip}nacnac)Mg}$_2$(μ-O)] **1a**.

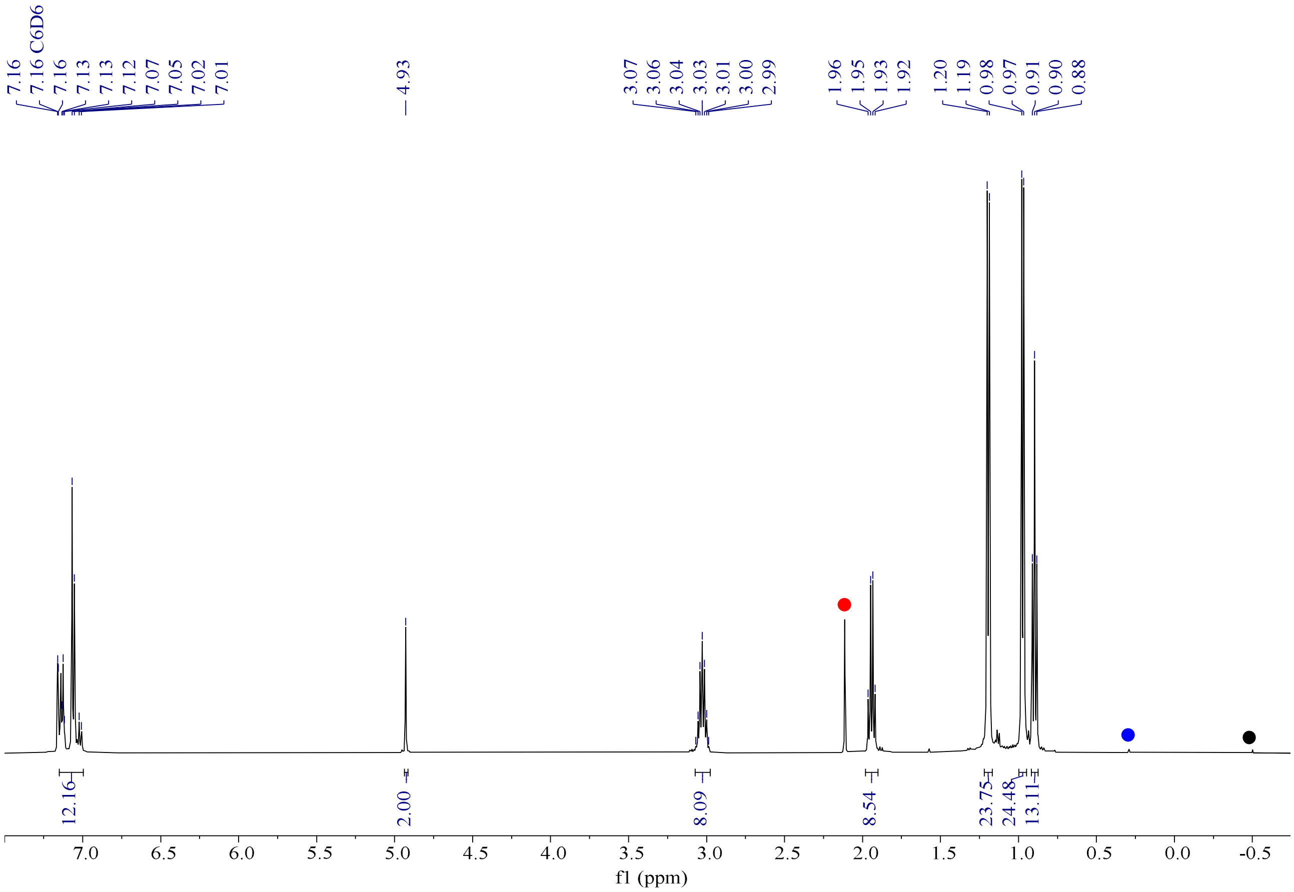

Figure S56. ^{1}H NMR spectrum (499.9 MHz, C_6D_6, 298 K) of *in-situ* generated [{(^{EtDip}nacnac)Mg}$_2$(μ-O)] **1b**. The blue circle denotes the resonance associated with silicone grease. The red circle denotes the resonance associated with toluene (Ph-CH_3). The black circle denotes the resonance associated with Mg-(OH)$_2$ of [{(^{EtDip}nacnac)Mg(μ-OH)}$_2$] **3b**.

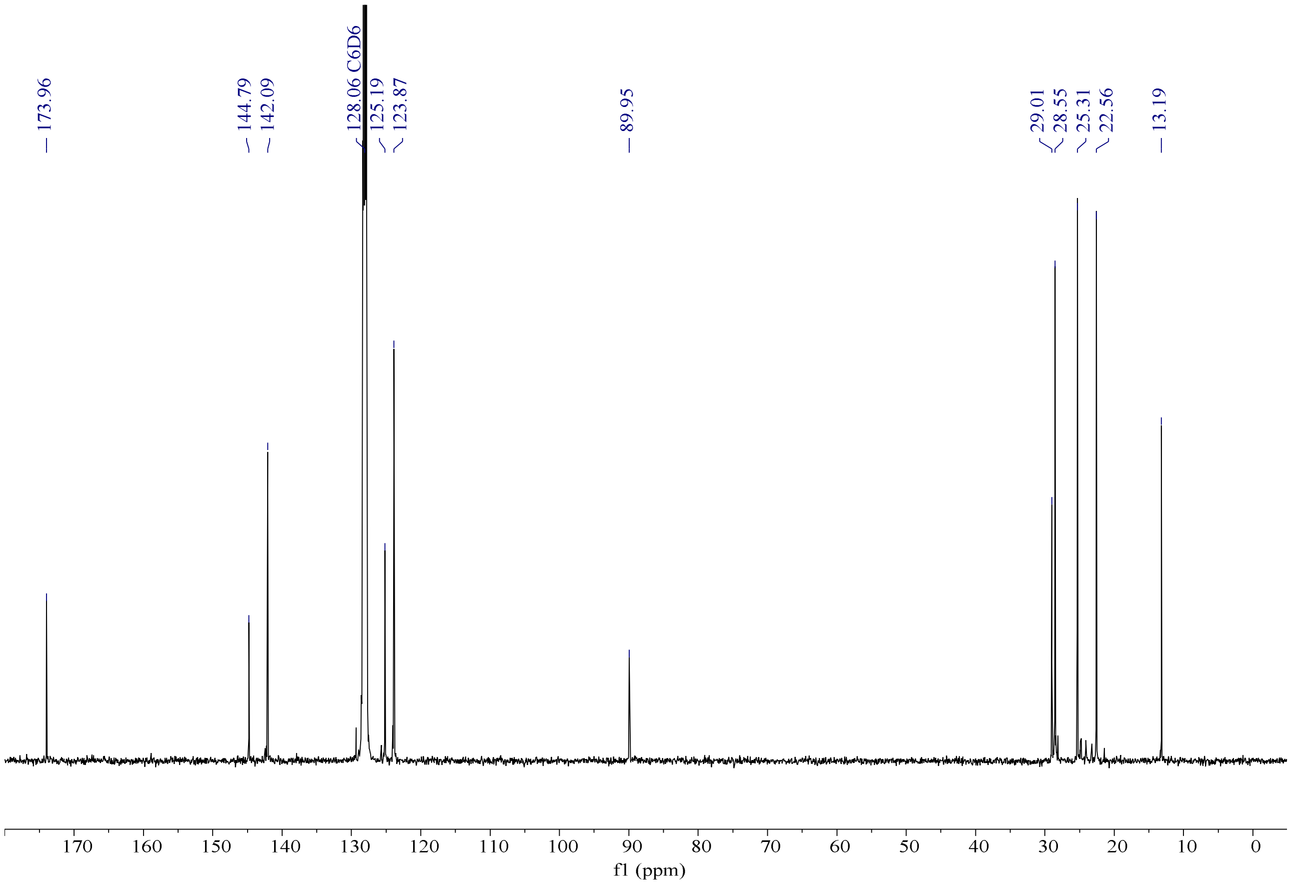

Figure S57. $^{13}C\{^{1}H\}$ NMR spectrum (125.7 MHz, C_6D_6, 298 K) of *in-situ* generated $[\{(^{EtDip}nacnac)Mg\}_2(\mu\text{-}O)]$ **1b**.

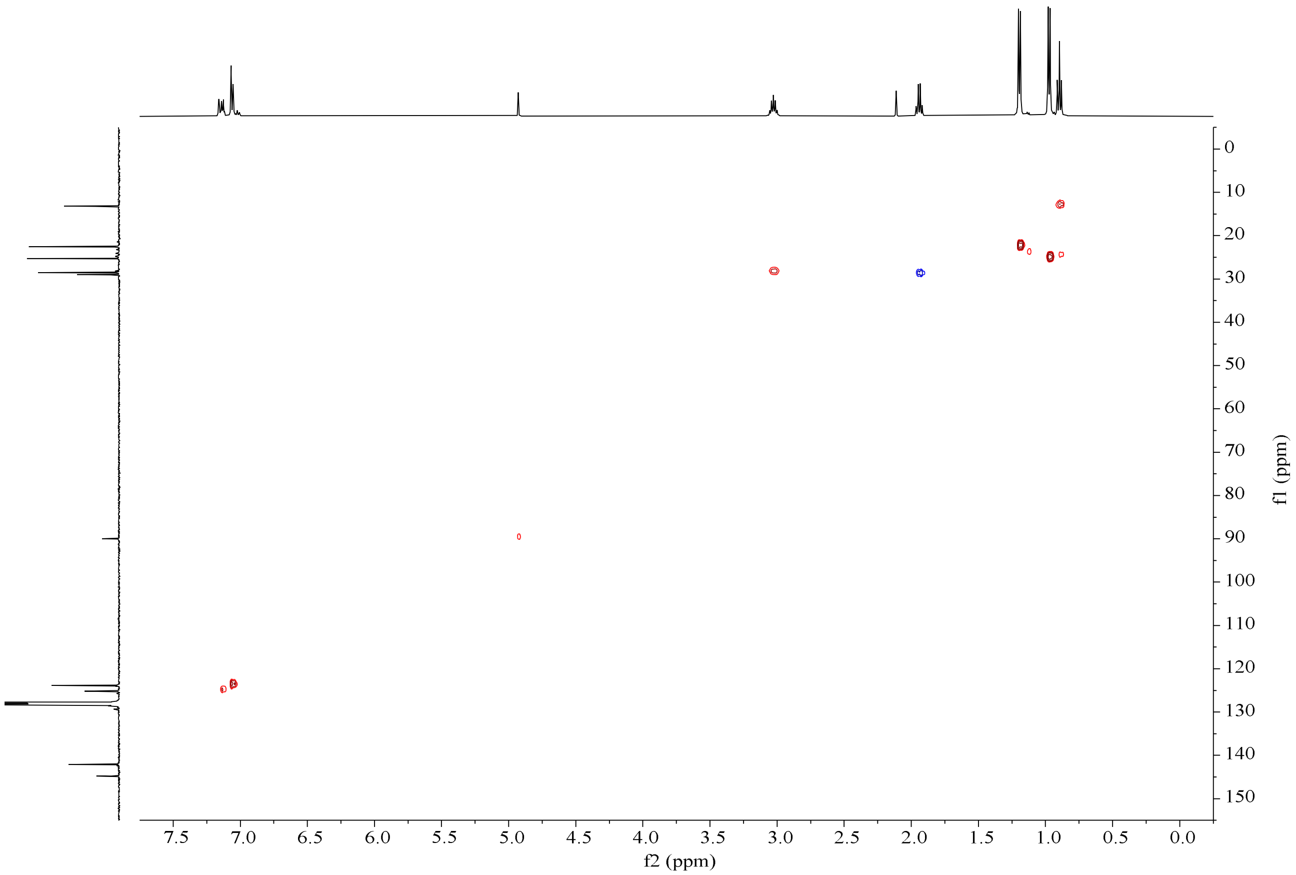

Figure S58. ^1H-^{13}C HSQC NMR spectrum of *in-situ* generated [{(^{EtDip}nacnac)Mg}$_2$(μ-O)] **1b**.

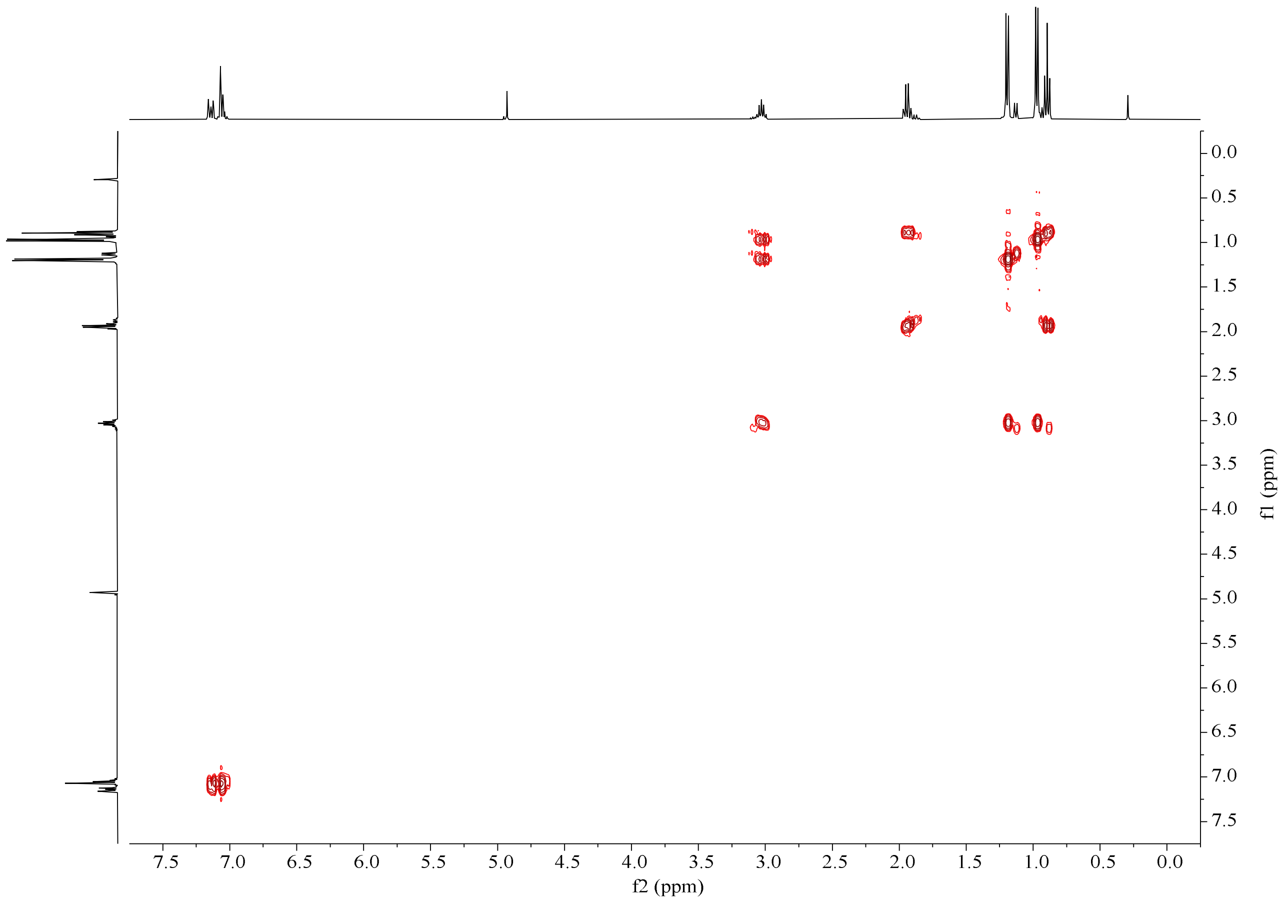

Figure S59. ^{1}H-^{1}H COSY NMR spectrum of *in-situ* generated [{(^{EtDip}nacnac)Mg}$_2$(μ-O)] **1b**.

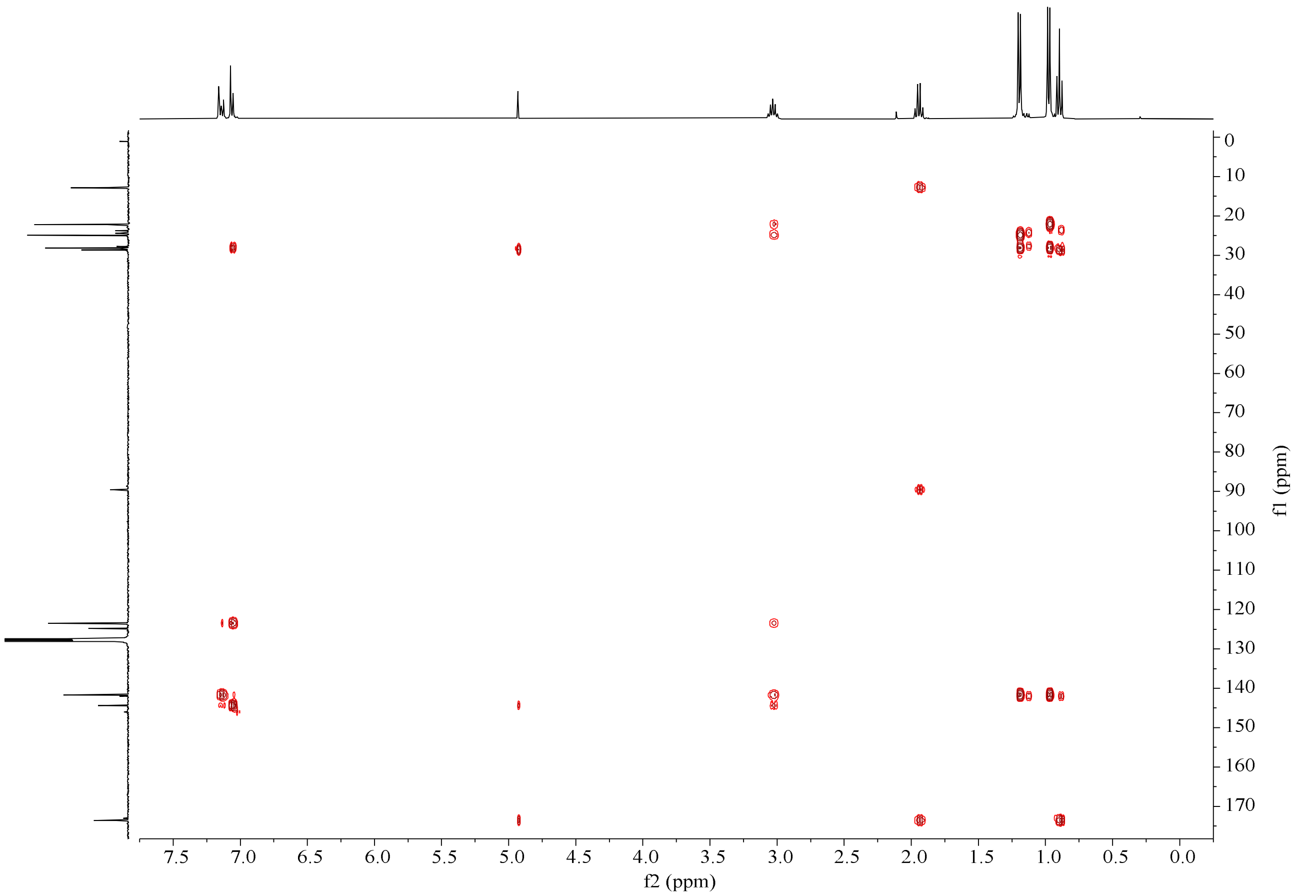

Figure S60. ^{1}H-^{13}C HMBC NMR spectrum of *in-situ* generated [{(^{EtDip}nacnac)Mg}$_2$(μ-O)] **1b**.

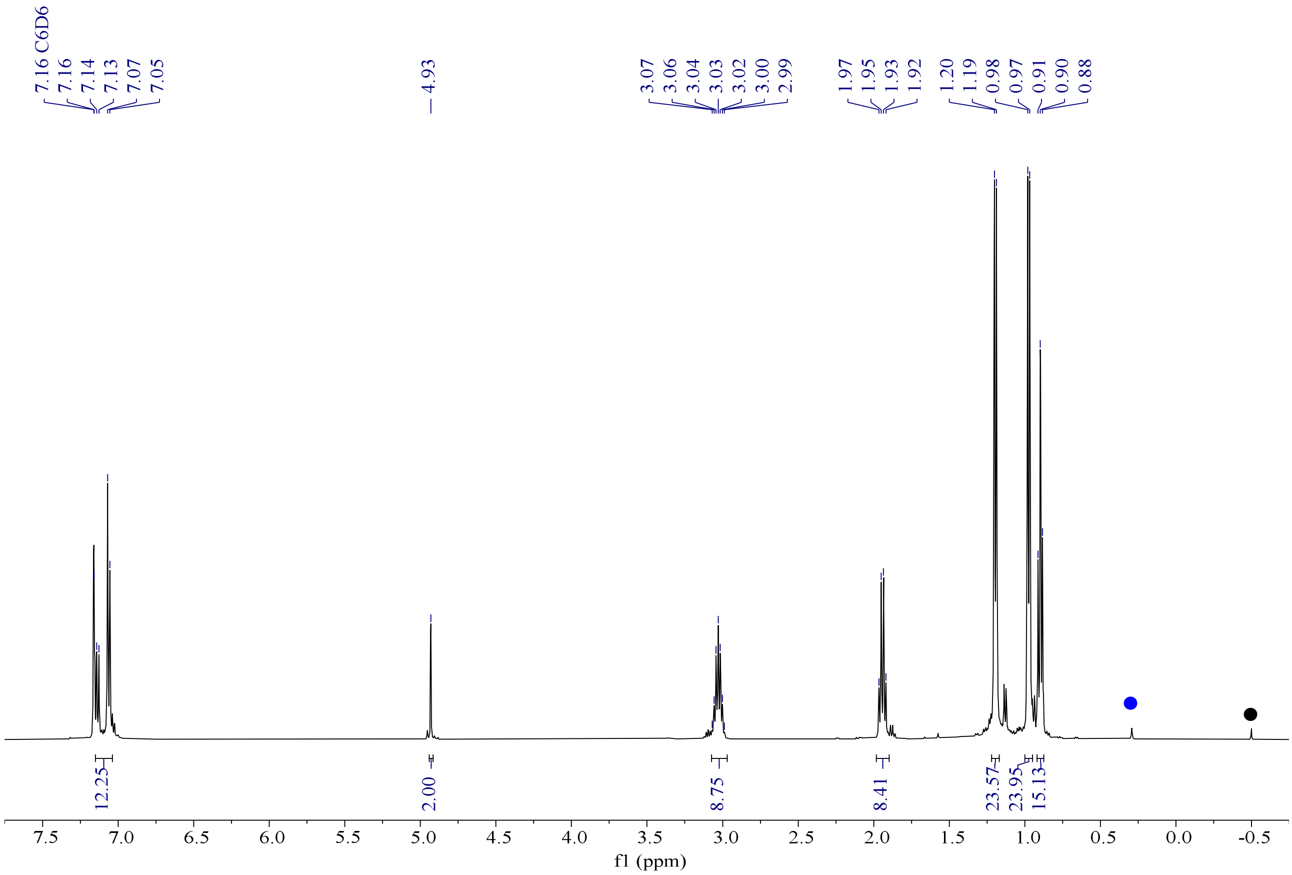

Figure S61. ^{1}H NMR spectrum (500.1 MHz, C_6D_6, 298 K) of isolated [{(^{EtDip}nacnac)Mg}$_2$(μ-O)] **1b**. The blue circle denotes the resonance associated with silicone grease. The black circle denotes the resonance associated with Mg-(O*H*)$_2$ of [{(^{EtDip}nacnac)Mg(μ-OH)}$_2$] **3b**.

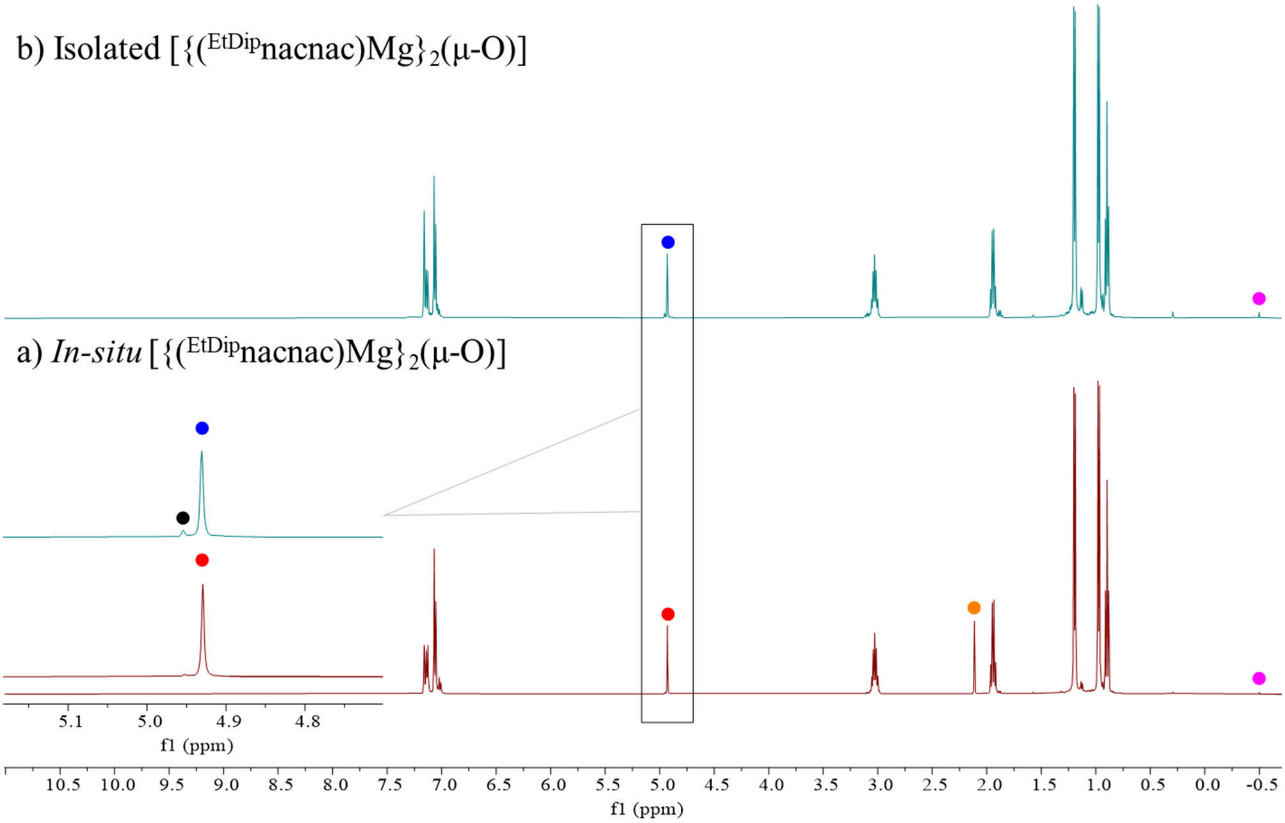

Figure S62. Stacked ^{1}H NMR spectra of *in-situ* generated [{(^{EtDip}nacnac)Mg}$_2$(μ-O)] **1b** and isolated [{(^{EtDip}nacnac)Mg}$_2$(μ-O)] **1b**. The orange circle denotes the resonance associated with toluene (Ph-CH_3). The red circle denotes the resonance associated with the backbone-C*H* of *in-situ* generated [{(^{EtDip}nacnac)Mg}$_2$(μ-O)] **1b**. The blue circle denotes the resonance associated with the backbone-C*H* of isolated [{(^{EtDip}nacnac)Mg}$_2$(μ-O)] **1b**. The black circle denotes the resonance associated with the backbone-C*H* of [{(^{EtDip}nacnac)Mg(μ-OH)}$_2$] **1b**. The pink circle denotes the resonance associated with Mg-(O*H*)$_2$ of [{(^{EtDip}nacnac)Mg(μ-OH)}$_2$] **3b**. Spectrum a) 499.9 MHz, C_6D_6, 298 K. Spectrum b) 500.1 MHz, C_6D_6, 298 K.

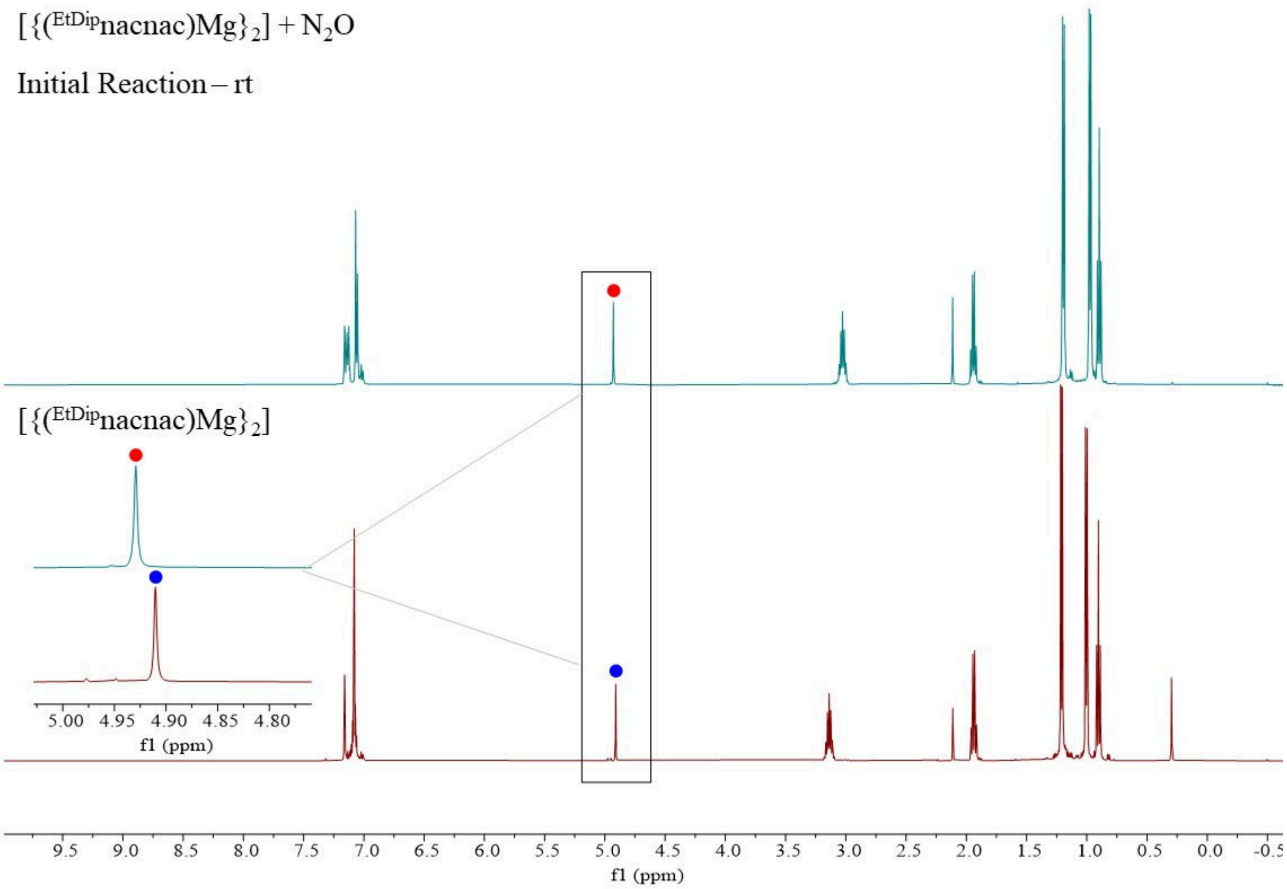

Figure S63. Stacked ^{1}H NMR spectra (499.9 MHz, C_6D_6, 298 K) of the reaction of a yellow solution of [{(^{EtDip}nacnac)Mg}$_2$] **2b** (10.0 mg, 10.6 μmol) and nitrous oxide (ca. 1 bar) in C_6D_6 (0.5 mL) at room temperature (rt), top. A reference spectrum of [{(^{EtDip}nacnac)Mg}$_2$] **2b**, bottom, is shown for comparison. The blue circle denotes the resonance associated with the backbone-C*H* of [{(^{EtDip}nacnac)Mg}$_2$] **2b**. The red circle denotes the resonance associated with the backbone-C*H* of [{(^{EtDip}nacnac)Mg}$_2$(μ-O)] **1b**.

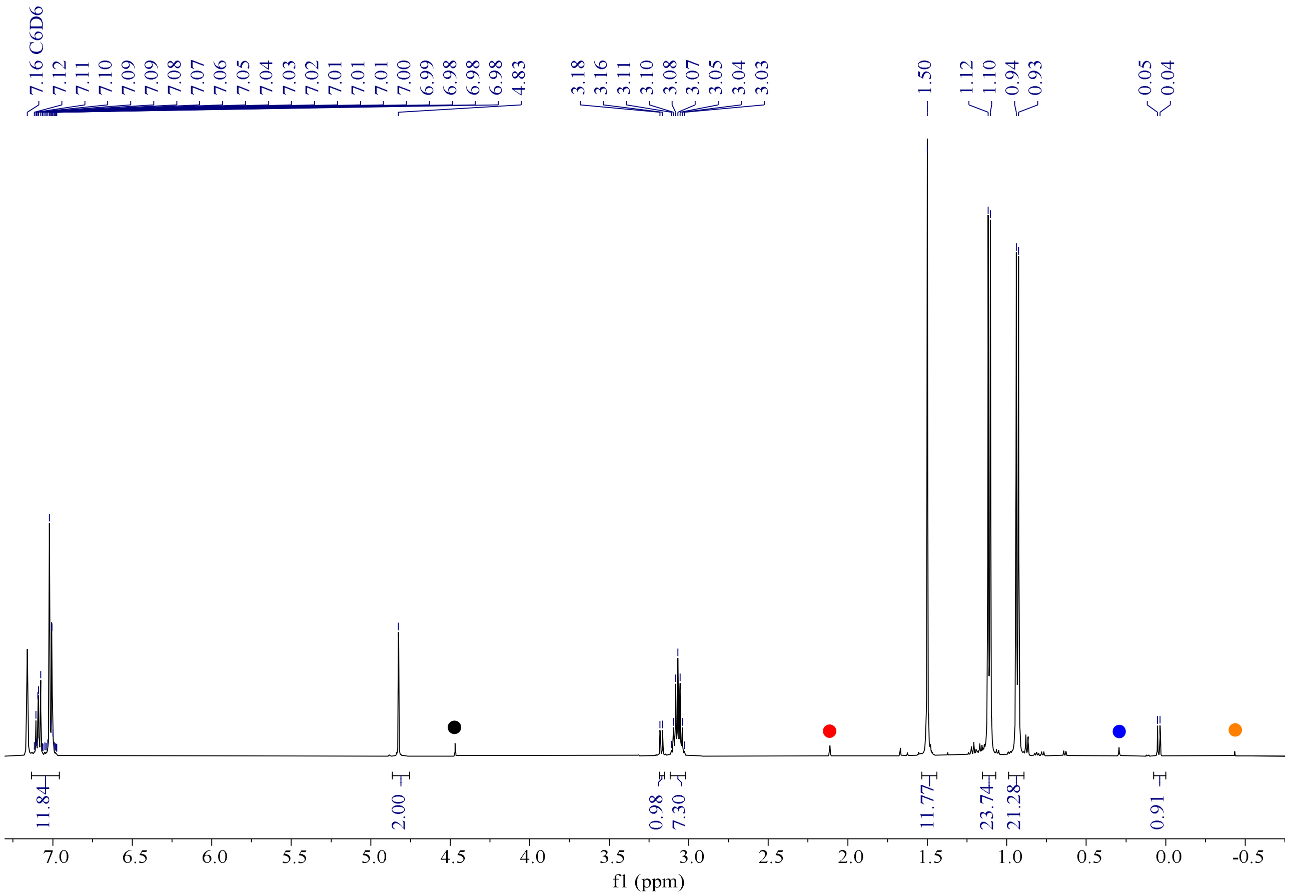

Figure S64. ^{1}H NMR spectrum (499.9 MHz, C_6D_6, 298 K) of *in-situ* generated [{(^{MeDip}nacnac)Mg}$_2$(μ-H)(μ-OH)] **4a**. The black circle denotes the resonance associated with dihydrogen. The red circle denotes the resonance associated with toluene (Ph-CH_3). The blue circle denotes the resonance associated with silicone grease. The orange circle denotes the resonance associated with Mg-(OH)$_2$ of [{(^{MeDip}nacnac)Mg(μ-OH)}$_2$] **3a.**

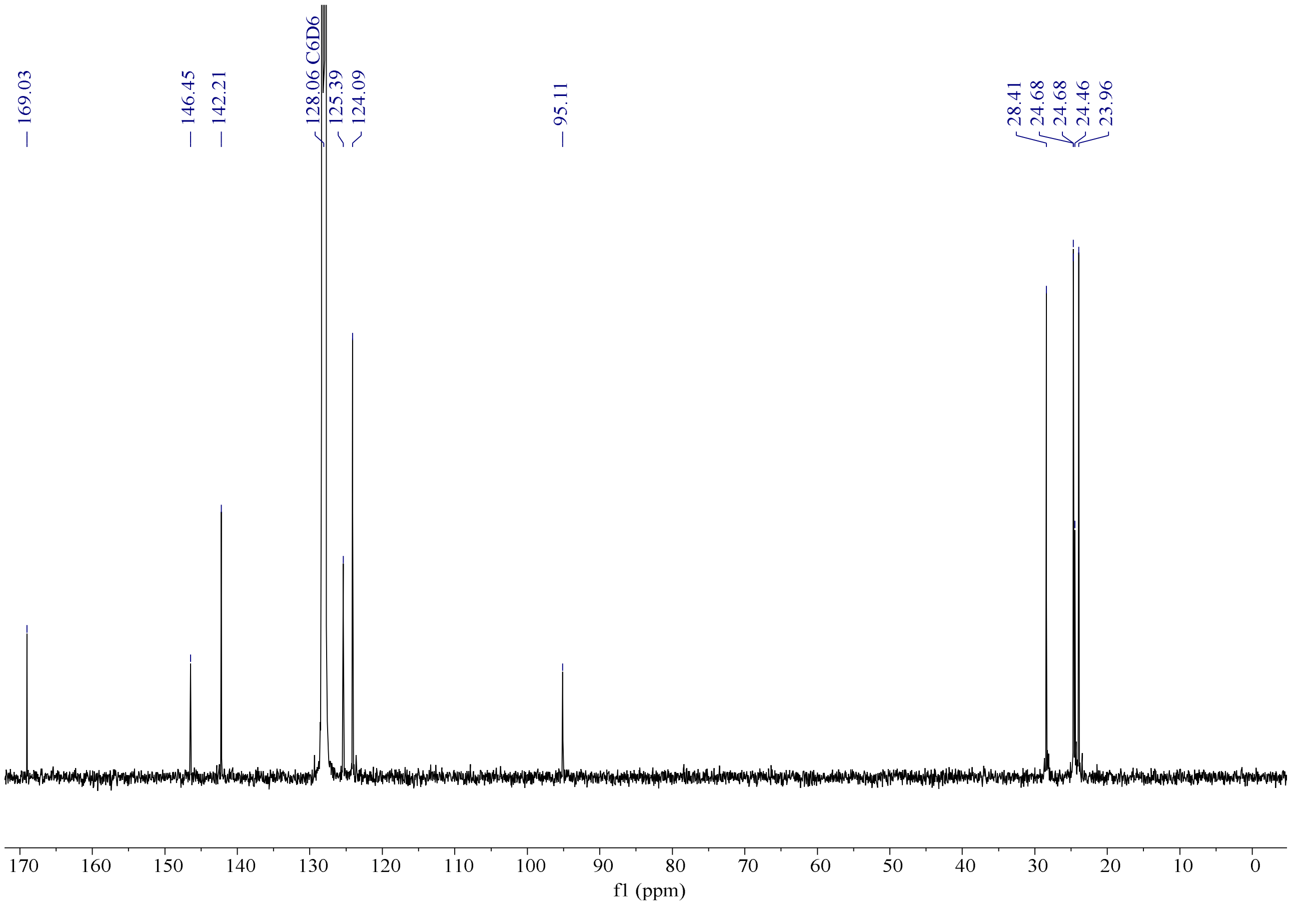

Figure S65. $^{13}C\{^{1}H\}$ NMR spectrum (125.7 MHz, C_6D_6, 298 K) of *in-situ* generated [{(^{MeDip}nacnac)Mg}$_2$(μ-H)(μ-OH)] **4a**.

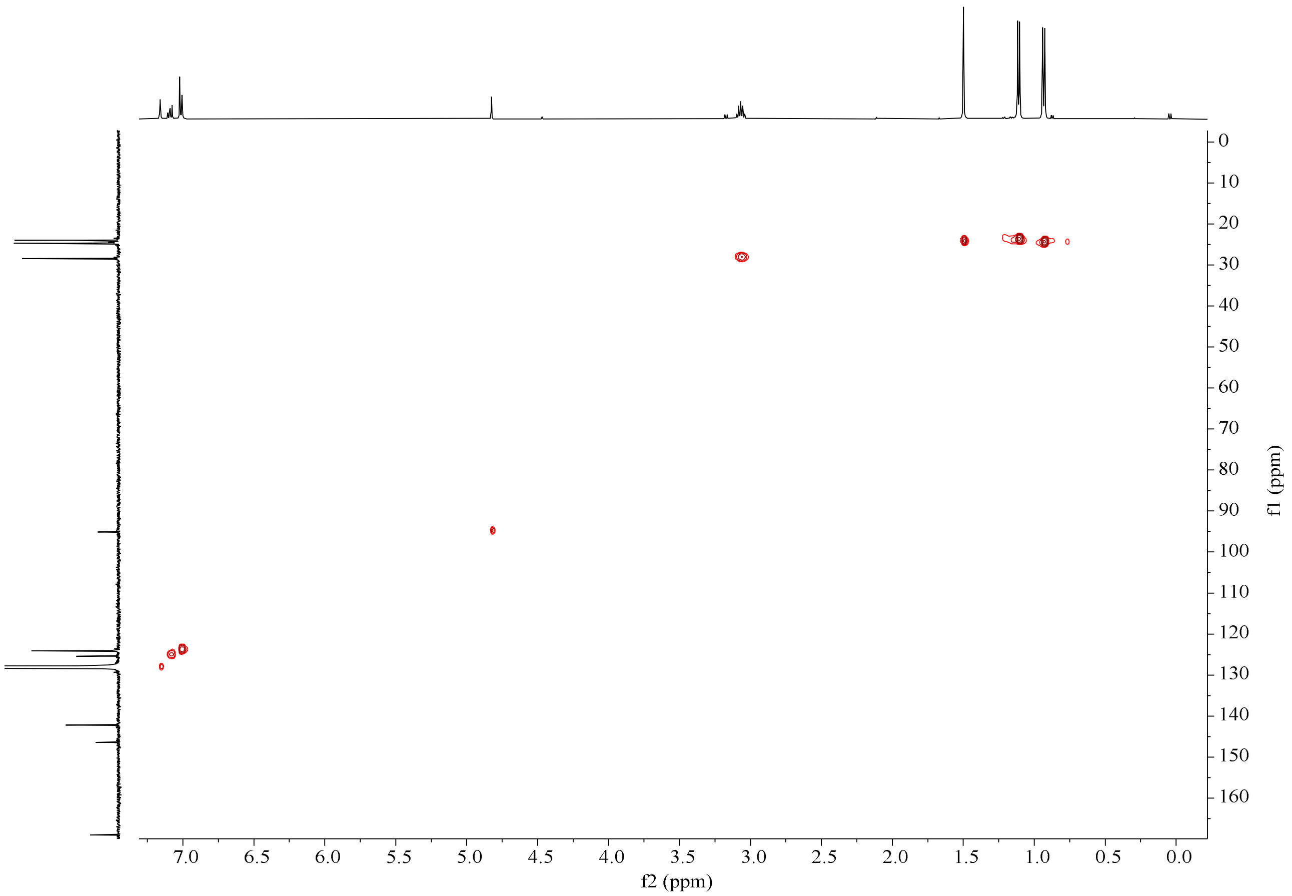

Figure S66. ^{1}H-^{13}C HSQC NMR spectrum of *in-situ* generated [{(^{MeDip}nacnac)Mg}$_2$(μ-H)(μ-OH)] **4a**.

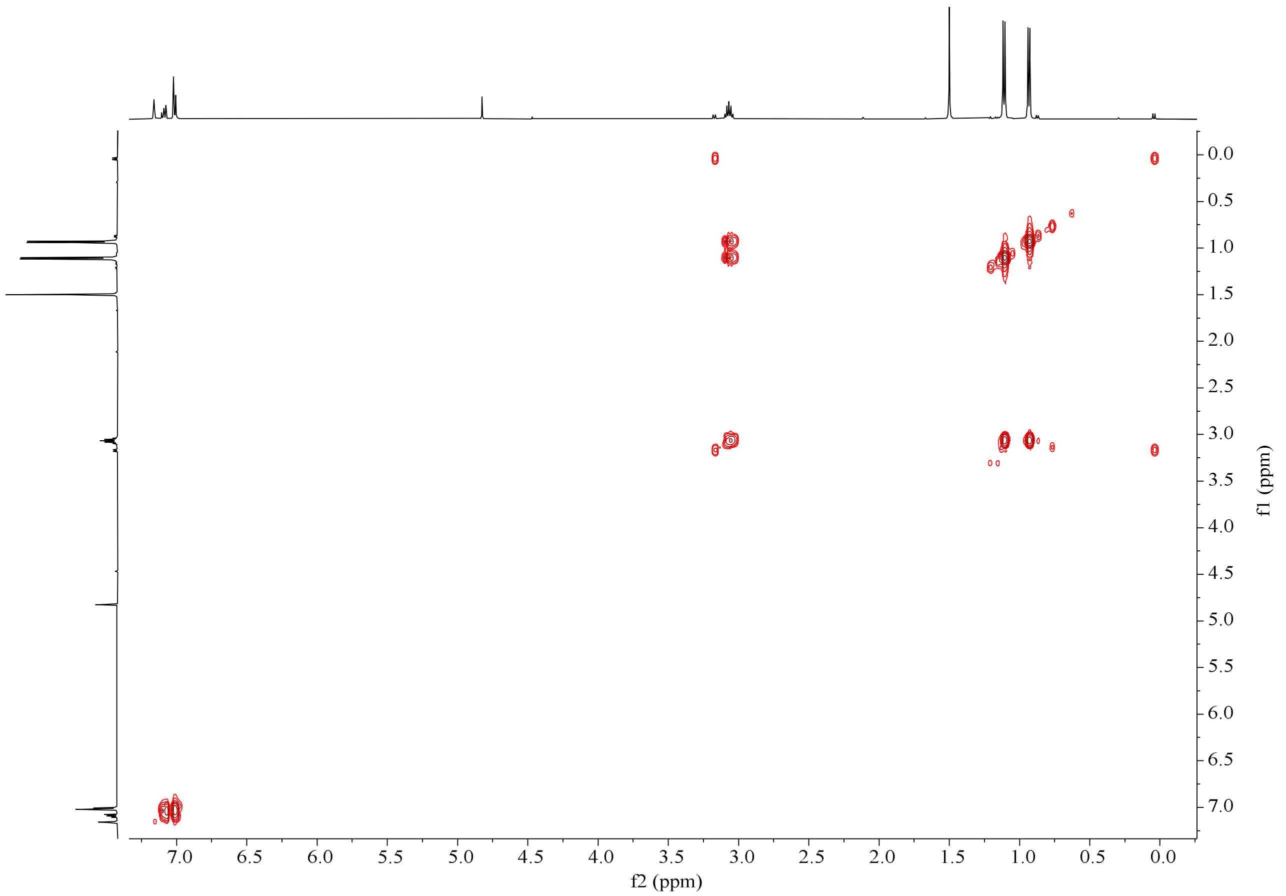

Figure S67. ^{1}H-^{1}H COSY NMR spectrum of *in-situ* generated [{(^{MeDip}nacnac)Mg}$_2$(μ-H)(μ-OH)] **4a**.

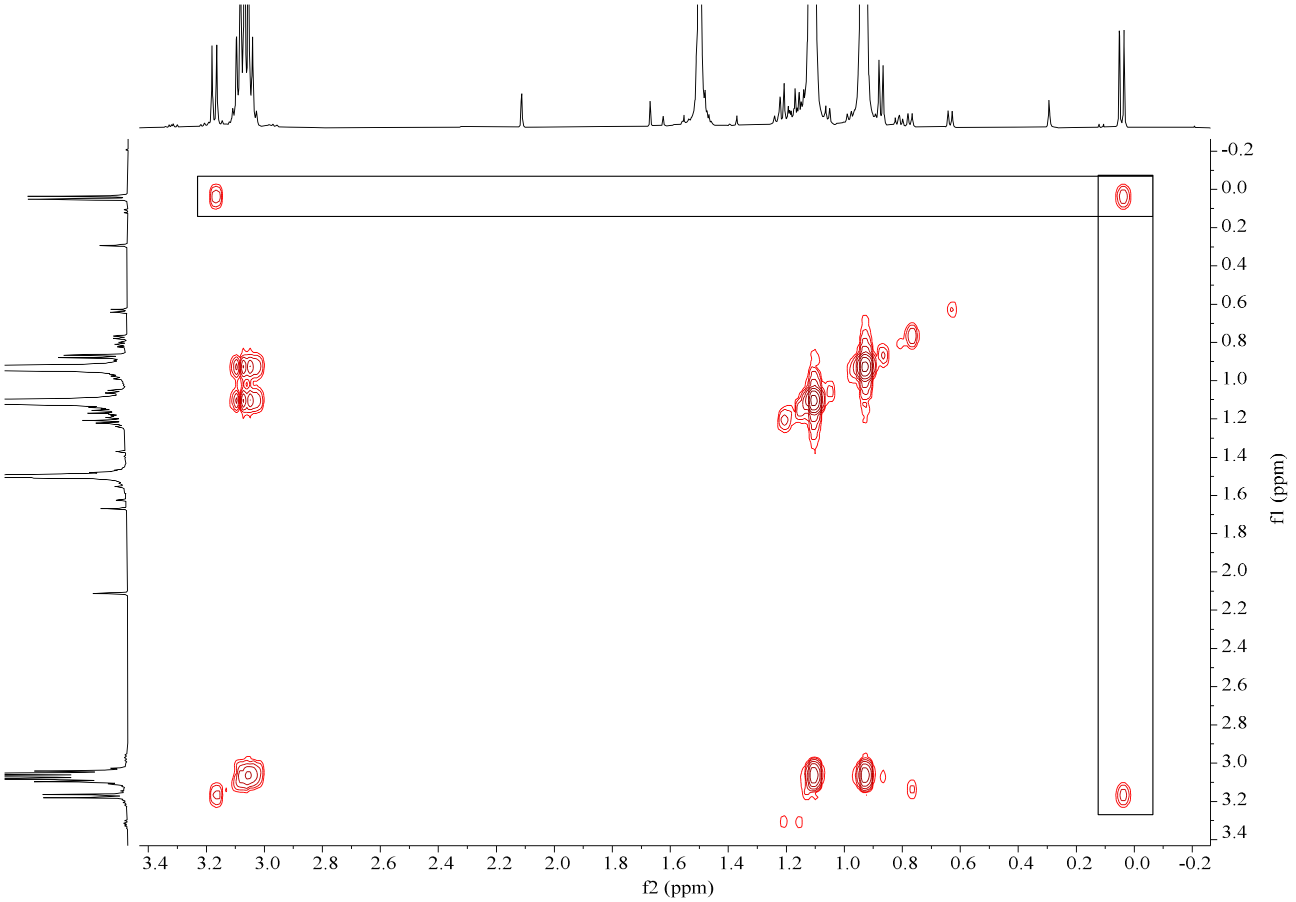

Figure S68. ^{1}H-^{1}H COSY NMR spectrum of *in-situ* generated [{(^{MeDip}nacnac)Mg}$_2$(μ-H)(μ-OH)] **4a** (chemical range: -0.2-3.4 ppm). The coupling between the hydroxide and hydride units is highlighted.

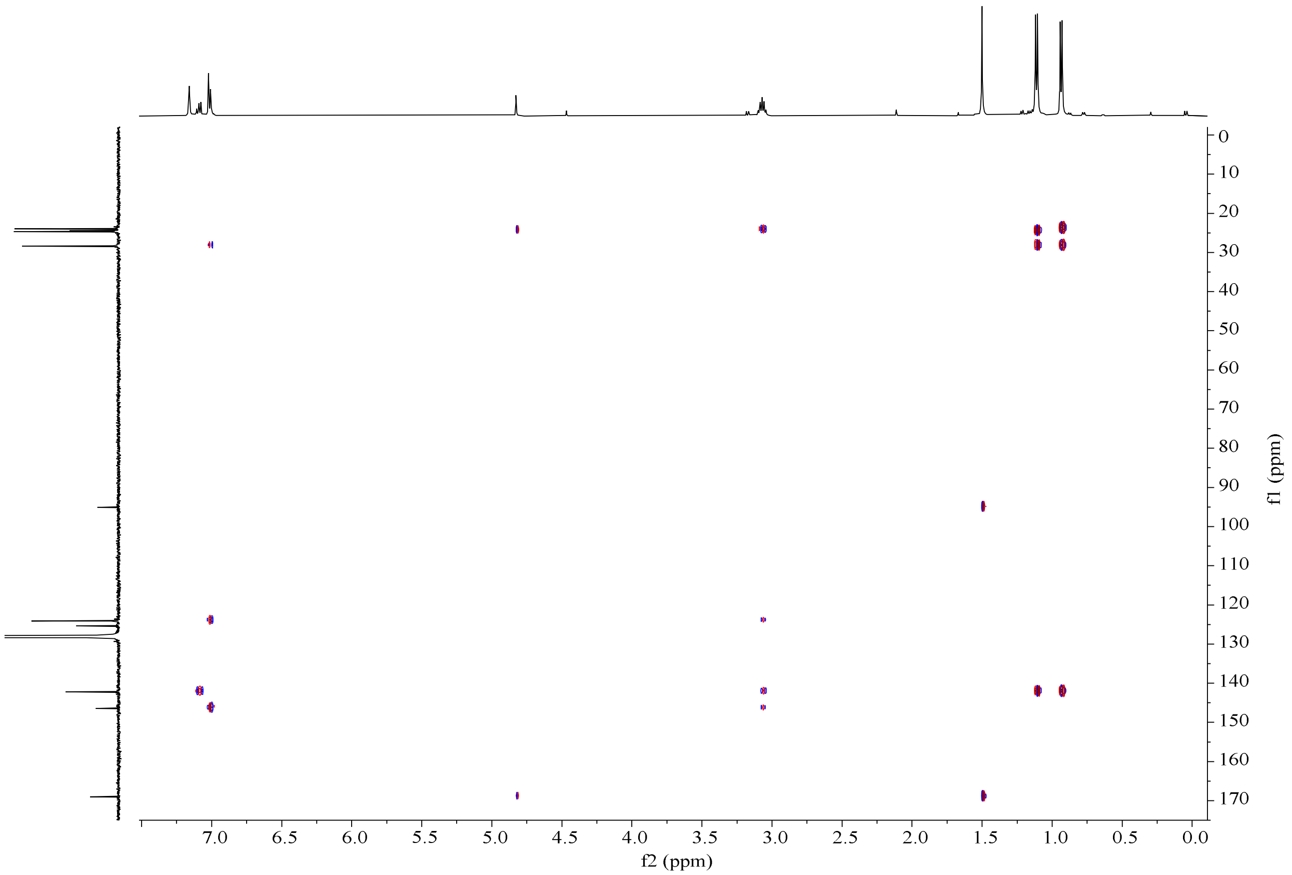

Figure S69. ^{1}H-^{13}C HMBC NMR spectrum of *in-situ* generated [{(^{MeDip}nacnac)Mg}$_2$(μ-H)(μ-OH)] **4a**.

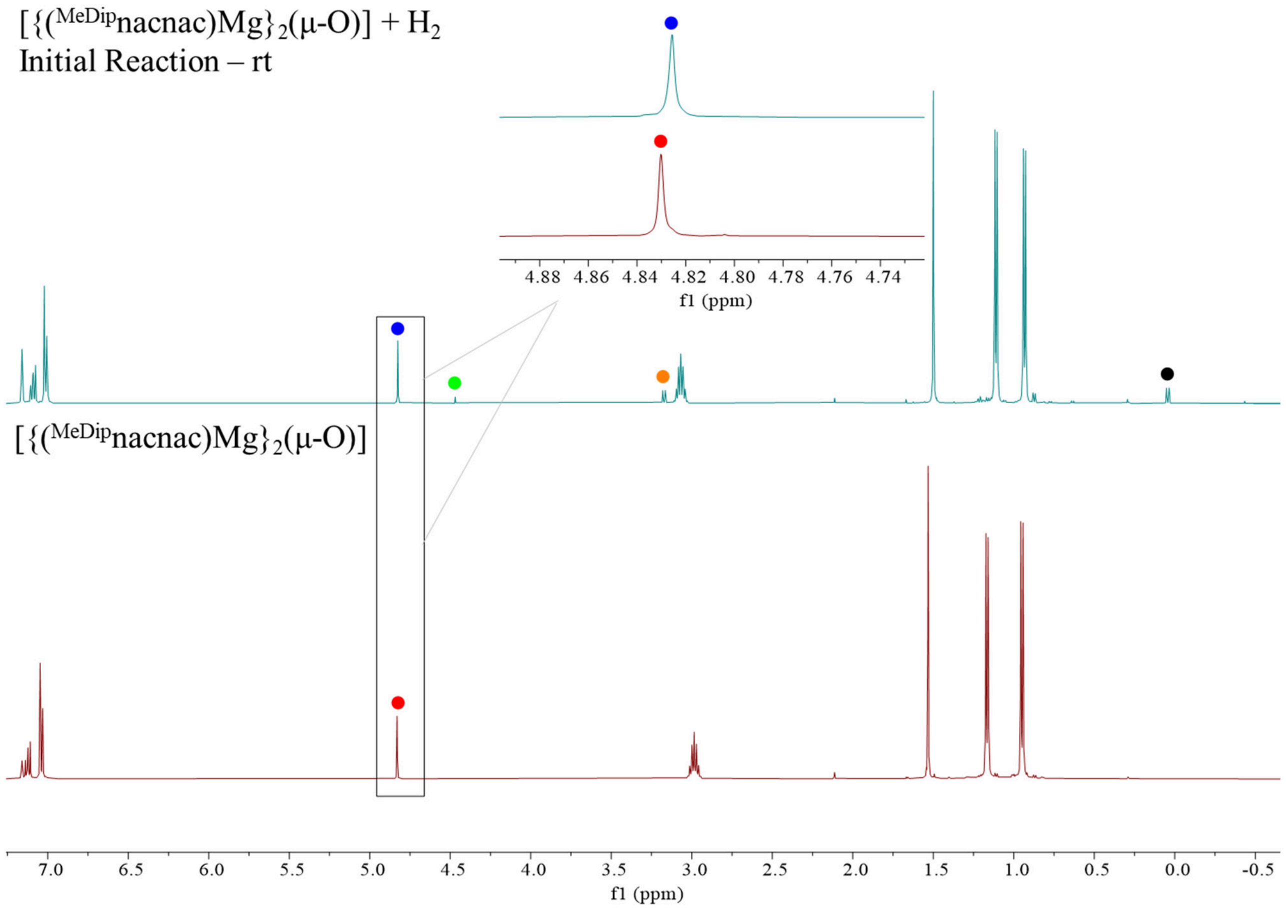

Figure S70. Stacked ^{1}H NMR spectra (499.9 MHz, C_6D_6, 298 K) of the reaction of a colourless *in-situ* generated solution of [{(^{MeDip}nacnac)Mg}$_2$(μ-O)] **1a** (18.4 mg, 20.4 μmol) and dihydrogen (ca. 1 bar) in C_6D_6 (0.6 mL) at room temperature (rt) in a J Young NMR tube. The green circle denotes the resonance associated with dihydrogen. The red circle denotes the resonance associated with the backbone-C*H* of [{(^{MeDip}nacnac)Mg}$_2$(μ-O)] **1a**. The blue circle denotes the resonance associated with the backbone-C*H* of [{(^{MeDip}nacnac)Mg}$_2$(μ-H)(μ-OH)] **4a**. The orange circle denotes the resonance associated with Mg-*H* of [{(^{MeDip}nacnac)Mg}$_2$(μ-H)(μ-OH)] **4a**. The black circle denotes the resonance associated with Mg-O*H* of [{(^{MeDip}nacnac)Mg}$_2$(μ-H)(μ-OH)] **4a**.

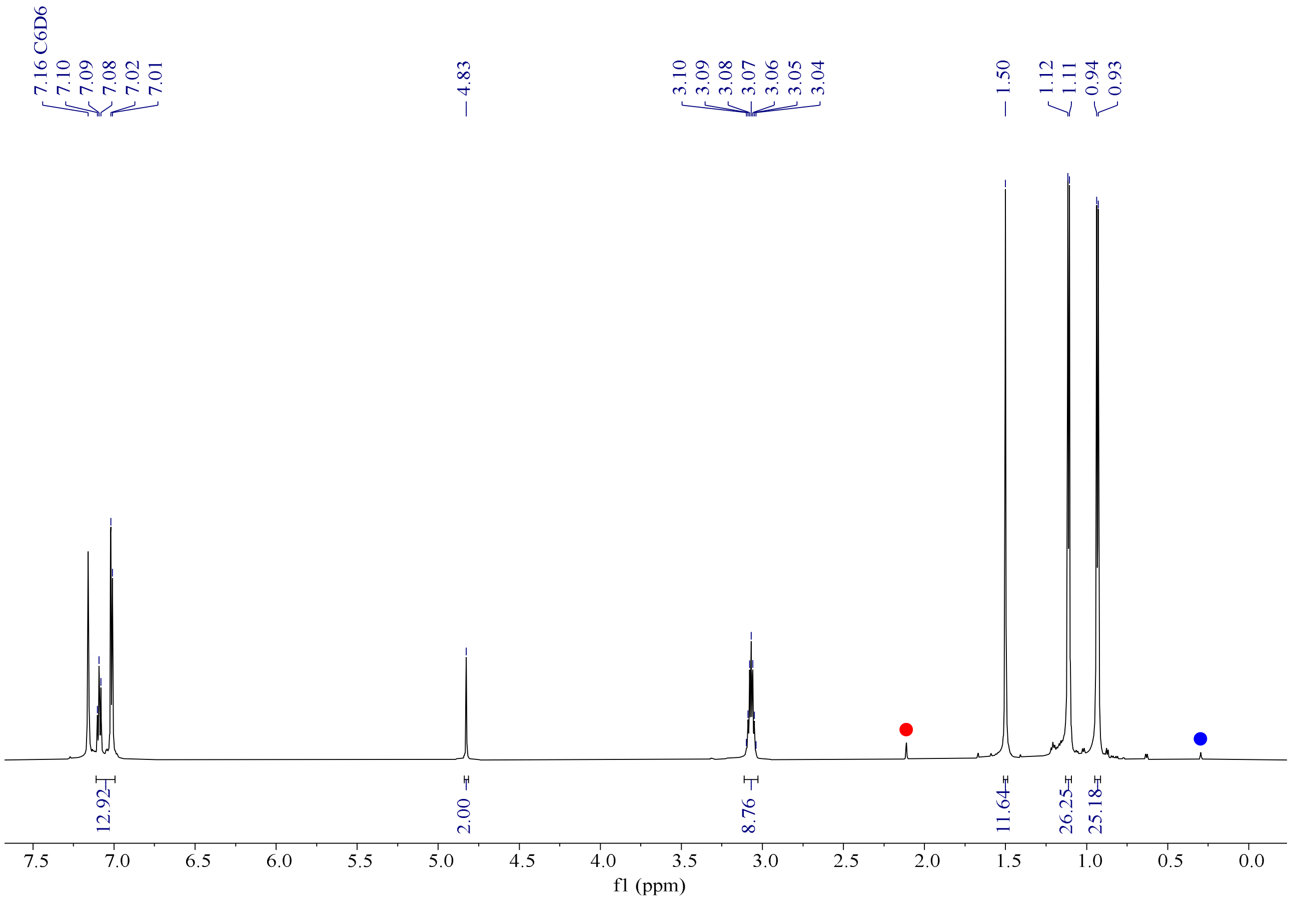

Figure S71. ^{1}H NMR spectrum (700.0 MHz, C_6D_6, 295 K) of *in-situ* generated [{(^{MeDip}nacnac)Mg}$_2$(μ-D)(μ-OD)] **4a-*d*$_2$**. The blue circle denotes the resonance associated with silicone grease. The red circle denotes the resonance associated with toluene (Ph-CH_3).

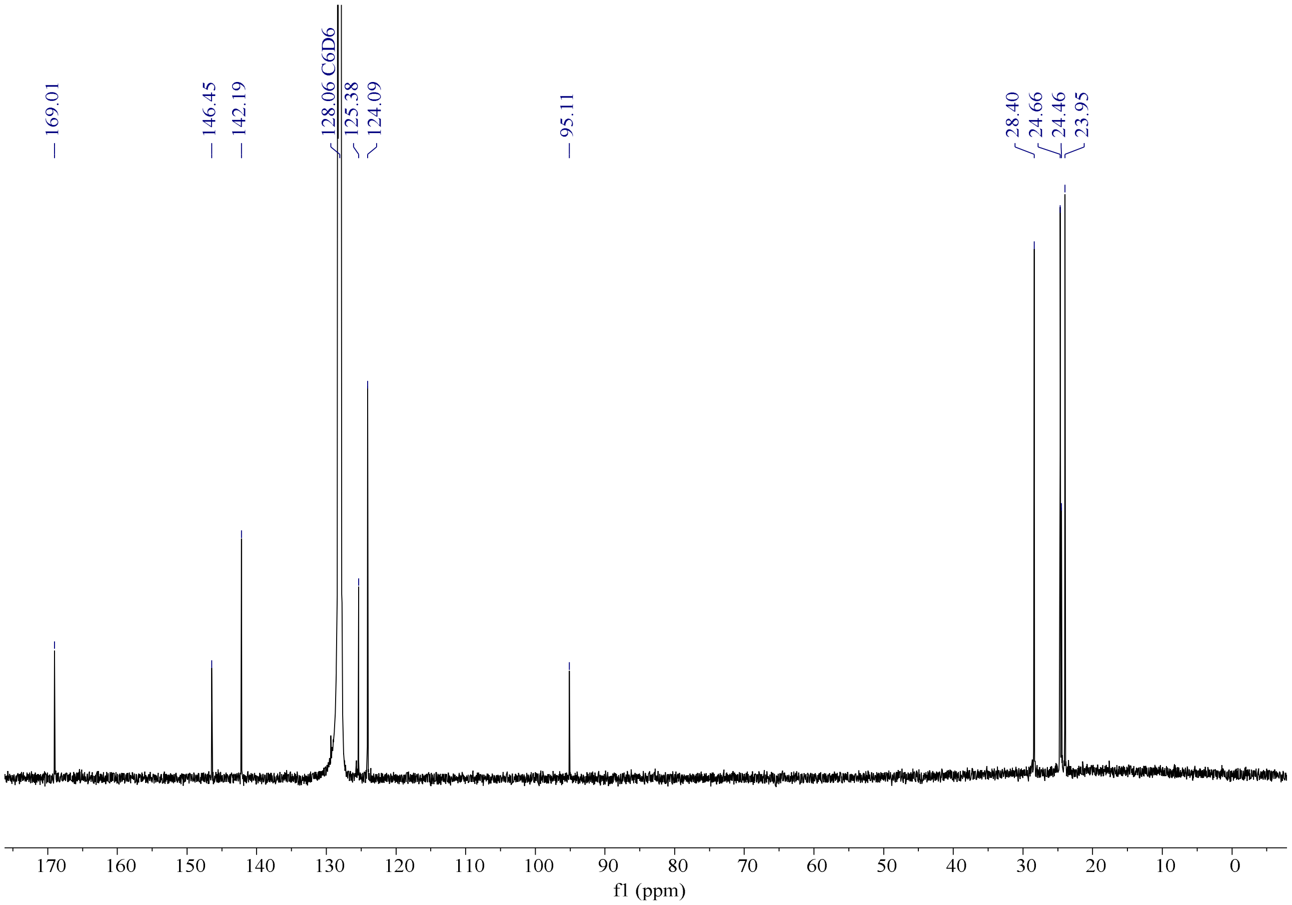

Figure S72. $^{13}C\{^1H\}$ NMR spectrum (176.0 MHz, C_6D_6, 295 K) of *in-situ* generated [{(^{MeDip}nacnac)Mg}$_2$(μ-D)(μ-OD)] **4a-*d*$_2$**.

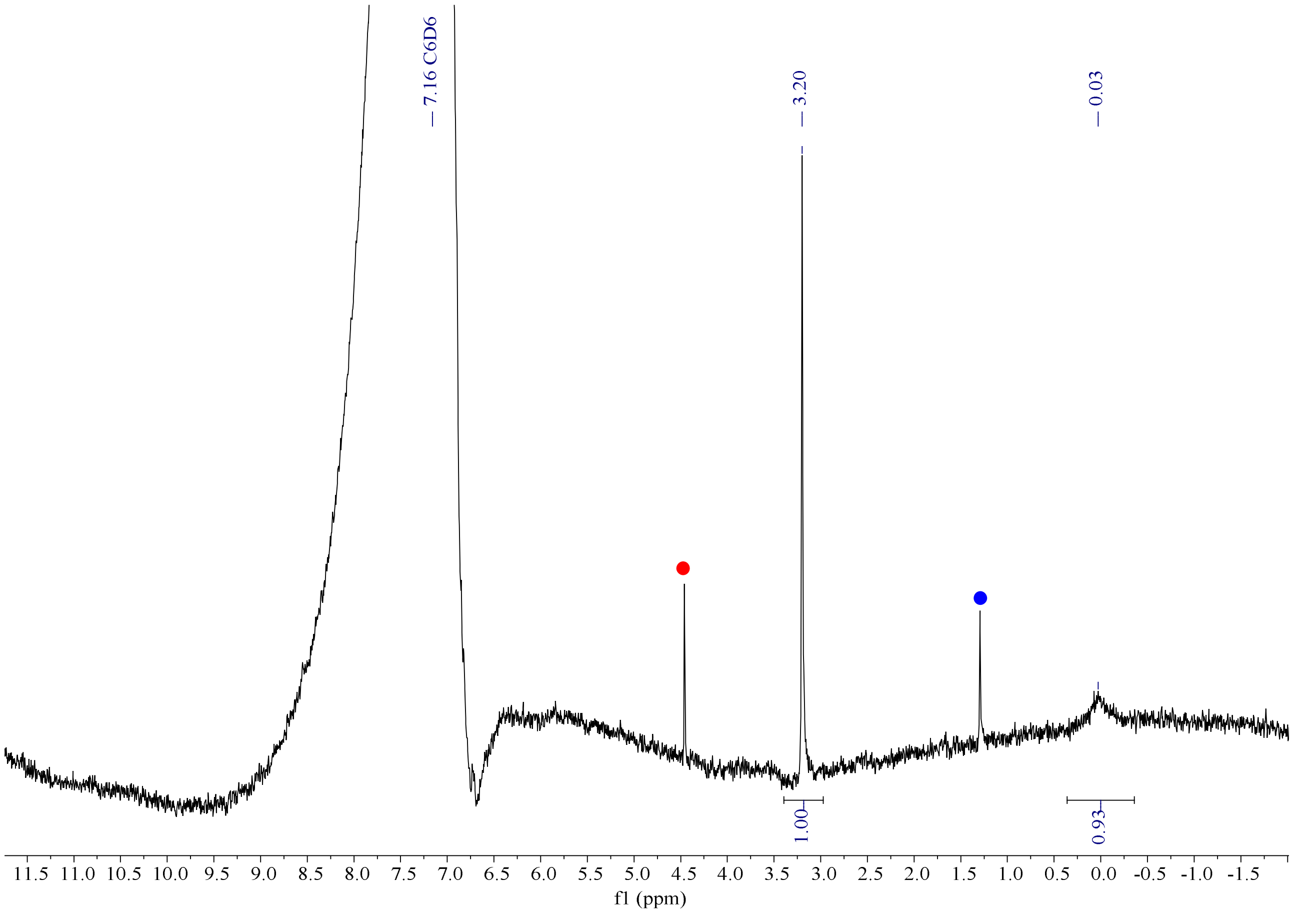

Figure S73. ^{2}H NMR spectrum (107.5 MHz, C_6D_6, 295 K) of *in-situ* generated [{(^{MeDip}nacnac)Mg}$_2$(μ-D)(μ-OD)] **4a-*d*$_2$**. The blue circle denotes the resonance associated with an unknown impurity (possibly deuterated cyclohexane). The red circle denotes the resonance associated with dideuterium.

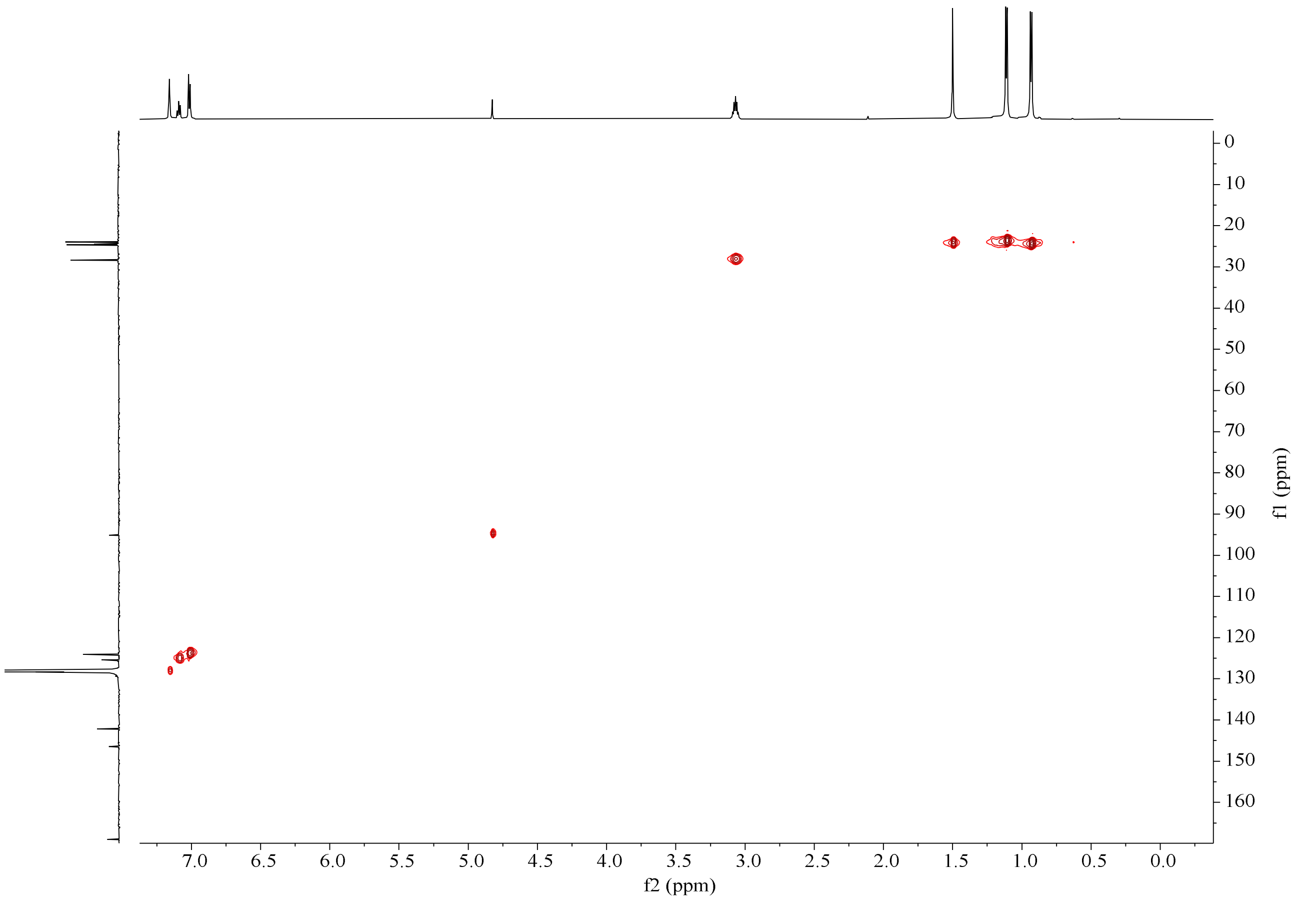

Figure S74. ^{1}H-^{13}C HSQC NMR spectrum of *in-situ* generated [{(^{MeDip}nacnac)Mg}$_2$(μ-D)(μ-OD)] **4a-*d*$_2$**.

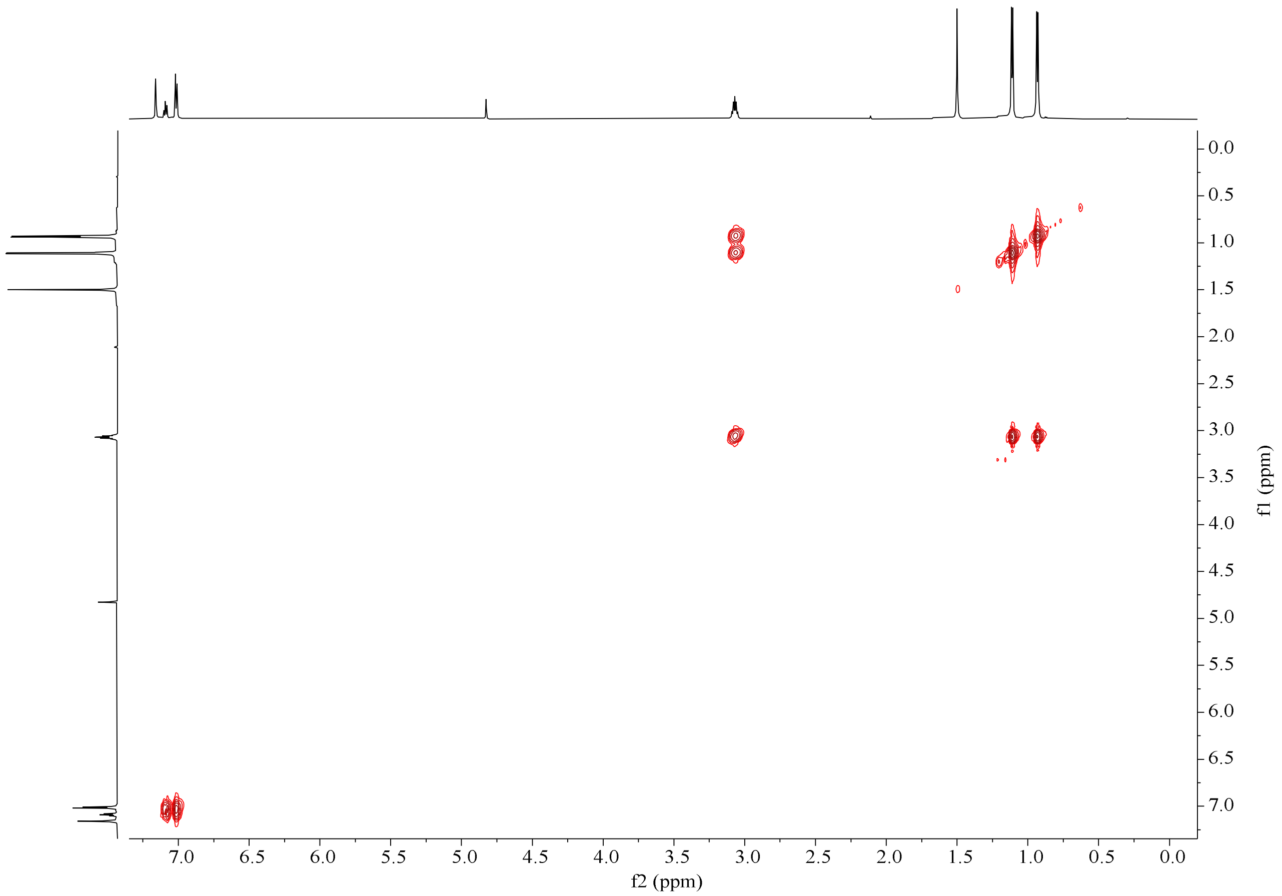

Figure S75. ^{1}H-^{1}H COSY NMR spectrum of *in-situ* generated [{(^{MeDip}nacnac)Mg}$_2$(μ-D)(μ-OD)] **4a-*d*$_2$**.

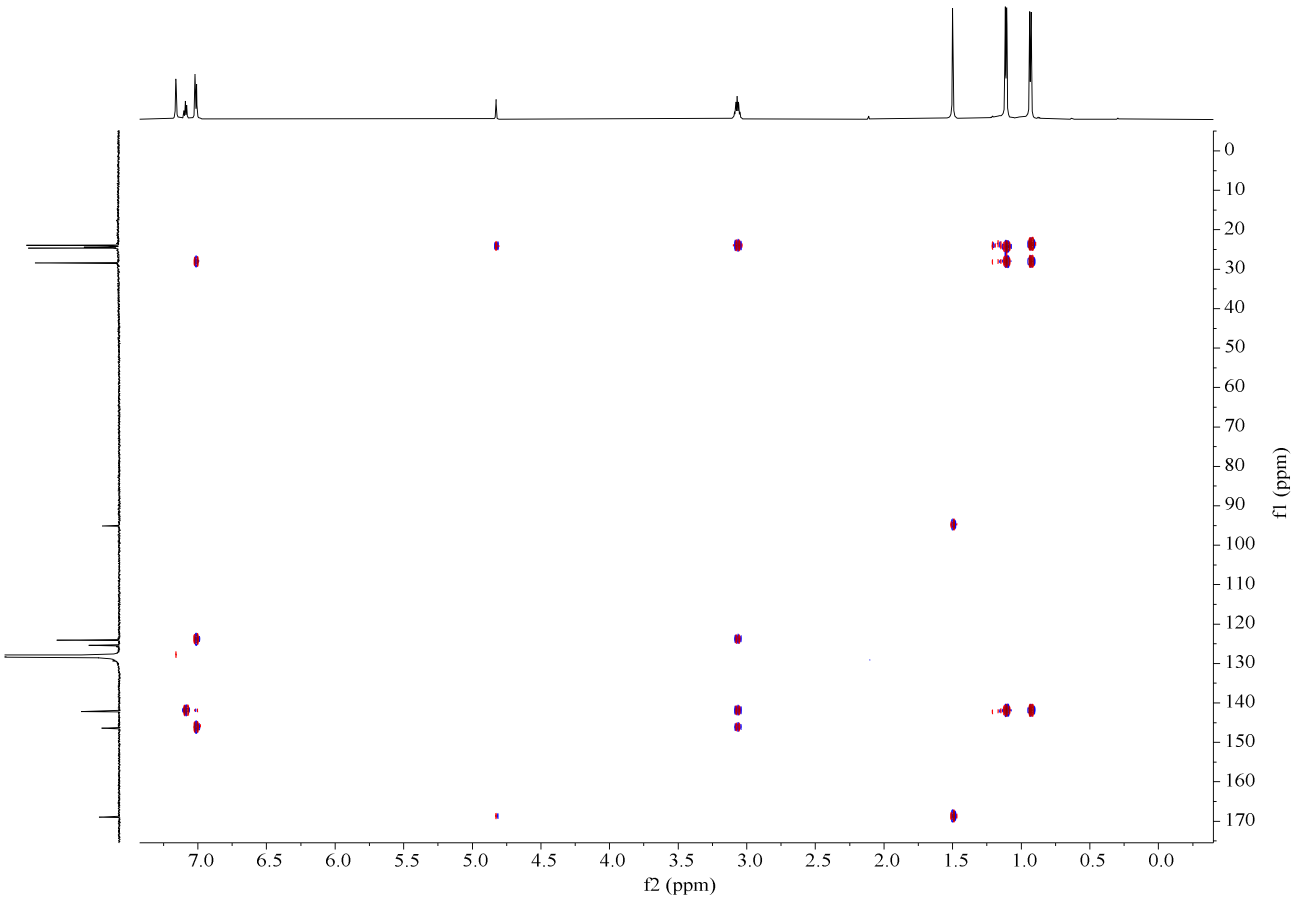

Figure S76. ^{1}H-^{13}C HMBC NMR spectrum of *in-situ* generated [{(^{MeDip}nacnac)Mg}$_2$(μ-D)(μ-OD)] **4a-*d*$_2$**.

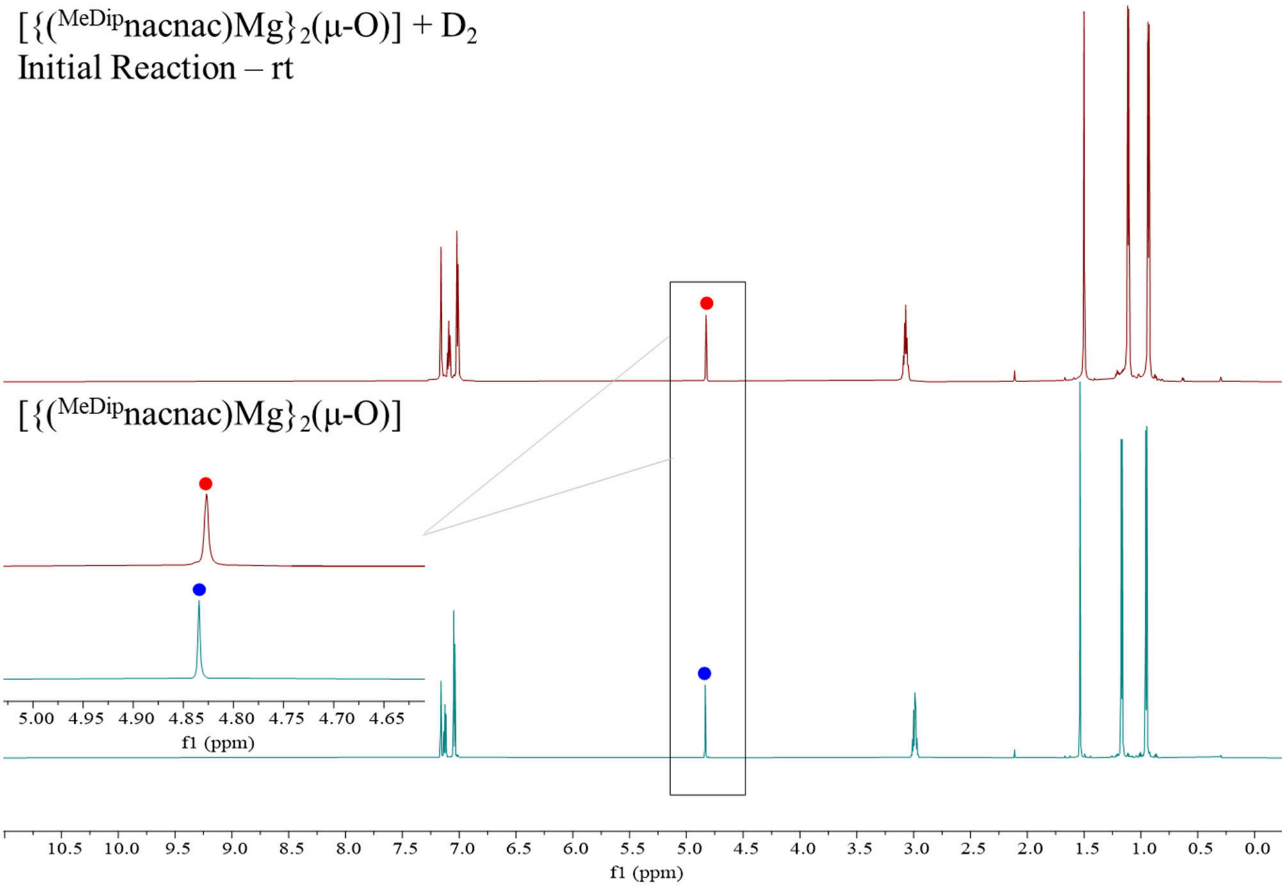

Figure S77. Stacked ^{1}H NMR spectra (700.0 MHz, C_6D_6, 295 K) of the reaction of a colourless *in-situ* generated solution of [{(^{MeDip}nacnac)Mg}$_2$(μ-O)] **1a** (10.7 mg, 11.9 μmol) in C_6D_6 (0.5 mL) with dideuterium (ca. 1 bar) in a J Young NMR tube at room temperature (rt). The blue circle denotes the resonance associated with the backbone-C*H* of [{(^{MeDip}nacnac)Mg}$_2$(μ-O)] **1a**. The red circle denotes the resonance associated with the backbone-C*H* of [{(^{MeDip}nacnac)Mg}$_2$(μ-D)(μ-OD)] **4a-*d*2**.

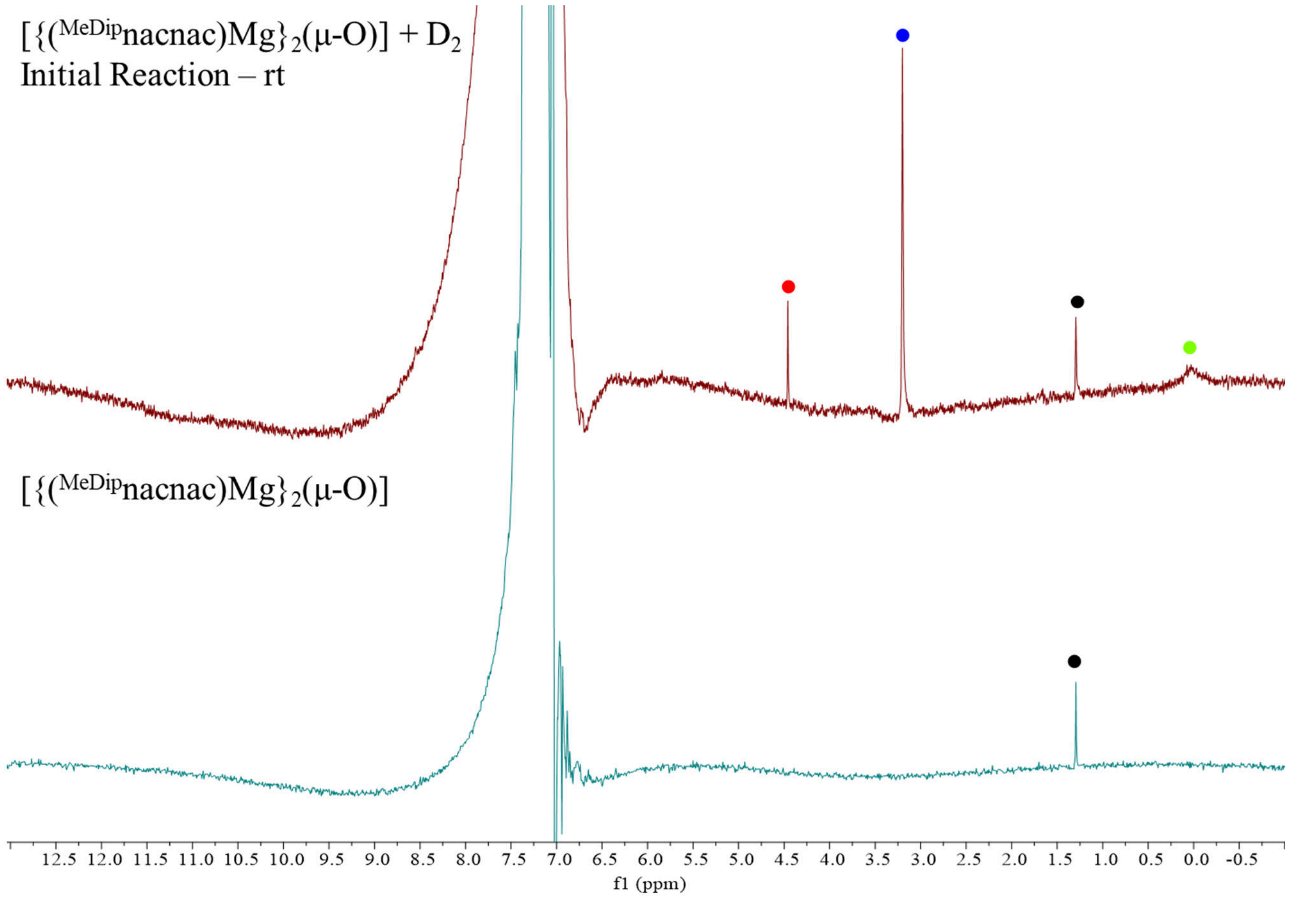

Figure S78. Stacked ^{2}H NMR spectra (107.5 MHz, C_6D_6, 295 K) of the reaction of a colourless *in-situ* generated solution of [{(^{MeDip}nacnac)Mg}$_2$(μ-O)] **1a** (10.7 mg, 11.9 μmol) in C_6D_6 (0.5 mL) with dideuterium (ca. 1 bar) in a J Young NMR tube at room temperature (rt). The black circle denotes the resonance associated with an unknown impurity (possibly deuterated cyclohexane). The red circle denotes the resonance associated with dideuterium. The blue circle denotes the resonance associated with Mg-*D* of [{(^{MeDip}nacnac)Mg}$_2$(μ-D)(μ-OD)] **4a-*d*₂**. The green circle denotes the resonance associated with Mg-O*D* of [{(^{MeDip}nacnac)Mg}$_2$(μ-D)(μ-OD)] **4a-*d*₂**.

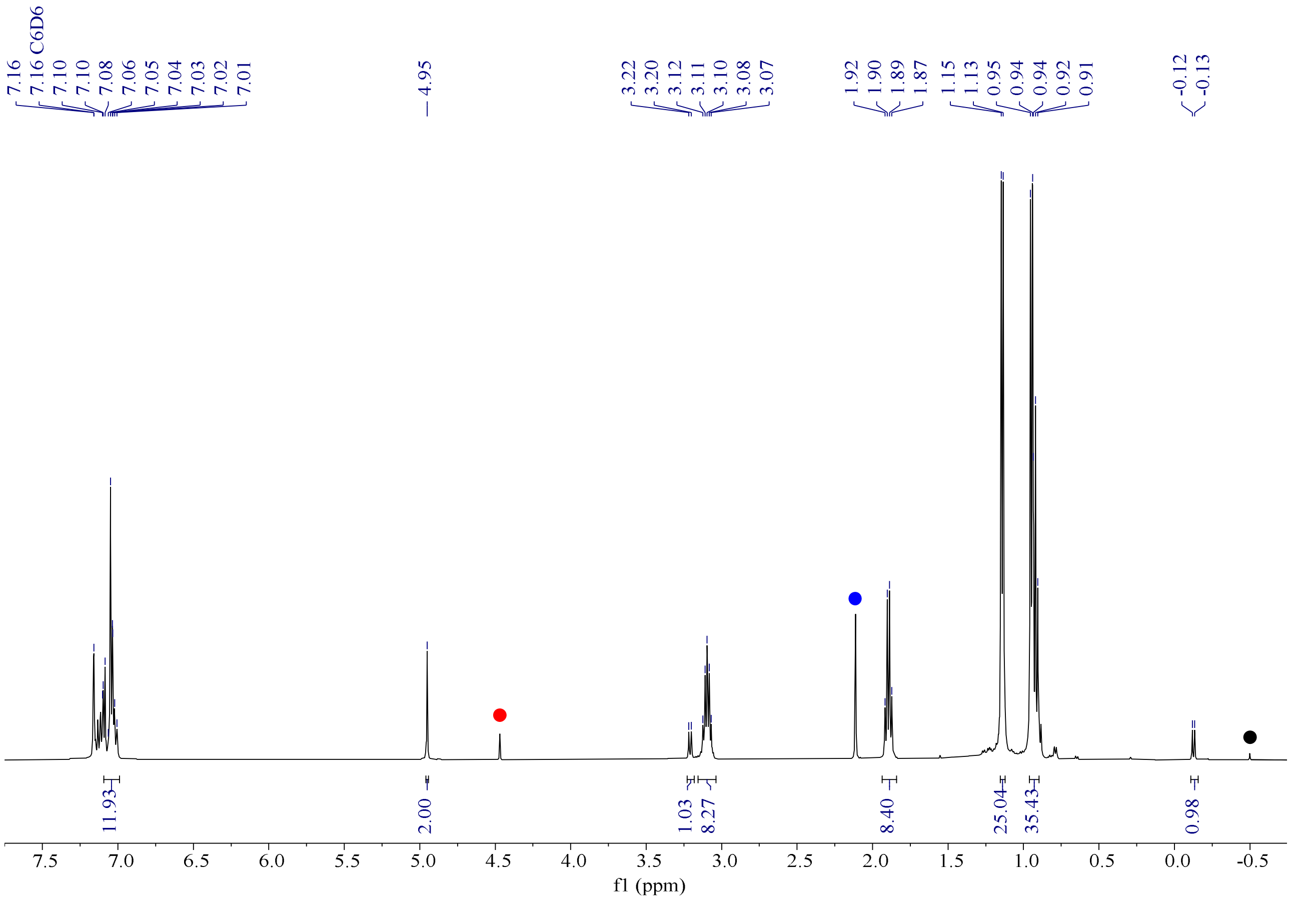

Figure S79. ^{1}H NMR spectrum (500.1 MHz, C_6D_6, 298 K) of *in-situ* generated [{(^{EtDip}nacnac)Mg}$_2$(μ-H)(μ-OH)] **4b**. The blue circle denotes the resonance associated with toluene (Ph-CH_3). The red circle denotes the resonance associated with dihydrogen. The black circle denotes the resonance associated with Mg-(OH)$_2$ of [{(^{EtDip}nacnac)Mg(μ-OH)}$_2$] **3b**.

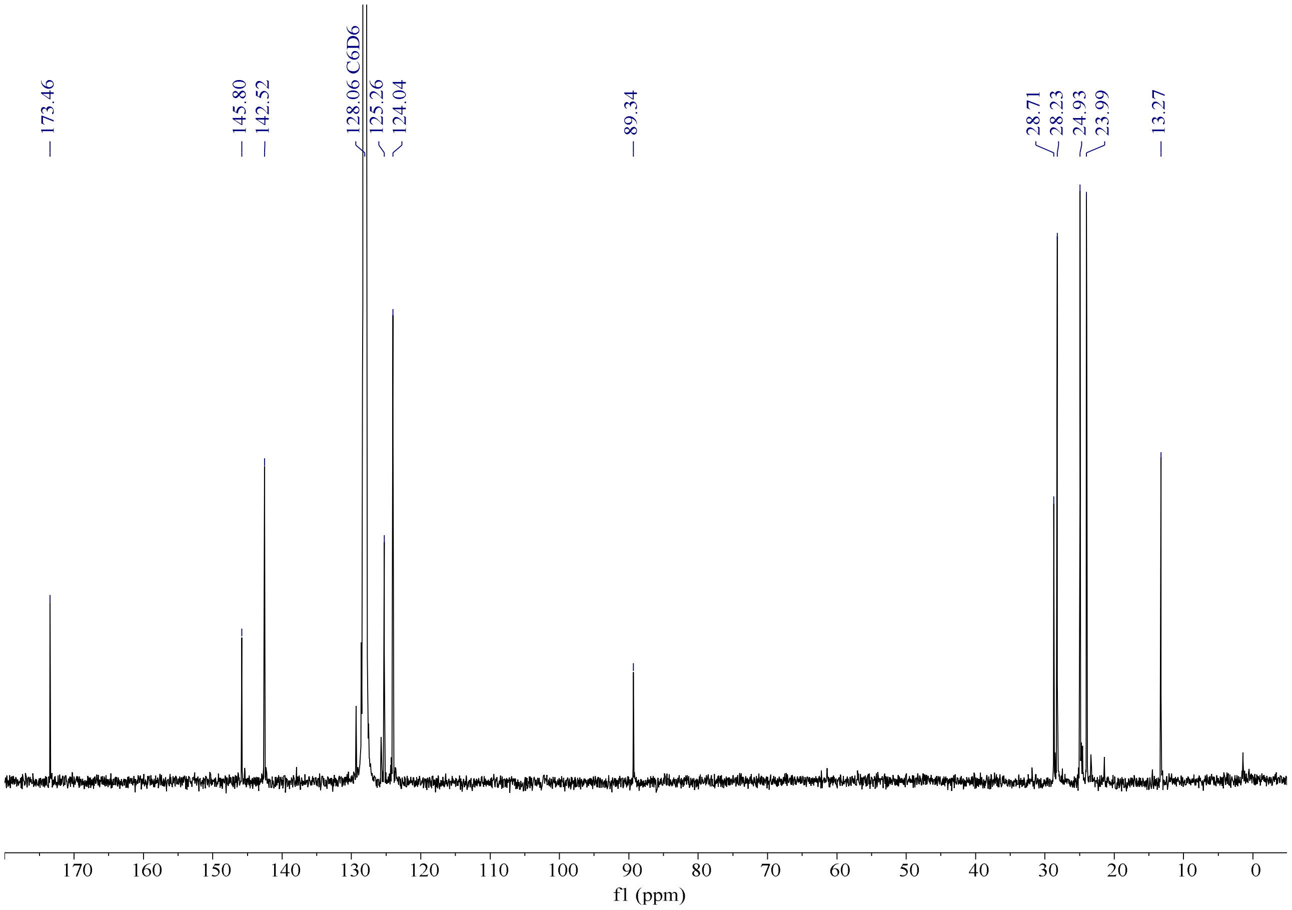

Figure S80. $^{13}C\{^{1}H\}$ NMR spectrum (125.8 MHz, C_6D_6, 295 K) of *in-situ* generated $[\{(^{EtDip}nacnac)Mg\}_2(\mu\text{-}H)(\mu\text{-}OH)]$ **4b**.

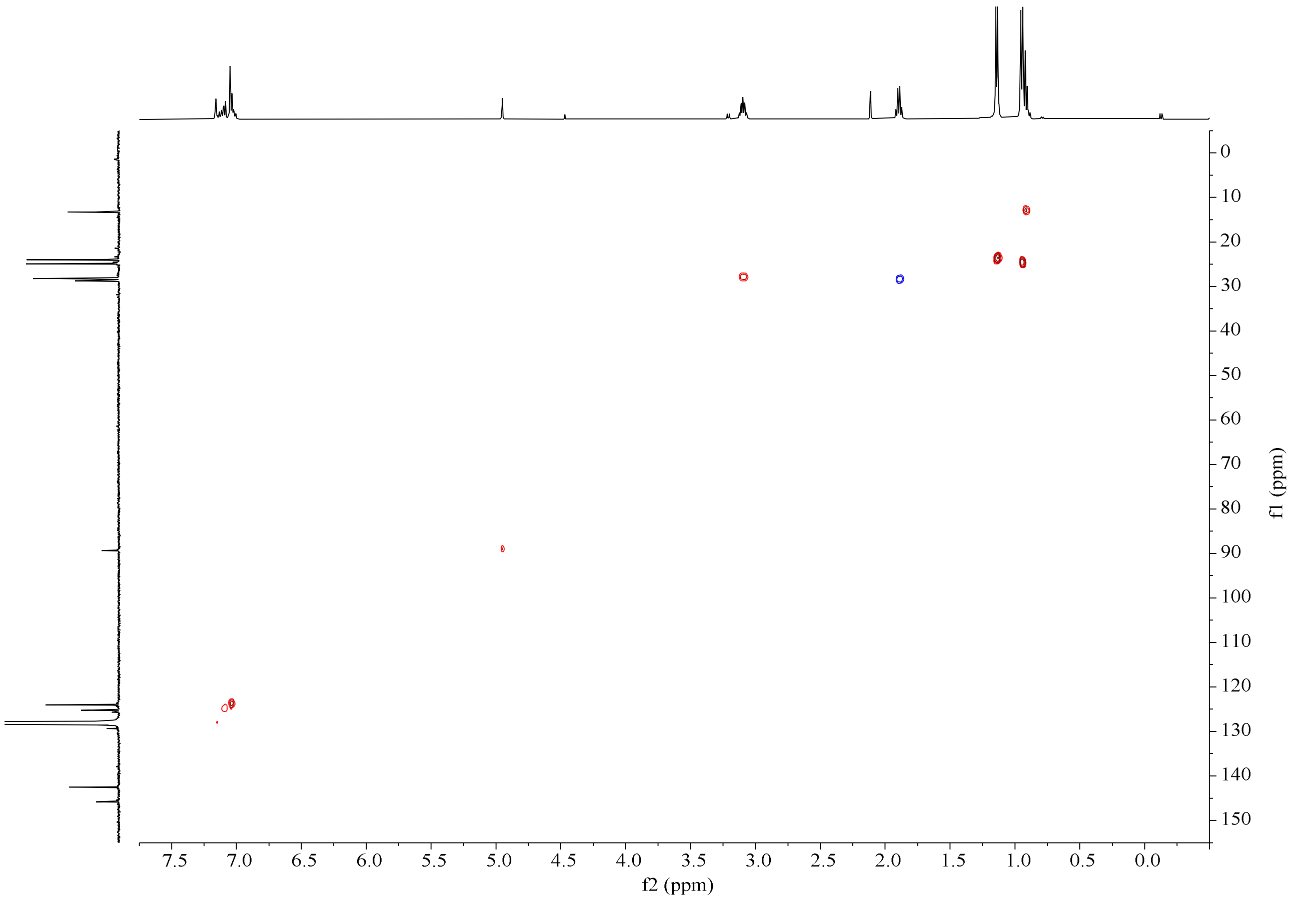

Figure S81. ^{1}H-^{13}C HSQC NMR spectrum of *in-situ* generated [{(^{EtDip}nacnac)Mg}$_2$(μ-H)(μ-OH)] **4b**.

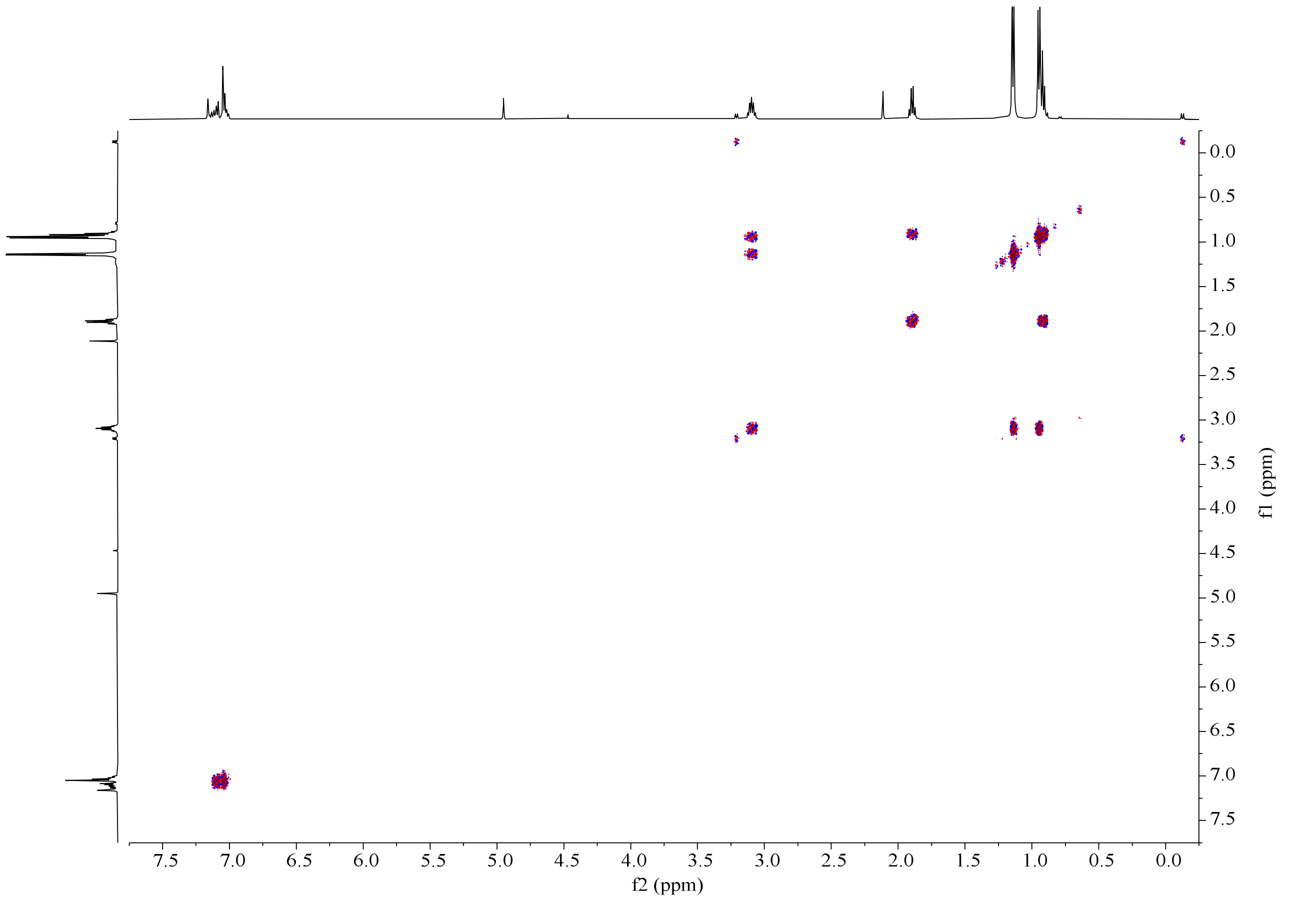

Figure S82. ^{1}H-^{1}H COSY NMR spectrum of *in-situ* generated [{(^{EtDip}nacnac)Mg}$_2$(μ-H)(μ-OH)] **4b**.

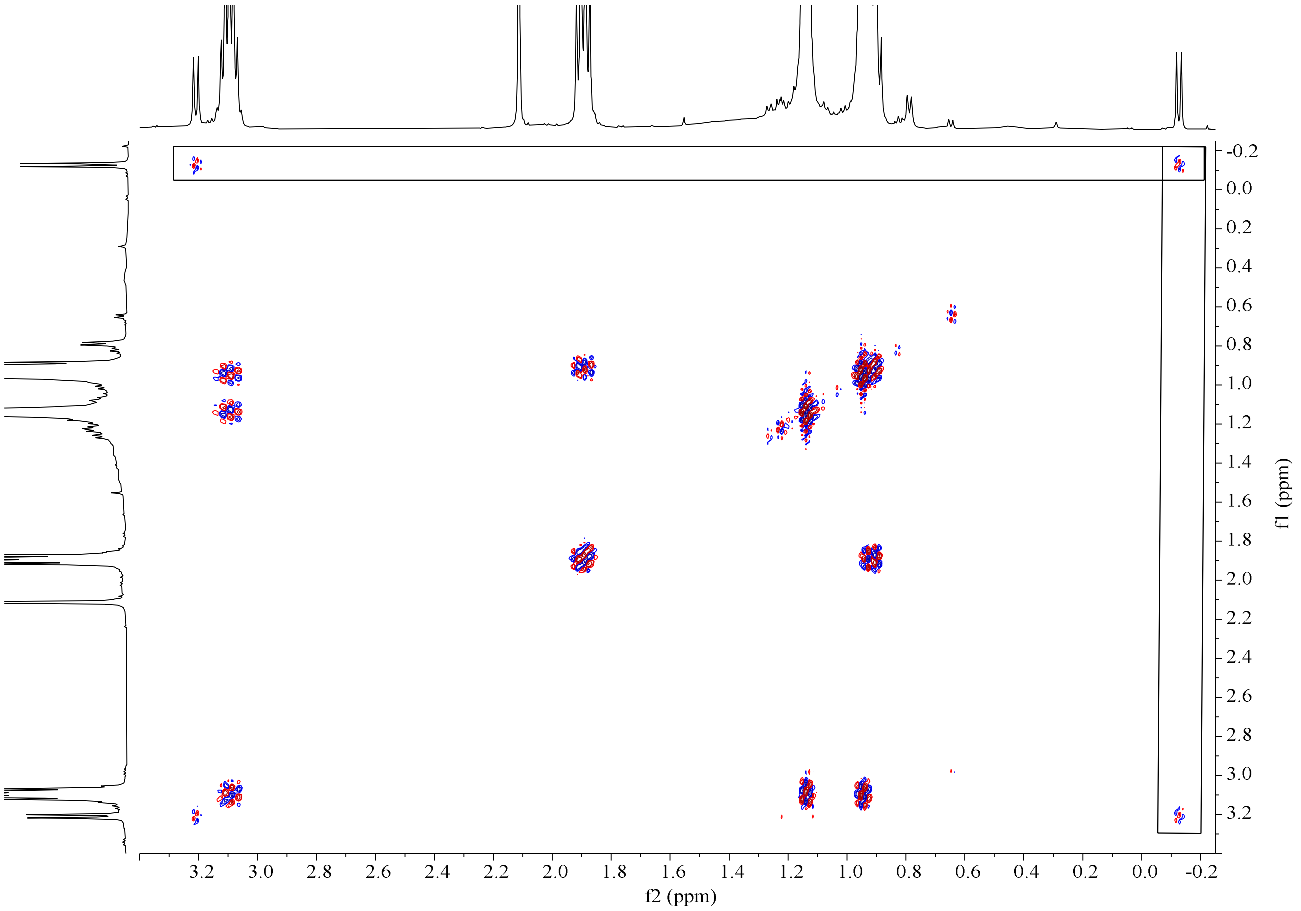

Figure S83. ^{1}H-^{1}H COSY NMR spectrum of *in-situ* generated [{(^{EtDip}nacnac)Mg}$_2$(μ-H)(μ-OH)] **4b** (chemical shift range: −0.2-3.4 ppm). The coupling between the hydroxide and hydride units is highlighted.

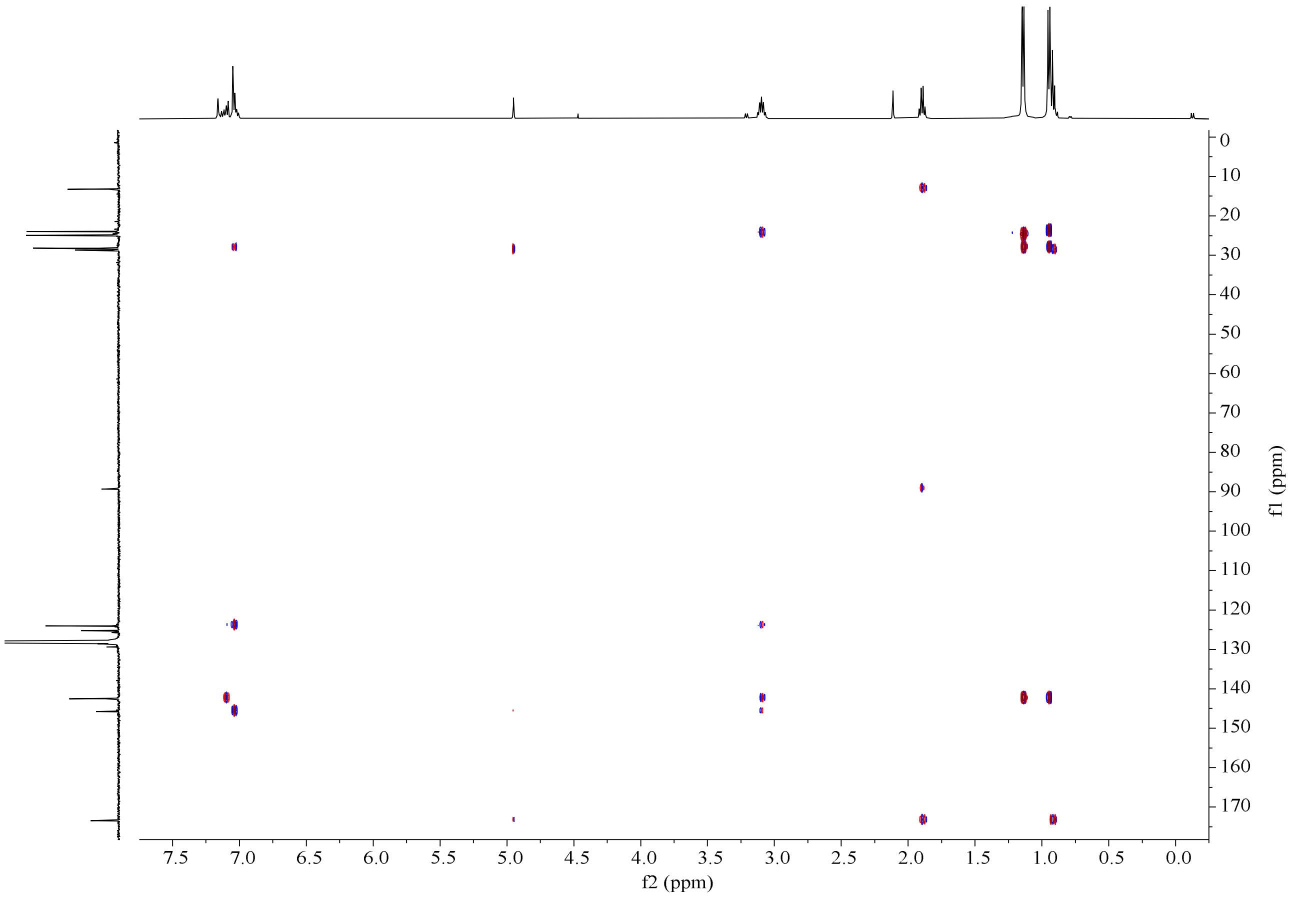

Figure S84. ^{1}H-^{13}C HMBC of *in-situ* generated [{(^{EtDip}nacnac)Mg}$_2$(μ-H)(μ-OH)] **4b**.

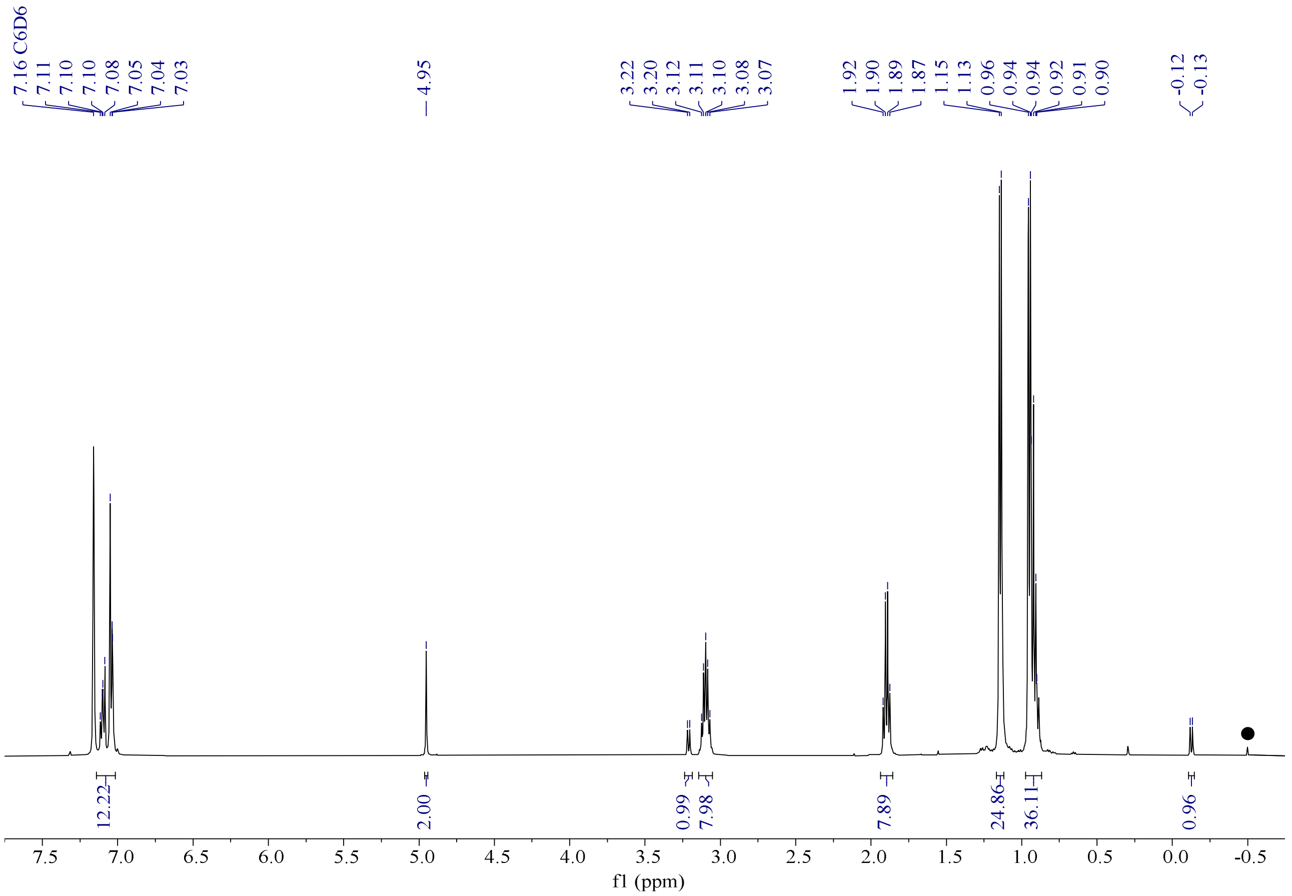

Figure S85. ^{1}H NMR spectrum (499.9 MHz, C_6D_6, 298 K) of isolated [{(^{EtDip}nacnac)Mg}$_2$(μ-H)(μ-OH)] **4b**. The black circle denotes the resonance associated with Mg-(O*H*)$_2$ of [{(^{EtDip}nacnac)Mg(μ-OH)}$_2$] **3b**.

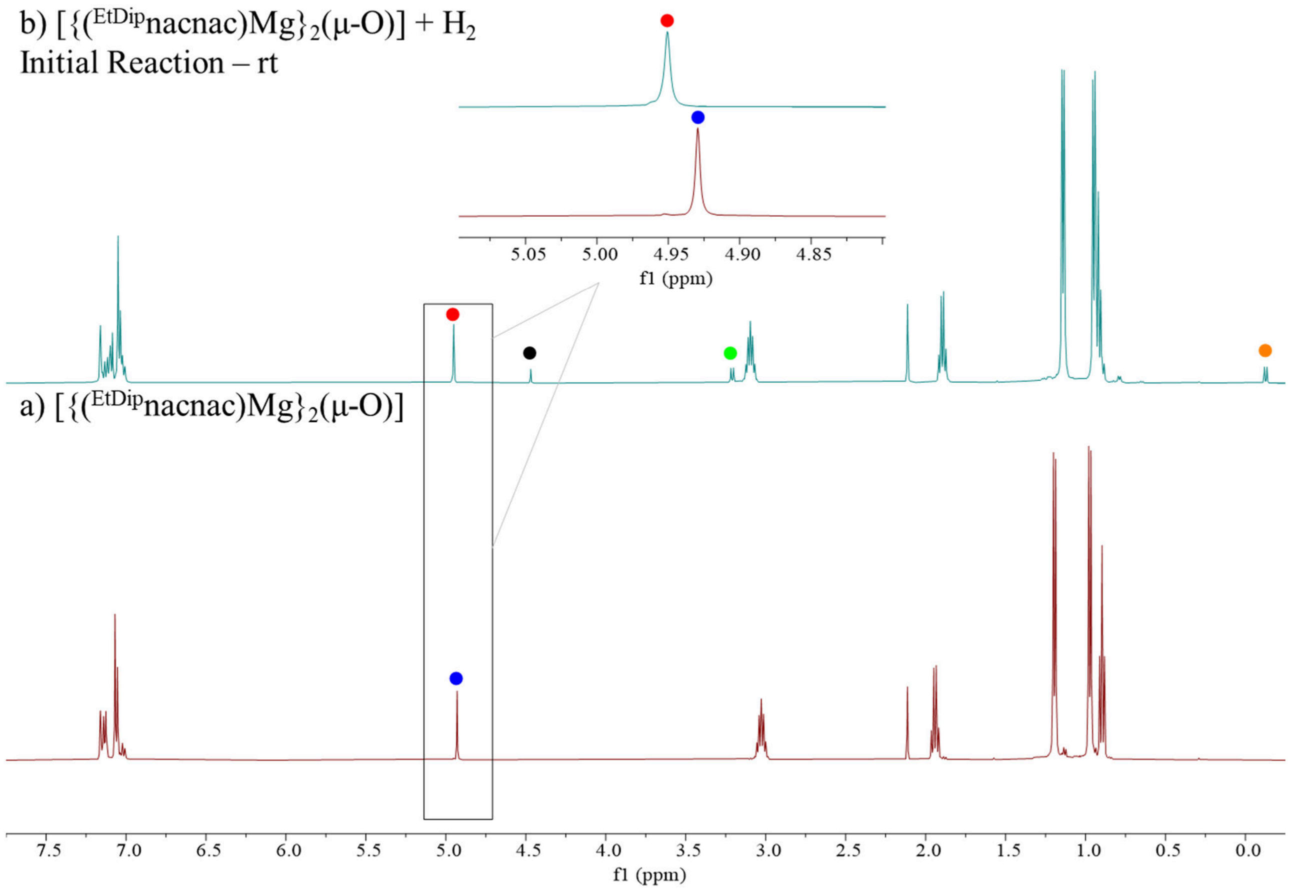

Figure S86. Stacked ^{1}H NMR spectra of the reaction of a colourless *in-situ* generated solution of [{(^{EtDip}nacnac)Mg}$_2$(μ-O)] **1b** (10.0 mg, 10.5 μmol) and dihydrogen (ca. 1 bar) in C_6D_6 (0.6 mL) at room temperature (rt) in a J Young NMR tube. The black circle denotes the resonance associated with dihydrogen. The blue circle denotes the resonance associated with the backbone-C*H* of [{(^{EtDip}nacnac)Mg}$_2$(μ-O)] **1b**. The red circle denotes the resonance associated with the backbone-C*H* of [{(^{EtDip}nacnac)Mg}$_2$(μ-H)(μ-OH)] **4b**. The green circle denotes the resonance associated with Mg-*H* of [{(^{EtDip}nacnac)Mg}$_2$(μ-H)(μ-OH)] **4b**. The orange circle denotes the resonance associated with Mg-O*H* of [{(^{EtDip}nacnac)Mg}$_2$(μ-H)(μ-OH)] **4b**. Spectrum a): 499.9 MHz, C_6D_6, 298 K. Spectrum b): 500.1 MHz, C_6D_6, 298 K.

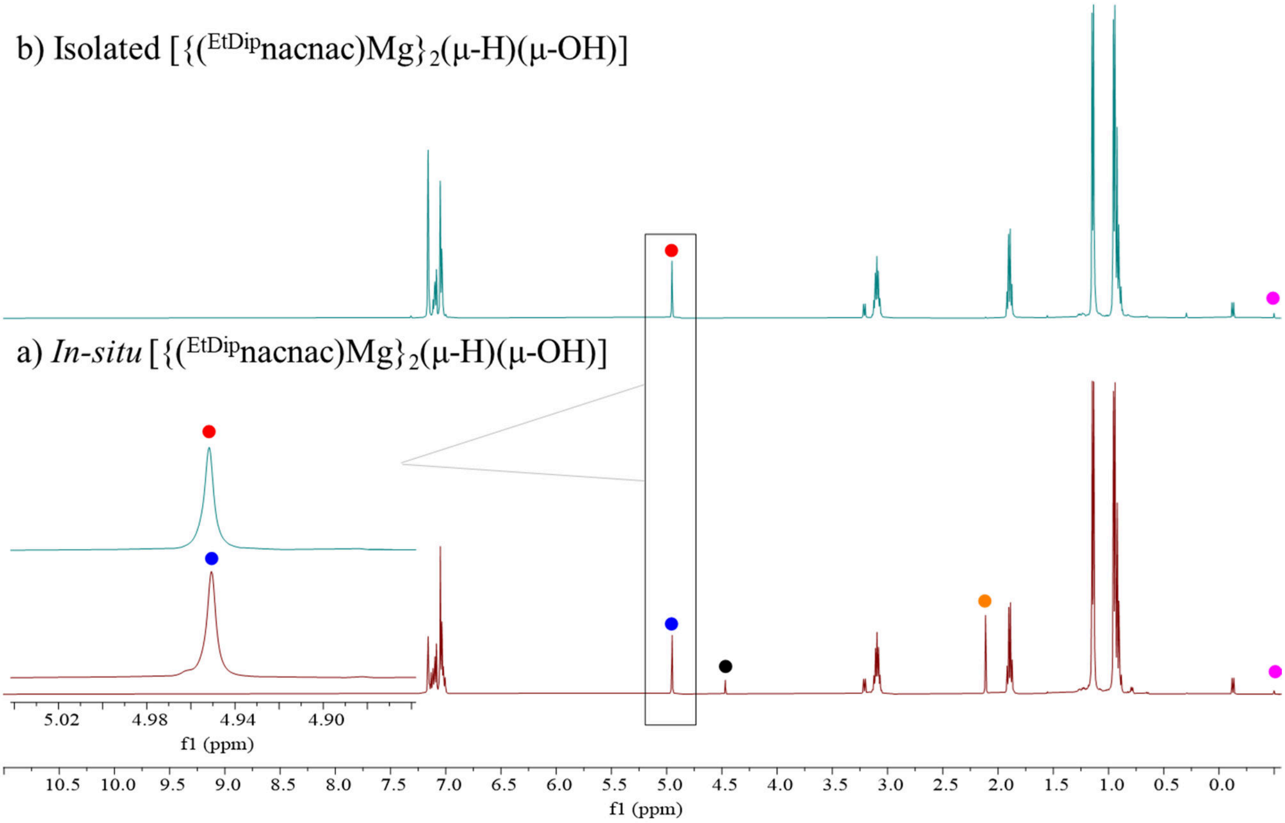

Figure S87. Stacked ^{1}H NMR spectra of *in-situ* generated [{(^{EtDip}nacnac)Mg}$_2$(μ-H)(μ-OH)] **4b** and isolated [{(^{EtDip}nacnac)Mg}$_2$(μ-H)(μ-OH)] **4b**. The orange circle denotes the resonance associated with toluene (Ph-CH_3). The black circle denotes the resonance associated with dihydrogen. The blue circle denotes the resonance associated with the backbone-CH of *in-situ* generated [{(^{EtDip}nacnac)Mg}$_2$(μ-H)(μ-OH)] **4b**. The red circle denotes the resonance associated with the backbone-CH of isolated [{(^{EtDip}nacnac)Mg}$_2$(μ-H)(μ-OH)] **4b**. The pink circle denotes the resonance associated with Mg-(OH)$_2$ of [{(^{EtDip}nacnac)Mg(μ-OH)}$_2$] **4b**. Spectrum a) 500.1 MHz, C_6D_6, 298 K. Spectrum b) 499.9 MHz, C_6D_6, 298 K.

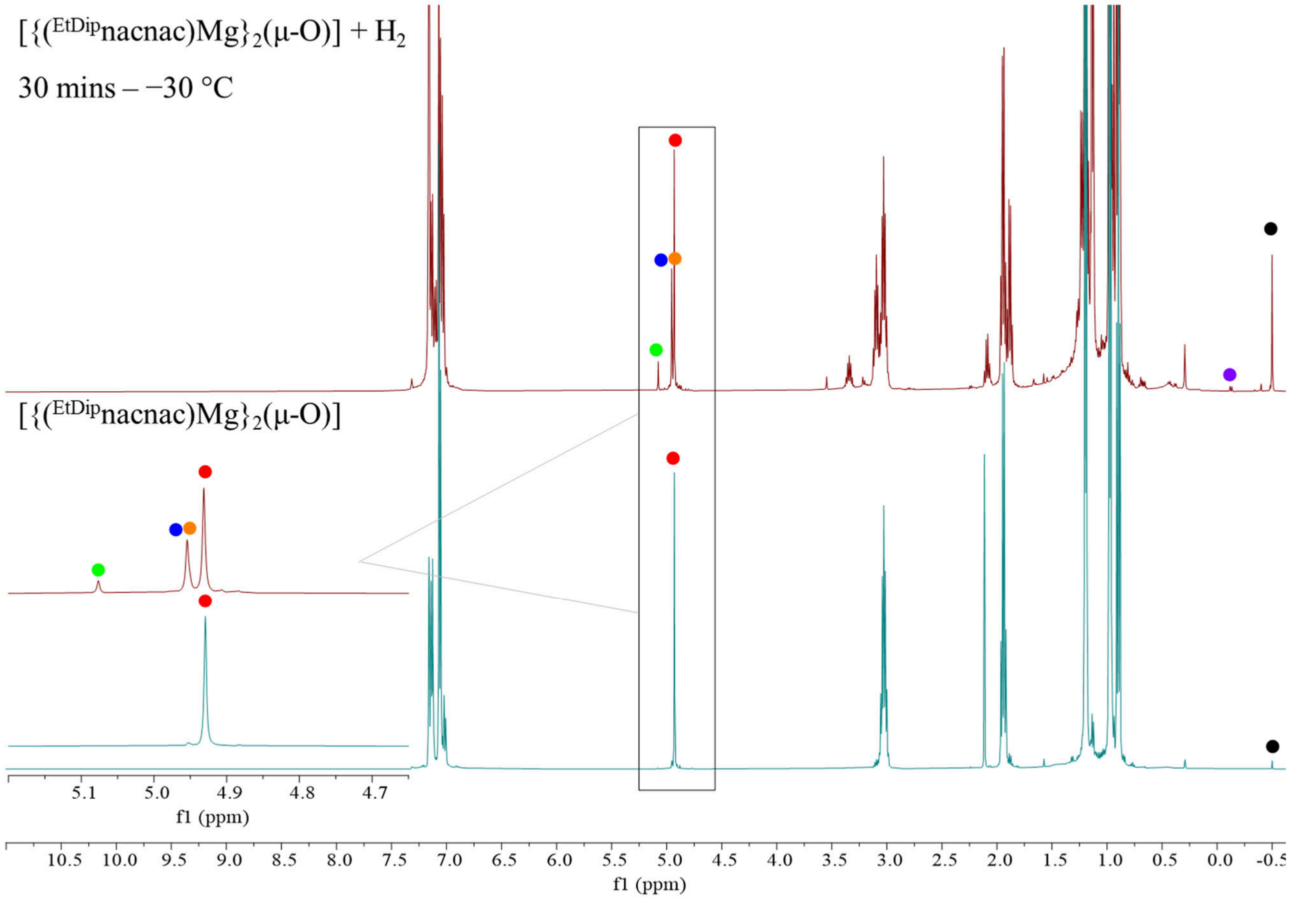

Figure S88. Stacked ^{1}H NMR spectra (499.9 MHz, C_6D_6, 298 K) of the reaction of a colourless *in-situ* generated solution of [{(^{EtDip}nacnac)Mg}$_2$(μ-O)] **1b** (10.0 mg, 10.5 μmol) and dihydrogen (ca. 1 bar) in *n*-hexane (0.5 mL) at −30°C for 30 minutes. The red circle denotes the resonance associated with the backbone-C*H* of [{(^{EtDip}nacnac)Mg}$_2$(μ-O)] **1b**. The blue circle denotes the resonance associated with the backbone-C*H* of [{(^{EtDip}nacnac)Mg}$_2$(μ-H)(μ-OH)] **4b**. The purple circle denotes the resonance associated with Mg-(O*H*) of [{(^{EtDip}nacnac)Mg}$_2$(μ-H)(μ-OH)] **4b**. The green circle denotes the resonance associated with the backbone-C*H* of ^{EtDip}nacnacH. The orange circle denotes the resonance associated with the backbone-C*H* of [{(^{EtDip}nacnac)Mg(μ-OH)}$_2$] **3b** (overlapping with the resonance associated with the backbone-C*H* of [{(^{EtDip}nacnac)Mg}$_2$(μ-H)(μ-OH)] **4b**). The black circle denotes the resonance associated with Mg-(O*H*)$_2$ of [{(^{EtDip}nacnac)Mg(μ-OH)}$_2$] **3b**.

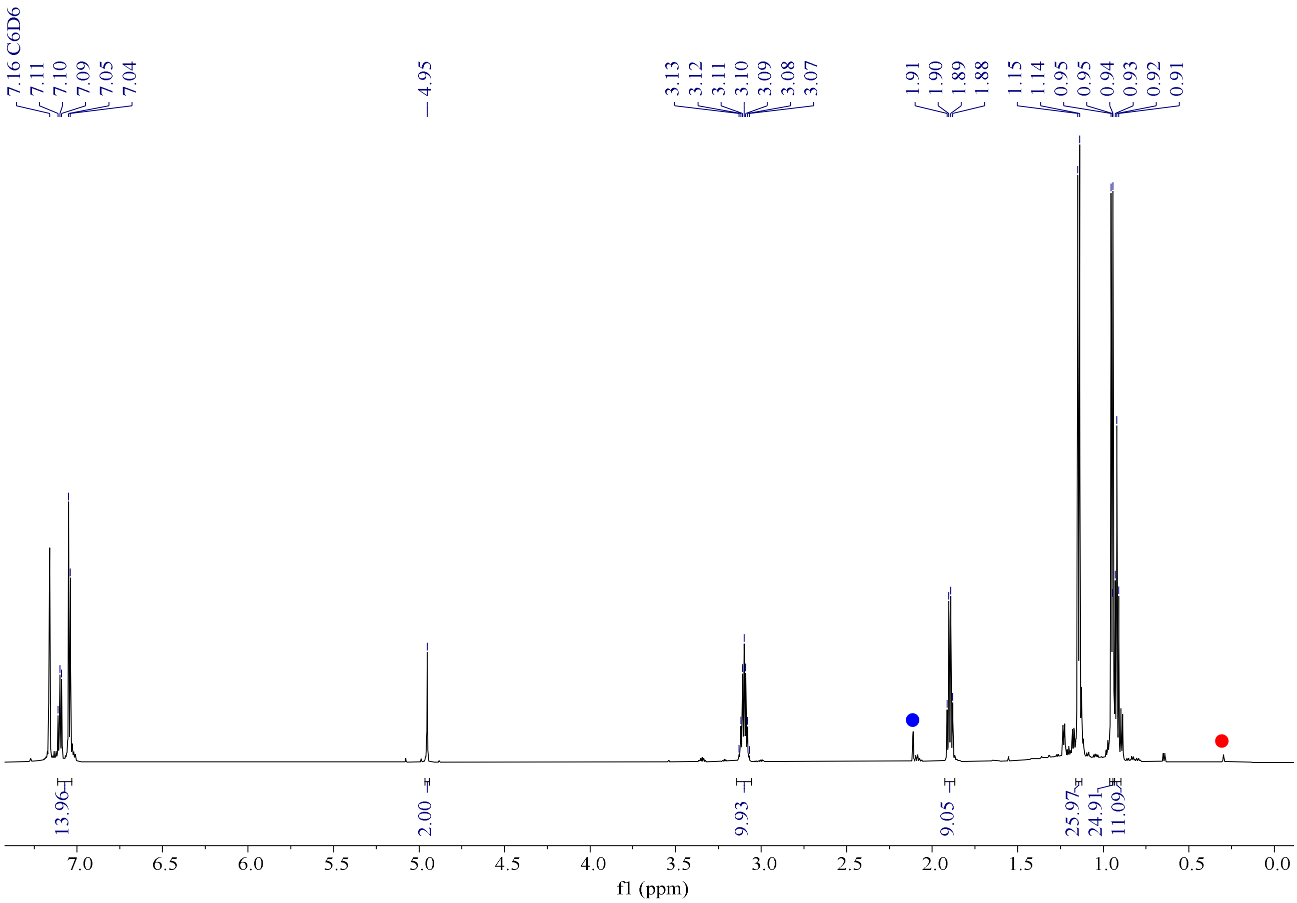

Figure S89. ^{1}H NMR spectrum (700.0 MHz, C_6D_6, 295 K) of *in-situ* generated [{(^{EtDip}nacnac)Mg}$_2$(μ-D)(μ-OD)] **4b-*d*$_2$**. The red circle denotes the resonance associated with silicone grease. The blue circle denotes the resonance associated with toluene (Ph-CH_3).

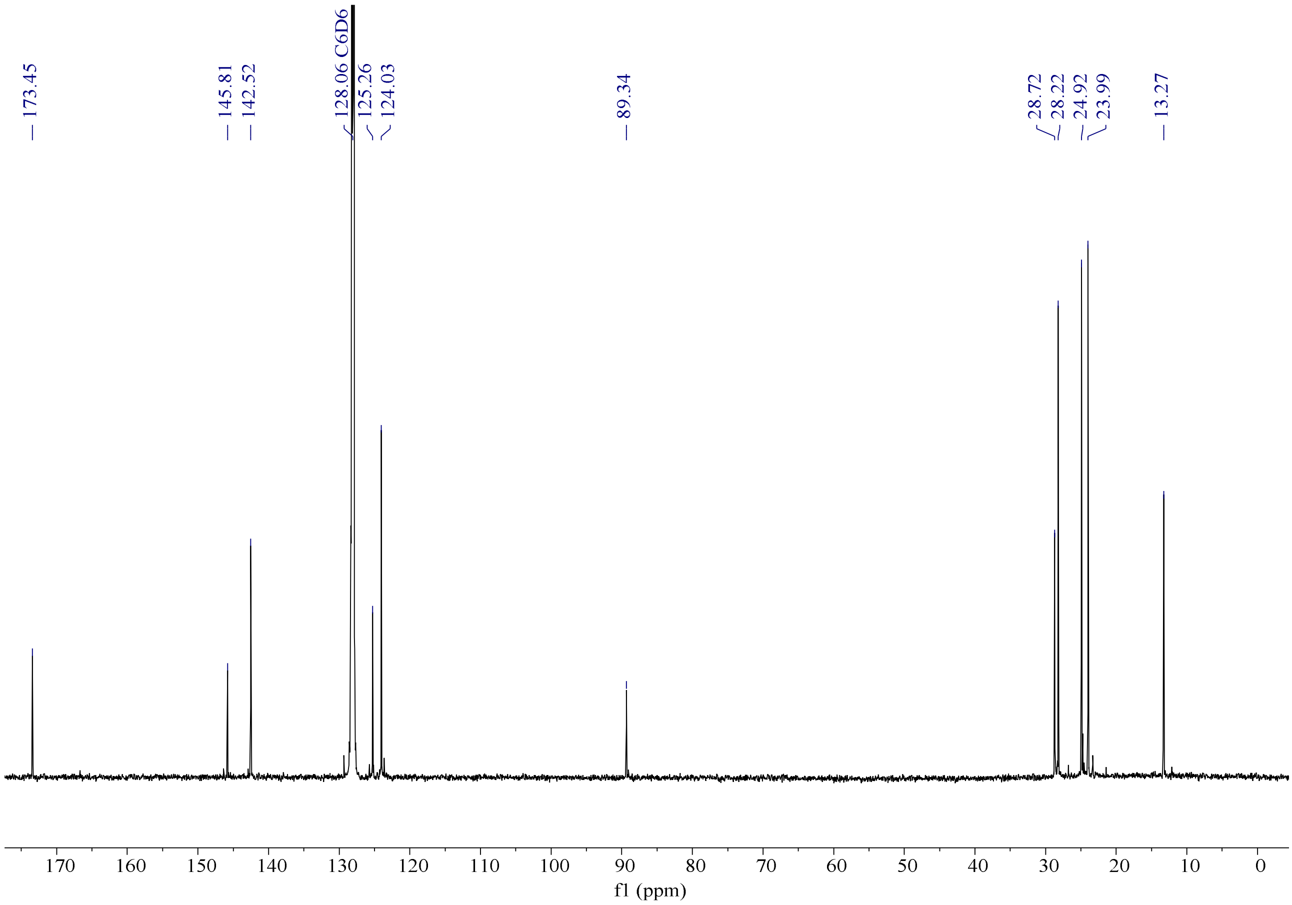

Figure S90. $^{13}C\{^{1}H\}$ NMR spectrum (176.0 MHz, C_6D_6, 295 K) of *in-situ* generated $[\{(^{EtDip}nacnac)Mg\}_2(\mu\text{-}D)(\mu\text{-}OD)]$ **4b-*d*₂**.

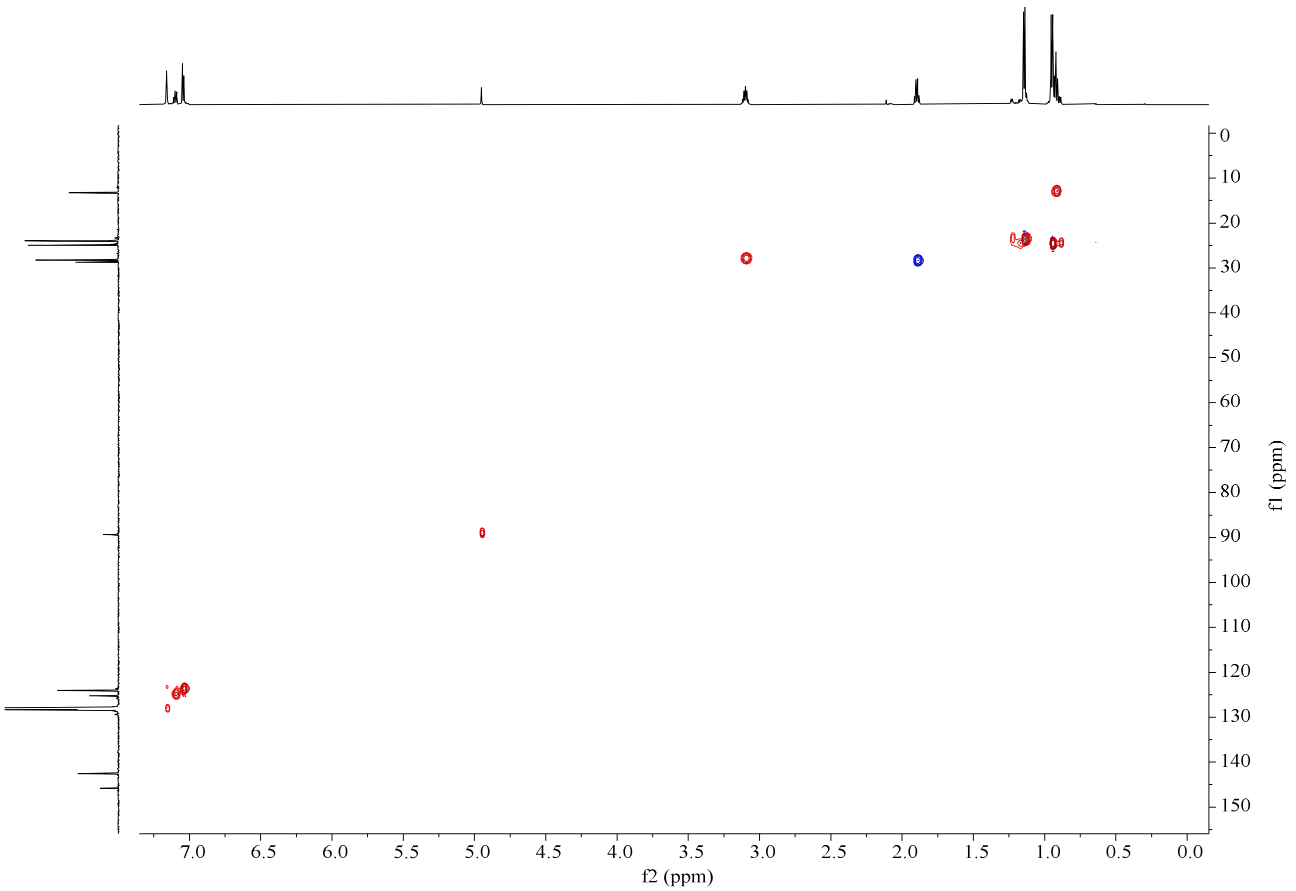

Figure S91. ^{1}H-^{13}C HSQC NMR spectrum of *in-situ* generated [{(^{EtDip}nacnac)Mg}$_2$(μ-D)(μ-OD)] **4b-*d*$_2$**.

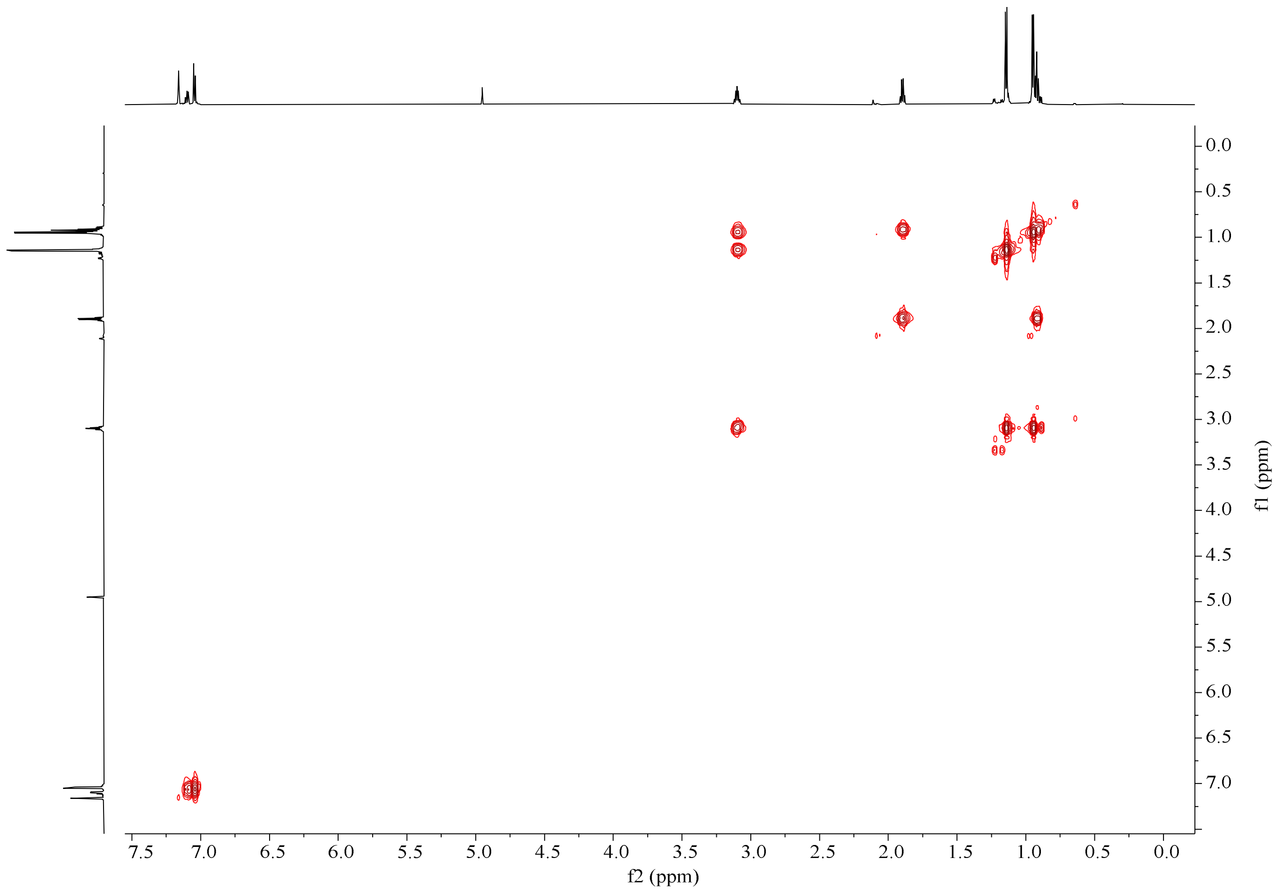

Figure S92. ^{1}H-^{1}H COSY NMR spectrum of *in-situ* generated [{(^{EtDip}nacnac)Mg}$_2$(μ-D)(μ-OD)] **4b-*d*$_2$**.

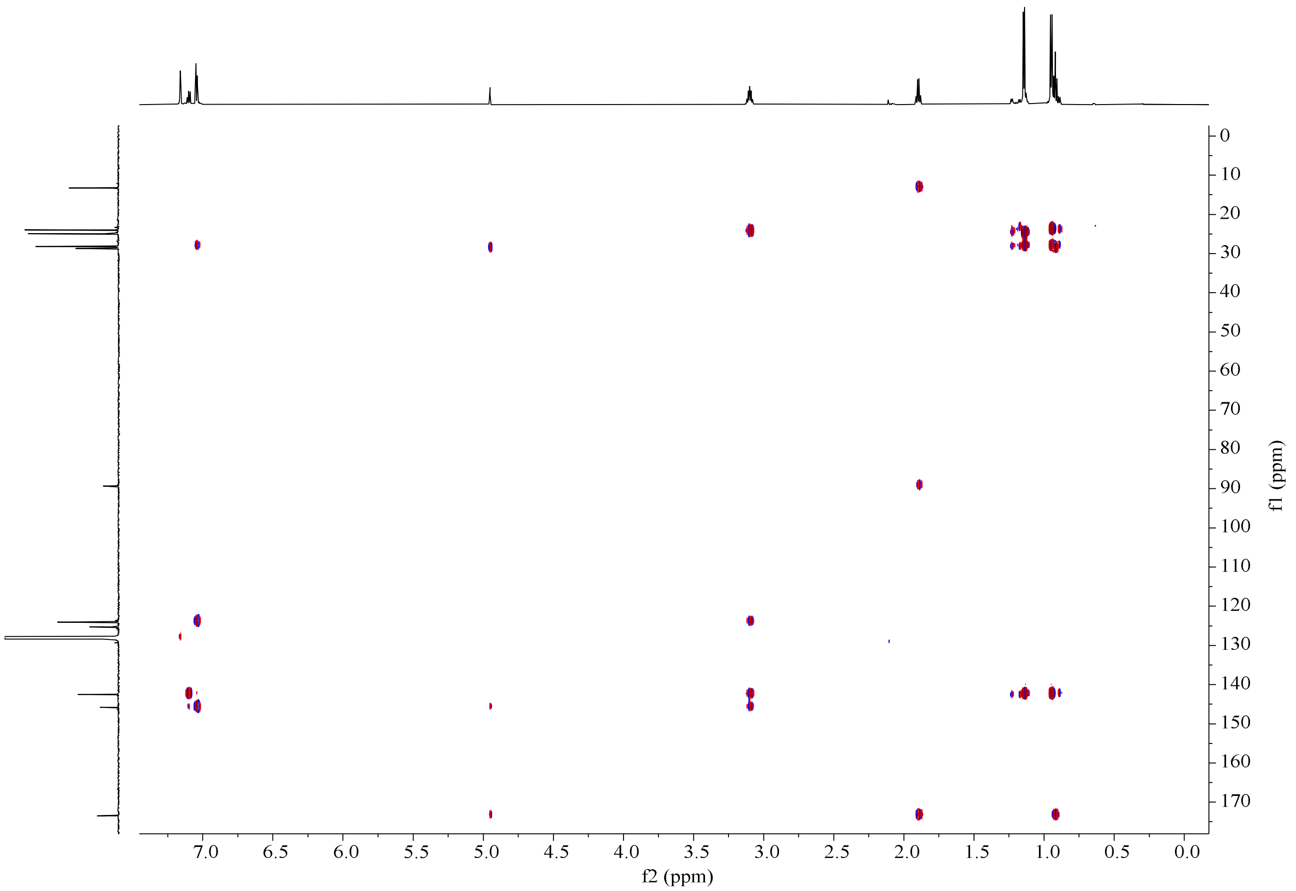

Figure S93. ^{1}H-^{13}C HMBC NMR spectrum of *in-situ* generated [{(^{EtDip}nacnac)Mg}$_2$(μ-D)(μ-OD)] **4b-*d*$_2$**.

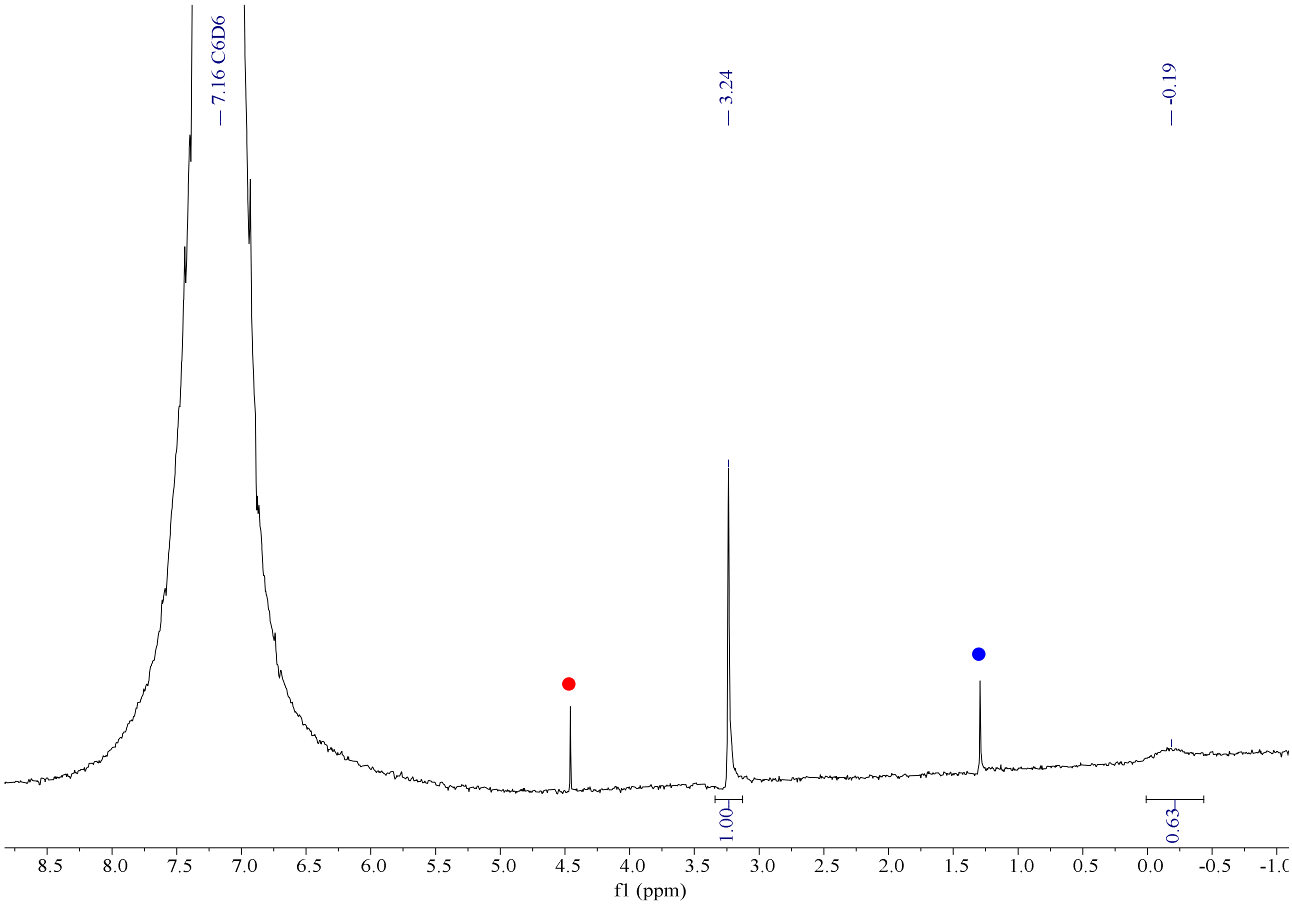

Figure S94. ^{2}H NMR spectrum (107.5 MHz, C_6D_6, 295 K) of *in-situ* generated $[\{(^{EtDip}nacnac)Mg\}_2(\mu\text{-}D)(\mu\text{-}OD)]$ **4b-*d*2**. The red circle denotes the resonance associated with dideuterium. The blue circle denotes the resonance associated with an unknown impurity (possibly deuterated cyclohexane).

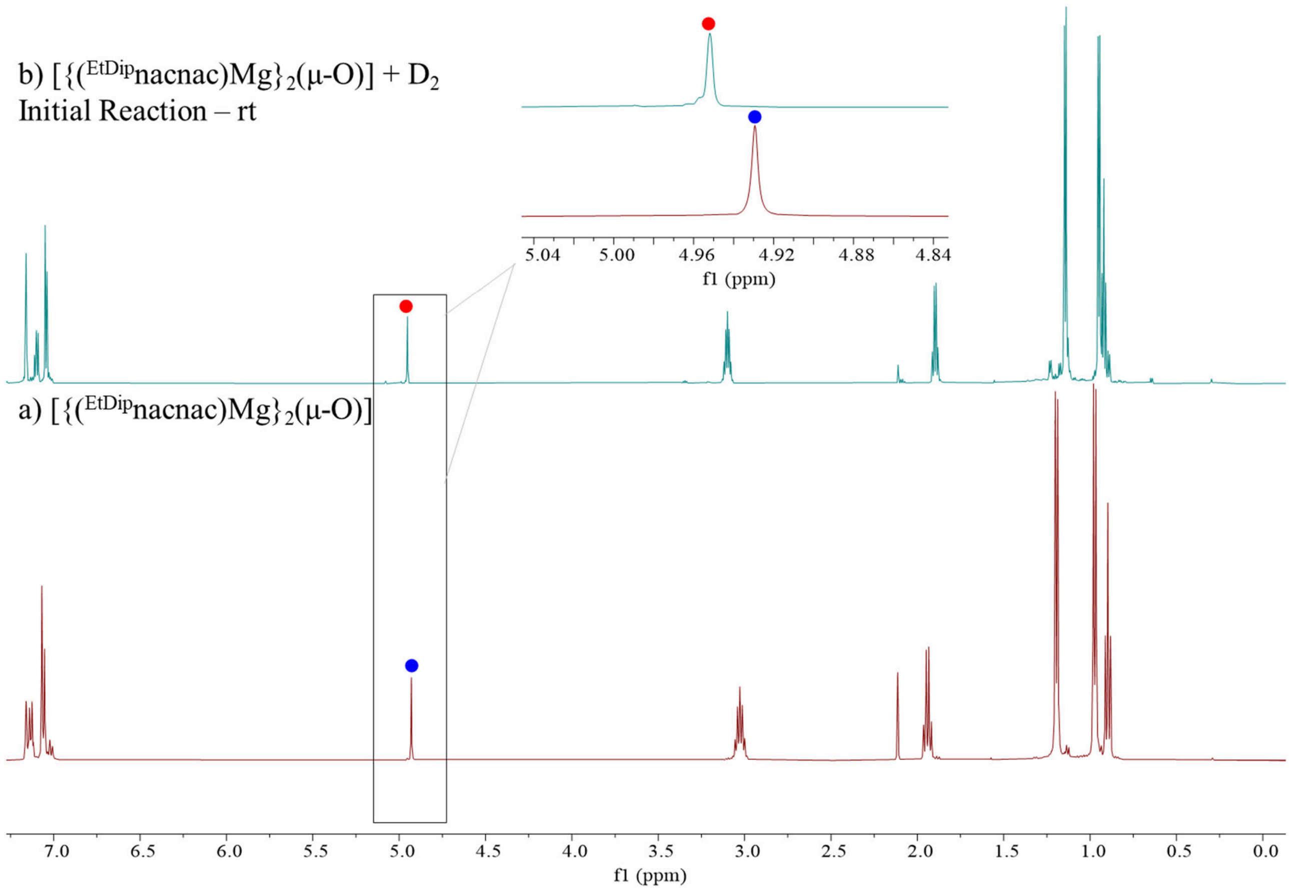

Figure S95. Stacked ^{1}H NMR spectra of the reaction of a colourless *in-situ* generated solution of [{(^{EtDip}nacnac)Mg}$_2$(μ-O)] **1b** (10.2 mg, 10.7 μmol) and dideuterium (ca. 1 bar) in C_6D_6 (0.5 mL) in a J Young NMR tube at room temperature (rt). The blue circle denotes the resonance associated with the backbone-C*H* of [{(^{EtDip}nacnac)Mg}$_2$(μ-O)] **1b**. The red circle denotes the resonance associated with the backbone-C*H* of [{(^{EtDip}nacnac)Mg}$_2$(μ-D)(μ-OD)] **4b-*d*$_2$**. Spectrum a) 499.9 MHz, C_6D_6, 298 K. Spectrum b) 700.0 MHz, C_6D_6, 295 K.

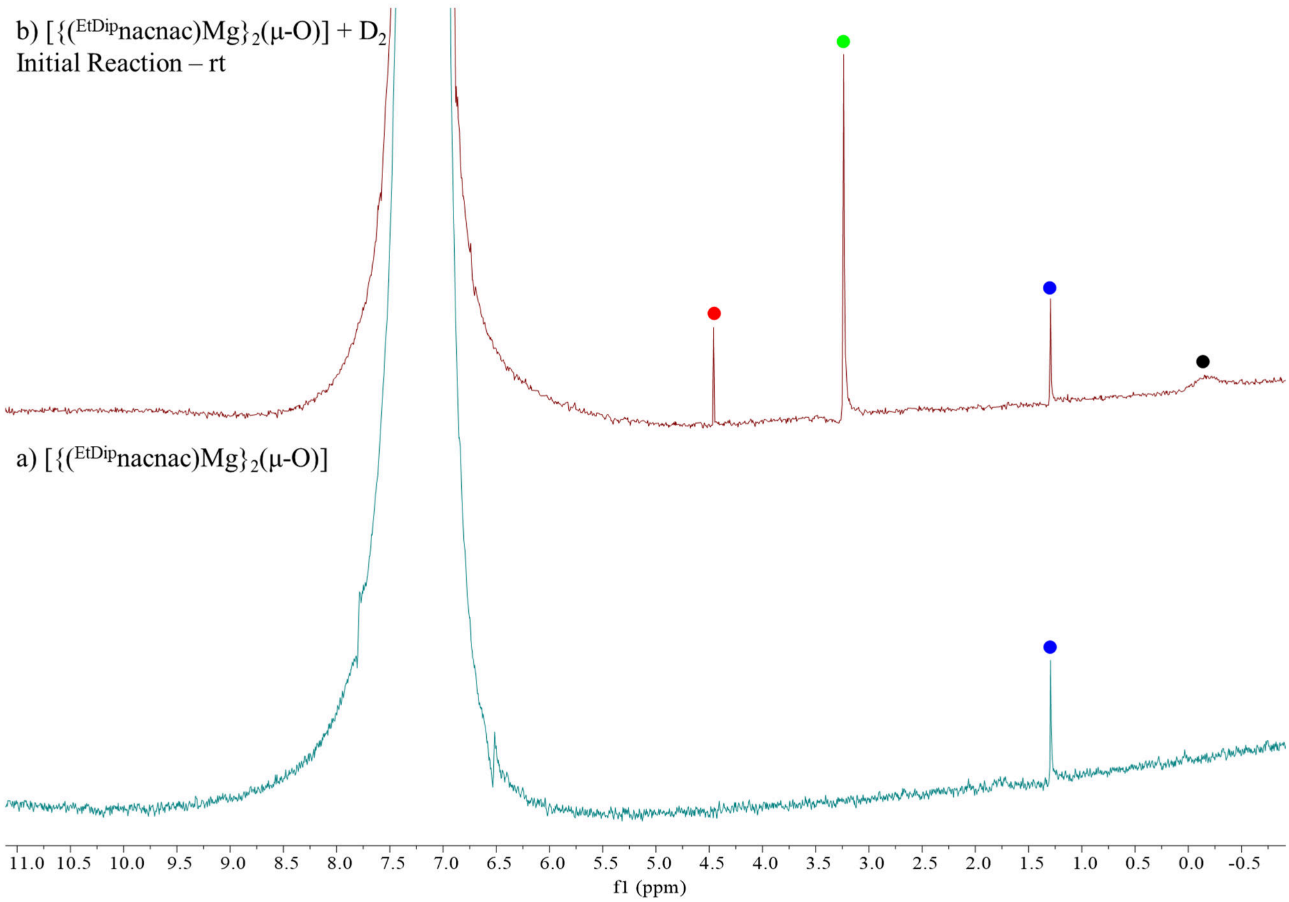

Figure S96. Stacked ^{2}H NMR spectra of the reaction of a colourless *in-situ* generated solution of [{(^{EtDip}nacnac)Mg}$_2$(μ-O)] **1b** (10.2 mg, 10.7 μmol) and dideuterium (ca. 1 bar) in C_6D_6 (0.5 mL) in a J Young NMR tube at room temperature (rt). The blue circle denotes the resonance associated with an unknown impurity (possibly deuterated cyclohexane). The red circle denotes the resonance associated with dideuterium. The green circle denotes the resonance associated with Mg-*D* of [{(^{EtDip}nacnac)Mg}$_2$(μ-D)(μ-OD)] **4b-*d*₂**. The black circle denotes the resonance associated with Mg-O*D* of [{(^{EtDip}nacnac)Mg}$_2$(μ-D)(μ-OD)] **4b-*d*₂**. Spectrum a) 76.8 MHz, C_6D_6, 298K. Spectrum b) 107.5 MHz, C_6D_6, 295 K.

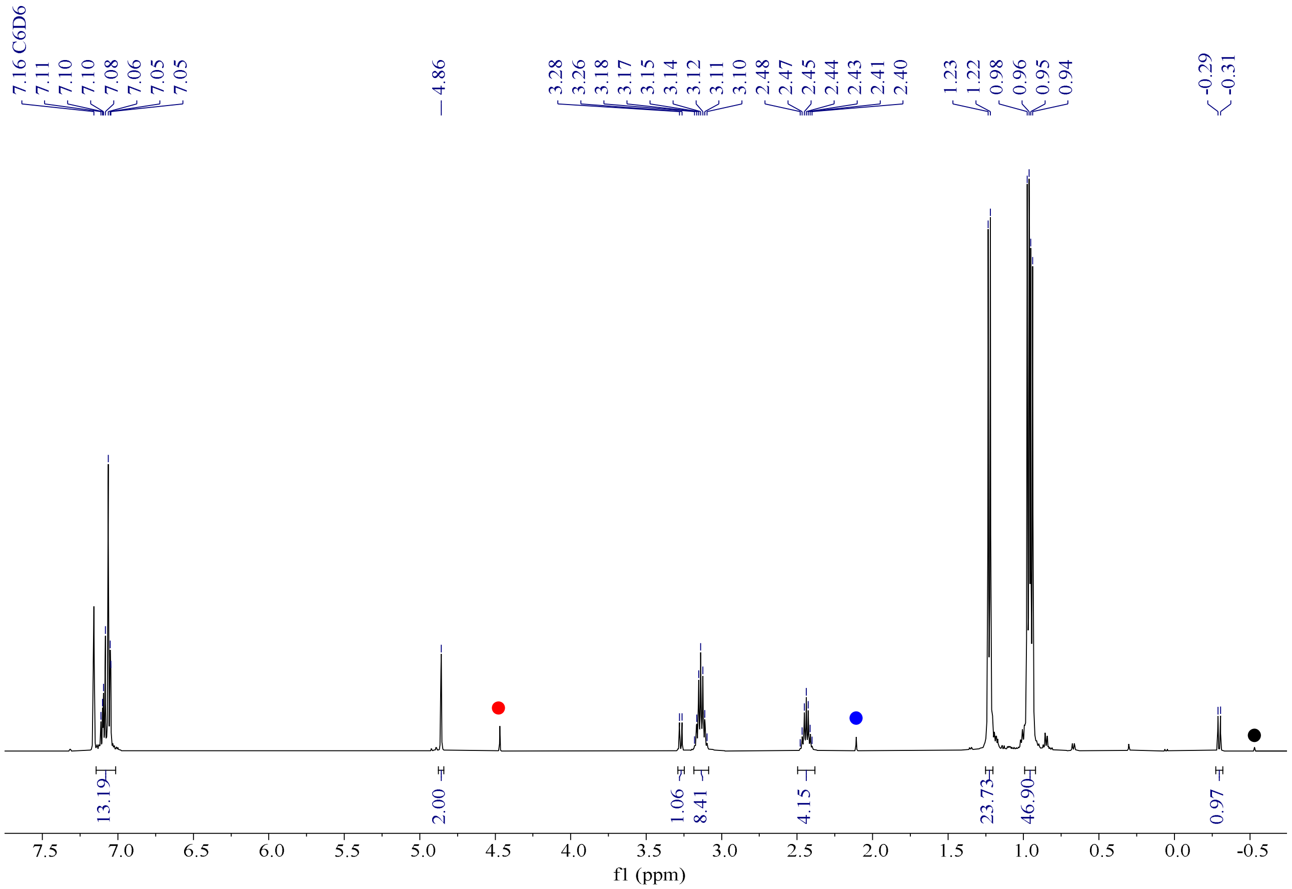

Figure S97. ^{1}H NMR spectrum (499.9 MHz, C_6D_6, 283 K) of *in-situ* generated [{(^{iPrDip}nacnac)Mg}$_2$(μ-H)(μ-OH)] **4c**. The red circle denotes the resonance associated with dihydrogen. The blue circle denotes the resonance associated with toluene (Ph-CH_3). The black circle denotes the resonance associated with Mg-(OH)$_2$ of [{(^{iPrDip}nacnac)Mg(μ-OH)}$_2$] **3c**.

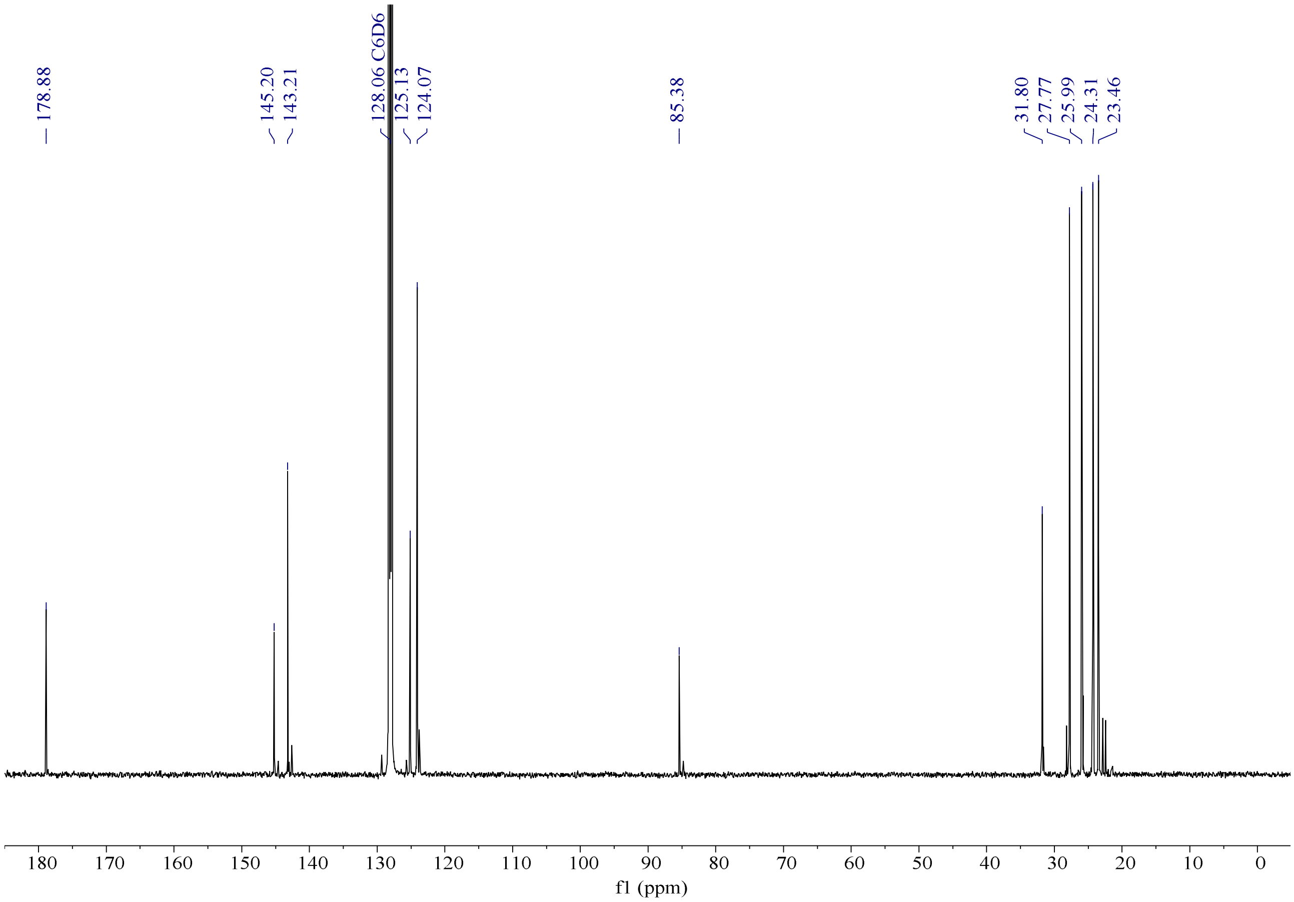

Figure S98. $^{13}C\{^1H\}$ NMR spectrum (125.7 MHz, C_6D_6, 323 K) of *in-situ* generated $[\{(^{iPrDip}nacnac)Mg\}_2(\mu\text{-}H)(\mu\text{-}OH)]$ **4c**.

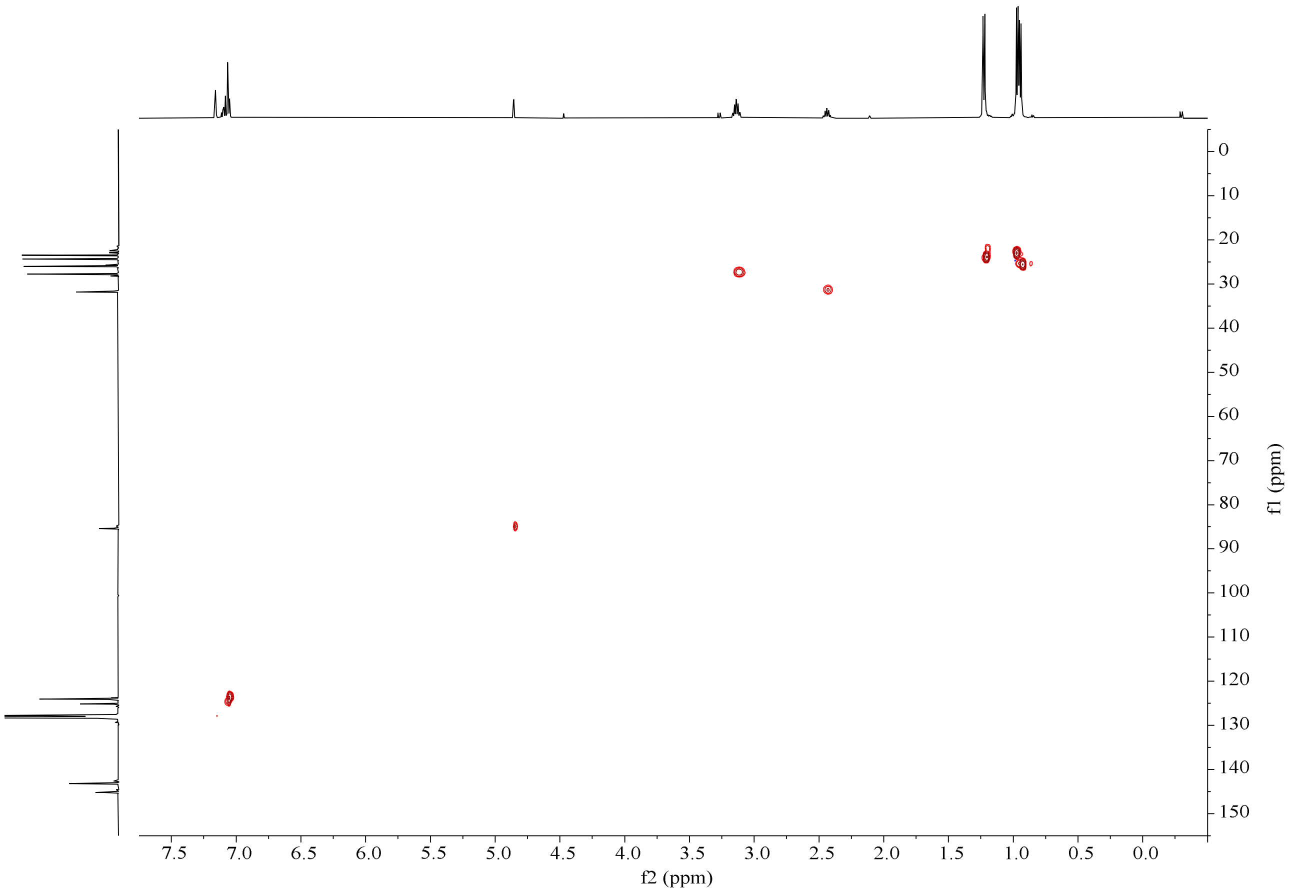

Figure S99. ^{1}H-^{13}C HSQC NMR spectrum of *in-situ* generated [{(^{iPrDip}nacnac)Mg}$_2$(μ-H)(μ-OH)] **4c**.

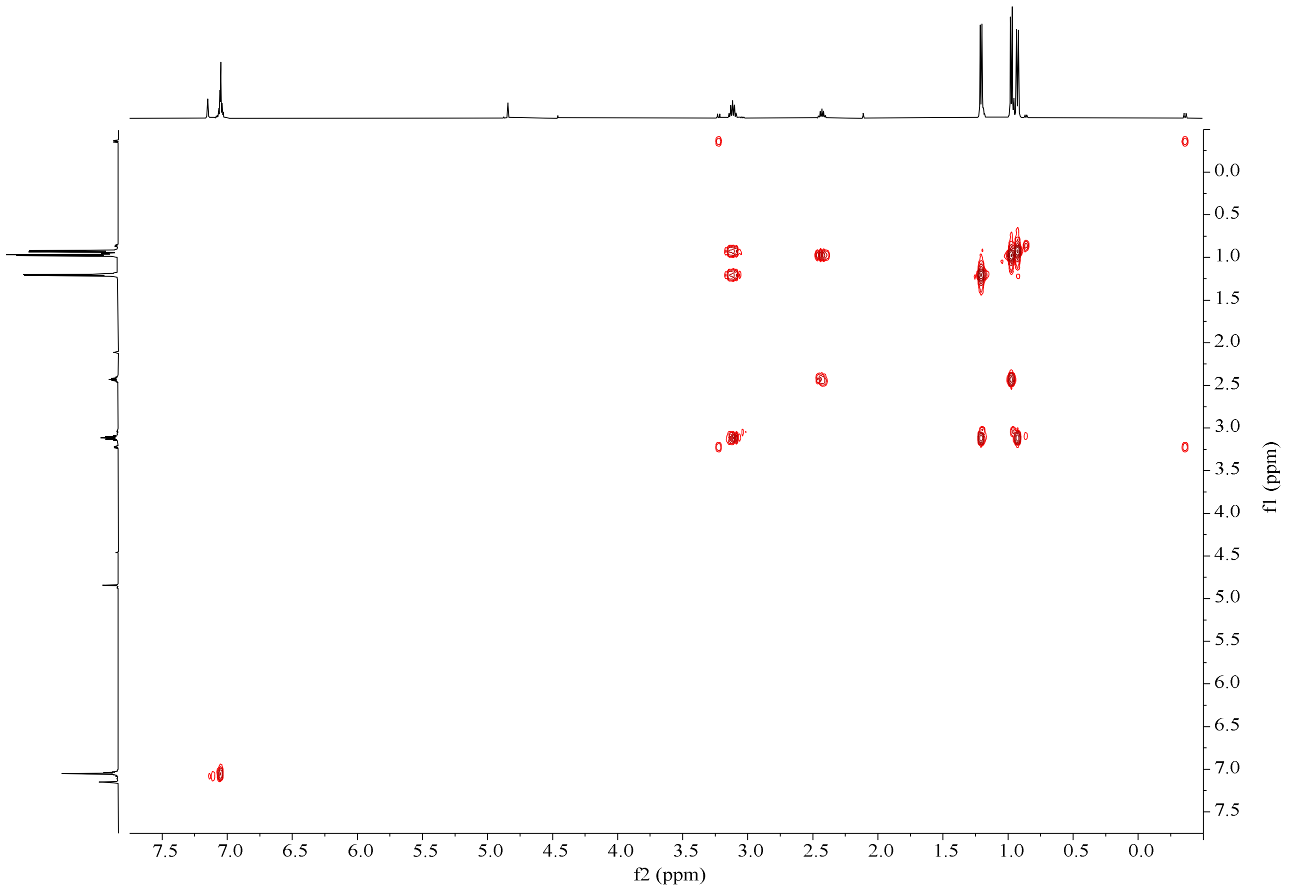

Figure S100. ^{1}H-^{1}H COSY NMR spectrum of *in-situ* generated [{(^{iPrDip}nacnac)Mg}$_2$(μ-H)(μ-OH)] **4c**.

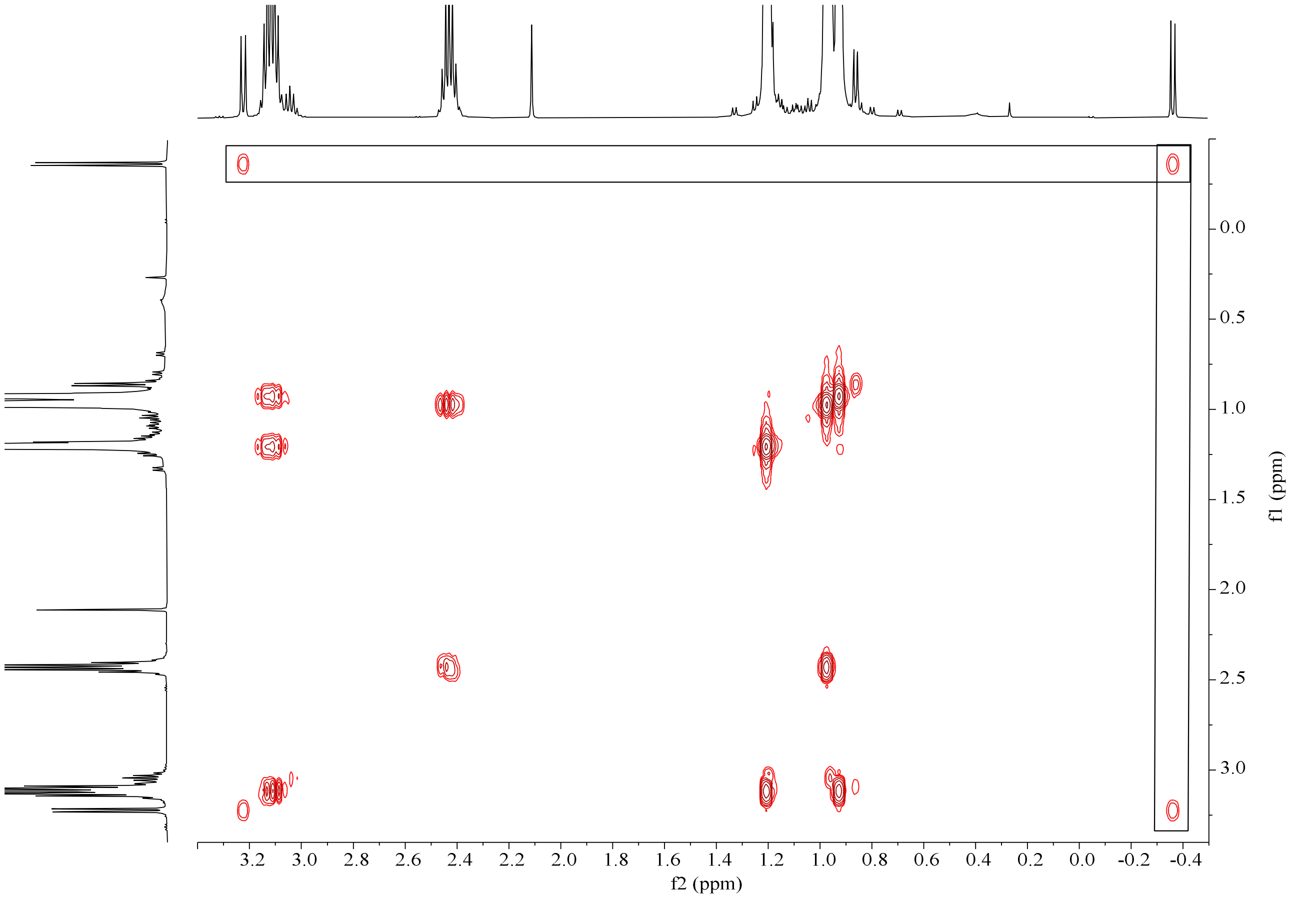

Figure S101. ^{1}H-^{1}H COSY NMR spectrum of *in-situ* generated [{(^{iPrDip}nacnac)Mg}$_2$(μ-H)(μ-OH)] **4c** (chemical range: −0.4-3.3 ppm). The coupling between the hydroxide and hydride units is highlighted.

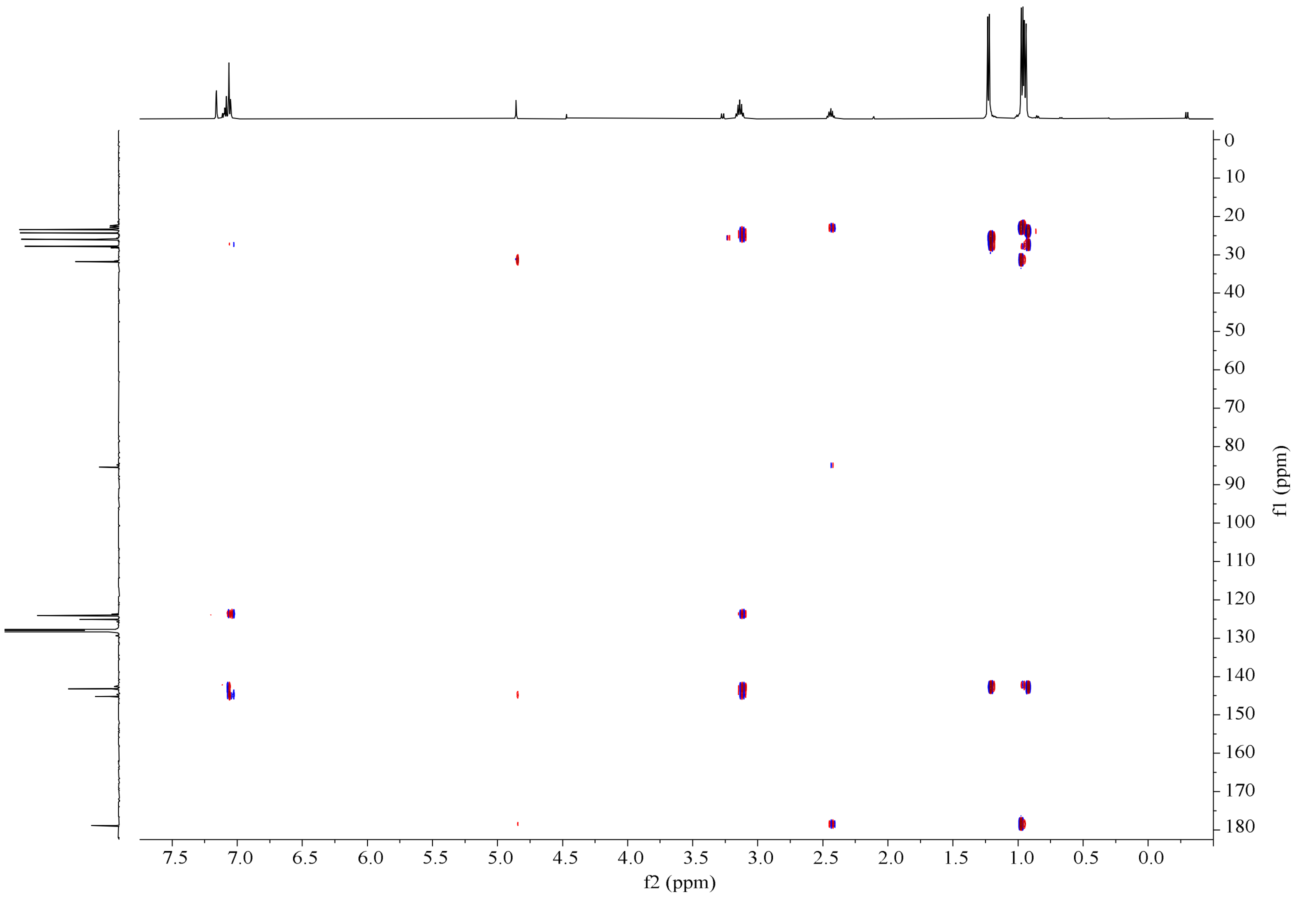

Figure S102. ^{1}H-^{13}C HMBC NMR spectrum of *in-situ* generated [{(^{iPrDip}nacnac)Mg}$_2$(μ-H)(μ-OH)] **4c**.

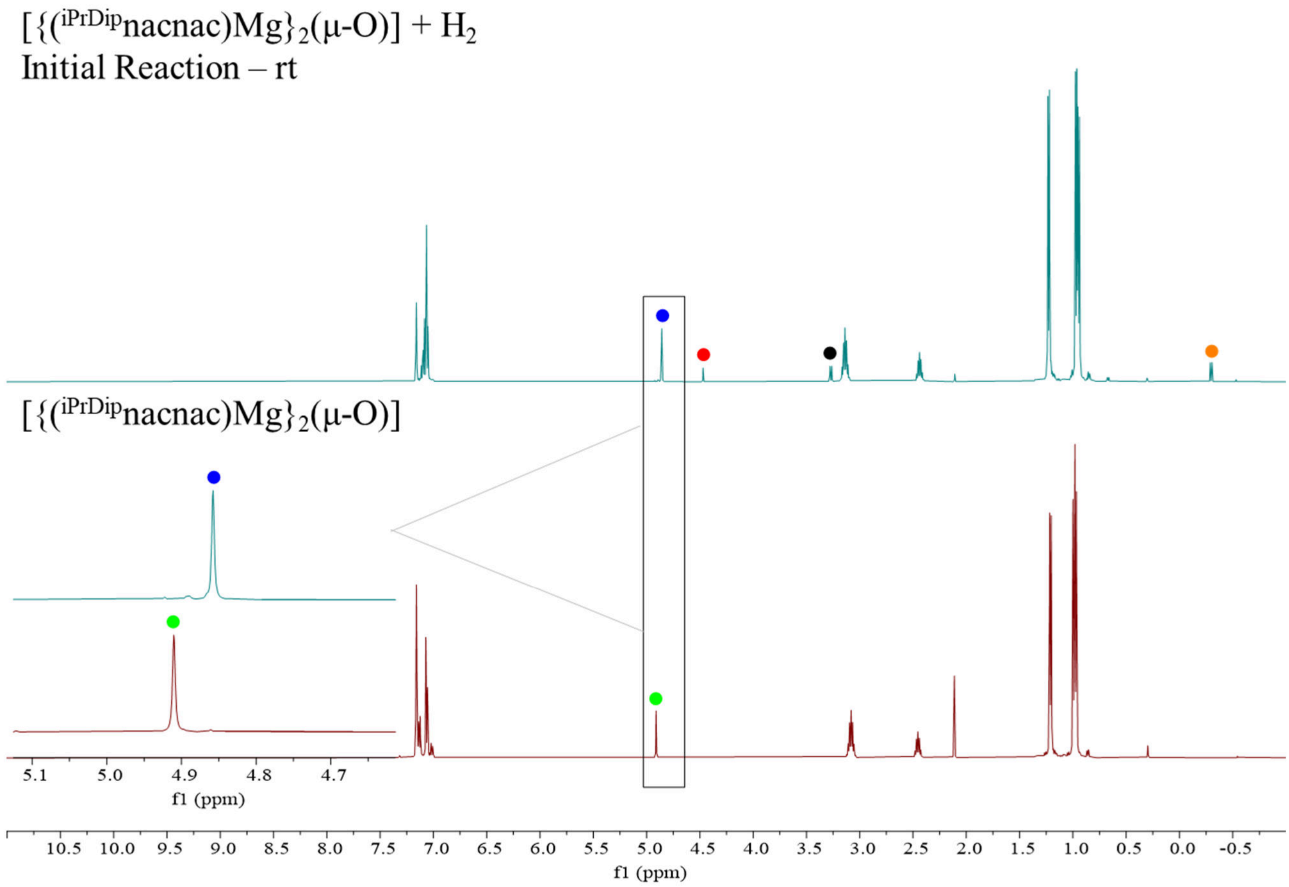

Figure S103. Stacked ^{1}H spectra of the reaction of a colourless *in-situ* generated solution of [{(^{iPrDip}nacnac)Mg}$_2$(μ-O)] **1c** (10.0 mg, 9.88 μmol) and dihydrogen (ca. 1 bar) in C_6D_6 (0.5 mL) at room temperature (rt) in a J Young NMR tube. The red circle denotes the resonance associated with dihydrogen. The green circle denotes the resonance associated with the backbone-C*H* of [{(^{iPrDip}nacnac)Mg}$_2$(μ-O)] **1c**. The blue circle denotes the resonance associated with the backbone-C*H* of [{(^{iPrDip}nacnac)Mg}$_2$(μ-H)(μ-OH)] **4c**. The black circle denotes the chemical resonance associated with Mg-*H* of [{(^{iPrDip}nacnac)Mg}$_2$(μ-H)(μ-OH)] **4c**. The orange circle denotes the chemical resonance associated with Mg-O*H* of [{(^{iPrDip}nacnac)Mg}$_2$(μ-H)(μ-OH)] **4c**.

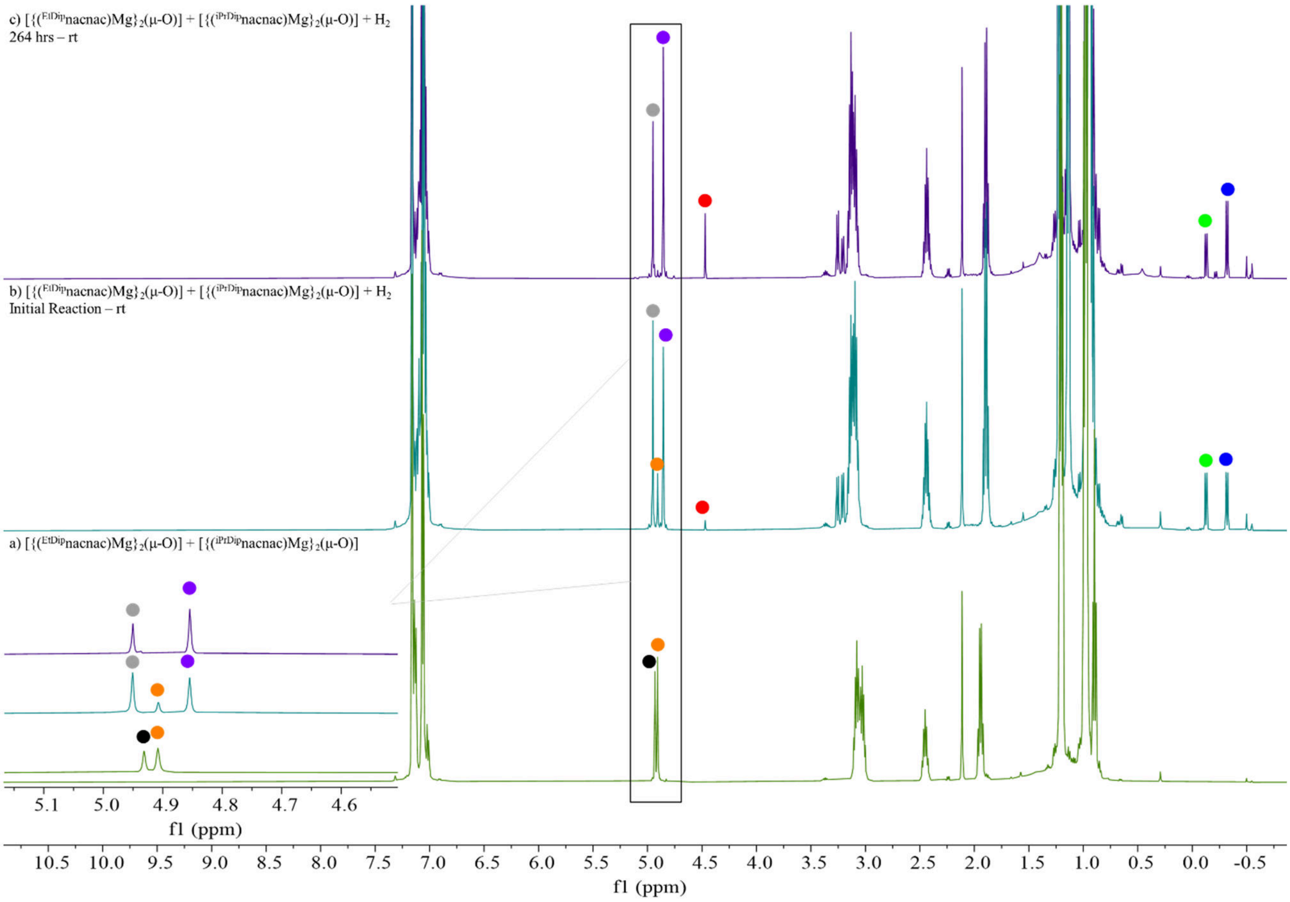

Figure S104. Stacked ^{1}H NMR spectra (500.1 MHz, C_6D_6, 298 K) of the reaction of a colourless *in-situ* generated solution of [{(^{EtDip}nacnac)Mg}$_2$(μ-O)] **1b** (7.9 mg, 8.4 μmol, 1.0 equiv.) and [{(^{iPrDip}nacnac)Mg}$_2$(μ-O)] **1c** (9.0 mg, 9.0 μmol, 1.1 equiv.) in C_6D_6 (0.6 mL) with dihydrogen (ca. 1 bar) at room temperature (rt). The red circle denotes the resonance associated with dihydrogen. The orange circle denotes the resonance associated with the backbone-C*H* of [{(^{iPrDip}nacnac)Mg}$_2$(μ-O)] **1c**. The black circle denotes the resonance associated with the backbone-C*H* of [{(^{EtDip}nacnac)Mg}$_2$(μ-O)] **1b**. The purple circle denotes the resonance associated with the backbone-C*H* of [{(^{iPrDip}nacnac)Mg}$_2$(μ-H)(μ-OH)] **4c**. The grey circle denotes the resonance associated with the backbone-C*H* of [{(^{EtDip}nacnac)Mg}$_2$(μ-H)(μ-OH)] **4b**. The blue circle denotes the resonance associated with Mg-(O*H*) of [{(^{iPrDip}nacnac)Mg}$_2$(μ-H)(μ-OH)] **4c**. The green circle denotes the resonance associated with Mg-(O*H*) of [{(^{EtDip}nacnac)Mg}$_2$(μ-H)(μ-OH)] **4b**.

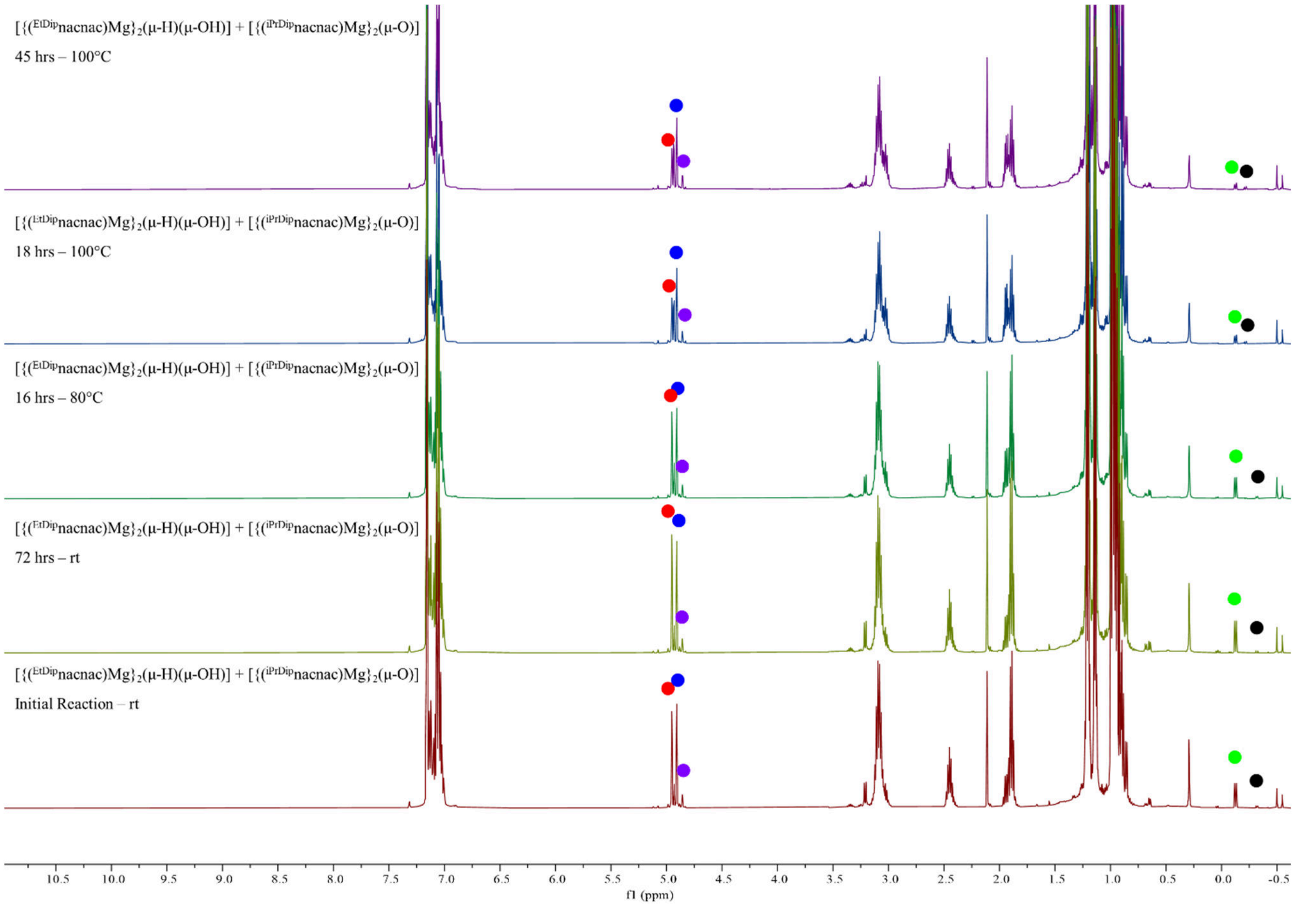

Figure S105. Stacked ^{1}H NMR spectra (500.1 MHz, C_6D_6, 298 K) of the reaction of a solution of [{(^{EtDip}nacnac)Mg}$_2$(μ-H)(μ-OH)] **4b** (11.2 mg, 11.7 µmol, 1.0 equiv.) and [{(^{iPrDip}nacnac)Mg}$_2$(μ-O)] **1c** (14.1 mg, 13.9 µmol, 1.2 equiv.) in C_6D_6 (0.8 mL). The red circle denotes the resonance associated with the backbone-C*H* of [{(^{EtDip}nacnac)Mg}$_2$(μ-H)(μ-OH)] **4b**. The blue circle denotes the resonance associated with the backbone-C*H* of [{(^{iPrDip}nacnac)Mg}$_2$(μ-O)] **1c**. The purple circle denotes the resonance associated with the backbone-C*H* of [{(^{iPrDip}nacnac)Mg}$_2$(μ-H)(μ-OH)] **4c**. The green circle denotes the resonance associated with Mg-O*H* of [{(^{EtDip}nacnac)Mg}$_2$(μ-H)(μ-OH)] **4b**. The black circle denotes the resonance associated with Mg-O*H* of [{(^{iPrDip}nacnac)Mg}$_2$(μ-H)(μ-OH)] **4c**.

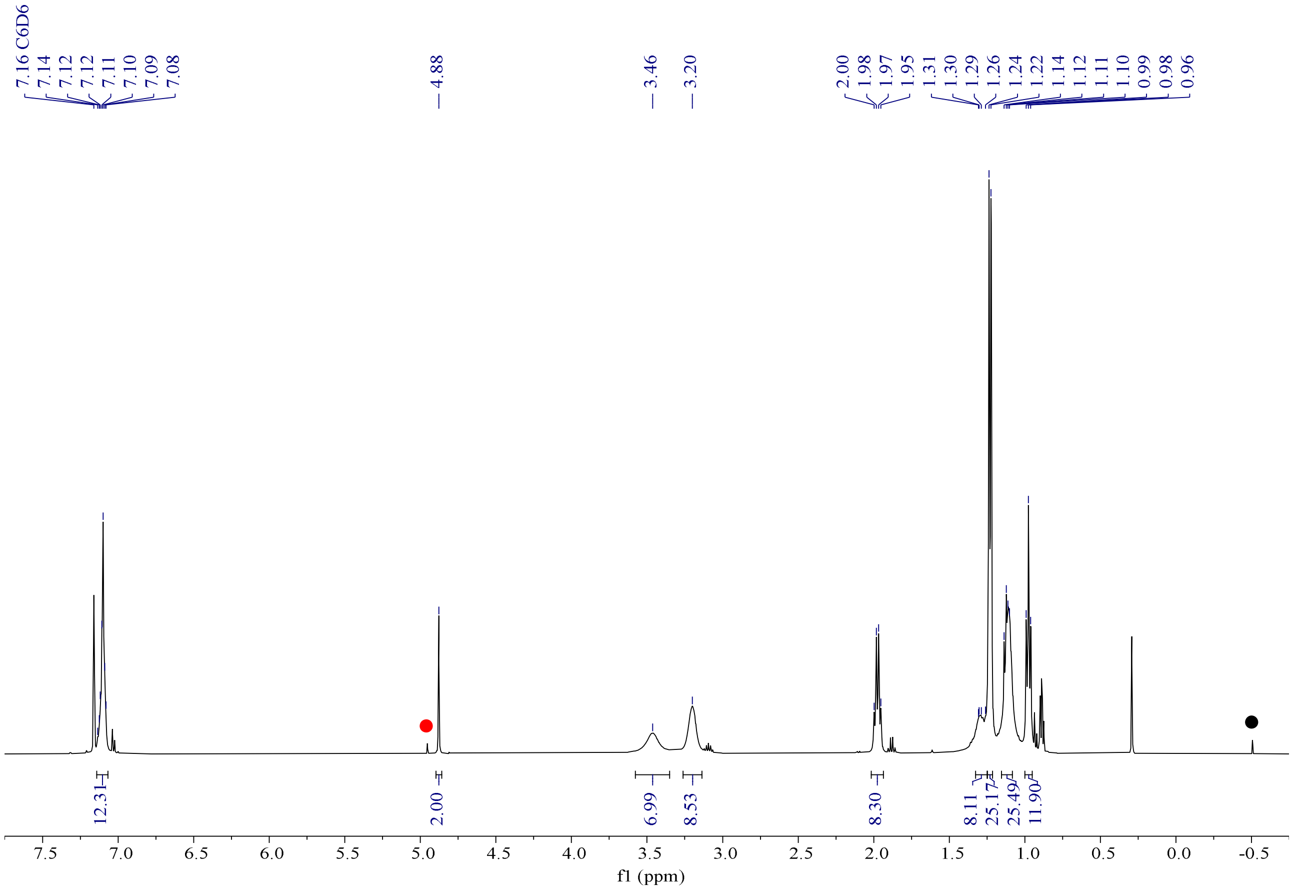

Figure S106. ^{1}H NMR spectrum (500.1 MHz, C_6D_6, 298 K) of isolated [{(^{EtDip}nacnac)Mg(THF)}$_2$(μ-O)] **5b**. The black circle denotes the resonance associated with Mg-(O*H*)$_2$ of [{(^{EtDip}nacnac)Mg(μ-OH)}$_2$] **3b**. The red circle denotes the resonance associated with the backbone-C*H* of [{(^{EtDip}nacnac)Mg(μ-OH)}$_2$] **3b**.

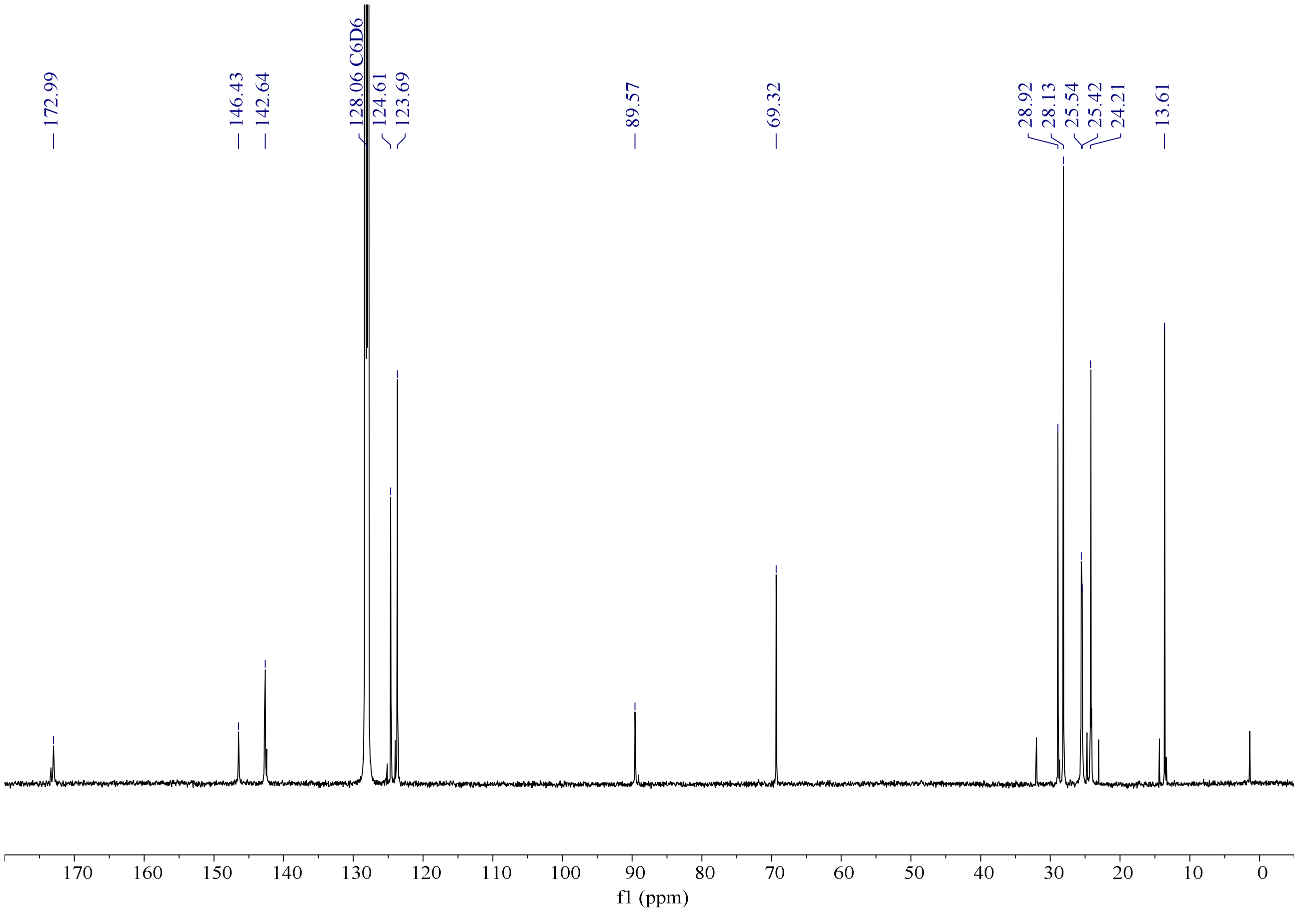

Figure S107. $^{13}C\{^{1}H\}$ NMR spectrum (125.8 MHz, C_6D_6, 298 K) of isolated [{(^{EtDip}nacnac)Mg(THF)}$_2$(μ-O)] **5b**.

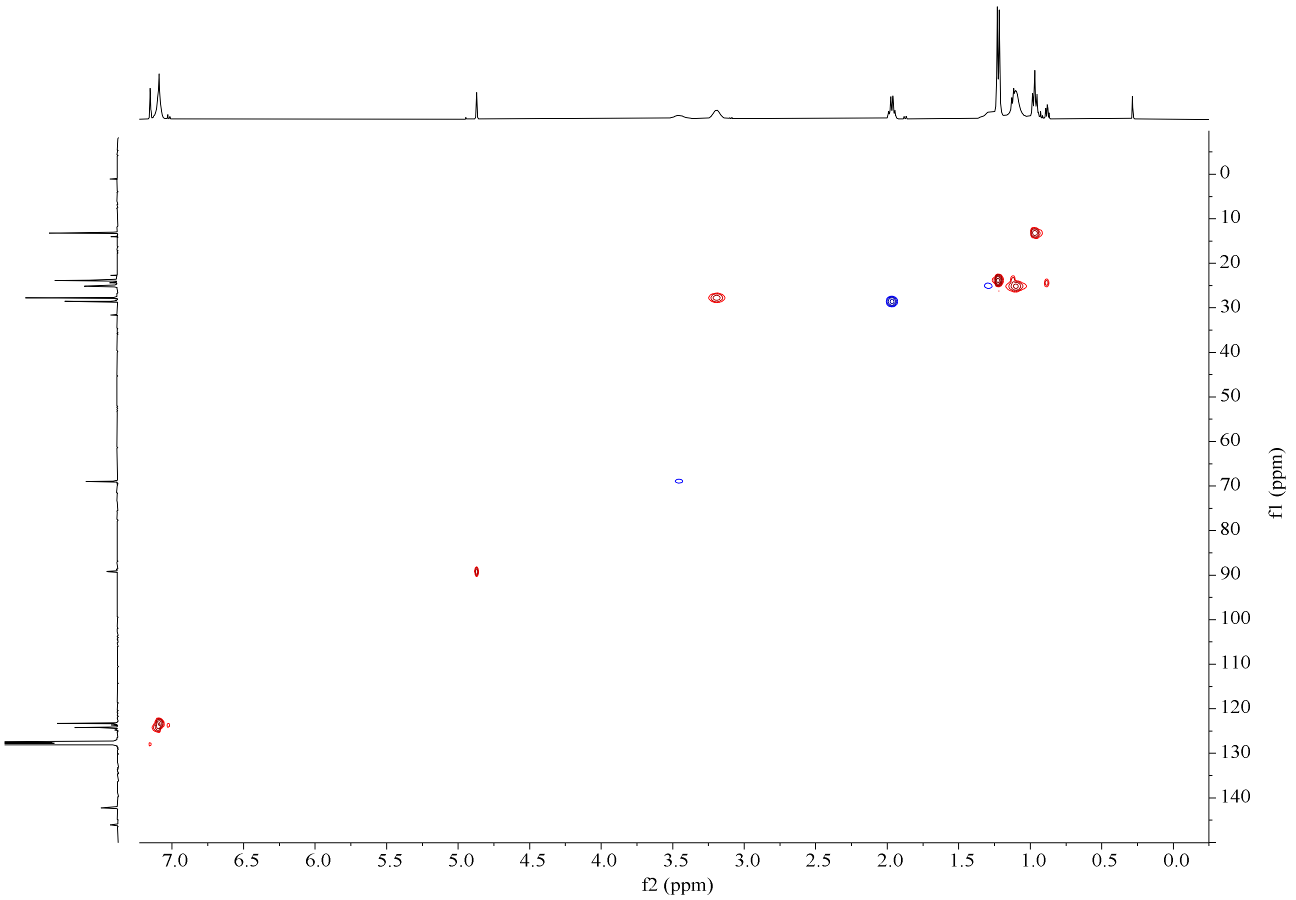

Figure S108. ^{1}H-^{13}C HSQC NMR spectrum of isolated [{(^{EtDip}nacnac)Mg(THF)}$_2$(μ-O)] **5b**.

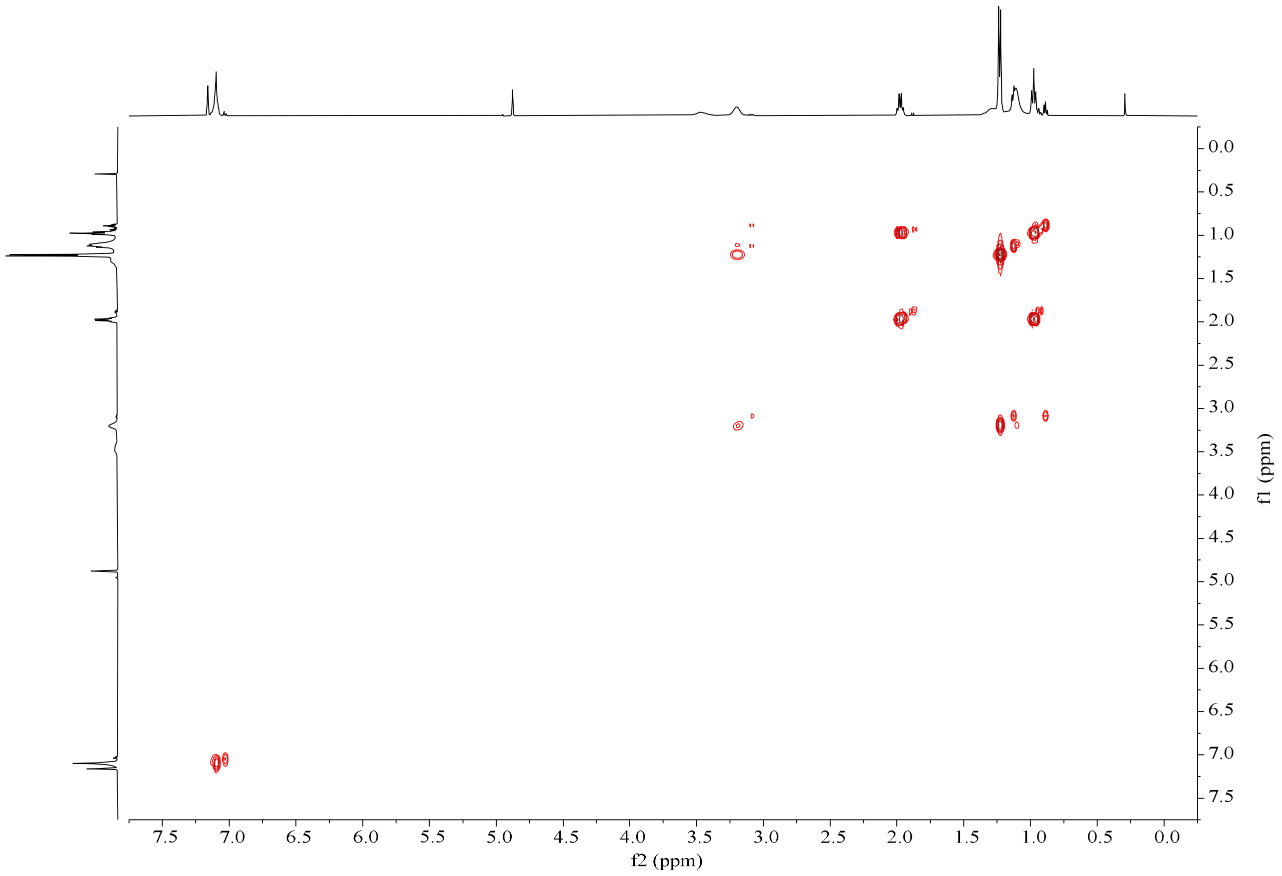

Figure S109. ^{1}H-^{1}H COSY NMR spectrum of isolated [{(^{EtDip}nacnac)Mg(THF)}$_2$(μ-O)] **5b**.

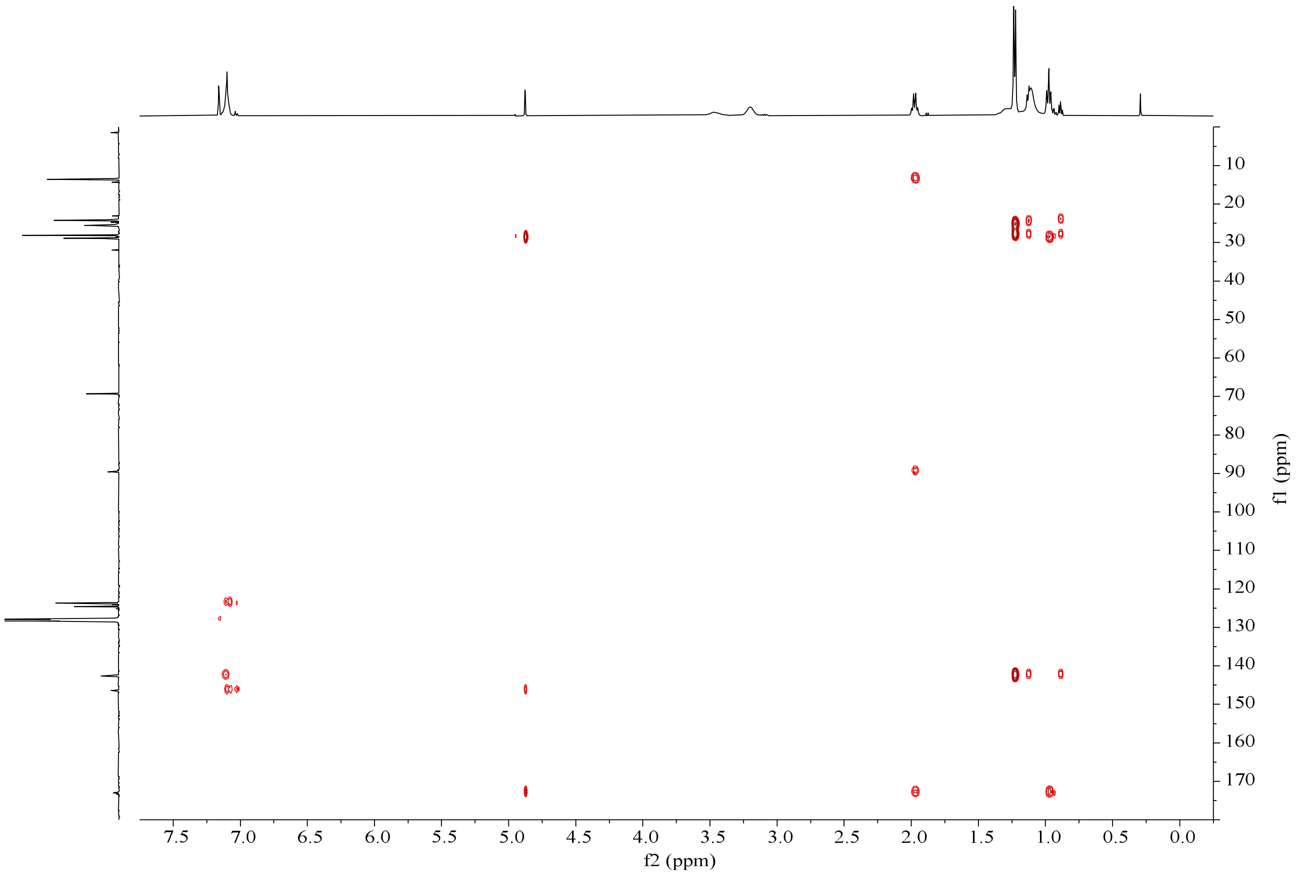

Figure S110. ^{1}H-^{13}C HMBC NMR spectrum of isolated [{(^{EtDip}nacnac)Mg(THF)}$_2$(μ-O)] **5b**.

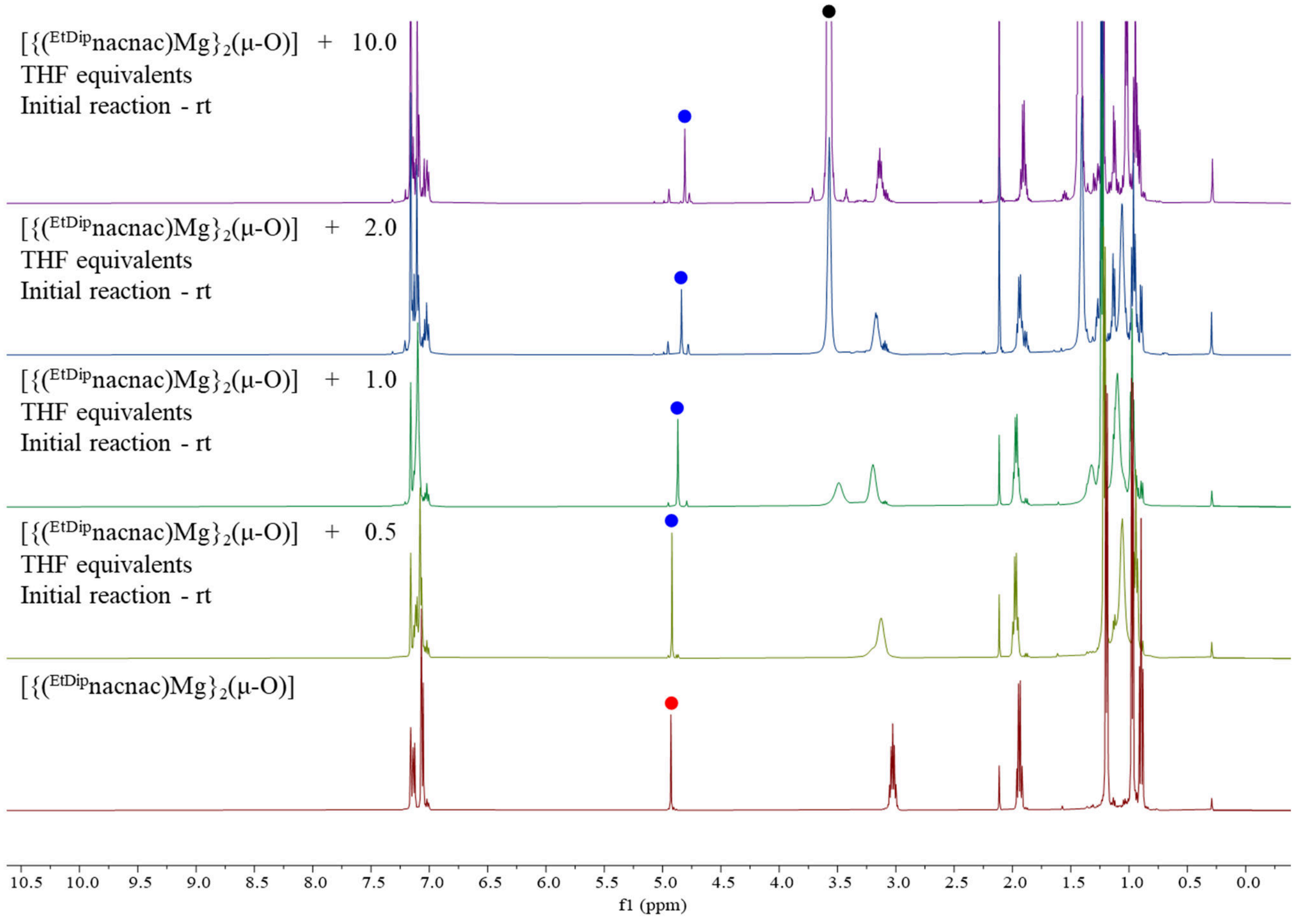

Figure S111. Stacked ^{1}H NMR spectra (499.9 MHz, C_6D_6, 298 K) from the titration of a colourless *in-situ* generated solution of [{(^{EtDip}nacnac)Mg}$_2$(μ-O)] **1b** (19.5 mg, 20.4 μmol, 1.0 equiv.) in C_6D_6 (0.5 mL) with THF (3.4 μL, 10.2 μmol, 0.5 equiv., from a 3 M THF stock solution in C_6D_6) at room temperature (rt) in a J Young NMR tube. The red circle denotes the resonance associated with the backbone-C*H* of [{(^{EtDip}nacnac)Mg}$_2$(μ-O)] **1b**. The blue circle denotes the resonance associated with the backbone-C*H* of [{(^{EtDip}nacnac)Mg(THF)}$_2$(μ-O)] **5b**. The black circle denotes the resonance associated with O(C*H*$_2$) of THF.

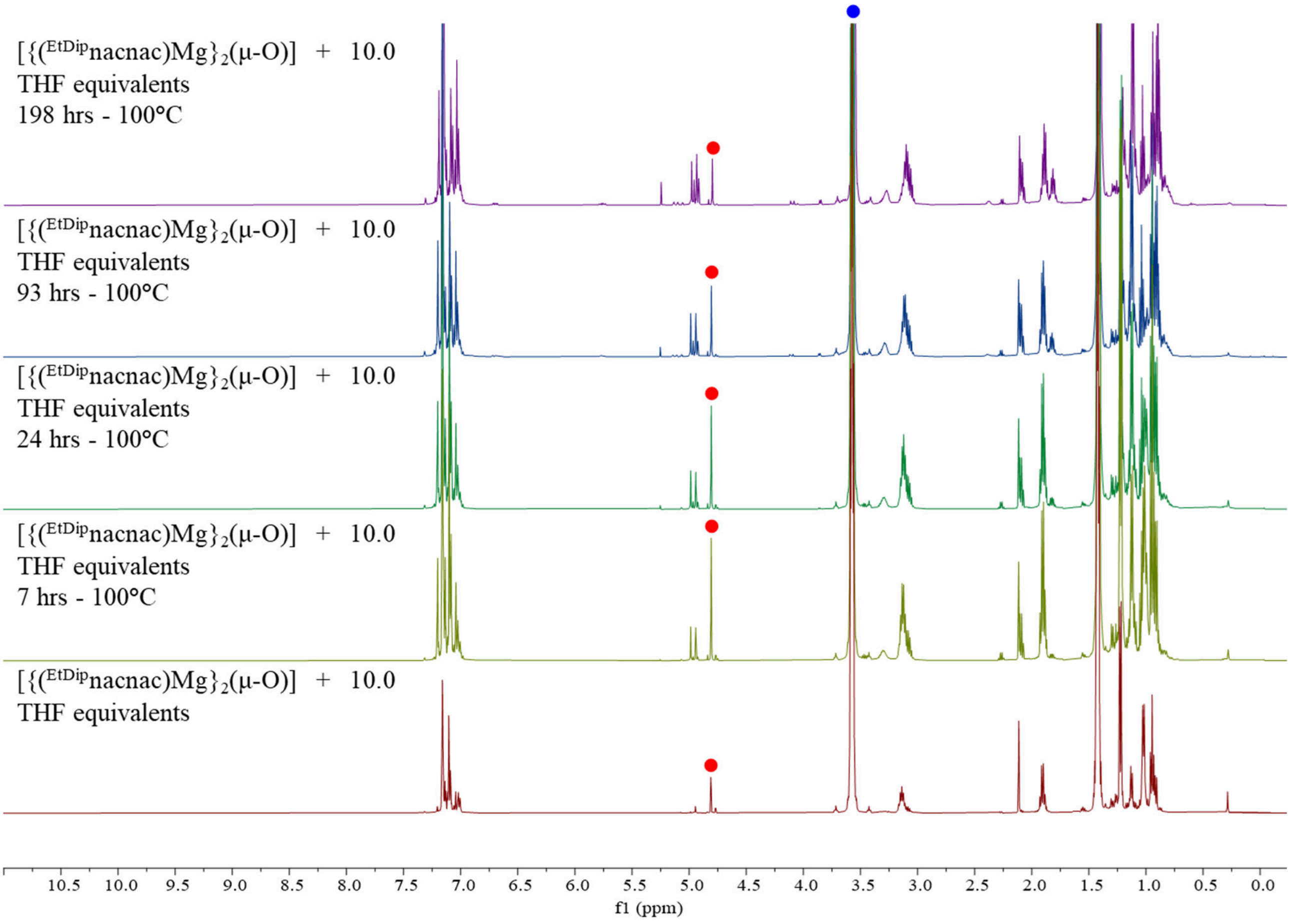

Figure S112. Stacked ^{1}H NMR spectra (499.9 MHz, C_6D_6, 298 K) of the decomposition reaction of a colourless *in-situ* generated solution of [{(^{EtDip}nacnac)Mg(THF)}$_2$(μ-O)] **5b** (22.4 mg, 20.4 μmol) in C_6D_6 (0.5 mL) in a J Young NMR tube. The red circle denotes the resonance associated with the backbone-C*H* of [{(^{EtDip}nacnac)Mg(THF)}$_2$(μ-O)] **5b**. The blue circle denotes the resonance associated with O(CH_2) of THF.

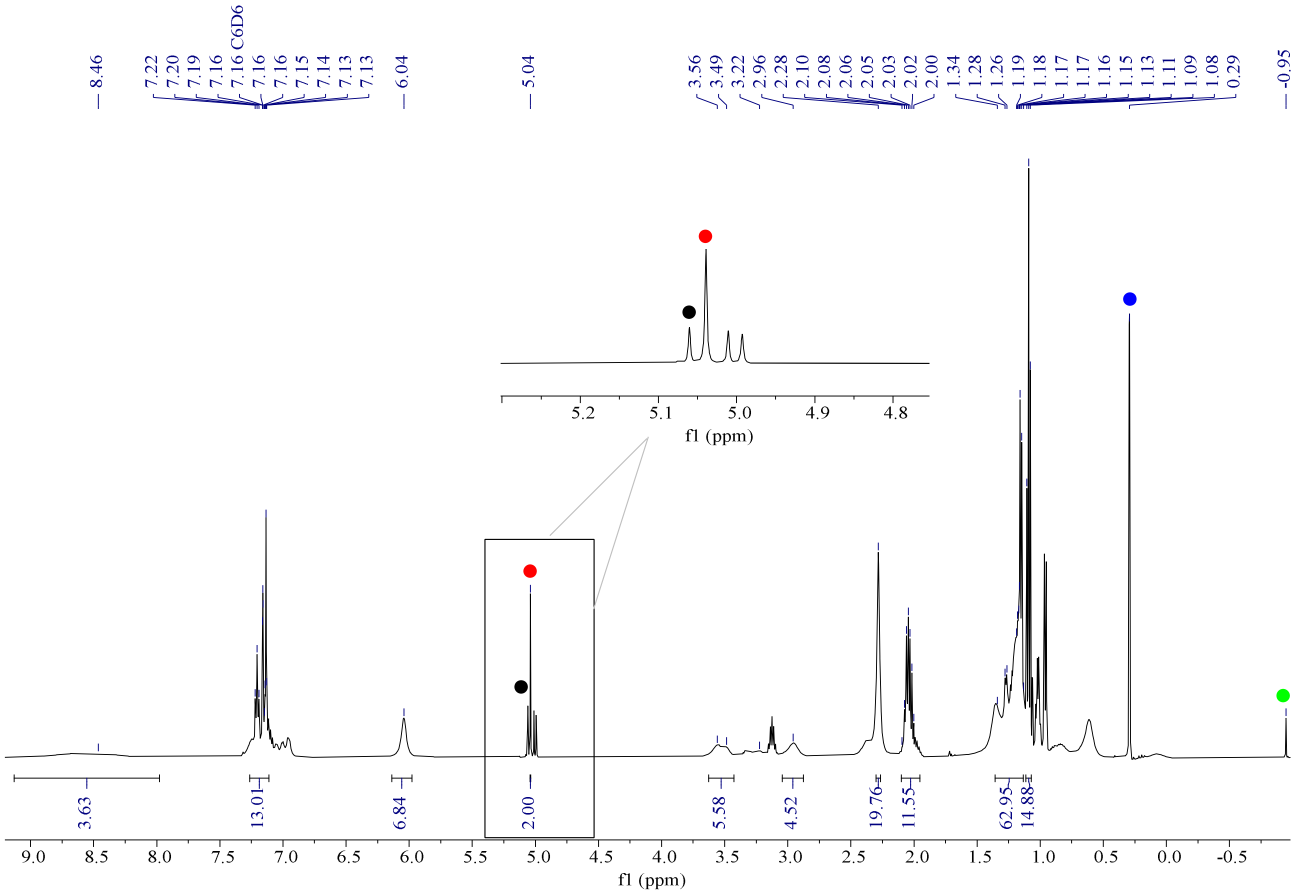

Figure S113. ^{1}H NMR spectrum (499.9 MHz, C_6D_6, 298 K) of isolated [{(^{EtDip}nacnac)Mg(DMAP)}$_2$(μ-O)] **6b**. The blue circle denotes the resonance associated with silicone grease. The red circle denotes the resonance associated with the backbone-C*H* of [{(^{EtDip}nacnac)Mg(DMAP)}$_2$(μ-O)] **6b**. The black circle denotes the resonance associated with the backbone-C*H* of [{(^{EtDip}nacnac)Mg(DMAP)(μ-OH)}$_2$] **8b**. The green circle denotes the resonance associated with Mg-(O*H*)$_2$ of [{(^{EtDip}nacnac)Mg(DMAP)(μ-OH)}$_2$] **8b**.

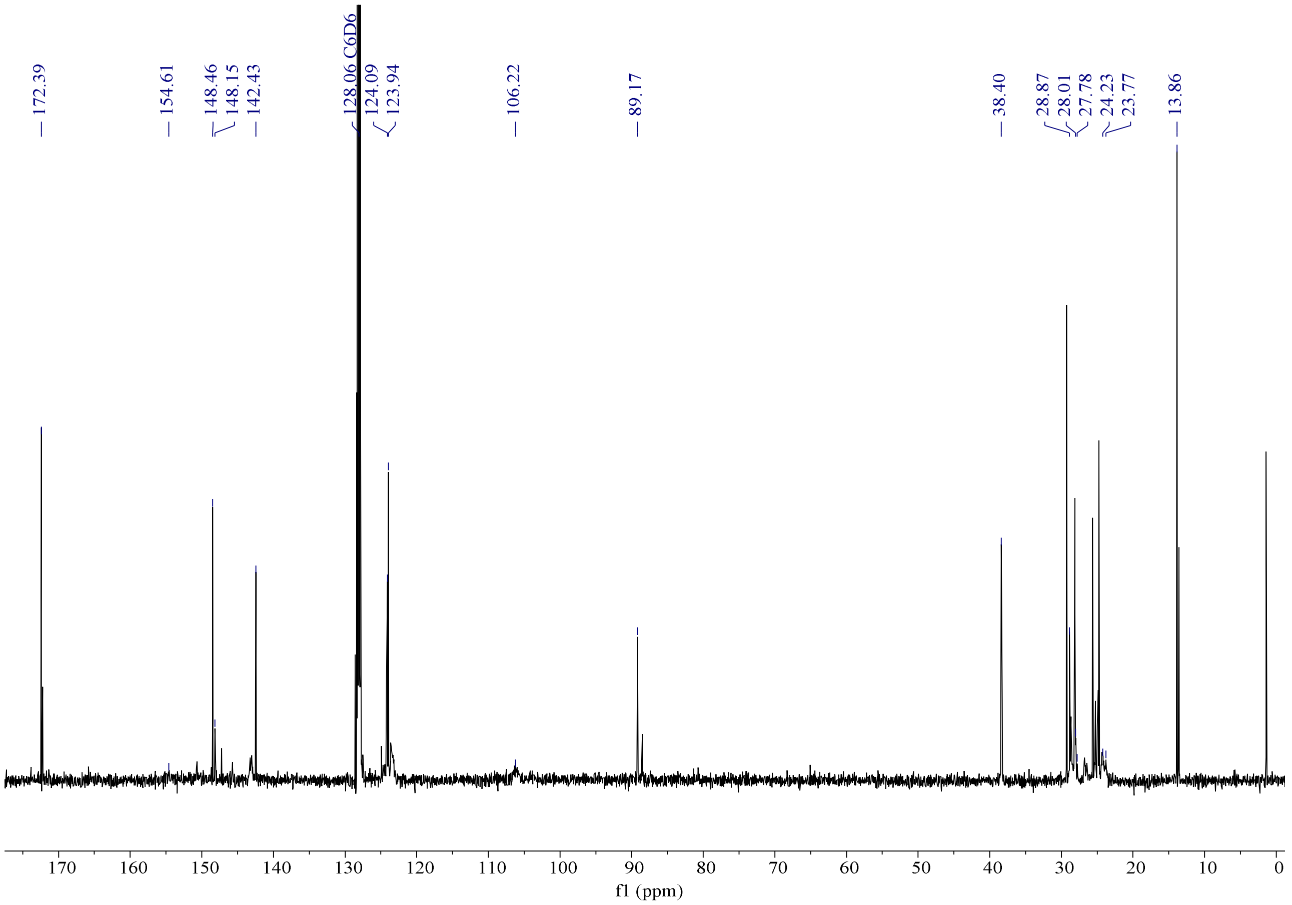

Figure S114. $^{13}C\{^{1}H\}$ NMR (125.7 MHz, C_6D_6, 298 K) spectrum of isolated $[\{(^{EtDip}nacnac)Mg(DMAP)\}_2(\mu\text{-}O)]$ **6b**.

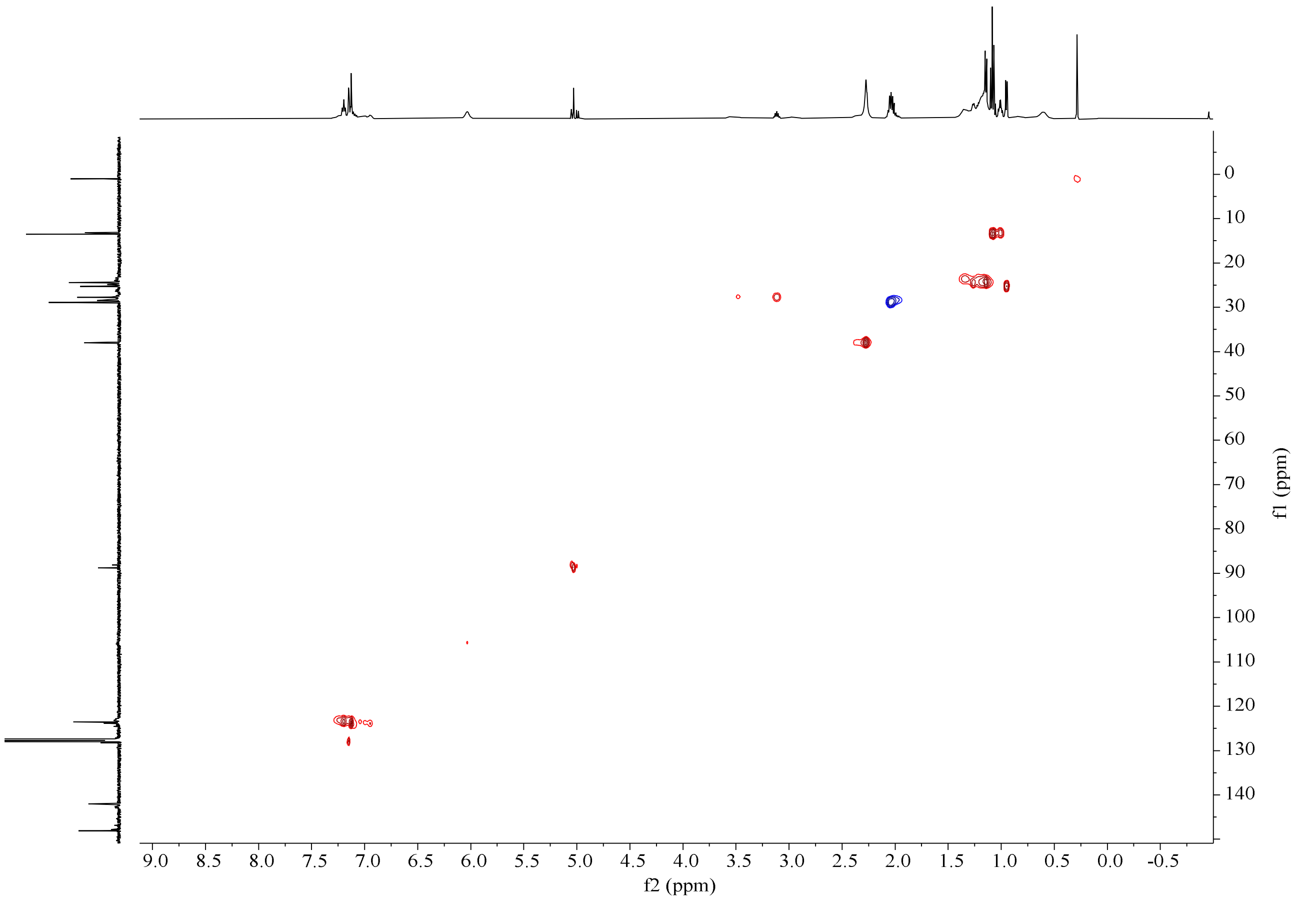

Figure S115. ^{1}H-^{13}C HSQC NMR spectrum of isolated [{(^{EtDip}nacnac)Mg(DMAP)}$_2$(μ-O)] **6b**.

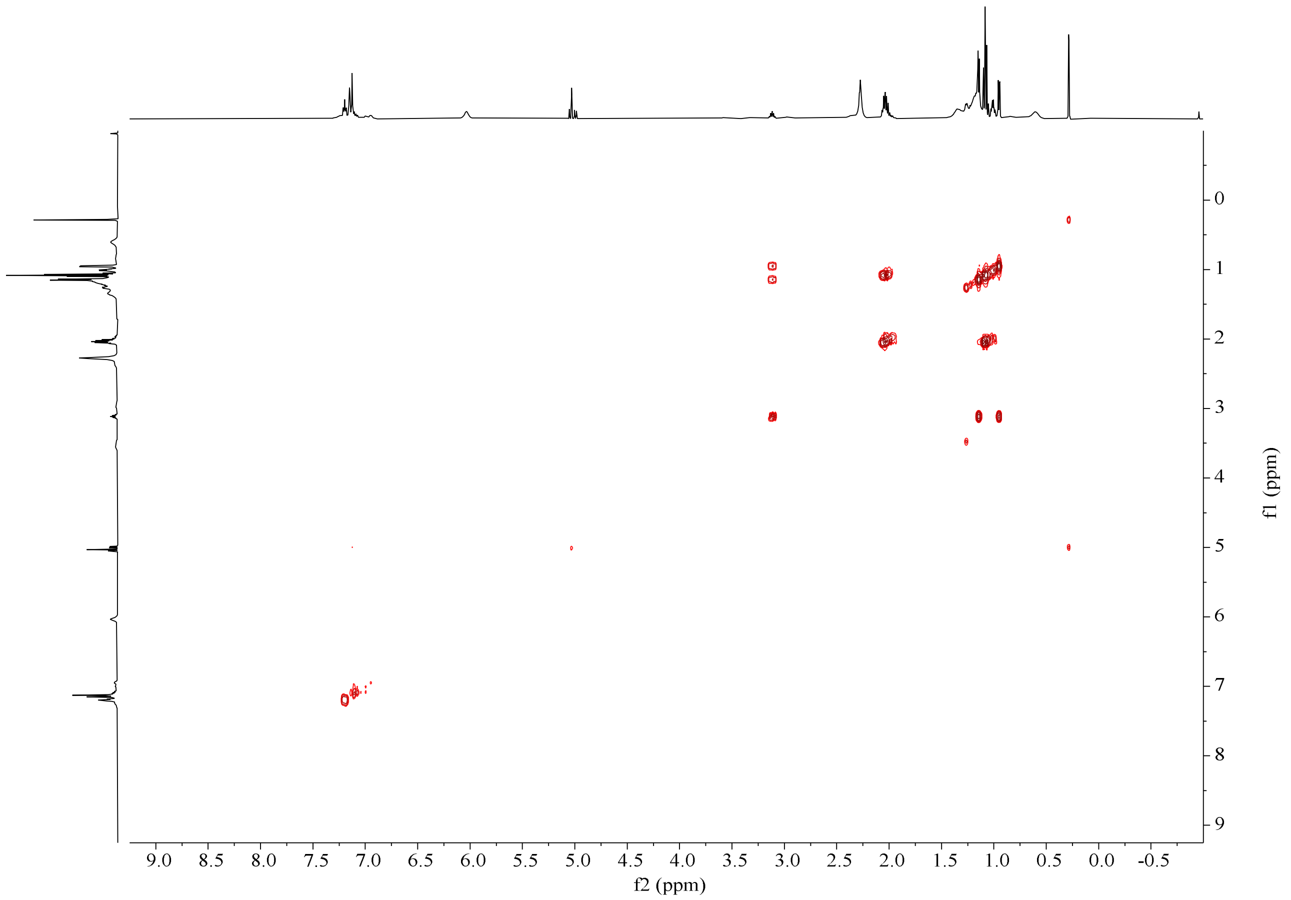

Figure S116. ^{1}H-^{1}H COSY NMR spectrum of isolated [{(^{EtDip}nacnac)Mg(DMAP)}$_2$(μ-O)] **6b**.

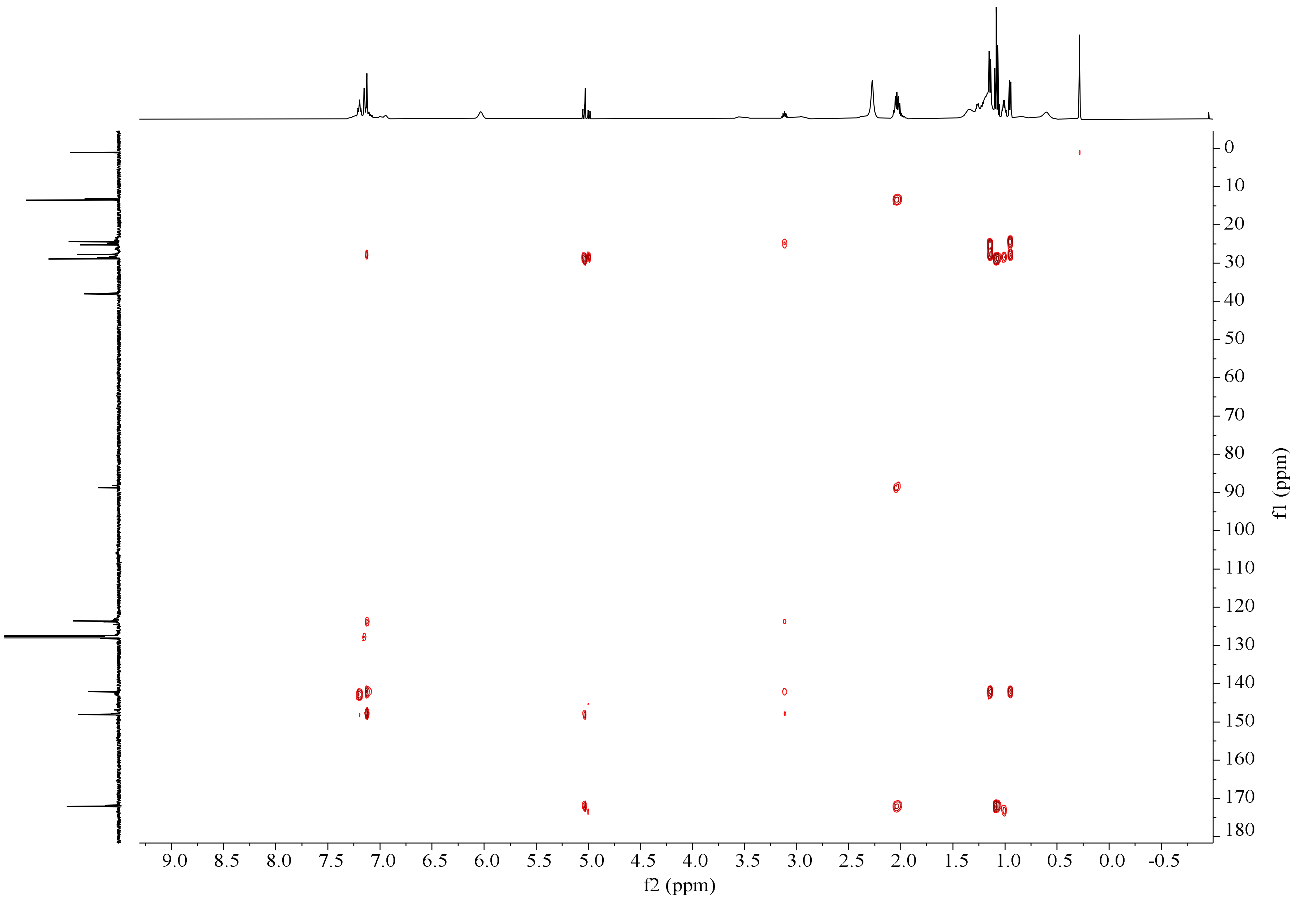

Figure S117. ^{1}H-^{13}C HMBC NMR spectrum of isolated [{(^{EtDip}nacnac)Mg(DMAP)}$_2$(μ-O)] **6b**.

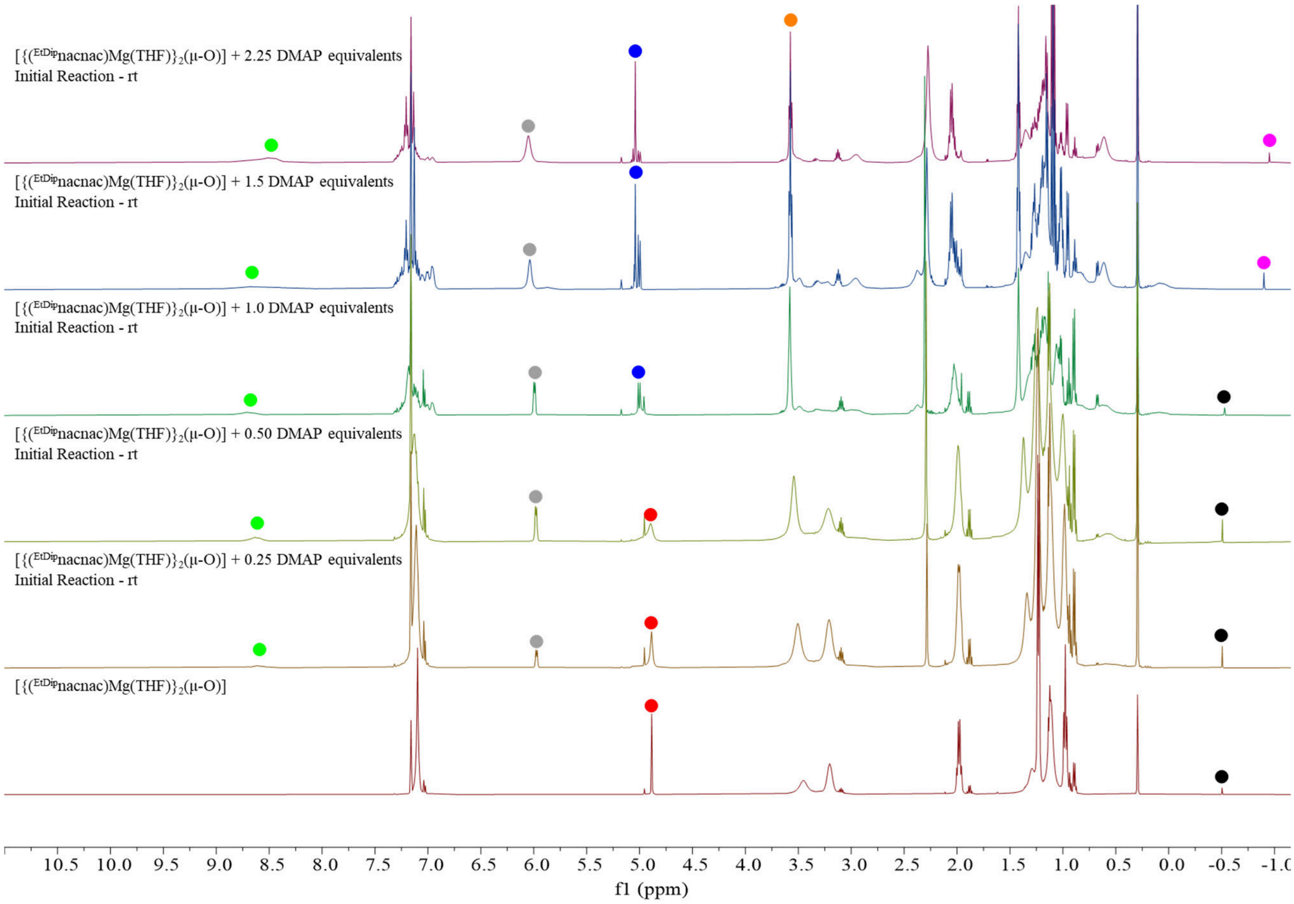

Figure S118. Stacked ^{1}H NMR spectra (499.9 MHz, C_6D_6, 298 K) from the titration reaction of a colourless solution of [{(^{EtDip}nacnac)Mg(THF)}$_2$(μ-O)] **5b** (13.5 mg, 12.3 μmol, 1.0 equiv.) in C_6D_6 (0.5 mL) with DMAP (18 μL, 3.0 μmol, 0.25 equiv., from a 0.165 M DMAP stock solution in C_6D_6) at room temperature (rt) in a J Young NMR tube. The red circle denotes the resonance associated with the backbone-C*H* of [{(^{EtDip}nacnac)Mg(THF)}$_2$(μ-O)] **5b**. The blue circle denotes the resonance associated with the backbone-C*H* of [{(^{EtDip}nacnac)Mg(DMAP)}$_2$(μ-O)] **6b**. The orange circle denotes the resonance associated with O(C*H*$_2$) of uncoordinated THF. The grey and green circles denote the resonance associated with Ar-*H*'s of DMAP. The black circle denotes the resonance associated with Mg-(O*H*)$_2$ of [{(^{EtDip}nacnac)Mg(μ-OH)}$_2$] **3b**. The pink circle denotes the resonance associated with Mg-(O*H*)$_2$ of [{(^{EtDip}nacnac)Mg(DMAP)(μ-OH)]$_2$ **8b**.

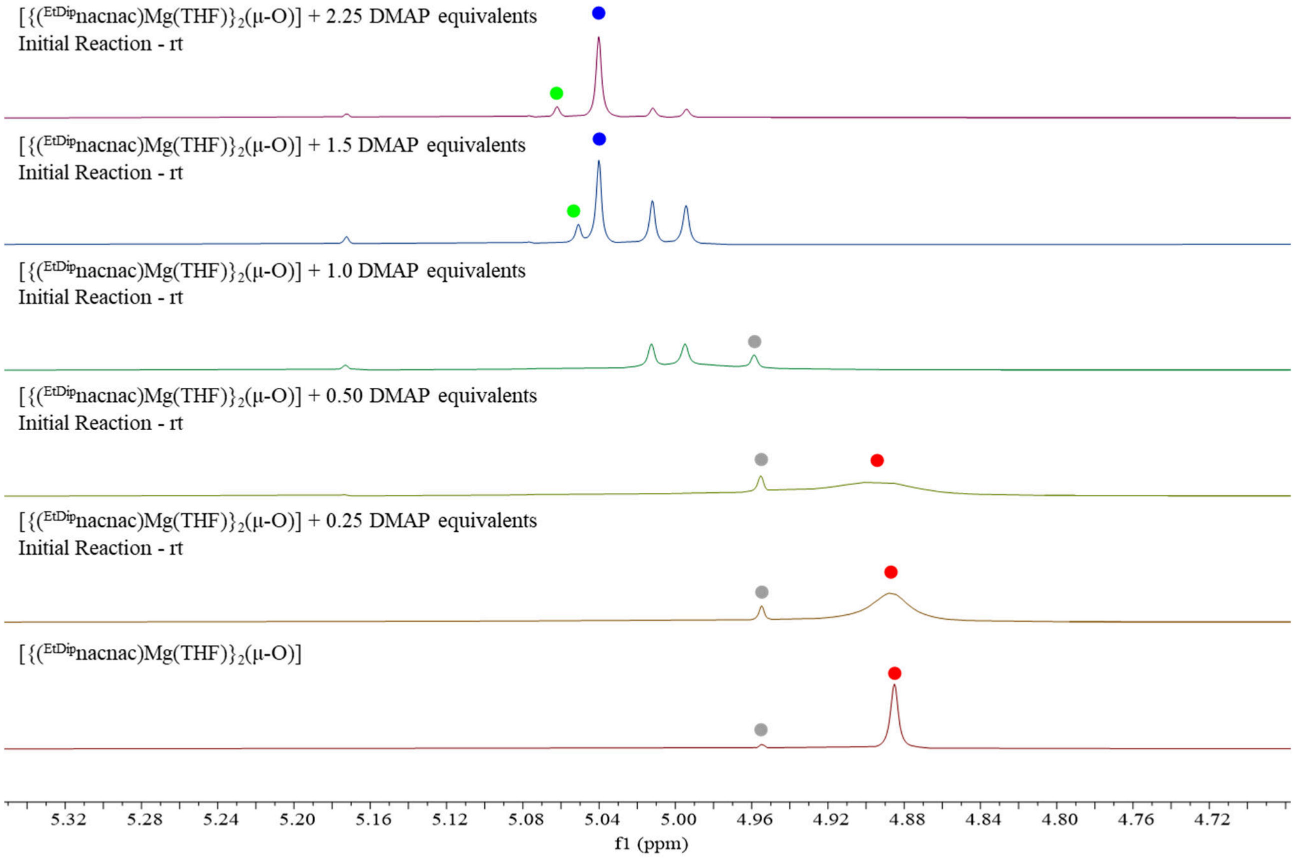

Figure S119. Stacked ^{1}H NMR spectra (499.9 MHz, C_6D_6, 298 K) from the titration reaction of a colourless solution of [{(^{EtDip}nacnac)Mg(THF)}$_2$(μ-O)] **5b** (13.5 mg, 12.3 μmol, 1.0 equiv.) in C_6D_6 (0.5 mL) with DMAP (18 μL, 3.0 μmol, 0.25 equiv., from a 0.165 M DMAP stock solution in C_6D_6) at room temperature (rt) in a J Young NMR tube (chemical range: 4.72-5.32 ppm). The red circle denotes the resonance associated with the backbone-C*H* of [{(^{EtDip}nacnac)Mg(THF)}$_2$(μ-O)] **5b**. The grey circle denotes the resonance associated with the backbone-C*H* of [{(^{EtDip}nacnac)Mg(μ-OH)}$_2$] **3b**. The blue circle denotes the resonance associated with the backbone-C*H* of [{(^{EtDip}nacnac)Mg(DMAP)}$_2$(μ-O)] **6b**. The green circle denotes the resonance associated with the backbone-C*H* of [{(^{EtDip}nacnac)Mg(DMAP)(μ-OH)]$_2$ **8b**.

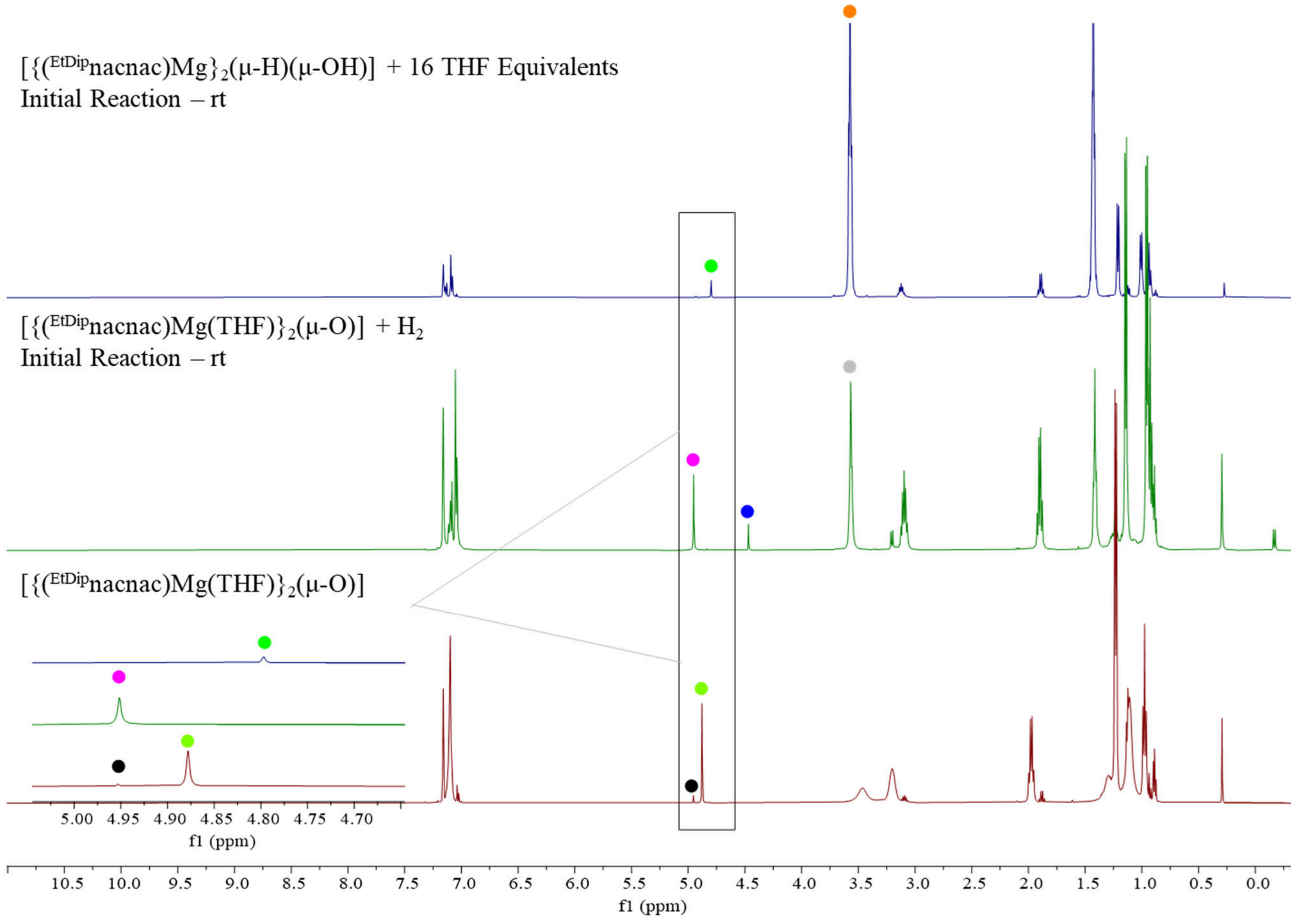

Figure S120. Stacked ^{1}H NMR spectra (500.1 MHz, C_6D_6, 298 K) of the reaction of a solution of [{(^{EtDip}nacnac)Mg(THF)}$_2$(μ-O)] **5b** (15.0 mg, 13.6 μmol, 1.0 equiv.) in C_6D_6 (0.6 mL) with dihydrogen (ca. 1 bar) at room temperature (rt) in a J Young NMR tube followed by the addition of THF (17.7 μL, 218 μmol, 16.0 equiv.) at room temperature. The green circle denotes the resonance associated with the backbone-C*H* of [{(^{EtDip}nacnac)Mg(THF)}$_2$(μ-O)] **5b**. The pink circle denotes the resonance associated with the backbone-C*H* of [{(^{EtDip}nacnac)Mg}$_2$(μ-H)(μ-OH)] **4b**. The blue circle denotes the resonance associated with dihydrogen. The grey circle denotes the resonance associated with O(CH_2) of uncoordinated THF. The orange circle denotes the resonance associated with O(CH_2) of coordinated THF. The black circle denotes the resonance associated with the backbone-C*H* of [{(^{EtDip}nacnac)Mg(μ-OH)}$_2$] **3b**.

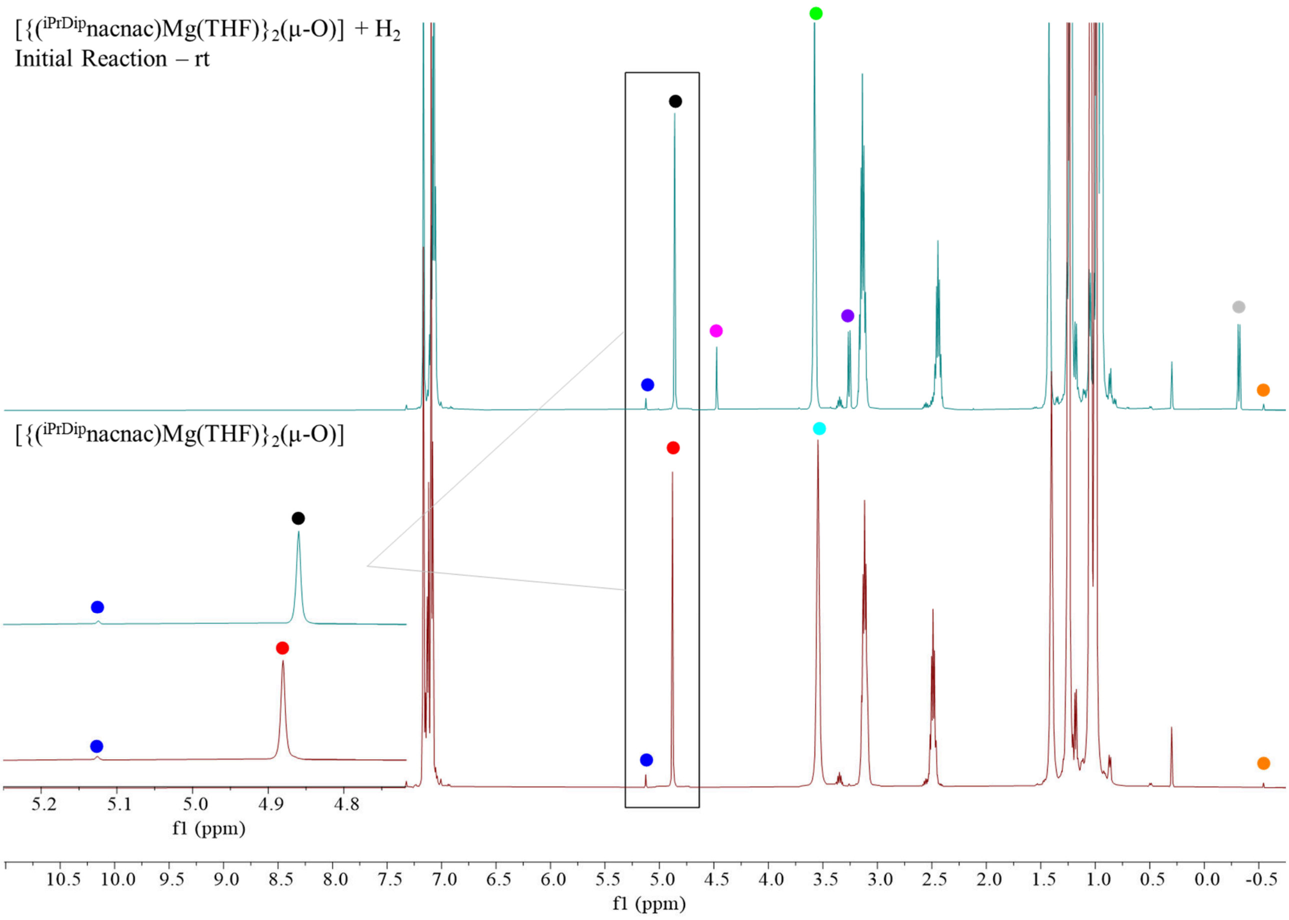

Figure S121. Stacked ^1H NMR spectra (499.9 MHz, C_6D_6, 298 K) of the reaction of a colourless solution of [{(^{iPrDip}nacnac)Mg(THF)}$_2$(μ-O)] **5c** (11.0 mg, 9.51 μmol) and dihydrogen (ca. 1 bar) in C_6D_6 (0.5 mL) at room temperature (rt) in a J Young NMR tube. The pink circle denotes the resonance associated with dihydrogen. The red circle denotes the resonance associated with the backbone-C*H* of [{(^{iPrDip}nacnac)Mg(THF)}$_2$(μ-O)] **5c**. The cyan circle denotes the resonance associated with O(C*H*$_2$) of [{(^{iPrDip}nacnac)Mg(THF)}$_2$(μ-O)] **5c**. The black circle denotes the resonance associated with the backbone-C*H* of [{(^{iPrDip}nacnac)Mg}$_2$(μ-H)(μ-OH)] **4c**. The green circle denotes the resonance associated with O(C*H*$_2$)$_{THF}$ of uncoordinated THF. The purple circle denotes the resonance associated with Mg-*H* of [{(^{iPrDip}nacnac)Mg}$_2$(μ-H)(μ-OH)] **4c**. The grey circle denotes the resonance associated with Mg-O*H* of [{(^{iPrDip}nacnac)Mg}$_2$(μ-H)(μ-OH)] **4c**. The blue circle denotes the resonance associated with the backbone-C*H* of [{(^{iPrDip}nacnac)Mg(μ-OH)}$_2$] **3c**. The orange circle denotes the resonance associated with Mg-(O*H*)$_2$ of [{(^{iPrDip}nacnac)Mg(μ-OH)}$_2$] **3c**.

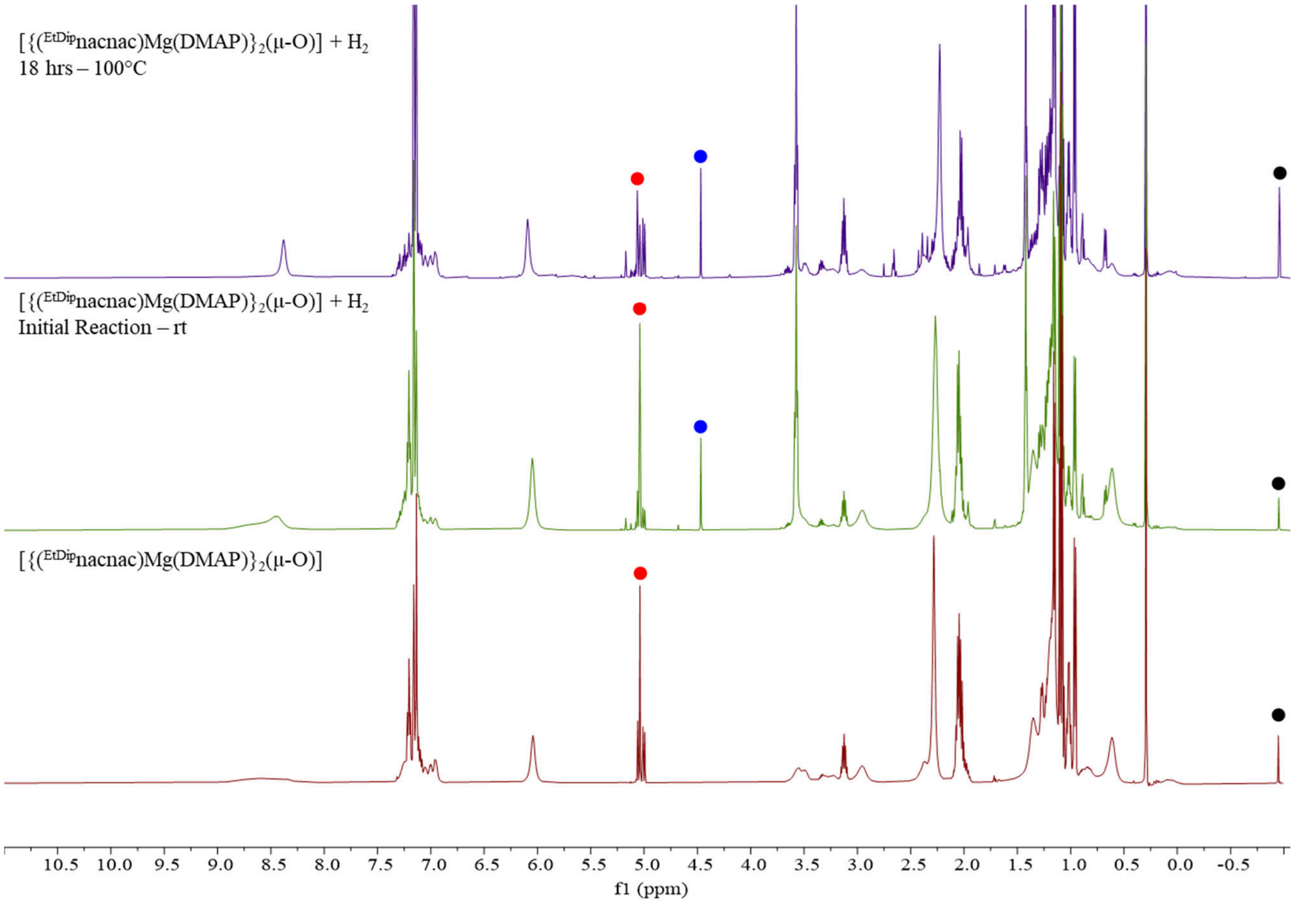

Figure S122. Stacked ^{1}H NMR spectra of the reaction of a colourless solution of [{(^{EtDip}nacnac)Mg(DMAP)}$_2$(μ-O)] **6b** (10.0 mg, 8.33 μmol) in C_6D_6 (0.6 mL) and dihydrogen (ca. 1 bar) in a J Young NMR tube. The blue circle denotes the resonance associated with dihydrogen. The red circle denotes the resonance associated with the backbone-C*H* of [{(^{EtDip}nacnac)Mg(DMAP)}$_2$(μ-O)] **6b**. The black circle denotes the resonance associated with Mg-(O*H*)$_2$ of [{(^{EtDip}nacnac)Mg(DMAP)(μ-OH)}$_2$] **8b**.

2.5 Dehydrogenation experiments

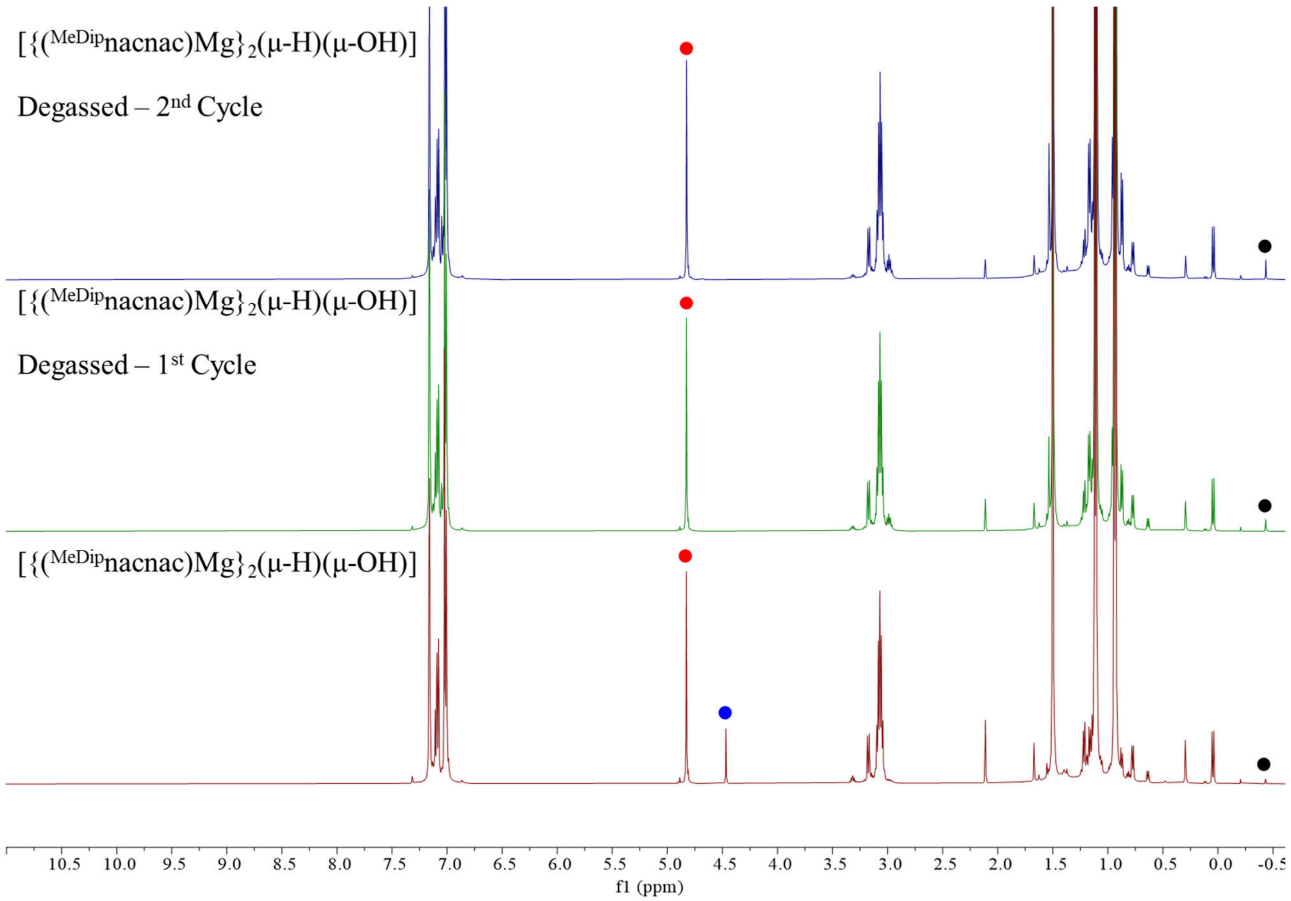

Figure S123. Stacked ^{1}H NMR spectra (499.9 MHz, C_6D_6, 298 K) before and after freeze-pump-thawing a colourless *in-situ* generated solution of [{(^{MeDip}nacnac)Mg}$_2$(μ-H)(μ-OH)] **4a** (12.0 mg, 13.3 μmol) in C_6D_6 (0.5 mL) in a J Young NMR tube. The blue circle denotes the resonance associated with dihydrogen. The red circle denotes the resonance associated with the backbone-C*H* of [{(^{MeDip}nacnac)Mg}$_2$(μ-H)(μ-OH)] **4a**. The black circle denotes the resonance associated with Mg-O*H* of [{(^{MeDip}nacnac)Mg(μ-OH)}$_2$] **3a**.

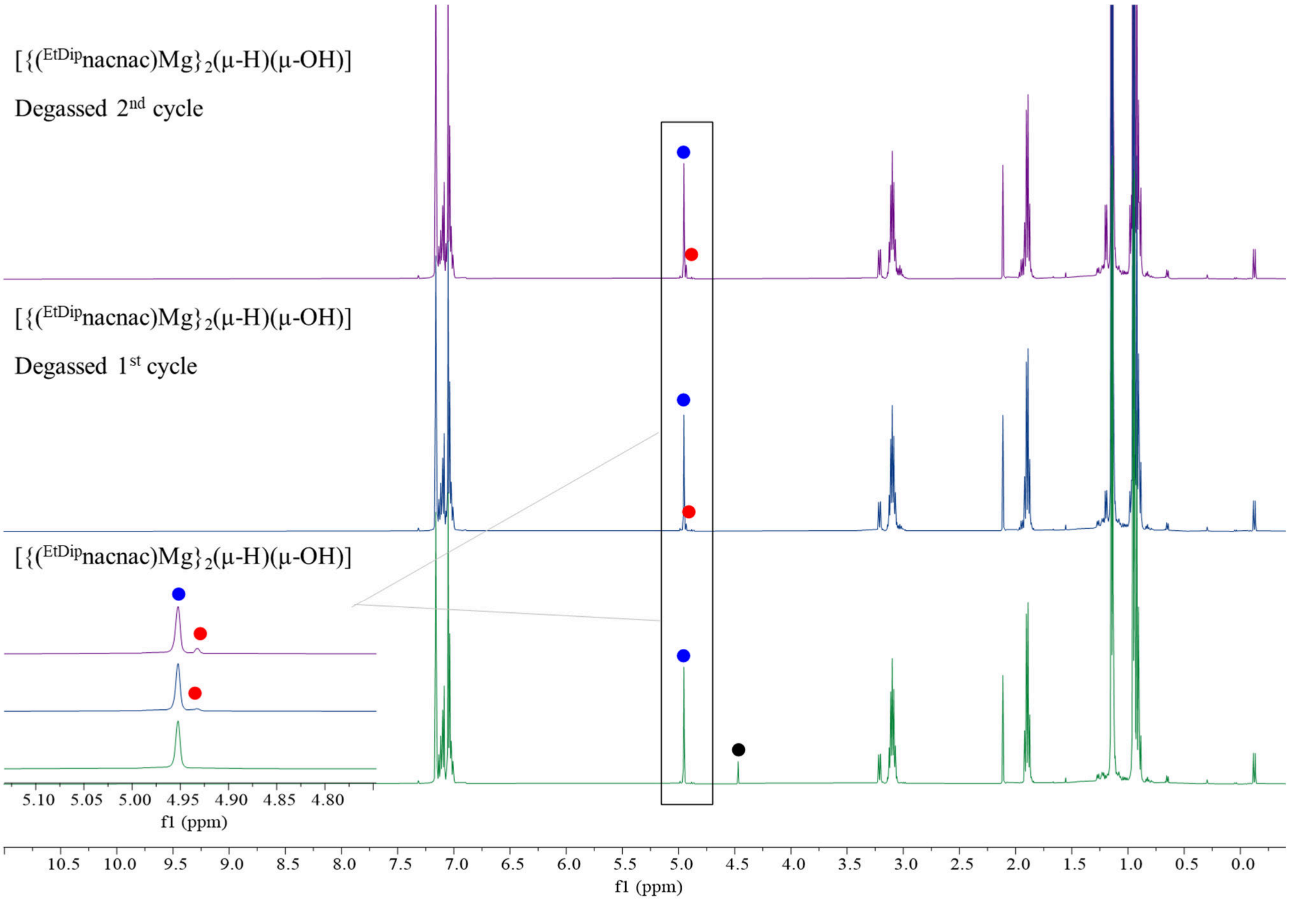

Figure S124. Stacked ^{1}H NMR spectra (500.1 MHz, C_6D_6, 298 K) before and after freeze-pump-thawing a colourless *in-situ* generated solution of [{(^{EtDip}nacnac)Mg}$_2$(μ-H)(μ-OH)] **4b** (10.0 mg, 10.4 μmol) in C_6D_6 (0.5 mL) in a J Young NMR tube. The black circle denotes the resonance associated with dihydrogen. The red circle denotes the resonance associated with the backbone-C*H* of [{(^{EtDip}nacnac)Mg}$_2$(μ-O)] **1b**. The blue circle denotes the resonance associated with the backbone-C*H* of [{(^{EtDip}nacnac)Mg}$_2$(μ-H)(μ-OH)] **4b**.

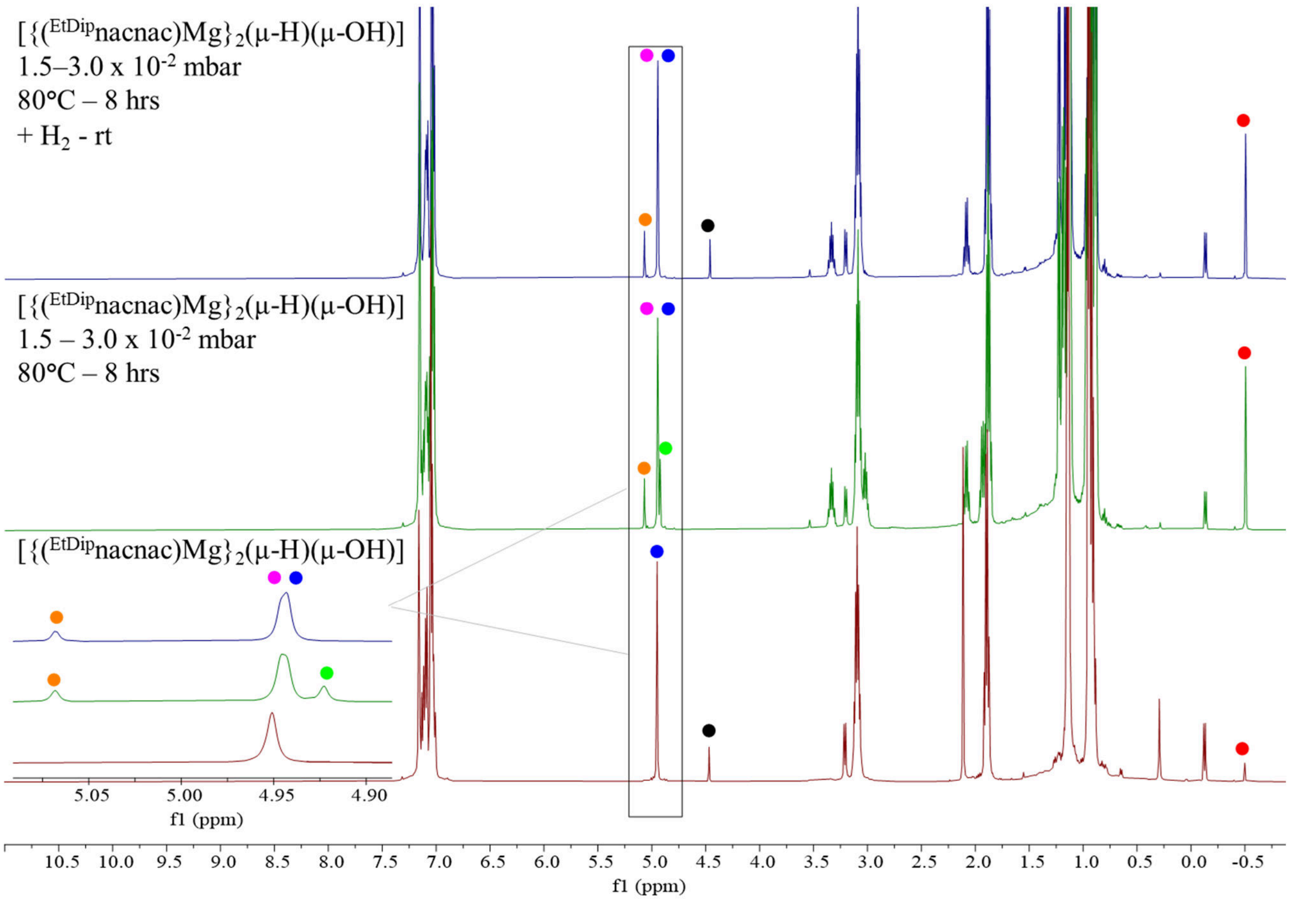

Figure S125. Stacked ^{1}H NMR spectra (499.9 MHz, C_6D_6, 298 K) before and after removing all volatiles from a colourless *in-situ* generated solution of [{(^{EtDip}nacnac)Mg}$_2$(μ-H)(μ-OH)] **4b** (18.0 mg, 18.8 μmol) in C_6D_6 (0.6 mL) in a J Young NMR tube and heating for eight hours at 80°C *in vacuo* (1.5-3.0 × 10^{-2} mbar) followed by the addition of dihydrogen. The black circle denotes the resonance associated with dihydrogen. The green circle denotes the resonance associated with the backbone-C*H* of [{(^{EtDip}nacnac)Mg}$_2$(μ-O)] **1b**. The blue circle denotes the resonance associated with the backbone-C*H* of [{(^{EtDip}nacnac)Mg}$_2$(μ-H)(μ-OH)] **4b**. The orange circle denotes the resonance associated with the backbone-C*H* of ^{EtDip}nacnacH. The pink circle denotes the resonance associated with the backbone-C*H* of [{(^{EtDip}nacnac)Mg(μ-OH)}$_2$] **3b.** The red circle denotes the resonance associated with Mg-(O*H*)$_2$ of [{(^{EtDip}nacnac)Mg(μ-OH)}$_2$] **3b**.

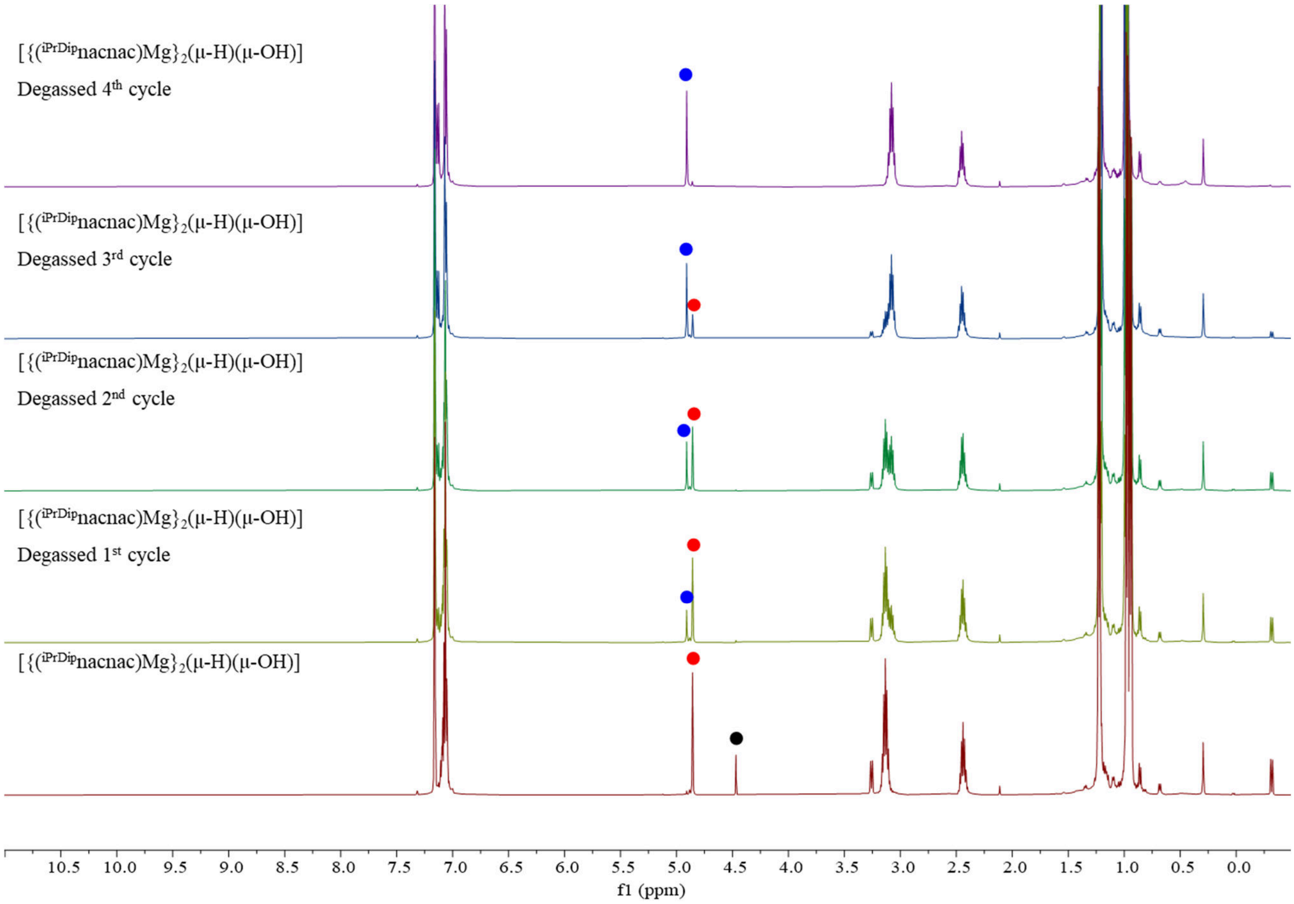

Figure S126. Stacked ^{1}H NMR spectra (500.1 MHz, C_6D_6, 298 K) before and after freeze-pump-thawing a colourless *in-situ* generated solution of [{(^{iPrDip}nacnac)Mg}$_2$(μ-H)(μ-OH)] **4c** (10.0 mg, 9.86 μmol) in C_6D_6 (0.5 mL) in a J Young NMR tube. The black circle denotes the resonance associated with dihydrogen. The red circle denotes the resonance associated with the backbone-C*H* of [{(^{iPrDip}nacnac)Mg}$_2$(μ-H)(μ-OH)] **4c**. The blue circle denotes the resonance associated with the backbone-C*H* of [{(^{iPrDip}nacnac)Mg}$_2$(μ-O)] **1c**.

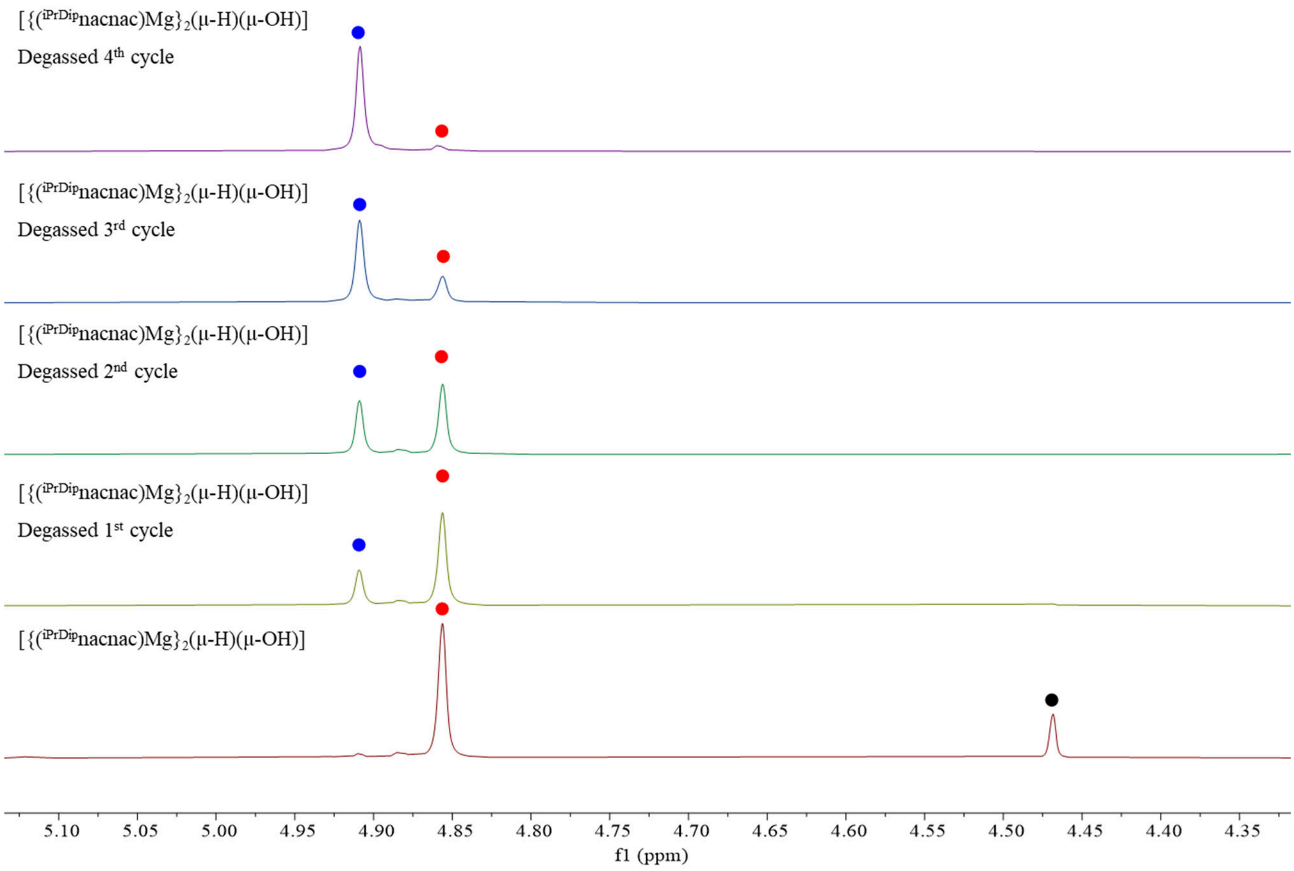

Figure S127. Stacked ^{1}H NMR spectra (500.1 MHz, C_6D_6, 298 K) before and after freeze-pump-thawing a colourless *in-situ* generated solution of [{(^{iPrDip}nacnac)Mg}$_2$(μ-H)(μ-OH)] **4c** (10.0 mg, 9.86 μmol) in C_6D_6 (0.5 mL) in a J Young NMR tube (chemical range: 4.35-5.10 ppm). The black circle denotes the resonance associated with dihydrogen. The red circle denotes the resonance associated with the backbone-C*H* of [{(^{iPrDip}nacnac)Mg}$_2$(μ-H)(μ-OH)] **4c**. The blue circle denotes the resonance associated with the backbone-C*H* of [{(^{iPrDip}nacnac)Mg}$_2$(μ-O)] **1c**.

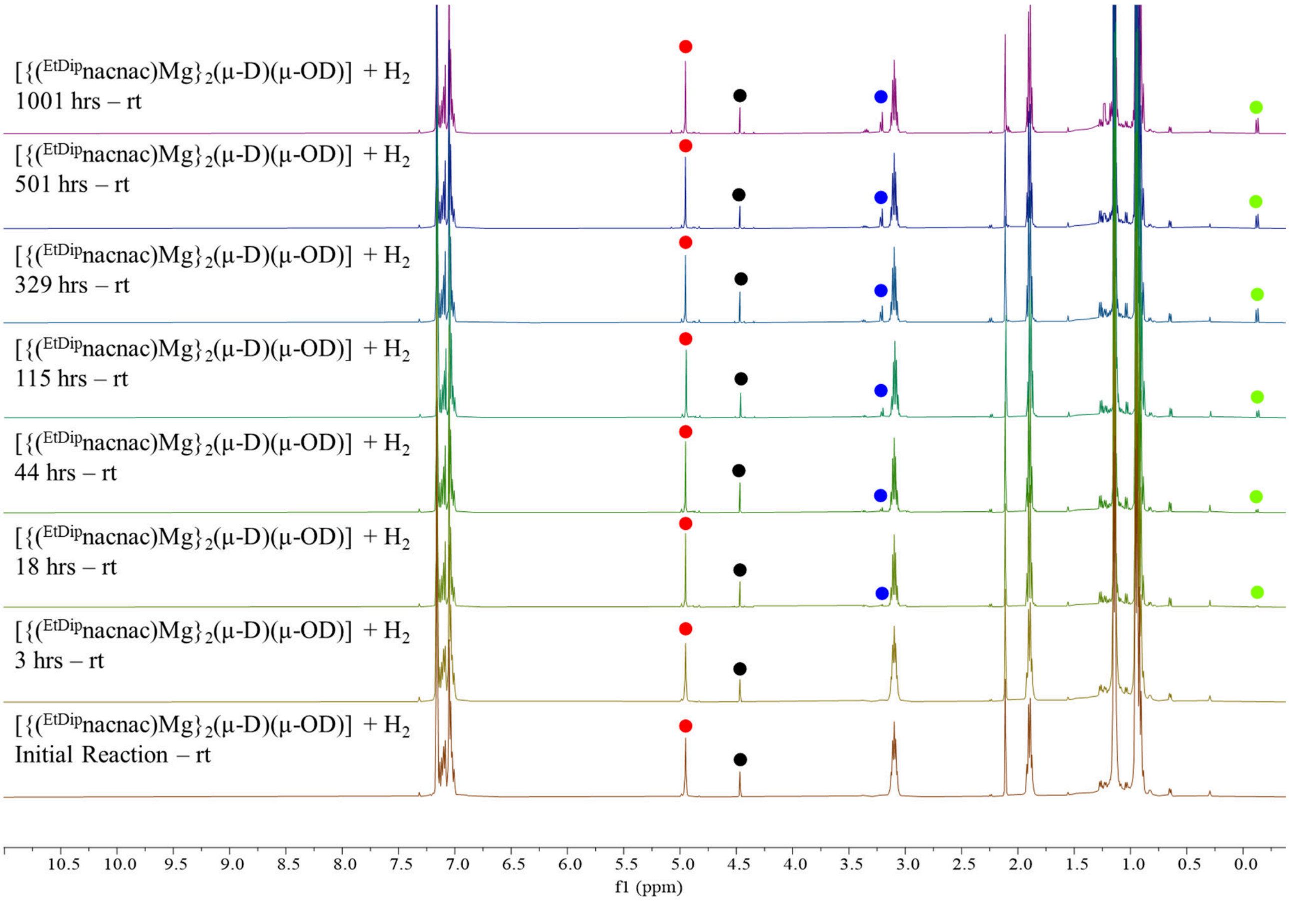

Figure S128. Stacked ^{1}H NMR spectra (500.1 MHz, C_6D_6, 298 K) of the reaction of a colourless *in-situ* generated solution of [{(^{EtDip}nacnac)Mg}$_2$(μ-D)(μ-OD)] **4b-*d*2** and pre-dried dihydrogen (ca. 1 bar) in C_6D_6 (0.6 mL) at room temperature (rt). The black circle denotes the resonance associated with dihydrogen. The red circle denotes the resonance associated with the backbone-C*H* of [{(^{EtDip}nacnac)Mg}$_2$(μ-D)(μ-OD)] **4b-*d*2**. The green circle denotes the resonance associated with Mg-(O*H*) of [{(^{EtDip}nacnac)Mg}$_2$(μ-H)(μ-OH)] **4b**. The blue circle denotes the resonance associated with Mg-*H* of [{(^{EtDip}nacnac)Mg}$_2$(μ-H)(μ-OH)] **4b**.

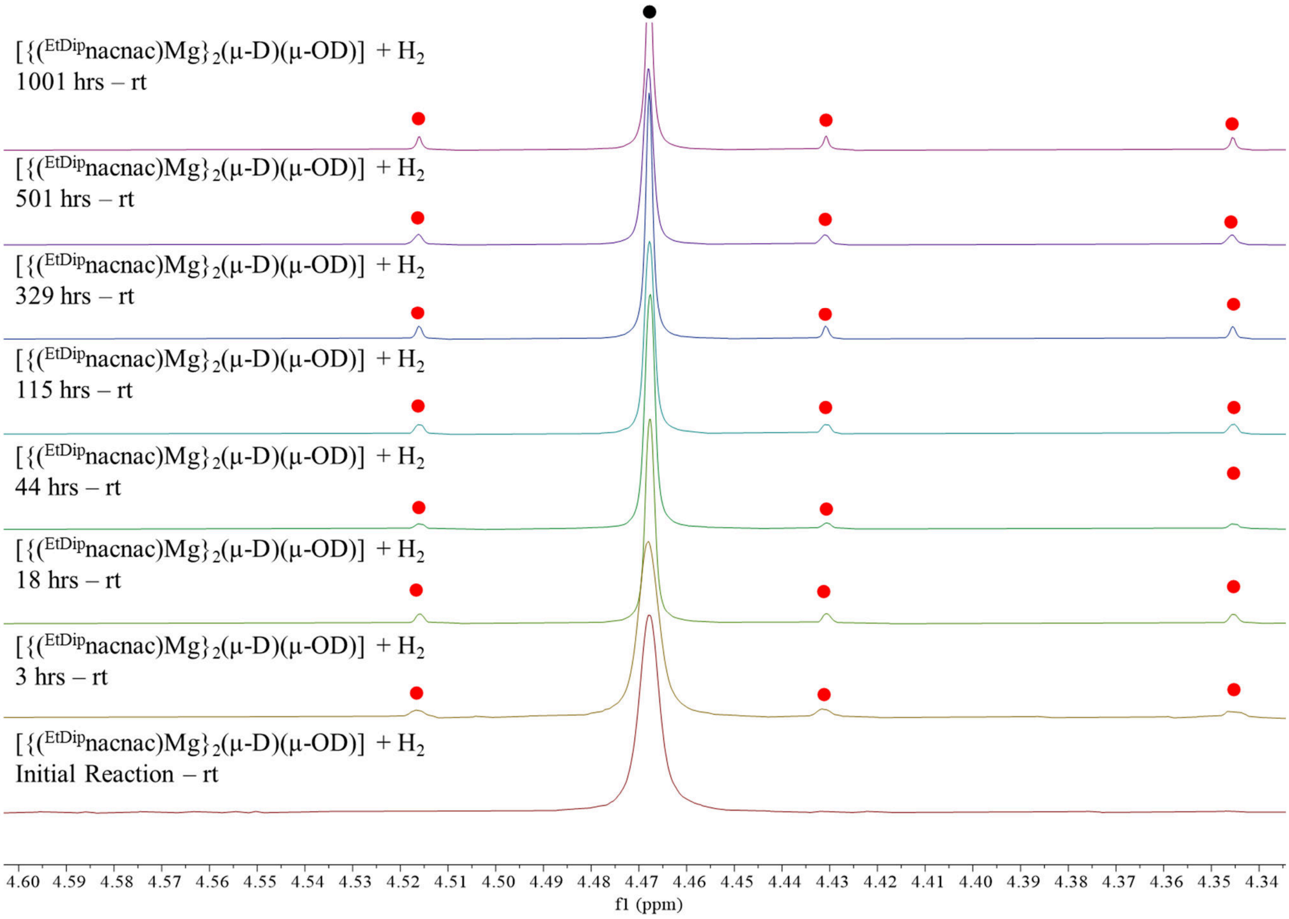

Figure S129. Stacked ^{1}H NMR spectra (500.1 MHz, C_6D_6, 298 K) of the reaction an *in-situ* generated solution of [{(^{EtDip}nacnac)Mg}$_2$(μ-D)(μ-OD)] **4b-*d*$_2$** and pre-dried dihydrogen (ca. 1 bar) in C_6D_6 (0.6 mL) at room temperature (rt) in a J Young NMR tube (chemical range: 4.27-4.65 ppm). The black circle denotes the resonance associated with dihydrogen. The red circles denote the resonance associated with hydrogen deuteride.

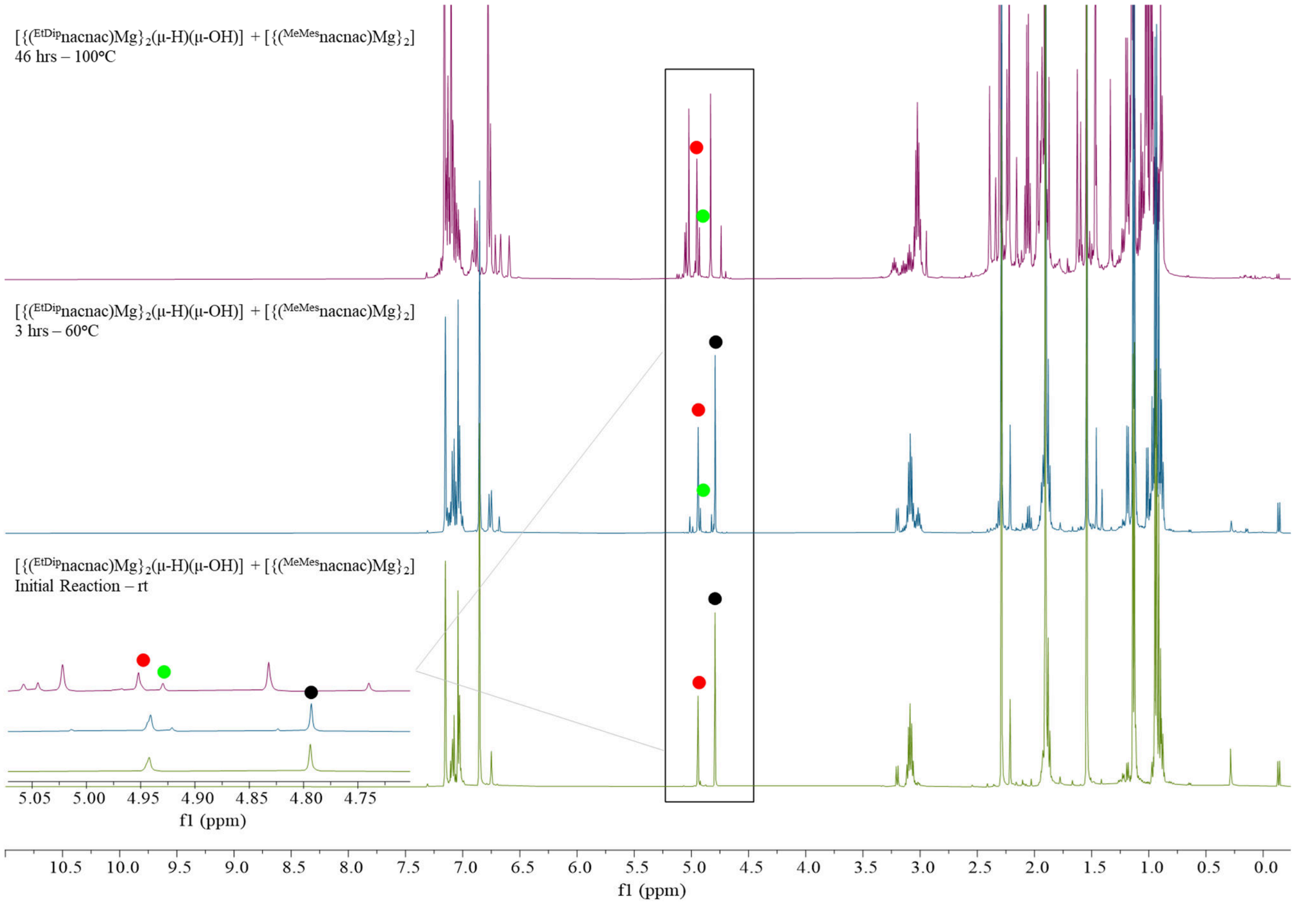

Figure S130. Stacked ^{1}H NMR spectra (499.9 MHz MHz, C_6D_6, 298 K) of the reaction of a colourless solution of [{(^{EtDip}nacnac)Mg}$_2$(μ-H)(μ-OH)] **4b** (9.9 mg, 10.3 μmol, 1.0 equiv.) and [{(^{MeMes}nacnac)Mg}$_2$] (8.9 mg, 12.4 μmol, 1.2 equiv.) in C_6D_6 (0.6 mL). The black circle denotes the resonance associated with the backbone-C*H* of [{(^{MeMes}nacnac)Mg}$_2$]. The green circle denotes the resonance associated with the backbone-C*H* [{(^{EtDip}nacnac)Mg}$_2$(μ-O)] **1b**. The red circle denotes the resonance associated with the backbone-C*H* of [{(^{EtDip}nacnac)Mg}$_2$(μ-H)(μ-OH)] **4b**.

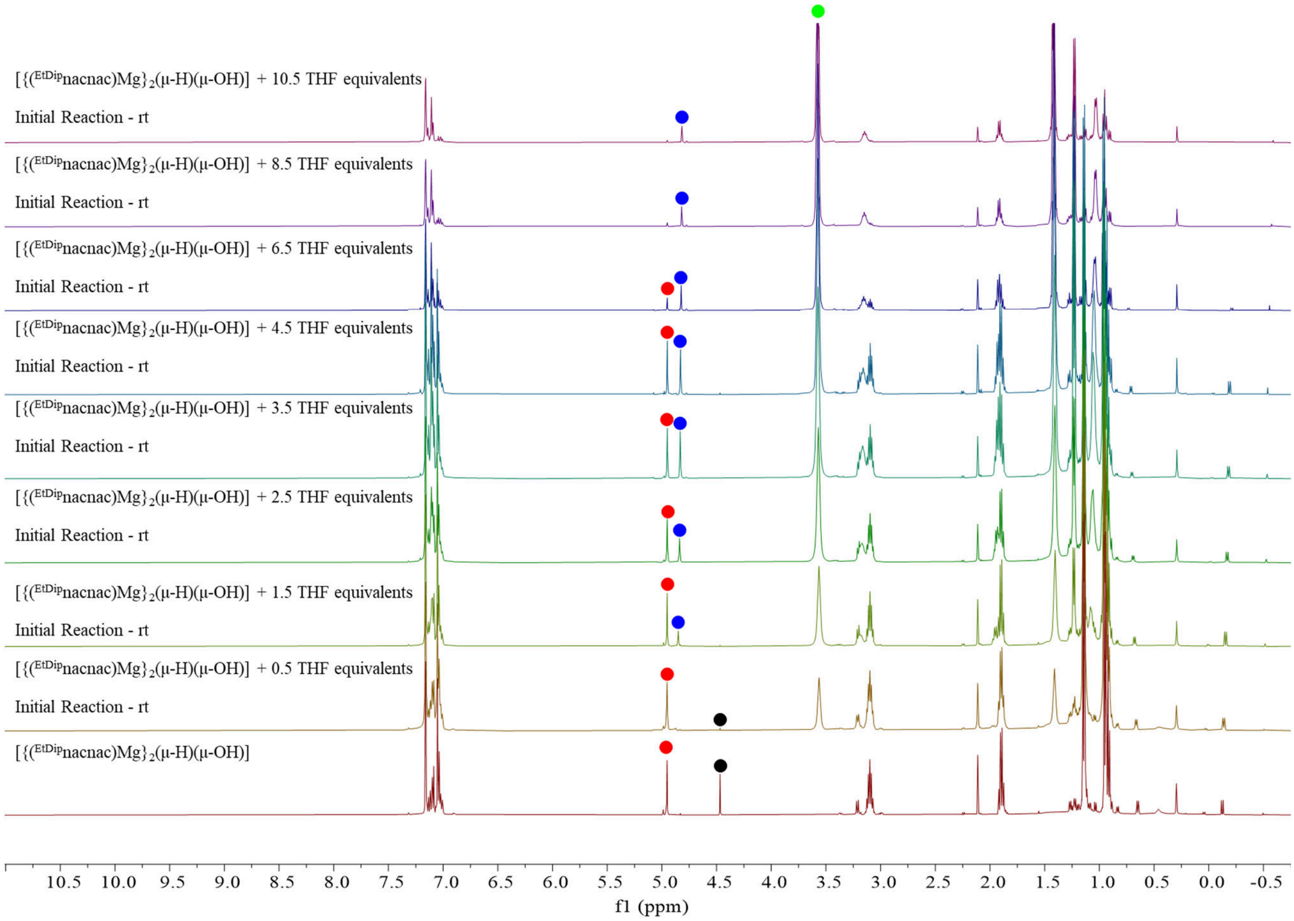

Figure S131. Stacked ^{1}H NMR spectra (500.1 MHz, C_6D_6, 298 K) from the titration of a colourless *in-situ* generated solution of [{(^{EtDip}nacnac)Mg}$_2$(μ-H)(μ-OH)] **4b** (20.6 mg, 21.5 μmol, 1.0 equiv.) in C_6D_6 (0.6 mL) and THF (8.8 μL, 10.8 μmol, 0.5 equiv., from a 1.22 M THF stock solution in C_6D_6) at room temperature (rt) in a J Young NMR tube. The black circle denotes the resonance associated with dihydrogen. The red circle denotes the resonance associated with the backbone-C*H* of [{(^{EtDipn}acnac)Mg}$_2$(μ-H)(μ-OH)] **4b**. The blue circle denotes the resonance associated with the backbone-C*H* of [{(^{EtDip}nacnac)Mg(THF)}$_2$(μ-O)] **5b**. The green circle denotes the resonance associated with O(C*H*$_2$) of THF.

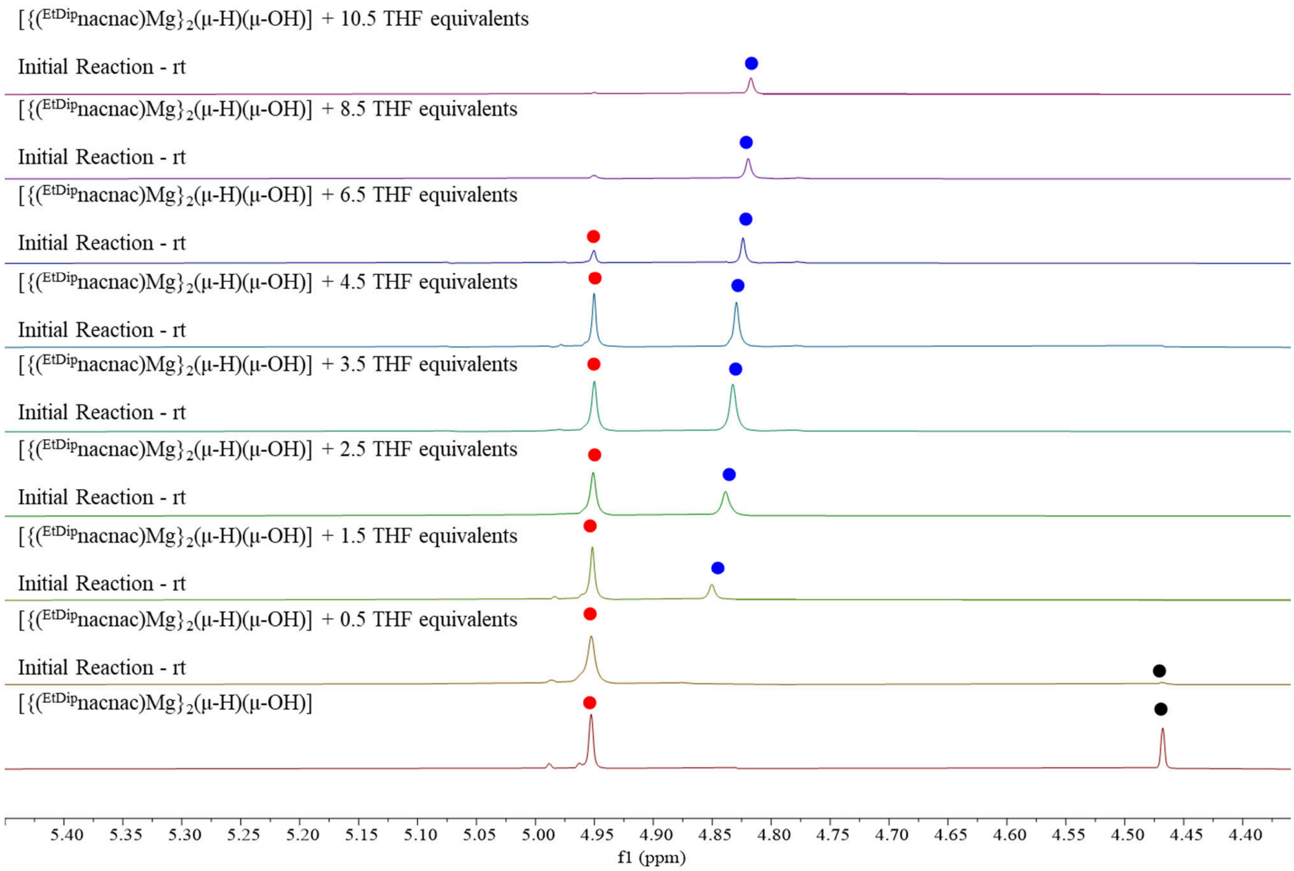

Figure S132. Stacked ^{1}H NMR spectra (500.1 MHz, C_6D_6, 298 K) from the titration of an *in-situ* generated solution of [{(^{EtDip}nacnac)Mg}$_2$(μ-H)(μ-OH)] **4b** (20.6 mg, 21.5 μmol, 1.0 equiv.) in C_6D_6 (0.6 mL) and THF (8.8 μL, 10.8 μmol, 0.5 equiv., from a 1.22 M THF stock solution in C_6D_6) at room temperature (rt) in a J Young NMR tube (chemical range: 4.35-5.40 ppm). The black circle denotes the resonance associated with dihydrogen. The red circle denotes the resonance associated with the backbone-C*H* of [{(^{EtDip}nacnac)Mg}$_2$(μ-H)(μ-OH)] **4b**. The blue circle denotes the resonance associated with the backbone-C*H* of [{(^{EtDip}nacnac)Mg(THF)}$_2$(μ-O)] **5b**.

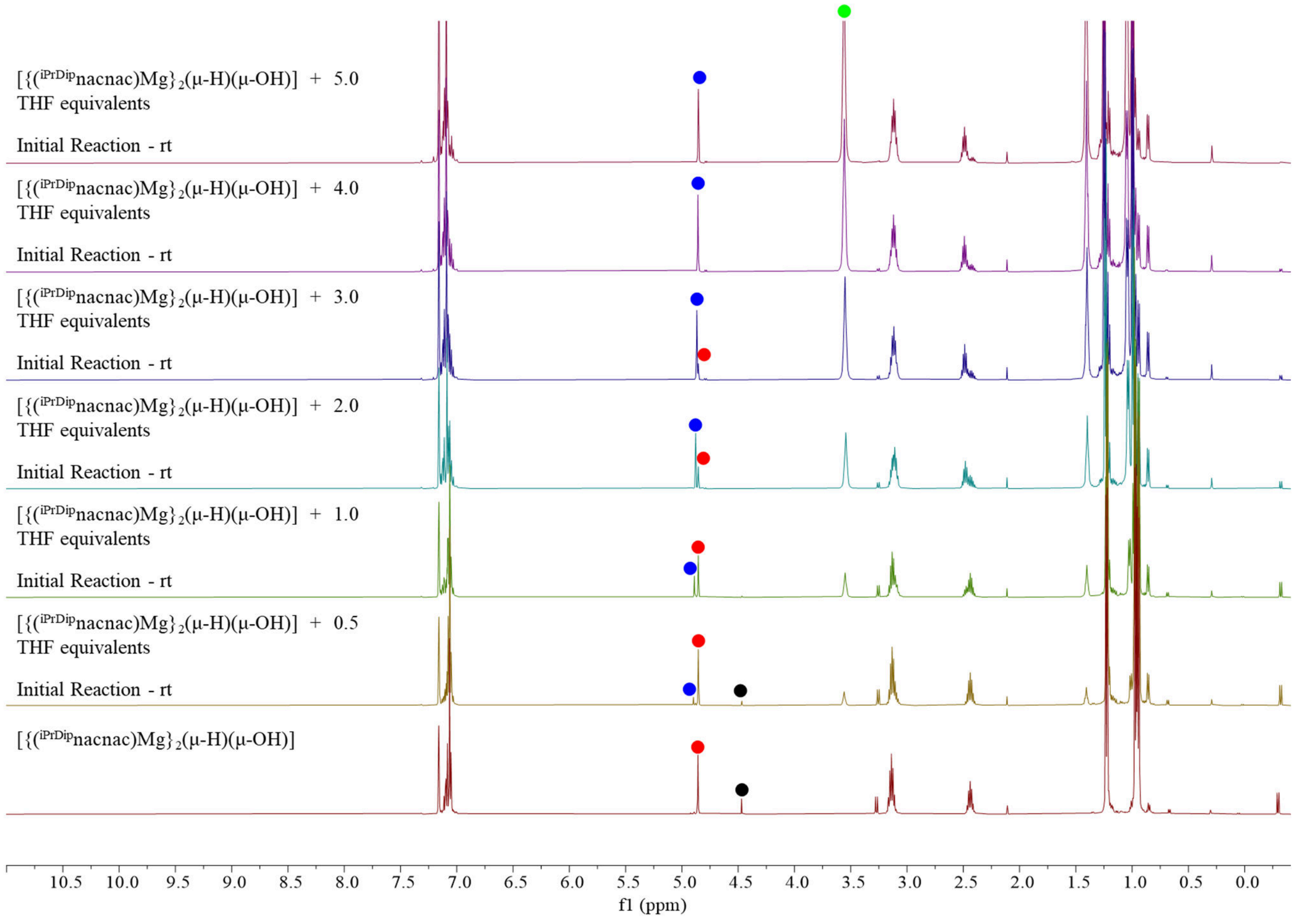

Figure S133. Stacked ^{1}H NMR spectra (499.9 MHz, C_6D_6, 298 K) from the titration of a colourless *in-situ* generated solution of [{(^{iPrDip}nacnac)Mg}$_2$(μ-H)(μ-OH)] **4c** (9.5 mg, 9.4 μmol, 1.0 equiv.) in C_6D_6 (0.6 mL) and THF (16.0 μL, 2.4 μmol, 0.25 equiv., from a 0.15 M THF stock solution in C_6D_6) at room temperature (rt) in a J Young NMR tube. The black circle denotes the resonance associated with dihydrogen. The red circle denotes the resonance associated with the backbone-C*H* of [{(^{iPrDip}nacnac)Mg}$_2$(μ-H)(μ-OH)] **4c**. The blue circle denotes the resonance associated with the backbone-C*H* of [{(^{iPrDip}nacnac)Mg(THF)}$_2$(μ-O)] **5c**. The green circle denotes the resonance associated with O(C*H*$_2$) of THF.

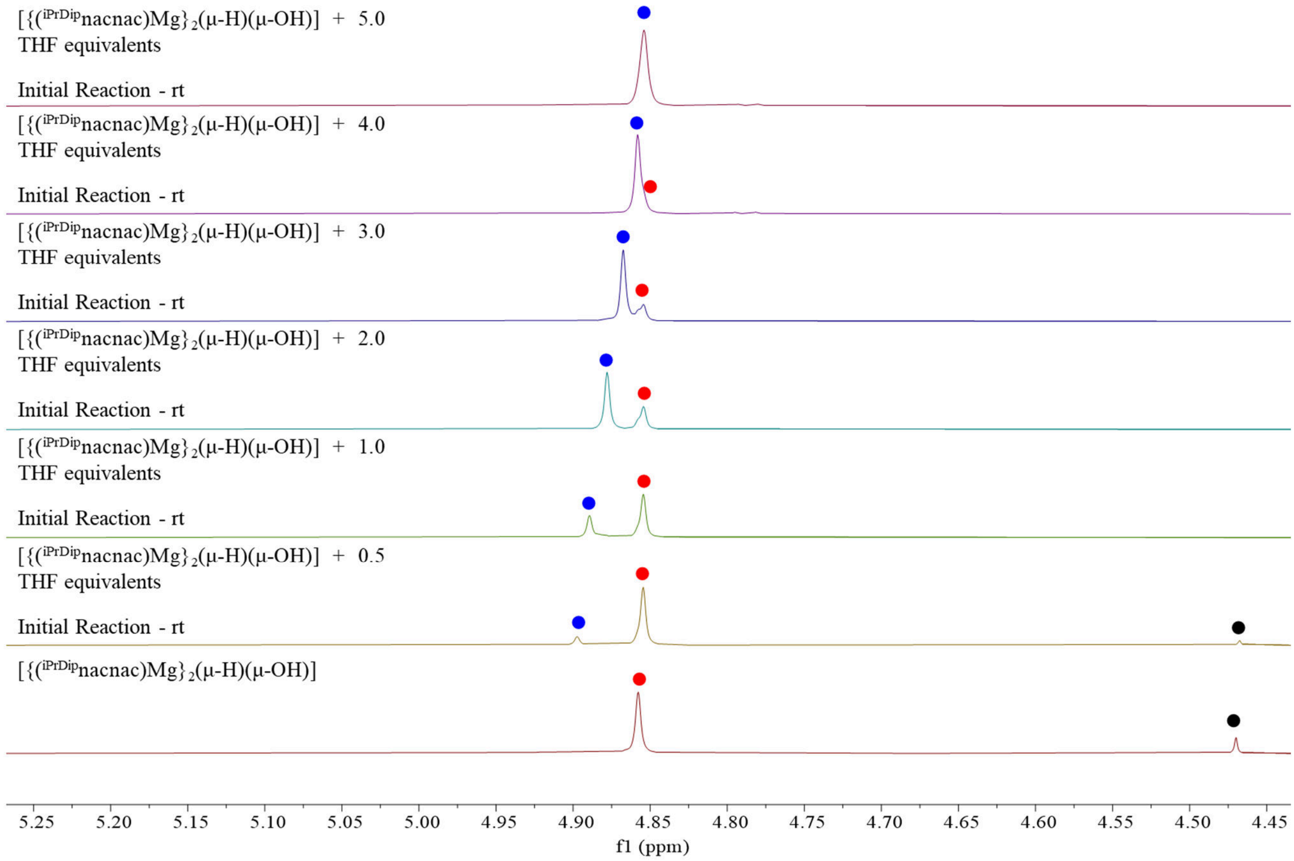

Figure S134. Stacked ^{1}H NMR spectra (499.9 MHz, C_6D_6, 298 K) from the titration reaction of a colourless *in-situ* generated solution of [{(^{iPrDip}nacnac)Mg}$_2$(μ-H)(μ-OH)] **4c** (9.5 mg, 9.4 μmol, 1.0 equiv.) in C_6D_6 (0.6 mL) and THF (16.0 μL, 2.4 μmol, 0.25 equiv., from a 0.15 M THF stock solution in C_6D_6) at room temperature (rt) in a J Young NMR tube (chemical range: 4.45-5.25 ppm). The black circle denotes the resonance associated with dihydrogen. The red circle denotes the resonance associated with the backbone-C*H* of [{(^{iPrDip}nacnac)Mg}$_2$(μ-H)(μ-OH)] **4c**. The blue circle denotes the resonance associated with the backbone-C*H* of [{(^{iPrDip}nacnac)Mg(THF)}$_2$(μ-O)] **5c**.

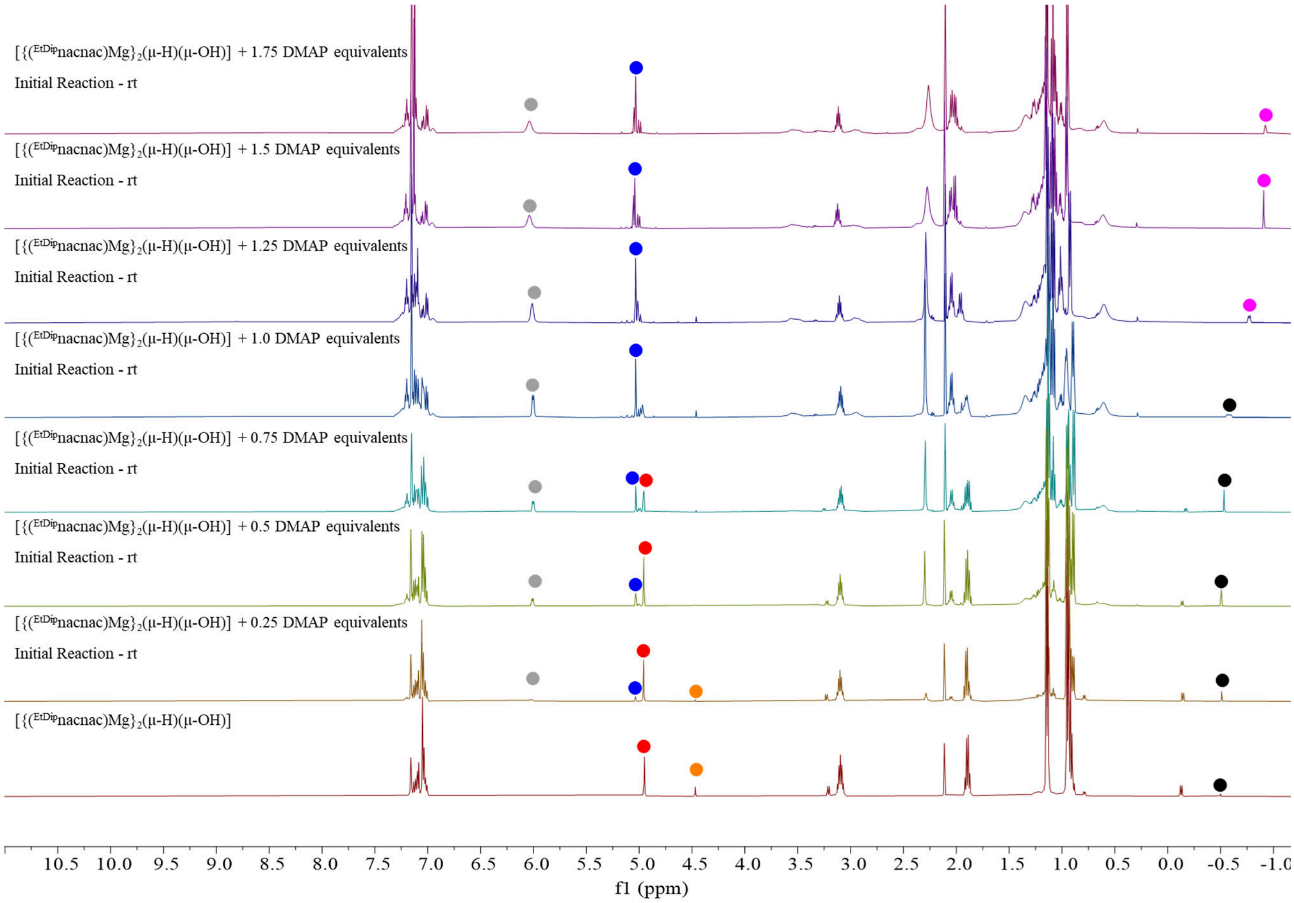

Figure S135. Stacked ^{1}H NMR spectra (500.1 MHz, C_6D_6, 298 K) from the titration of an *in-situ* generated solution of [{(^{EtDip}nacnac)Mg}$_2$(μ-H)(μ-OH)] **4b** (17.7 mg, 18.5 μmol, 1.0 equiv.) in C_6D_6 (0.6 mL) and DMAP (28 μL, 4.63 μmol, 0.25 equiv., from a 0.165 M DMAP stock solution in C_6D_6) at room temperature (rt) in a J Young NMR tube. The orange circle denotes the resonance associated with dihydrogen. The red circle denotes the resonance associated with the backbone-C*H* of [{(^{EtDip}nacnac)Mg}$_2$(μ-H)(μ-OH)] **4b**. The blue circle denotes the resonance associated with the backbone-C*H* of [{(^{EtDip}nacnac)Mg(DMAP)}$_2$(μ-O)] **6b**. The grey circle denotes the resonance associated with Ar_{DMAP}-*H* of [{(^{EtDip}nacnac)Mg(DMAP)}$_2$(μ-O)] **6b**. The black circle denotes the resonance associated with Mg-(O*H*)$_2$ of [{(^{EtDip}nacnac)Mg(μ-OH)}$_2$] **3b**. The pink circle denotes the resonance associated with Mg-(O*H*)$_2$ of [{(^{EtDip}nacnac)Mg(DMAP)(μ-OH)}$_2$] **8b**.

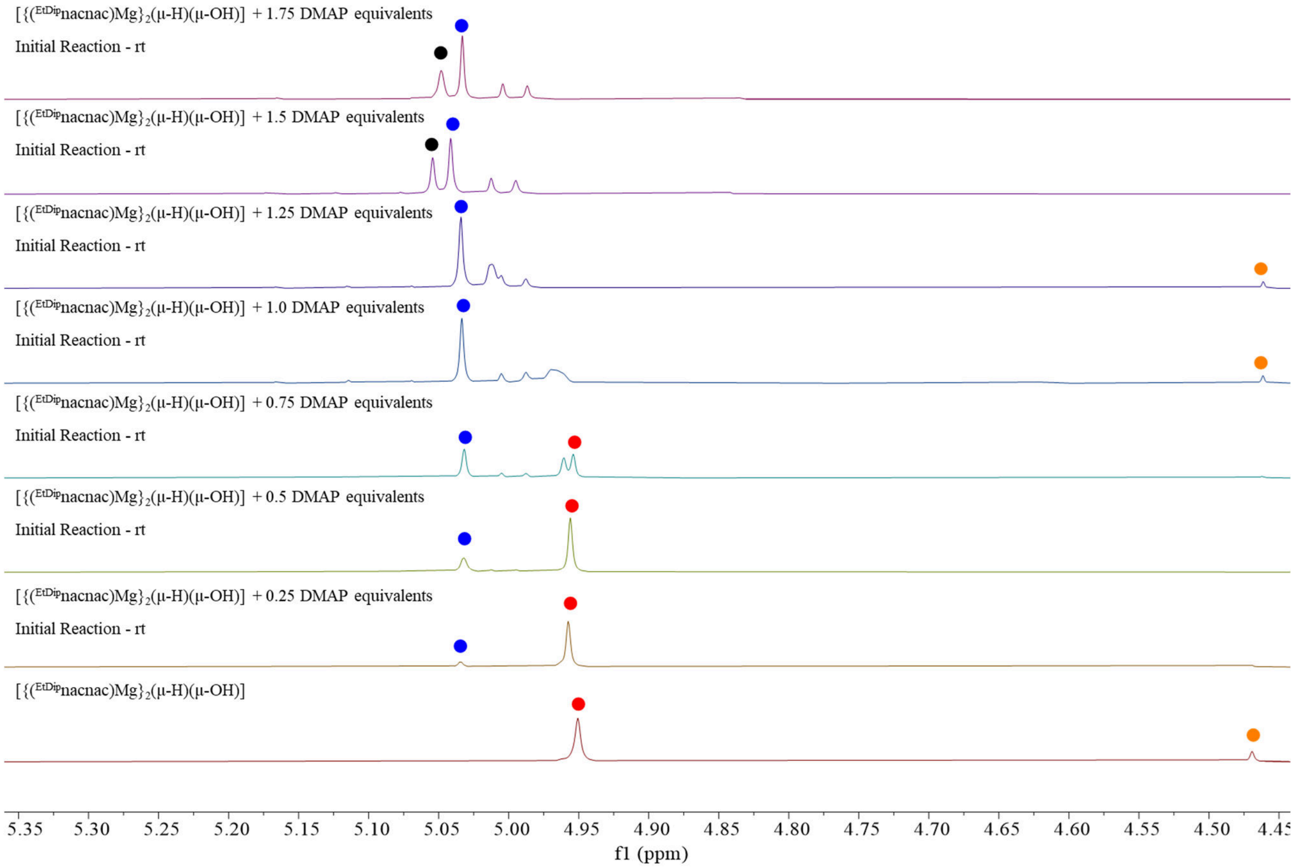

Figure S136. Stacked ^{1}H NMR spectra (500.1 MHz, C_6D_6, 298 K) from the titration reaction of a colourless *in-situ* generated solution of [{(^{EtDip}nacnac)Mg}$_2$(μ-H)(μ-OH)] **4b** (17.7 mg, 18.5 μmol, 1.0 equiv.) in C_6D_6 (0.6 mL) and DMAP (28 μL, 4.63 μmol, 0.25 equiv., from a 0.165 M DMAP stock solution in C_6D_6) at room temperature (rt) in a J Young NMR tube (chemical range: 4.45-5.35 ppm). The orange circle denotes the resonance associated with dihydrogen. The red circle denotes the resonance associated with the backbone-C*H* of [{(^{EtDip}nacnac)Mg}$_2$(μ-H)(μ-OH)] **4b**. The blue circle denotes the resonance associated with the backbone-C*H* of [{(^{EtDip}nacnac)Mg(DMAP)}$_2$(μ-O)] **6b**. The black circle denotes the resonance associated with the backbone-C*H* of [{(^{EtDip}nacnac)Mg(DMAP)(μ-OH)}$_2$] **8b**.

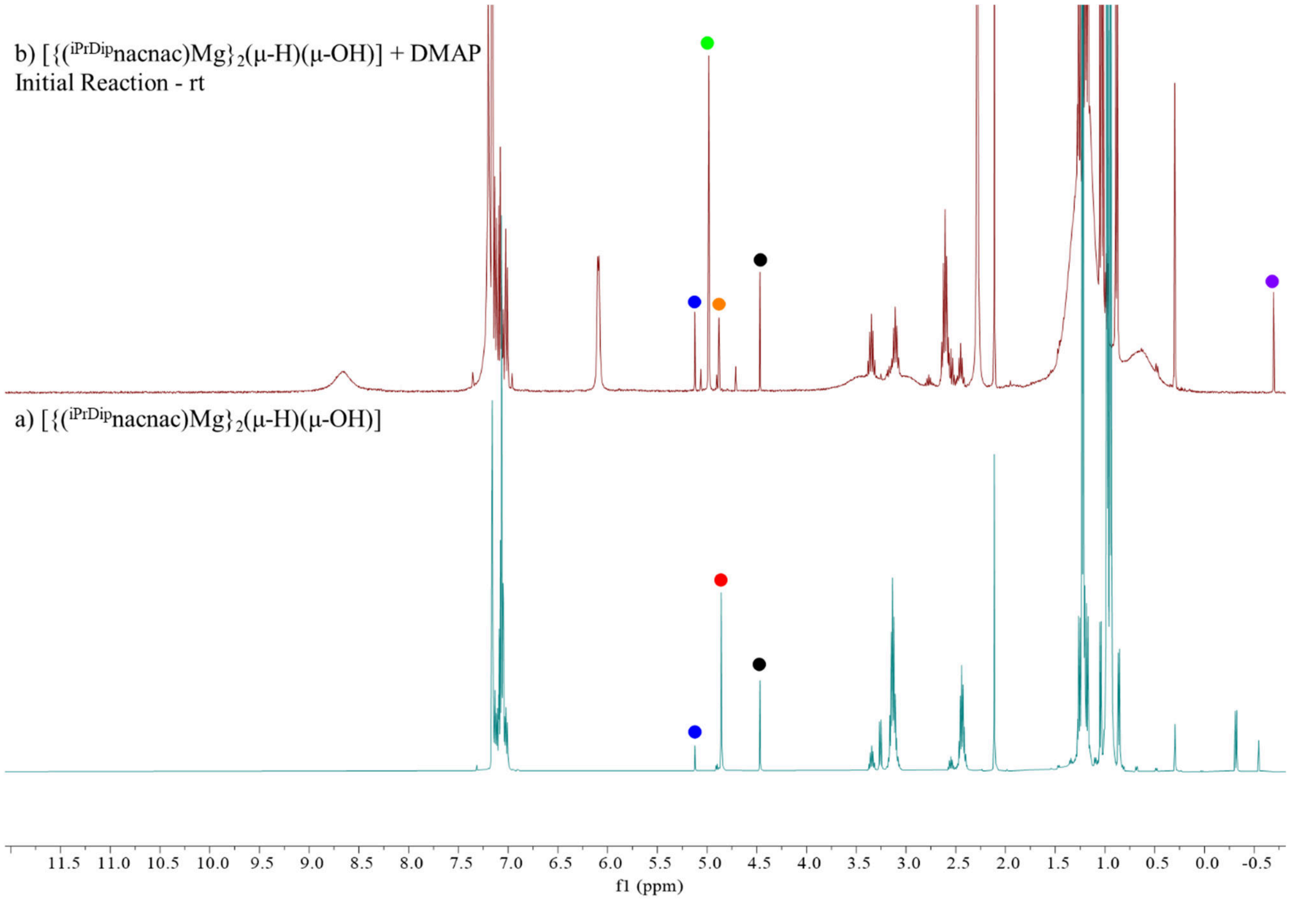

Figure S137. Stacked ^{1}H NMR spectra of the reaction of a colourless *in-situ* generated solution of [{(^{iPrDip}nacnac)Mg}$_2$(μ-H)(μ-OH)] **4c** (10.0 mg, 9.9 μmol, 1.0 equiv.) and DMAP (2.5 mg, 20 μmol, 2.1 equiv.) in C_6D_6 (0.6 mL) in a J Young NMR tube at room temperature (rt). The black circle denotes the resonance associated with dihydrogen. The red circle denotes the resonance associated with the backbone-C*H* of [{(^{iPrDip}nacnac)Mg}$_2$(μ-H)(μ-OH)] **4c**. The blue circle denotes the resonance associated with the backbone-C*H* of ^{iPrDip}nacnacH. The green circle denotes the resonance associated with the backbone-C*H* of [{(^{iPrDip}nacnac)Mg(DMAP)}$_2$(μ-O)] **6c**. The orange circle denotes the resonance associated with the backbone-C*H* of [{(^{iPrDip}nacnac)Mg(DMAP)(μ-OH)}$_2$] **8c**. The purple circle denotes the resonance associated with Mg-(O*H*)$_2$ of [{(^{iPrDip}nacnac)Mg(DMAP)(μ-OH)}$_2$] **8c**. Spectrum a) 499.9 MHz, C_6D6, 298 K. Spectrum b) 400.1 MHz, C_6D_6, 295 K.

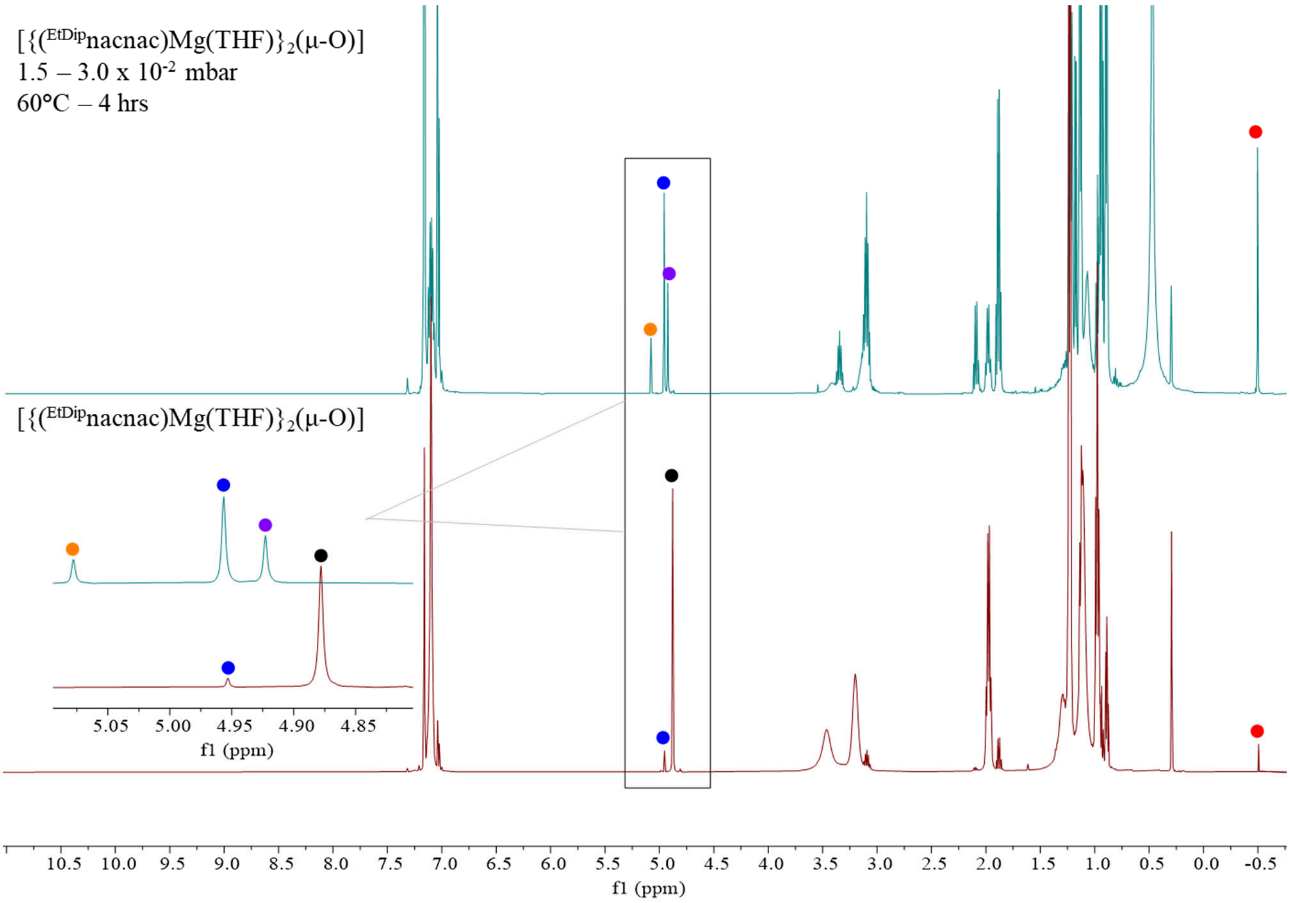

Figure S138. Stacked ^{1}H NMR spectra (499.9 MHz, C_6D_6, 298 K) after removing all volatiles from a colourless solution of [{(^{EtDip}nacnac)Mg(THF)}$_2$(μ-O)] **5b** (15.0 mg, 13.6 μmol) in C_6D_6 (0.5 mL) in a J Young NMR tube and heating for four hours at 60°C *in vacuo* (1.5-3.0 × 10^{-2} mbar). The black circle denotes the resonance associated with the backbone-C*H* of [{(^{EtDip}nacnac)Mg(THF)}$_2$(μ-O)] **5b**. The blue circle denotes the resonance associated with the backbone-C*H* of [{(^{EtDip}nacnac)Mg(μ-OH)}$_2$] **3b**. The red the backbone denotes the resonance associated with Mg-(O*H*)$_2$ of [{(^{EtDip}nacnac)Mg(μ-OH)}$_2$] **3b**. The purple circle denotes the resonance associated with the backbone-C*H* of [{(^{EtDip}nacnac)Mg}$_2$(μ-O)] **1b**. The orange circle denotes the backbone-C*H* of ^{EtDip}nacnacH.

2.6 Synthesis and reactions of magnesium hydroxide complexes

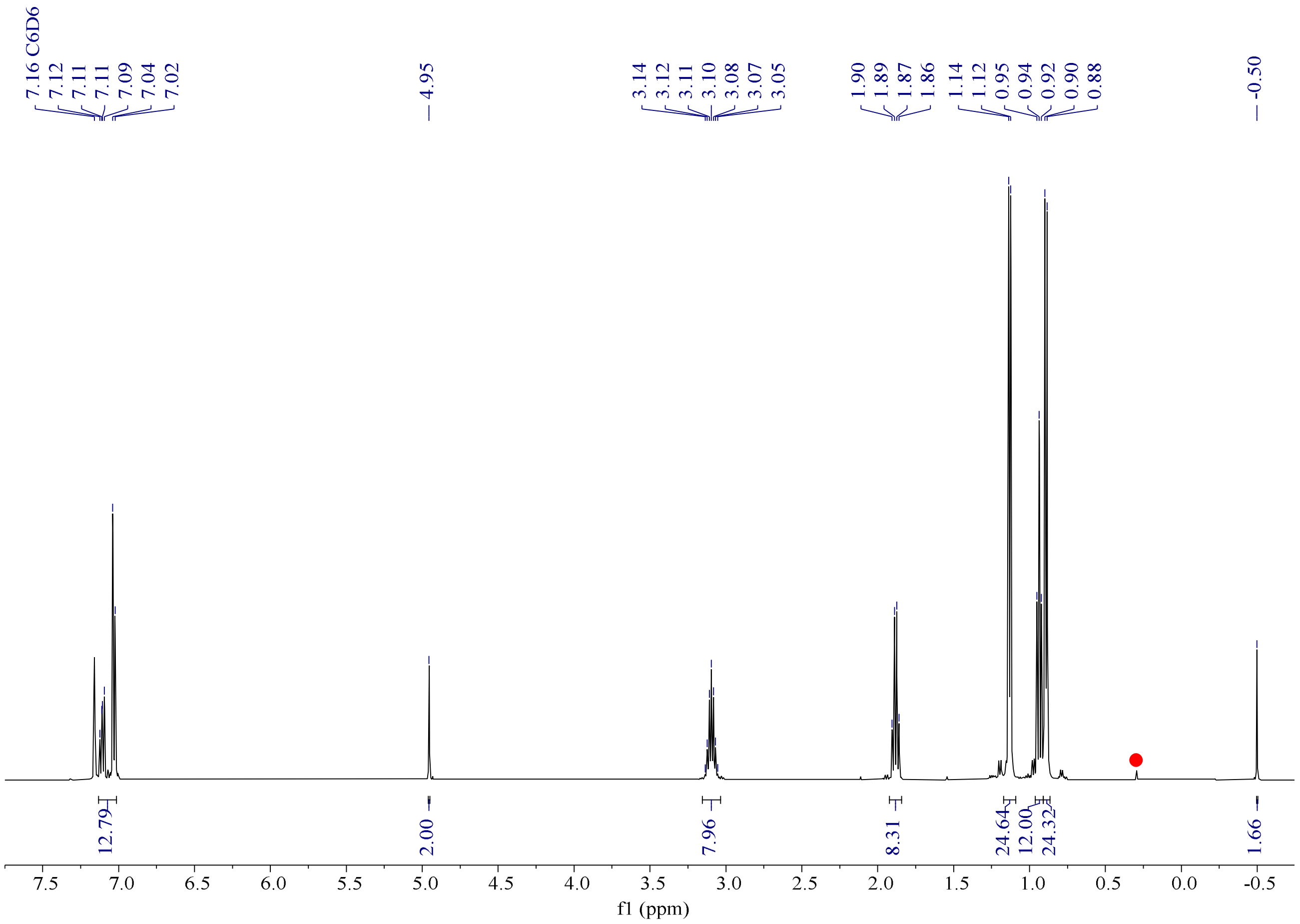

Figure S139. ^{1}H NMR spectrum (500.1 MHz, C_6D_6, 298 K) of isolated [{(^{EtDip}nacnac)Mg(μ-OH)}$_2$] **3b**. The red circle denotes the resonance associated with silicone grease.

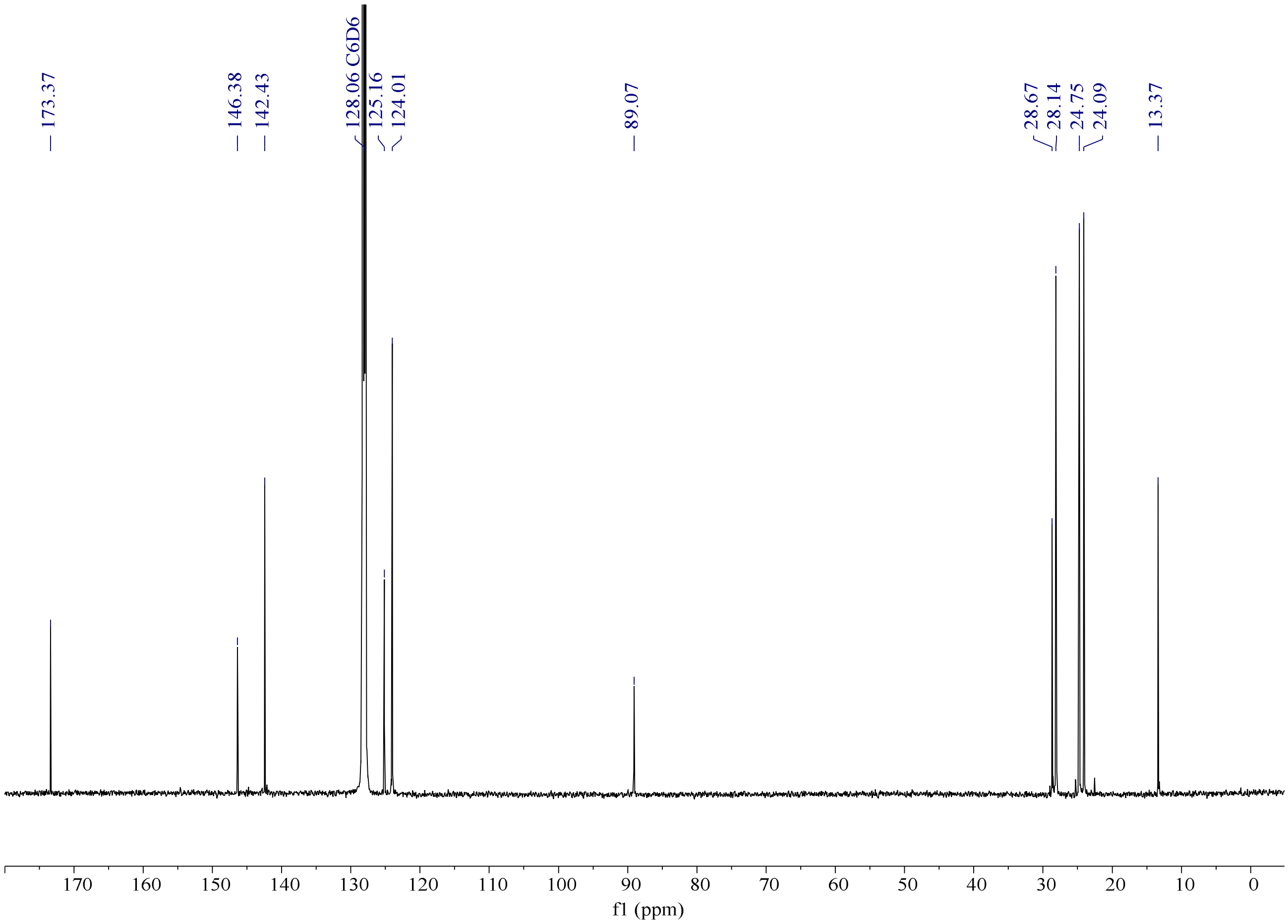

Figure S140. $^{13}C\{^1H\}$ NMR spectrum (125.8 MHz, C_6D_6, 298 K) of isolated $[\{(^{EtDip}nacnac)Mg(\mu\text{-}OH)\}_2]$ **3b**.

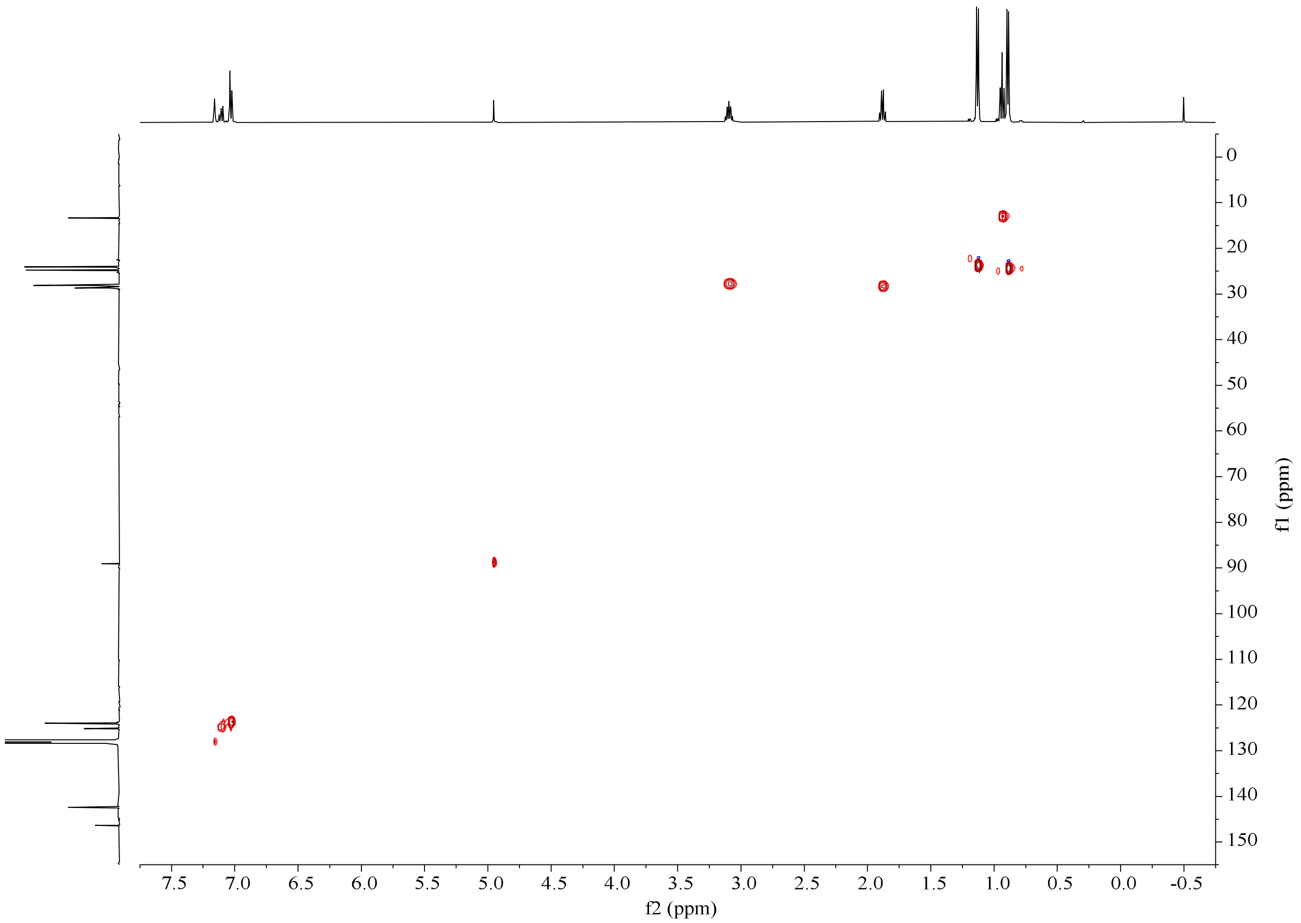

Figure S141. ^{1}H-^{13}C HSQC NMR spectrum of isolated [{(^{EtDip}nacnac)Mg(μ-OH)}$_2$] **3b**.

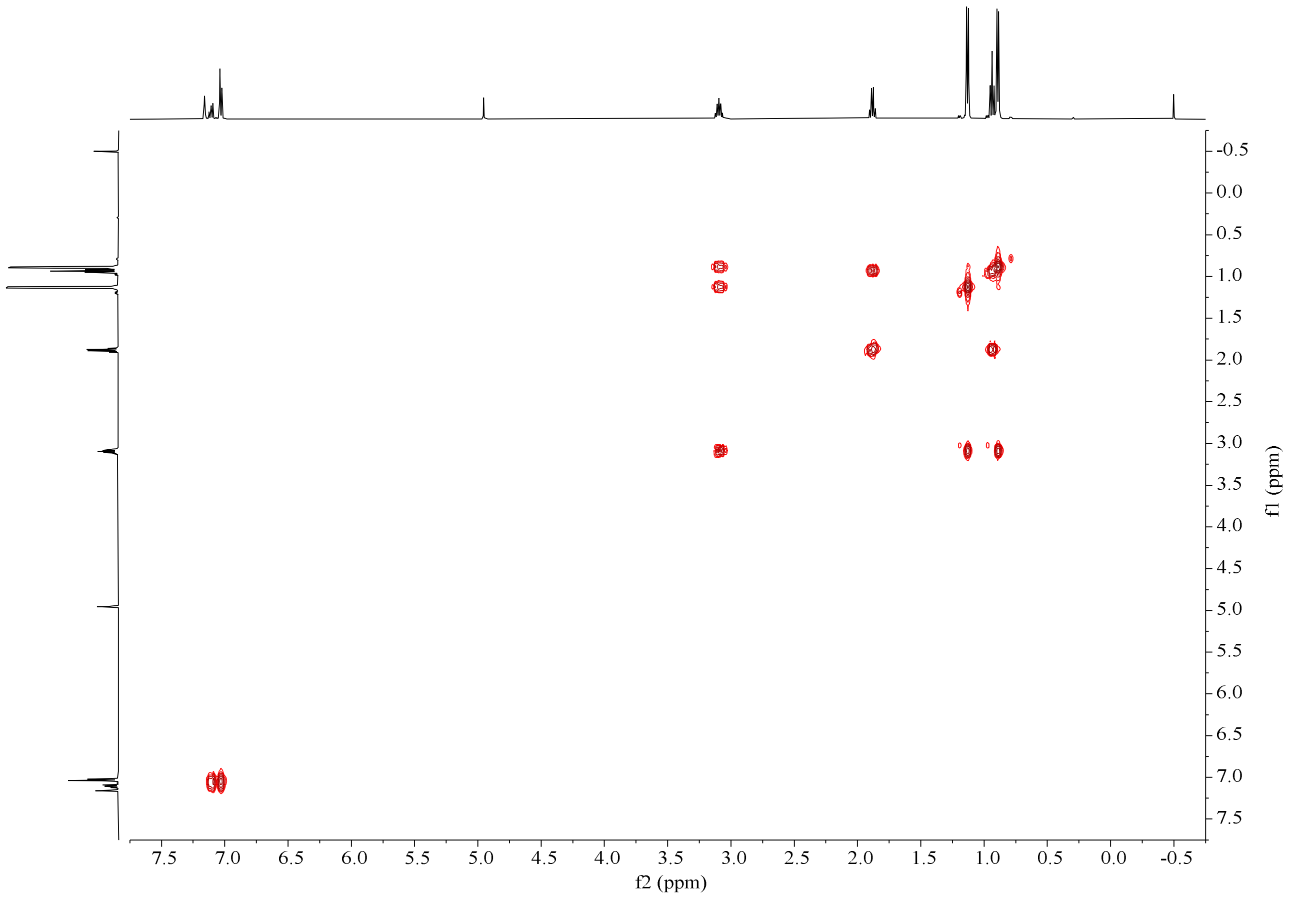

Figure S142. ^{1}H-^{1}H COSY NMR spectrum of isolated [{(^{EtDip}nacnac)Mg(μ-OH)}$_2$] **3b**.

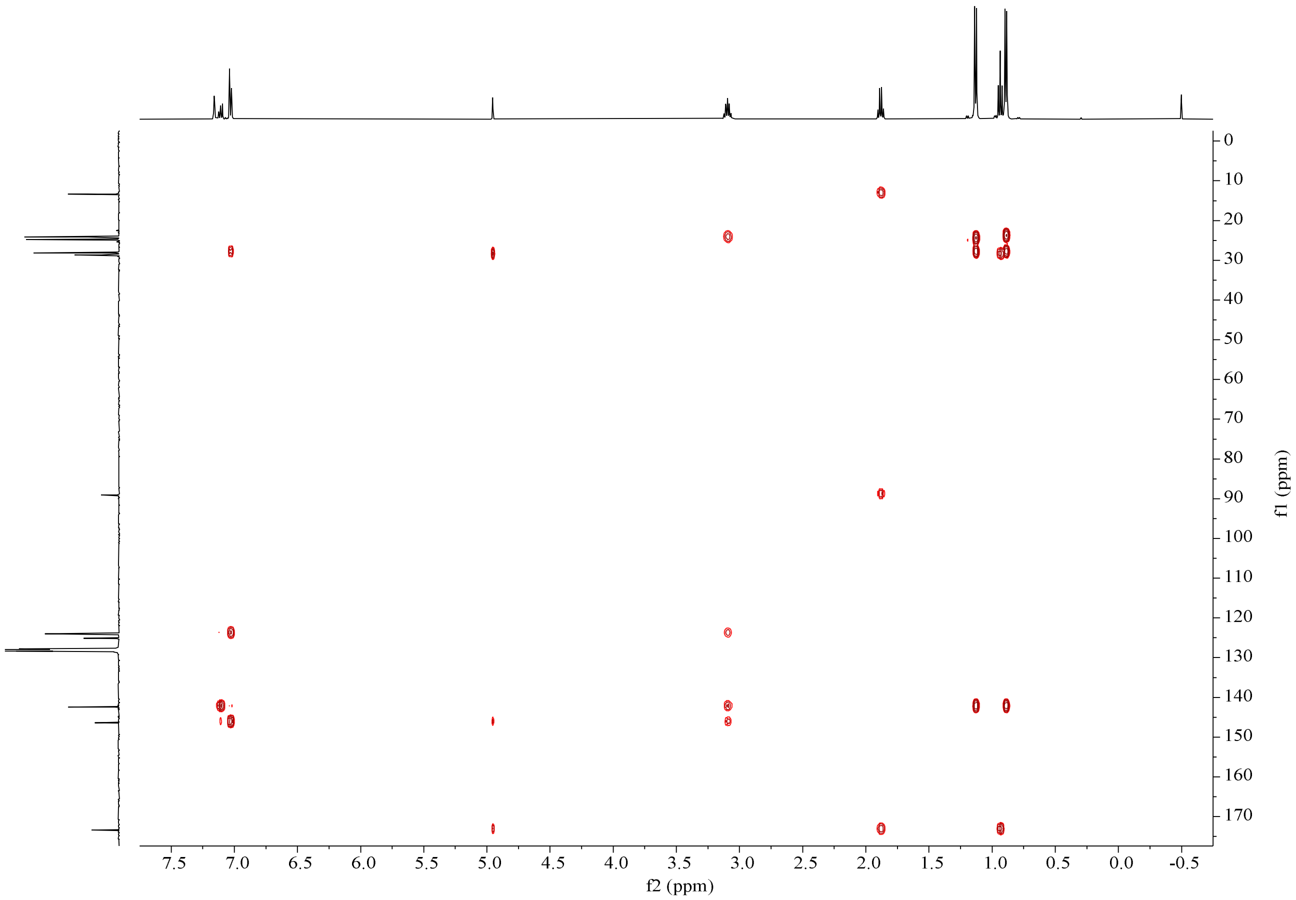

Figure S143. ^{1}H-^{13}C HMBC NMR spectrum of isolated [{(^{EtDip}nacnac)Mg(μ-OH)}$_2$] **3b**.

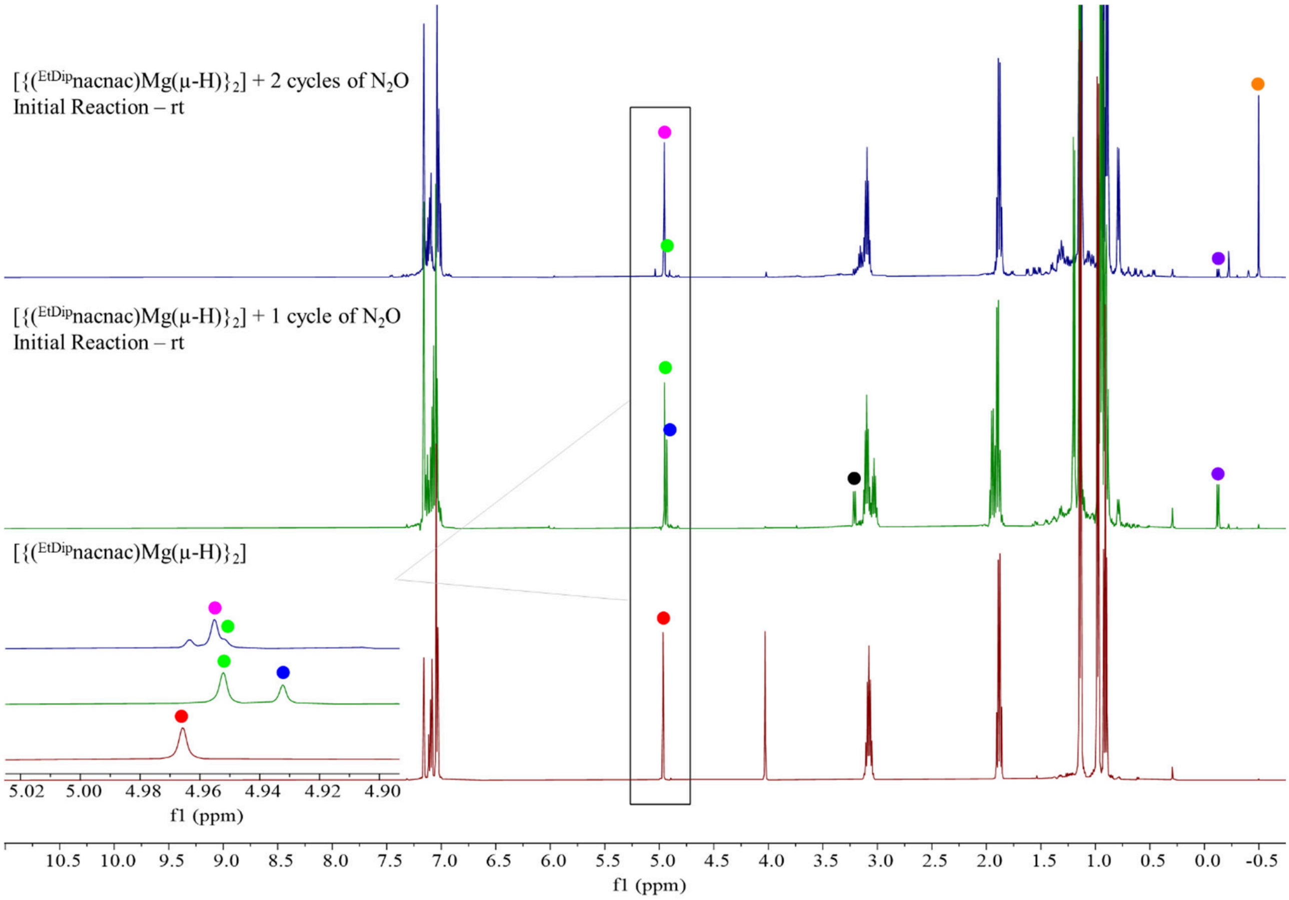

Figure S144. Stacked ^{1}H NMR spectra (500.1 MHz, C_6D_6, 298 K) of the reaction of a colourless solution of [{(^{EtDip}nacnac)Mg(μ-H)}$_2$] **7b** (10.0 mg, 10.6 μmol) in C_6D_6 (0.5 mL) and nitrous oxide (ca. 1 bar) at room temperature (rt) in a J Young NMR tube. The red circle denotes the resonance associated with the backbone-C*H* of [{(^{EtDip}nacnac)Mg(μ-H)}$_2$] **7b**. The blue circle denotes the resonance associated with the backbone-C*H* of [{(^{EtDip}nacnac)Mg}$_2$(μ-O)] **1b**. The green circle denotes the resonance associated with the backbone-C*H* of [{(^{EtDip}nacnac)Mg}$_2$(μ-H)(μ-OH)] **4b**. The black circle denotes the resonance associated with Mg-*H* of [{(^{EtDip}nacnac)Mg}$_2$(μ-H)(μ-OH)] **4b**. The purple circle denotes the resonance associated with Mg-O*H* of [{(^{EtDip}nacnac)Mg}$_2$(μ-H)(μ-OH)] **4b**. The pink circle denotes the resonance associated with the backbone-C*H* of [{(^{EtDip}nacnac)Mg(μ-OH)}$_2$] **3b**. The orange circle denotes the resonance associated with Mg-(O*H*)$_2$ of [{(^{EtDip}nacnac)Mg(μ-OH)}$_2$] **3b**.

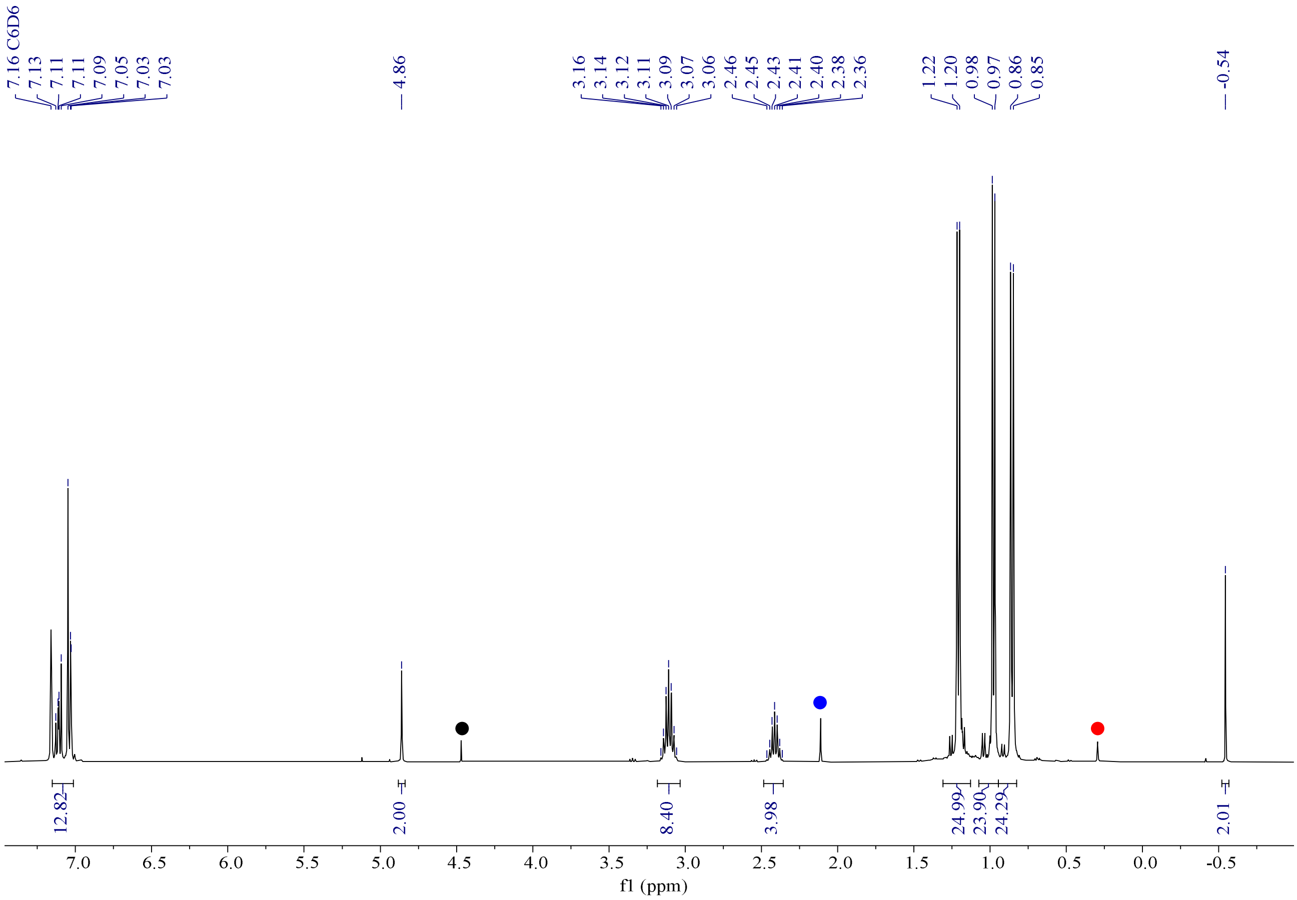

Figure S145. ^{1}H NMR spectrum of (400.1 MHz, C_6D_6, 295 K) of *in-situ* generated [{(^{iPrDip}nacnac)Mg(μ-OH)}$_2$] **3c**. The red circle denotes the resonance associated with silicone grease. The blue circle denotes the resonance associated with toluene (Ph-CH_3). The black circle denotes the resonance associated with dihydrogen.

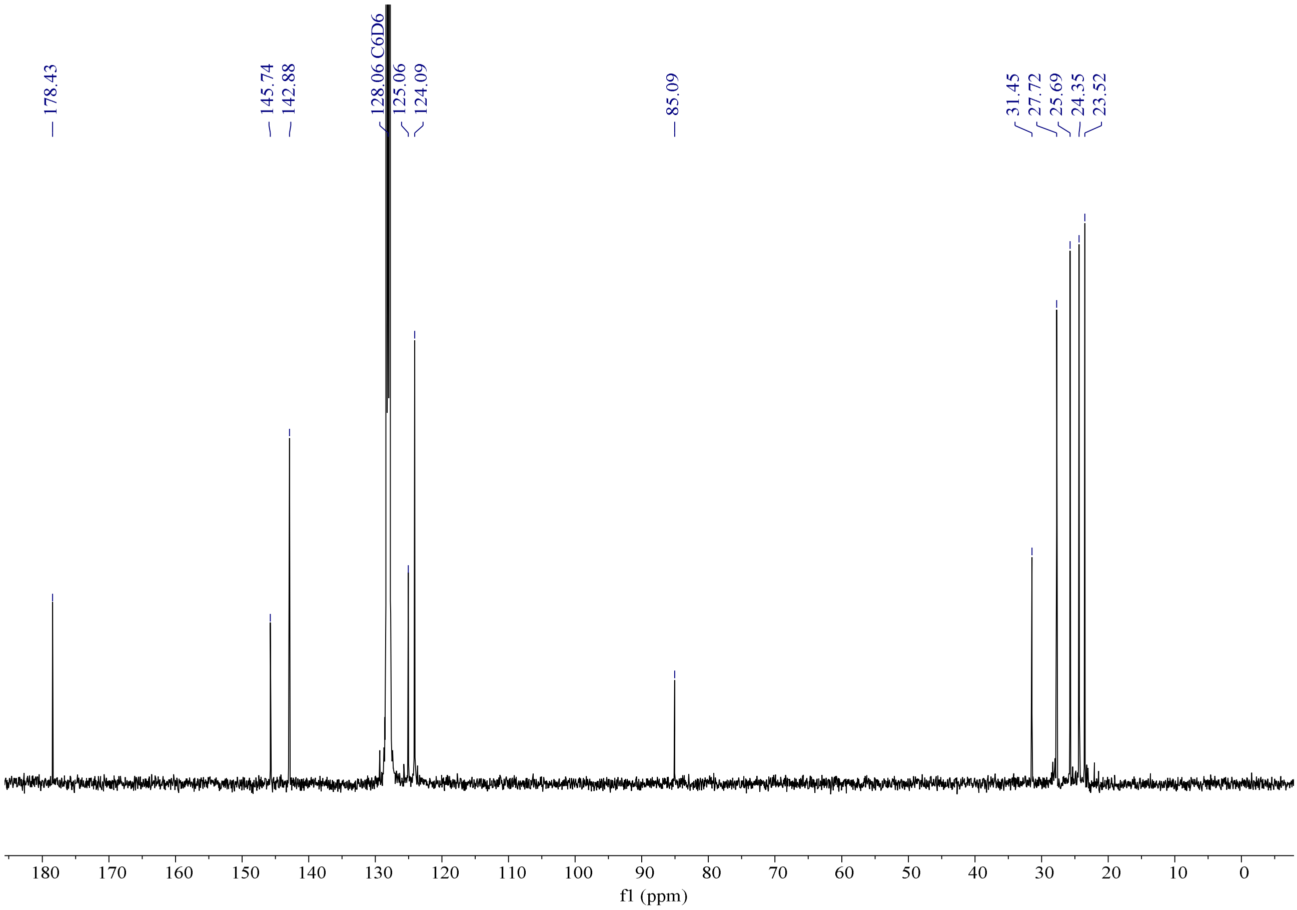

Figure S146. $^{13}C\{^{1}H\}$ NMR spectrum (100.6 MHz, C_6D_6, 295 K) of *in-situ* generated $[\{(^{iPrDip}nacnac)Mg(\mu\text{-}OH)\}_2]$ **3c**.

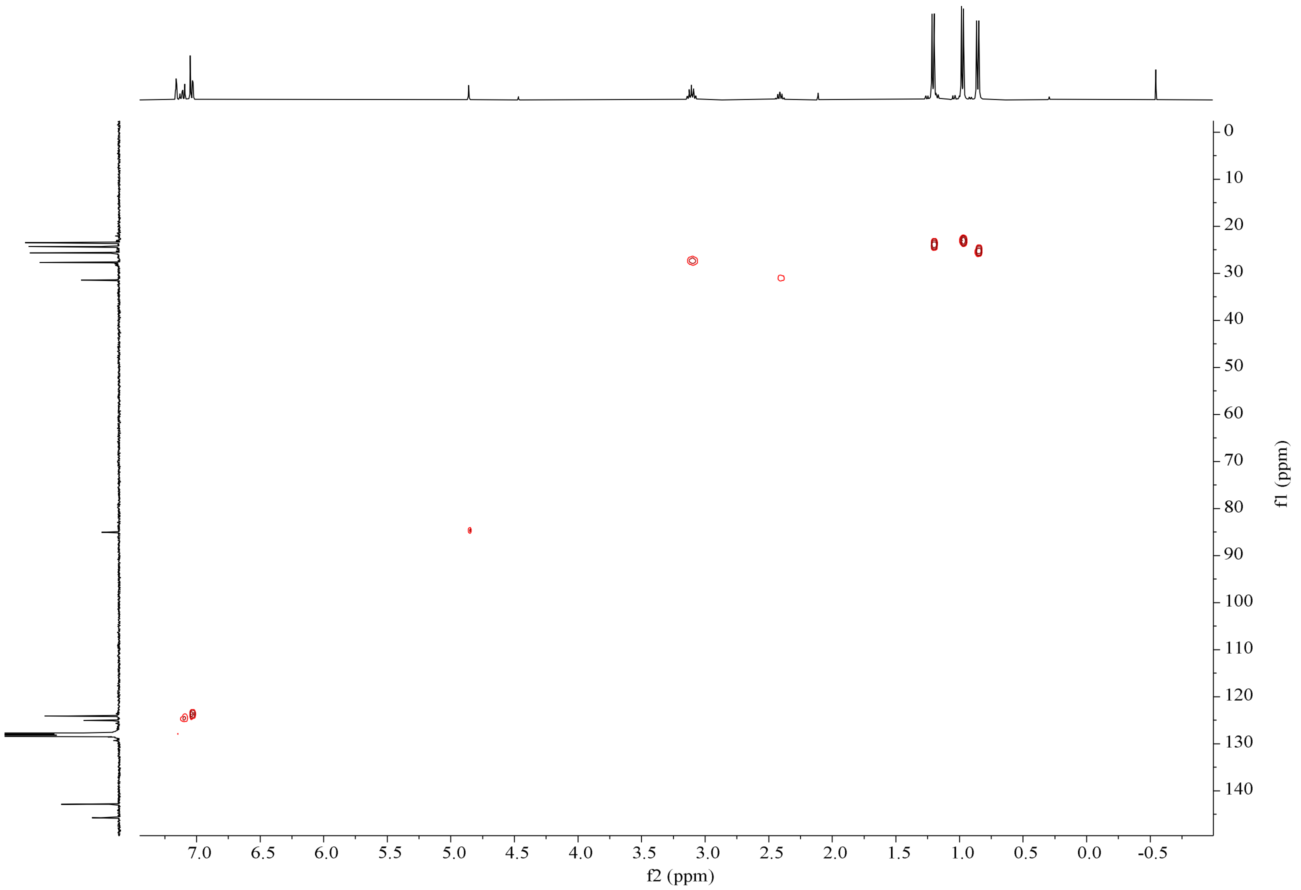

Figure S147. ^{1}H-^{13}C HSQC NMR spectrum of *in-situ* generated [{(^{iPrDip}nacnac)Mg(μ-OH)}$_2$] **3c**.

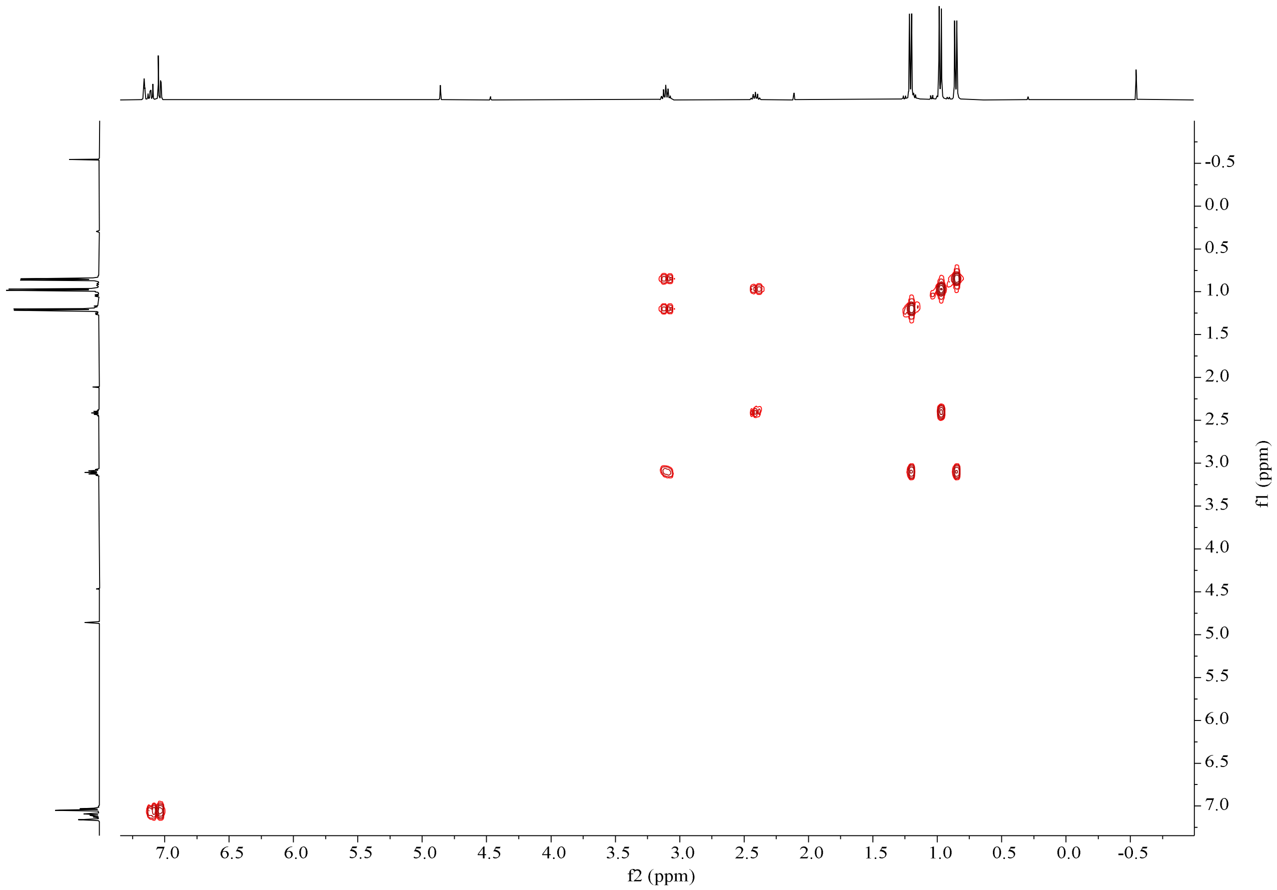

Figure S148. ^1H-^1H COSY NMR spectrum of *in-situ* generated [{(^{iPrDip}nacnac)Mg(μ-OH)}$_2$] **3c**.

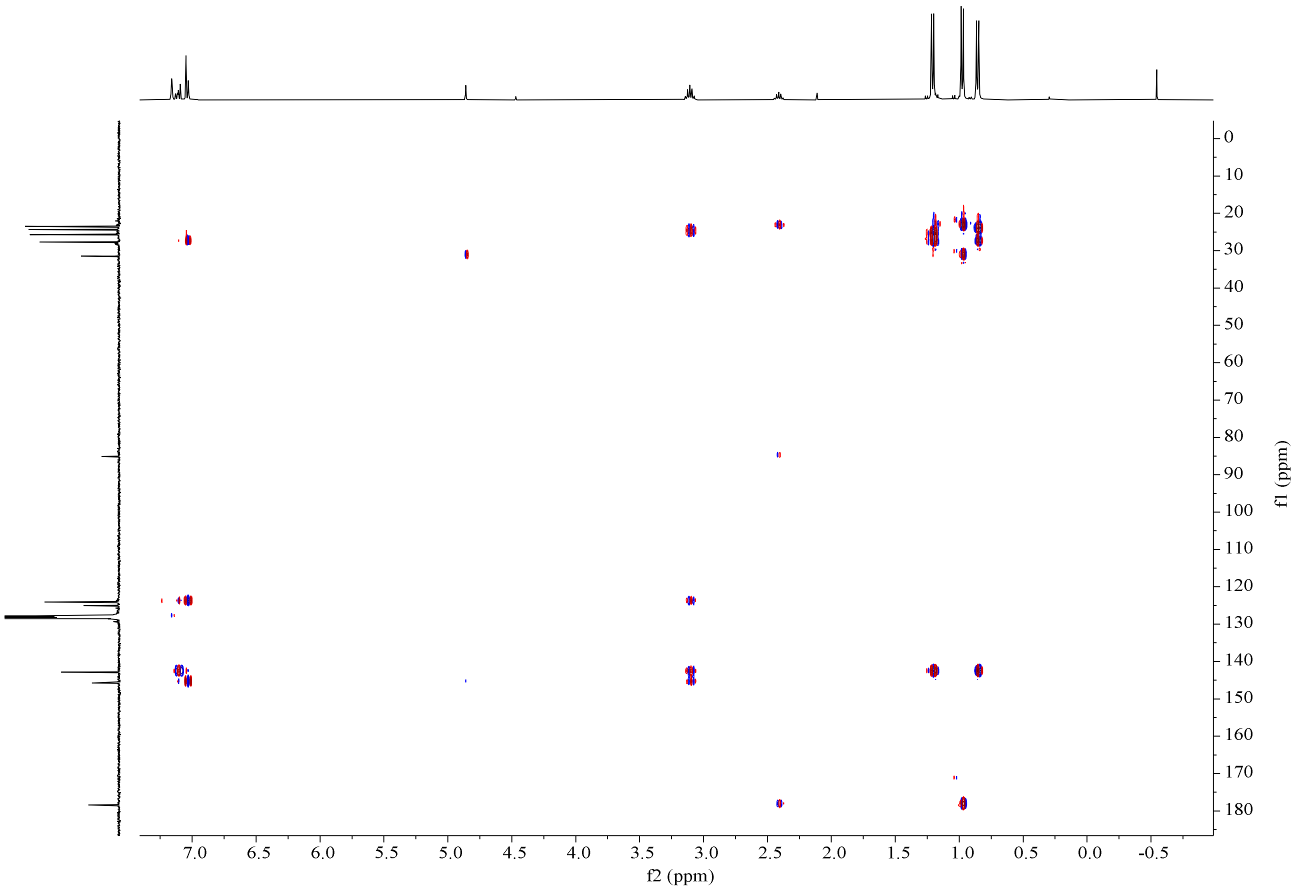

Figure S149. ^{1}H-^{13}C HMBC NMR spectrum of *in-situ* generated [{(^{iPrDip}nacnac)Mg(μ-OH)}$_2$] **3c**.

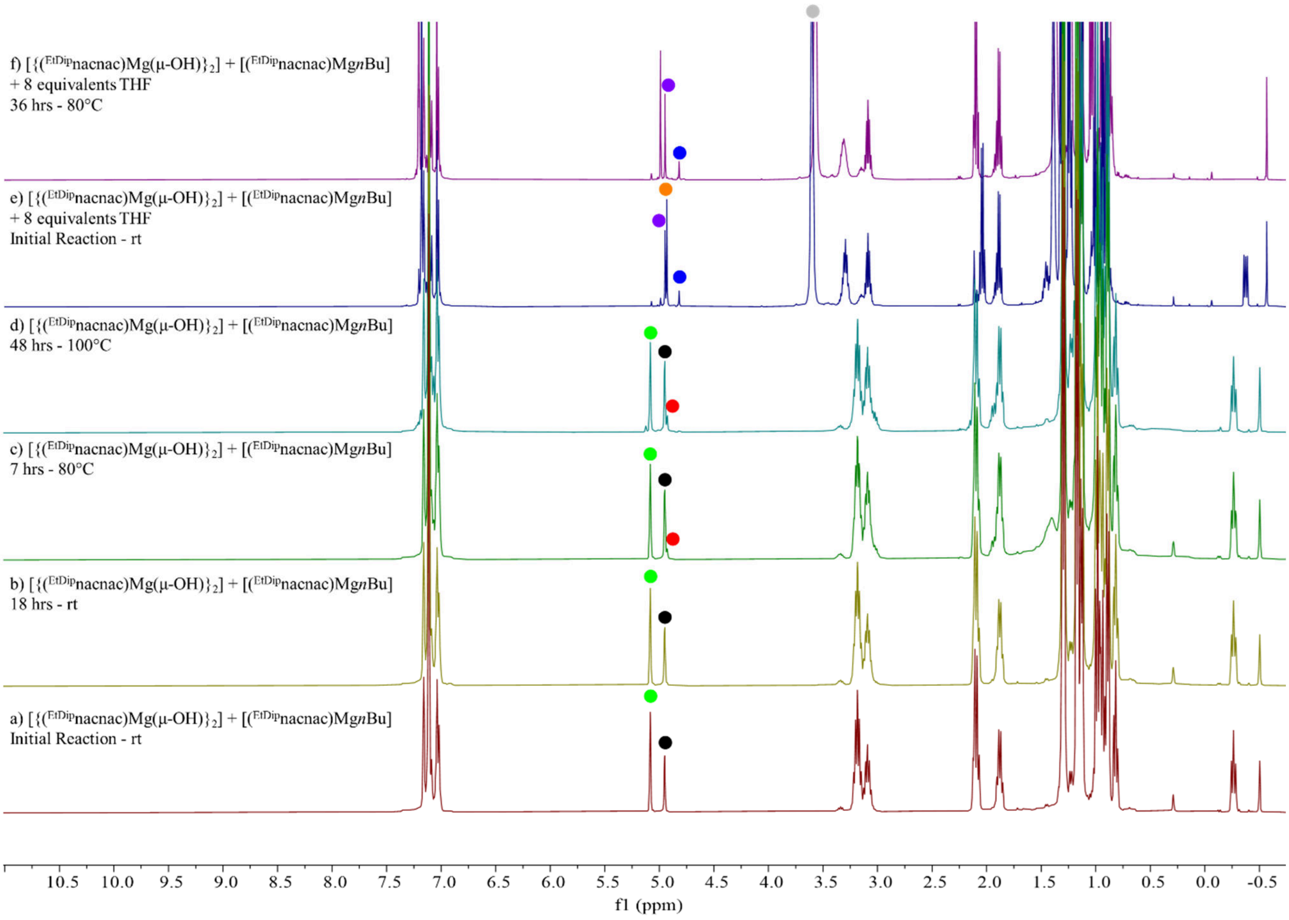

Figure S150. Stacked ^{1}H NMR spectra of the reaction of a colourless solution of [{(^{EtDip}nacnac)Mg(μ-OH)}$_2$] **3b** (20.0 mg, 20.5 μmol, 1.0 equiv.) and [(^{EtDip}nacnac)MgnBu] **9b** (21.6 mg, 41.1 μmol, 2.0 equiv.) and THF (13.4 μL, 164 μmol, 8.0 equiv.) in C_6D_6 (0.6 mL) in a J Young NMR tube. The black circle denotes the resonance associated with the backbone-C*H* of [{(^{EtDip}nacnac)Mg(μ-OH)}$_2$] **3b**. The green circle denotes the resonance associated with the backbone-C*H* of [(^{EtDip}nacnac)MgnBu] **9b**. The red circle denotes the resonance associated with the backbone-C*H* of [{(^{EtDip}nacnac)Mg}$_2$(μ-O)] **1b**. The purple circle denotes the resonance associated with the backbone-C*H* of [{(^{EtDip}nacnac)Mg(THF)(μ-OH)}$_2$]. The orange circle denotes the resonance associated with the backbone-C*H* of [(^{EtDip}nacnac)Mg(THF)nBu]. The blue circle denotes the resonance associated with the backbone-C*H* of [{(^{EtDip}nacnac)Mg(THF)}$_2$(μ-O)] **5b**. The grey circle denotes the resonance associated with O(CH_2) of THF. Spectra a), b), c) and d): 400.1 MHz, C_6D_6, 298 K. Spectra e) and f): 499.9 MHz, C_6D_6, 298 K.

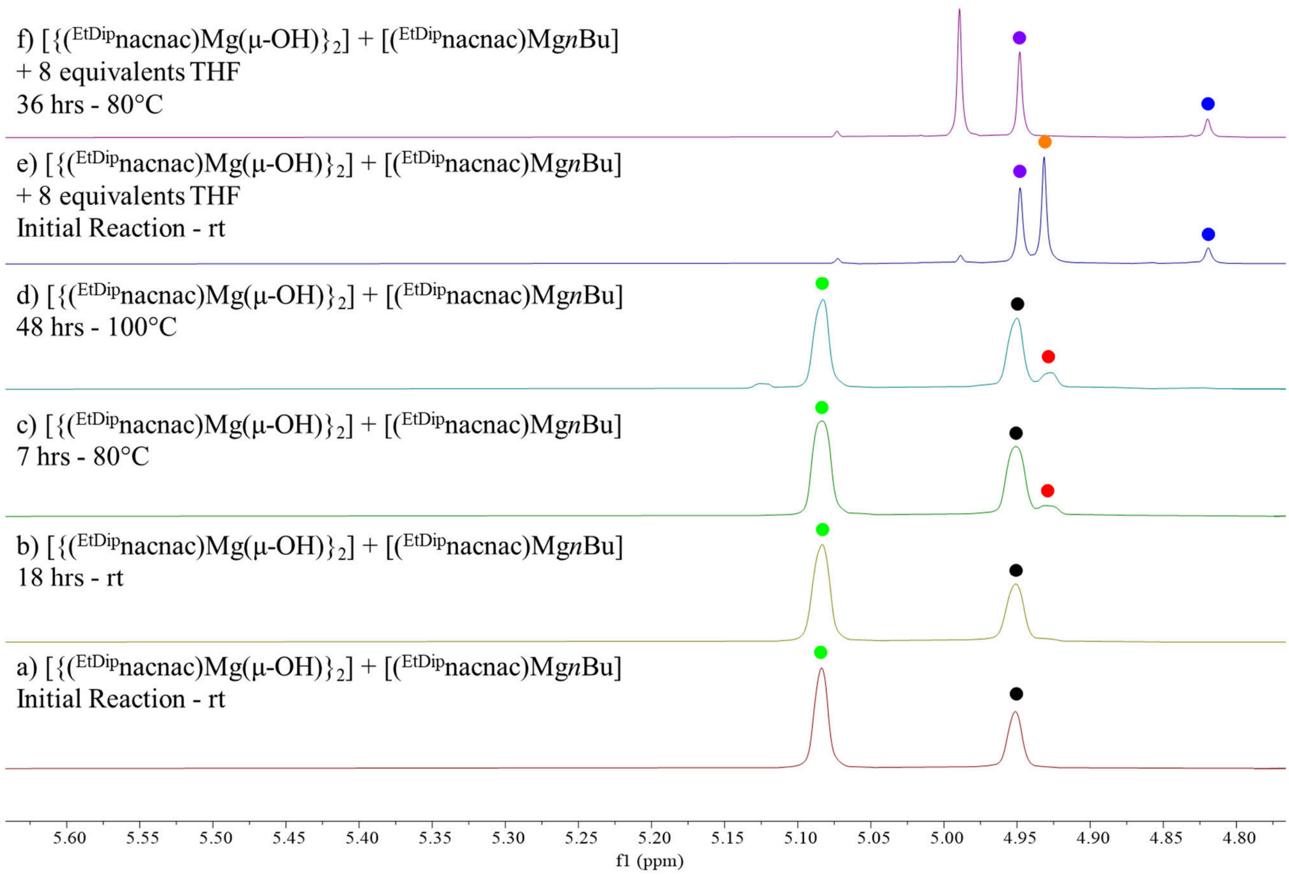

Figure S151. Stacked ^{1}H NMR spectra of the reaction of a colourless solution of [{(^{EtDip}nacnac)Mg(μ-OH)}$_2$] **3b** (20.0 mg, 20.5 μmol, 1.0 equiv.) and [(^{EtDip}nacnac)Mg*n*Bu] **9b** (21.6 mg, 41.1 μmol, 2.0 equiv.) and THF (13.4 μL, 164 μmol, 8.0 equiv.) in C_6D_6 (0.6 mL) in a J Young NMR tube (chemical range: 4.80-5.60 ppm). The black circle denotes the resonance associated with the backbone-C*H* of [{(^{EtDip}nacnac)Mg(μ-OH)}$_2$] **3b**. The green circle denotes the resonance associated with the backbone-C*H* of [(^{EtDip}nacnac)Mg*n*Bu] **9b**. The red circle denotes the resonance associated with the backbone-C*H* of [{(^{EtDip}nacnac)Mg}$_2$(μ-O)] **1b**. The purple circle denotes the resonance associated with the backbone-C*H* of [{(^{EtDip}nacnac)Mg(THF)(μ-OH)}$_2$]. The orange circle denotes the resonance associated with the backbone-C*H* of [(^{EtDip}nacnac)Mg(THF)*n*Bu]. The blue circle denotes the resonance associated with the backbone-C*H* of [{(^{EtDip}nacnac)Mg(THF)}$_2$(μ-O)] **5b**. Spectra a), b), c) and d): 400.1 MHz, C_6D_6, 298 K. Spectra e) and f): 499.9 MHz, C_6D_6, 298 K.

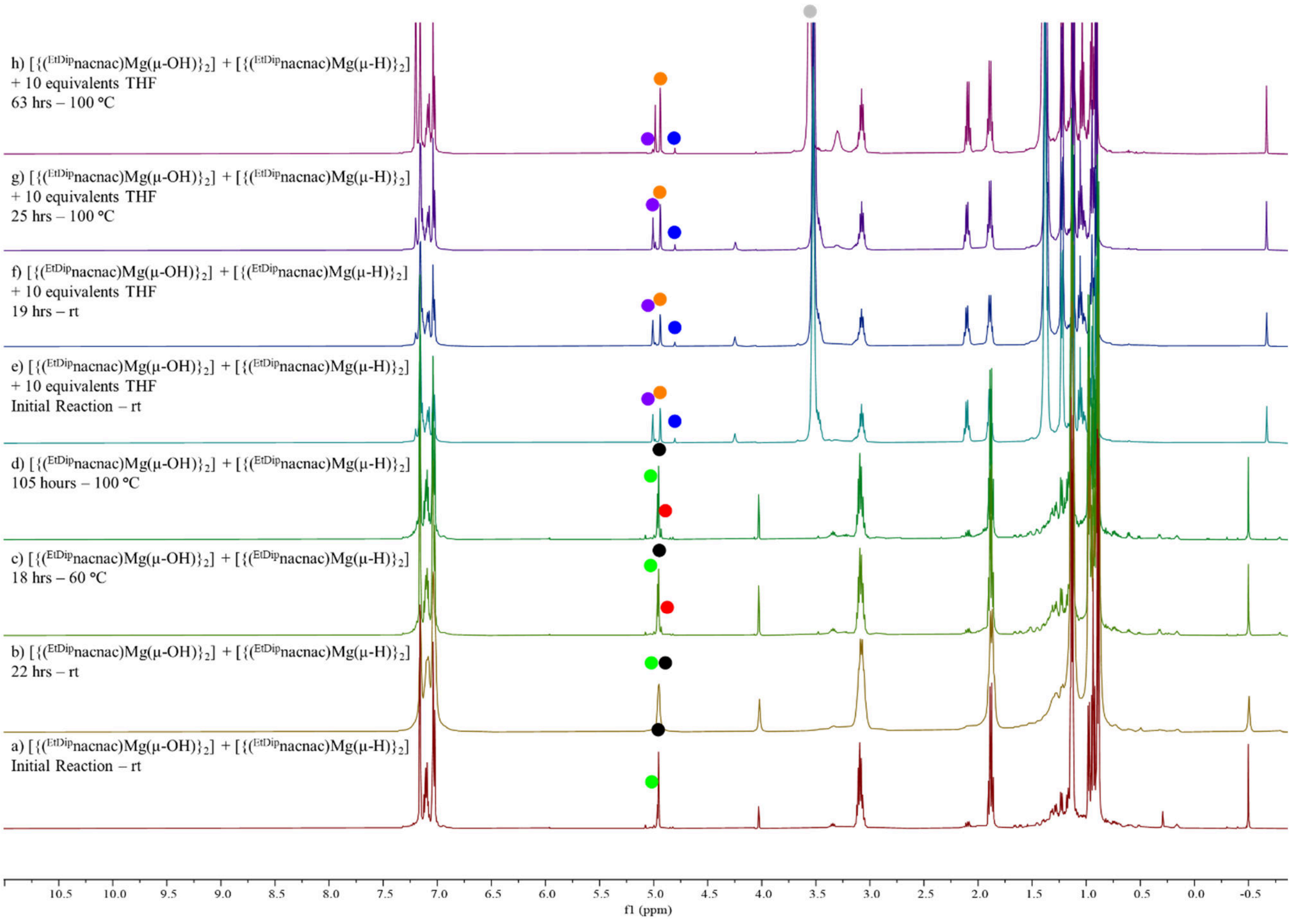

Figure S152. Stacked ^{1}H NMR (500.1 MHz, C_6D_6, 298 K) spectra of the reaction of a colourless solution of [{(^{EtDip}nacnac)Mg(μ-OH)}$_2$] **3b** (22.0 mg, 22.6 μmol, 1.0 equiv.) and [{(^{EtDip}nacnac)Mg(μ-H)}$_2$] **7b** (23.4 mg, 24.8 μmol, 1.1 equiv.) and THF (18.4 μL, 227 μmol, 10.0 equiv.) in C_6D_6 (0.6 mL) in a J Young NMR tube. The green circle denotes the resonance associated with the backbone-C*H* of [{(^{EtDip}nacnac)Mg(μ-H)}$_2$] **7b**. The black circle denotes the resonance associated with the backbone-C*H* of [{(^{EtDip}nacnac)Mg(μ-OH)}$_2$] **3b**. The red circle denotes the resonance associated with the backbone-C*H* of [{(^{EtDip}nacnac)Mg}$_2$(μ-O)] **1b**. The purple circle denotes the resonance associated with the backbone-C*H* of [{(^{EtDip}nacnac)Mg(THF)(μ-OH)}$_2$]. The orange circle denotes the resonance associated with the backbone-C*H* of [{(^{EtDip}nacnac)Mg(THF)(μ-H)}$_2$] **10b**. The blue circle denotes the resonance associated with the backbone-C*H* of [{(^{EtDip}nacnac)Mg(THF)}$_2$(μ-O)] **5b**. The grey circle denotes the resonance associated with O(CH_2) of THF.

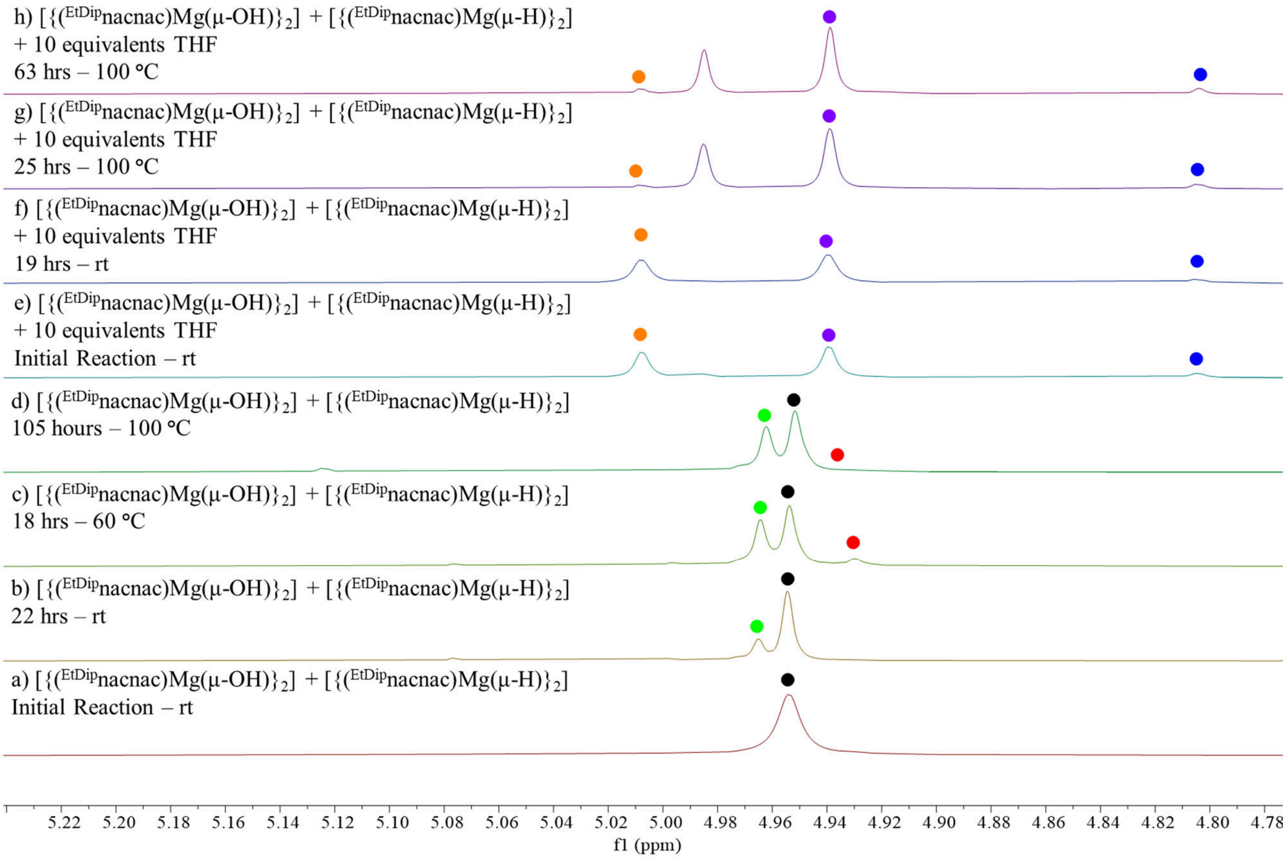

Figure S153. Stacked ^{1}H NMR spectra of the reaction of a colourless solution of [{(^{EtDip}nacnac)Mg(μ-OH)}$_2$] **3b** (22.0 mg, 22.6 μmol, 1.0 equiv.) and [{(^{EtDip}nacnac)Mg(μ-H)}$_2$] **7b** (23.4 mg, 24.8 μmol, 1.1 equiv.) and THF (18.4 μL, 227 μmol, 10.0 equiv.) in C_6D_6 (0.6 mL) in a J Young NMR tube (chemical range 4.78-5.22 ppm). The green circle denotes the resonance associated with the backbone-C*H* of [{(^{EtDip}nacnac)Mg(μ-H)}$_2$] **7b**. The black circle denotes the resonance associated with the backbone-C*H* of [{(^{EtDip}nacnac)Mg(μ-OH)}$_2$] **3b**. The red circle denotes the resonance associated with the backbone-C*H* of [{(^{EtDip}nacnac)Mg}$_2$(μ-O)] **1b**. The purple circle denotes the resonance associated with the backbone-C*H* of [{(^{EtDip}nacnac)Mg(THF)(μ-OH)}$_2$]. The orange circle denotes the resonance associated with the backbone-C*H* of [{(^{EtDip}nacnac)Mg(THF)(μ-H)}$_2$] **10b**. The blue circle denotes the resonance associated with the backbone-C*H* of [{(^{EtDip}nacnac)Mg(THF)}$_2$(μ-O)] **5b**.

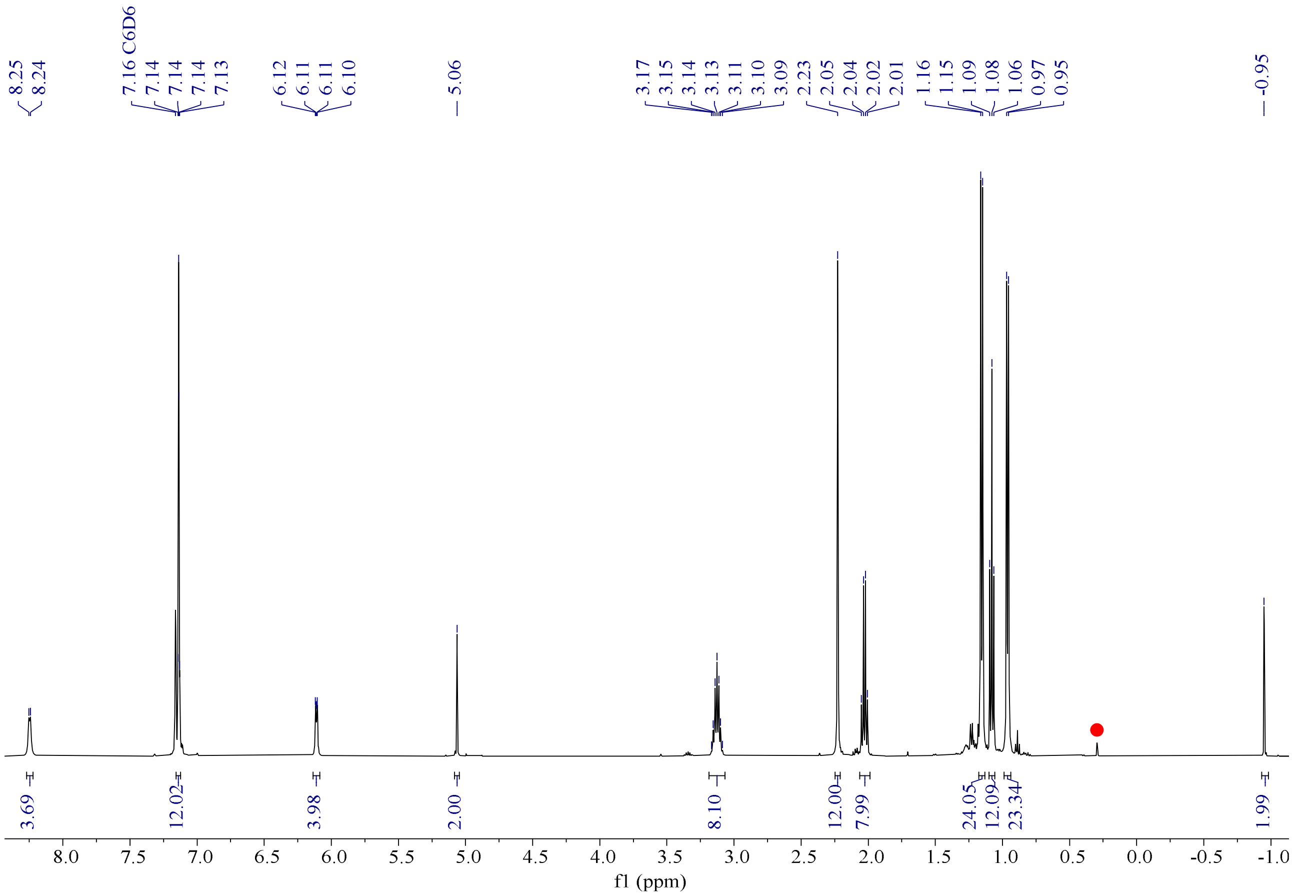

Figure S154. ^{1}H NMR spectrum (499.9 MHz, C_6D_6, 298 K) of *in-situ* prepared [{(^{EtDip}nacnac)Mg(DMAP)(μ-OH)}$_2$] **8b**. The red circle denotes the resonance associated with silicone grease.

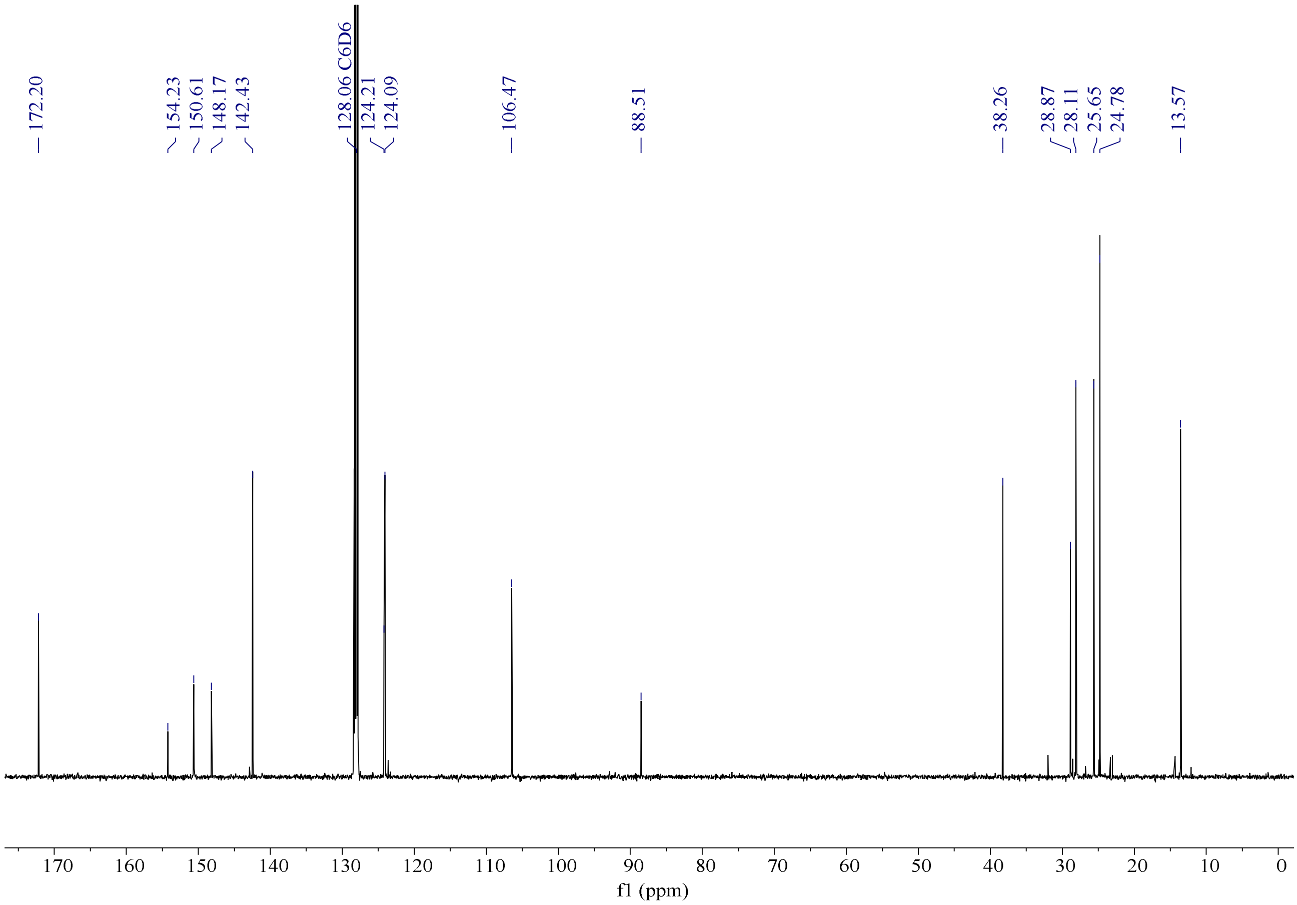

Figure S155. $^{13}C\{^{1}H\}$ NMR spectrum (125.7 MHz, C_6D_6, 298 K) of *in-situ* prepared [{(^{EtDip}nacnac)Mg(DMAP)(μ-OH)}$_2$] **8b**.

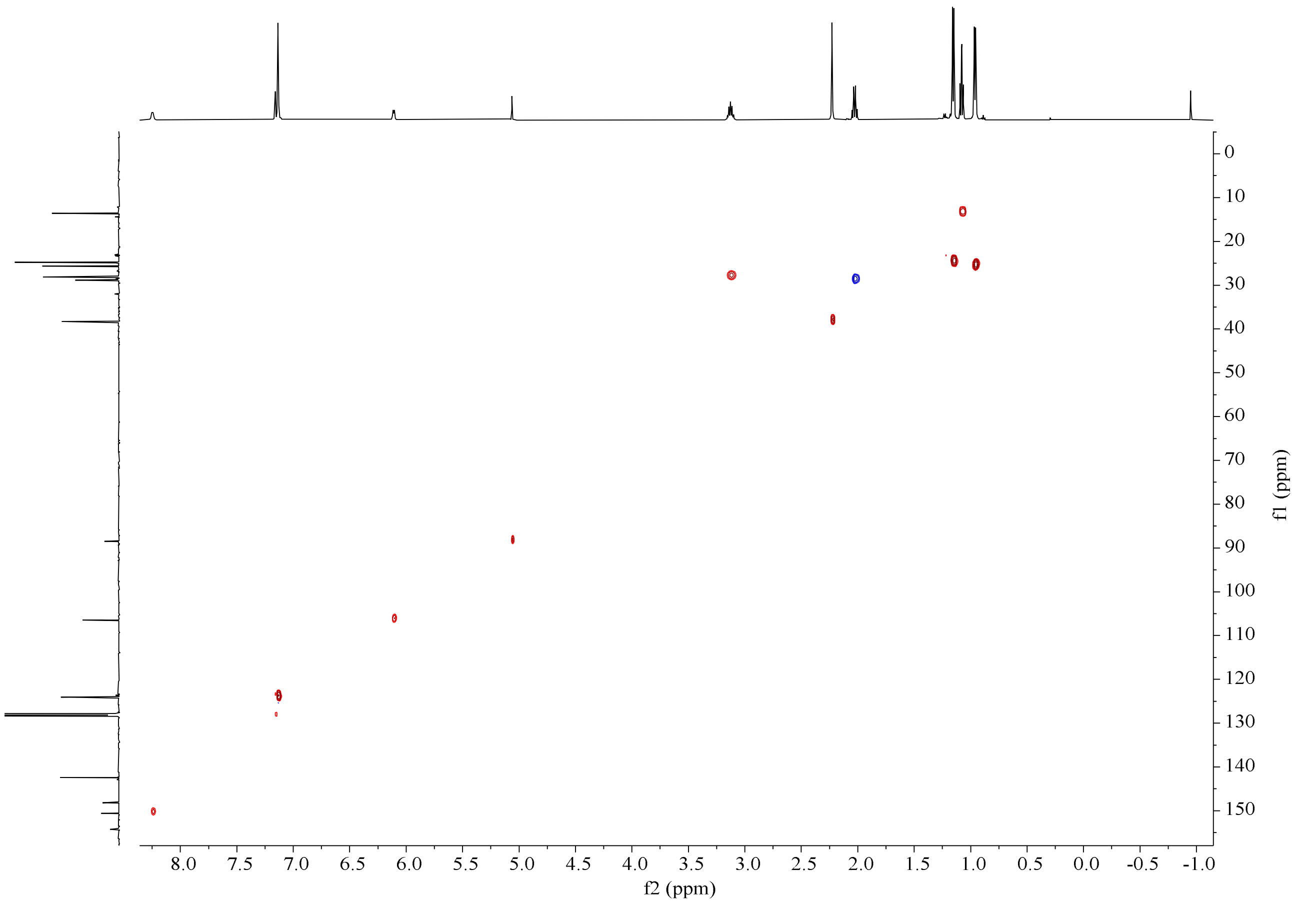

Figure S156. ^{1}H-^{13}C HSQC NMR spectrum of *in-situ* prepared [{(^{EtDip}nacnac)Mg(DMAP)(μ-OH)}$_2$] **8b**.

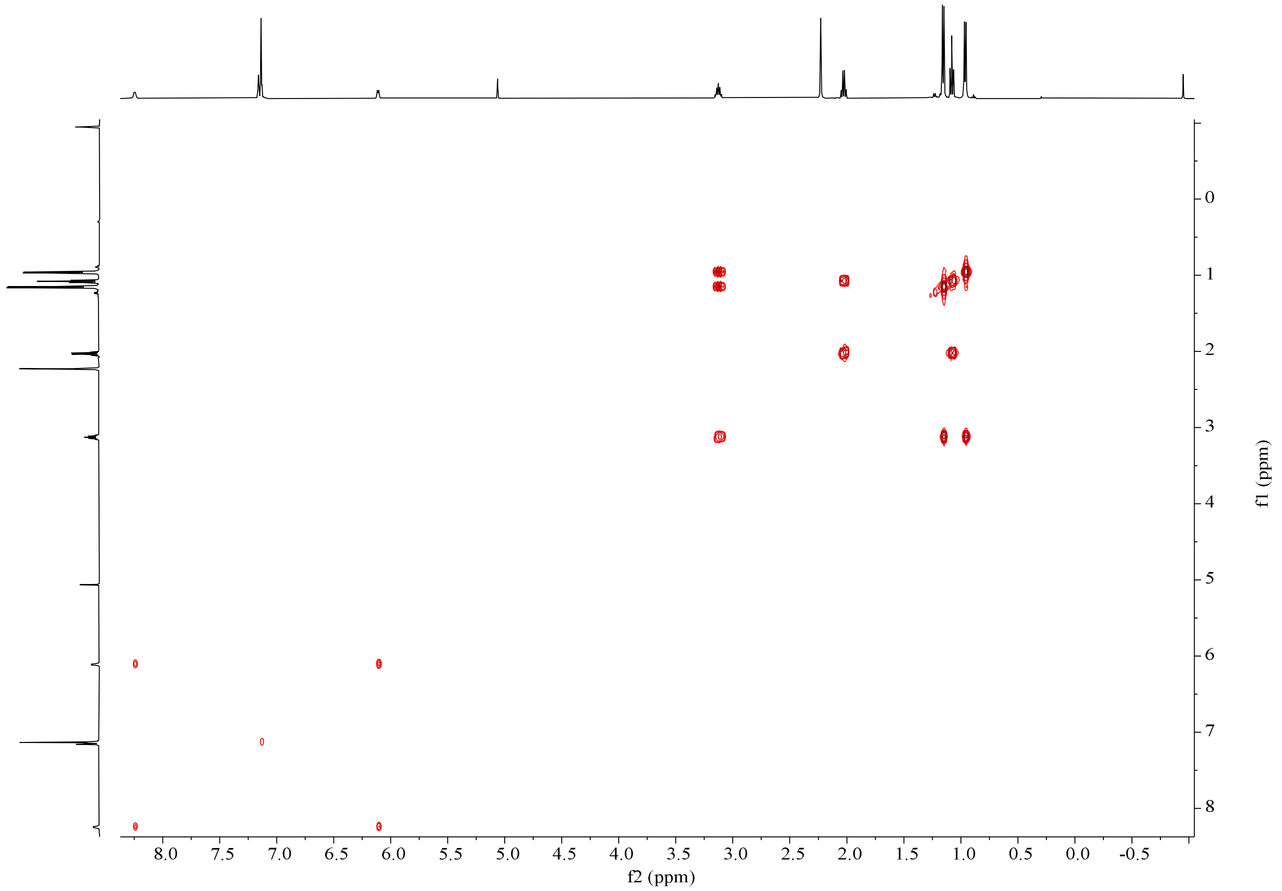

Figure S157. ^1H-^1H COSY NMR spectrum of *in-situ* prepared [{(^{EtDip}nacnac)Mg(DMAP)(μ-OH)}$_2$] **8b**.

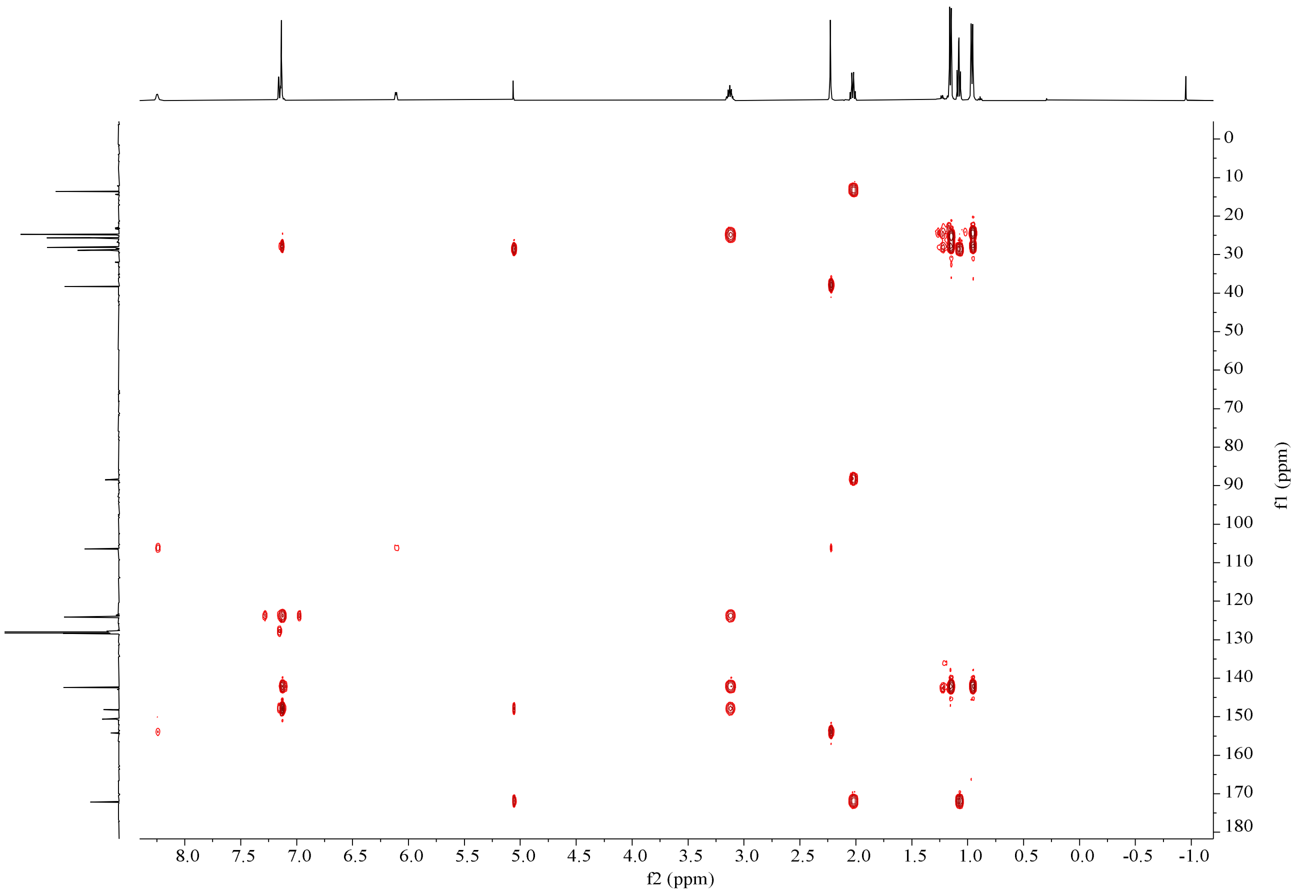

Figure S158. ^{1}H-^{13}C HMBC NMR spectrum of *in-situ* prepared [{(^{EtDip}nacnac)Mg(DMAP)(μ-OH)}$_2$] **8b**.

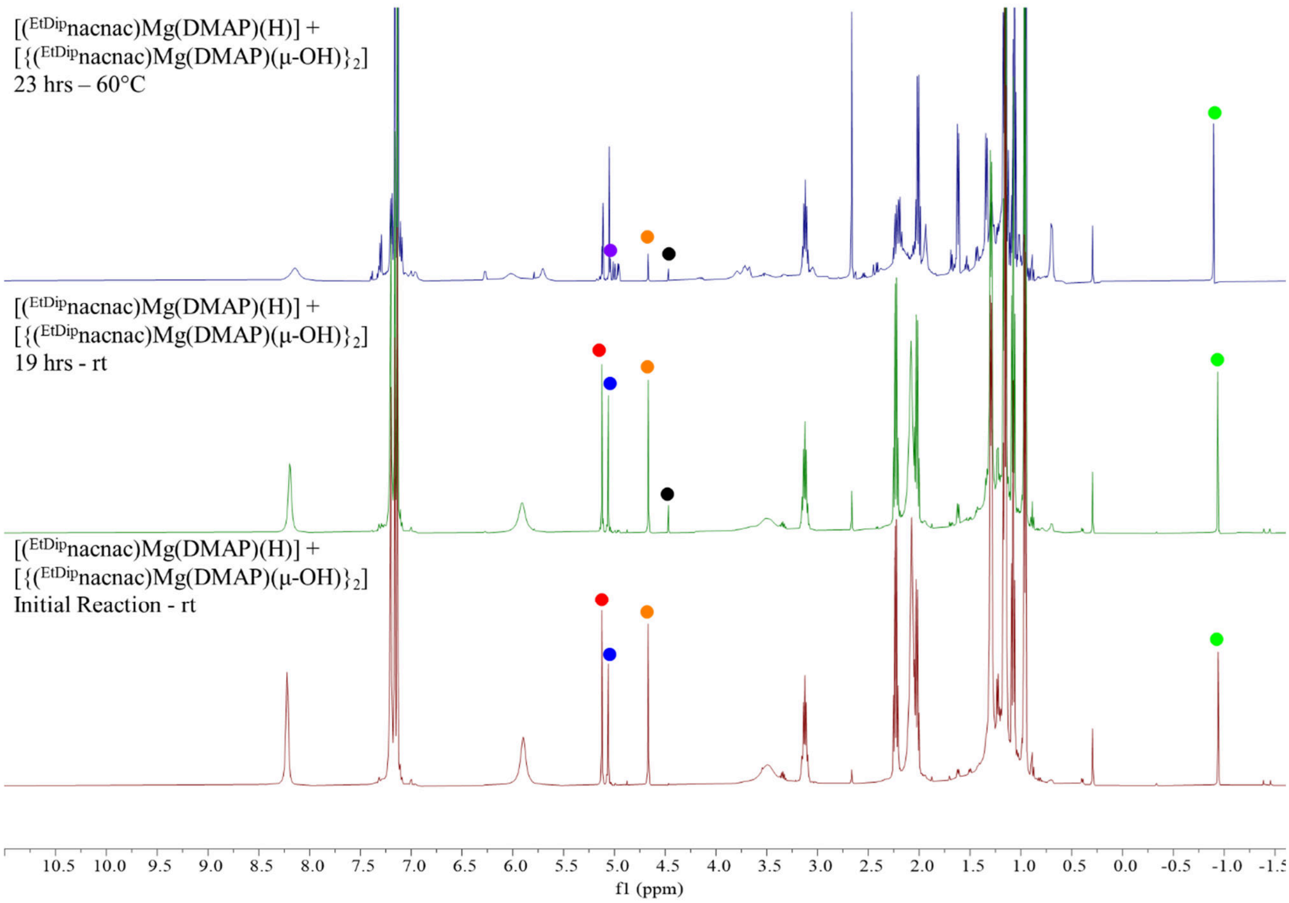

Figure S159. Stacked ^{1}H NMR spectra (499.9 MHz, C_6D_6, 298 K) of the reaction of a colourless *in-situ* prepared solution of [{(^{EtDip}nacnac)Mg(DMAP)(μ-OH)}$_2$] **8b** (15.2 mg, 12.5 μmol, 1.0 equiv.) and a colourless *in-situ* prepared solution of [(^{EtDip}nacnac)Mg(DMAP)H] **12b** (15.6 mg, 26.3 μmol, 2.1 equiv.) in C_6D_6 (0.8 mL) in a J Young NMR tube. The black circle denotes the resonance associated with dihydrogen. The red circle denotes the resonance associated with the backbone-C*H* of [(^{EtDip}nacnac)Mg(DMAP)H] **12b**. The orange circle denotes the resonance associated with Mg-*H* of [(^{EtDip}nacnac)Mg(DMAP)H] **12b**. The blue circle denotes the resonance associated with the backbone-C*H* of [{(^{EtDip}nacnac)Mg(DMAP)(μ-OH)}$_2$] **8b**. The green circle denotes the resonance associated with Mg-O*H* of [{(^{EtDip}nacnac)Mg(DMAP)(μ-OH)}$_2$] **8b**. The purple circle denotes the resonance associated with the backbone-C*H* of [{(^{EtDip}nacnac)Mg(DMAP)}$_2$(μ-O)] **6b**.

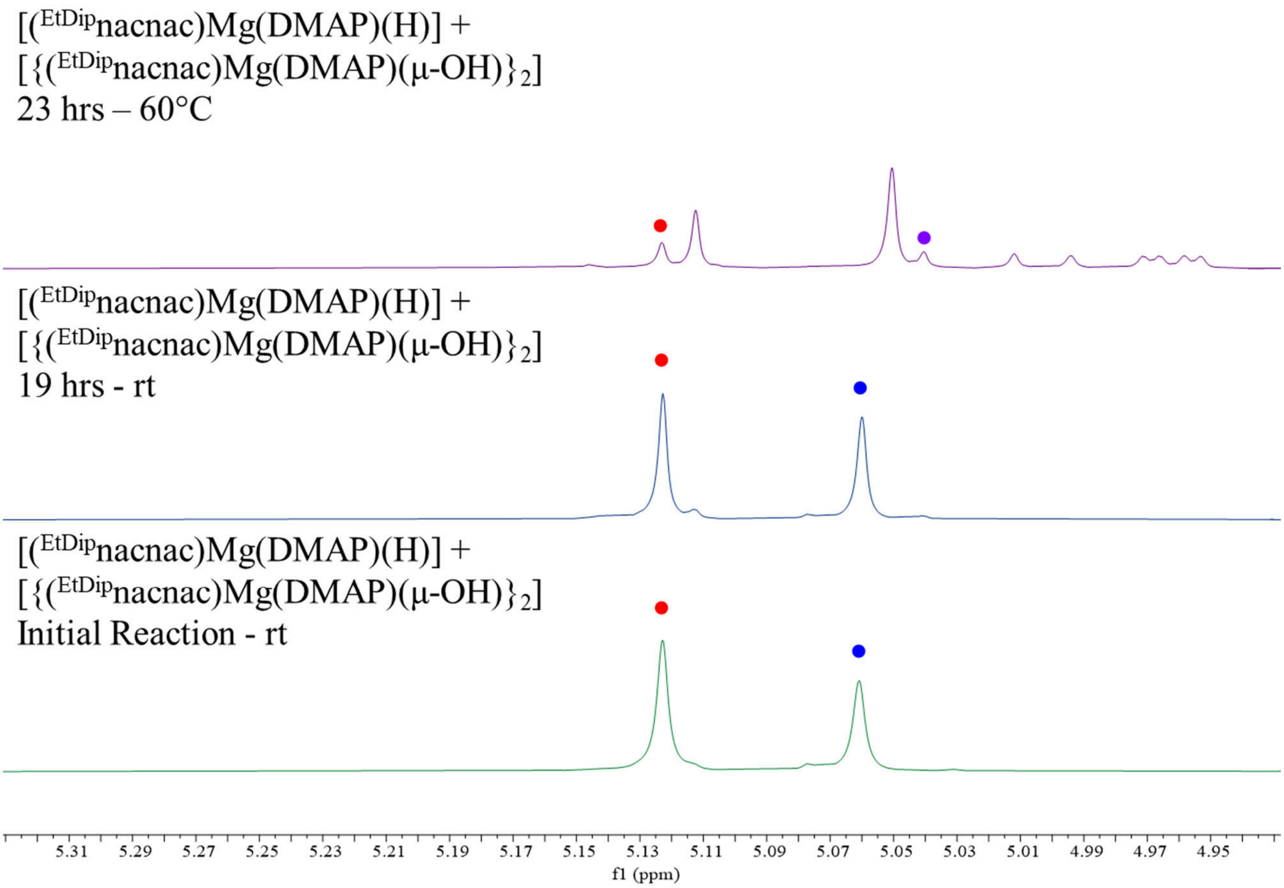

Figure S160. Stacked ^{1}H NMR spectra (499.9 MHz, C_6D_6, 298 K)) of the reaction of a colourless *in-situ* generated solution of [{(^{EtDip}nacnac)Mg(DMAP)(μ-OH)}$_2$] **8b** (15.2 mg, 12.5 μmol, 1.0 equiv.) and a colourless *in-situ* generated solution of [(^{EtDip}nacnac)Mg(DMAP)H] **12b** (15.6 mg, 26.3 μmol, 2.1 equiv.) in C_6D_6 (0.8 mL) in a J Young NMR tube (chemical range: 4.95-5.31 ppm). The red circle denotes the resonance associated with the backbone-C*H* of [(^{EtDip}nacnac)Mg(DMAP)H] **12b**. The blue circle denotes the resonance associated with the backbone-C*H* of [{(^{EtDip}nacnac)Mg(DMAP)(μ-OH)}$_2$] **8b**. The purple circle denotes the resonance associated with the backbone-C*H* of [{(^{EtDip}nacnac)Mg(DMAP)}$_2$(μ-O)] **6b**.

2.7 Hydrolysis experiments

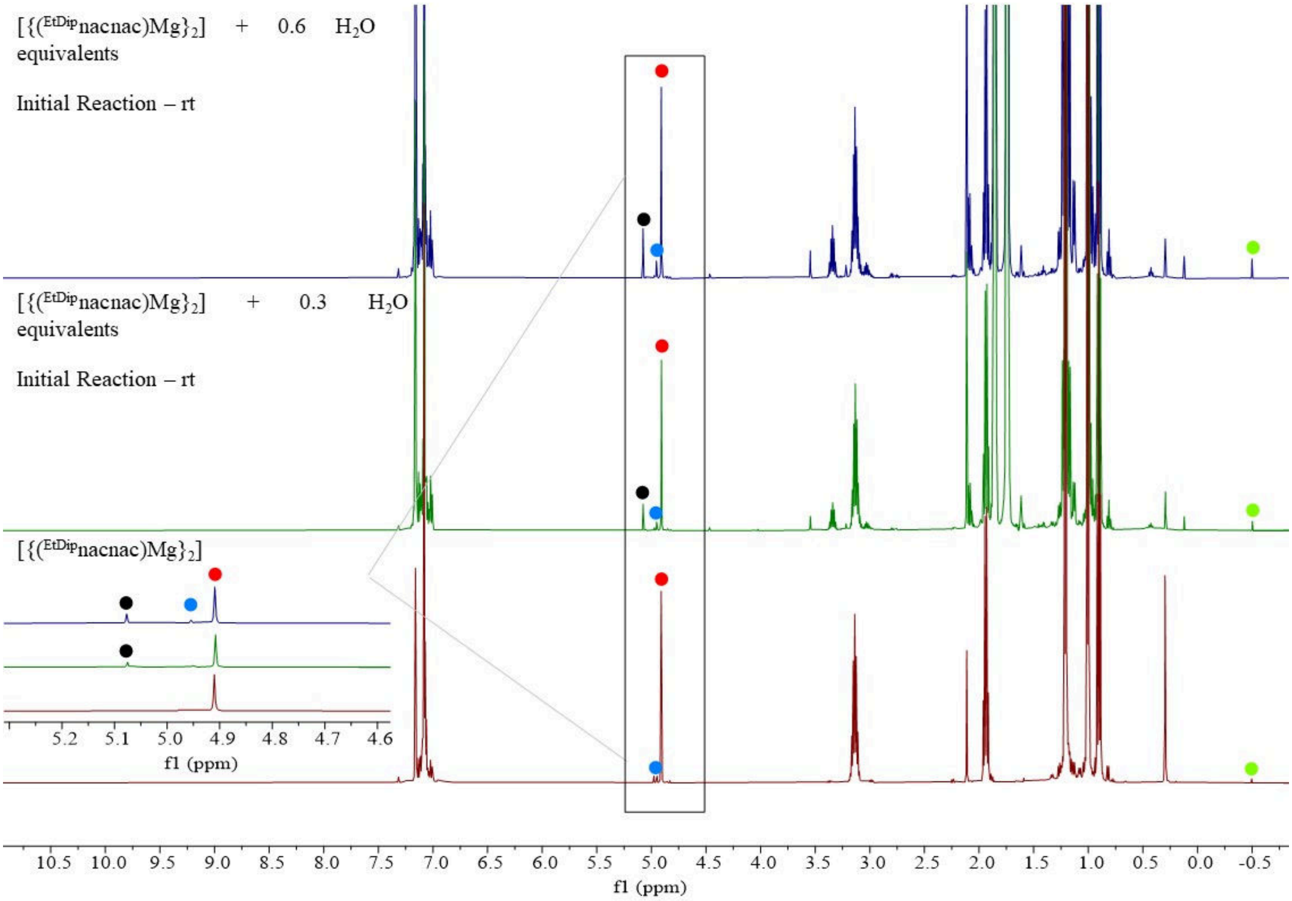

Figure S161. Stacked ^1H spectra (499.9 MHz, C_6D_6, 298 K) of the reaction of a yellow solution of [{(^{EtDip}nacnac)Mg}$_2$] **2b** (10.7 mg, 11.4 µmol, 1.0 equiv.) in C_6D_6 (0.6 mL) and sub-stoichiometric amounts of water (from a 6.4 mM water stock solution in C_6D_6) at room temperature (rt) in a J Young NMR tube. The red circle denotes the resonance associated with the backbone-C*H* of [{(^{EtDip}nacnac)Mg}$_2$] **2b**. The blue circle denotes the resonance associated with the backbone-C*H* of [{(^{EtDip}nacnac)Mg(µ-OH)}$_2$] **3b**. The green circle denotes the resonance associated with Mg-(O*H*)$_2$ of [{(^{EtDip}nacnac)Mg(µ-OH)}$_2$] **3b**. The black circle denotes the resonance associated with the backbone-C*H* of ^{EtDip}nacnacH.

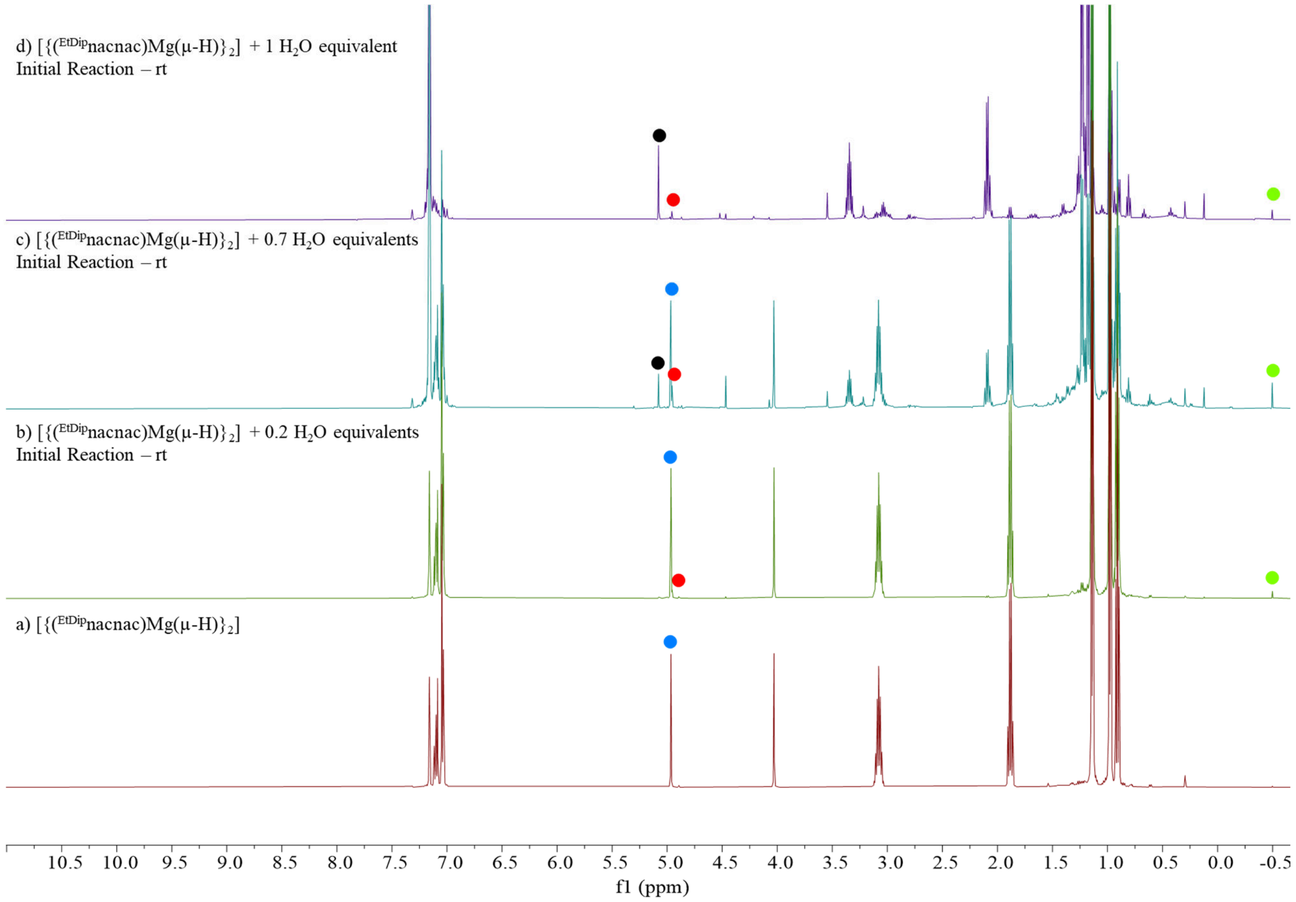

Figure S162. Stacked ^{1}H NMR spectra of the reaction of a colourless solution of [{(^{EtDip}nacnac)Mg(μ-H)}$_2$] **7b** (20.6 mg, 21.9 μmol, 1.0 equiv.) in C_6D_6 (0.6 mL) with sub-stoichiometric amounts of water (from a 6.4 mM water stock solution in C_6D_6) at room temperature (rt) in a J Young NMR tube. The blue circle denotes the resonance associated with the backbone-C*H* of [{(^{EtDip}nacnac)Mg(μ-H)}$_2$] **7b**. The green circle denotes the resonance associated with Mg-(O*H*)$_2$ of [{(^{EtDip}nacnac)Mg(μ-OH)}$_2$] **3b**. The red circle denotes the resonance associated with the backbone-C*H* of [{(^{EtDip}nacnac)Mg(μ-OH)}$_2$] **3b**. The black circle denotes the resonance associated with the backbone-C*H* of ^{EtDip}nacnacH.

2.8 Hydrogenation experiments of DPE

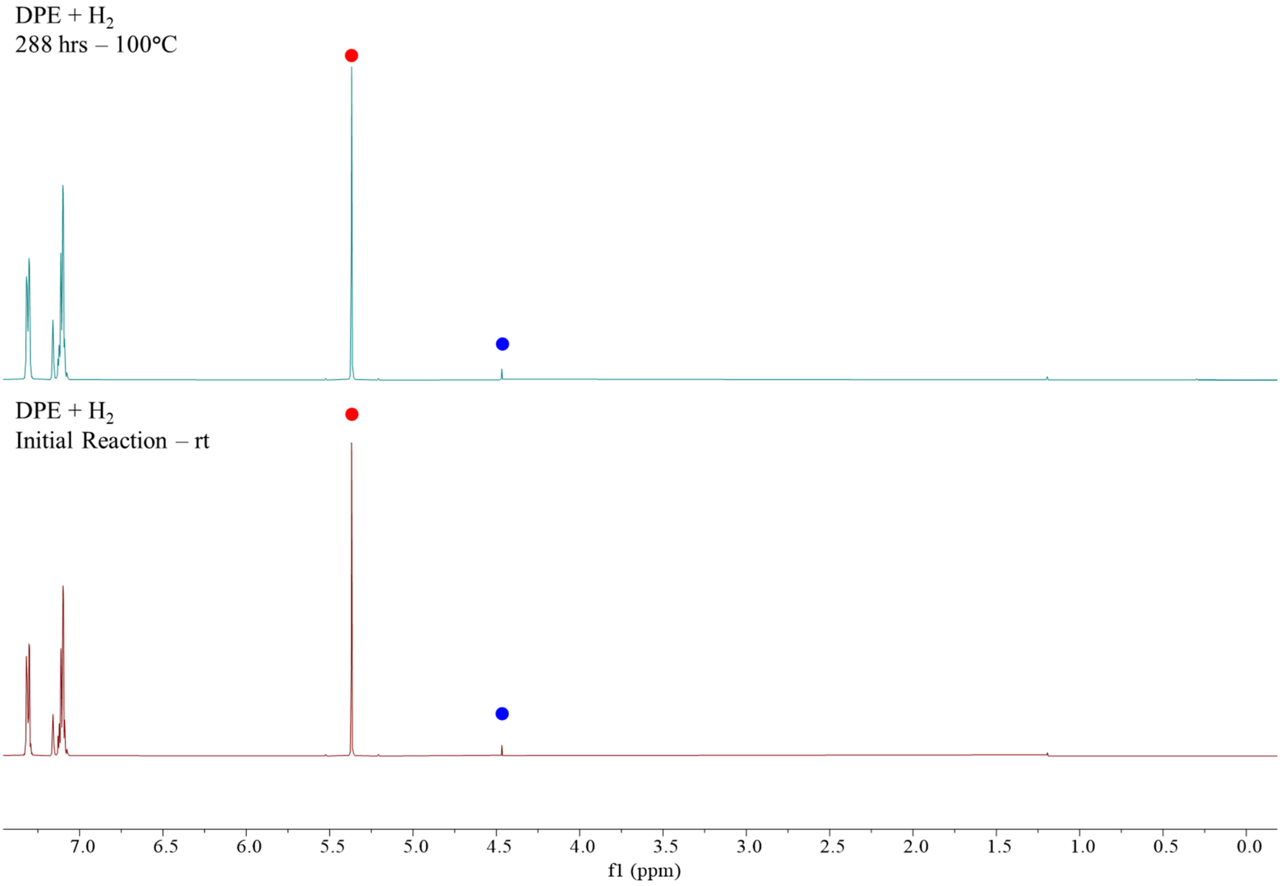

Figure S163. Stacked ^{1}H NMR spectra (499.9 MHz, C_6D_6, 298 K) of the reaction of a solution of DPE (4.0 µL, 23 µmol) and dihydrogen (ca. 1 bar) in C_6D_6 (0.6 mL) at 100°C for 288 hours. The blue circle denotes the resonance associated with dihydrogen. The red circle denotes the resonance associated with Ph_2CCH_2 of DPE.

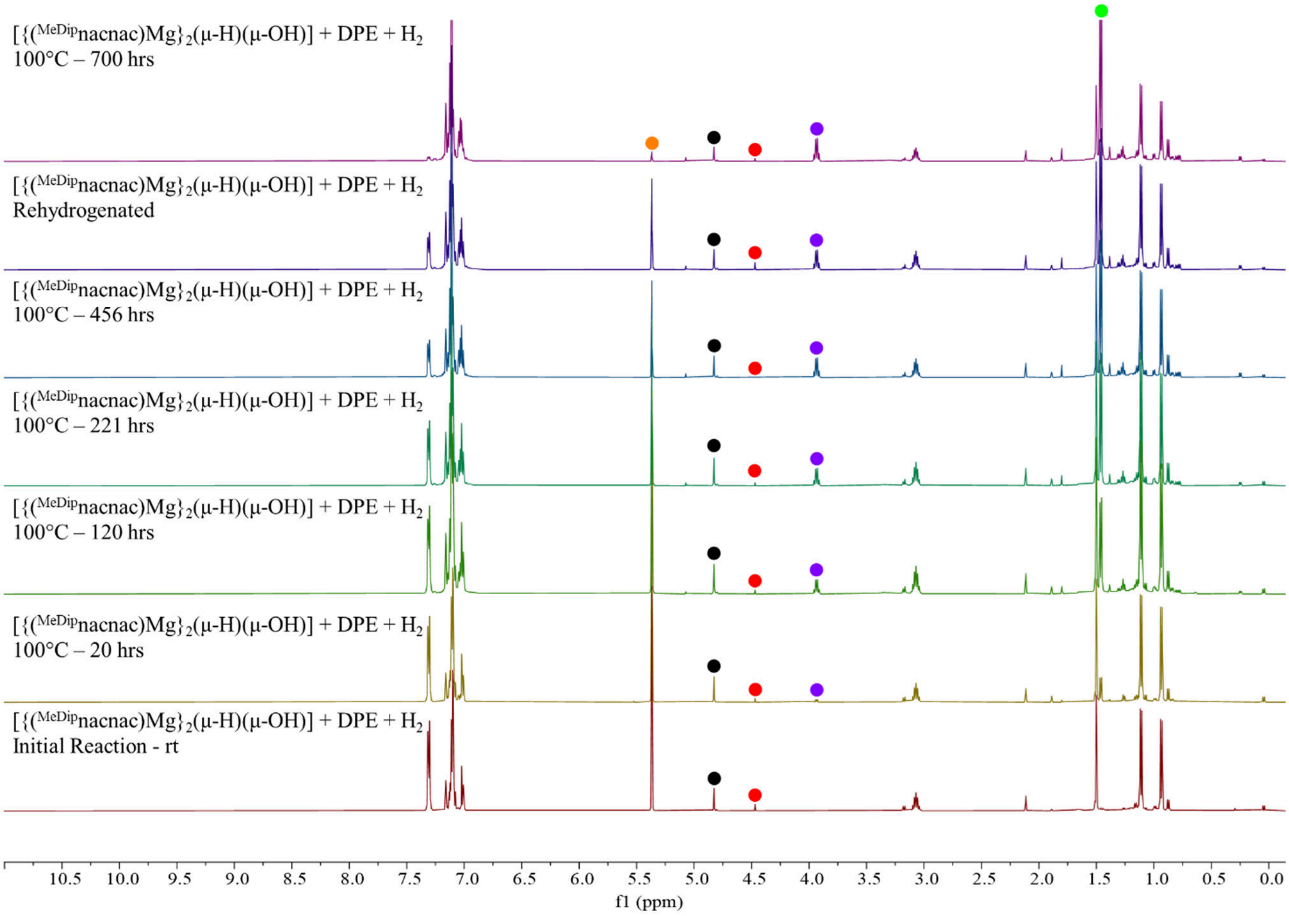

Figure S164. Stacked ^{1}H NMR spectra (499.9 MHz, C_6D_6, 298 K) of the reaction of a colourless *in-situ* generated solution of [{(^{MeDip}nacnac)Mg}$_2$(μ-H)(μ-OH)] **4a** (16.5 mg, 18.3 μmol, 14.0 mol%), DPE (23.1 μL, 131 μmol, 7.1 equiv.) and dihydrogen (ca. 1 bar). The orange circle denotes the resonance associated with $Ph_2CC\mathit{H}_2$ of DPE. The black circle denotes the resonance associated with the backbone-C*H* of [{(^{MeDip}nacnac)Mg}$_2$(μ-H)(μ-OH)] **4a**. The red circle denotes the resonance associated with dihydrogen. The purple circle denotes the resonance associated with $Ph_2C\mathit{H}CH_3$ of 1,1-diphenylethane. The green circle denotes the resonance associated with $Ph_2CHC\mathit{H}_3$ of 1,1-diphenylethane.

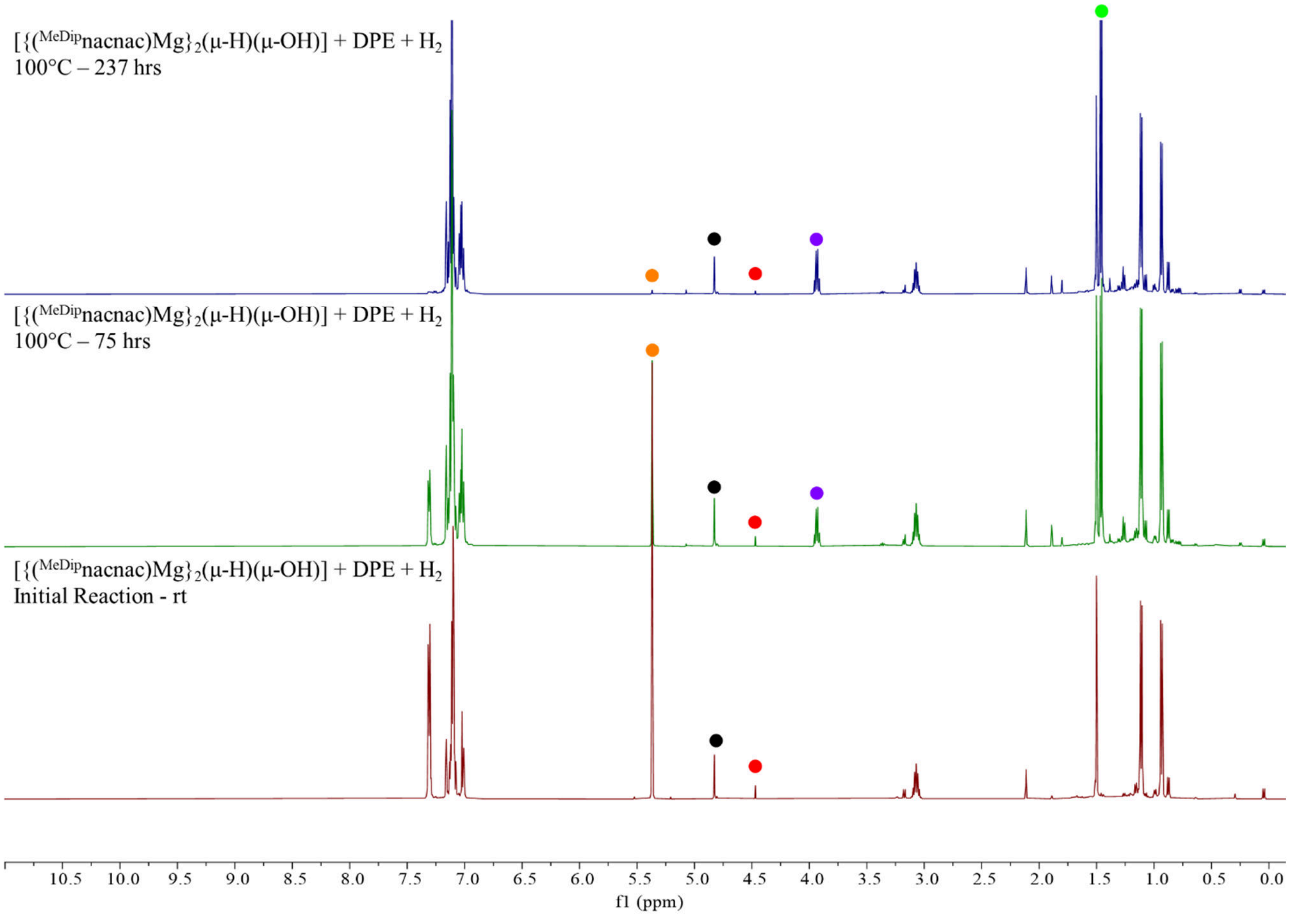

Figure S165. Stacked ^{1}H NMR spectra (499.9 MHz, C_6D_6, 298 K) of the reaction of a colourless *in-situ* generated solution of [{(^{MeDip}nacnac)Mg}$_2$(μ-H)(μ-OH)] **4a** (15.2 mg, 16.9 μmol, 12.6 mol%), DPE (23.6 μL, 133.8 μmol, 7.9 equiv.) and dihydrogen at higher pressure (ca. 2-3 bar). The orange circle denotes the resonance associated with Ph_2CCH_2 of DPE. The black circle denotes the resonance associated with the backbone-C*H* of [{(^{MeDip}nacnac)Mg}$_2$(μ-H)(μ-OH)] **4a**. The red circle denotes the resonance associated with dihydrogen. The purple circle denotes the resonance associated with $Ph_2C\mathit{H}CH_3$ of 1,1-diphenylethane. The green circle denotes the resonance associated with $Ph_2CHC\mathit{H}_3$ of 1,1-diphenylethane.

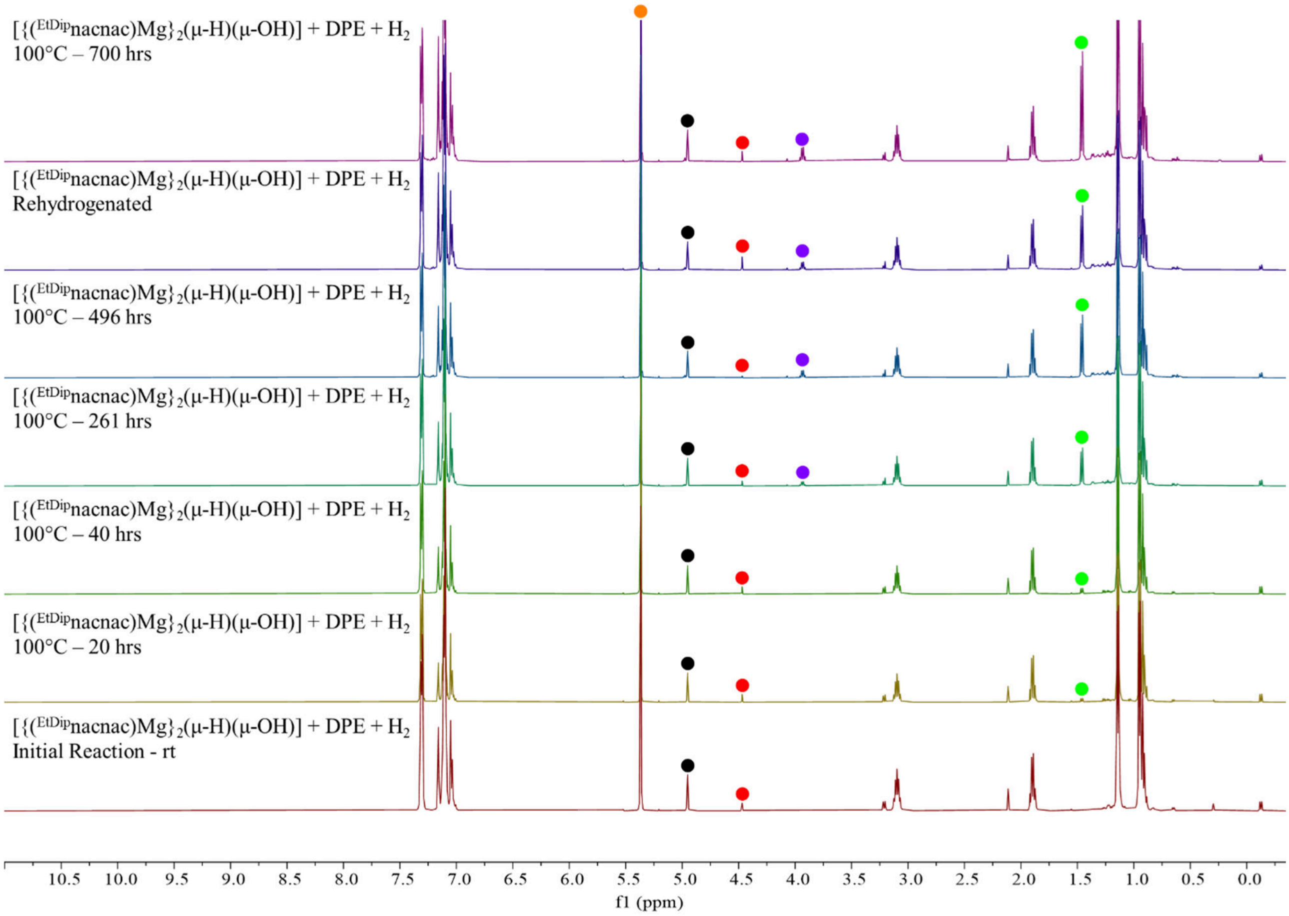

Figure S166. Stacked ^{1}H NMR spectra (499.9 MHz, C_6D_6, 298 K) of the reaction of a colourless *in-situ* generated solution of [{(^{EtDip}nacnac)Mg}$_2$(μ-H)(μ-OH)] **4b** (14.3 mg, 14.9 μmol, 15.2 mol%), DPE (17.4 μL, 98.5 μmol, 6.6 equiv.) and dihydrogen (ca. 1 bar). The orange circle denotes the resonance associated with $Ph_2CC\mathit{H}_2$ of DPE. The black circle denotes the resonance associated with the backbone-C*H* of [{(^{EtDip}nacnac)Mg}$_2$(μ-H)(μ-OH)] **4b**. The red circle denotes the resonance associated with dihydrogen. The purple circle denotes the resonance associated with $Ph_2C\mathit{H}CH_3$ of 1,1-diphenylethane. The green circle denotes the resonance associated with $Ph_2CHC\mathit{H}_3$ of 1,1-diphenylethane.

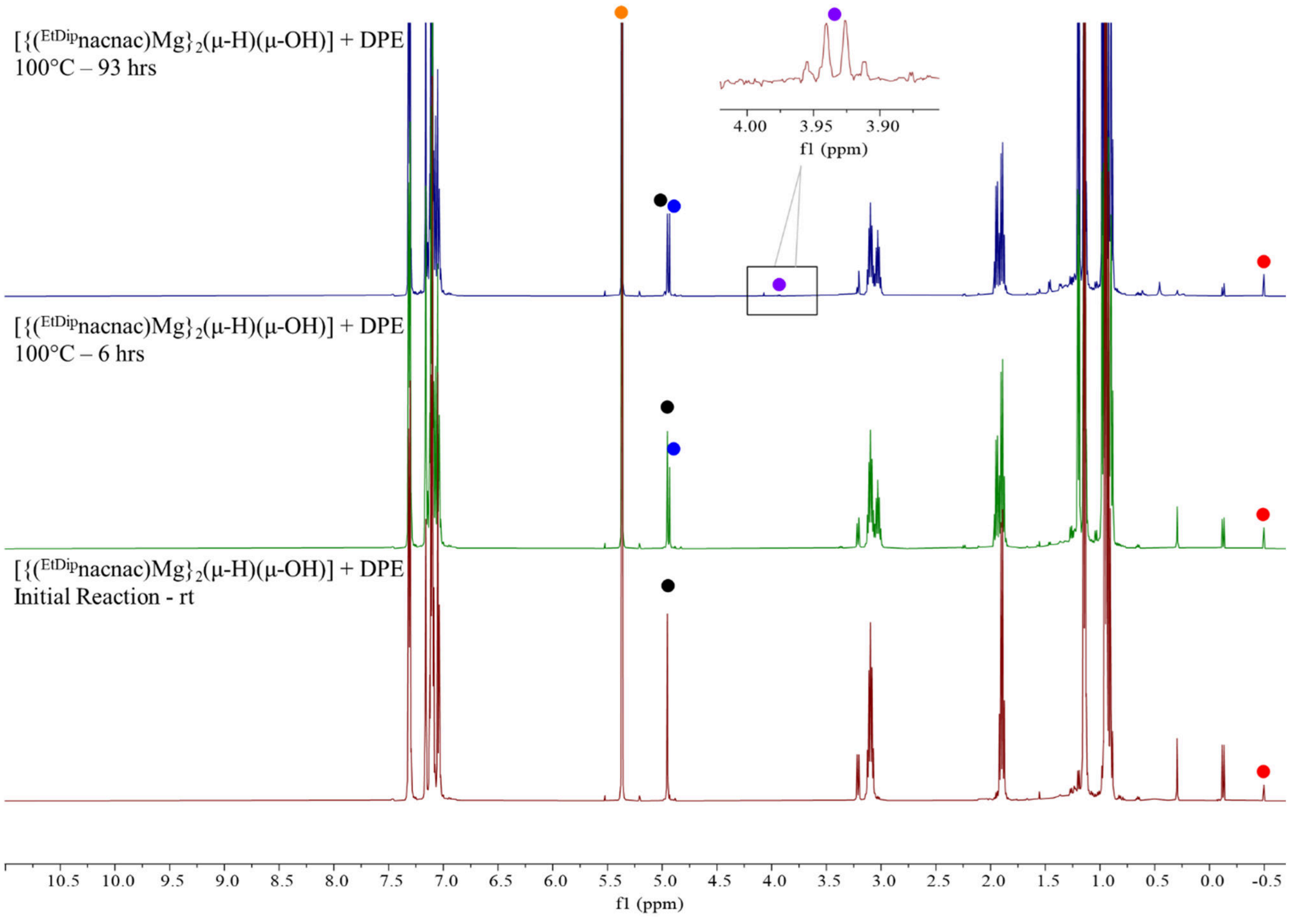

Figure S167. Stacked ^{1}H NMR spectra of the reaction of a colourless solution of [{(^{EtDip}nacnac)Mg}$_2$(μ-H)(μ-OH)] **4b** (10.1 mg, 10.5 μmol, 1.0 equiv.) and DPE (7.4 μL, 42 μmol, 4.0 equiv.) in C_6D_6 (0.5 mL) in a J Young NMR tube. The orange circle denotes the resonance associated with $Ph_2CC\mathit{H}_2$ of DPE. The black circle denotes the resonance associated with the backbone-C*H* of [{(^{EtDip}nacnac)Mg}$_2$(μ-H)(μ-OH)] **4b**. The blue circle denotes the resonance associated with the backbone-C*H* of [{(^{EtDip}nacnac)Mg}$_2$(μ-O)] **1b**. The purple circle denotes the resonance associated with $Ph_2C\mathit{H}CH_3$ of 1,1-diphenylethane. The red circle denotes the resonance associated with Mg-(O*H*)$_2$ of [{(^{EtDip}nacnac)Mg(μ-OH)}$_2$] **3b**.

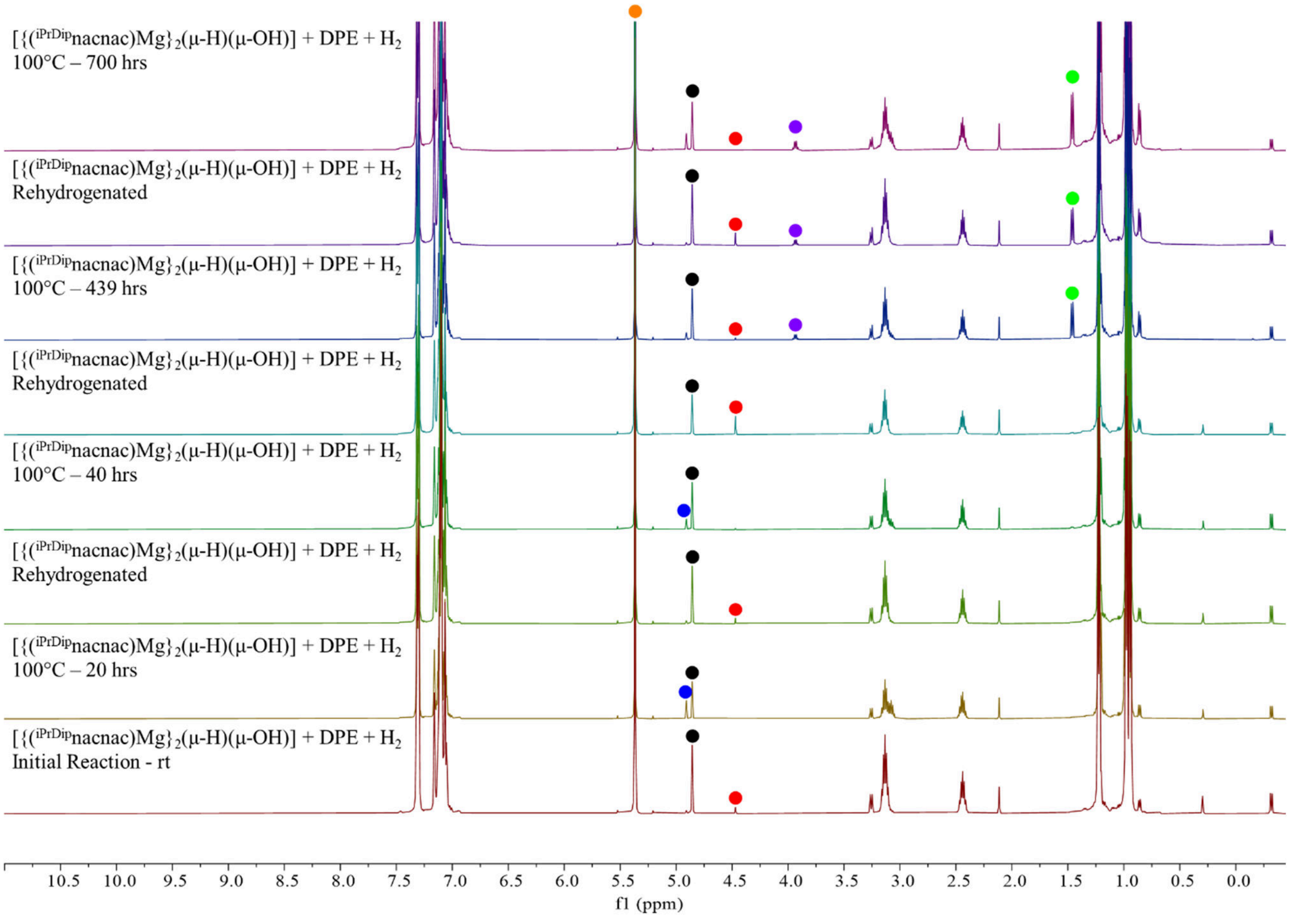

Figure S168. Stacked ^{1}H NMR spectra (499.9 MHz, C_6D_6, 298 K) of the reaction of a colourless *in-situ* generated solution of [{(^{iPrDip}nacnac)Mg}$_2$(μ-H)(μ-OH)] **4c** (15.1 mg, 14.9 μmol, 14.9 mol%), DPE (17.6 μL, 99.8 μmol, 6.71 equiv.) and dihydrogen (ca. 1 bar). The orange circle denotes the resonance associated with Ph_2CCH_2 of DPE. The black circle denotes the resonance associated with the backbone-C*H* of [{(^{iPrDip}nacnac)Mg}$_2$(μ-H)(μ-OH)] **4c**. The blue circle denotes the resonance associated with the backbone-C*H* of [{(^{iPrDip}nacnac)Mg}$_2$(μ-O)] **1c**. The red circle denotes the resonance associated with dihydrogen. The purple circle denotes the resonance associated with Ph_2CHCH_3 of 1,1-diphenylethane. The green circle denotes the resonance associated with Ph_2CHCH_3 of 1,1-diphenylethane.

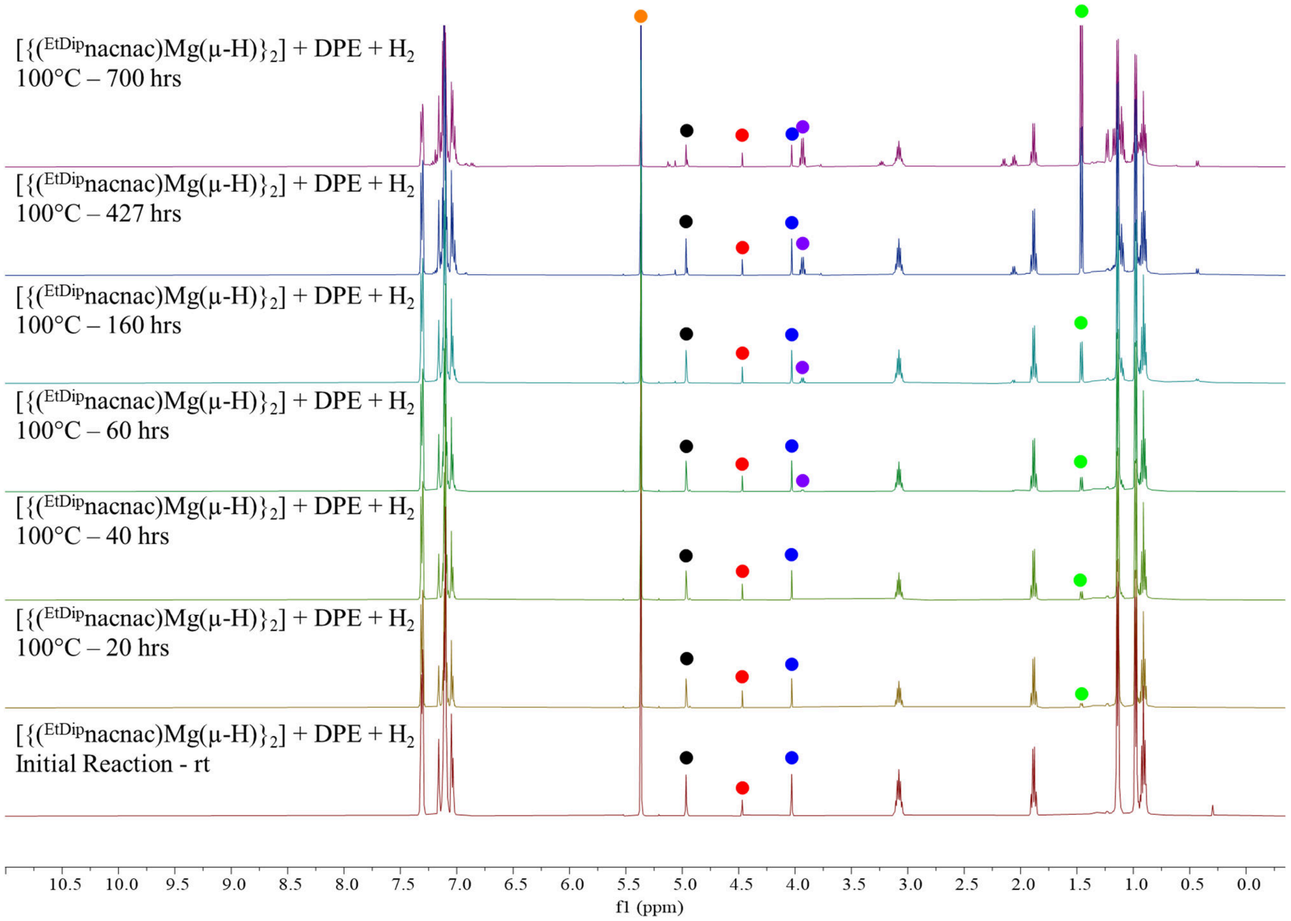

Figure S169. Stacked ^{1}H NMR spectra (499.9 MHz, C_6D_6, 298 K) of the reaction of a colourless *in-situ* generated solution of [{(^{EtDip}nacnac)Mg(μ-H)}$_2$] **7b** (10.0 mg, 10.6 μmol, 15.9 mol%), DPE (11.8 μL, 66.9 μmol, 6.30 equiv.) and dihydrogen (ca. 1 bar). The orange circle denotes the resonance associated with $Ph_2CC\mathit{H}_2$ of DPE. The black circle denotes the resonance associated with the backbone-C*H* of [{(^{EtDip}nacnac)Mg(μ-H)}$_2$] **7b**. The red circle denotes the resonance associated with dihydrogen. The blue circle denotes the resonance associated with Mg-*H* of [{(^{EtDip}nacnac)Mg(μ-H)}$_2$] **7b**. The purple circle denotes the resonance associated with $Ph_2C\mathit{H}CH_3$ of 1,1-diphenylethane. The green circle denotes the resonance associated with $Ph_2CHC\mathit{H}_3$ of 1,1-diphenylethane.

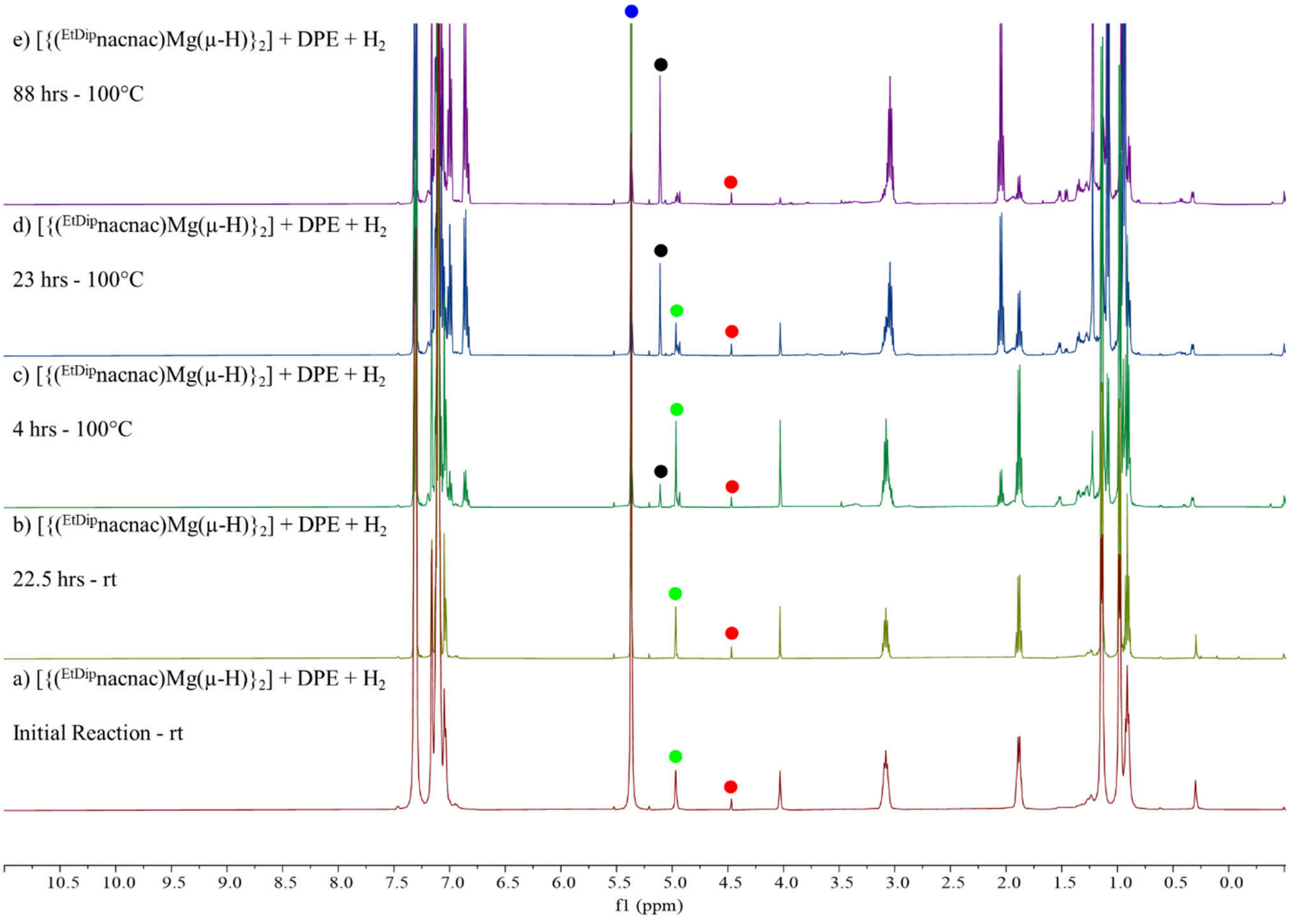

Figure S170. Stacked ^{1}H NMR spectra of the reaction of a solution of [{(^{EtDip}nacnac)Mg(μ-H)}$_2$] **7b** (12.5 mg, 13.3 μmol, 7.0 mol%) in C_6D_6 (0.6 mL) in a J Young NMR tube with DPE (33.6 μL, 190 μmol, 14.3 equiv.) and dihydrogen (ca.<1 bar). The red circle denotes the resonance associated with dihydrogen. The green circle denotes the resonance associated with the backbone-C*H* of [{(^{EtDip}nacnac)Mg(μ-H)}$_2$] **7b**. The black circle denotes the resonance associated with the backbone-C*H* of [(^{EtDip}nacnac)Mg{CPh_2CH_3}]. The blue circle denotes the resonance associated with C*H*$_2$CPh$_2$ of DPE. The Mg-*H* resonance of the starting material [{(^{EtDip}nacnac)Mg(μ-H)}$_2$] **7b** can be seen at δ 4.03 ppm.

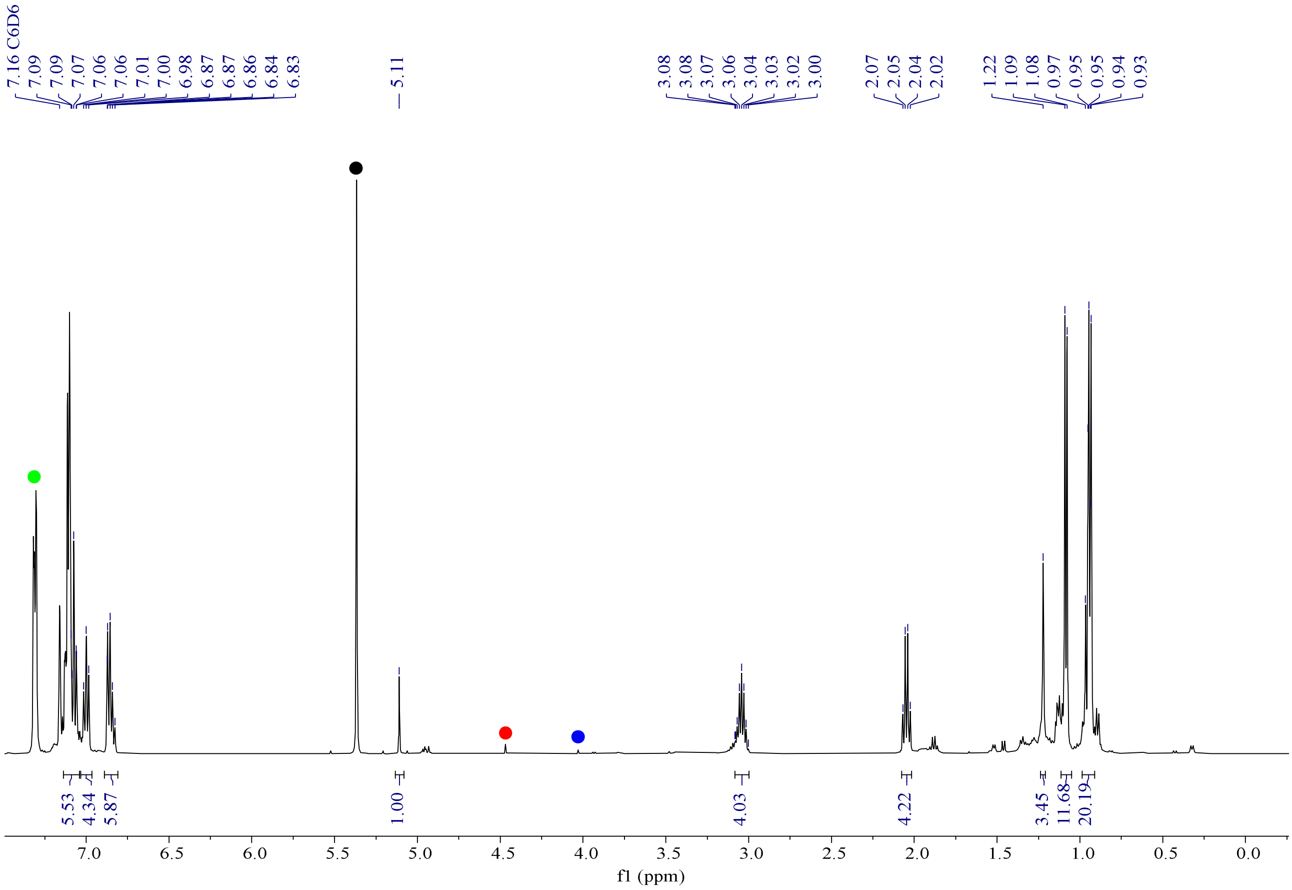

Figure S171. ^{1}H NMR spectrum (499.9 MHz, C_6D_6, 298 K) of *in-situ* generated [(^{EtDip}nacnac)Mg{CPh_2CH_3}]. The red circle denotes the resonance associated with dihydrogen. The blue circle denotes the resonance associated with Mg-*H* of [{(^{EtDip}nacnac)Mg(μ-H)}$_2$] **7b**. The black circle denotes the resonance associated with C*H*$_2$CPh$_2$ of DPE. The green circle denotes the resonance associated with Ph-*H* of DPE.

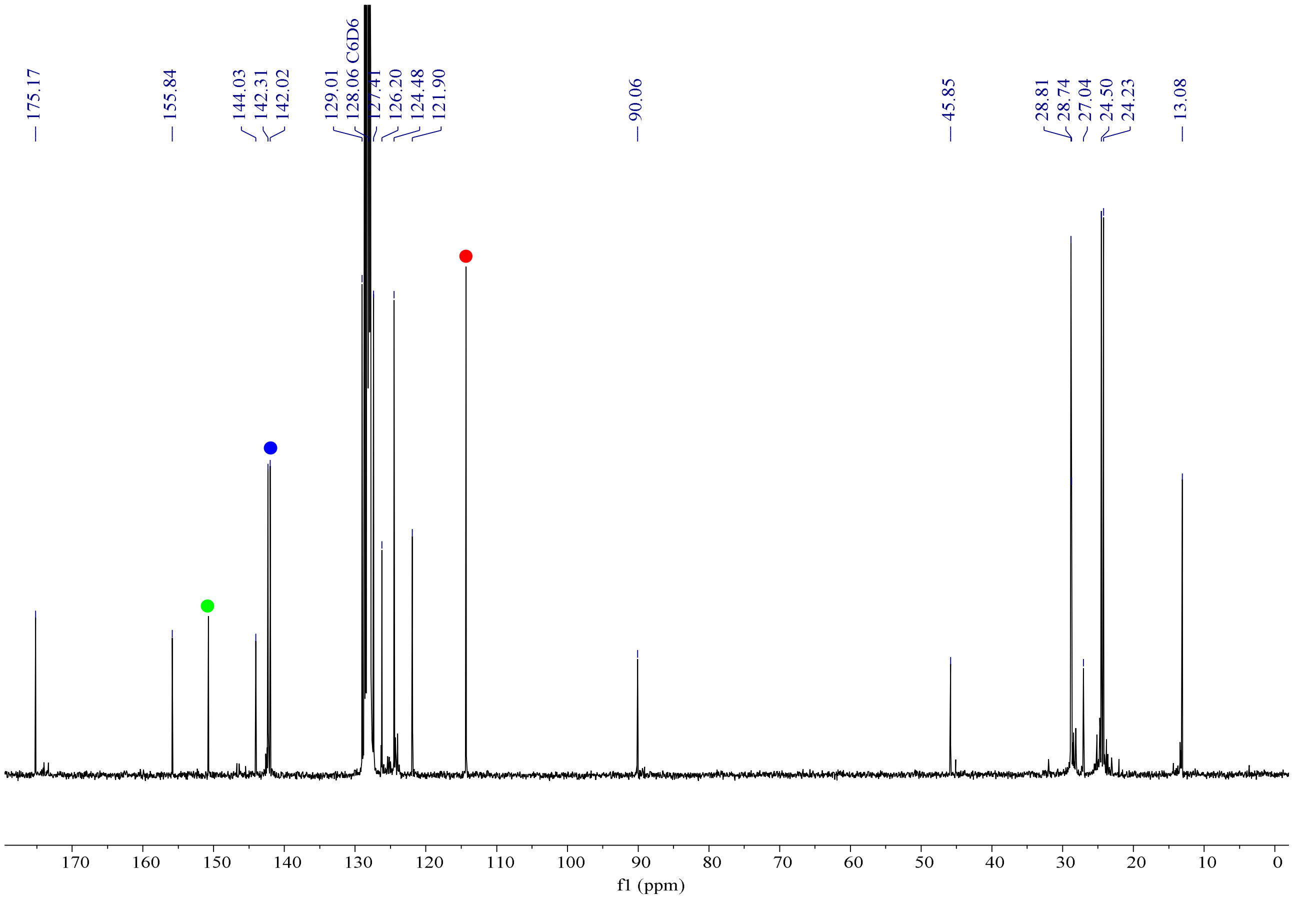

Figure S172. $^{13}C\{^1H\}$ NMR spectrum (125.7 MHz, C_6D_6, 298 K) of *in-situ* generated [(^{EtDip}nacnac)Mg{CPh_2CH_3}]. The red circle denotes the resonance associated with $\mathit{C}H_2CPh_2$ of DPE. The blue circle denotes the resonance associated with $CH_2\mathit{C}Ph_2$ of DPE. The green circle denotes the resonance associated with Ph-*C* of DPE.

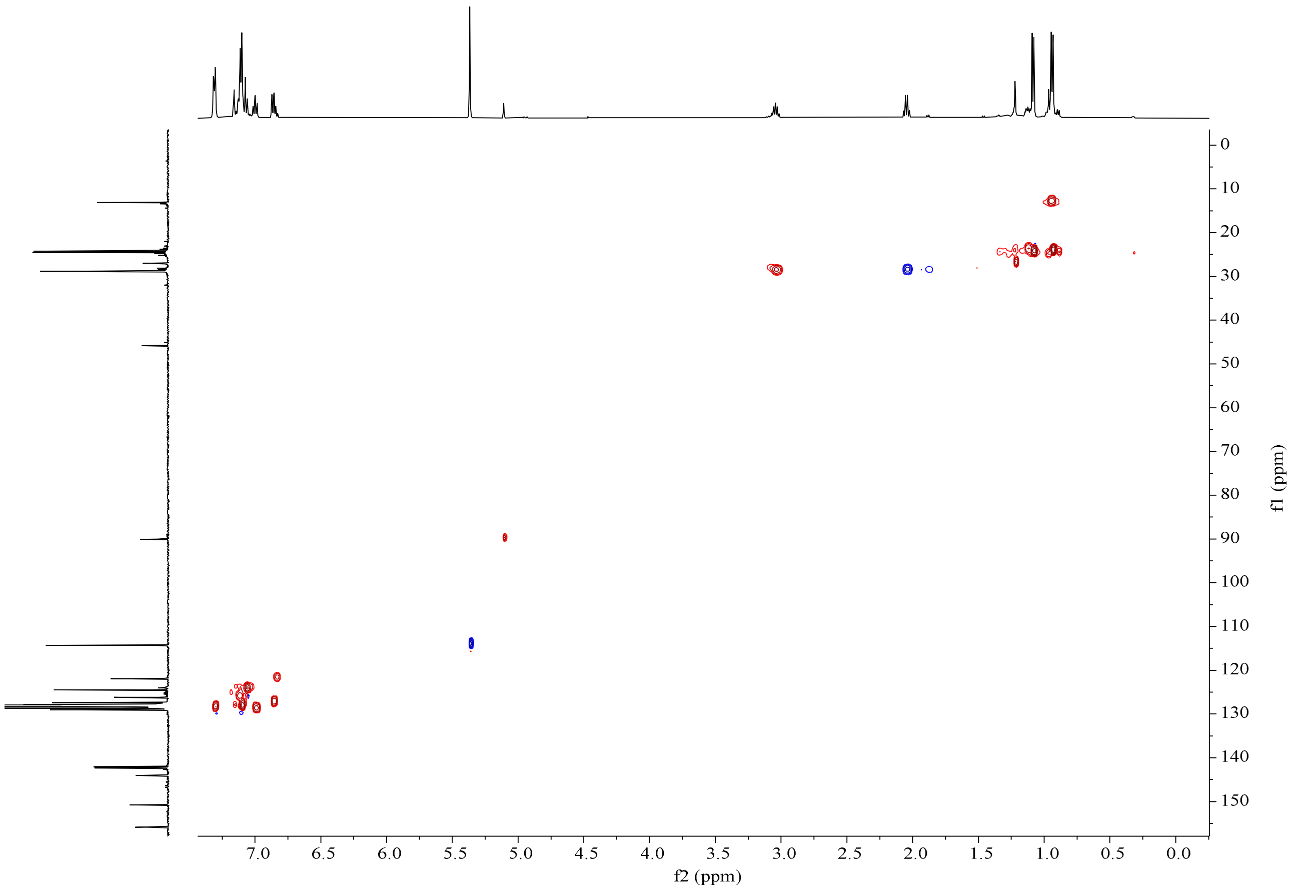

Figure S173. ^{1}H-^{13}C HSQC NMR spectrum of *in-situ* generated [(^{EtDip}nacnac)Mg{CPh_2CH_3}].

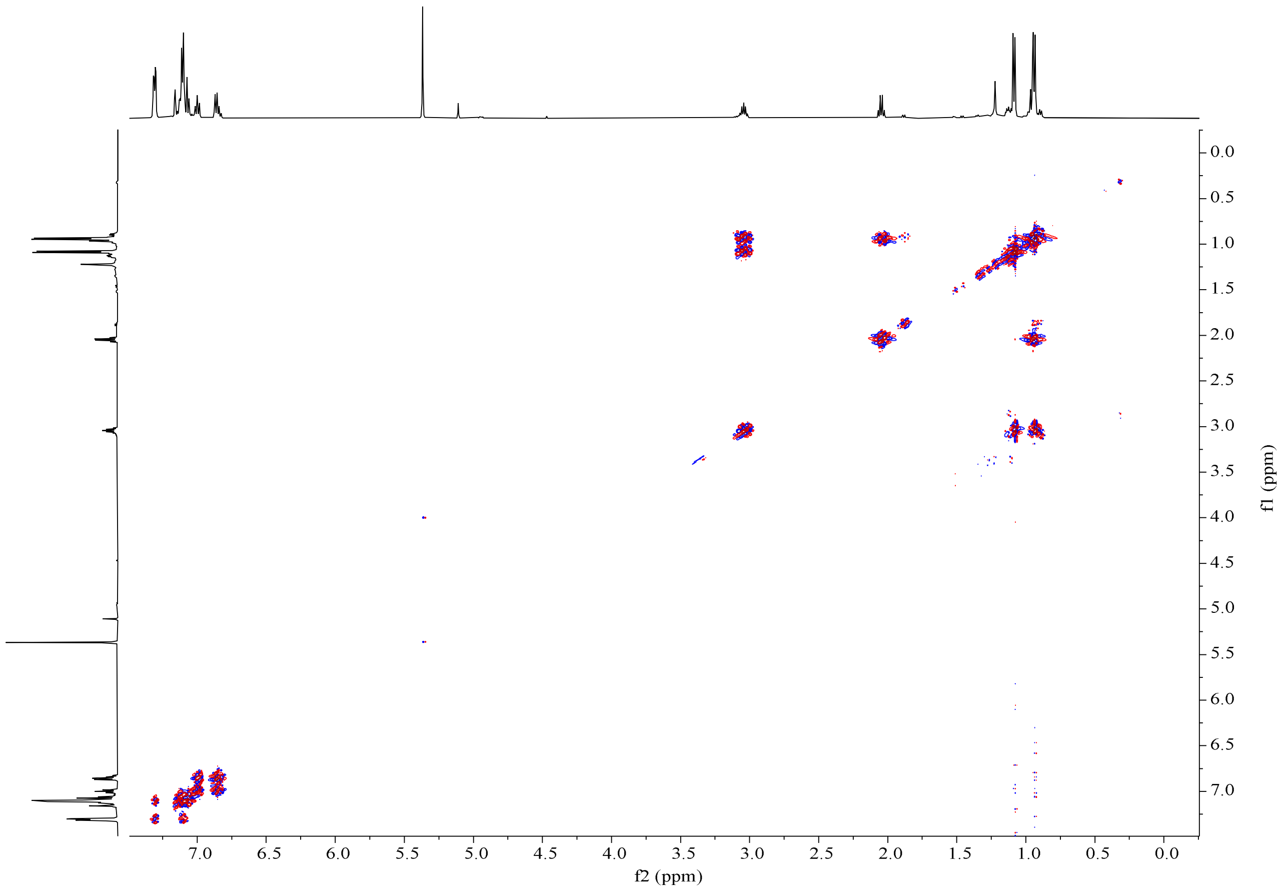

Figure S174. ^{1}H-^{1}H COSY NMR spectrum of *in-situ* generated [(^{EtDip}nacnac)Mg{CPh_2CH_3}].

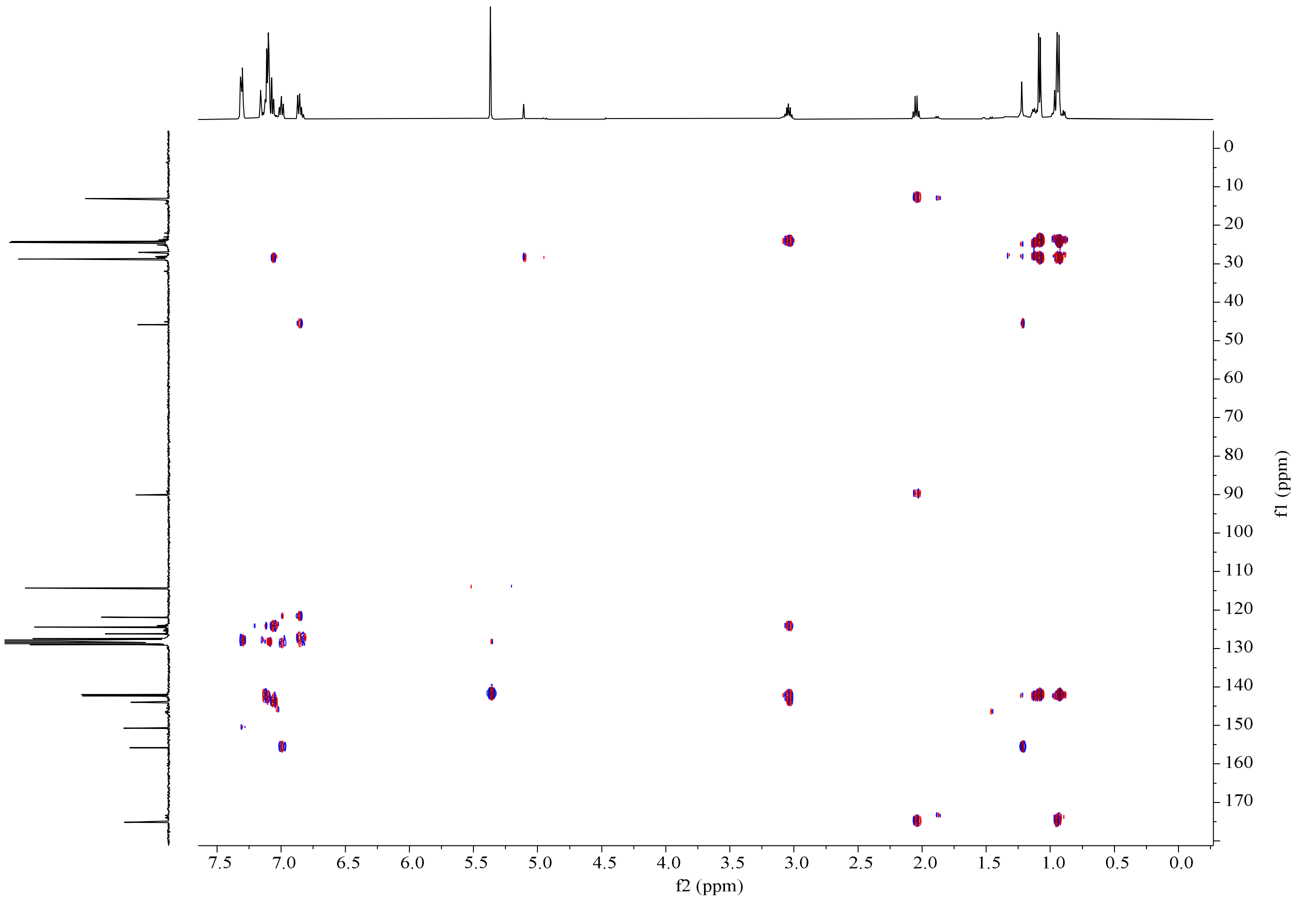

Figure S175. ^{1}H-^{13}C HMBC NMR spectrum of *in-situ* generated [(^{EtDip}nacnac)Mg{CPh_2CH_3}].

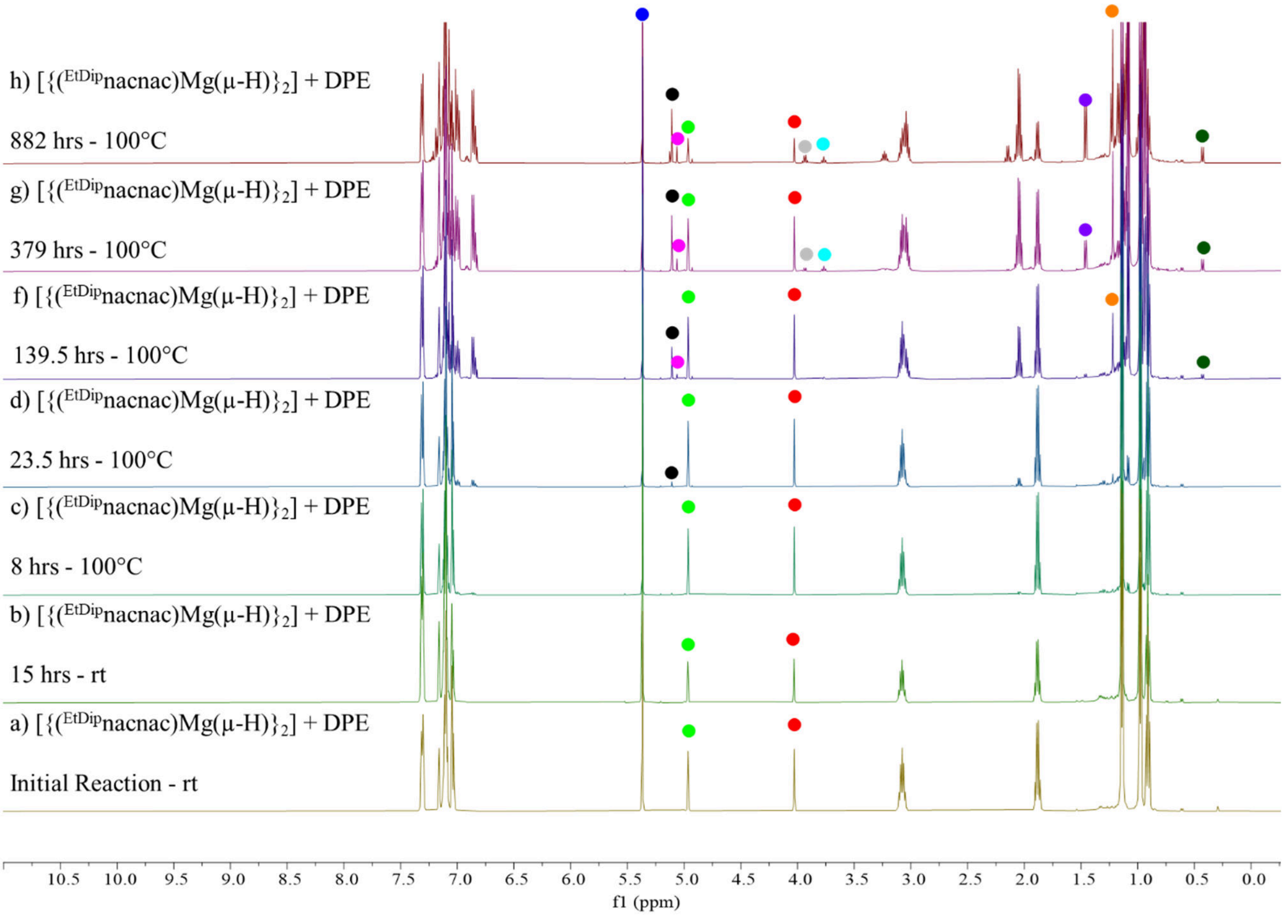

Figure S176. Stacked ^{1}H NMR spectra (499.9 MHz, C_6D_6, 298 K) of the reaction of a colourless solution of [{(^{EtDip}nacnac)Mg(μ-H)}$_2$] **7b** (17.8 mg, 18.9 μmol, 1.0 equiv.) and DPE (9.2 μL, 52 μmol, 2.8 equiv.) in a J Young NMR tube in C_6D_6 (0.6 mL) at 100°C for 882 hours. The green circle denotes the resonance associated with the backbone-C*H* of [{(^{EtDip}nacnac)Mg(μ-H)}$_2$] **7b**. The red circle denotes the resonance associated with Mg-*H* of [{(^{EtDip}nacnac)Mg(μ-H)}$_2$] **7b.** The black circle denotes the resonance associated with the backbone-C*H* of [(^{EtDip}nacnac)Mg{CPh_2CH_3}]. The orange circle denotes the resonance associated with Mg-CPh_2CH_3 of [(^{EtDip}nacnac)Mg{CPh_2CH_3}]. The pink circle denotes the resonance associated with the backbone-C*H* of [(^{EtDip}nacnac)Mg{CH_2CHPh_2}]. The light blue circle denotes the resonance associated with Mg-CH_2C*H*Ph_2 of [(^{EtDip}nacnac)Mg{CH_2CHPh_2}]. The dark green circle denotes the resonance associated with Mg-CH_2CHPh_2 of [(^{EtDip}nacnac)Mg{CH_2CHPh_2}]. The purple circle denotes the resonance associated with Ph_2CHCH_3 of 1,1-diphenylethane. The grey circle denotes the resonance associated with Ph_2C*H*CH_3 of 1,1-diphenylethane. The blue circle denotes the resonance associated with C$H_2$$CPh_2$ of DPE.

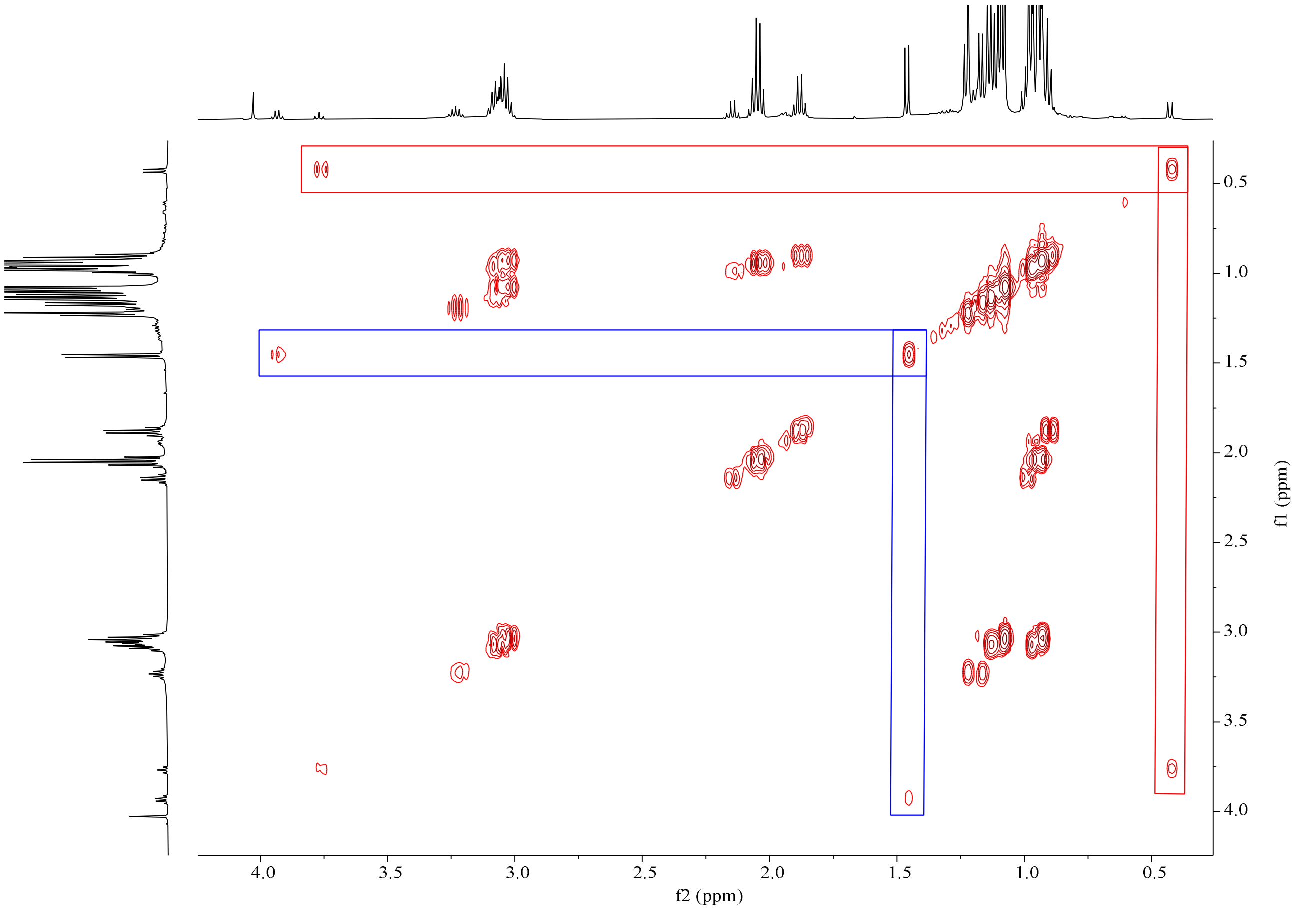

Figure S177. ^{1}H-^{1}H NMR COSY spectrum of the reaction of a colourless solution of [{(^{EtDip}nacnac)Mg(μ-H)}$_2$] **7b** (17.8 mg, 18.9 μmol, 1.0 equiv.) and DPE (9.2 μL, 52 μmol, 2.8 equiv.) in a J Young NMR tube in C_6D_6 (0.6 mL) at 100°C for 882 hours (chemical range: -0.5-4.0 ppm). The coupling between the Mg-CH$_2$C*H*Ph$_2$ and Mg-C*H*$_2$CHPh$_2$ resonances from [(^{EtDip}nacnac)Mg{CH$_2$CHPh$_2$}] is highlighted in red. The coupling between the C*H*$_3$ and CPh$_2$*H* resonances from 1,1-diphenylethane is highlighted in blue.

3 X-ray Crystallography

X-ray diffraction data for compounds **3c** and **2b''** were collected at 173 K using a Rigaku MM-007HF High Brilliance RA generator/confocal optics with XtaLAB P100 diffractometer [Cu Kα radiation (λ = 1.54187 Å)]. Diffraction data for all other compounds were collected at temperatures between 93 and 173 K using a Rigaku FR-X Ultrahigh Brilliance Microfocus RA generator/confocal optics with XtaLAB P200 diffractometer [Mo Kα radiation (λ = 0.71073 Å)]. Intensity data for all compounds were collected using either CrystalClear[9] (using ω steps and accumulating area detector images spanning at least a hemisphere of reciprocal space) or CrysAlisPro[10] (using a calculated strategy), and processed (including correction for Lorentz, polarization and absorption) using CrysAlisPro.[10] Structures were solved by dual space (SHELXT[11]) or direct (SIR2011[12]) methods and refined by full-matrix least-squares against F^2 (SHELXL-2019/3[13]). Non-hydrogen atoms were refined anisotropically, and hydrogen atoms were refined using a riding model except for the hydrogen atoms on magnesium hydroxyl groups and/or bridging magnesium hydrides of **3b**, **3c**, **4a**, **4b**, **4c**, **7b'**, **7b''**, **8b**, and **12b**, which were located from the difference Fourier map and refined isotropically subject to distance restraints. Crystals of **2b'** were affected by non-merohedral twinning, with a refined twin fraction of 0.3962(13). Crystals of $[(^{EtDip}nacnac)MgI(OEt_2)]\cdot 0.5\ C_6H_6$ were affected by non-merohedral twinning, identified using PLATON,[14] which refined to a twin fraction of 0.4240(9). Calculations were performed using the CrystalStructure[15] or Olex2,[16] interfaces. Selected crystallographic data is summarised in Table S1. Specific details on individual structures or on their refinements are described in the following section with images of the molecular structures. Please note that the order of molecular structures is presented in relation to the order the compound synthesis is described in section 1. Table S2 provides an overview of the distortion in the overall structures of the presented dimagnesium complexes. CCDC 2401221-2401239 contains the supplementary crystallographic data for this paper. These data can be obtained free of charge from The Cambridge Crystallographic Data Centre via www.ccdc.cam.ac.uk/structures.

Table S1. Crystallographic data.

Compound reference	[(^{EtDip}nacnac)MgI(OEt$_2$)]·0.5 C_6H_6	[{(^{EtDip}nacnac)Mg(μ-I)}$_2$]	**2b'**	**2b''**
Chemical formula	$C_{38}H_{58}IMgN_2O$	$C_{62}H_{90}I_2Mg_2N_4$	$C_{62}H_{90}Mg_2N_4$	$C_{62}H_{90}Mg_2N_4$
Formula weight	710.07	1193.79	939.99	939.99
Temperature/K	125	173	93	173
Radiation type	Mo	Mo	Mo	Cu
Wavelength/Å	0.71073	0.71073	0.71073	1.54184
Crystal system	monoclinic	monoclinic	monoclinic	tetragonal
Space group	$P2_1/c$	$P2_1/n$	$C2/c$	$P4_12_12$
a/Å	22.6600(6)	9.94707(14)	15.8210(5)	11.41442(18)
b/Å	18.1130(4)	24.1456(4)	16.3120(4)	11.41442(18)
c/Å	18.7246(4)	13.0268(2)	22.4910(5)	44.5289(10)
α/°	90	90	90	90
β/°	90.121(2)	102.3476(15)	93.455(2)	90
γ/°	90	90	90	90
Unit cell volume/Å^3	7685.3(3)	3056.37(8)	5793.8(3)	5801.6(2)
No. of formula units per unit cell, Z	8	2	4	4
Density (calc)/ Mg/m^3	1.227	1.297	1.078	1.076
Absorption coefficient, μ/mm^{-1}	0.879	1.089	0.081	0.658
F(000)	2984	1240	2056	2056
Theta range/°	1.803 to 29.187	1.687 to 29.436	1.795 to 28.441	3.971 to 70.986
Reflections collected	138763	65770	92954	30612
Independent reflections	18268	7580	9368	5216
R_{int}	0.0811	0.0237	0.0982	0.0602
Completeness (to theta full)/%	99.9	100	99.9	98.7
Restraints / parameter	70 / 855	0 / 326	0 / 318	0 / 317
Goodness of fit on F^2	1.021	1.039	1.135	1.068
Final R_1 values ($I > 2\sigma(I)$)	0.0529	0.0232	0.0498	0.0346
Final $wR(F^2)$ values ($I > 2\sigma(I)$)	0.1203	0.0602	0.1590	0.0847
Final R_1 values (all data)	0.0775	0.0268	0.0555	0.0443
Final $wR(F^2)$ values (all data)	0.1301	0.0617	0.1642	0.0976
Largest diff. peak and hole/e·Å^{-3}	2.928 and -1.354	0.403 and -0.593	0.393 and -0.192	0.165 and -0.171
Flack x parameter	-	-	-	0.02(3)
CCDC number	2401223	2401235	2401234	2401227

Table S1 (continued). Crystallographic data.

Compound reference	**2c**	**9b**	[{(^{EtDip}nacnac)Mg(μ-O*n*Bu)}$_2$]·4 C_6H_6	**7b'**
Chemical formula	$C_{66}H_{98}Mg_2N_4$	$C_{35}H_{54}MgN_2$	$C_{94}H_{132}Mg_2N_4O_2$	$C_{62}H_{92}Mg_2N_4$
Formula weight	996.10	527.11	1398.65	942.01
Temperature/K	100	173	100	100
Radiation type	Mo	Mo	Mo	Mo
Wavelength/Å	0.71073	0.71073	0.71073	0.71073
Crystal system	orthorhombic	monoclinic	monoclinic	monoclinic
Space group	$C222_1$	$P2_1/n$	$P2_1/n$	$C2$/c
a/Å	16.46267(16)	11.2423(2)	14.0321(3)	15.7289(4)
b/Å	16.19933(15)	21.2645(3)	18.4762(3)	16.3091(4)
c/Å	46.6890(4)	14.2292(2)	17.3844(4)	22.5201(5)
α/°	90	90	90	90
β/°	90	92.2297(16)	109.786(2)	92.591(2)
γ/°	90	90	90	90
Unit cell volume/Å^3	12451.2(2)	3399.10(10)	4241.01(16)	5771.0(2)
No. of formula units per unit cell, Z	8	4	2	4
Density (calc)/ Mg/m^3	1.063	1.030	1.095	1.084
Absorption coefficient, μ/mm^{-1}	0.079	0.075	0.077	0.082
F(000)	4368	1160	1528	2064
Theta range/°	2.196 to 28.506	1.723 to 29.563	1.663 to 29.127	2.041 to 29.132
Reflections collected	138414	73054	91831	60887
Independent reflections	14551	8516	10382	7004
R_{int}	0.0425	0.0225	0.0339	0.0775
Completeness (to theta full)/%	99.9	100	100	99.9
Restraints / parameter	2 / 694	55 / 453	72 / 526	0 / 321
Goodness of fit on F^2	1.057	1.030	1.083	1.019
Final R_1 values ($I > 2\sigma(I)$)	0.0322	0.0551	0.0487	0.0468
Final $wR(F^2)$ values ($I > 2\sigma(I)$)	0.0754	0.1542	0.1202	0.1052
Final R_1 values (all data)	0.0350	0.0790	0.0620	0.0682
Final $wR(F^2)$ values (all data)	0.0764	0.1723	0.1253	0.1136
Largest diff. peak and hole/e·Å^{-3}	0.218 and -0.185	0.263 and -0.139	0.331 and -0.257	0.281 and -0.250
Flack x parameter	0.01(3)	-	-	-
CCDC number	2401229	2401226	2401231	2401237

Table S1 (continued). Crystallographic data.

Compound reference	**7b''**	**12b**	**1c**	**4a**
Chemical formula	$C_{62}H_{92}Mg_2N_4O_{0.20}$	$C_{38}H_{56}MgN_4$	$C_{66}H_{98}Mg_2N_4O$	$C_{58}H_{84}Mg_2N_4O$
Formula weight	945.21	593.17	1012.10	901.91
Temperature/K	150	100	100	100
Radiation type	Mo	Mo	Mo	Mo
Wavelength/Å	0.71073	0.71073	0.71073	0.71073
Crystal system	tetragonal	triclinic	tetragonal	monoclinic
Space group	$P4_12_12$	$P\bar{1}$	$I4_1cd$	$C2$/c
a/Å	11.38485(9)	9.3950(3)	16.8521(2)	22.8021(3)
b/Å	11.38485(9)	12.1352(4)	16.8521(2)	14.7342(2)
c/Å	44.6023(6)	16.3551(5)	44.0285(8)	16.0484(2)
α/°	90	80.122(3)	90	90
β/°	90	84.094(2)	90	90.6003(12)
γ/°	90	77.649(3)	90	90
Unit cell volume/Å^3	5781.12(12)	1790.24(9)	12503.8(4)	5391.48(13)
No. of formula units per unit cell, Z	4	2	8	4
Density (calc)/ Mg/m^3	1.086	1.100	1.075	1.111
Absorption coefficient, μ/mm^{-1}	0.082	0.080	0.081	0.086
F(000)	2070	648	4432	1968
Theta range/°	1.826 to 29.173	1.987 to 29.063	1.850 to 29.172	2.072 to 29.038
Reflections collected	127275	39061	130183	54026
Independent reflections	7336	8270	7738	6474
R_{int}	0.0502	0.0484	0.0634	0.0318
Completeness (to theta full)/%	100	99.9	100	99.9
Restraints / parameter	7 / 328	0 / 404	1 / 342	2 / 332
Goodness of fit on F^2	1.076	1.032	1.081	1.045
Final R_1 values ($I > 2\sigma(I)$)	0.0407	0.0586	0.0461	0.0448
Final $wR(F^2)$ values ($I > 2\sigma(I)$)	0.0932	0.1085	0.0868	0.1173
Final R_1 values (all data)	0.0500	0.1039	0.0607	0.0514
Final $wR(F^2)$ values (all data)	0.0959	0.1206	0.0901	0.1214
Largest diff. peak and hole/e·Å^{-3}	0.195 and -0.156	0.276 and -0.252	0.258 and -0.257	1.310 and -0.254
Flack x parameter	0.01(5)	-	0.05(6)	-
CCDC number	2401228	2401238	2401230	2401224

Table S1 (continued). Crystallographic data.

Compound reference	**4b**	**4c**	**5b**·C_6H_6	**6c**
Chemical formula	$C_{62}H_{92}Mg_2N_4O$	$C_{66}H_{100}Mg_2N_4O$	$C_{76}H_{112}Mg_2N_4O_3$	$C_{80}H_{118}Mg_2N_8O$
Formula weight	958.01	1014.11	1178.31	1256.48
Temperature/K	100	173	100	93
Radiation type	Mo	Mo	Mo	Mo
Wavelength/Å	0.71073	0.71073	0.71073	0.71073
Crystal system	monoclinic	orthorhombic	Triclinic	monoclinic
Space group	$C2/c$	$C222_1$	$P\overline{1}$	$P2_1/n$
a/Å	15.8209(8)	16.59496(17)	11.7858(3)	13.8688(4)
b/Å	16.3432(8)	16.27848(17)	12.6925(3)	14.4601(5)
c/Å	22.5086(11)	46.8499(5)	14.2313(4)	18.9128(5)
α/°	90	90	99.319(2)	90
β/°	93.314(5)	90	110.799(3)	90.640(2)
γ/°	90	90	111.948(3)	90
Unit cell volume/Å^3	5810.2(5)	12656.1(2)	1736.06(9)	3792.6(2)
No. of formula units per unit cell, Z	4	8	1	2
Density (calc)/ Mg/m^3	1.095	1.064	1.127	1.100
Absorption coefficient, μ/mm^{-1}	0.083	0.080	0.084	0.080
F(000)	2096	4448	644	1372
Theta range/°	1.812 to 29.078	1.739 to 29.564	1.999 to 29.108	2.154 to 26.372
Reflections collected	32552	139933	36621	42303
Independent reflections	6787	15683	8041	7741
R_{int}	0.0869	0.0313	0.0423	0.1281
Completeness (to theta full)/%	99.9	100	99.7	99.9
Restraints / parameter	1 / 331	14 / 703	36 / 395	0 / 426
Goodness of fit on F^2	1.008	1.039	1.024	1.036
Final R_1 values ($I > 2\sigma(I)$)	0.0603	0.0396	0.0415	0.0578
Final $wR(F^2)$ values ($I > 2\sigma(I)$)	0.1163	0.0912	0.0898	0.1439
Final R_1 values (all data)	0.1460	0.0452	0.0658	0.0684
Final $wR(F^2)$ values (all data)	0.1383	0.0931	0.081	0.1499
Largest diff. peak and hole/e·Å^{-3}	0.584 and -0.248	0.311 and -0.213	0.281 and -0.281	0.43 and -0.33
Flack x parameter	-	0.00(2)	-	-
CCDC number	2401222	2401239	2401236	2401225

Table S1 (continued). Crystallographic data.

Compound reference	**3b**	**3c**·C_6H_6	**8b**
Chemical formula	$C_{62}H_{92}Mg_2N_4O_2$	$C_{84}H_{118}Mg_2N_4O_2$	$C_{76}H_{112}Mg_2N_8O_2$
Formula weight	974.01	1264.49	1218.35
Temperature/K	173	173	173
Radiation type	Mo	Cu	Mo
Wavelength/Å	0.71073	1.54184	0.71073
Crystal system	monoclinic	triclinic	monoclinic
Space group	$C2/c$	$P\overline{1}$	$P2_1/n$
a/Å	15.7771(9)	10.6273(2)	15.1364(4)
b/Å	16.4363(9)	13.6427(2)	14.3619(4)
c/Å	22.7751(12)	14.5100(2)	16.3960(4)
α/°	90	91.3160(10)	90
β/°	93.091(5)	100.0350(10)	91.736(2)
γ/°	90	111.143(2)	90
Unit cell volume/Å^3	5897.4(5)	1923.65(6)	3562.66(15)
No. of formula units per unit cell, Z	4	1	2
Density (calc)/ Mg/m^3	1.097	1.091	1.136
Absorption coefficient, μ/mm^{-1}	0.084	0.632	0.084
F(000)	2128	690	1328
Theta range/°	1.791 to 29.378	3.107 to 68.284	1.804 to 29.084
Reflections collected	33433	20648	72225
Independent reflections	7100	6863	8606
R_{int}	0.0664	0.0378	0.0564
Completeness (to theta full)/%	100	97.9	100
Restraints / parameter	2 / 331	15 / 510	1 / 413
Goodness of fit on F^2	1.015	1.064	1.030
Final R_1 values ($I > 2\sigma(I)$)	0.0639	0.0428	0.0451
Final $wR(F^2)$ values ($I > 2\sigma(I)$)	0.1256	0.1163	0.1009
Final R_1 values (all data)	0.1352	0.0507	0.0761
Final $wR(F^2)$ values (all data)	0.1437	0.1213	0.1114
Largest diff. peak and hole/e·Å^{-3}	0.331 and -0.222	0.28 and -0.24	0.426 and -0.241
Flack x parameter	-	-	-
CCDC number	2401232	2401221	2401233

Table S2. "Twisting" and "bending" in selected [(nacnac)Mg"X_2"Mg(nacnac)] complexes as determined from X-ray diffraction and selected DFT studies.

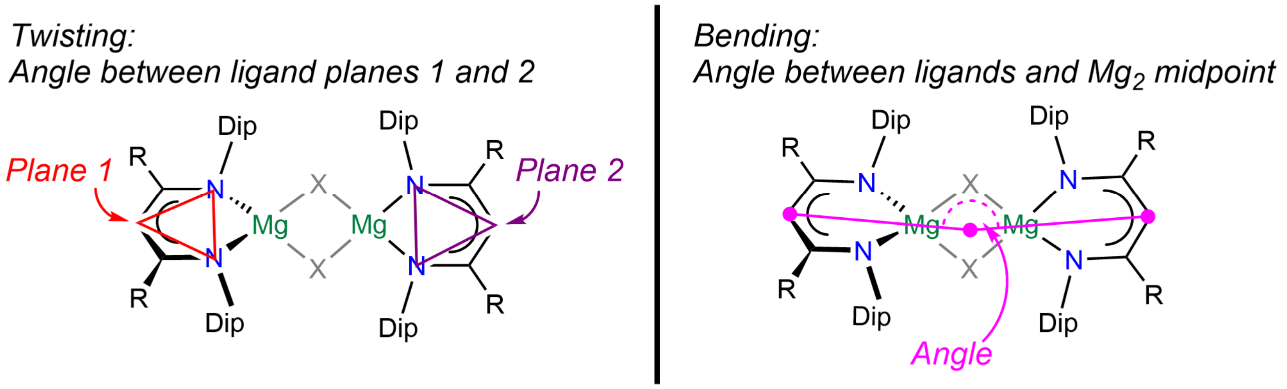

R, Ar<br>in ^{RAr}nacnac species	X_2 (**compound number**)	Twisting:<br>N···C(H)···N planes angle/°	Bending:<br>C(H)···Mg_2-midpoint···C(H) angle /°
Et, Dip	I, I	co-planar	180°
Et, Dip	O*n*Bu, O*n*Bu	co-planar	180°
*i*Pr, Dip	O (**1c**)	39.3	177.1°
Et, Dip	- (**2b'**)	82.8°	165.2
Et, Dip	- (**2b''**)	85.2°	163.8
*i*Pr, Dip	- (**2c**)	85.6, 79.5[b]	161.5, 160.6[b]
Et, Dip	H, H (**7b'**)	80.8	164.8°
Et, Dip	H, H (**7b''**)	83.5	163.3°
Me, Dip	H, OH (**4a**)	co-planar	180°
Et, Dip	H, OH (**4b**)	81.6	164.8
*i*Pr, Dip	H, OH (**4c**)	88.0, 89.6[b]	161.3, 160.9[b]
*i*Pr, Dip (DFT)[a]<br>(optimised from co-planar)	H, OH (**4c**)	20.3	172.03°
*i*Pr, Dip (DFT)[a]<br>(optimised from orthogonal)	H, OH (**4c**)	86.3	166.1°
TS (*i*Pr, Dip) (DFT)[a]	O···H···H (**TS** of **4c**)	38.0	175.2
Et, Dip	OH, OH (**3b**)	79.1	166.0
*i*Pr, Dip	OH, OH (**3c**)	co-planar	180°
From the literature:			
Me, Dip[7]	H, H	co-planar	180°
tBu, Dip[3]	H, H	73.3	161.0

[a]From DFT studies (vide infra) [b]Two values from two independent molecules

Table S2 collects information on the relative orientations of the β-diketiminate ligands in a range of structurally characterised complexes. Here, "twisting", e.g. to broadly distinguish between co-planar (0°) versus orthogonal (90°) arrangements of the (N···C(H)···N planes angle) ligand planes, and "bending" between ligand arrangements (C(H)···Mg_2-midpoint···C(H) angle) is provided as a measure of overall geometry and distortion. Not all distortions are, however, expressed by these two values.

[(^{EtDip}nacnac)MgI(OEt$_2$)]

The compound crystallised with two full molecules and one molecule of benzene in the asymmetric unit. Geometry restraints were applied in the refinement of the coordinated ether unit in molecule 2 and the benzene molecule.

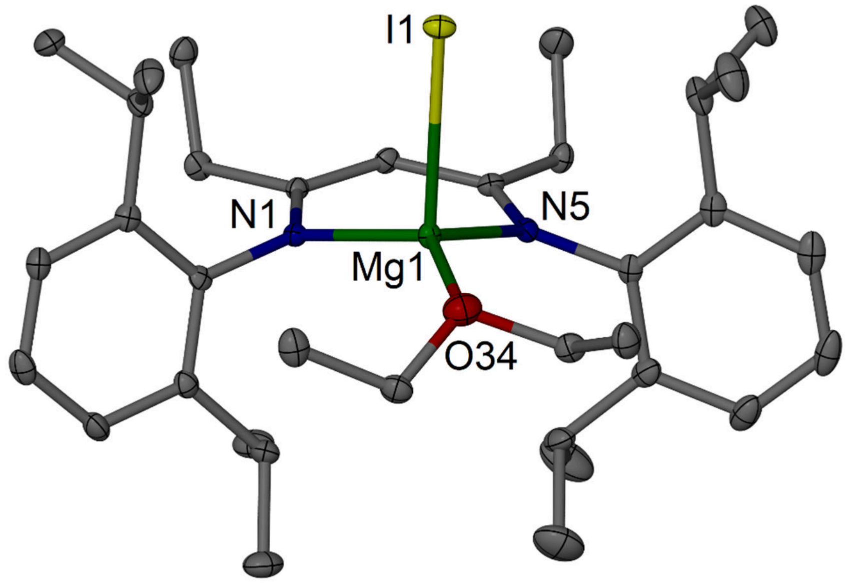

Figure S178. Molecular structure of one independent molecule of [(^{EtDip}nacnac)MgI(OEt$_2$)]·0.5 C_6H_6 (30% thermal ellipsoids). Hydrogen atoms and solvent are omitted for clarity. Selected bond lengths (Å) and angles (°): Molecule 1: I1-Mg1 2.9186(14), Mg1-O34 2.035(4), Mg1-N1 2.047(4), Mg1-N5 2.045(4); N5-Mg1-N1 97.05(15), O34-Mg1-I1 96.74(12). Molecule 2: I4-Mg4 2.9375(14), Mg4-O74 2.040(4), Mg4-N41 2.047(5), Mg4-N45 2.051(5); N41-Mg4-N45 95.30(14), O74-Mg4-I4 96.94(12).

[{(^{EtDip}nacnac)Mg(μ-I)}$_2$]

The compound crystallised with half a molecule in the asymmetric unit.

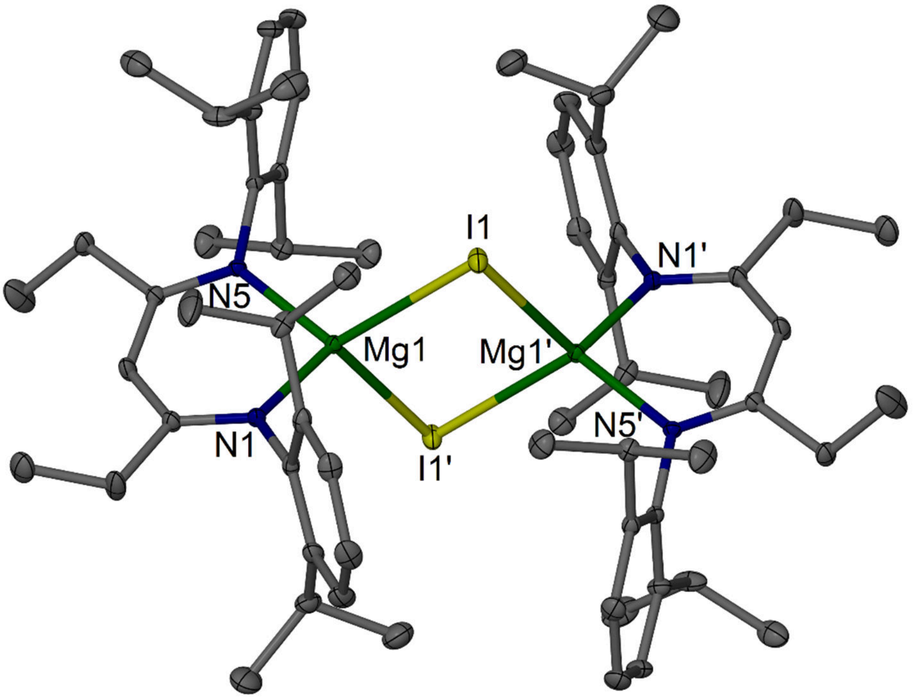

Figure S179. Molecular structure of [{(^{EtDip}nacnac)Mg(μ-I)}$_2$] (30% thermal ellipsoids). Hydrogen atoms are omitted for clarity. Selected bond lengths (Å) and angles (°): I1-Mg1 2.7771(5), I1-Mg1’ 2.7848(5), Mg1-N1 2.0265(13), Mg1-N5 2.0266(13); I1-Mg1-I1’ 92.969(14), Mg1-I1-Mg1’ 87.033(14), N1-Mg1-N5 95.89(5).

[{(^{EtDip}nacnac)Mg}$_2$] 2b: 2b' and 2b''

Two independent molecular structures were obtained. In both cases, compound **2b** crystallised with half a molecule in the asymmetric unit.

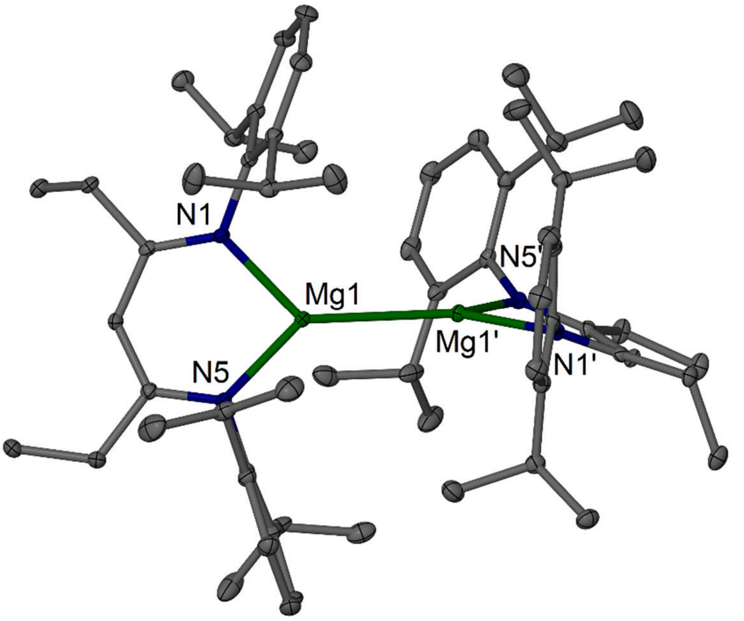

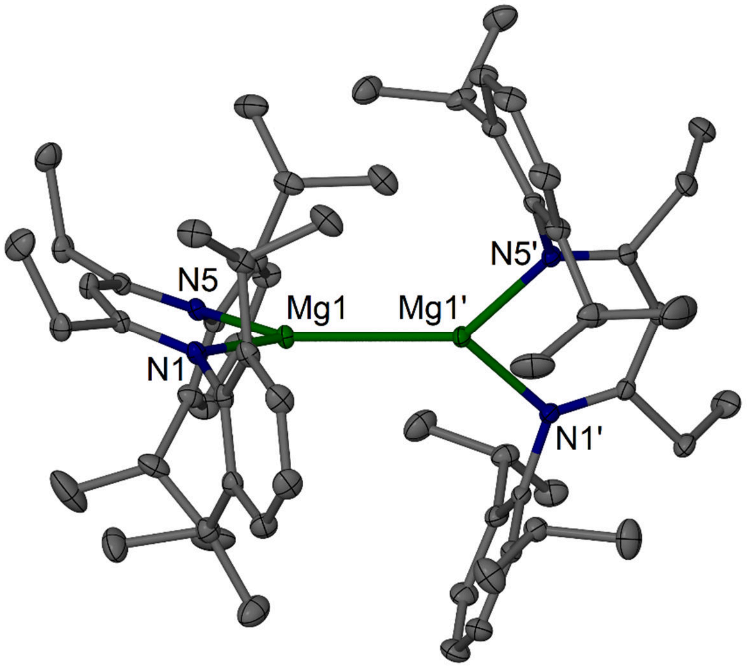

Figure S180. Molecular structure of [{(^{EtDip}nacnac)Mg}$_2$] **2b** (30% thermal ellipsoids). Hydrogen atoms are omitted for clarity. Selected bond lengths (Å) and angles (°): Top structure (**2b'**): Mg1-Mg1' 2.8755(16), Mg1-N1 2.070(2), Mg1-N5 2.068(2); N5-Mg1-N1 91.09(8). Bottom structure (**2b''**): Mg1-Mg1' 2.8554(14), Mg1-N1 2.066(2), Mg1-N5 2.069(2); N1-Mg1-N5 91.35(8).

[{(^{iPrDip}nacnac)Mg}$_2$] 2c

This compound has been reported previously[4] but has not been structurally characterised. The compound crystallised with two very similar independent half molecules in the asymmetric unit. The second molecule shows disorder in one isopropyl group that was modelled with two positions for the atoms in each methyl group and refined using geometry restraints.

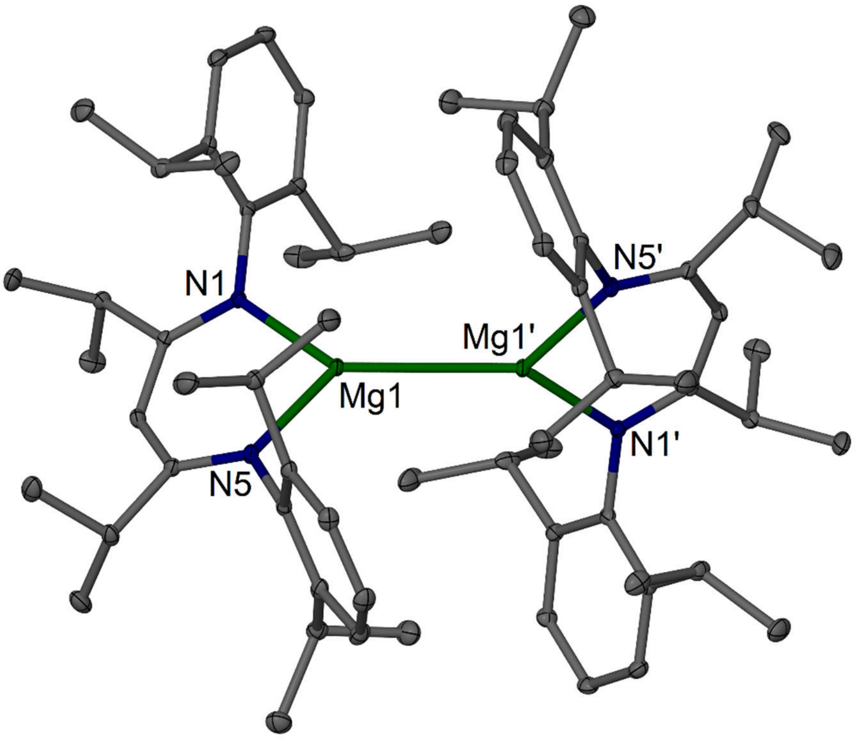

Figure S181. Molecular structure of one independent molecule of [{(^{iPrDip}nacnac)Mg}$_2$] **2c** (30% thermal ellipsoids). Hydrogen atoms and minor component of disorder are omitted for clarity. Selected bond lengths (Å) and angles (°): Molecule 1: Mg1-Mg1' 2.8655(10), Mg1-N1 2.0740(14), Mg1-N5 2.0689(15); N5-Mg1-N1 92.65(5), N1-Mg1-Mg1' 136.48(4), N5-Mg1-Mg1' 130.25(4). Molecule 2: Mg2-Mg2' 2.8739(10), Mg2-N41 2.0653(14), Mg2-N45 2.0734(14); N41-Mg2-N45 91.90(5), N41-Mg2-Mg2' 133.28(4), N45-Mg2-Mg2' 134.42(4).

[(^{EtDip}nacnac)Mg*n*Bu] 9b

The compound crystallised with a full molecule in the asymmetric unit. The molecule is generally poorly ordered and several substituents (*n*Bu, one *i*Pr, two Et) were modelled with two positions for each atom and refined using geometry restraints.

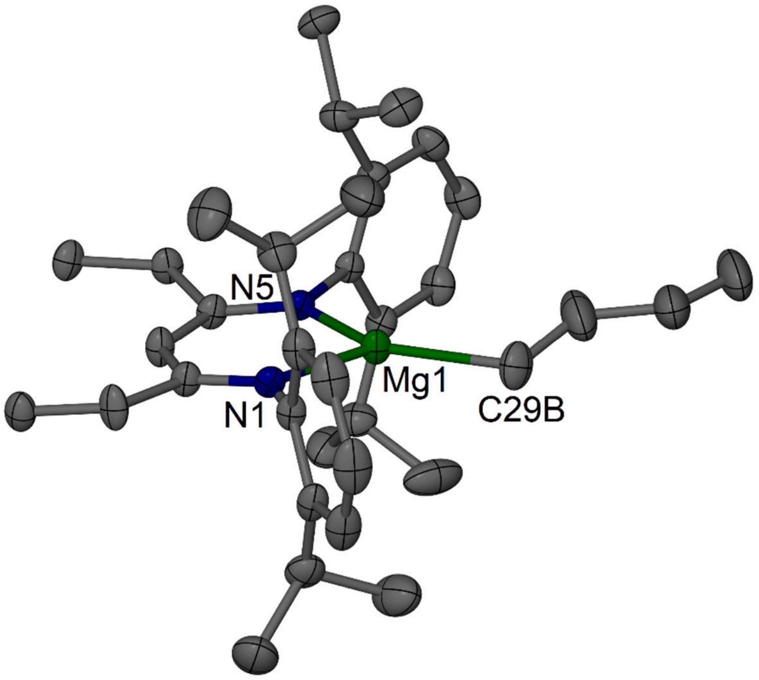

Figure S182. Molecular structure of [(^{RDip}nacnac)Mg*n*Bu] **9b** (30% thermal ellipsoids). Hydrogen atoms and minor component of disorder are omitted for clarity. Selected bond lengths (Å) and angles (°): Mg1-N1 2.0165(11), Mg1-N5 2.0114(11), Mg1-C29A 2.090(9), Mg1-C29B 2.120(8); N5-Mg1-N1 93.33(5), N1-Mg1-C29A 134.6(2), N5-Mg1-C29A 131.5(2), N1-Mg1-C29B 137.0(2), N5-Mg1-C29B 128.6(2).

[{(^{EtDip}nacnac)Mg(μ-O*n*Bu)}$_2$]

The compound crystallised with half a molecule and two benzene molecules in the asymmetric unit. One benzene molecule is disordered and was modelled with two positions for each atom and refined using geometry restraints.

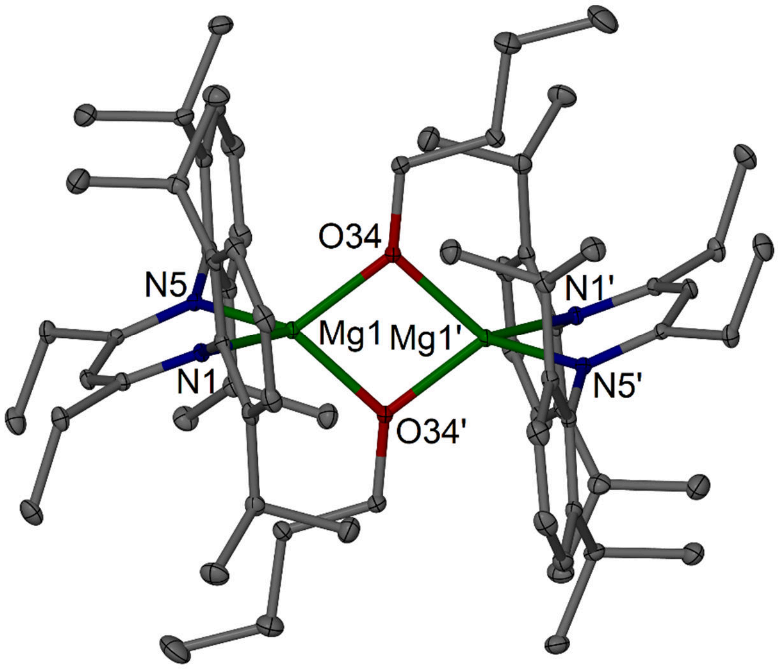

Figure S183. Molecular structure of [{(^{EtDip}nacnac)Mg(μ-O*n*Bu)}$_2$]·4 C_6H_6 (30% thermal ellipsoids). Hydrogen atoms and solvent are omitted for clarity. Selected bond lengths (Å) and angles (°): Mg1-O34 1.9633(10), Mg1-O34' 1.9805(10), Mg1-N1 2.0985(11), Mg1-N5 2.0933(12); N5-Mg1-N1 92.34(4), O34-Mg1-O34' 80.78(4).

[{(^{EtDip}nacnac)Mg(μ-H)}$_2$] 7b: 7b' and 7b''

Two independent molecular structures were obtained. In the first (**7b'**, Figure S183), the compound crystallised with half a molecule in the asymmetric unit showing two independent Mg-H positions. In the second (**7b''**, Figure S184), the compound crystallised with half a full molecule in the asymmetric unit. Two independent H positions were found, leading to symmetry-disordered hydrides, and a residual electron density peak was refined (with geometry restraint) as a low occupancy (20% OH in the dimeric molecule) hydroxide group.

The two structures of [{(^{EtDip}nacnac)Mg(μ-H)}$_2$] **7b** are essentially isomorphous to the two determined structures of [{(^{EtDip}nacnac)Mg}$_2$] **2b** (see Table S1). The structure pairs, **2b'** and **7b'**, and **2b''** and **7b''**, give almost identical unit cells and show essentially the same overall structures (except for Mg_2 versus Mg_2H_2 cores) and orientations of the ligand substituents. All four structures show broadly similar "twisting" and "bending" values (Table S2) that suggest that ligand bulk and crystal packing play an important role in determining the overall geometry in these molecular structures.

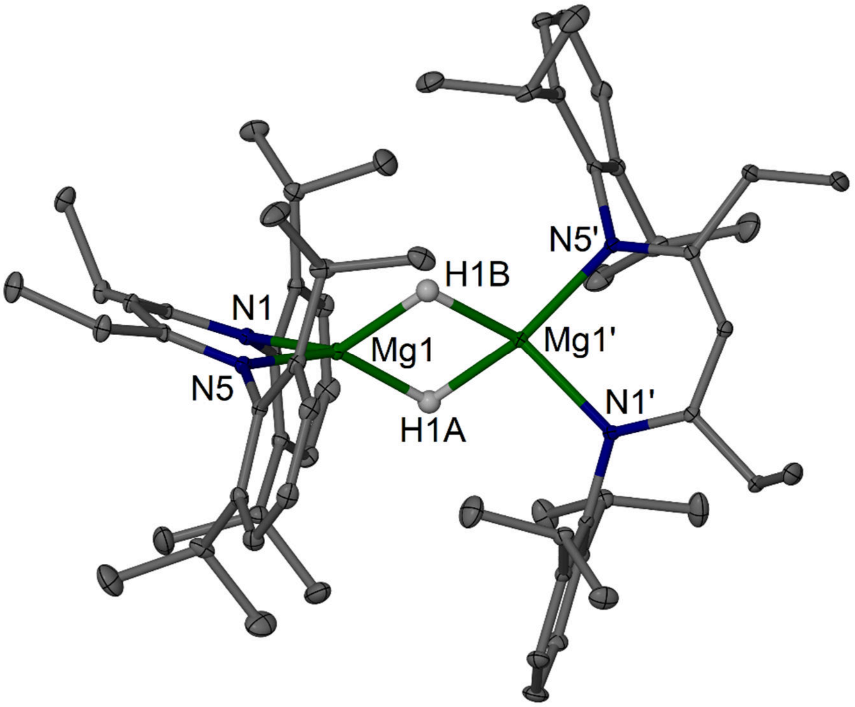

Figure S184. Molecular structure of [{(^{EtDip}nacnac)Mg(μ-H)}$_2$] **7b'** (30% thermal ellipsoids). Hydrogen atoms, except MgH, are omitted for clarity. Selected bond lengths (Å) and angles (°) for **7b'**: Mg1-N1 2.0492(11), Mg1-N5 2.0535(12), Mg1-H1A 1.857(16), Mg1-H1B 1.831(16), Mg1····Mg1 2.8424(8); N1-Mg1-N5 92.95(4), H1A-Mg1-H1B 79.2(8).

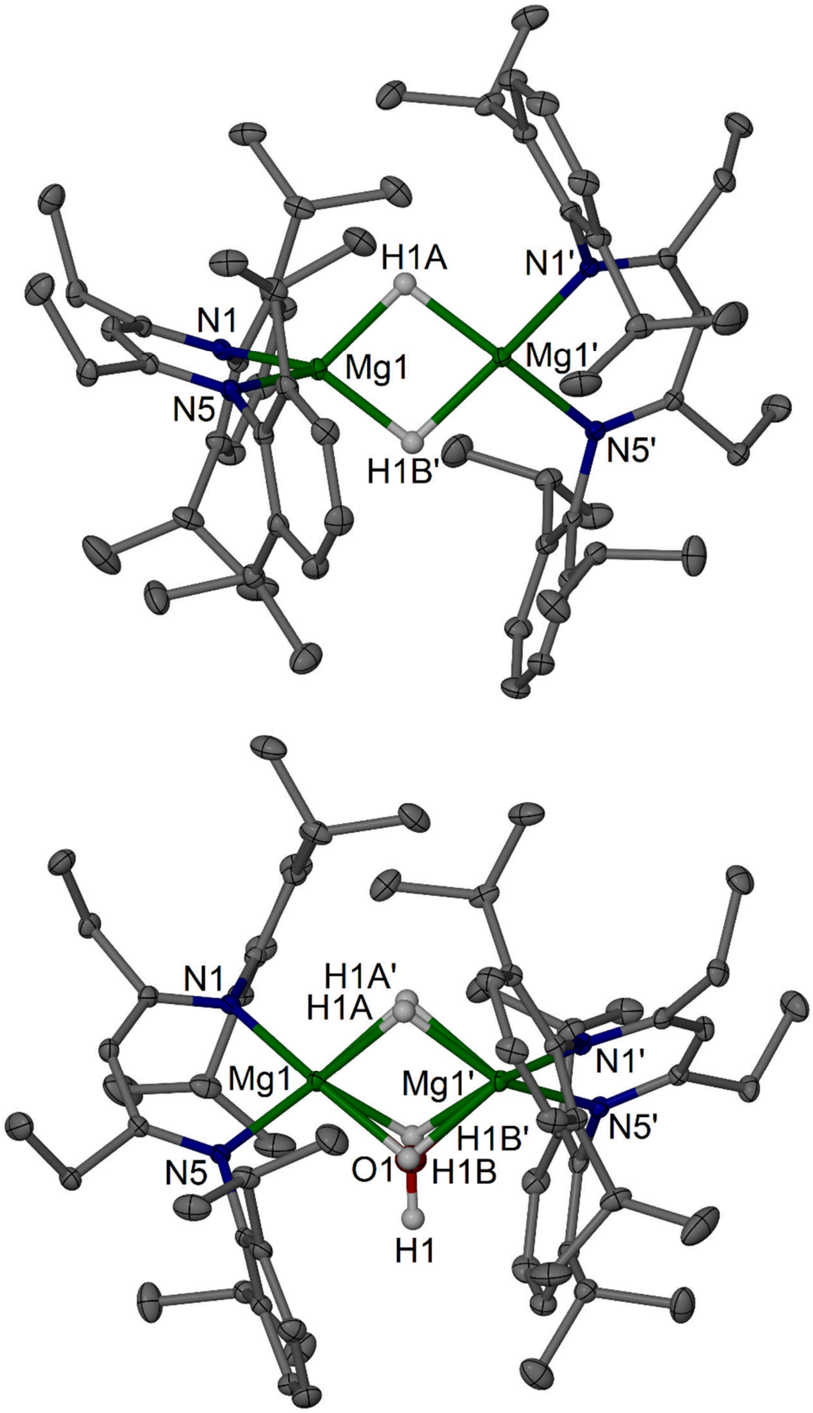

Figure S185. Two views of the molecular structure of $[\{(^{EtDip}nacnac)Mg(\mu\text{-}H)\}_2]$ **7b''** (30% thermal ellipsoids). Hydrogen atoms, except MgH and MgOH, are omitted for clarity. The bottom view shows symmetry-disordered MgH as well as the MgOH positions. Selected bond lengths (Å) and angles (°) for **7b''**: Mg1-N1 2.0483(15), Mg1-N5 2.0429(16), Mg1-H1A 1.90(5), Mg1'-H1A 1.88(5), Mg1-H1B 1.91(3), Mg1'-H1B 1.85(9), Mg1-O1 1.897(10), Mg1····Mg1' 2.8412(11); N5-Mg1-N1 92.86(6), H1B-Mg1-H1A 75(3).

[(^{EtDip}nacnac)Mg(DMAP)H] 12b

The compound crystallised with a full molecule in the asymmetric unit.

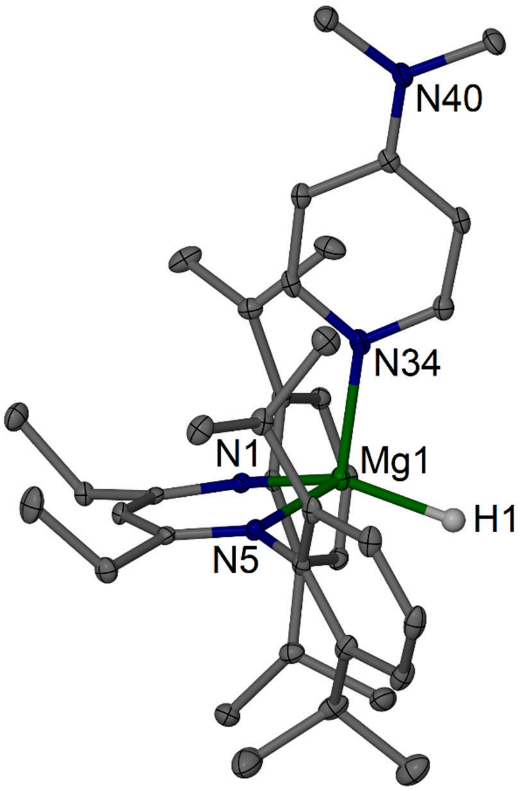

Figure S186. Molecular structure of [(^{EtDip}nacnac)Mg(DMAP)H] **12b** (30% thermal ellipsoids). Hydrogen atoms, except the MgH, are omitted for clarity. Selected bond lengths (Å) and angles (°): Mg1-N1 2.0673(15), Mg1-N5 2.0675(15), Mg1-N34 2.1109(17), Mg1-H1 1.800(16); N1-Mg1-N5 92.22(6), N34-Mg1-H1 103.8(5).

[{(^{iPrDip}nacnac)Mg}$_2$(μ-O)] 1c

The compound crystallised with half a molecule in the asymmetric unit. An image from a partial, poor-quality crystal structure had previously been reported,[4] and the overall geometry and Mg–O and Mg–N distances agree well with those determined by a DFT computational study.[17]

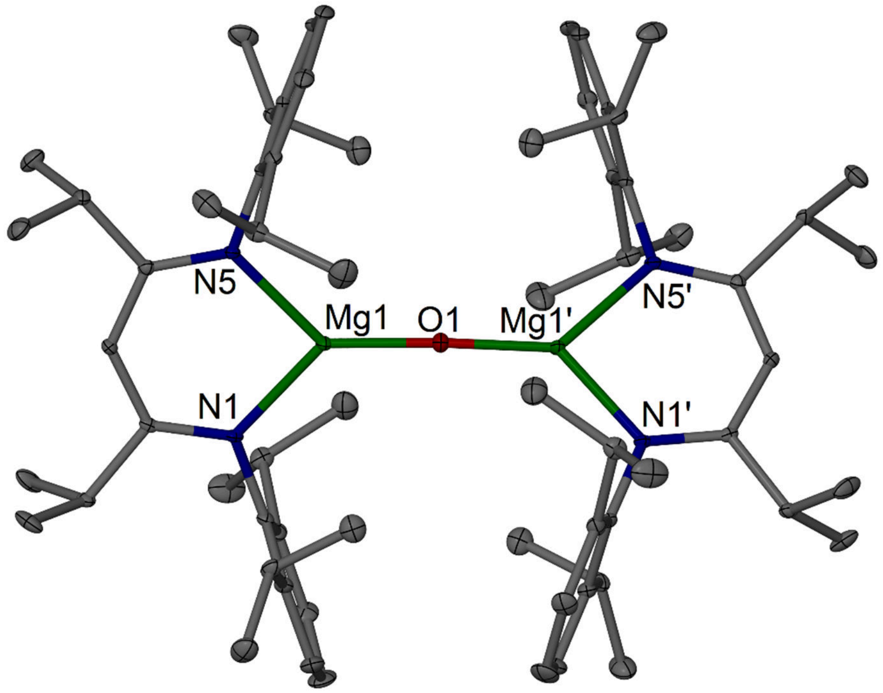

Figure S187. Molecular structure of [{(^{iPrDip}nacnac)Mg}$_2$(μ-O)] **1c** (30% thermal ellipsoids). Hydrogen atoms are omitted for clarity. Selected bond lengths (Å) and angles (°): Mg1-O1 1.8042(7), Mg1-N1 2.0512(19), Mg1-N5 2.0410(18), Mg1····Mg1 3.6073(14); O1-Mg1-N1 134.21(10), O1-Mg1-N5 132.41(9), N5-Mg1-N1 93.21(7), Mg1-O1-Mg1' 177.28(16).

[{(^{MeDip}nacnac)Mg}$_2$(μ-H))(μ-OH)] 4a

The compound crystallised with half a molecule in the asymmetric unit. The OH and H positions are disordered by symmetry over both bridging positions. One isopropyl group per half molecule is disordered and was modelled with two positions for atoms of the methyl groups.

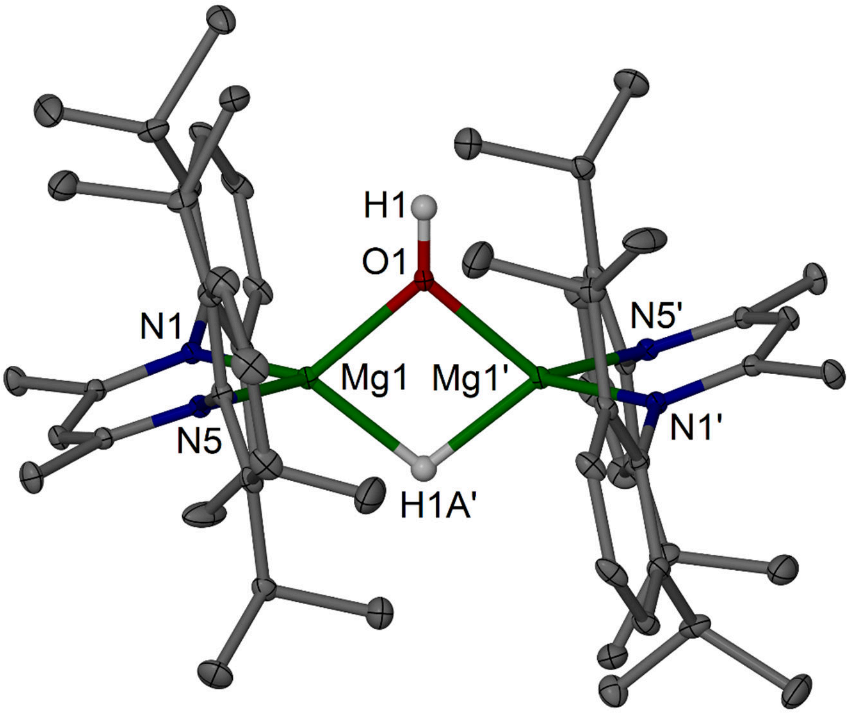

Figure S188. Molecular structure of [{(^{MeDip}nacnac)Mg}$_2$(μ-H))(μ-OH)] **4a** (30% thermal ellipsoids). Hydrogen atoms, except MgH and MgOH, and minor component of disorder are omitted for clarity. Selected bond lengths (Å) and angles (°): Mg1-O1 1.981(2), Mg1'-O1 1.9886(19), Mg1-N1 2.0750(11), Mg1-N5 2.0700(10), Mg1-H1A 1.91(2), Mg1'-H1A 1.91(2), Mg1····Mg1' 2.9843(8); O1-Mg1-O1' 82.52(9), N5-Mg1-N1 92.29(4).

[{(^{EtDip}nacnac)Mg}$_2$(μ-H))(μ-OH)] 4b

The compound crystallised with half a molecule in the asymmetric unit with independent OH and H positions that lead to two close symmetry-disordered OH positions in the dimeric unit.

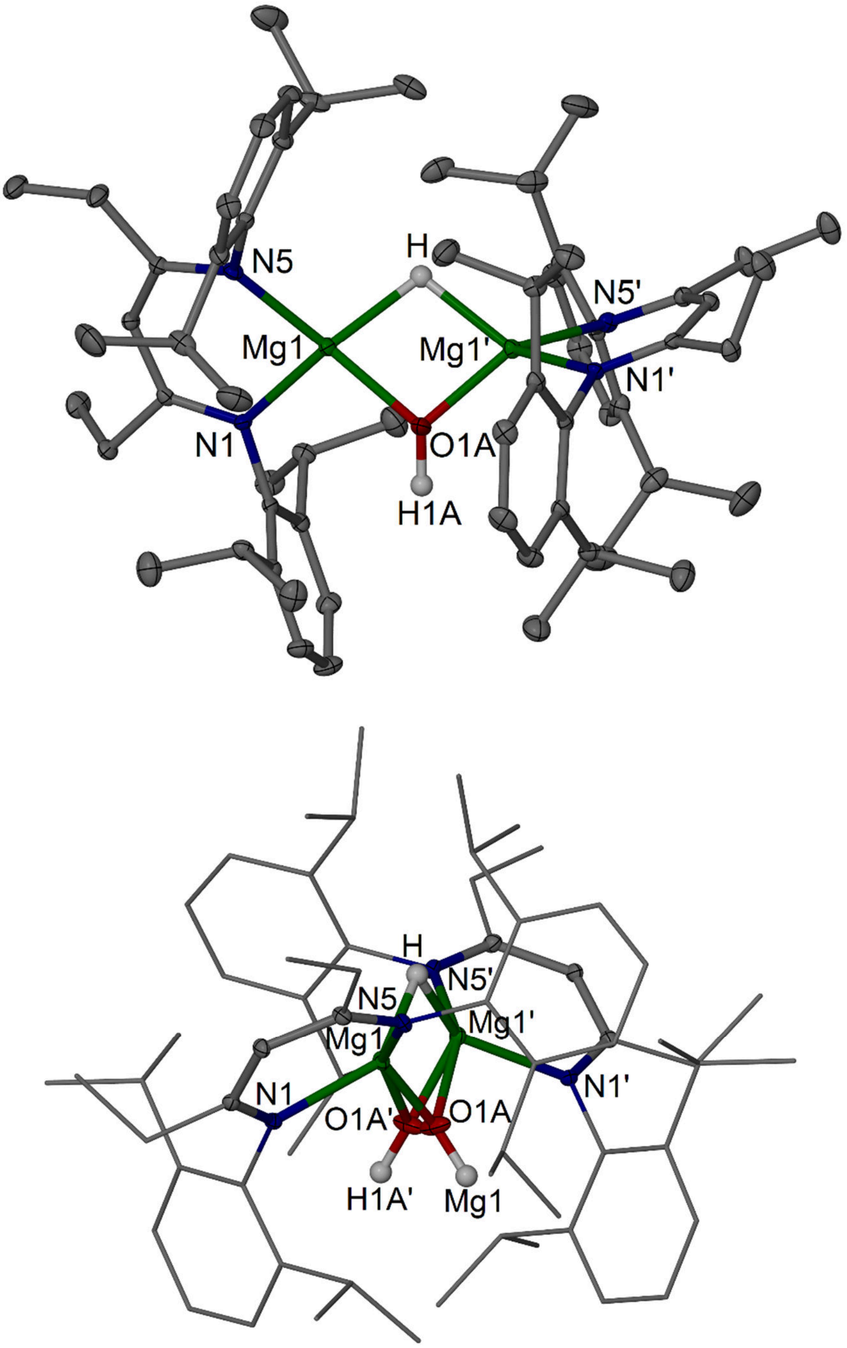

Figure S189. Molecular structure of [{(^{EtDip}nacnac)Mg}$_2$(μ-H))(μ-OH)] **4b** (30% thermal ellipsoids). Hydrogen atoms, except MgH and MgOH, are omitted for clarity. The bottom image shows partial wireframe and illustrates both symmetry-disordered MgOH positions. Selected bond lengths (Å) and angles (°): Mg1-O1A 1.902(19), Mg1-O1A’ 1.958(19), Mg1-N1 2.0569(16), Mg1-N5 2.0565(17),

Mg1-H 1.891(16), O1A-H1A 0.957(19), Mg1····Mg1 2.9161(13); N5-Mg1-N1 92.02(6), Mg1-O1A-Mg1' 98.11(17), O1A-Mg1-H 80.7(8), O1A-Mg1'-H 79.3(7).

[{(^{iPrDip}nacnac)Mg}$_2$(μ-H))(μ-OH)] 4c

The compound crystallised with two highly similar half molecules in the asymmetric unit. Both show independent OH and H positions that lead to two close symmetry-disordered positions for both OH and H, in the dimeric units.

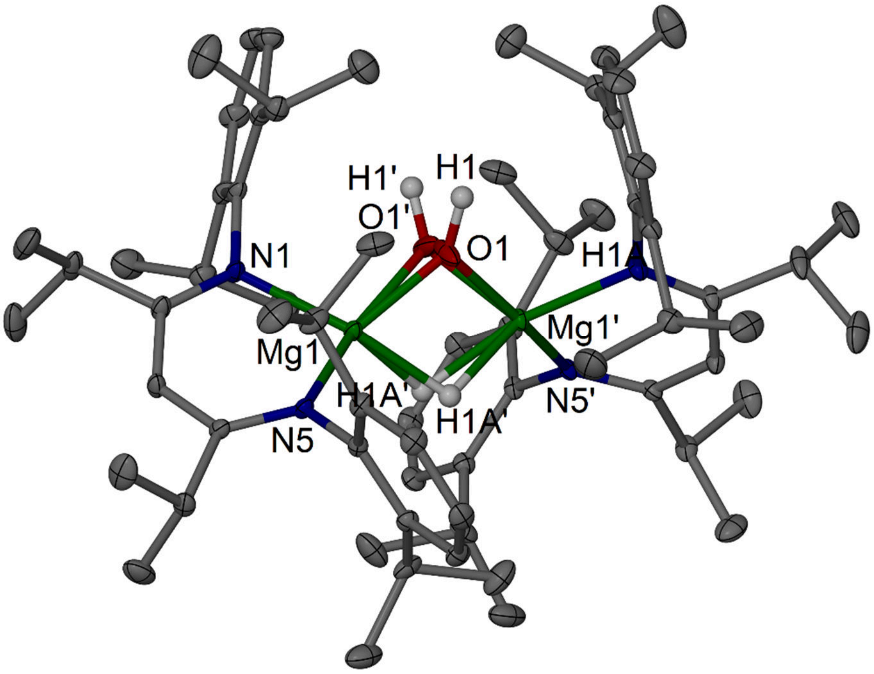

Figure S190. Molecular structure of one independent molecule of [{(^{iPrDip}nacnac)Mg}$_2$(μ-H))(μ-OH)] **4c** (30% thermal ellipsoids). Hydrogen atoms, except MgH and MgOH, are omitted for clarity, and the image illustrates both symmetry-disordered MgOH and MgH positions. Selected bond lengths (Å) and angles (°): Molecule 1: Mg1-O1 1.891(4), Mg1'-O1 1.955(4), Mg1-N1 2.0670(16), Mg1-N5 2.0653(16), Mg1-H1A 1.87(5), Mg1'-H1A 1.82(5), Mg1····Mg1 2.9294(12); N5-Mg1-N1 93.05(6), Mg1-O1-Mg1' 99.20(13). Molecule 2: Mg2-O2 1.921(7), Mg2'-O2 1.934(7), Mg2-N41 2.0689(16), Mg2-N45 2.0584(16), Mg2-H2A 1.85(5), Mg2'-H2A 1.86(5), Mg2····Mg2' 2.9238(11); N45-Mg2-N41 92.78(6), Mg2-O2-Mg2' 98.65(13).

4a

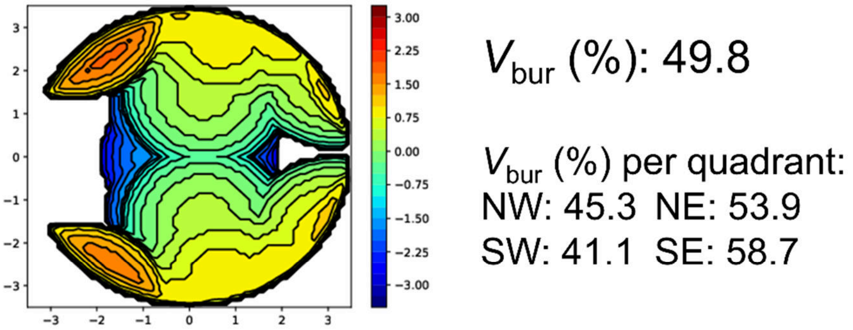

4b

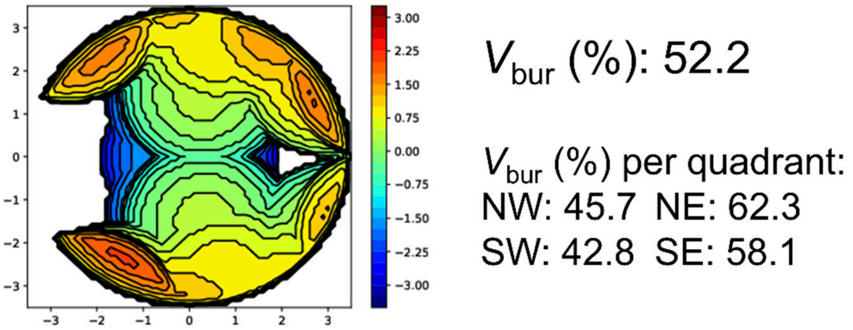

4c

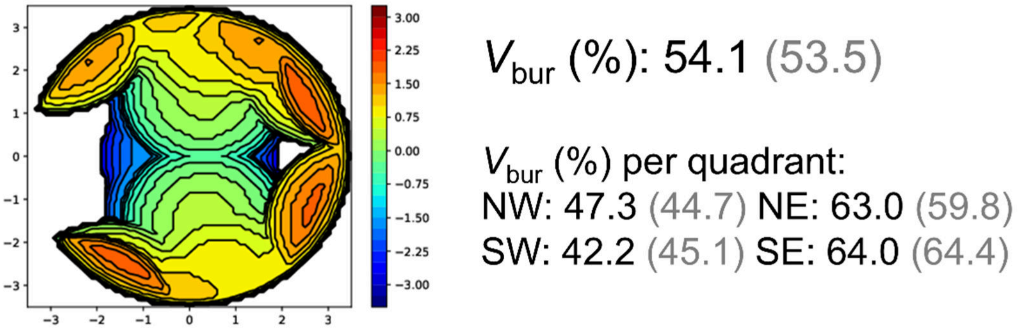

Figure S191. Buried volume (V_{bur}) analysis[17] of the molecular structures of [{(^{RDip}nacnac)Mg}$_2$(μ-H))(μ-OH)] **4a**, **4b** and **4c**. For **4c**, the values for Mg1 are used and the values for Mg2 are given in brackets.

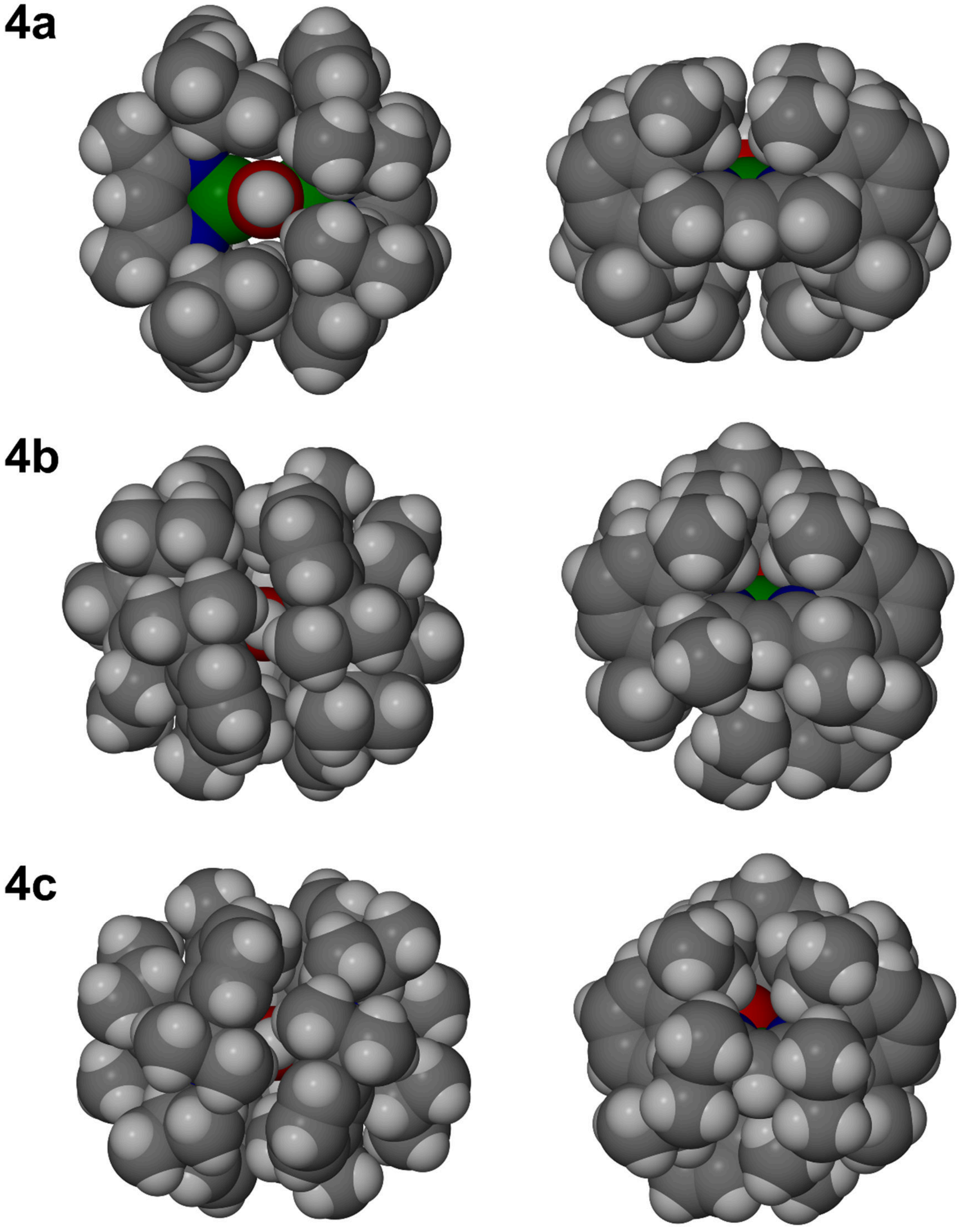

Figure S192. Space-filling models (van der Waals spheres[18]) of the molecular structures of [{(^{RDip}nacnac)Mg}$_2$(μ-H))(μ-OH)] **4a**, **4b** and **4c**. The images on the left show a view that looks down on the H–O bond and the images on the right show a view along the Mg····Mg vector. Note the different orientations of the two β-diketiminate ligand planes (Table S2) that lead to co-planar (**4a**) versus broadly orthogonal ligand arrangements (**4b**, **4c**) and the influence of the backbone substituents on the steric crowding in the complexes.

[{(^{EtDip}nacnac)Mg(THF)}$_2$(μ-O)] 5b

The compound crystallised with half a molecule and half a molecule of benzene in the asymmetric unit. Some geometric restraints were required for parts of the coordinated THF molecule.

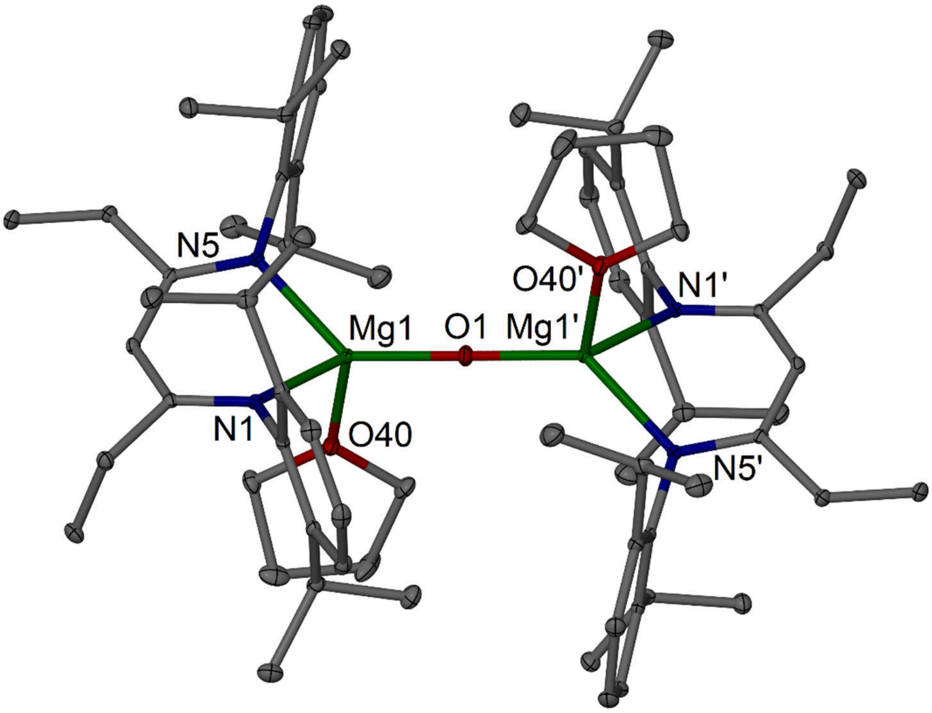

Figure S193. Molecular structure of [{(^{EtDip}nacnac)Mg(THF)}$_2$(μ-O)]·C_6H_6 **5b**·C_6H_6 (30% thermal ellipsoids). Hydrogen atoms and solvent are omitted for clarity. Selected bond lengths (Å) and angles (°): Mg1-O1 1.8260(4), Mg1-O40 2.0894(10), Mg1-N1 2.1102(11), Mg1-N5 2.1112(11); O1-Mg1-O40 100.95(3), N1-Mg1-N5 88.89(4), Mg1-O1-Mg1’ 180.0.

[{(^{iPrDip}nacnac)Mg(DMAP)}$_2$(μ-O)] 6c

The compound crystallised with half a molecule in the asymmetric unit.

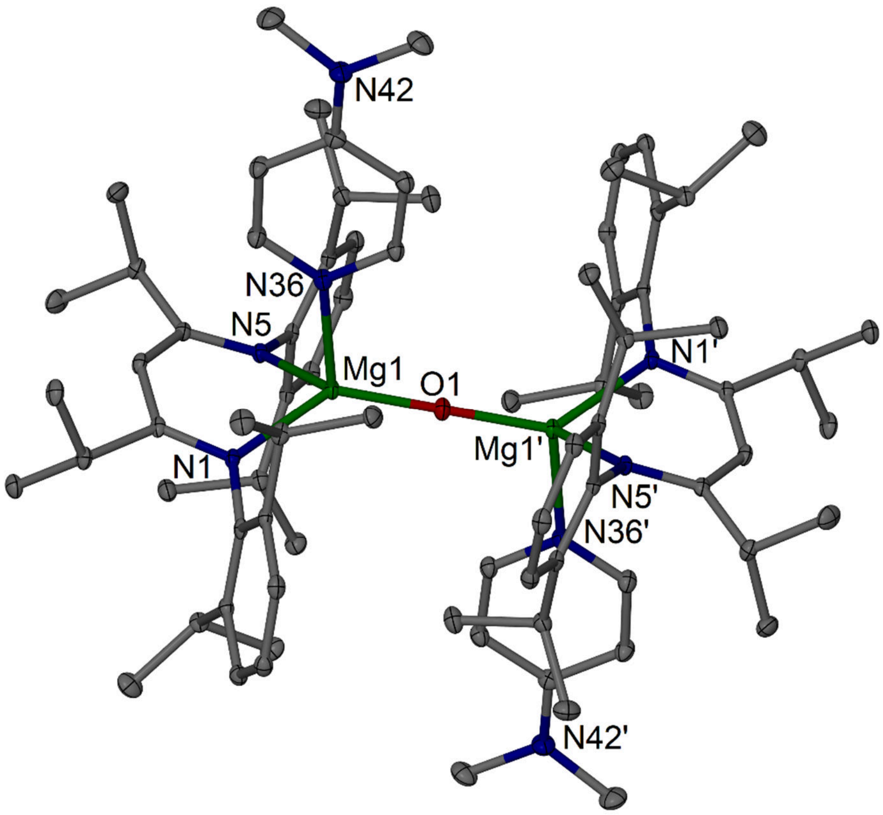

Figure S194. Molecular structure of [{(^{iPrDip}nacnac)Mg(DMAP)}$_2$(μ-O)] **6c** (30% thermal ellipsoids). Hydrogen atoms are omitted for clarity. Selected bond lengths (Å) and angles (°): Mg1-O1 1.8348(5), Mg1-N1 2.1375(14), Mg1-N5 2.0951(14), Mg1-N36 2.1798(14), Mg1····Mg1' 3.6695(10); N5-Mg1-N1 90.77(5), Mg1-O1-Mg1' 180.00(2), O1-Mg1-N36 104.35(4).

[{(^{EtDip}nacnac)Mg(μ-OH)}$_2$] 3b

The compound crystallised with half a molecule in the asymmetric unit.

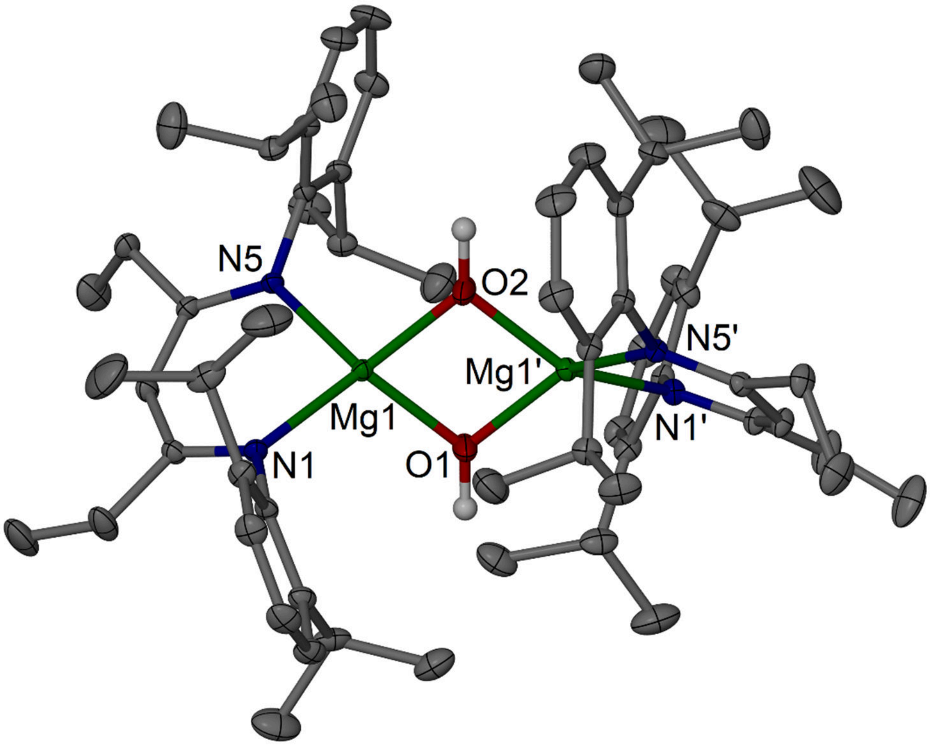

Figure S195. Molecular structure of [{(^{EtDip}nacnac)Mg(μ-OH)}$_2$] **3b** (30% thermal ellipsoids). Hydrogen atoms, except MgOH, are omitted for clarity. Selected bond lengths (Å) and angles (°): Mg1-O1 1.9293(14), Mg1-O2 1.9392(14), Mg1-N1 2.0744(16), Mg1-N5 2.0581(15), O1-H1 0.949(19), Mg····Mg 2.9751(11); N5-Mg1-N1 91.90(6), O1-Mg1-O2 79.46(7), Mg1-O1-Mg1' 100.89(10), Mg1-O2-Mg1' 100.18(10).

[{(^{iPrDip}nacnac)Mg(μ-OH)}$_2$] 3c

The compound crystallised with half a molecule and three half benzene molecules in the asymmetric unit. In the half molecule of **3c**, two isopropyl groups are disordered; one was modelled with two positions for each atom, one with two positions for each methyl group. One benzene molecule is disordered and was refined using two positions for each atom. Geometry restraints were applied to all disordered groups.

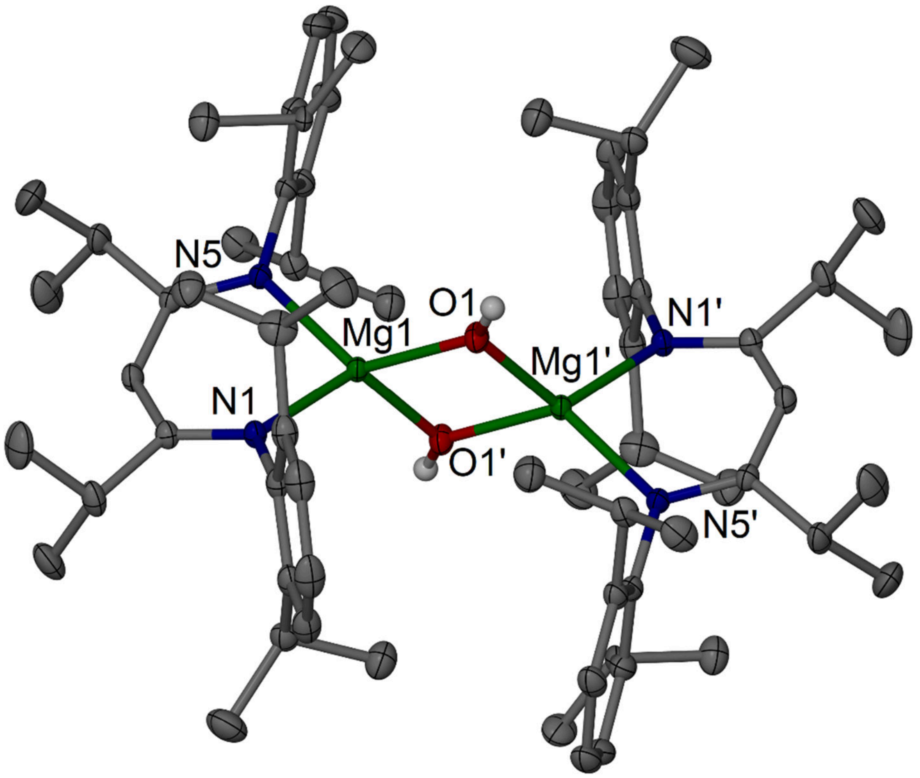

Figure S196. Molecular structure of [{(^{iPrDip}nacnac)Mg(μ-OH)}$_2$]·C_6H_6 **3c**·C_6H_6 (30% thermal ellipsoids). Hydrogen atoms, except MgOH, solvent, and minor components of disorder are omitted for clarity. Selected bond lengths (Å) and angles (°): Mg1-O1 1.9613(11), Mg1-O1' 1.9688(11), Mg1-N1 2.0946(12), Mg1-N5 2.1102(12), Mg1····Mg1' 3.0188(9); O1-Mg1-O1' 79.63(5), N1-Mg1-N5 91.84(5), Mg1-O1-Mg1' 100.37(5).

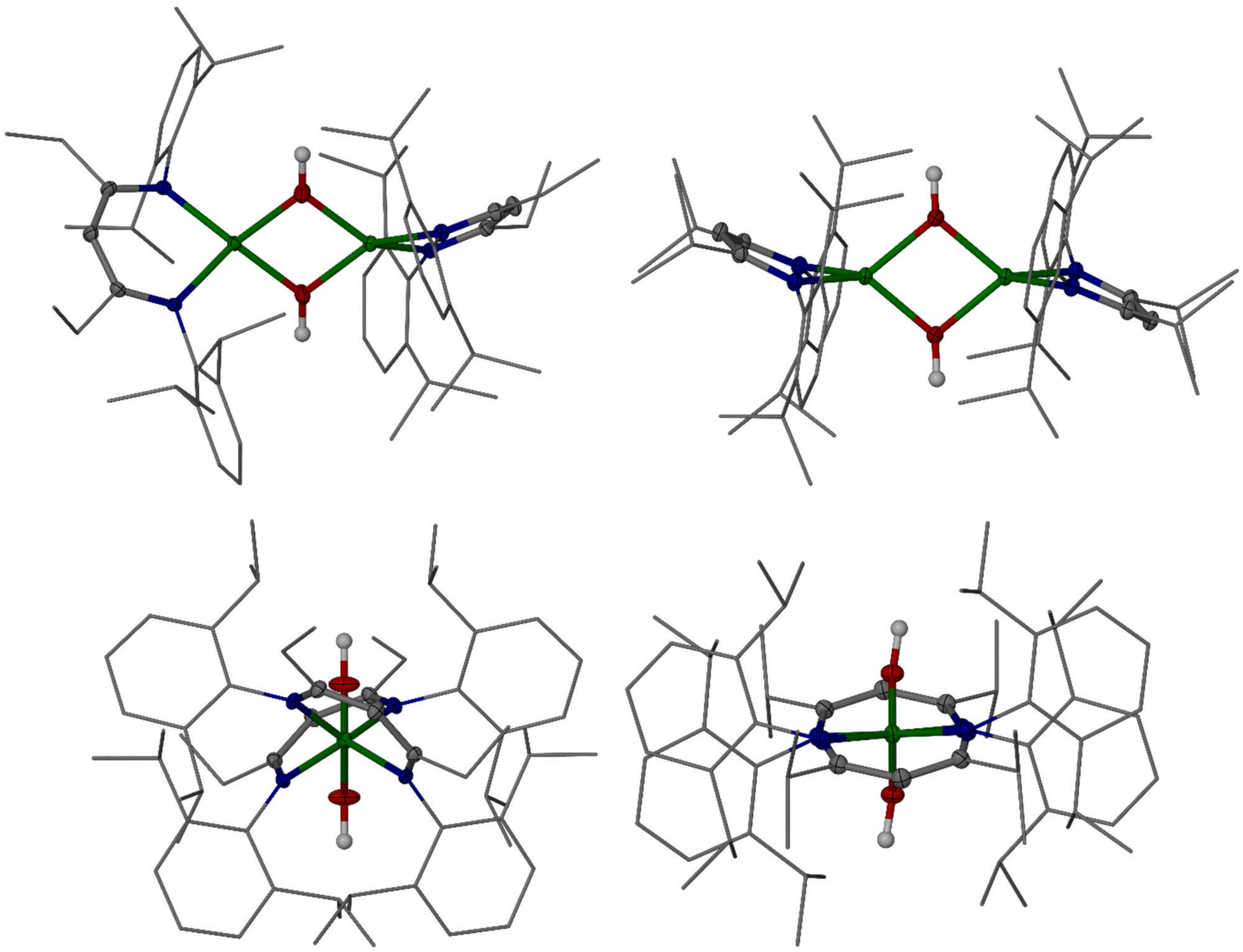

Figure S197. Comparison between the molecular structures of [{(^{EtDip}nacnac)Mg(μ-OH)}$_2$] **3b** (left) and [{(^{iPrDip}nacnac)Mg(μ-OH)}$_2$] **3c** (right) (30% thermal ellipsoids, wireframe). Only the OH hydrogen atoms are shown for clarity. The bottom view is along the Mg····Mg vector. Complex **3b** crystallised in an approximate orthogonal arrangement whereas complex **3c** crystallised in a co-planar ligand arrangement.

[{(^{EtDip}nacnac)Mg(DMAP)(μ-OH)}$_2$] 8b

The compound crystallised with half a molecule in the asymmetric unit. In contrast to this structure, [(^{EtDip}nacnac)Mg(DMAP)H] **12b** is a monomeric species, despite similar Mg–OH and Mg–H distances - the former are only marginally longer than the latter. This is likely influenced by the "electron-deficient" nature of the ionic Mg–H–Mg bridges (formally 3c2e bonds) whereas Mg–O(H)–Mg bridges are not electron deficient. In the latter, more electron pairs are involved in coordination and "saturation" of the Mg^{2+} ion, and thus a bridging dimeric structure is favoured for the hydroxide complex.

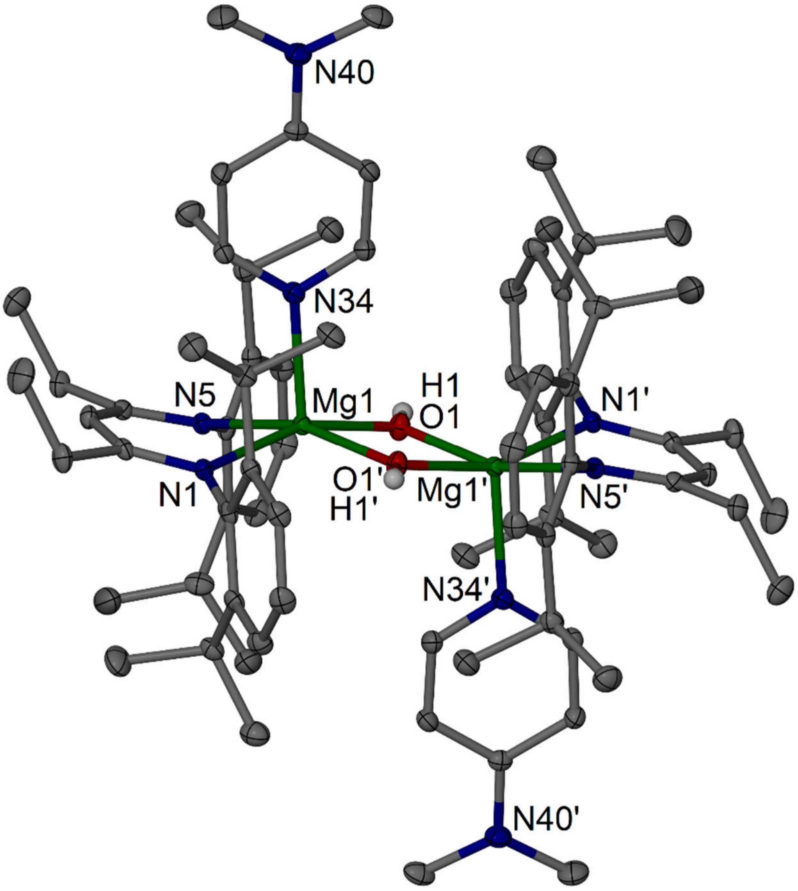

Figure S198. Molecular structure of [{(^{EtDip}nacnac)Mg(DMAP)(μ-OH)}$_2$] **8b** (30% thermal ellipsoids). Hydrogen atoms, except MgOH, are omitted for clarity. Selected bond lengths (Å) and angles (°): Mg1-O1 1.9918(10), Mg1-O1' 2.0129(11), Mg1-N1 2.1964(11), Mg1-N5 2.2082(12), Mg1-N34 2.1799(12), O1-H1 0.949(15), Mg1····Mg1 3.1666(8); O1-Mg1-N34 101.72(5), N1-Mg1-N5 84.52(4).

4 DFT Computational Studies

4.1 General considerations

Compounds were optimised from the starting geometries obtained by X-ray diffraction, or derived from those, using the M06-L[19] density functional coupled with the def2-SVP basis set augmented with a D3 dispersion term,[20] followed by single point calculations at the M06-D3/def2-TZVP level in the gas phase as well as in the solvent phase using the Polarizable Continuum Model.[21,22] Natural Population Analysis (NPA) charges and Wiberg bond orders were calculated using Natural Bond Orbital (NBO) analysis,[23] at the $M06\text{-}D3_{PCM(benzene)}$/def2-TZVP level. Quantum Theory of Atoms in Molecules (QTAIM) analysis was conducted at the same level using AIMALL.[24] The calculations were performed using Gaussian 16.[25] Non-covalent interactions were studied using NCIPLOT.[26]

For the mechanistic analyses, counterpoise correction was added to eliminate Basis Set Superposition Error (BSSE).[27] The transition states (TSs) were characterised by following the intrinsic reaction coordinate (IRC) to determine the minima connecting it.[25] However, when the IRC calculations did not converge properly, conventional geometry optimisations were performed starting from the optimised transition state, where the atoms were displaced manually along the imaginary vibrational mode (both in the forward and reverse directions), to ensure the transition state connects the desired minima.

A computational investigation into the reaction pathway was carried out for the full model system [{(^{iPrDip}nacnac)Mg}$_2$(μ-O)] **1c** and the cut-back model system [{(^{MeMe}nacnac)Mg}$_2$(μ-O)]. The influence of steric effects was further investigated by studying products and transition states of [{(^{EtDip}nacnac)Mg}$_2$(μ-O)] **1b**, and [{(^{iPrDep}nacnac)Mg}$_2$(μ-O)] (by replacing the Dip-isopropyl groups with ethyl groups derived from **1c**), in their reaction with H_2. For all the systems, it was observed that the H_2 molecule approaches the magnesium oxide complexes in one plane with the Mg-O vector. QTAIM was used for the calculation of electron density and its Laplacian, bond critical points, bond paths, and bond ellipticity for the study of the transition state of the reaction of **1c** with H_2.

Gibbs free energies *ΔG* (kcal/mol) for the transition states and products of these studies are listed in Tables S3-S7. Note that only for the bulkiest examples could both co-planar and orthogonal isomer structures be optimised. For the [{(^{MeMe}nacnac)Mg}$_2$(μ-O)] system, the orthogonal geometry optimised to a co-planar one and for the [{(^{iPrDep}nacnac)Mg}$_2$(μ-O)] system, the optimisation of the orthogonal isomer did not converge, despite multiple attempts.

Table S3. Gibbs free energies (ΔG in kcal/mol) for transition states and products in the reactions between magnesium oxide complexes and H_2.

Complex/reaction	TS (kcal/mol)	Product co-planar (kcal/mol)	Product orthogonal (kcal/mol)
**1c→4c** M06-D3/def2-TZVP//M06-L-D3/def2-SVP	18.93	-2.78	-9.77
**1c→4c** M06-D3$_{PCM(benzene)}$/def2-TZVP//M06-L-D3/def2-SVP	18.34	-3.16	-9.91
**1b→4b** M06-D3/def2-TZVP//M06-L-D3/def2-SVP	22.20	-2.57	-7.99
**1b→4b** M06-D3$_{PCM(benzene)}$/def2-TZVP//M06-L-D3/def2-SVP	21.90	-2.98	-7.96
[{(^{iPrDep}nacnac)Mg}$_2$(μ-O)]→ [{(^{iPrDep}nacnac)Mg}$_2$(μ-H)(μ-OH)] M06-D3/def2-TZVP//M06-L-D3/def2-SVP	28.35	-13.30	-
[{(^{iPrDep}nacnac)Mg}$_2$(μ-O)]→ [{(^{iPrDep}nacnac)Mg}$_2$(μ-H)(μ-OH)] M06-D3$_{PCM(benzene)}$/def2-TZVP//M06-L-D3/def2-SVP	26.78	-12.85	-
[{(^{MeMe}nacnac)Mg}$_2$(μ-O)]→ [{(^{MeMe}nacnac)Mg}$_2$(μ-H)(μ-OH)] M06-D3/def2-TZVP//M06-L-D3/def2-SVP	16.73	-19.31	-
[{(^{MeMe}nacnac)Mg}$_2$(μ-O)]→ [{(^{MeMe}nacnac)Mg}$_2$(μ-H)(μ-OH)] M06-D3$_{PCM(benzene)}$/def2-TZVP//M06-L-D3/def2-SVP	17.20	-16.01	-

4.2 The reaction of [{(^{iPrDip}nacnac)Mg}$_2$(*μ*-O)] 1c with H_2 to [{(^{iPrDip}NacNac)Mg}$_2$(μ-H)(μ-OH)] 4c

The computational study of the reaction of **1c** with H_2 shows that the reaction is exergonic by ΔG (298 K) = -9.91 kcal/mol with an activation barrier of $\Delta G^{\ddagger}$(298 K) = 18.34 kcal/mol (Figure S199). A weak adduct of **1c** and H_2 (**Int-1**) was optimised (Figure S200). The reaction was found to proceed via the formation of a weak intermediate **Int-2** where the H_2 molecule shows a close distance to the O atom (2.230 Å) (Figure S200). This is followed by the transition state **TS** where rupture of the H_2 molecule takes place to give **Int-3** with cleaved H–H bond and newly formed hydroxide and hydride ions (Figure S201). A series of related cleaved species (**Int-4**, **Int-5**, plus **Int-6**, see Figure S202, Figure S204) were optimised as part of this study, which can rearrange to the lowest-energy configurations of these ions to afford product [{(^{iPrDip}NacNac)Mg}$_2$(μ-H)(μ-OH)] **4c** (Figure S203). Two product isomers (co-planar, orthogonal) have been optimised, where the nacnac ligands can either take up a co-planar or an orthogonal arrangement. The orthogonal product was found to be more stable by ΔG (298 K) = -6.75 kcal/mol. The energy difference suggests that both isomers are easily accessible at room temperature. An analysis of the co-planar versus orthogonal product structures is provided in the captions of Figure S205.

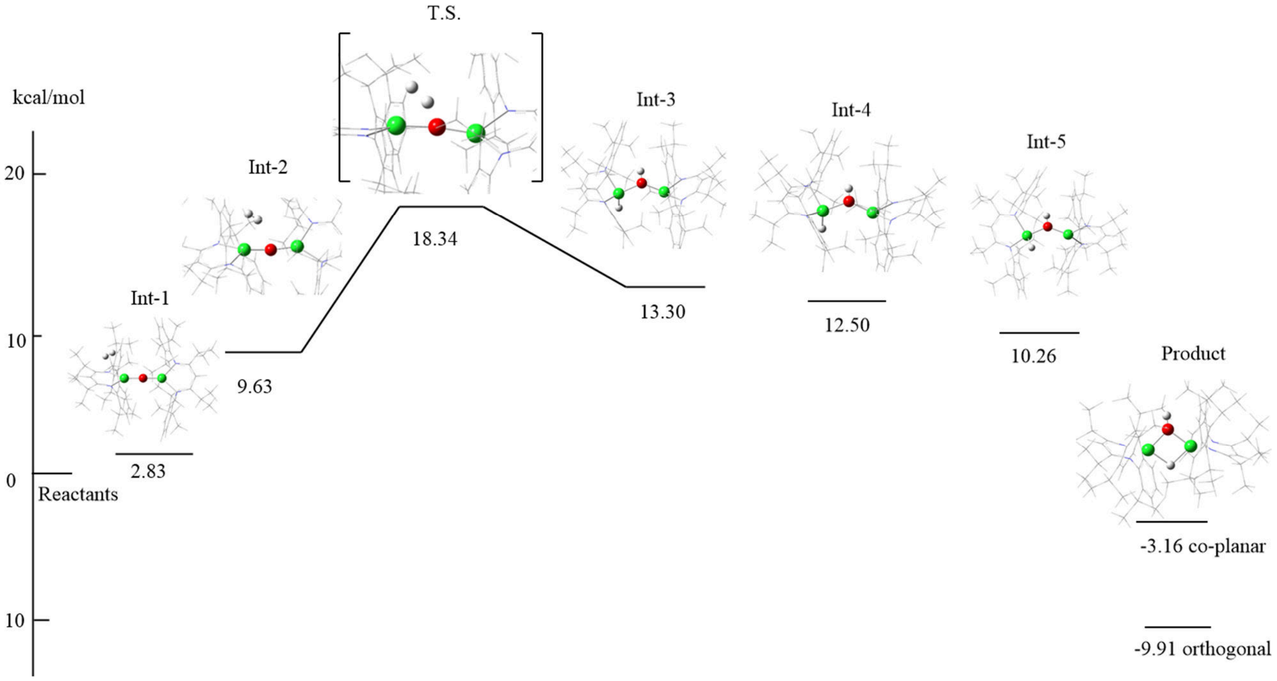

Figure S199. Relative free energies (kcal/mol) of key intermediates and TS in the reaction between **1c** and H_2 computed at M06-D3$_{PCM(benzene)}$/def2-TZVP//M06-L-D3/def2-SVP level of theory. Mg represented as green, O as red and H as white spheres.

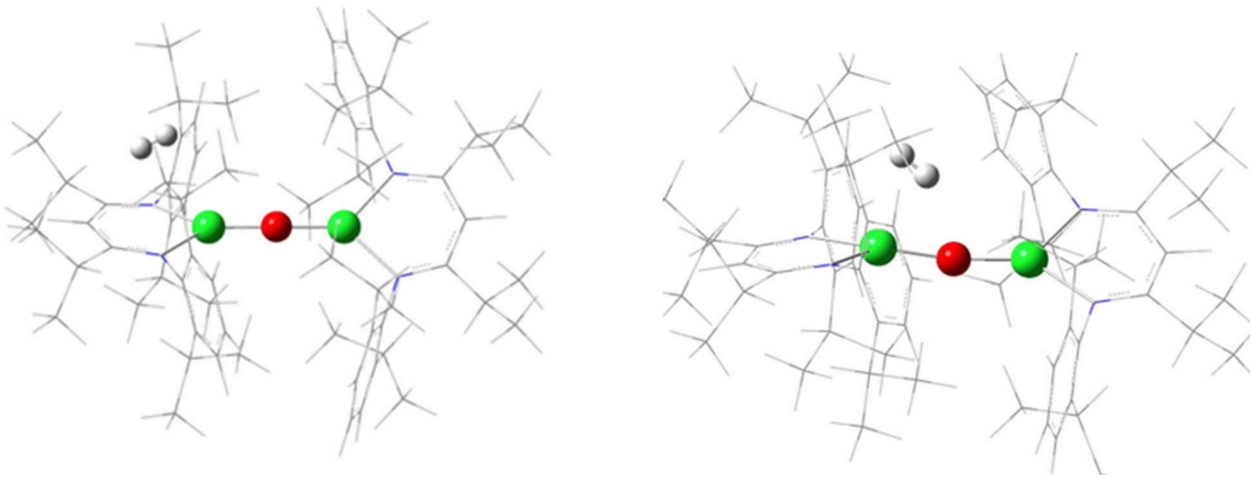

Figure S200. Optimised geometry of **Int-1** (left) and **Int-2** (right) in the reaction between **1c** and H_2 computed at the M06-D3$_{PCM(benzene)}$/def2-TZVP//M06-L-D3/def2-SVP level of theory. Wiberg bond indices for H–H in the H_2 molecular unit are 0.978 in **Int-1** and 0.947 in **Int-2.**

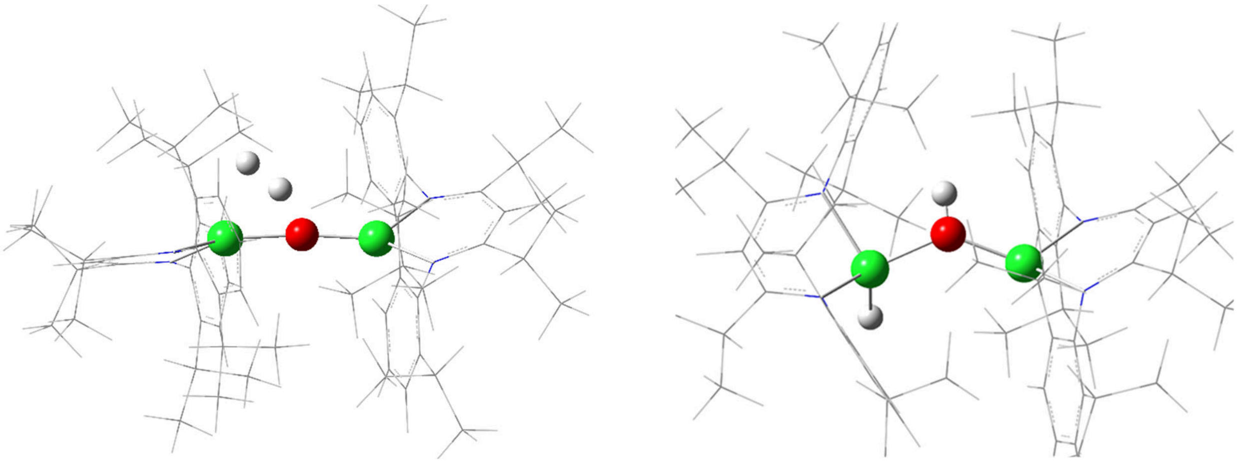

Figure S201. Optimised geometry of **TS** (left) and **Int-3** (right) in the reaction between **1c** and H_2 computed at the M06-D3$_{PCM(benzene)}$/def2-TZVP//M06-L-D3/def2-SVP level of theory. The Wiberg bond index for H·····H in the H_2 molecular unit is 0.556 in the **TS.**

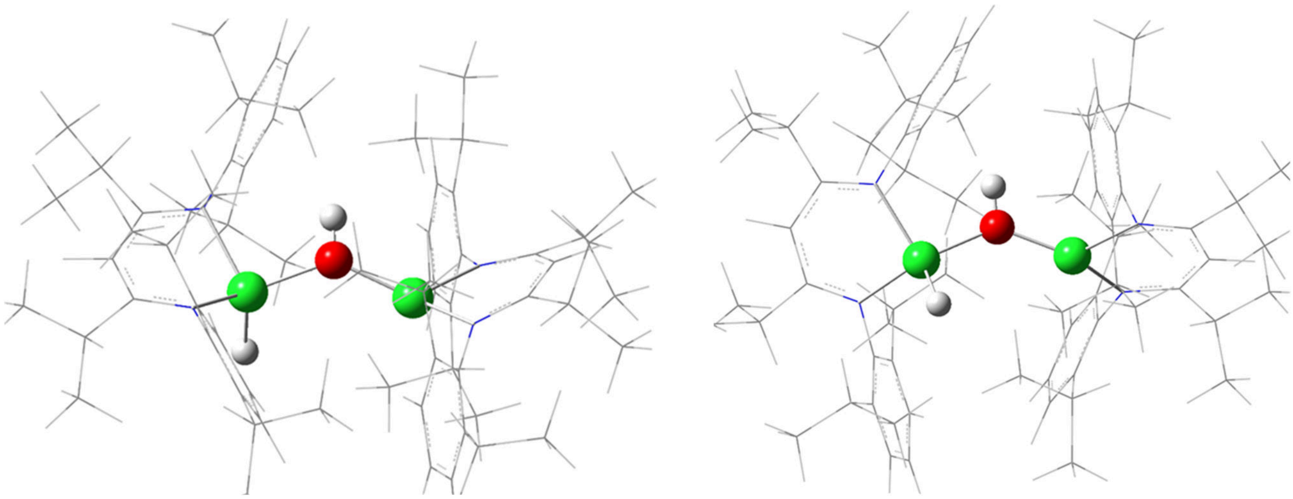

Figure S202. Optimised geometry of **Int-4** (left) and **Int-5** (right) in the reaction between **1c** and H_2 computed at the M06-D3$_{PCM(benzene)}$/def2-TZVP//M06-L-D3/def2-SVP level of theory.

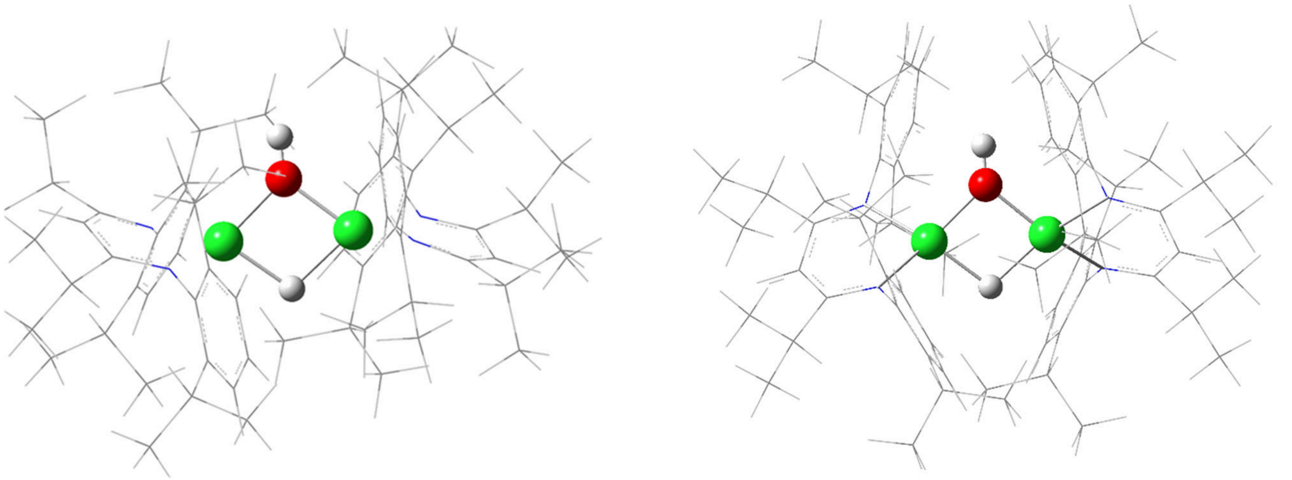

Figure S203. Optimised geometry of **4c, co-planar isomer** (left) and **4c, orthogonal isomer** (right) in the reaction between **1c** and H_2 computed at the M06-D3$_{PCM(benzene)}$/def2-TZVP//M06-L-D3/def2-SVP level of theory.

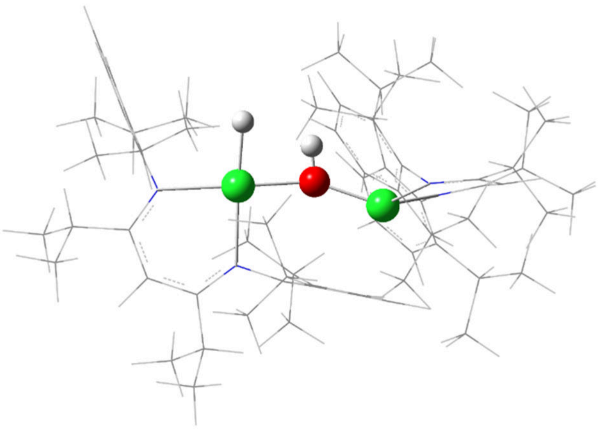

Figure S204. Optimised geometry of **Int-6** (+36.28 kcal/mol), a full-ligand model derived from an intermediate in the cut back-system [{(^{MeMe}nacnac)Mg}$_2$(μ-O)] + H_2 by reintroducing the bulky substituents, followed by optimization at the M06-D3$_{PCM(benzene)}$/def2-TZVP//M06-L-D3/def2-SVP level of theory. This shows a relatively close $H^+\cdots H^-$ distance (2.098 Å) and can be regarded as a high-energy distorted snapshot structure of **4c**.

In the geometries of **Int-3**, **Int-4** and **Int-5** it is observed that the (O–)H and (Mg–)H hydrogen atoms show approximately *syn* to *gauche* arrangements, and not the lower energy *anti* orientation as in product **4c**. Based on a hydrogen addition product obtained from the cut-back model [{(^{MeMe}nacnac)Mg}$_2$(μ-H)(μ-OH)], steric bulk was reintroduced and the geometry optimised. This led to an optimised form of the high-lying (Δ*G* (298 K) = 36.28 kcal/mol) form **Int-6** (Figure S204) that contains the two hydrogen atoms in an approximate *syn*-arrangement with a close $H^+\cdots H^-$ distance. These types of distortions and isomers of complex **4c** are believed to facilitate the reverse reaction to **1c** that eliminates dihydrogen.

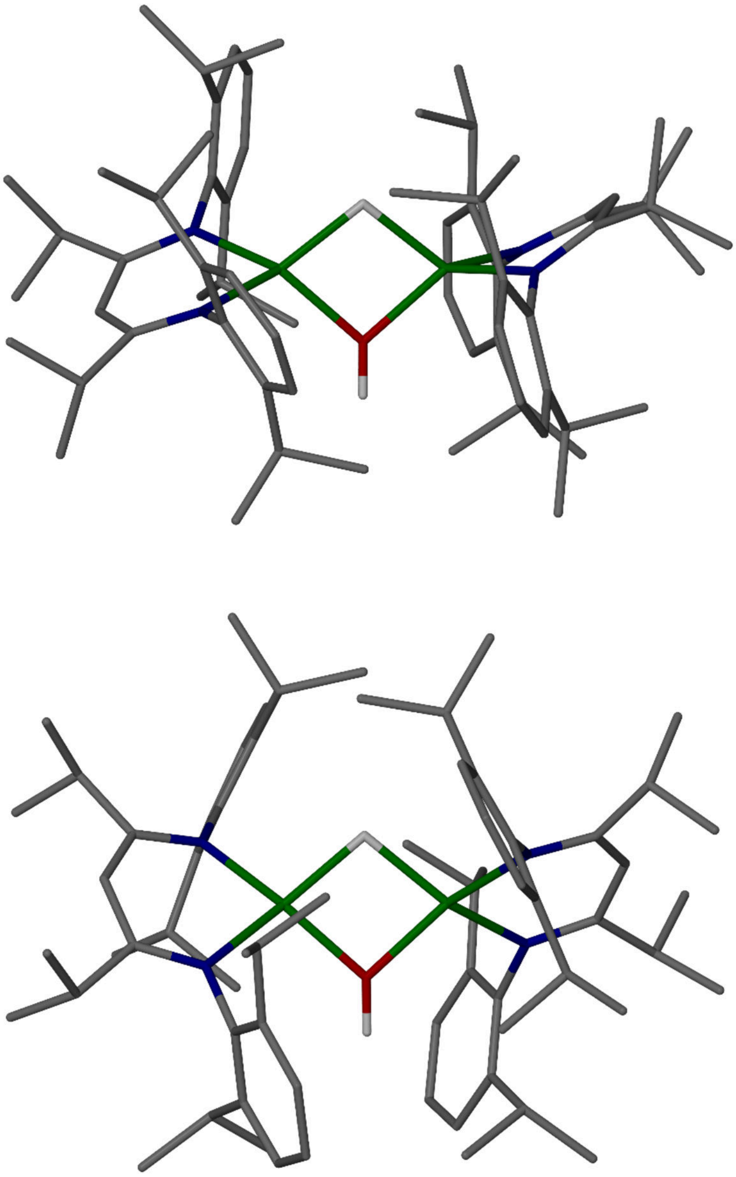

Figure S205. The two main geometries of DFT-optimised [(^{iPrDip}NacNac)Mg(μ-H)(μ-OH)] **4c** (wireframe, c.f. Figure S203) – co-planar isomer top, orthogonal isomer bottom. Mean bond lengths (Å): Co-planar: Mg-O 1.9675, Mg-N 2.082, Mg-H 1.8865, Mg···Mg 2.942; Orthogonal: Mg-O 1.9455, Mg-N 2.0635, Mg-H: 1.882, Mg···Mg 2.899. The magnesium-ligand bond lengths in the co-planar geometry are slightly larger than those in the orthogonal geometry for this ligand (c.f. the molecular structures of the magnesium hydroxide complexes **3b** and **3c**). The orthogonal arrangement appears to allow closer metal-ligand contacts for this specific ligand which may account for the relative stabilisation due to electrostatic effects.

Comparison of energies between computational results obtained for “gas phase” and “solvent phase” products shows that the inclusion of the solvent model (benzene) led to very minor changes only, and generally afforded a small decrease in the energy of the system, see Table S4.

Table S4. Relative free energies (kcal/mol) of key intermediates and TS in the reaction between **1c** and hydrogen using M06-D3$_{PCM(benzene)}$/def2-TZVP//M06L-D3/def2-SVP level of theory in the gas phase and solvent phase.

**Species**	**Δ*G* in gas phase (kcal/mol)**	**Δ*G* in solvent phase (benzene) (kcal/mol)**
Int-1	2.82	2.83
Int-2	9.88	9.63
TS	18.93	18.34
Int-3	14.59	13.30
Int-4	13.78	12.50
Int-5	11.07	10.26
Product co-planar (**4c**)	-2.78	-3.16
Product orthogonal (**4c**)	-9.77	-9.91

To study the amount of strain experienced within the Mg complex fragments alone (minus the H_2 molecular fragment) during the reaction, energy changes of the fragments between **Int-2** and **TS**, and between **TS** and **Int-3**, were compared. It was observed that only minor configurational rearrangements of 1.8 kcal/mol were required in the Mg species (minus the H_2 molecule fragment) upon change from **Int-2** to the **TS**. However, more significant rearrangements occur within the Mg species upon changing from **TS** to **Int-3** requiring an energy change of 30.8 kcal/mol.

To study the effect of dispersion on the system, energy calculations were performed on the transition state at the B3LYP$_{PCM(benzene)}$/def2-TZVP//B3LYP/def2-SVP and B3LYP-D3$_{PCM(benzene)}$/def2-TZVP//B3LYP-D3/def2-SVP level of theory; i.e., without and with D3 addition. It was found that the addition of the dispersion correction causes a significant decrease in energy of Δ*G* (298 K) = -178.47 kcal/mol indicating the importance of dispersion in the system. Some of these are visualised in the NCIPLOT analysis, vide infra (Figure S209), where van der Waals interactions are shown as green areas.

Dissociation of [(^{iPrDip}NacNac)Mg(μ-H)(μ-OH)] 4c

Comparison between the energies of **4c**, and the two monomeric species [(^{iPrDip}nacnac)MgH] and [(^{iPrDip}nacnac)MgOH] (Figure S206), shows that **4c** is much more stable (-38.63 kcal/mol) than the sum of the two monomeric species (energies determined in the solvent phase). Thus, the dissociation of **4c** into its monomeric constituents is energetically unfavourable for the system and is higher than the activation barrier for dehydrogenation via the determined pathway ($\Delta G^{\ddagger}$(298 K) = +18.34 kcal/mol in Figure S199).

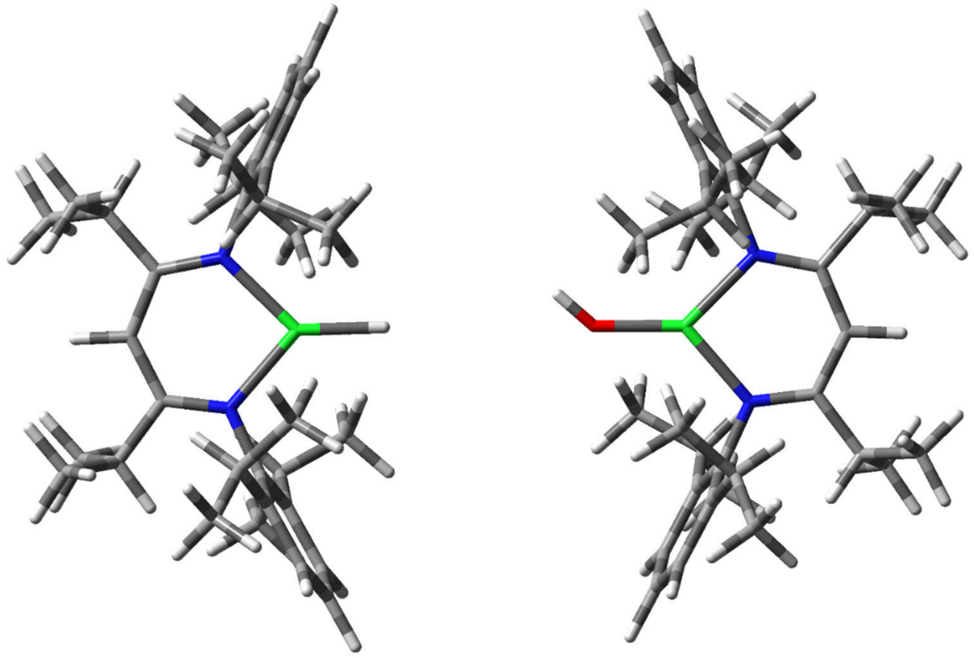

Figure S206. Optimised geometries of the two monomeric species [(^{iPrDip}nacnac)MgH] (left) and [(^{iPrDip}nacnac)MgOH] (right) at the M06-D3$_{PCM(benzene)}$/def2-TZVP//M06-L-D3/def2-SVP level of theory.

The transition state (TS) of [{(^{iPrDip}nacnac)Mg}$_2$(*μ*-O)] 1c + H_2

The approach of the H_2 molecule towards the O atom has been investigated by analysis of the molecular orbitals in the transition state (Figure S207) as well as by QTAIM analysis (Figure S208). This is suggestive of an S_N2 mechanism with simultaneous formation of the hydroxide O–H bond, rupture of H–H bond, and formation of the hydride ion (in the Mg–H interaction), see the main text.

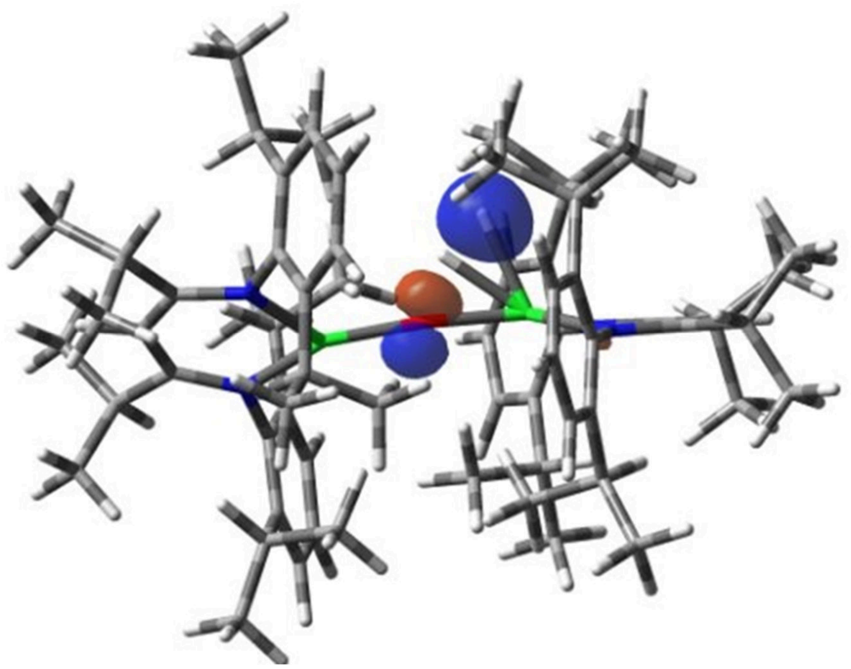

Figure S207. HOMO-2 of the transition state for the reaction of **1c** + H_2 (isovalue 0.06) obtained from optimisation at the M06-D3$_{PCM(benzene)}$/def2-TZVP//M06-L-D3/def2-SVP level of theory.

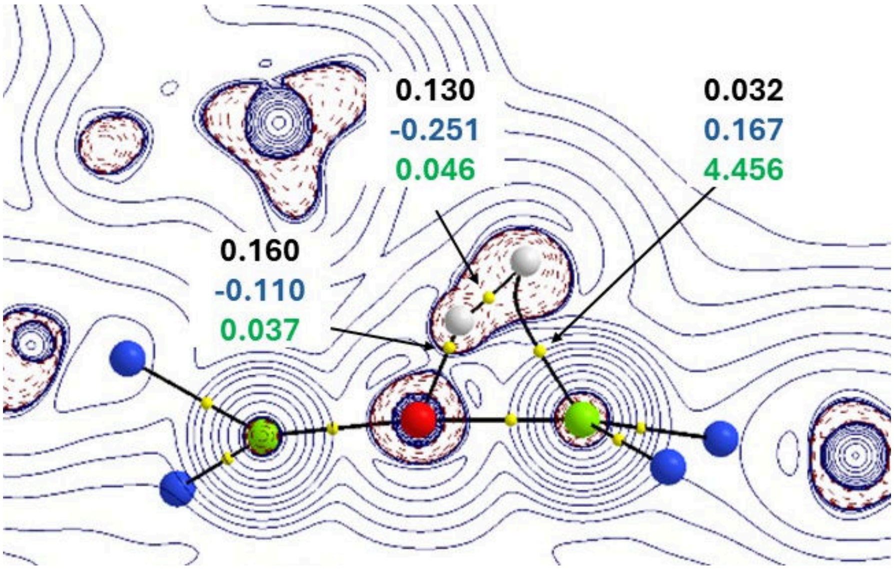

Figure S208. QTAIM contour plots showing the Laplacian of the electron density (solid lines: positive, dashed lines: negative) for the transition state of **1c** + H_2. Bond critical points: yellow, bond paths: black lines, values for the electron density ρ [e/bohr^3], (black), Laplacian, $\nabla^2\rho$ [e/bohr^5], (blue), and bond ellipticity ε, (green) are shown for selected bond critical points.

The contribution of non-covalent interactions as part of the H–H bond rupture in the transition state (**TS** for **1c** + H_2) was studied using NCIPLOT (Figure S209). Green areas highlight weak van der Waals interactions. The NCIPLOT shows the presence of attractive forces (blue) between the Mg and H atom that forms the hydride ion (as well as electrostatic coordination bonds, e.g. Mg–N, Mg–O), whereas a repulsive force (red) is found between the Mg and the H atom that forms the O–H bond. This study qualitatively agrees with the QTAIM analysis.

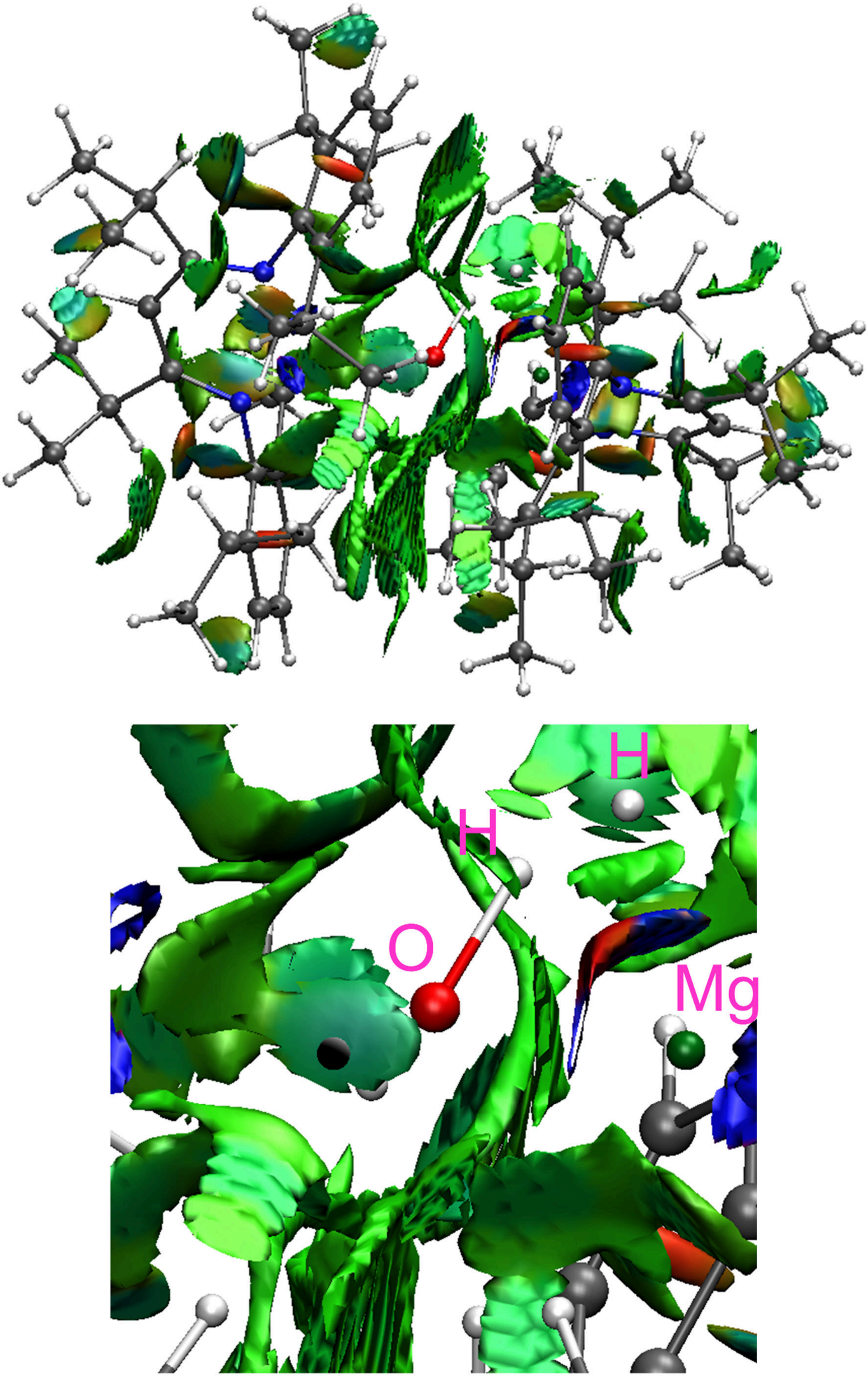

Figure S209. NCIPLOT analysis of the transition state (**TS**) of the reaction of **1c** + H_2 (top: full **TS**, bottom: labelled zoom) showing the isosurface for s = 0.5 au and colour scale $-0.05 < \rho < 0.05$ au.

4.3 The reaction of [{(^{EtDip}nacnac)Mg}$_2$(μ-O)] 1b with H_2 to [{(^{EtDip}NacNac)Mg}$_2$(μ-H)(μ-OH)] 4b

The backbone isopropyl groups in **1c** were replaced with ethyl groups to afford **1b** and the key species were optimised. Some optimisations could be started from atomic coordinates obtained from X-ray diffraction. This led to an energy profile with a slightly higher activation barrier in the transition state (by $\Delta G^{\ddagger}$ (298 K) = 3.6 kcal/mol (solvent)) compared with that of **1c** (see main text and Figure S199). The difference in energy between the co-planar and orthogonal product geometries of **4b** (ca. 5.0 kcal/mol) is not very large and suggests that both are easily accessible at room temperature, Table S5.

Table S5. Relative free energies (kcal/mol) of key intermediates and TS in the reaction between hydrogen and **1b** at the M06-D3$_{PCM(benzene)}$/def2-TZVP//M06-L-D3/def2-SVP level of theory in the gas phase and solvent phase.

**Species**	**Δ*G* in gas phase (kcal/mol)**	**Δ*G* in solvent phase (benzene) (kcal/mol)**
Int-1	6.17	6.36
TS	22.20	21.90
Int-2	11.50	10.79
Product co-planar	-2.57	-2.98
Product orthogonal	-7.99	-7.96

4.4 The reaction of [{(^{iPrDep}nacnac)Mg}$_2$(μ-O)] with H_2 to [{(^{iPrDep}NacNac)Mg}$_2$(μ-H)(μ-OH)]

The effect of reducing the size of the bulk was assessed by studying the system [{(^{iPrDep}nacnac)Mg}$_2$(μ-O)], as derived from geometries of **1c** by replacing the aryl isopropyl groups with ethyl groups. This led to an increase in the activation barrier for this reaction (by $\Delta G^{\ddagger}$ (298 K) = 8.44 kcal/mol (solvent)) compared with **1c** and a stabilised co-planar product geometry, see main text and Table S6.

Table S6. Relative free energies (kcal/mol) of key intermediates and TS in the reaction between [{(^{iPrDep}nacnac)Mg}$_2$(μ-O)] and H_2 at the M06-D3$_{PCM(benzene)}$/def2-TZVP//M06-L-D3/def2-SVP level of theory in the gas phase and solvent phase.

**Species**	**ΔG in gas phase (kcal/mol)**	**ΔG in solvent phase (benzene) (kcal/mol)**
TS	28.35	26.78
Product co-planar	-13.30	-12.85

4.5 The reaction of [{(^{MeMe}nacnac)Mg}$_2$(*μ*-O)] with H_2 to [{(^{MeMe}NacNac)Mg}$_2$(μ-H)(μ-OH)]

To study a sterically unencumbered model, [{(^{MeMe}nacnac)Mg}$_2$(*μ*-O)] was used. We had previously found that [{(^{MeMe}nacnac)Mg}$_2$(*μ*-O)] mimics the overall structure of magnesium oxide complexes well, whereas the overall structure of related sulfide and selenide complexes was not well modelled.[28] Possible oligomerisation of the oxide [{(^{MeMe}nacnac)Mg}$_2$(*μ*-O)] was not considered. The reduction in bulk for the hydrogen activation reaction led to a lower activation barrier for this reaction ($\Delta G^{\ddagger}$(298 K) = 17.18 kcal/mol, compared with **1c**, but also to more stable products (ΔG(298 K) = 12.85 kcal/mol, compared with **1c**. The reaction proceeds via the formation of intermediates **Int-1** and **Int-2**, see Figure S210. It was observed that in the intermediate **Int-1**, one nacnac ligand is essentially free to rotate taking on an either co-planar or orthogonal ligand arrangement, and that both isomers show a very similar energy. The initial product after hydrogen cleavage in the transition state, **Int-3**, shows a *syn*-addition product of hydrogen. This requires facile ionic rearrangement to the *anti*-addition product.

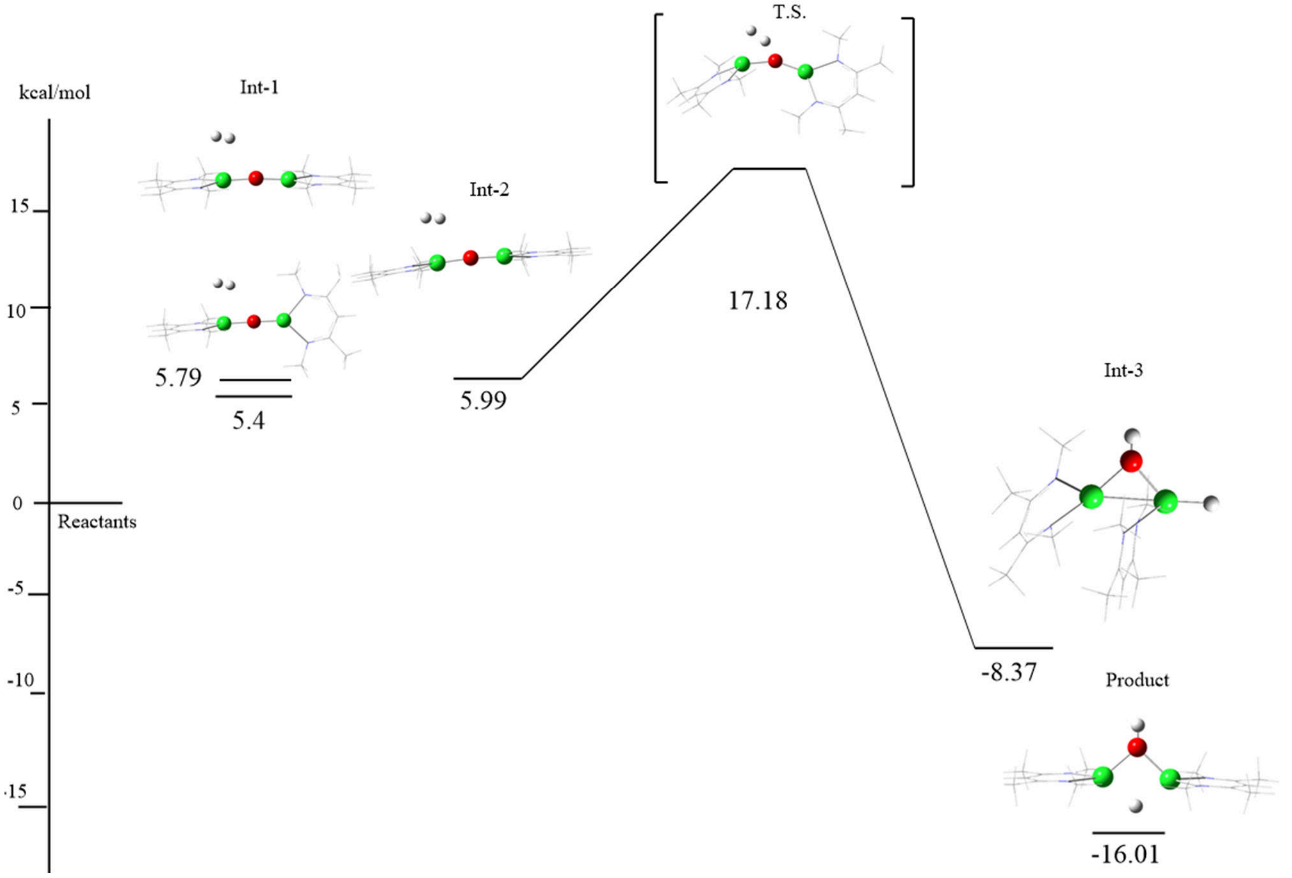

Figure S210. Relative free energies (kcal/mol) of key intermediates and TS in the reaction between hydrogen and [{(^{MeMe}NacNac)Mg}$_2$(*μ*-O)] at the M06-D3$_{PCM(benzene)}$/def2-TZVP//M06-L-D3/def2-SVP level of theory.

Comparison of energies in the gas phase and solvent phase shows that the presence of the solvent (benzene) causes a significant increase in the energy of the system (Table S7).

Table S7. Relative free energies (kcal/mol) of key intermediates and TS in the reaction between [{(^{MeMe}nacnac)Mg}$_2$(μ-O)] and H_2 at the M06-D3$_{PCM(benzene)}$/def2-TZVP//M06-L-D3/def2-SVP level of theory in the gas phase and solvent phase.

**Species**	**Δ*G* in gas phase (kcal/mol)**	**Δ*G* in solvent phase (benzene) (kcal/mol)**
Int-1	4.35	5.40
Int-2	4.86	5.99
TS	16.73	17.18
Int-3	-9.30	-8.37
Product co-planar	-19.31	-16.01

4.6 Atomic coordinates (XYZ) (in Å)

[{(^{iPrDip}nacnac)Mg}$_2$(μ-O)] + H_2 → [{(^{iPrDip}nacnac)Mg}$_2$(μ-H))(μ-OH)]
M06-D3$_{PCM(benzene)}$/def2-TZVP//M06-L-D3/def2-SVP

[{(^{iPrDip}nacnac)Mg}$_2$(μ-O)] **1c**
171
-3267.629445

Mg 1.81840600 -0.00000400 0.00000500
O 0.00000000 0.00000300 0.00000600
N 3.20117900 1.40476000 0.52936400
C 5.09821300 0.00000500 0.00000400
H 6.18791700 0.00000700 0.00000400
C 2.69265000 2.65211400 0.98351000
C 2.34398800 3.63766000 0.03327800
C 4.51724300 1.17719000 0.50563500
C 5.49454100 2.24376100 0.99639800
H 4.91179400 2.98898800 1.55947800
C 2.50770100 2.88091300 2.36621000
C 1.66070100 5.09868000 1.85236100
H 1.26334200 6.05853400 2.19229700
C 2.81160100 1.78087300 3.36300800
H 3.60607800 1.15072200 2.92901900
C 1.82075500 4.85158000 0.49243200
H 1.54269100 5.62310900 -0.23030600
C 2.00194200 4.11656000 2.77882800
H 1.86268700 4.31218300 3.84540300
C 2.50633300 3.36213800 -1.44860400
H 3.34641100 2.65384300 -1.55826400
C 6.55028100 1.69562300 1.94867900
H 7.27697700 1.04187300 1.44427700
H 6.10307200 1.11632400 2.76964100
H 7.12269000 2.52017000 2.39835000
C 6.13074400 2.96979800 -0.18465300
H 6.80338000 3.76811900 0.16149300
H 5.37426800 3.43380700 -0.83310600
H 6.72248100 2.28090500 -0.80707300
C 1.25893200 2.68218200 -2.00768400
H 0.93725200 1.79485600 -1.43350100
H 1.40686800 2.37772300 -3.05524300
H 0.39961700 3.36817900 -1.97677300
C 1.58280500 0.89100300 3.54250000
H 0.76091500 1.45196400 4.01491300
H 1.80136000 0.01961700 4.17834200
H 1.18466900 0.51683700 2.58221000
C 3.32389800 2.28913800 4.70144300
H 4.19039600 2.95608000 4.57976700
H 3.63459100 1.45158800 5.34245100
H 2.55501300 2.84452000 5.25943500
C 2.84240300 4.60134400 -2.26558500
H 1.99627400 5.30378000 -2.31422400
H 3.08100500 4.32711100 -3.30319700
H 3.70411500 5.14926100 -1.85676300
Mg -1.81840600 0.00000200 0.00000200
N -3.20118300 1.40476000 -0.52935600
C -5.09821400 -0.00000400 -0.00000600
H -6.18791800 -0.00000500 -0.00000800
C -2.69265600 2.65211700 -0.98349900
C -2.34399500 3.63766000 -0.03326400
C -4.51724600 1.17718500 -0.50563300
C -5.49454600 2.24375500 -0.99639700
H -4.91179900 2.98897900 -1.55948000
C -2.50770700 2.88091900 -2.36619900
C -1.66071000 5.09868500 -1.85234400
H -1.26335300 6.05854000 -2.19227800
C -2.81160500 1.78088000 -3.36299900
H -3.60608100 1.15072700 -2.92901100
C -1.82076400 4.85158300 -0.49241600
H -1.54270000 5.62311000 0.23032400
C -2.00195000 4.11656700 -2.77881400
H -1.86269400 4.31219300 -3.84538800
C -2.50633900 3.36213400 1.44861700
H -3.34641800 2.65383900 1.55827600
C -6.55028800 1.69561600 -1.94867300
H -7.27698600 1.04187000 -1.44426700
H -6.10308200 1.11631200 -2.76963400

H	-7.12269600	2.52016200	-2.39834700
C	-6.13074400	2.96979600	0.18465400
H	-6.80338300	3.76811500	-0.16149200
H	-5.37426600	3.43380800	0.83310200
H	-6.72247800	2.28090500	0.80707900
C	-1.25893900	2.68217500	2.00769400
H	-0.93725600	1.79485400	1.43350700
H	-1.40687600	2.37770900	3.05525100
H	-0.39962400	3.36817300	1.97679000
C	-1.58280700	0.89101200	-3.54249100
H	-0.76091800	1.45197400	-4.01490200
H	-1.80136100	0.01962600	-4.17833400
H	-1.18467200	0.51684500	-2.58220100
C	-3.32390200	2.28914600	-4.70143300
H	-4.19040100	2.95608600	-4.57975700
H	-3.63459300	1.45159600	-5.34244200
H	-2.55501800	2.84453100	-5.25942300
C	-2.84240700	4.60133800	2.26560200
H	-1.99627800	5.30377400	2.31424100
H	-3.08100700	4.32710300	3.30321300
H	-3.70411900	5.14925600	1.85678300
N	3.20118500	-1.40475800	-0.52935600
C	2.69265900	-2.65211400	-0.98350400
C	2.34399500	-3.63766000	-0.03327200
C	4.51724800	-1.17718300	-0.50562800
C	5.49455000	-2.24375100	-0.99639100
H	4.91180600	-2.98897600	-1.55947600
C	2.50771200	-2.88091200	-2.36620500
C	1.66071400	-5.09867900	-1.85235800
H	1.26335700	-6.05853300	-2.19229500
C	2.81161200	-1.78086900	-3.36300100
H	3.60608700	-1.15071800	-2.92901000
C	1.82076500	-4.85158100	-0.49242900
H	1.54269900	-5.62311000	0.23030800
C	2.00195600	-4.11655800	-2.77882400
H	1.86270200	-4.31218200	-3.84539900
C	2.50633700	-3.36213800	1.44860900
H	3.34641400	-2.65384200	1.55827200
C	6.55029200	-1.69560900	-1.94866600
H	7.27698700	-1.04186100	-1.44425900
H	6.10308600	-1.11630600	-2.76962700
H	7.12270300	-2.52015300	-2.39833900
C	6.13074900	-2.96979200	0.18466000
H	6.80338800	-3.76811000	-0.16148700
H	5.37427100	-3.43380500	0.83310700
H	6.72248200	-2.28090100	0.80708500
C	1.25893400	-2.68218200	2.00768600
H	0.93725200	-1.79485900	1.43350200
H	1.40686900	-2.37772000	3.05524400
H	0.39962000	-3.36818100	1.97677700
C	1.58281500	-0.89100100	-3.54249400
H	0.76092600	-1.45196300	-4.01490800
H	1.80137000	-0.01961400	-4.17833500
H	1.18467800	-0.51683600	-2.58220400
C	3.32391200	-2.28913300	-4.70143600
H	4.19041100	-2.95607300	-4.57975900
H	3.63460500	-1.45158100	-5.34244200
H	2.55502900	-2.84451500	-5.25942900
C	2.84240500	-4.60134300	2.26559100
H	1.99627700	-5.30378100	2.31422700
H	3.08100300	-4.32711000	3.30320400
H	3.70412000	-5.14925800	1.85677200
N	-3.20118200	-1.40476300	0.52935100
C	-2.69265400	-2.65211900	0.98349400
C	-2.34398800	-3.63766100	0.03325900
C	-4.51724500	-1.17719100	0.50562200
C	-5.49454500	-2.24376300	0.99638000
H	-4.91179900	-2.98898800	1.55946400
C	-2.50770900	-2.88092300	2.36619400
C	-1.66070600	-5.09868600	1.85233900
H	-1.26334700	-6.05854100	2.19227400
C	-2.81161200	-1.78088500	3.36299500
H	-3.60608900	-1.15073400	2.92900600
C	-1.82075600	-4.85158200	0.49241100
H	-1.54268800	-5.62310800	-0.23032900
C	-2.00195100	-4.11657000	2.77881000
H	-1.86269700	-4.31219700	3.84538400
C	-2.50632800	-3.36213400	-1.44862100
H	-3.34640700	-2.65384000	-1.55828300

C -6.55029100 -1.69562800 1.94865500
H -7.27698600 -1.04188000 1.44424800
H -6.10308800 -1.11632700 2.76961900
H -7.12270000 -2.52017600 2.39832300
C -6.13073800 -2.96980300 -0.18467400
H -6.80337600 -3.76812400 0.16146900
H -5.37425800 -3.43381100 -0.83312100
H -6.72247300 -2.28091100 -0.80709800
C -1.25892700 -2.68217300 -2.00769400
H -0.93725000 -1.79484800 -1.43350700
H -1.40686000 -2.37771000 -3.05525200
H -0.39961000 -3.36816800 -1.97678300
C -1.58281700 -0.89101500 3.54249200
H -0.76092700 -1.45197700 4.01490400
H -1.80137400 -0.01963000 4.17833600
H -1.18468000 -0.51684600 2.58220300
C -3.32391200 -2.28915500 4.70142700
H -4.19040900 -2.95609700 4.57974800
H -3.63460600 -1.45160700 5.34243700
H -2.55502800 -2.84453800 5.25941900
C -2.84239100 -4.60133700 -2.26560800
H -1.99626100 -5.30377100 -2.31424700
H -3.08099000 -4.32710100 -3.30322100
H -3.70410400 -5.14925800 -1.85679200

H_2
2
-1.170703414

H 0.00000000 0.00000000 0.37726500
H 0.00000000 0.00000000 -0.37726500

Int-1
173
-3268.806367

Mg -1.82538700 0.03798100 0.00772300
O -0.01034500 -0.00368700 -0.09328600
N -3.19238700 1.45046400 -0.53680100
C -5.08043100 -0.04084600 -0.26850100
H -6.16181800 -0.08193200 -0.38596800
C -2.68718400 2.71484300 -0.94584900
C -2.31632800 3.66471400 0.03071700
C -4.50240600 1.19120100 -0.62082800
C -5.46691400 2.29104500 -1.06121600
H -4.89497300 2.99399200 -1.68628300
C -2.52711300 2.99651200 -2.32473000
C -1.71855900 5.21350000 -1.74592700
H -1.35385600 6.19519600 -2.05856300
C -2.82444800 1.92512900 -3.35599500
H -3.64484200 1.30112700 -2.96371800
C -1.83079900 4.90758100 -0.39401100
H -1.54290100 5.65279500 0.35232500
C -2.05574500 4.25660800 -2.70086800
H -1.94263200 4.49456400 -3.76161600
C -2.38502700 3.32281700 1.50588800
H -3.17094400 2.55594000 1.63178500
C -6.63900000 1.80967000 -1.90446700
H -7.38670000 1.25399300 -1.31936800
H -6.31453500 1.15892500 -2.72967000
H -7.16389200 2.66933300 -2.34572800
C -5.94157300 3.06974300 0.16258000
H -6.60880400 3.89367900 -0.12945300
H -5.09908600 3.50709700 0.71661700
H -6.49507300 2.41804900 0.85629100
C -1.05782600 2.71402800 1.95367300
H -0.74414600 1.83950600 1.35453200
H -1.08939300 2.41205900 3.01169500
H -0.24568500 3.44715300 1.84320700
C -1.61069500 1.01168700 -3.52488500
H -0.76901200 1.56363100 -3.97196500
H -1.83595800 0.15835100 -4.18235000
H -1.23871400 0.61045400 -2.56558800
C -3.28771900 2.47297100 -4.69657900
H -4.14305300 3.15633500 -4.58598100
H -3.59754000 1.65564000 -5.36334200
H -2.49130700 3.02245400 -5.22090800
C -2.74910200 4.50594300 2.39162900
H -1.94375400 5.25568200 2.42611500

H	-2.91415000	4.17704500	3.42761600
H	-3.66248600	5.01652900	2.05266300
Mg	1.80805000	0.00439800	-0.11956600
N	3.18947800	1.40289200	0.44972700
C	5.04445100	0.04638400	-0.32346300
H	6.12666900	0.08156200	-0.44250000
C	2.72838600	2.60410100	1.05875500
C	2.36114000	3.70800700	0.25530400
C	4.48971100	1.22953100	0.20652300
C	5.49293400	2.34416800	0.50170000
H	4.91968900	3.23386000	0.80372700
C	2.63108900	2.67256600	2.46677600
C	1.85480300	4.96747900	2.27177300
H	1.52335900	5.89448700	2.74631700
C	2.93067000	1.45314700	3.31537400
H	3.67855700	0.84637200	2.77635200
C	1.92480700	4.87840200	0.88423900
H	1.64668100	5.74252100	0.27567000
C	2.19922500	3.86776400	3.05145300
H	2.12940400	3.93840400	4.13999000
C	2.44561100	3.60592600	-1.25283100
H	3.31177700	2.96164500	-1.47982900
C	6.40272800	1.97489300	1.66814800
H	7.02809200	1.09987500	1.43396800
H	5.82913500	1.73468200	2.57451800
H	7.07813800	2.80691300	1.91542500
C	6.29972700	2.73186700	-0.73298900
H	6.90902200	3.62449400	-0.52882800
H	5.65200100	2.96369400	-1.59132200
H	6.98964000	1.93557200	-1.04984200
C	1.20356600	2.92184600	-1.81790700
H	0.96917200	1.95138900	-1.34591700
H	1.29485300	2.75812700	-2.90374200
H	0.31201100	3.54329800	-1.65074000
C	1.67210400	0.59889100	3.44902800
H	0.89019600	1.14095200	4.00467100
H	1.87608200	-0.33818300	3.98910800
H	1.22791000	0.33557800	2.47245700
C	3.50597200	1.78075600	4.68499300
H	4.37929100	2.44579100	4.61854300
H	3.82187200	0.86357700	5.20233700
H	2.76858900	2.27147900	5.33804500
C	2.67905000	4.93877400	-1.94712600
H	1.80254600	5.60091200	-1.87478900
H	2.86858100	4.78623300	-3.01934200
H	3.54150700	5.47854300	-1.52825800
N	-3.21345900	-1.37763900	0.50372100
C	-2.73873300	-2.59269400	1.07071700
C	-2.40074500	-3.67633700	0.22873700
C	-4.51416900	-1.22314200	0.24588200
C	-5.50266800	-2.35341200	0.53181600
H	-4.91647900	-3.25405200	0.77000200
C	-2.59882600	-2.69275200	2.47284500
C	-1.82781900	-4.98050000	2.19792400
H	-1.47991000	-5.91787700	2.63933900
C	-2.87848600	-1.49112400	3.35354700
H	-3.65030000	-0.88423100	2.84926500
C	-1.94110700	-4.85935000	0.81538400
H	-1.67989500	-5.70843900	0.17867600
C	-2.15087400	-3.90166100	3.01543000
H	-2.04814900	-3.99949800	4.09931500
C	-2.54170300	-3.53687100	-1.27297200
H	-3.40139700	-2.86905700	-1.45558300
C	-6.35255500	-2.03120700	1.75655400
H	-6.97671600	-1.13981800	1.59044600
H	-5.73441600	-1.83888500	2.64482000
H	-7.02517200	-2.86742200	1.99721700
C	-6.37255800	-2.69522600	-0.67250900
H	-6.96365900	-3.60068500	-0.47137000
H	-5.77189200	-2.88631600	-1.57356700
H	-7.08578300	-1.89459000	-0.91838600
C	-1.30874900	-2.86345900	-1.87024700
H	-1.03627900	-1.91512900	-1.37507100
H	-1.44889800	-2.66080200	-2.94318400
H	-0.42555300	-3.51080600	-1.76388700
C	-1.62682300	-0.62279700	3.46331200
H	-0.83051600	-1.15227300	4.01010300
H	-1.83088500	0.31680900	3.99901400
H	-1.19266900	-0.36433600	2.48046400

C -3.40465400 -1.84783400 4.73539200
H -4.27738800 -2.51497900 4.68561100
H -3.70562300 -0.94265500 5.28195000
H -2.64315100 -2.34859100 5.35210000
C -2.83365300 -4.84921800 -1.98383300
H -1.97330900 -5.53532100 -1.95355000
H -3.05573500 -4.67191900 -3.04592600
H -3.69485800 -5.37504900 -1.54480000
N 3.15917400 -1.44983200 -0.59064600
C 2.65423700 -2.73573800 -0.93241600
C 2.31483900 -3.64111000 0.09621000
C 4.46689600 -1.19144200 -0.66767100
C 5.43860100 -2.28684700 -1.10037700
H 4.85307300 -3.04636500 -1.64089900
C 2.45371900 -3.08245000 -2.29044400
C 1.64491900 -5.26295400 -1.58758700
H 1.26014200 -6.25354000 -1.84305300
C 2.71528300 -2.06554300 -3.38245700
H 3.51947600 -1.39897200 -3.03017300
C 1.80543200 -4.89676300 -0.25612100
H 1.53356400 -5.60308100 0.53296200
C 1.96307700 -4.35470900 -2.59420200
H 1.81527700 -4.63778200 -3.63964300
C 2.41871800 -3.23975500 1.55355900
H 3.17227900 -2.43481300 1.62810800
C 6.52808600 -1.81173900 -2.05220000
H 7.28316900 -1.18286800 -1.55780400
H 6.11672600 -1.23339500 -2.89289200
H 7.06497700 -2.67373600 -2.47421100
C 6.02407700 -2.96807600 0.13273500
H 6.68910200 -3.79647500 -0.15177900
H 5.23583400 -3.38219800 0.77759500
H 6.60966100 -2.25891200 0.73820200
C 1.07558400 -2.67670300 2.01207600
H 0.72794100 -1.82383900 1.40080400
H 1.11081600 -2.35643300 3.06441000
H 0.29402800 -3.44545400 1.92576800
C 1.47305100 -1.20084600 -3.59181400
H 0.64167500 -1.80568900 -3.98660300
H 1.66097200 -0.38672300 -4.30929100
H 1.10315700 -0.74656400 -2.65427800
C 3.18616500 -2.67512500 -4.69388800
H 4.05648800 -3.33268100 -4.54977400
H 3.47654100 -1.88824500 -5.40481800
H 2.40119800 -3.26924100 -5.18562800
C 2.86291500 -4.37223600 2.46908900
H 2.09452400 -5.15561600 2.55639200
H 3.04437300 -3.99783800 3.48683700
H 3.78761300 -4.85475700 2.11991400
H 2.94458500 0.96860900 -2.69193100
H 3.62357900 0.81076700 -2.38028800

Int-2
173
-3268.797296

Mg -1.86527500 -0.16982700 -0.08167300
O -0.05519200 0.01309400 -0.15015100
N -2.99125900 -1.77541200 0.48958200
C -5.11000500 -0.67749900 0.12935600
H -6.18536900 -0.84485000 0.19524000
C -2.23042100 -2.91520800 0.86456400
C -1.80076100 -3.81359100 -0.13865100
C -4.32258900 -1.76555300 0.55039700
C -5.08288200 -2.98187200 1.07276800
H -4.33637700 -3.68459400 1.47630500
C -1.83893900 -3.09663200 2.20942300
C -0.53284800 -5.03164900 1.53797600
H 0.14207100 -5.85087900 1.79852700
C -2.29481600 -2.12993900 3.28253200
H -3.20596000 -1.63012900 2.91381200
C -0.94497900 -4.85877000 0.22084400
H -0.59274600 -5.55256100 -0.54642800
C -0.98652200 -4.15960800 2.52223400
H -0.66633900 -4.30394300 3.55830900
C -2.23031900 -3.61654800 -1.57816900
H -3.20271400 -3.09511100 -1.56129700
C -6.03107000 -2.62581900 2.21245100

H -6.87613600 -2.00853500 1.87327900
H -5.52307600 -2.07087100 3.01434200
H -6.45597900 -3.53714000 2.65837200
C -5.81939100 -3.69831900 -0.05418400
H -6.32056300 -4.60294800 0.32001600
H -5.13787900 -4.00788200 -0.85889800
H -6.59064200 -3.05521600 -0.50506200
C -1.24439500 -2.71455000 -2.31131400
H -1.04372800 -1.76611800 -1.78422000
H -1.59335800 -2.47344500 -3.32735800
H -0.26184400 -3.20429000 -2.39465500
C -1.24191300 -1.04629100 3.48720500
H -0.29916800 -1.48126600 3.85723100
H -1.57133400 -0.28960100 4.21582300
H -0.99659600 -0.52378000 2.54605200
C -2.65257900 -2.81090300 4.59562700
H -3.38506500 -3.61864300 4.45051800
H -3.08586500 -2.08955400 5.30369000
H -1.77315000 -3.25006100 5.09023100
C -2.42046900 -4.92173600 -2.33753300
H -1.46404500 -5.43877200 -2.51119100
H -2.85996700 -4.73415300 -3.32783100
H -3.08110900 -5.62018900 -1.80339600
Mg 1.76508900 0.10195400 0.04698900
N 3.40344700 -1.03906400 -0.46723400
C 4.95432300 0.81233600 -0.30783200
H 6.00353800 1.06049400 -0.46716100
C 3.10215400 -2.36021800 -0.90485900
C 3.11294400 -3.45438500 -0.00503200
C 4.63355500 -0.53113300 -0.56906200
C 5.80811900 -1.40306400 -1.00579000
H 5.43769100 -2.43850600 -1.07604800
C 2.70250900 -2.54440000 -2.25272500
C 2.24758400 -4.88458700 -1.77408600
H 1.90464500 -5.86686900 -2.10945900
C 2.71673600 -1.39202400 -3.24283900
H 3.40409500 -0.62591800 -2.84777100
C 2.66903000 -4.69982800 -0.46220300
H 2.66744600 -5.54939200 0.22783900
C 2.28195500 -3.81372100 -2.66143100
H 1.96729500 -3.96400000 -3.69814300
C 3.69116600 -3.34514500 1.39583500
H 3.83213100 -2.27086600 1.61537800
C 6.32664300 -1.01121000 -2.38533300
H 6.72369600 0.01534200 -2.38955300
H 5.54398800 -1.06296200 -3.15383900
H 7.14139800 -1.68058000 -2.69829300
C 6.93743500 -1.37763100 0.02063900
H 7.68277500 -2.15556200 -0.20303100
H 6.57280000 -1.54758400 1.04362600
H 7.46906900 -0.41459700 0.02148000
C 2.80489400 -3.93854400 2.48683700
H 1.79243800 -3.51434800 2.50105900
H 3.25387600 -3.77342500 3.47755900
H 2.69405700 -5.02756200 2.36826500
C 1.34546800 -0.73226500 -3.37022600
H 0.62880400 -1.41621400 -3.85153900
H 1.40197700 0.17412100 -3.99230000
H 0.90295800 -0.44985200 -2.39791700
C 3.23780300 -1.80037300 -4.61542200
H 4.20161700 -2.32692100 -4.55580600
H 3.37604900 -0.91723100 -5.25609200
H 2.53636900 -2.46433500 -5.14285600
C 5.05910500 -4.02771700 1.45037900
H 4.95114100 -5.11590900 1.32073000
H 5.55158200 -3.86056300 2.42025800
H 5.74022200 -3.67864500 0.66422900
N -3.48090500 1.02172500 -0.43893500
C -3.19485700 2.35613700 -0.83798900
C -2.92815300 3.32604700 0.15400700
C -4.74456400 0.59611700 -0.34349800
C -5.89948400 1.52692700 -0.70821200
H -5.47238100 2.38096700 -1.25625200
C -3.13345700 2.68833700 -2.21061600
C -2.58385800 4.97400300 -1.60021700
H -2.35184200 5.99975100 -1.89784200
C -3.33220000 1.61096700 -3.25695900
H -3.99467800 0.84460300 -2.82075300
C -2.61774500 4.62861400 -0.25262600

H -2.40596300 5.38970200 0.50298000
C -2.83746700 4.00624200 -2.56896000
H -2.79493100 4.28006200 -3.62647100
C -2.93116100 2.94167700 1.62055000
H -3.64091200 2.10405600 1.73984100
C -6.92245100 0.87594200 -1.63090400
H -7.50572500 0.08981700 -1.12922000
H -6.44913800 0.42033000 -2.51290500
H -7.64233800 1.62539100 -1.99123400
C -6.55590500 2.08370000 0.55101200
H -7.35862100 2.79083400 0.29566800
H -5.83401800 2.61855600 1.18431900
H -6.99920400 1.28110400 1.16047900
C -1.55000800 2.43992200 2.03552700
H -1.14768600 1.64422400 1.38288800
H -1.55160700 2.06496800 3.07066400
H -0.81374000 3.25383300 1.97626500
C -1.99664100 0.93258400 -3.55775400
H -1.30647200 1.63497100 -4.05063900
H -2.12078900 0.06371100 -4.22162100
H -1.47764300 0.58815700 -2.64511100
C -3.99801900 2.10622200 -4.53112200
H -4.94667700 2.62225400 -4.32096700
H -4.21694700 1.26737400 -5.20738000
H -3.35890800 2.80467900 -5.09203900
C -3.38445800 4.06241400 2.54485100
H -2.65389400 4.88489400 2.58275400
H -3.49348500 3.69357600 3.57480100
H -4.34988500 4.49089600 2.23794800
N 2.86989100 1.79391300 0.43577500
C 2.20488100 2.91559500 1.00549800
C 1.65045200 3.91734400 0.17812900
C 4.16821600 1.89061100 0.13517300
C 4.91197700 3.21638300 0.30096400
H 4.15913800 4.00317900 0.45910200
C 2.12362200 3.01148300 2.41396900
C 0.97353400 5.13653600 2.17044800
H 0.50119700 6.01112200 2.62503900
C 2.64646600 1.87819700 3.27503400
H 3.51597500 1.44207800 2.75359400
C 1.03060400 5.01506100 0.78469600
H 0.60085600 5.80115700 0.15850200
C 1.51371900 4.13747900 2.97570500
H 1.45681200 4.23492400 4.06286400
C 1.75069500 3.79970600 -1.32874700
H 2.69295000 3.26721300 -1.54715500
C 5.79818900 3.18674000 1.54175600
H 6.57300800 2.40830800 1.46388000
H 5.21787000 2.98482600 2.45313700
H 6.30759300 4.15122900 1.68286700
C 5.70537300 3.60913000 -0.94004300
H 6.08815200 4.63523300 -0.83807300
H 5.08884600 3.57501000 -1.85003900
H 6.57668700 2.95939200 -1.10967900
C 0.61003600 2.94951700 -1.88112100
H 0.53360000 1.95813200 -1.40328700
H 0.71804900 2.80320200 -2.96718200
H -0.35683000 3.44495000 -1.70573200
C 1.58979600 0.77966500 3.37899800
H 0.75258900 1.10226900 4.01805500
H 1.99862700 -0.14971400 3.80645600
H 1.13028000 0.53389900 2.40543600
C 3.10960700 2.30664100 4.65813500
H 3.84582800 3.12220500 4.61331900
H 3.57570800 1.46466500 5.18960000
H 2.27442600 2.65153800 5.28628100
C 1.81764600 5.14308700 -2.03937400
H 0.86241400 5.68748800 -1.98149600
H 2.03334700 5.00304700 -3.10839500
H 2.59980600 5.79586300 -1.62258700
H 0.74246300 -1.62235700 1.13863000
H 1.37348400 -1.88334000 1.49964400

Transition State
173
-3268.782122

Mg -1.87307400 0.06419700 0.06216000
O -0.04485400 0.24013600 -0.21369700

N	-3.33101100	1.36455200	-0.49618800
C	-5.09796000	-0.27595000	-0.32607100
H	-6.16850300	-0.40135200	-0.47436800
C	-2.90008300	2.66326600	-0.88525000
C	-2.55788000	3.60762400	0.10549700
C	-4.61584700	1.01445200	-0.60883800
C	-5.65499900	2.06412100	-0.99532600
H	-5.13637900	2.83254100	-1.58924900
C	-2.76858800	2.97487200	-2.26170100
C	-2.01925700	5.20276300	-1.64437400
H	-1.67957600	6.19772400	-1.94256400
C	-3.06792200	1.92508100	-3.31679700
H	-3.91742500	1.32040300	-2.95607000
C	-2.11270500	4.87183900	-0.29883800
H	-1.83785900	5.61072200	0.45877900
C	-2.33767400	4.25541900	-2.61447900
H	-2.23994200	4.51899300	-3.67033800
C	-2.61094100	3.24755100	1.57716100
H	-3.32797800	2.41376000	1.69049900
C	-6.80366300	1.54597800	-1.84800900
H	-7.50386000	0.91444700	-1.28127300
H	-6.44957400	0.95850700	-2.70772400
H	-7.39091100	2.38825700	-2.24108400
C	-6.16414300	2.75034000	0.26971900
H	-6.88356400	3.54476500	0.02395500
H	-5.34398500	3.21075700	0.83828900
H	-6.66949800	2.03226300	0.93394000
C	-1.24005700	2.75763900	2.03715900
H	-0.84725700	1.91713700	1.43715900
H	-1.25374800	2.45044600	3.09388100
H	-0.48955300	3.55510000	1.93282300
C	-1.88764800	0.97029000	-3.48142500
H	-0.98672100	1.49809000	-3.82961300
H	-2.11334700	0.17262800	-4.20489500
H	-1.60699900	0.48163600	-2.53496900
C	-3.48326000	2.50786200	-4.65847900
H	-4.31419200	3.22183600	-4.55648300
H	-3.80951700	1.71063200	-5.34127100
H	-2.65515000	3.03186300	-5.15900600
C	-3.09071200	4.38695600	2.46584800
H	-2.36115100	5.20984200	2.50760700
H	-3.23141900	4.04005700	3.49967400
H	-4.04539200	4.81026700	2.12131900
Mg	1.86926200	0.14741000	-0.36389100
N	3.20656400	1.50603100	0.38735700
C	5.10450300	0.10108700	-0.15054900
H	6.19250700	0.12350200	-0.18223700
C	2.69689500	2.73139100	0.89532600
C	2.29116100	3.75555800	0.01278400
C	4.51987600	1.31613900	0.25644100
C	5.50307000	2.45154100	0.53904700
H	4.91112700	3.36954800	0.68203100
C	2.55256800	2.89118700	2.29567300
C	1.66805300	5.12509800	1.92412500
H	1.27641000	6.06309300	2.32617300
C	2.85901300	1.73872500	3.23157100
H	3.64891900	1.12674000	2.76470900
C	1.77318700	4.94034400	0.55088600
H	1.45813600	5.73871100	-0.12878500
C	2.05230100	4.09984500	2.78616200
H	1.94799700	4.23961400	3.86543500
C	2.42816700	3.61830300	-1.48977100
H	2.83611000	2.61581500	-1.70307500
C	6.29205800	2.21240900	1.82132600
H	6.90083900	1.29768600	1.75345800
H	5.63686300	2.10739300	2.69690100
H	6.97605000	3.05048500	2.02040500
C	6.43872000	2.69706100	-0.64091000
H	6.99050300	3.63901100	-0.50445300
H	5.89215100	2.76378500	-1.59240100
H	7.18785300	1.89921700	-0.75233700
C	1.07398600	3.71930500	-2.17974700
H	0.33973300	3.02404400	-1.74485500
H	1.15730700	3.48643900	-3.25223400
H	0.65751900	4.73433200	-2.09406900
C	1.62804400	0.84523800	3.36285000
H	0.80708400	1.38453100	3.86192500
H	1.84571100	-0.05649500	3.95575600
H	1.24254200	0.51946300	2.38202000

C	3.35826700	2.16970600	4.60221000
H	4.20842400	2.86402800	4.53392000
H	3.68388300	1.29841300	5.18850500
H	2.57433200	2.66939000	5.19126900
C	3.40582900	4.64579200	-2.05293700
H	3.02562700	5.67128900	-1.92407000
H	3.56656300	4.49199200	-3.13035100
H	4.38657200	4.60094300	-1.55775400
N	-3.14528400	-1.47919600	0.46463600
C	-2.61182600	-2.66361200	1.04635400
C	-2.18199800	-3.72821300	0.22173300
C	-4.44645100	-1.42586300	0.15501200
C	-5.33824200	-2.65027600	0.36082500
H	-4.67851300	-3.52136100	0.49256800
C	-2.51850000	-2.74930100	2.45358800
C	-1.60929900	-4.98891600	2.22040700
H	-1.22597700	-5.90379100	2.67922600
C	-2.89712400	-1.55785800	3.31180300
H	-3.70295600	-1.01467900	2.78821800
C	-1.67804200	-4.88040700	0.83346200
H	-1.34639000	-5.71656600	0.21315500
C	-2.02341700	-3.92836000	3.02056800
H	-1.95571200	-4.01738600	4.10777900
C	-2.29232400	-3.60782700	-1.28521100
H	-3.21890700	-3.04410200	-1.49448100
C	-6.15544200	-2.51011800	1.64118100
H	-6.83058300	-1.64190600	1.59275400
H	-5.51330600	-2.38195600	2.52386600
H	-6.77323500	-3.40394300	1.81074100
C	-6.22999100	-2.95180800	-0.83766600
H	-6.71693100	-3.92964800	-0.71066000
H	-5.65947200	-2.98507800	-1.77715400
H	-7.03325900	-2.21183300	-0.96788700
C	-1.13118300	-2.79963600	-1.85739900
H	-1.02362500	-1.80431500	-1.39816100
H	-1.24263200	-2.65193200	-2.94212900
H	-0.17451500	-3.31451300	-1.68601800
C	-1.71507200	-0.59803400	3.43158000
H	-0.89918300	-1.04886800	4.01768700
H	-2.00258000	0.34181200	3.92610200
H	-1.26157000	-0.33756200	2.45679800
C	-3.41574700	-1.92908300	4.69248000
H	-4.24180100	-2.65287700	4.64128800
H	-3.78362000	-1.03880700	5.22192400
H	-2.63099800	-2.37043300	5.32499600
C	-2.40869200	-4.94624800	-1.99726300
H	-1.47236600	-5.52241200	-1.94322400
H	-2.62365100	-4.79656700	-3.06474100
H	-3.21208000	-5.57043300	-1.57809700
N	3.22741000	-1.38250700	-0.53030400
C	2.74155000	-2.67363600	-0.87215800
C	2.33606500	-3.54684200	0.16565300
C	4.54112700	-1.14227000	-0.49138300
C	5.53107700	-2.26807100	-0.78397200
H	4.99427300	-3.02427400	-1.37743100
C	2.62153200	-3.06855600	-2.22545400
C	1.76232300	-5.22572200	-1.49653700
H	1.39312400	-6.22551200	-1.73973300
C	2.99087500	-2.14900900	-3.37411200
H	3.39249800	-1.21512100	-2.94685200
C	1.84909600	-4.81400700	-0.16926200
H	1.53598400	-5.49471700	0.62679300
C	2.13905300	-4.35145400	-2.50977400
H	2.05233500	-4.66656500	-3.55437000
C	2.35923300	-3.08830900	1.60989900
H	3.15493100	-2.32897700	1.71040500
C	6.74012900	-1.83771400	-1.60405100
H	7.45863900	-1.24241500	-1.02140800
H	6.45240500	-1.24058900	-2.48159000
H	7.28607500	-2.72144700	-1.96591000
C	5.95510500	-2.94195000	0.51766000
H	6.65051400	-3.77143100	0.32242500
H	5.09448600	-3.35463200	1.06238100
H	6.46199500	-2.22870700	1.18607500
C	1.03097400	-2.41666200	1.94283600
H	0.80764500	-1.55495300	1.29169200
H	1.00509100	-2.06673000	2.98562100
H	0.20355700	-3.12679800	1.80627700
C	1.75565400	-1.77862200	-4.19078600

H 1.29675300 -2.66930500 -4.64941100
H 2.01465000 -1.08433900 -5.00392200
H 0.99450600 -1.28570600 -3.56904100
C 4.06969300 -2.75549800 -4.26659600
H 4.96706300 -3.04246300 -3.69969300
H 4.38213400 -2.04453100 -5.04565200
H 3.70894600 -3.65931800 -4.78154000
C 2.64923300 -4.20269300 2.60511400
H 1.81700700 -4.92004500 2.67335800
H 2.79072700 -3.79095600 3.61473600
H 3.55488200 -4.77014200 2.34513100
H 0.36326300 0.47974100 -1.36876600
H 1.09355800 0.58876000 -2.10009200

Int-3
173
-3268.801266

Mg 1.70560800 -0.07403000 -0.12261400
O -0.10927100 0.48620100 0.08320600
N 2.81163000 -1.63626000 0.57783800
C 3.84352100 -1.40434200 1.40334600
C 4.45491000 -0.14893900 1.56225100
H 5.32123300 -0.15898300 2.22126400
C 4.27347200 1.05168700 0.84796600
N 3.20237500 1.29714000 0.09080600
C 2.51717200 -2.97309900 0.18857200
C 2.97160100 -3.40607300 -1.08008400
C 2.71048200 -4.72737900 -1.46054600
H 3.05789900 -5.08967900 -2.43046600
C 1.97731300 -5.57984300 -0.64110000
H 1.77825900 -6.60615500 -0.95881300
C 1.46753900 -5.11282600 0.56665600
H 0.85575300 -5.77554900 1.18297200
C 1.72569800 -3.81048700 1.00424700
C 3.58568200 -2.41645600 -2.05938600
H 2.99096100 -1.48434500 -1.94847700
C 5.03344500 -2.03499200 -1.76522600
H 5.15486900 -1.56065400 -0.78235200
H 5.40838900 -1.32333800 -2.51762900
H 5.68916100 -2.91889800 -1.80053200
C 3.43946700 -2.84234200 -3.51290800
H 4.08046900 -3.70309700 -3.75580700
H 3.74459900 -2.02579000 -4.18371300
H 2.40448500 -3.11618700 -3.76628800
C 1.14176400 -3.28453800 2.30028600
H 1.85605800 -2.54935400 2.70733900
C 0.94495300 -4.35884700 3.35863100
H 0.14362200 -5.06275100 3.08619900
H 0.65260700 -3.90707600 4.31713200
H 1.86079200 -4.94473300 3.52946100
C -0.16400200 -2.53964900 2.03833900
H -0.04153300 -1.69239500 1.34453700
H -0.57647900 -2.12641200 2.97110900
H -0.92210800 -3.20853100 1.60232400
C 4.49099300 -2.54638900 2.19050800
H 3.84032500 -3.42743600 2.09739700
C 4.58779600 -2.21958400 3.67734000
H 4.89468900 -3.11162300 4.24266900
H 3.62633500 -1.88004000 4.08932300
H 5.32938400 -1.43542200 3.88914000
C 5.84863000 -2.94377700 1.62135600
H 5.76303500 -3.33984700 0.60073600
H 6.30938600 -3.72845000 2.23871500
H 6.54568700 -2.09147300 1.59311700
C 5.44259100 2.03266500 0.86011300
H 5.04829700 3.03042400 0.61537600
C 6.17563800 2.14750900 2.18799300
H 5.48770100 2.30564400 3.03125000
H 6.87046500 2.99871500 2.16348300
H 6.77985900 1.25695200 2.41558600
C 6.39717600 1.64433600 -0.26836100
H 6.82865500 0.64583000 -0.09517700
H 7.22733700 2.36116900 -0.34524800
H 5.88697300 1.62240900 -1.24262400
C 3.04671800 2.55447200 -0.55326100
C 2.71311600 3.70257000 0.20814000
C 2.58167900 4.92046500 -0.46669100
H 2.33391500 5.82358200 0.09405600

C 2.76411800 5.01214300 -1.84428100
H 2.66356800 5.97792500 -2.34475600
C 3.04284200 3.86931900 -2.58304400
H 3.15294500 3.94113100 -3.66815100
C 3.17796100 2.62655800 -1.95698900
C 2.52286800 3.60870100 1.71455000
H 3.41058900 3.09546700 2.12823000
C 1.30990700 2.76003900 2.10031400
H 0.41869000 3.05399500 1.52261200
H 1.06259400 2.89486600 3.16306300
H 1.48929200 1.68423000 1.95847500
C 2.44454900 4.96509200 2.39763200
H 3.30530300 5.60495300 2.15659400
H 2.41790100 4.84206500 3.48911800
H 1.53023000 5.50812400 2.11445700
C 3.38073400 1.36243500 -2.76777400
H 3.89197300 0.63208600 -2.11479900
C 2.02008300 0.78258200 -3.15445900
H 1.31097700 0.66189600 -2.30942300
H 2.10618400 -0.18865600 -3.66461300
H 1.47332300 1.45981700 -3.82772200
C 4.24983300 1.54325800 -4.00292000
H 3.76873100 2.17942300 -4.76037800
H 4.44552600 0.57422300 -4.48494500
H 5.22065800 1.99708600 -3.75750400
H -0.24353700 1.24896400 0.66052300
Mg -1.78138300 0.26029800 -1.01769700
N -2.97419700 1.69620500 -0.05433800
C -4.23864700 1.64438300 -0.45949000
C -4.83846500 0.48245200 -0.99770600
H -5.84716700 0.63974600 -1.37485900
C -4.42405400 -0.86095600 -0.99461700
N -3.19831200 -1.27235800 -0.64094800
C -2.44427200 2.86808000 0.53752700
C -2.43381300 3.01314900 1.94735000
C -1.90220700 4.18131900 2.50423600
H -1.90302800 4.30118600 3.59167600
C -1.36359200 5.18521300 1.70612900
H -0.95999400 6.09377000 2.16032600
C -1.34153500 5.01909400 0.32494900
H -0.90980900 5.80128200 -0.30777100
C -1.88476000 3.88072100 -0.28103600
C -2.93478400 1.90957000 2.85665000
H -3.55943700 1.23702400 2.24641900
C -3.78509800 2.41785800 4.01380800
H -4.60284100 3.06916600 3.67333800
H -4.23320100 1.57813500 4.56540700
H -3.19366100 2.99279400 4.74264800
C -1.75309200 1.09352900 3.37793100
H -1.06349100 1.72960100 3.95613100
H -2.08340800 0.27866400 4.03970300
H -1.17739900 0.63633400 2.55776400
C -1.87055700 3.76296300 -1.79157000
H -2.46499000 2.87935900 -2.07552500
C -2.49911100 4.97610300 -2.46835400
H -1.90210600 5.88825000 -2.31369900
H -2.57381200 4.82178200 -3.55479600
H -3.51115700 5.18150300 -2.08951600
C -0.45160700 3.52967100 -2.29358100
H 0.01115500 2.65539200 -1.80729800
H -0.43962400 3.34067400 -3.37741300
H 0.18766300 4.40138200 -2.08850400
C -5.15520300 2.85912900 -0.33949300
H -4.52873500 3.71691400 -0.04643200
C -5.82322900 3.22171200 -1.66108800
H -6.31604700 4.20238200 -1.58594300
H -5.09947200 3.27347800 -2.48708200
H -6.59808300 2.49676300 -1.95032100
C -6.18424000 2.64219200 0.76453300
H -5.70552000 2.40016800 1.72429900
H -6.80011000 3.54116400 0.91581900
H -6.86134600 1.80943900 0.51875100
C -5.43500200 -1.88248500 -1.52029300
H -5.20426400 -2.84467800 -1.03711500
C -5.19765600 -2.06914600 -3.01713600
H -4.16167300 -2.37088200 -3.22565000
H -5.86246000 -2.84184100 -3.43047300
H -5.38279500 -1.13380600 -3.56709600
C -6.89475600 -1.57667400 -1.22134600

H -7.28549000 -0.74057300 -1.82046300
H -7.52154300 -2.44912200 -1.45723700
H -7.05804600 -1.32765800 -0.16186100
C -2.96379100 -2.63809000 -0.36619900
C -2.08320900 -3.38751900 -1.18071100
C -1.79280000 -4.71292400 -0.83876200
H -1.09803900 -5.28406600 -1.46101600
C -2.37334800 -5.31546700 0.27068500
H -2.14237000 -6.35432300 0.52015700
C -3.26080500 -4.58387100 1.05742600
H -3.72076400 -5.06093300 1.92650000
C -3.56882600 -3.25340600 0.76467000
C -1.40417900 -2.74505700 -2.36980700
H -1.98795100 -1.85784600 -2.66639000
C -0.01571200 -2.26347700 -1.95557400
H 0.63516700 -3.12818400 -1.77090600
H 0.43541300 -1.61958700 -2.72702000
H -0.06398800 -1.70591100 -1.00036700
C -1.29173300 -3.64752900 -3.58974400
H -2.26844200 -4.05417400 -3.88830200
H -0.88763800 -3.08772400 -4.44598800
H -0.61648700 -4.50075200 -3.41708300
C -4.47410100 -2.45423500 1.68245700
H -5.04575000 -1.74174500 1.06743500
C -3.62612200 -1.61485000 2.63015500
H -2.90816000 -1.00138300 2.06620800
H -4.24735900 -0.93883000 3.23877800
H -3.05244400 -2.25596300 3.31890400
C -5.49357100 -3.29268300 2.43615400
H -5.02596300 -3.95089500 3.18424900
H -6.19574100 -2.64600100 2.98185700
H -6.08342000 -3.92845800 1.75873500
H -1.37541300 0.45872800 -2.71741500

Int-4
173
-3268.801158

Mg 1.70364000 -0.07774800 -0.12150100
O -0.11202600 0.48273600 0.07940100
N 2.80370600 -1.64152600 0.58540700
C 3.83261200 -1.41004800 1.41464800
C 4.44538900 -0.15535300 1.57401300
H 5.30886100 -0.16590600 2.23672000
C 4.27001100 1.04415800 0.85645400
N 3.20294000 1.29099900 0.09381200
C 2.50788200 -2.97865000 0.19804300
C 2.96573700 -3.41543400 -1.06803000
C 2.70345200 -4.73715900 -1.44625800
H 3.05340200 -5.10233700 -2.41418500
C 1.96625000 -5.58644100 -0.62713800
H 1.76644500 -6.61315500 -0.94307300
C 1.45351400 -5.11572700 0.57791100
H 0.83878900 -5.77599700 1.19392700
C 1.71247700 -3.81279400 1.01323200
C 3.58519800 -2.42936400 -2.04750600
H 2.99175300 -1.49592300 -1.94113700
C 5.03257400 -2.04985300 -1.74889100
H 5.15115700 -1.57312000 -0.76681800
H 5.41173600 -1.34093300 -2.50176300
H 5.68675500 -2.93507500 -1.77935000
C 3.44362600 -2.85868400 -3.50048100
H 4.08388900 -3.72128200 -3.73874900
H 3.75281300 -2.04447600 -4.17227000
H 2.40907600 -3.13121200 -3.75702100
C 1.12581400 -3.28287600 2.30641900
H 1.84030700 -2.54813800 2.71386800
C 0.92406900 -4.35441300 3.36663700
H 0.12241800 -5.05763800 3.09339200
H 0.62955300 -3.89995100 4.32320600
H 1.83842900 -4.94139800 3.54160900
C -0.17769700 -2.53602800 2.03913200
H -0.05214100 -1.69203200 1.34197900
H -0.59081900 -2.11802000 2.96951300
H -0.93653600 -3.20496400 1.60459500
C 4.47558400 -2.55178900 2.20597700
H 3.82408000 -3.43209100 2.11190800
C 4.56772800 -2.22288200 3.69264200
H 4.87115600 -3.11458600 4.26036300

H	3.60542400	-1.88113500	4.10080400
H	5.30990400	-1.43966800	3.90589000
C	5.83465100	-2.95191700	1.64218000
H	5.75205800	-3.34924400	0.62181000
H	6.29206700	-3.73641700	2.26223800
H	6.53304700	-2.10066400	1.61525600
C	5.44199800	2.02178600	0.87208300
H	5.05207400	3.02022800	0.62335500
C	6.16894900	2.13746800	2.20326600
H	5.47738000	2.29931400	3.04283700
H	6.86616700	2.98676100	2.18024600
H	6.76970000	1.24582600	2.43574000
C	6.40094400	1.62817300	-0.25085200
H	6.82874700	0.62885300	-0.07330000
H	7.23349100	2.34249000	-0.32533300
H	5.89546200	1.60544700	-1.22755800
C	3.05593700	2.54730100	-0.55444600
C	2.72431400	3.69918600	0.20204600
C	2.60567800	4.91645900	-0.47636200
H	2.36043800	5.82252400	0.08070900
C	2.79787300	5.00382900	-1.85293500
H	2.70751900	5.96917400	-2.35615900
C	3.07338600	3.85735800	-2.58729700
H	3.19110200	3.92597500	-3.67179100
C	3.19620400	2.61519100	-1.95751400
C	2.52305800	3.60986800	1.70732600
H	3.40620100	3.09497100	2.12875300
C	1.30465300	2.76581700	2.08635700
H	0.41945100	3.05842000	1.49872200
H	1.04777600	2.90644900	3.14606200
H	1.48346000	1.68902400	1.95159800
C	2.44421100	4.96830800	2.38622400
H	3.30973200	5.60400900	2.15132200
H	2.40731900	4.84820400	3.47773800
H	1.53471700	5.51433700	2.09344600
C	3.39640200	1.34774200	-2.76369700
H	3.90217400	0.61722200	-2.10663400
C	2.03477600	0.77231000	-3.15354800
H	1.32259200	0.65572500	-2.31050000
H	2.11894000	-0.20023900	-3.66148900
H	1.49265700	1.45024200	-3.82987900
C	4.27049800	1.52106000	-3.99636900
H	3.79458100	2.15667900	-4.75755600
H	4.46395800	0.54971800	-4.47460700
H	5.24227600	1.97178500	-3.74900900
H	-0.24712500	1.24452900	0.65779700
Mg	-1.78052300	0.26601500	-1.03033700
N	-2.96899400	1.70853300	-0.06968700
C	-4.24124900	1.64977800	-0.44713800
C	-4.84249600	0.49080100	-0.99106000
H	-5.85541000	0.64977800	-1.35659600
C	-4.42601900	-0.85140000	-1.00178200
N	-3.19932600	-1.26433300	-0.65186600
C	-2.43723900	2.87790100	0.52638600
C	-2.43389900	3.02365000	1.93613300
C	-1.90192400	4.19028700	2.49564900
H	-1.90850400	4.31033800	3.58305600
C	-1.35701400	5.19274800	1.70014400
H	-0.95370700	6.10052700	2.15612200
C	-1.32808200	5.02597900	0.31922000
H	-0.89076600	5.80675100	-0.31128000
C	-1.87045700	3.88855200	-0.28946300
C	-2.94580500	1.92374800	2.84348100
H	-3.56592100	1.25068600	2.22955200
C	-3.80752100	2.43814600	3.98942600
H	-4.61920800	3.09057500	3.63676100
H	-4.26440800	1.60164200	4.53878300
H	-3.22271300	3.01412400	4.72278000
C	-1.77111600	1.10660200	3.37848500
H	-1.08632700	1.74219700	3.96291300
H	-2.10953900	0.29291700	4.03759000
H	-1.18770500	0.64756800	2.56479400
C	-1.84199600	3.76722900	-1.79943000
H	-2.45250100	2.89591200	-2.08787300
C	-2.43184200	4.99241300	-2.48905700
H	-1.81108400	5.88877700	-2.33581500
H	-2.50088300	4.83272600	-3.57504300
H	-3.44089100	5.22900500	-2.12074800
C	-0.42163700	3.50111400	-2.28126700

H 0.01313300 2.61614800 -1.78904100
H -0.39764800 3.31201200 -3.36488100
H 0.23393200 4.35785100 -2.06500200
C -5.16692400 2.85422200 -0.29684500
H -4.55356500 3.70142300 0.04959000
C -5.79734400 3.26595300 -1.62285500
H -6.31234100 4.23273400 -1.52130300
H -5.04797800 3.36826200 -2.42059900
H -6.54574900 2.53865800 -1.97020400
C -6.22844700 2.58869200 0.76512400
H -5.78029900 2.28872500 1.72339400
H -6.83924900 3.48530400 0.94796100
H -6.90796300 1.77906600 0.45728400
C -5.43533100 -1.87078500 -1.53484400
H -5.20613000 -2.83484600 -1.05459000
C -5.19215100 -2.05143400 -3.03151600
H -4.15514800 -2.35149600 -3.23727500
H -5.85482800 -2.82296200 -3.45039900
H -5.37594600 -1.11404600 -3.57843100
C -6.89637400 -1.56683600 -1.24070000
H -7.28471700 -0.72799200 -1.83759000
H -7.52184900 -2.43828300 -1.48359900
H -7.06447100 -1.32309400 -0.18071600
C -2.96688700 -2.63026500 -0.37643700
C -2.08468500 -3.38121700 -1.18779700
C -1.79638200 -4.70666600 -0.84416500
H -1.10010200 -5.27865100 -1.46396500
C -2.38098400 -5.30825800 0.26357800
H -2.15175300 -6.34721200 0.51426700
C -3.27038600 -4.57547200 1.04705000
H -3.73358100 -5.05193500 1.91469900
C -3.57598400 -3.24472400 0.75307800
C -1.40230700 -2.74117800 -2.37623000
H -1.98295000 -1.85221900 -2.67362700
C -0.01262300 -2.26384200 -1.96138900
H 0.63554900 -3.13037000 -1.77566300
H 0.44093300 -1.62198500 -2.73305000
H -0.06009300 -1.70530500 -1.00672000
C -1.29195800 -3.64438100 -3.59586000
H -2.26984600 -4.04762300 -3.89517300
H -0.88509200 -3.08625100 -4.45189800
H -0.61996000 -4.49999200 -3.42231900
C -4.48363000 -2.44455800 1.66788100
H -5.05896500 -1.73749700 1.05006400
C -3.63926000 -1.59673700 2.61129900
H -2.92279800 -0.98388200 2.04471600
H -4.26379600 -0.91954100 3.21534100
H -3.06425400 -2.23179500 3.30450000
C -5.49931600 -3.28301500 2.42662500
H -5.02855000 -3.93573000 3.17755900
H -6.20325300 -2.63608800 2.96975100
H -6.08757600 -3.92426200 1.75300900
H -1.36830800 0.45837000 -2.72907800

Int-5
173
-3268.803789

Mg 1.74506700 -0.20764700 0.20206400
O 0.01775700 0.53096900 -0.17463800
N 3.40247000 0.91071700 0.59577000
C 4.95572100 -0.82084600 -0.04608700
H 6.02171500 -1.02971000 -0.10082200
C 3.16446200 2.23948600 1.04284600
C 3.11022400 3.29612100 0.10526200
C 4.64719500 0.48159000 0.38809400
C 5.84080500 1.39326900 0.66546600
H 5.45754500 2.42334200 0.73542600
C 2.96604200 2.46703300 2.42276800
C 2.66968500 4.82596600 1.94363800
H 2.47359100 5.84213500 2.29482600
C 2.97105800 1.30181000 3.39310400
H 3.66734400 0.54484500 2.99248100
C 2.84706900 4.58406000 0.58487900
H 2.79400400 5.42193300 -0.11327300
C 2.73266100 3.77558300 2.85261500
H 2.58320200 3.97563600 3.91657000
C 3.41233900 3.03711200 -1.36298700
H 4.34761600 2.44604200 -1.38974800

C	6.47149200	1.05551600	2.01342200
H	6.85484800	0.02370400	2.02818000
H	5.75568100	1.15560400	2.84053300
H	7.31587700	1.72687700	2.22633900
C	6.88432800	1.37403900	-0.44536400
H	7.62101000	2.17492200	-0.28800900
H	6.43696800	1.52517200	-1.43847000
H	7.44618600	0.42911800	-0.48092000
C	2.34727100	2.19804100	-2.06929700
H	2.36892000	1.13990300	-1.76519100
H	2.51374600	2.19150300	-3.15673400
H	1.33623700	2.59996400	-1.89968200
C	1.59186100	0.65224500	3.45640100
H	0.85134600	1.31039100	3.93447300
H	1.60901700	-0.29277400	4.02005300
H	1.14679500	0.44339000	2.46619500
C	3.44836200	1.66768700	4.79009900
H	4.42393300	2.17498300	4.77407900
H	3.54819400	0.76829800	5.41406800
H	2.73976100	2.33148000	5.30737200
C	3.66813800	4.30785600	-2.15884400
H	2.75161200	4.90717500	-2.26976600
H	4.01420700	4.06250200	-3.17241900
H	4.43166100	4.94603800	-1.69246500
Mg	-1.79965500	0.45122400	0.76636800
N	-2.75969500	1.91044200	-0.41724400
C	-4.90187600	0.78431300	-0.41816700
H	-5.95018600	0.94909300	-0.66594000
C	-1.94723900	2.99088100	-0.83267400
C	-1.46075300	3.90527800	0.13562400
C	-4.05515800	1.85602600	-0.73938500
C	-4.73596300	3.04154500	-1.42249800
H	-3.94488200	3.69157500	-1.82766000
C	-1.54238100	3.12238800	-2.18824500
C	-0.19677000	5.06213600	-1.59239600
H	0.47445600	5.87282000	-1.88820700
C	-2.00847400	2.15879000	-3.26461500
H	-2.80169600	1.52407400	-2.83730000
C	-0.58520600	4.91981800	-0.26518300
H	-0.20514600	5.61902700	0.48649300
C	-0.67776000	4.16422700	-2.54055300
H	-0.36514300	4.26535400	-3.58501500
C	-1.85735500	3.81322900	1.59558200
H	-2.57508000	2.98215600	1.70118000
C	-5.63825000	2.64853600	-2.58551500
H	-6.55987700	2.14964300	-2.25146700
H	-5.13632400	1.96690100	-3.28761500
H	-5.94842900	3.54035100	-3.15069700
C	-5.49779600	3.85772600	-0.38291100
H	-5.92390100	4.76982600	-0.82646500
H	-4.84432400	4.16151600	0.44663600
H	-6.32618400	3.27627200	0.04983900
C	-0.64491300	3.50225100	2.46408600
H	-0.07856700	2.63829000	2.08293500
H	-0.94433400	3.26504900	3.49595400
H	0.04135600	4.36186900	2.49704000
C	-0.87272900	1.22967300	-3.68033900
H	-0.03280600	1.79451600	-4.11703000
H	-1.20433900	0.49808600	-4.43308800
H	-0.48321300	0.66178900	-2.82225300
C	-2.59208000	2.87875000	-4.47512300
H	-3.38525500	3.58557100	-4.19179000
H	-3.02388900	2.16157200	-5.18888700
H	-1.82602900	3.45089500	-5.02045700
C	-2.54715400	5.08318800	2.08393600
H	-1.86559900	5.94760500	2.05608900
H	-2.88252000	4.97035800	3.12527000
H	-3.42480000	5.34512100	1.47643600
N	2.78881700	-1.86652300	-0.39033700
C	2.07067000	-3.01852500	-0.82593800
C	1.42644400	-3.83039100	0.13234600
C	4.13271900	-1.90283800	-0.39284700
C	4.85608600	-3.20852300	-0.72446000
H	4.19505900	-3.79803300	-1.37616400
C	1.99264900	-3.32715700	-2.20490900
C	0.66755400	-5.28785700	-1.65488400
H	0.12508200	-6.17988100	-1.97844800
C	2.61097300	-2.39703400	-3.22966800
H	3.49920400	-1.93042500	-2.77178500

C 0.73089900 -4.96256900 -0.30410800
H 0.22974900 -5.59932900 0.42837900
C 1.28853500 -4.46919000 -2.59498800
H 1.22020500 -4.72381200 -3.65562300
C 1.42645500 -3.41845000 1.58948800
H 2.38291600 -2.90128000 1.78889800
C 6.16919900 -3.04093400 -1.47587900
H 6.97927800 -2.65075000 -0.84218000
H 6.06938400 -2.36656200 -2.33895600
H 6.51144700 -4.01512100 -1.85358100
C 5.04748100 -4.01678400 0.55683400
H 5.53971500 -4.97669500 0.34426800
H 4.08849000 -4.23985200 1.04534300
H 5.67306600 -3.47118200 1.28004300
C 0.29175000 -2.42384000 1.82249200
H 0.23222400 -1.63703200 1.04732400
H 0.33474900 -1.92978900 2.80293400
H -0.68259000 -2.92674500 1.74821600
C 1.63228000 -1.26998100 -3.54538000
H 0.73924200 -1.65689400 -4.05997800
H 2.08473100 -0.49936800 -4.18775700
H 1.27290700 -0.77387600 -2.62831200
C 3.07931000 -3.09489800 -4.49648700
H 3.75541700 -3.93409900 -4.27504600
H 3.62001000 -2.39381700 -5.14804300
H 2.24155200 -3.49257800 -5.08849900
C 1.31081900 -4.57389500 2.57029400
H 0.32643500 -5.06211800 2.50922900
H 1.41946200 -4.21588900 3.60376700
H 2.07974400 -5.34056600 2.39713400
N -3.41672900 -0.89388500 0.48671300
C -3.19819200 -2.23461800 0.89040900
C -2.85911600 -3.18682300 -0.10613900
C -4.63085200 -0.48198800 0.13852800
C -5.85309800 -1.38341800 0.32204100
H -5.52447200 -2.27098300 0.88581500
C -3.25961100 -2.61710300 2.25152200
C -2.61781000 -4.88093300 1.62192000
H -2.39538000 -5.91300600 1.90693800
C -3.68660200 -1.66594500 3.35245200
H -3.80796200 -0.66556700 2.90424300
C -2.57129800 -4.49760800 0.28315900
H -2.31215400 -5.23844500 -0.47697700
C -2.96557000 -3.94503500 2.58807900
H -3.01939900 -4.24900600 3.63813800
C -2.79572800 -2.77977600 -1.56669900
H -3.59388100 -2.03709300 -1.73637000
C -6.93552200 -0.69480500 1.14875100
H -7.44010400 0.10506500 0.58680600
H -6.52955000 -0.24223800 2.06478300
H -7.71131900 -1.41608200 1.44691400
C -6.41686900 -1.87352000 -1.00714400
H -7.29724000 -2.51263500 -0.84367100
H -5.68606500 -2.46439300 -1.57623800
H -6.73240800 -1.03389000 -1.64569700
C -1.47844300 -2.08363800 -1.88952700
H -1.34604000 -1.14852800 -1.32724800
H -1.42312200 -1.82395300 -2.95793600
H -0.62178900 -2.73676100 -1.66035500
C -2.63333000 -1.54511100 4.44757700
H -2.44426400 -2.51220700 4.94082400
H -2.96374300 -0.84224500 5.22674900
H -1.68804600 -1.16028000 4.04085100
C -5.02513800 -2.09888200 3.94865900
H -5.80281900 -2.23444700 3.18392800
H -5.39491400 -1.35766900 4.67305700
H -4.93089600 -3.05695700 4.48405600
C -3.02752100 -3.93378200 -2.53055800
H -2.18134300 -4.63873900 -2.53596600
H -3.13105500 -3.56004900 -3.55942500
H -3.93574400 -4.50519100 -2.28826800
H 0.00038600 1.28726600 -0.77594400
H -1.38538900 0.39918900 2.45890700

Product-coplanar (**4c**)
173
-3268.829832

Mg 1.43152600 -0.14922000 0.08055300

O	0.02170400	-0.33377500	-1.26607800
N	2.54605900	-1.80893500	0.59885900
C	3.59353500	-1.64652100	1.41254800
C	4.27347500	-0.42433500	1.55982900
H	5.14460800	-0.47751700	2.21101700
C	4.18702300	0.75654500	0.79530800
N	3.13463300	1.07620400	0.03827900
C	2.13142500	-3.11131100	0.21709600
C	2.47832500	-3.56750400	-1.08076100
C	2.10033500	-4.86257400	-1.44990200
H	2.36826600	-5.24135700	-2.43849800
C	1.35272500	-5.67184700	-0.59916500
H	1.05869000	-6.67562500	-0.91611200
C	0.96281100	-5.18788900	0.64361500
H	0.34880000	-5.81177700	1.29980400
C	1.35146200	-3.91628400	1.07745500
C	3.16868300	-2.64209100	-2.07300200
H	2.62639000	-1.67556000	-2.00682700
C	4.63286100	-2.34845400	-1.75177000
H	4.76887900	-1.83599000	-0.79085300
H	5.07582300	-1.70351600	-2.52670600
H	5.22408300	-3.27760400	-1.73190700
C	3.06057900	-3.11693500	-3.51504900
H	3.66089500	-4.02259400	-3.69191100
H	3.44608700	-2.34886800	-4.20129100
H	2.02666600	-3.34313000	-3.81078400
C	0.91925800	-3.41489300	2.44001600
H	1.52357400	-2.52459400	2.67584400
C	1.15046700	-4.43298500	3.54995600
H	0.50626100	-5.31797600	3.43661000
H	0.92152200	-3.99676400	4.53347700
H	2.19152200	-4.78754800	3.57789400
C	-0.53487700	-2.96510100	2.41234800
H	-0.71263200	-2.22570800	1.61556800
H	-0.81985900	-2.49649600	3.36615000
H	-1.21637500	-3.80960300	2.22683200
C	4.19607900	-2.82022800	2.18766500
H	3.51695600	-3.67724600	2.07640100
C	4.29497400	-2.51353800	3.67847600
H	4.57934800	-3.41757200	4.23718100
H	3.34091400	-2.15450400	4.09126100
H	5.05383300	-1.74834500	3.89845700
C	5.54384200	-3.25681700	1.62414700
H	5.45775400	-3.60871500	0.58768100
H	5.96011000	-4.08387600	2.21780900
H	6.27847900	-2.43658100	1.63831300
C	5.45756700	1.60610400	0.76219600
H	5.18930400	2.60662600	0.39081800
C	6.14426900	1.79156800	2.10832800
H	5.44548400	2.11059400	2.89530400
H	6.92725200	2.55937300	2.03054900
H	6.63954800	0.87443300	2.45967200
C	6.41196400	0.99031000	-0.25841000
H	6.74845600	-0.00663500	0.06724800
H	7.30330900	1.61919800	-0.39932600
H	5.92883200	0.86825300	-1.23819400
C	3.12265500	2.31465500	-0.66246500
C	2.90188300	3.51958000	0.05920200
C	2.85886500	4.72116200	-0.65460700
H	2.69043600	5.65864500	-0.12152700
C	3.03579100	4.75605000	-2.03678200
H	3.00069900	5.70941100	-2.56954500
C	3.25333100	3.57337600	-2.72870000
H	3.38712000	3.59857000	-3.81424600
C	3.29804600	2.34136800	-2.06410600
C	2.75854900	3.50371800	1.57386200
H	3.63730700	2.96351100	1.97010900
C	1.52333100	2.73539200	2.05279200
H	0.65471700	2.89801700	1.39658800
H	1.22840400	3.05818500	3.06160700
H	1.69647600	1.65267000	2.11544200
C	2.79884500	4.89175500	2.19482600
H	3.69580200	5.45624700	1.90161400
H	2.79878900	4.81660000	3.29117900
H	1.91815400	5.48950100	1.91569800
C	3.48692100	1.07380900	-2.87370900
H	3.66217800	0.25230400	-2.15770000
C	2.21438500	0.75851100	-3.65771200
H	1.32971400	0.76359800	-3.00628300

H 2.27410500 -0.22297000 -4.15436100
H 2.03910600 1.51261400 -4.44157100
C 4.68488200 1.13404300 -3.81420900
H 4.55162700 1.89607800 -4.59694600
H 4.82553900 0.17227900 -4.33036100
H 5.62078000 1.37035000 -3.28857700
H 0.03374700 -0.64339800 -2.17348400
Mg -1.48978500 0.15815800 -0.09002000
N -2.58718400 1.93002300 0.02818600
C -3.76945200 2.04064600 -0.58345400
C -4.42514800 0.97118600 -1.22189500
H -5.34383400 1.25124700 -1.73631100
C -4.20852300 -0.41499000 -1.15057700
N -3.16602300 -0.98175200 -0.53743200
C -2.02158800 3.06579500 0.67583900
C -2.05487600 3.17452400 2.08428900
C -1.48864100 4.30540500 2.68620600
H -1.51652400 4.39588600 3.77637100
C -0.89276100 5.30608900 1.93262800
H -0.46313900 6.18361700 2.42212300
C -0.84773300 5.18422100 0.54556000
H -0.37776800 5.97376000 -0.04611100
C -1.40896200 4.08232300 -0.10414500
C -2.66349400 2.10786300 2.97316100
H -3.16455600 1.37559800 2.31612200
C -3.71346200 2.67157100 3.92579600
H -4.49201800 3.24532500 3.40390900
H -4.21000800 1.86376800 4.48399800
H -3.26411000 3.34367000 4.67276000
C -1.57641500 1.38036500 3.76261400
H -1.03460400 2.08138700 4.41765000
H -2.00494800 0.59906700 4.40867400
H -0.83492100 0.90629900 3.10233400
C -1.36976400 3.97323100 -1.61603800
H -2.30918500 3.49481000 -1.93718700
C -1.29072700 5.31640600 -2.32497400
H -0.31529000 5.80495900 -2.17756100
H -1.41479100 5.18531400 -3.40953200
H -2.06986400 6.01326400 -1.98078800
C -0.23279200 3.06221200 -2.06066000
H -0.32216400 2.04833900 -1.64430700
H -0.20353000 2.96048300 -3.15658300
H 0.73659100 3.46533500 -1.73706500
C -4.55987200 3.34912600 -0.54347100
H -3.88097600 4.14542600 -0.20336600
C -5.11918400 3.78503500 -1.89219100
H -5.53064000 4.80235500 -1.81968100
H -4.35221500 3.79555000 -2.68010800
H -5.93857400 3.13862400 -2.23953600
C -5.67002700 3.22514200 0.49619000
H -5.27263500 2.92229700 1.47504500
H -6.19805500 4.18072200 0.63006500
H -6.41169200 2.46866300 0.19630600
C -5.28219900 -1.27270200 -1.81951100
H -4.95254800 -2.32164800 -1.75518800
C -5.41710100 -0.92573200 -3.29918200
H -4.44456200 -0.92644300 -3.81236600
H -6.06463900 -1.65403100 -3.81011100
H -5.86493100 0.06733400 -3.45144100
C -6.62805900 -1.17844900 -1.11022300
H -7.03352800 -0.15561800 -1.14271000
H -7.36620600 -1.83969400 -1.58738800
H -6.55935500 -1.47077100 -0.05354100
C -3.16913400 -2.38011400 -0.27646400
C -2.54263200 -3.28719800 -1.15981800
C -2.46711300 -4.63723400 -0.79844900
H -1.96561700 -5.34087000 -1.47034600
C -3.03861400 -5.10172800 0.38002800
H -2.97550100 -6.16058500 0.64291300
C -3.70957300 -4.21095100 1.21386200
H -4.17733700 -4.58280900 2.12950200
C -3.78455200 -2.84862000 0.91106900
C -2.04167900 -2.85516300 -2.52259300
H -2.16231200 -1.76004100 -2.58492000
C -0.56921600 -3.17919400 -2.73258700
H -0.39992500 -4.26344900 -2.82511100
H -0.19517300 -2.71705900 -3.66098600
H 0.04945700 -2.83107500 -1.89458700
C -2.87972900 -3.48429900 -3.63417000

H -3.95385900 -3.28698200 -3.51466900
H -2.57832000 -3.10498000 -4.62237200
H -2.75190800 -4.57766200 -3.65176300
C -4.47790300 -1.88781200 1.85882100
H -4.81753500 -1.01919700 1.26958300
C -3.49723000 -1.35475800 2.89597700
H -2.61186600 -0.89292600 2.42920800
H -3.96789300 -0.59483400 3.53836000
H -3.13254200 -2.16478000 3.54668900
C -5.70138500 -2.48520100 2.54024200
H -5.43179700 -3.26368800 3.26978100
H -6.25157200 -1.71180000 3.09536400
H -6.39708100 -2.94028600 1.82034700
H -0.09677400 0.19350300 1.15922900

Product-orthogonal (**4c**)
173
-3268.83433

Mg -1.43131100 0.23589300 -0.19199900
N -3.03644300 -0.60985700 -1.16026100
N -2.57946700 1.70919700 0.69945500
C -4.14350600 0.10324900 -1.39793900
C -4.45402900 1.31891800 -0.76396300
H -5.42230600 1.73622200 -1.04339200
C -3.81721000 1.98773200 0.30150500
C -2.98883900 -1.97118600 -1.57021300
C -2.38318700 -2.36352500 -2.78399600
C -2.40579300 -3.71924700 -3.13656600
H -1.95893200 -4.02700100 -4.08701300
C -2.98485800 -4.67670800 -2.31467400
H -2.99868800 -5.72704300 -2.61512500
C -3.53523800 -4.28740500 -1.09628300
H -3.96517700 -5.04456500 -0.43544100
C -3.54232100 -2.94734300 -0.70138100
C -1.74168900 -1.37240400 -3.73611700
H -1.74373000 -0.38444400 -3.24473400
C -0.29014700 -1.74187900 -4.02655600
H 0.32607200 -1.70267300 -3.11679400
H 0.15727800 -1.05366000 -4.75838500
H -0.20261900 -2.75508700 -4.44875100
C -2.53594400 -1.25520400 -5.03464700
H -2.10773800 -0.48460700 -5.69339400
H -3.58810500 -0.99195800 -4.85671900
H -2.52911400 -2.20327500 -5.59494200
C -4.02290000 -2.56483400 0.68602600
H -4.39052200 -1.52455400 0.65235500
C -2.83879600 -2.60263000 1.65015100
H -2.41232900 -3.61694200 1.70629000
H -3.13672300 -2.31411500 2.66854600
H -2.01105100 -1.93677800 1.34813200
C -5.15563800 -3.43359500 1.21363500
H -5.99543100 -3.49893600 0.50670300
H -5.54549000 -3.02662700 2.15771700
H -4.82658800 -4.46170300 1.42738600
C -5.20396000 -0.41737100 -2.36720400
H -4.76893600 -1.27964500 -2.89616100
C -5.56797400 0.62011500 -3.42314700
H -6.19060700 0.16644000 -4.20855500
H -4.67493800 1.04224300 -3.90665000
H -6.14135000 1.46081000 -3.00579000
C -6.43887500 -0.92028000 -1.62902900
H -6.19150100 -1.73064100 -0.92906400
H -7.18613400 -1.31172200 -2.33493000
H -6.91737900 -0.11618600 -1.04854600
C -4.66277100 3.03528700 1.02187000
H -4.08548000 3.37047100 1.89962100
C -4.94418700 4.26059900 0.15928500
H -4.02538800 4.76919100 -0.16186800
H -5.54741700 4.99324100 0.71513400
H -5.50827300 3.99454800 -0.74756600
C -5.96839600 2.42485600 1.52733900
H -6.46910200 3.10314500 2.23435300
H -5.80523700 1.46525700 2.03916300
H -6.67392500 2.23394400 0.70501600
C -1.94173800 2.41954800 1.75250000
C -1.23176700 3.60631700 1.44540300
C -0.50070200 4.23896800 2.45409600
H 0.05461500 5.15115000 2.21989700

C	-0.46129700	3.72738600	3.74759400
H	0.11686800	4.23369700	4.52432500
C	-1.17066500	2.56985800	4.04267900
H	-1.14957900	2.17354400	5.06260300
C	-1.91314300	1.89616900	3.06540400
C	-1.20543600	4.14195800	0.02912200
H	-2.14565300	3.83930900	-0.45929900
C	-0.07115800	3.49777300	-0.75705400
H	-0.08293700	3.80480500	-1.81274000
H	-0.12004900	2.39576400	-0.73902700
H	0.91082400	3.77611700	-0.34280500
C	-1.11434700	5.65854900	-0.05378000
H	-0.13323900	6.03289200	0.27628400
H	-1.88122700	6.15408200	0.55963900
H	-1.24323500	5.99664000	-1.09205000
C	-2.71082500	0.66883200	3.46398500
H	-3.10189200	0.20761300	2.53932200
C	-1.86739600	-0.37478800	4.18801100
H	-1.40226000	0.03402000	5.09824100
H	-1.05952700	-0.78489100	3.56306400
H	-2.48170300	-1.23000000	4.50740100
C	-3.90406300	1.06982500	4.32971900
H	-4.56712900	0.21245400	4.52205600
H	-4.50509100	1.86250700	3.86358100
H	-3.56941300	1.45279600	5.30648700
Mg	1.43124500	-0.22265200	-0.17570200
N	3.04721700	0.60158600	-1.14654400
N	2.56701400	-1.69764500	0.72989800
C	4.14637100	-0.12495800	-1.38015500
C	4.44399400	-1.34013200	-0.73893200
H	5.40824400	-1.76906900	-1.01509000
C	3.79957300	-1.99570800	0.33017600
C	3.01667200	1.96210000	-1.56119900
C	2.41485900	2.35923600	-2.77528600
C	2.45524300	3.71359300	-3.13154500
H	2.01127000	4.02464800	-4.08228000
C	3.04833400	4.66536100	-2.31320400
H	3.07578600	5.71459400	-2.61650000
C	3.59655300	4.27182300	-1.09522000
H	4.03947600	5.02477500	-0.43827200
C	3.58635000	2.93292400	-0.69669700
C	1.76027100	1.37462000	-3.72516600
H	1.75144800	0.38742300	-3.23247200
C	0.31288900	1.76101500	-4.01447100
H	-0.30213900	1.73320700	-3.10349400
H	-0.14460700	1.07574300	-4.74288300
H	0.23752200	2.77367100	-4.44041400
C	2.55155800	1.24705300	-5.02457400
H	2.11400600	0.48079500	-5.68225200
H	3.60089700	0.97211100	-4.84764800
H	2.55480600	2.19459000	-5.58581000
C	4.07273700	2.54574600	0.68746500
H	4.43703100	1.50440800	0.64941000
C	2.89650800	2.58194400	1.66094900
H	2.46846000	3.59517500	1.72385600
H	3.20486100	2.29021200	2.67532400
H	2.06852500	1.91400900	1.36452000
C	5.21153200	3.40964100	1.20996300
H	6.04615000	3.47508200	0.49692400
H	5.60724300	2.99840500	2.14970600
H	4.88684800	4.43791400	1.42953500
C	5.21009700	0.37636500	-2.35609500
H	4.78566800	1.24360500	-2.88555900
C	5.55018800	-0.67107300	-3.41034100
H	6.17713700	-0.23158800	-4.20039100
H	4.64717000	-1.07846900	-3.88797000
H	6.10982400	-1.52075300	-2.99248100
C	6.45787400	0.86208400	-1.62824200
H	6.22845300	1.67784900	-0.92845000
H	7.20550600	1.24046300	-2.34083300
H	6.92913900	0.05247000	-1.04958000
C	4.63571900	-3.04329000	1.06113400
H	4.03518900	-3.40264000	1.91324700
C	4.97485300	-4.25008000	0.19371800
H	4.07973400	-4.76546700	-0.17977600
H	5.56015200	-4.98372000	0.76708300
H	5.57928700	-3.96448300	-0.68041300
C	5.90838000	-2.41038000	1.62084800
H	6.40588900	-3.08973700	2.32912000

H 5.70299700 -1.46687600 2.14721500
H 6.63182500 -2.18219100 0.82392400
C 1.91607400 -2.40192000 1.77861800
C 1.21118500 -3.59164100 1.46902300
C 0.46472000 -4.21706800 2.47059400
H -0.08506700 -5.13216200 2.23520200
C 0.40241800 -3.69443400 3.75901900
H -0.18939600 -4.19432000 4.52964400
C 1.10792300 -2.53554400 4.05705200
H 1.07073300 -2.13114700 5.07346100
C 1.86864400 -1.87060700 3.08744600
C 1.21397800 -4.13896100 0.05694600
H 2.17734800 -3.86659600 -0.40349900
C 0.12337100 -3.47333200 -0.77194100
H 0.16240200 -3.79111200 -1.82380100
H 0.19746100 -2.37286800 -0.76297700
H -0.87793300 -3.72330200 -0.38755700
C 1.08358100 -5.65292200 -0.01751500
H 0.08114800 -5.99644200 0.28116200
H 1.81496500 -6.16460100 0.62530300
H 1.23794600 -6.00304100 -1.04827700
C 2.66407100 -0.64422500 3.49347900
H 3.09497800 -0.20459300 2.57613000
C 1.80433000 0.42022000 4.16683500
H 1.29202000 0.02738500 5.05873300
H 1.03275700 0.83558800 3.50083800
H 2.41586200 1.27000200 4.50557000
C 3.81954500 -1.04158200 4.41076700
H 4.48518600 -0.18789700 4.61045200
H 4.42770400 -1.85029000 3.98282200
H 3.44535400 -1.40056200 5.38224300
H 0.00704500 0.01623000 -1.38399700
O -0.00946600 -0.00062800 1.11418600
H -0.01814900 -0.01954900 2.07341300

Int-6
173
-3268.767011

Mg 1.83686900 0.06581800 0.14430900
O 0.01833900 -0.03707600 -0.51379100
N 3.43984100 -1.11264300 -0.48886700
C 4.98685200 0.75159200 -0.30224700
H 6.04768800 0.97221100 -0.41750900
C 4.65329300 -0.57642400 -0.63276800
C 5.76569900 -1.34766700 -1.33709300
Mg -2.05471200 0.13258700 -0.31975400
N -2.04856200 -0.68461200 1.65711400
C -4.38315800 -1.15169200 1.91680500
H -5.11645100 -1.57402800 2.60474600
C -3.04874200 -1.26595100 2.31973800
C -2.79270200 -2.08477500 3.58750300
N 2.87047000 1.87490000 0.02650300
C 4.19818900 1.89974600 -0.10038500
C 4.98114200 3.21129400 -0.06363000
H 4.23772600 4.01635100 0.00143800
N -4.17997800 -0.12572000 -0.25732700
C -4.92229300 -0.56911500 0.74856700
C -6.44758400 -0.49919900 0.69994900
H 5.43941300 -2.39682200 -1.41924300
H -6.72131600 0.08277000 -0.19371100
H -1.70915900 -2.28310000 3.63236900
H -0.08372900 0.34765700 -1.39832100
H -2.00151900 1.01794700 -1.85265100
C -0.72235200 -0.70458000 2.09190400
C 0.13256300 -1.81255600 1.85847600
C -0.17378000 0.53180200 2.54350800
C 1.49239500 -1.67836200 2.16856600
C 1.19586300 0.60940000 2.82098300
C 2.04014400 -0.49195300 2.65372300
H 2.13885800 -2.53088400 1.95991700
H 1.62099200 1.56001700 3.14684500
H 3.11124200 -0.41388000 2.86517200
C 3.16487500 -2.49326000 -0.70258300
C 3.66448000 -3.49237600 0.18365300
C 2.26173000 -2.87025800 -1.73226700
C 3.17300100 -4.79792900 0.07264700
C 1.81751500 -4.19521300 -1.80511500
C 2.24357000 -5.15575600 -0.89706100

H	3.54433200	-5.56062800	0.76419700
H	1.12188200	-4.47626600	-2.60079400
H	1.87401800	-6.18195400	-0.95817000
C	2.02047200	3.00865000	-0.14719400
C	1.27576700	3.04484400	-1.36493700
C	1.79072000	3.99877400	0.84730200
C	0.27459500	4.00673300	-1.52095300
C	0.78430900	4.94908200	0.62412800
C	0.01630300	4.94915000	-0.53062100
H	-0.32216000	4.01691900	-2.43464100
H	0.60629000	5.71563900	1.38477000
H	-0.77792900	5.68683200	-0.66795900
C	-4.80282900	0.32483900	-1.45443700
C	-5.13577100	1.68506700	-1.62633500
C	-5.01345800	-0.59937400	-2.50381200
C	-5.66050100	2.10064300	-2.85380200
C	-5.54505000	-0.13902600	-3.71149700
C	-5.86649900	1.20240200	-3.89426900
H	-5.91281500	3.15625800	-2.99509200
H	-5.70535700	-0.84568100	-4.53036800
H	-6.27795800	1.54546500	-4.84643100
C	5.90395700	-0.79599400	-2.75673200
H	4.93377200	-0.73118700	-3.26799300
H	6.32754000	0.21985000	-2.74300600
H	6.56626600	-1.42828200	-3.36638700
C	5.86963400	3.25445800	1.17944500
H	5.33204500	2.99458200	2.10166500
H	6.70796700	2.54750200	1.08750800
H	6.30283600	4.25660100	1.31522400
C	-7.01198200	0.23700600	1.91026200
H	-6.89120800	-0.33695100	2.84112200
H	-6.52036300	1.20947800	2.06137600
H	-8.08861300	0.42480300	1.78260400
C	-3.50683600	-3.43348000	3.55388300
H	-4.59214900	-3.32649700	3.69560700
H	-3.35705200	-3.96923000	2.60599000
H	-3.14223000	-4.08090800	4.36584100
C	5.83479600	3.50074500	-1.29479600
H	6.60035600	2.72877400	-1.46277500
H	5.24500100	3.59194500	-2.21446000
H	6.36774100	4.45339000	-1.15922400
C	7.11411100	-1.31739000	-0.62800000
H	7.59774500	-0.33282800	-0.70862300
H	7.03891100	-1.55673100	0.44136100
C	-3.16369900	-1.33598000	4.86318400
H	-2.53236900	-0.45550200	5.02742700
H	-4.21089100	-0.99675000	4.83830000
H	-3.04912700	-1.99030800	5.73993500
C	-7.06967000	-1.88231700	0.54743800
H	-6.70526400	-2.39686100	-0.35277300
H	-6.84326300	-2.52803800	1.40992800
H	-8.16449700	-1.81235000	0.46706400
C	-0.22579500	-3.14006800	1.19492800
H	0.64063900	-3.32755300	0.53526900
C	-0.22319000	-4.29481900	2.19595300
H	0.71353200	-4.32847000	2.77221900
H	-1.05186400	-4.23131000	2.91482800
H	-0.31879500	-5.25668500	1.67060200
C	1.83434800	-1.91309400	-2.82854200
H	2.10487400	-0.89113000	-2.50927900
C	0.33637600	-1.94610600	-3.10063200
H	0.03139900	-1.12975700	-3.77224100
H	0.02834800	-2.88553800	-3.58495300
H	-0.23954200	-1.83945700	-2.17376700
C	1.59050500	2.09044900	-2.50979500
H	1.74118200	1.08319800	-2.07769500
C	2.91213500	2.42726100	-3.19495000
H	3.07198800	1.77194800	-4.06486400
H	2.92102700	3.46637900	-3.55928300
H	3.77153000	2.28913100	-2.52933400
C	-4.58384700	-2.04666400	-2.35809100
H	-4.58994600	-2.29144300	-1.28220100
C	-5.50466200	-3.03706300	-3.05620900
H	-5.46179100	-2.94367900	-4.15185400
H	-5.21590200	-4.07126800	-2.81787100
H	-6.55537100	-2.90553800	-2.75879800
C	-1.43557800	-3.20009700	0.27451200
H	-2.40525300	-3.18123800	0.79021600
H	-1.42922800	-2.38132500	-0.45997200

H -1.39285000 -4.13423400 -0.30665800
C 2.60829300 -2.21220900 -4.11213500
H 3.69459700 -2.23731400 -3.95128300
H 2.32195700 -3.19570100 -4.51601700
H 2.39553200 -1.46361900 -4.89028000
C 0.48613100 1.97311700 -3.54850400
H 0.73848300 1.18505900 -4.27362900
H -0.49403100 1.72904600 -3.10533000
H 0.37621700 2.90270900 -4.12762500
C -3.14698500 -2.20113800 -2.84541600
H -3.07749000 -2.02134400 -3.93006200
H -2.48084600 -1.45578900 -2.37987900
H -2.74372100 -3.20489100 -2.63981300
C -4.93280800 2.70531100 -0.52394100
H -4.61513800 2.15903100 0.38123800
C 2.64898800 4.17484700 2.09296900
H 3.32785700 3.30794900 2.15595200
C 3.48275500 5.45878600 1.99678500
H 2.83258300 6.34145900 2.09699800
H 4.22329700 5.50995200 2.80902900
H 4.01906700 5.57269700 1.04675800
C 4.78008300 -3.25357600 1.19397500
H 5.05144400 -2.18510500 1.16971500
C 6.01159900 -4.07419500 0.79966000
H 6.29843000 -3.93646800 -0.25122300
H 6.87997900 -3.81424500 1.42315500
H 5.82322000 -5.14938900 0.94120000
C 4.42054100 -3.59829100 2.63921800
H 5.31728900 -3.55301600 3.27400100
H 3.68514300 -2.91872800 3.08602000
H 4.02073900 -4.62030800 2.72552800
C -0.98795600 1.81480300 2.66068200
H -0.25067100 2.57232800 2.96723300
C -3.82271800 3.68818600 -0.88612600
H -4.09445200 4.27854300 -1.77573700
H -3.63365800 4.39887300 -0.06615100
H -2.88403100 3.16153400 -1.12025300
C -6.22665000 3.43549200 -0.17795300
H -6.08618100 4.10467500 0.68440700
H -6.57815000 4.05865300 -1.01458100
H -7.03971600 2.73709700 0.06937400
C -2.06444300 1.80423900 3.73748300
H -2.47616300 2.81679000 3.86210400
H -2.90568600 1.14767300 3.47212700
H -1.67023600 1.49008000 4.71425300
C -1.56898100 2.34217000 1.34991600
H -0.87781700 2.24081800 0.49662800
H -2.54232700 1.87796600 1.11184000
H -1.78475400 3.41632600 1.43932000
H 7.80424900 -2.04271600 -1.08312900
C 1.87232800 4.26012300 3.40491800
H 1.32051500 3.34839500 3.65677200
H 2.55841300 4.46009500 4.24076300
H 1.14264400 5.08343100 3.39063800

Transition state

B3LYP-D3$_{PCM(benzene)}$/def2-TZVP//B3LYP-D3/def2-SVP

173
-3271.240086

Mg -1.87859900 -0.04132300 -0.08127300
O -0.03897700 0.13489400 -0.30820300
N -3.35055300 1.28747000 -0.55744600
C -5.14864100 -0.27947800 -0.12168900
H -6.23231500 -0.36647300 -0.15568700
C -2.93047700 2.60079700 -0.94266100
C -2.62821800 3.54772400 0.06356400
C -4.65133200 0.98515800 -0.49868800
C -5.70768700 2.05329000 -0.82059100
H -5.17498600 2.94543800 -1.17443800
C -2.78982400 2.92072500 -2.31580300
C -2.09036100 5.16280200 -1.67373600
H -1.76489000 6.16575600 -1.96122100
C -3.06016500 1.86850500 -3.38664700
H -3.77354900 1.14607000 -2.96459600
C -2.20167800 4.82484700 -0.32650600
H -1.95487100 5.56697900 0.43501800
C -2.37554400 4.21437100 -2.65649700

H	-2.26500500	4.48673200	-3.70768900
C	-2.73388400	3.16888500	1.53606600
H	-3.42164000	2.31237900	1.60318500
C	-6.66472600	1.62630500	-1.94455300
H	-7.30651700	0.78294000	-1.64504600
H	-6.11518300	1.32653300	-2.84989600
H	-7.32589100	2.46599400	-2.21347300
C	-6.47298400	2.46017000	0.45030500
H	-7.16682800	3.28759100	0.22941700
H	-5.78508600	2.79782100	1.24033000
H	-7.06198800	1.62092100	0.85300800
C	-1.36689600	2.70874500	2.06930900
H	-0.91060100	1.91842500	1.45108700
H	-1.45267300	2.33221100	3.10093900
H	-0.64828600	3.53966300	2.06558400
C	-1.77362600	1.08914100	-3.71085300
H	-1.00700700	1.75431900	-4.13856300
H	-1.97112900	0.27911000	-4.43198500
H	-1.32496800	0.64152800	-2.81229100
C	-3.69815600	2.43560000	-4.66130900
H	-4.59967300	3.02581300	-4.43233800
H	-3.98756100	1.61583600	-5.33829600
H	-3.00257400	3.08338600	-5.21873500
C	-3.31285000	4.28449100	2.41677300
H	-2.62754600	5.14390000	2.49263700
H	-3.47970800	3.91338700	3.44094700
H	-4.27380300	4.65270200	2.02389200
Mg	1.89265600	0.19029800	-0.40905400
N	3.16668500	1.58225100	0.41293600
C	5.13343900	0.22228300	-0.01187300
H	6.21886700	0.26501300	0.02916100
C	2.59860400	2.79070400	0.92340000
C	2.19323000	3.81364700	0.03421500
C	4.49286600	1.41725200	0.37568700
C	5.43018800	2.57601200	0.75550400
H	4.80027400	3.45405000	0.95277400
C	2.40086900	2.93020100	2.32284500
C	1.47826900	5.15225600	1.94018000
H	1.05117100	6.07657900	2.33785100
C	2.70643600	1.77055100	3.26466300
H	3.49678500	1.16343200	2.80119800
C	1.62873400	4.98333000	0.56611000
H	1.31126500	5.77850100	-0.11274600
C	1.85796300	4.12587500	2.80807300
H	1.71113300	4.25568600	3.88200300
C	2.37312700	3.67851600	-1.47243900
H	2.82431100	2.69395800	-1.66548000
C	6.22778300	2.28785900	2.03757900
H	6.90722800	1.43101000	1.90488400
H	5.56668700	2.06264800	2.88632000
H	6.83953700	3.16357900	2.30961300
C	6.36970800	2.94263800	-0.40661900
H	6.90058900	3.88224900	-0.18198200
H	5.81405500	3.08013100	-1.34599000
H	7.12927400	2.16451400	-0.58049200
C	1.02350000	3.71979100	-2.20184800
H	0.31797300	2.98727600	-1.78580400
H	1.15282200	3.49088400	-3.27202300
H	0.55804500	4.71303900	-2.12227200
C	1.46938800	0.86486800	3.39328100
H	0.64034800	1.40546900	3.87585800
H	1.69466500	-0.02755000	3.99875900
H	1.11016400	0.52701400	2.41005700
C	3.21058400	2.20309900	4.64727100
H	4.05936900	2.90051600	4.56982400
H	3.54017700	1.32403600	5.22412000
H	2.42259200	2.69870500	5.23725700
C	3.33329500	4.74460500	-2.02713500
H	2.91148000	5.75727600	-1.91669100
H	3.51985300	4.57769600	-3.10079600
H	4.30163700	4.73064000	-1.50484100
N	-3.15307600	-1.54342900	0.44894700
C	-2.58067300	-2.75545000	0.95079800
C	-2.11704500	-3.73443200	0.04071100
C	-4.48310800	-1.44135600	0.31310100
C	-5.38366700	-2.64795400	0.62490100
H	-4.73811800	-3.45520800	0.99361300
C	-2.46848600	-2.94333200	2.35123300
C	-1.47397400	-5.12553900	1.93326900

H	-1.04412400	-6.05410300	2.31743600
C	-2.89527800	-1.83504200	3.30886300
H	-3.68276100	-1.25346800	2.80854500
C	-1.55547900	-4.91079500	0.55769800
H	-1.18064700	-5.67355500	-0.12672500
C	-1.92585300	-4.14647700	2.82023100
H	-1.83938800	-4.31951300	3.89450500
C	-2.21940100	-3.50143600	-1.46273100
H	-3.01958400	-2.76258100	-1.61959000
C	-6.40572200	-2.35035300	1.73365000
H	-7.14483000	-1.59580000	1.42184300
H	-5.91406100	-1.98172800	2.64677600
H	-6.95692600	-3.26844600	1.99429100
C	-6.06827300	-3.16924800	-0.65024800
H	-6.63542100	-4.08794000	-0.42835800
H	-5.33074100	-3.40835500	-1.43148500
H	-6.77109600	-2.43012000	-1.06601800
C	-0.91648300	-2.89552500	-2.01275600
H	-0.60861900	-1.98512600	-1.47569400
H	-1.02023900	-2.64132100	-3.07992600
H	-0.08647300	-3.60726300	-1.91269400
C	-1.72481100	-0.86874500	3.56680900
H	-0.91121800	-1.37469000	4.10912100
H	-2.04949000	-0.00180400	4.16454600
H	-1.28753500	-0.48560700	2.63156000
C	-3.48224100	-2.34611000	4.63022800
H	-4.29952600	-3.06343100	4.45552200
H	-3.88359900	-1.50569900	5.21895200
H	-2.72342500	-2.84454200	5.25483800
C	-2.60656600	-4.76182900	-2.24847900
H	-1.80167500	-5.51422600	-2.23673200
H	-2.79427200	-4.50773400	-3.30406400
H	-3.51610000	-5.23111800	-1.84073100
N	3.31563000	-1.29419000	-0.55347300
C	2.87785900	-2.61060500	-0.90075400
C	2.49003400	-3.49757700	0.13536600
C	4.61702000	-1.03123200	-0.40220100
C	5.65994000	-2.13747000	-0.62894600
H	5.14399500	-2.97943500	-1.10901600
C	2.79167500	-3.00735200	-2.25780300
C	2.01226700	-5.20181700	-1.53727900
H	1.68303000	-6.21427400	-1.78527000
C	3.14403000	-2.05821400	-3.39733500
H	3.49891100	-1.11860500	-2.94912500
C	2.05742300	-4.78541200	-0.20615300
H	1.74992900	-5.47597000	0.58086600
C	2.37016400	-4.31321200	-2.54926900
H	2.31120600	-4.63491100	-3.59201700
C	2.48798900	-3.03336400	1.58642400
H	3.21754700	-2.21463300	1.67280900
C	6.79617900	-1.70848400	-1.57080200
H	7.47461800	-0.98058200	-1.09896400
H	6.40604900	-1.25315300	-2.49331800
H	7.40151500	-2.58567800	-1.85166200
C	6.21160300	-2.64867600	0.71257600
H	6.92040300	-3.47634600	0.54593300
H	5.40658300	-3.01955300	1.36331100
H	6.74110100	-1.84806500	1.25357000
C	1.10614600	-2.46579900	1.94654600
H	0.79435500	-1.65136300	1.27530900
H	1.09548500	-2.08204900	2.97723600
H	0.34205200	-3.24795000	1.86585800
C	1.90132900	-1.71765300	-4.23788100
H	1.48005500	-2.62016600	-4.71125600
H	2.15969900	-1.00480900	-5.03822900
H	1.12258400	-1.25463900	-3.61540800
C	4.27477400	-2.61247700	-4.28045500
H	5.16132500	-2.87798000	-3.68527500
H	4.57936200	-1.86664800	-5.03286400
H	3.95574900	-3.51726400	-4.82339100
C	2.89609600	-4.12391800	2.58637300
H	2.13465900	-4.91711700	2.65980500
H	3.00836400	-3.69172100	3.59379800
H	3.85071500	-4.59756300	2.30772200
H	0.37779000	0.43206600	-1.44879100
H	1.09866700	0.61280800	-2.16708400

Transition state
B3LYP$_{PCM(benzene)}$/def2-TZVP//B3LYP/def2-SVP

173
-3270.945255

Mg -1.92335800 0.03788100 -0.00454000
O -0.05000200 0.08158600 -0.14322500
N -3.29455200 1.47368800 -0.59665400
C -5.20505100 0.08754800 -0.01462600
H -6.29267600 0.10320400 -0.01905200
C -2.77910900 2.73038700 -1.06700500
C -2.49294800 3.75400200 -0.12335600
C -4.61481800 1.26847500 -0.51265800
C -5.61113000 2.35208500 -0.96700000
H -5.02404900 3.17035000 -1.40458900
C -2.51382300 2.92914600 -2.44658200
C -1.68982000 5.16693700 -1.93841700
H -1.26931700 6.11659900 -2.27873700
C -2.81188500 1.86280700 -3.49954200
H -3.33656100 1.03811900 -2.99433900
C -1.94979300 4.96037000 -0.58439700
H -1.72545900 5.75587300 0.12882400
C -1.96806600 4.15591000 -2.85400500
H -1.76007400 4.32257200 -3.91379400
C -2.74361000 3.54621800 1.36962000
H -3.51301000 2.76636200 1.46612800
C -6.56963300 1.84514500 -2.05965200
H -7.29110400 1.11090900 -1.66832000
H -6.02891700 1.37042500 -2.89164100
H -7.14915300 2.68788900 -2.47086900
C -6.40060000 2.94246400 0.21537300
H -7.04369200 3.76678400 -0.13406000
H -5.73526000 3.34440600 0.99323100
H -7.05063100 2.18837900 0.68689100
C -1.47739500 3.02587000 2.07304200
H -1.06925300 2.12355500 1.58941300
H -1.68439500 2.78504800 3.12910500
H -0.67273500 3.77643000 2.04635700
C -1.52006500 1.28381200 -4.10404000
H -0.96337000 2.05360700 -4.66359000
H -1.75161800 0.46493900 -4.80528400
H -0.84117100 0.89559200 -3.33055200
C -3.74012100 2.38369500 -4.61207900
H -4.66621700 2.81747300 -4.20545700
H -4.01953300 1.56478000 -5.29531900
H -3.24857700 3.16107600 -5.21919900
C -3.27997000 4.79601600 2.08525200
H -2.52419500 5.59542800 2.14567200
H -3.56618200 4.54698000 3.12003700
H -4.16559700 5.21012500 1.57794300
Mg 1.90543100 0.09908200 -0.27472900
N 3.33724400 1.40490700 0.52690400
C 5.17003500 -0.09318200 -0.04474900
H 6.25667500 -0.13215400 -0.03217200
C 2.89448400 2.65231800 1.08358700
C 2.72255100 3.79172300 0.25163300
C 4.64259200 1.11612900 0.45447600
C 5.70209900 2.12441900 0.94266000
H 5.17319700 3.04919600 1.20953800
C 2.58146800 2.72920500 2.46909800
C 1.92508800 5.05895300 2.17882700
H 1.55287200 5.99457900 2.60445300
C 2.72765700 1.52142800 3.39303000
H 3.38178800 0.79717500 2.88652500
C 2.23143600 4.97559100 0.82321900
H 2.09679500 5.85597600 0.18988400
C 2.10371900 3.93952400 2.98992200
H 1.86511500 4.00840200 4.05370100
C 3.08319900 3.79027300 -1.23332100
H 3.51046700 2.80510600 -1.47045300
C 6.42311600 1.63094800 2.21012200
H 7.02272800 0.72923300 2.00829100
H 5.71661000 1.38769600 3.01640600
H 7.10638000 2.41045000 2.58590900
C 6.72436400 2.48371600 -0.15074400
H 7.34721700 3.33066600 0.18128000
H 6.23505800 2.77295900 -1.09206700
H 7.40345600 1.64560500 -0.37217900

C	1.84628700	3.98016400	-2.12684500
H	1.08936300	3.20595500	-1.94060500
H	2.12945800	3.92528700	-3.19128400
H	1.37554600	4.96156900	-1.95645900
C	1.36963700	0.83085700	3.60719400
H	0.66500400	1.50146000	4.12589800
H	1.48110200	-0.07967700	4.21832300
H	0.90544900	0.54482100	2.65103500
C	3.37873800	1.85995500	4.74437200
H	4.33199100	2.39596700	4.61708400
H	3.58064700	0.93688300	5.31198500
H	2.72604100	2.48732400	5.37274700
C	4.14850100	4.85301700	-1.56495000
H	3.75224100	5.87412500	-1.44037200
H	4.47598400	4.75557300	-2.61323900
H	5.03705600	4.76331500	-0.92251700
N	-3.33862100	-1.36044300	0.57277700
C	-2.87837700	-2.64286600	1.02731600
C	-2.58734000	-3.65170800	0.07077000
C	-4.65414500	-1.11254200	0.47505000
C	-5.68617800	-2.17768700	0.89529500
H	-5.12844200	-3.03046100	1.30361200
C	-2.68492800	-2.88499300	2.41244500
C	-1.89963000	-5.13068800	1.88177900
H	-1.52087400	-6.10042800	2.21486400
C	-2.98934700	-1.81888100	3.46351700
H	-3.61308400	-1.05218000	2.98177500
C	-2.09411900	-4.88327000	0.52372400
H	-1.86301700	-5.66747800	-0.19964500
C	-2.19559200	-4.13691700	2.81261500
H	-2.04463900	-4.33924200	3.87549700
C	-2.79399900	-3.40683600	-1.42333600
H	-3.53279400	-2.59769800	-1.51941900
C	-6.62593300	-1.67897500	2.00762100
H	-7.30345400	-0.88696200	1.65196900
H	-6.06795100	-1.27933300	2.86756900
H	-7.25264300	-2.51019300	2.37024800
C	-6.49337800	-2.70224200	-0.30604600
H	-7.15381200	-3.52515300	0.01311000
H	-5.83876000	-3.08789100	-1.10147800
H	-7.12751000	-1.91635400	-0.74590800
C	-1.49581600	-2.92189800	-2.09333600
H	-1.06179300	-2.04377800	-1.58796800
H	-1.67428500	-2.65471400	-3.14792800
H	-0.72217200	-3.70393800	-2.06768300
C	-1.70139100	-1.12025000	3.93294900
H	-1.02416300	-1.83178000	4.43298400
H	-1.92869500	-0.31110200	4.64654500
H	-1.14572100	-0.68208400	3.09017200
C	-3.78286400	-2.36091700	4.66395800
H	-4.69674500	-2.88577100	4.34504400
H	-4.08076700	-1.53416800	5.32931300
H	-3.18833200	-3.06381300	5.26960700
C	-3.36277000	-4.62338500	-2.17092700
H	-2.63699400	-5.45034000	-2.22908900
H	-3.61606800	-4.34638600	-3.20708300
H	-4.27492000	-5.01055400	-1.68983800
N	3.24799700	-1.46644500	-0.63350800
C	2.73231000	-2.74430200	-1.03472100
C	2.41597900	-3.70469300	-0.03401700
C	4.56823900	-1.27813500	-0.51748800
C	5.56102400	-2.39448600	-0.90052400
H	4.97634600	-3.21438300	-1.33809900
C	2.50399900	-3.03988000	-2.40493400
C	1.67730000	-5.24653800	-1.77117100
H	1.27619800	-6.22207600	-2.05856600
C	2.82382400	-2.05123800	-3.52520400
H	3.28242900	-1.16443500	-3.06373300
C	1.88963300	-4.94239300	-0.42696500
H	1.64545600	-5.68721300	0.33298600
C	1.98116700	-4.29771000	-2.74366500
H	1.81174100	-4.53839700	-3.79630600
C	2.61537100	-3.39670200	1.44901500
H	3.37355100	-2.60353200	1.51905100
C	6.57166600	-1.93833000	-1.96842200
H	7.29847300	-1.21459400	-1.56768400
H	6.07429700	-1.46789700	-2.82936400
H	7.14295100	-2.80521700	-2.33948500
C	6.29315000	-2.96637200	0.32662900

H 6.93233500 -3.81294600 0.02600600
H 5.59100700 -3.33193600 1.08967600
H 6.93958800 -2.21097800 0.80095900
C 1.32035100 -2.84410400 2.06976800
H 0.93631800 -1.97228500 1.51730400
H 1.48587000 -2.54020700 3.11658600
H 0.52357200 -3.60312500 2.05793400
C 1.55035500 -1.58052900 -4.25024700
H 1.04025300 -2.42064600 -4.75034800
H 1.79942500 -0.83362500 -5.02200200
H 0.84120700 -1.11456500 -3.55182900
C 3.83321600 -2.62673200 -4.53624300
H 4.74802900 -2.98895400 -4.04403900
H 4.12463600 -1.85743200 -5.27026600
H 3.40461800 -3.47116700 -5.10055800
C 3.13407300 -4.59198500 2.26427000
H 2.37935600 -5.38953500 2.35711700
H 3.38786900 -4.27097700 3.28767800
H 4.03599300 -5.03605800 1.81430900
H 0.38255100 0.54160100 -1.26523700
H 1.07085500 0.79330600 -1.94007200

Strain Analysis:
M06-D3$_{PCM(benzene)}$/def2-TZVP//M06-L-D3/def2-SVP

Int-2
171
-3266.235995

Mg -1.86172500 -0.17297600 -0.07760200
O -0.05128100 0.00657900 -0.14543000
N -2.99089100 -1.77544800 0.49611000
C -5.10747300 -0.67444500 0.13260700
H -6.18317000 -0.83974700 0.19821700
C -2.23229800 -2.91587900 0.87369800
C -1.80369300 -3.81695300 -0.12755400
C -4.32223500 -1.76309400 0.55617000
C -5.08498700 -2.97704700 1.08045300
H -4.33996200 -3.68032600 1.48575000
C -1.84188000 -3.09542100 2.21911900
C -0.53888100 -5.03405300 1.55210800
H 0.13442900 -5.85398600 1.81460300
C -2.29661700 -2.12585700 3.29011900
H -3.20666400 -1.62512900 2.91993800
C -0.94997900 -4.86296700 0.23441900
H -0.59856500 -5.55885700 -0.53132600
C -0.99153600 -4.15931600 2.53444000
H -0.67218100 -4.30223300 3.57096700
C -2.23210800 -3.62190600 -1.56768400
H -3.20357800 -3.09870100 -1.55235000
C -6.03316300 -2.61711400 2.21892600
H -6.87693800 -1.99897400 1.87810400
H -5.52461900 -2.06153600 3.02003200
H -6.45994600 -3.52681700 2.66636000
C -5.82215700 -3.69433900 -0.04553000
H -6.32515100 -4.59735200 0.33012800
H -5.14075700 -4.00666300 -0.84927100
H -6.59200900 -3.05072600 -0.49806700
C -1.24417000 -2.72308000 -2.30201300
H -1.04209800 -1.77399800 -1.77662900
H -1.59214300 -2.48330200 -3.31871100
H -0.26245100 -3.21473300 -2.38387100
C -1.24189200 -1.04370100 3.49329500
H -0.30012900 -1.47964800 3.86467600
H -1.57036000 -0.28502600 4.22027800
H -0.99512400 -0.52343600 2.55127700
C -2.65631900 -2.80365900 4.60432000
H -3.39016800 -3.61036600 4.46035600
H -3.08870300 -2.08017900 5.31075900
H -1.77794800 -3.24343700 5.10025200
C -2.42417300 -4.92820700 -2.32464800
H -1.46858000 -5.44728300 -2.49678500
H -2.86279100 -4.74174000 -3.31554700
H -3.08635400 -5.62445200 -1.78953700
Mg 1.76904800 0.09256500 0.05254600
N 3.40564600 -1.05236300 -0.45858200
C 4.95974100 0.79656600 -0.30187400
H 6.00948500 1.04254200 -0.46109900
C 3.10223200 -2.37381500 -0.89383800

C	3.11057200	-3.46627000	0.00809200
C	4.63671600	-0.54682700	-0.56070400
C	5.80995900	-1.42169200	-0.99510900
H	5.43771900	-2.45660300	-1.06358500
C	2.70299900	-2.55986900	-2.24156900
C	2.24362800	-4.89831700	-1.75869300
H	1.89911800	-5.88062600	-2.09237000
C	2.71983000	-1.40942300	-3.23388400
H	3.40834000	-0.64378900	-2.83990600
C	2.66468300	-4.71179300	-0.44693400
H	2.66120100	-5.56002700	0.24473600
C	2.28040100	-3.82921900	-2.64807100
H	1.96604300	-3.98092500	-3.68466700
C	3.68821800	-3.35537400	1.40906700
H	3.83098300	-2.28093000	1.62662600
C	6.32994000	-1.03341400	-2.37511400
H	6.72883000	-0.00758300	-2.38108400
H	5.54761600	-1.08524200	-3.14395200
H	7.14366900	-1.70483800	-2.68633900
C	6.93875400	-1.39630600	0.03189400
H	7.68282500	-2.17599600	-0.18987200
H	6.57325400	-1.56364400	1.05500300
H	7.47210800	-0.43422400	0.03118100
C	2.80028800	-3.94509400	2.50071500
H	1.78858400	-3.51906300	2.51356300
H	3.24902000	-3.77887600	3.49136600
H	2.68756900	-5.03413800	2.38417100
C	1.34981400	-0.74746100	-3.36329400
H	0.63219300	-1.43105100	-3.84369000
H	1.40828500	0.15762700	-3.98707500
H	0.90727500	-0.46239200	-2.39177400
C	3.24092000	-1.82133600	-4.60539200
H	4.20375800	-2.34949000	-4.54423400
H	3.38109700	-0.93967400	-5.24767900
H	2.53859000	-2.48505500	-5.13193900
C	5.05490500	-4.04028300	1.46567600
H	4.94506700	-5.12852700	1.33805600
H	5.54714700	-3.87214800	2.43550500
H	5.73707600	-3.69393800	0.67923500
N	-3.47502600	1.02077200	-0.43804200
C	-3.18637400	2.35390300	-0.83949700
C	-2.91848100	3.32523700	0.15078400
C	-4.73949600	0.59760600	-0.34248700
C	-5.89255000	1.52977600	-0.70962500
H	-5.46362000	2.38199900	-1.25906600
C	-3.12362600	2.68335700	-2.21272500
C	-2.57027700	4.96920500	-1.60640800
H	-2.33626500	5.99396400	-1.90587200
C	-3.32372000	1.60433700	-3.25710900
H	-3.98780600	0.83999700	-2.81980000
C	-2.60552200	4.62646400	-0.25817600
H	-2.39279500	5.38862200	0.49608600
C	-2.82508400	4.00004100	-2.57343300
H	-2.78147700	4.27175400	-3.63144300
C	-2.92298200	2.94368800	1.61805900
H	-3.63429500	2.10756700	1.73856500
C	-6.91617200	0.87885000	-1.63163200
H	-7.50112500	0.09473300	-1.12876300
H	-6.44318900	0.42070100	-2.51249500
H	-7.63452000	1.62889100	-1.99379700
C	-6.54866600	2.09013700	0.54816600
H	-7.34997600	2.79821300	0.29102200
H	-5.82617200	2.62491700	1.18084400
H	-6.99373400	1.28950600	1.15892600
C	-1.54295700	2.44026400	2.03476200
H	-1.14169900	1.64259700	1.38387300
H	-1.54579500	2.06730100	3.07061500
H	-0.80520200	3.25274300	1.97434500
C	-1.98921000	0.92299300	-3.55586300
H	-1.29751600	1.62319900	-4.04971300
H	-2.11454700	0.05307100	-4.21813100
H	-1.47133000	0.57939200	-2.64227400
C	-3.98795200	2.09833500	-4.53258700
H	-4.93580100	2.61646300	-4.32394700
H	-4.20800800	1.25858300	-5.20735600
H	-3.34728500	2.79457100	-5.09449000
C	-3.37478300	4.06700600	2.53995800
H	-2.64277100	4.88825000	2.57668700
H	-3.48503500	3.70034100	3.57055300

H -4.33927400 4.49662200 2.23170000
N 2.87665900 1.78329000 0.43869600
C 2.21334200 2.90725000 1.00589800
C 1.66115900 3.90839800 0.17630200
C 4.17532000 1.87709200 0.13862700
C 4.92135800 3.20184800 0.30228500
H 4.16984000 3.99029000 0.45849700
C 2.13148100 3.00598600 2.41413700
C 0.98532700 5.13261900 2.16590400
H 0.51430500 6.00891900 2.61855500
C 2.65182500 1.87342300 3.27766400
H 3.52083900 1.43475100 2.75754200
C 1.04294100 5.00838300 0.78042000
H 0.61494400 5.79404200 0.15248100
C 1.52328300 4.13414600 2.97337500
H 1.46595300 4.23377900 4.06031300
C 1.76202000 3.78768900 -1.33029000
H 2.70344100 3.25309500 -1.54715500
C 5.80683400 3.17300400 1.54362100
H 6.58030300 2.39304100 1.46766700
H 5.22565400 2.97387700 2.45506700
H 6.31788400 4.13685000 1.68316300
C 5.71613700 3.59079500 -0.93903400
H 6.10069300 4.61640600 -0.83882200
H 5.10005000 3.55603000 -1.84930400
H 6.58638100 2.93917700 -1.10694200
C 0.62014600 2.93848100 -1.88166200
H 0.54167600 1.94815300 -1.40196900
H 0.72849400 2.78988900 -2.96738000
H -0.34592900 3.43597600 -1.70775800
C 1.59313600 0.77698200 3.38315100
H 0.75615600 1.10230700 4.02112500
H 2.00007000 -0.15230400 3.81261700
H 1.13371600 0.53016900 2.40980800
C 3.11497100 2.30369300 4.66019600
H 3.85267300 3.11785300 4.61422300
H 3.57927400 1.46190700 5.19353300
H 2.28006300 2.65128600 5.28721700
C 1.83176300 5.12958200 -2.04345500
H 0.87747400 5.67579900 -1.98715000
H 2.04780000 4.98710500 -3.11208600
H 2.61485900 5.78175800 -1.62748900

Transition State
171
-3266.233178

Mg -1.87043600 0.06543300 0.05699700
O -0.04245500 0.24187900 -0.22011800
N -3.32922700 1.36478600 -0.50145100
C -5.09542500 -0.27627900 -0.32899300
H -6.16601000 -0.40221600 -0.47653600
C -2.89904900 2.66337200 -0.89177200
C -2.55661000 3.60862200 0.09804200
C -4.61399400 1.01409500 -0.61304400
C -5.65379600 2.06305900 -0.99969700
H -5.13584300 2.83122800 -1.59452600
C -2.76852200 2.97397500 -2.26854100
C -2.01968900 5.20263300 -1.65338100
H -1.68058300 6.19749800 -1.95254100
C -3.06809000 1.92325900 -3.32264900
H -3.91713400 1.31852300 -2.96093800
C -2.11218100 4.87270200 -0.30753500
H -1.83716100 5.61227300 0.44934800
C -2.33832900 4.25442100 -2.62256400
H -2.24134900 4.51722500 -3.67868500
C -2.60862600 3.24965600 1.57001300
H -3.32526500 2.41567000 1.68443000
C -6.80277900 1.54381200 -1.85127700
H -7.50238000 0.91243900 -1.28362800
H -6.44898700 0.95582200 -2.71076000
H -7.39060000 2.38555800 -2.24463600
C -6.16243400 2.75004600 0.26513500
H -6.88231800 3.54400000 0.01920500
H -5.34210800 3.21122100 0.83284800
H -6.66709800 2.03228000 0.93021600
C -1.23726700 2.76063500 2.02954400
H -0.84450400 1.91982900 1.42994600
H -1.25018800 2.45424600 3.08650900

H -0.48714100 3.55831100 1.92413600
C -1.88754200 0.96880600 -3.48727100
H -0.98703700 1.49669300 -3.83641700
H -2.11337100 0.17050200 -4.20999200
H -1.60611900 0.48098700 -2.54061400
C -3.48448100 2.50484900 -4.66452200
H -4.31563200 3.21857400 -4.56256200
H -3.81084400 1.70696800 -5.34650300
H -2.65688500 3.02879200 -5.16595800
C -3.08829900 4.38955200 2.45812200
H -2.35903700 5.21275700 2.49880300
H -3.22823500 4.04339000 3.49230000
H -4.04335700 4.81222400 2.11385600
Mg 1.87160500 0.14979100 -0.37141600
N 3.20883300 1.50951300 0.37797000
C 5.10699400 0.10490400 -0.16002600
H 6.19497000 0.12772200 -0.19240000
C 2.69899400 2.73506100 0.88531300
C 2.29231500 3.75839200 0.00223700
C 4.52213900 1.32003700 0.24639200
C 5.50505900 2.45604200 0.52752500
H 4.91284300 3.37392500 0.67017000
C 2.55546300 2.89587300 2.28562600
C 1.66984100 5.12915000 1.91291100
H 1.27807600 6.06729900 2.31448200
C 2.86293700 1.74424800 3.22221800
H 3.65279700 1.13221700 2.75533900
C 1.77420500 4.94338600 0.53975000
H 1.45842200 5.74110900 -0.14033900
C 2.05502200 4.10470900 2.77549700
H 1.95132500 4.24526400 3.85472600
C 2.42845200 3.62004000 -1.50029600
H 2.83665900 2.61755000 -1.71308300
C 6.29492900 2.21820200 1.80950200
H 6.90402800 1.30366700 1.74196000
H 5.64031300 2.11359900 2.68555900
H 6.97871300 3.05669900 2.00751900
C 6.43988800 2.70102600 -0.65319400
H 6.99138400 3.64329700 -0.51779700
H 5.89270900 2.76680600 -1.60440000
H 7.18926700 1.90339200 -0.76447000
C 1.07380800 3.71998200 -2.18951800
H 0.34009600 3.02476500 -1.75364300
H 1.15656200 3.48632700 -3.26187700
H 0.65699400 4.73491000 -2.10436100
C 1.63240000 0.85037800 3.35493700
H 0.81153500 1.38973000 3.85410300
H 1.85078600 -0.05081500 3.94839900
H 1.24642500 0.52370100 2.37459300
C 3.36286200 2.17647500 4.59221900
H 4.21270400 2.87107900 4.52287600
H 3.68918100 1.30576000 5.17898100
H 2.57909200 2.67630200 5.18137700
C 3.40536400 4.64748200 -2.06484900
H 3.02483700 5.67292800 -1.93653400
H 3.56549700 4.49292000 -3.14224400
H 4.38642800 4.60339800 -1.57023500
N -3.14179000 -1.47815200 0.46143600
C -2.60750900 -2.66191200 1.04373200
C -2.17776800 -3.72697500 0.21966300
C -4.44316800 -1.42556700 0.15257000
C -5.33435100 -2.65017300 0.35986800
H -4.67419800 -3.52089700 0.49187300
C -2.51328600 -2.74648600 2.45097400
C -1.60334600 -4.98592200 2.21894900
H -1.21938200 -5.90029400 2.67823300
C -2.89185200 -1.55453600 3.30850900
H -3.69821900 -1.01207500 2.78500300
C -1.67298300 -4.87850200 0.83196400
H -1.34138300 -5.71500500 0.21209400
C -2.01739000 -3.92491600 3.01855200
H -1.94898300 -4.01308300 4.10578900
C -2.28906700 -3.60778600 -1.28730500
H -3.21600000 -3.04458600 -1.49643700
C -6.15081900 -2.50935600 1.64061800
H -6.82633200 -1.64144700 1.59194100
H -5.50819200 -2.38026500 2.52281000
H -6.76815600 -3.40329400 1.81124200
C -6.22671600 -2.95297300 -0.83784400

H -6.71319300 -3.93090700 -0.70979000
H -5.65676100 -2.98673900 -1.77765600
H -7.03035500 -2.21341400 -0.96813800
C -1.12859600 -2.79957700 -1.86082400
H -1.02114800 -1.80386300 -1.40241500
H -1.24076900 -2.65274800 -2.94559900
H -0.17162000 -3.31394700 -1.68963700
C -1.71010400 -0.59415500 3.42682500
H -0.89367800 -1.04421900 4.01277600
H -1.99767900 0.34595600 3.92080400
H -1.25730300 -0.33425200 2.45156600
C -3.40948100 -1.92490700 4.68978800
H -4.23528100 -2.64906500 4.63965800
H -3.77737900 -1.03437100 5.21877600
H -2.62417000 -2.36546400 5.32216000
C -2.40534500 -4.94679800 -1.99826000
H -1.46875900 -5.52255200 -1.94435500
H -2.62101800 -4.79801900 -3.06572000
H -3.20823000 -5.57097800 -1.57812300
N 3.23025300 -1.37971900 -0.53749200
C 2.74469200 -2.67130000 -0.87805900
C 2.34018800 -3.54387000 0.16066900
C 4.54389900 -1.13893500 -0.49956200
C 5.53411200 -2.26457100 -0.79189700
H 4.99724200 -3.02143900 -1.38444700
C 2.62399800 -3.06730300 -2.23097800
C 1.76608600 -5.22424800 -1.49988300
H 1.39713200 -6.22436900 -1.74208600
C 2.99227400 -2.14849100 -3.38056700
H 3.39379100 -1.21411800 -2.95426900
C 1.85351200 -4.81148300 -0.17297700
H 1.54115700 -5.49170600 0.62379100
C 2.14185000 -4.35060800 -2.51402000
H 2.05461500 -4.66655300 -3.55832100
C 2.36406200 -3.08422200 1.60454900
H 3.15952200 -2.32450100 1.70398500
C 6.74249100 -1.83436600 -1.61304800
H 7.46112400 -1.23833900 -1.03130200
H 6.45399300 -1.23802700 -2.49086700
H 7.28856300 -2.71816100 -1.97456500
C 5.95920500 -2.93728600 0.50999000
H 6.65482000 -3.76664300 0.31496300
H 5.09908300 -3.34988900 1.05555500
H 6.46622400 -2.22333200 1.17754700
C 1.03574300 -2.41284300 1.93778700
H 0.81167500 -1.55172100 1.28612100
H 1.01036200 -2.06212300 2.98032000
H 0.20852200 -3.12340900 1.80228000
C 1.75640600 -1.77921500 -4.19676500
H 1.29757400 -2.67043000 -4.65442600
H 2.01462900 -1.08545400 -5.01059100
H 0.99544600 -1.28612300 -3.57493000
C 4.07078200 -2.75523900 -4.27324900
H 4.96861300 -3.04141700 -3.70667700
H 4.38246500 -2.04474600 -5.05304000
H 3.71007500 -3.65959500 -4.78727900
C 2.65511100 -4.19773000 2.60043800
H 1.82321000 -4.91535600 2.66974300
H 2.79706300 -3.78516400 3.60965800
H 3.56082400 -4.76502100 2.34033400

Int-3
171
-3266.184116

Mg 1.70274900 -0.07185000 -0.12495800
O -0.11200000 0.48934700 0.07937700
N 2.80738700 -1.63462400 0.57646300
C 3.83873800 -1.40320400 1.40278600
C 4.45065900 -0.14811500 1.56211500
H 5.31644800 -0.15857900 2.22182200
C 4.27042600 1.05256800 0.84762100
N 3.20006500 1.29854400 0.08958900
C 2.51253800 -2.97132900 0.18703200
C 2.96775600 -3.40460900 -1.08123600
C 2.70624700 -4.72579800 -1.46183700
H 3.05425100 -5.08833300 -2.43145900
C 1.97197300 -5.57783300 -0.64293400
H 1.77263400 -6.60405700 -0.96075200

C	1.46147700	-5.11048300	0.56438800
H	0.84884900	-5.77285100	1.18024900
C	1.71997000	-3.80825700	1.00211700
C	3.58314300	-2.41536800	-2.06009800
H	2.98882300	-1.48293800	-1.94971500
C	5.03087000	-2.03465000	-1.76479700
H	5.15175500	-1.56032300	-0.78185200
H	5.40679100	-1.32323300	-2.51693700
H	5.68614900	-2.91890200	-1.79953100
C	3.43786900	-2.84125500	-3.51371400
H	4.07861300	-3.70236000	-3.75605300
H	3.74396800	-2.02489900	-4.18431700
H	2.40294600	-3.11456900	-3.76790900
C	1.13527400	-3.28193100	2.29766000
H	1.84962800	-2.54710100	2.70524600
C	0.93705000	-4.35608000	3.35590300
H	0.13556700	-5.05957700	3.08286600
H	0.64417300	-3.90410400	4.31414600
H	1.85244300	-4.94243800	3.52749900
C	-0.16989000	-2.53637000	2.03462600
H	-0.04641900	-1.68921700	1.34087800
H	-0.58289700	-2.12286600	2.96704300
H	-0.92799700	-3.20487600	1.59803900
C	4.48497800	-2.54555000	2.19052700
H	3.83392100	-3.42625900	2.09694200
C	4.58076100	-2.21871600	3.67741900
H	4.88673100	-3.11088600	4.24304100
H	3.61914800	-1.87864400	4.08861300
H	5.32259100	-1.43493300	3.88977200
C	5.84286100	-2.94368200	1.62248500
H	5.75787600	-3.33976200	0.60181800
H	6.30271000	-3.72856400	2.24025500
H	6.54038900	-2.09174600	1.59476000
C	5.44005000	2.03293200	0.86065300
H	5.04647800	3.03088500	0.61554700
C	6.17209300	2.14746100	2.18911400
H	5.48356300	2.30600300	3.03181100
H	6.86738700	2.99830100	2.16511600
H	6.77566300	1.25659900	2.41723900
C	6.39533500	1.64404100	-0.26703500
H	6.82615000	0.64531700	-0.09345200
H	7.22593500	2.36043300	-0.34329400
H	5.88590200	1.62233000	-1.24170500
C	3.04558600	2.55592300	-0.55466900
C	2.71197800	3.70423700	0.20640300
C	2.58172200	4.92216500	-0.46859700
H	2.33398400	5.82544300	0.09190300
C	2.76531400	5.01367400	-1.84604600
H	2.66567300	5.97948200	-2.34665200
C	3.04402900	3.87066400	-2.58452400
H	3.15503900	3.94236000	-3.66954600
C	3.17799200	2.62786500	-1.95829500
C	2.52047300	3.61054900	1.71266500
H	3.40759200	3.09687000	2.12708400
C	1.30675700	2.76254600	2.09750200
H	0.41615800	3.05694000	1.51907000
H	1.05866300	2.89756000	3.16004500
H	1.48568900	1.68663500	1.95586400
C	2.44232000	4.96701800	2.39561200
H	3.30360300	5.60641300	2.15523100
H	2.41473200	4.84406300	3.48708300
H	1.52851400	5.51051500	2.11167600
C	3.38075000	1.36359300	-2.76885000
H	3.89108100	0.63301000	-2.11542700
C	2.02010500	0.78443500	-3.15659500
H	1.31025800	0.66416700	-2.31212200
H	2.10610400	-0.18687600	-3.66662900
H	1.47424100	1.46192100	-3.83033200
C	4.25093400	1.54389200	-4.00330900
H	3.77077400	2.18027000	-4.76118600
H	4.44650400	0.57472900	-4.48512500
H	5.22180100	1.99722300	-3.75713900
Mg	-1.78334700	0.26426400	-1.02285400
N	-2.97617800	1.70085000	-0.06052800
C	-4.24032900	1.64967100	-0.46669100
C	-4.84032700	0.48802700	-1.00532600
H	-5.84864300	0.64583200	-1.38329500
C	-4.42662500	-0.85559800	-1.00183300
N	-3.20138400	-1.26762600	-0.64716000

C	-2.44611100	2.87247800	0.53170000
C	-2.43670600	3.01761700	1.94152300
C	-1.90493200	4.18553700	2.49877300
H	-1.90656200	4.30546200	3.58620600
C	-1.36514900	5.18910500	1.70104500
H	-0.96143800	6.09747300	2.15551700
C	-1.34207300	5.02290000	0.31989200
H	-0.90942800	5.80482700	-0.31252300
C	-1.88541000	3.88478100	-0.28646800
C	-2.93898600	1.91435000	2.85048000
H	-3.56350300	1.24210000	2.23978400
C	-3.78996000	2.42314700	4.00692900
H	-4.60708700	3.07486700	3.66576900
H	-4.23894700	1.58368900	4.55821300
H	-3.19880500	2.99781100	4.73621200
C	-1.75814200	1.09771500	3.37275100
H	-1.06867000	1.73345500	3.95147000
H	-2.08941700	0.28306000	4.03430100
H	-1.18203200	0.64017400	2.55307000
C	-1.87005800	3.76693400	-1.79698400
H	-2.46472800	2.88362800	-2.08136900
C	-2.49743100	4.98036900	-2.47433600
H	-1.90007100	5.89221000	-2.31925100
H	-2.57134300	4.82602900	-3.56082900
H	-3.50967300	5.18632100	-2.09632000
C	-0.45082900	3.53286900	-2.29784500
H	0.01108300	2.65837300	-1.81114500
H	-0.43807700	3.34380800	-3.38165700
H	0.18873500	4.40425500	-2.09230200
C	-5.15634200	2.86490600	-0.34749300
H	-4.52965800	3.72237700	-0.05397500
C	-5.82311800	3.22776900	-1.66964200
H	-6.31548000	4.20870200	-1.59494400
H	-5.09867200	3.27911000	-2.49505800
H	-6.59812100	2.50321200	-1.95945800
C	-6.18637800	2.64856900	0.75571900
H	-5.70855500	2.40634500	1.71588200
H	-6.80189600	3.54787300	0.90646400
H	-6.86372500	1.81615900	0.50943900
C	-5.43768900	-1.87662400	-1.52826500
H	-5.20784400	-2.83891200	-1.04485100
C	-5.19924100	-2.06349000	-3.02490800
H	-4.16325000	-2.36578100	-3.23257500
H	-5.86412000	-2.83585700	-3.43873600
H	-5.38344700	-1.12808200	-3.57506500
C	-6.89752100	-1.57002900	-1.23050500
H	-7.28733500	-0.73375500	-1.82997900
H	-7.52457700	-2.44216000	-1.46685200
H	-7.06152900	-1.32087000	-0.17116400
C	-2.96780100	-2.63346700	-0.37215000
C	-2.08696100	-3.38340200	-1.18591600
C	-1.79752300	-4.70894100	-0.84366400
H	-1.10256400	-5.28048200	-1.46533100
C	-2.37927800	-5.31112000	0.26534900
H	-2.14904600	-6.35008400	0.51506100
C	-3.26698000	-4.57901500	1.05133900
H	-3.72788600	-5.05578900	1.92007000
C	-3.57406600	-3.24840400	0.75826600
C	-1.40664000	-2.74136100	-2.37450200
H	-1.98970700	-1.85385900	-2.67160000
C	-0.01825300	-2.26048900	-1.95918100
H	0.63202300	-3.12552800	-1.77394600
H	0.43382900	-1.61687800	-2.73029900
H	-0.06700100	-1.70284700	-1.00404300
C	-1.29369100	-3.64395700	-3.59430000
H	-2.27037400	-4.05010400	-3.89362000
H	-0.88861500	-3.08441100	-4.45025000
H	-0.61903200	-4.49752600	-3.42105300
C	-4.47965600	-2.44870800	1.67528400
H	-5.05043700	-1.73595000	1.05976700
C	-3.63199600	-1.60971800	2.62361700
H	-2.91326000	-0.99665900	2.06021400
H	-4.25336500	-0.93333900	3.23170600
H	-3.05920800	-2.25109600	3.31286000
C	-5.50017100	-3.28657900	2.42820800
H	-5.03350900	-3.94499700	3.17671300
H	-6.20243800	-2.63949900	2.97331400
H	-6.08981100	-3.92208000	1.75035000

Monomers:
M06-D3$_{PCM(benzene)}$/def2-TZVP//M06-L-D3/def2-SVP

[(^{iPrDip}nacnac)MgH]
86
-1596.716528

```
Mg -0.00526400 0.25188900 -1.28971300
N -1.48110700 0.02062800 0.09688800
N 1.48463900 -0.04075900 0.07051800
C -1.27144900 -0.32551800 1.37045800
C 0.00761600 -0.50405400 1.92580100
H 0.01600400 -0.77095900 2.98221900
C 1.28398500 -0.38019500 1.34418500
C -2.78964200 0.08505800 -0.45267800
C -3.45455200 -1.11621100 -0.80645200
C -4.69804300 -1.03172800 -1.43560800
H -5.22449700 -1.94831500 -1.71256400
C -5.27961100 0.20141900 -1.72192200
H -6.25164300 0.24679700 -2.21805400
C -2.79857600 -2.45894300 -0.54164500
H -2.20238700 -2.36418400 0.38083500
C -1.82217900 -2.81534400 -1.65975000
H -2.34268300 -2.93347200 -2.62196200
H -1.28785200 -3.75280500 -1.44567600
H -1.05859500 -2.03520500 -1.81676700
C -3.79045400 -3.58793000 -0.30714700
H -4.52000600 -3.33333900 0.47591500
H -3.26856700 -4.50292800 0.00740700
H -4.35606900 -3.84422600 -1.21546200
C -2.45901400 -0.48043800 2.31601200
H -3.34598100 -0.67621300 1.69254200
C -2.33974100 -1.63723900 3.29787700
H -2.07001600 -2.57908400 2.79784200
H -3.29793100 -1.79790300 3.81276400
H -1.58973600 -1.45361400 4.08126800
C 2.46740900 -0.67070700 2.26308700
H 3.38264400 -0.58540400 1.65600400
C 2.41445100 -2.09518200 2.80618800
H 3.33471600 -2.33258000 3.35985000
H 2.31028100 -2.83790600 2.00229500
H 1.57112300 -2.24152700 3.49743700
C 2.79182100 0.14318900 -0.45619100
C 3.46624500 -0.93478900 -1.06907700
C 4.70416600 -0.69114200 -1.67272300
H 5.23435700 -1.51555000 -2.15782400
C 5.27167200 0.57836800 -1.66666400
H 6.23979600 0.74890600 -2.14284400
C 2.85098100 -2.31965800 -1.10929500
H 2.05461400 -2.35004200 -0.34604500
C 2.19206400 -2.57111700 -2.46346400
H 1.44958000 -1.79648800 -2.71688600
H 1.68439000 -3.54687900 -2.49073800
H 2.93874200 -2.56098100 -3.27235200
C 3.84695100 -3.41970900 -0.76620400
H 4.63122100 -3.52213900 -1.53115800
H 3.34370800 -4.39509300 -0.69618100
H 4.34925300 -3.23232400 0.19425700
C -3.37038300 1.33978800 -0.73732500
C -4.61590900 1.37147600 -1.37607200
H -5.07209600 2.33796100 -1.60843600
C -2.65156100 2.63760100 -0.42038500
H -1.82916500 2.39996800 0.27814600
C -2.03148500 3.22353800 -1.68747400
H -2.81107300 3.48798100 -2.41845500
H -1.45566900 4.13591900 -1.47204000
H -1.36091400 2.51255800 -2.19879000
C -3.54959500 3.66274500 0.26149300
H -4.03634200 3.25620500 1.15956300
H -2.97204700 4.54789900 0.56514900
H -4.34740600 4.01885300 -0.40735500
C -2.70166800 0.84215800 3.03759600
H -2.85207600 1.66780800 2.32795800
H -3.59466000 0.78589000 3.67687300
H -1.84608300 1.10704900 3.67766200
C 2.57329800 0.34786100 3.39252200
H 3.46827700 0.15882300 4.00296400
H 2.64421400 1.37754000 3.01452000
```

H 1.70281300 0.30370800 4.06429100
C 3.36044900 1.43804400 -0.43719700
C 4.60217900 1.63103700 -1.04900300
H 5.05583900 2.62541200 -1.04378000
C 2.60869800 2.59433200 0.19461700
H 1.96842600 2.17557200 0.98932900
C 1.67967100 3.25499800 -0.82037900
H 0.98804200 2.53892800 -1.29733400
H 1.06553400 4.04122100 -0.35644600
H 2.24920100 3.71104500 -1.64430000
C 3.51649900 3.62958700 0.84235000
H 4.10578700 4.18928100 0.10069600
H 2.92536600 4.37107900 1.39866600
H 4.22523200 3.16959300 1.54600200
H 0.00704000 0.46363200 -2.97947900

[(^{iPrDip}nacnac)MgOH]
87
-1672.008499

Mg 0.00841500 0.01188200 -1.17891800
N 1.49037200 0.06212600 0.19690100
N -1.48441300 -0.04678100 0.18548000
C 1.28005100 0.00172000 1.51364700
C -0.00098100 0.01027700 2.09568000
H -0.00468500 0.01241400 3.18478100
C -1.27859100 0.01533600 1.50368000
C 2.79307500 0.00738900 -0.36565800
C 3.38846700 1.18706900 -0.86276400
C 4.62378400 1.08786400 -1.51364300
H 5.09315600 1.99295800 -1.90931100
C 5.26293900 -0.13665400 -1.66670700
H 6.22789500 -0.19265900 -2.17552900
C 2.69065900 2.52929900 -0.75130200
H 1.90178300 2.42874200 0.01496100
C 2.00788300 2.88667700 -2.07037500
H 2.75244700 3.01902500 -2.87097300
H 1.44077200 3.82597800 -1.98876000
H 1.31348100 2.10243100 -2.41629900
C 3.62255200 3.64909100 -0.30535100
H 4.15531800 3.40047100 0.62379500
H 3.06072300 4.57818200 -0.13135700
H 4.38420900 3.87806000 -1.06575300
C 2.46685200 -0.00754500 2.47167500
H 3.35321600 -0.29951900 1.88573600
C 2.71565700 1.40482500 2.99276900
H 2.86606200 2.11945100 2.17144200
H 3.61071600 1.43836000 3.63077800
H 1.86385900 1.76343900 3.59075200
C -2.47124400 0.02306600 2.45486100
H -3.34377200 0.36454300 1.87555800
C -2.32217500 0.97102700 3.63649600
H -3.27782100 1.06102300 4.17291400
H -2.02257700 1.98110200 3.32090500
H -1.58122500 0.62171400 4.37049900
C -2.79446300 -0.00686100 -0.36689100
C -3.44830600 1.24040300 -0.51225600
C -4.70059400 1.27078000 -1.12909700
H -5.22060100 2.22480600 -1.24600500
C -5.29730100 0.10465200 -1.60252500
H -6.27675600 0.14736800 -2.08421200
C -2.76494700 2.51310700 -0.05044200
H -2.14213500 2.25948400 0.82367900
C -1.81999400 3.03232000 -1.13215900
H -1.10591100 2.26694700 -1.47933700
H -1.23412200 3.89423900 -0.77956600
H -2.37731900 3.34432400 -2.02819100
C -3.72964900 3.60016600 0.39668800
H -4.32426200 4.00080200 -0.43790800
H -3.18334200 4.44971400 0.83078500
H -4.43379100 3.23162300 1.15735700
C 3.43321400 -1.24687400 -0.51726100
C 4.66776200 -1.29331600 -1.16840700
H 5.17552600 -2.25323600 -1.29152300
C 2.75702200 -2.51007700 -0.01977800
H 2.15208700 -2.23853200 0.86113700
C 1.78892200 -3.05158500 -1.06899500
H 2.32336900 -3.36666200 -1.97799700

H 1.22467000 -3.91704500 -0.69104400
H 1.05068200 -2.30016100 -1.39636500
C 3.73100700 -3.58824000 0.42942700
H 4.45293300 -3.20371200 1.16503400
H 3.19382200 -4.42676100 0.89516000
H 4.30578600 -4.00894700 -0.40919600
C 2.33422000 -1.00733100 3.61224200
H 2.08106300 -2.01509500 3.25177600
H 3.28188700 -1.08167600 4.16506600
H 1.56449400 -0.71672900 4.34211900
C -2.76737100 -1.40167200 2.91358600
H -3.66025600 -1.43211000 3.55473500
H -2.94799300 -2.07163200 2.06135200
H -1.92575200 -1.81645100 3.48943100
C -3.38448800 -1.19413700 -0.85011900
C -4.63873100 -1.11126200 -1.46609400
H -5.10588100 -2.02188300 -1.85108100
C -2.65565900 -2.52236800 -0.77775300
H -1.85820400 -2.42210600 -0.01989100
C -1.98596200 -2.82759400 -2.11738100
H -1.33028900 -2.01303400 -2.46908600
H -1.38968700 -3.75116800 -2.06571700
H -2.74332900 -2.96819700 -2.90424700
C -3.55212400 -3.67622600 -0.34696600
H -4.31891400 -3.90570900 -1.10199000
H -2.96392700 -4.59458300 -0.20610100
H -4.07683300 -3.46681400 0.59654000
O -0.04900600 0.00698700 -2.99863300
H 0.55986700 0.05136800 -3.73240200

[{(^{EtDip}nacnac)Mg}$_2$(μ-O)] + H_2 → [{(^{EtDip}nacnac)Mg}$_2$(μ-H))(μ-OH)]
M06-D3$_{PCM(benzene)}$/def2-TZVP//M06-L-D3/def2-SVP

[{(^{EtDip}nacnac)Mg}$_2$(μ-O)] **1b**
159
-3110.443093

Mg 1.81707800 -0.01374200 -0.02888000
O -0.00000200 -0.02143000 -0.00002300
N 3.19213000 1.44935500 0.34267600
C 5.04177200 0.16600100 -0.56288300
H 6.10948500 0.24187300 -0.77803800
C 2.72721800 2.67169300 0.90420500
C 2.33346000 3.73200800 0.05502200
C 4.47183100 1.33516900 -0.01878600
C 5.43402100 2.48759800 0.18037500
H 4.92137500 3.44438500 -0.00209500
C 2.62796100 2.79442900 2.30838600
C 1.82509800 5.06910100 2.02133600
H 1.48536200 6.01167500 2.45784700
C 2.93202100 1.60755200 3.20137500
H 3.68624400 0.98884700 2.68408600
C 1.88500900 4.92175300 0.63796000
H 1.58558300 5.75449800 -0.00308700
C 2.18537400 4.00791500 2.84559500
H 2.11556100 4.12201300 3.93047600
C 2.37927400 3.55647700 -1.45007400
H 3.26129200 2.93350700 -1.68205400
C 6.06393000 2.50823000 1.56469700
H 6.55001400 1.54965300 1.79877500
H 5.31764900 2.70297400 2.34703600
H 6.82560200 3.29623000 1.63973400
C 1.14383400 2.80330000 -1.93820900
H 0.96990100 1.84647900 -1.41767500
H 1.20403100 2.59672100 -3.01787600
H 0.23563800 3.39807100 -1.76179500
C 1.67686900 0.75268900 3.36861200
H 0.90544500 1.30320700 3.93006200
H 1.89003100 -0.17522100 3.92067700
H 1.20450900 0.47648300 2.40819600
C 3.50138000 1.98545900 4.56056700
H 4.37770300 2.64450700 4.47575200
H 3.81194300 1.08778100 5.11389800
H 2.76146700 2.50232600 5.19009200
C 2.53617300 4.86248200 -2.21358700
H 1.63548900 5.49114400 -2.14019700

H 2.69538900 4.66660700 -3.28337100
H 3.38874200 5.45594100 -1.85150000
Mg -1.81708100 -0.01368600 0.02886900
N -3.19204900 1.44950100 -0.34266700
C -5.04175700 0.16625500 0.56291600
H -6.10946400 0.24218800 0.77807900
C -2.72707600 2.67181100 -0.90420400
C -2.33326400 3.73211200 -0.05502700
C -4.47175400 1.33538800 0.01880700
C -5.43388500 2.48786600 -0.18035400
H -4.92119100 3.44462900 0.00210400
C -2.62781900 2.79453600 -2.30838500
C -1.82484900 5.06917100 -2.02135100
H -1.48507400 6.01172800 -2.45786700
C -2.93194900 1.60767100 -3.20136700
H -3.68620200 0.98901000 -2.68406900
C -1.88476100 4.92183300 -0.63797300
H -1.58529500 5.75456900 0.00306700
C -2.18517800 4.00799800 -2.84560300
H -2.11536600 4.12208800 -3.93048500
C -2.37908400 3.55659200 1.45007100
H -3.26114100 2.93368100 1.68205900
C -6.06380000 2.50851700 -1.56467300
H -6.54994200 1.54996600 -1.79873600
H -5.31751000 2.70320600 -2.34701800
H -6.82542500 3.29656200 -1.63971800
C -1.14369200 2.80333600 1.93820800
H -0.96983400 1.84649500 1.41768700
H -1.20389700 2.59677700 3.01787800
H -0.23545400 3.39803800 1.76177500
C -1.67684600 0.75273700 -3.36860900
H -0.90539400 1.30321000 -3.93006600
H -1.89006000 -0.17516300 -3.92067000
H -1.20448900 0.47651100 -2.40819700
C -3.50129500 1.98560200 -4.56055700
H -4.37758100 2.64470000 -4.47574100
H -3.81191200 1.08793900 -5.11388100
H -2.76135600 2.50242300 -5.19008900
C -2.53589400 4.86261100 2.21357800
H -1.63517100 5.49121600 2.14017700
H -2.69511300 4.66675200 3.28336400
H -3.38842900 5.45612200 1.85149600
N 3.17859400 -1.37986800 -0.69602000
C 2.67658600 -2.64584100 -1.10602800
C 2.36742800 -3.62111400 -0.13198200
C 4.47060800 -1.08583600 -0.86367600
C 5.43679600 -2.13892300 -1.36337000
H 4.97887700 -2.72259200 -2.17655900
C 2.43348700 -2.89350700 -2.47883000
C 1.63098100 -5.11435500 -1.90259000
H 1.22927100 -6.08181300 -2.21474300
C 2.65670400 -1.79767400 -3.50375500
H 3.50674200 -1.18314900 -3.16343400
C 1.83822800 -4.84604800 -0.55387500
H 1.59222200 -5.60786900 0.19055200
C 1.92632600 -4.14074300 -2.85364800
H 1.74285200 -4.35119700 -3.91031200
C 2.57006200 -3.32753700 1.34110200
H 3.37928900 -2.57887600 1.41598000
C 5.88872600 -3.08551400 -0.26162500
H 6.65138700 -3.78500900 -0.62991700
H 5.05332100 -3.68764400 0.12170300
H 6.32050800 -2.53571700 0.58752400
C 1.30640300 -2.71177900 1.93695400
H 0.92498500 -1.83854800 1.37660300
H 1.46657700 -2.40709300 2.98255900
H 0.48172400 -3.44003600 1.92612700
C 1.43753700 -0.87874500 -3.56018000
H 0.56434000 -1.41561300 -3.96306500
H 1.61972400 -0.00609400 -4.20554200
H 1.13507800 -0.50554900 -2.56598300
C 3.00863000 -2.31393600 -4.89026400
H 3.86052700 -3.00926600 -4.86595800
H 3.27576600 -1.48185000 -5.55699800
H 2.16633700 -2.83944400 -5.36499100
C 2.99395700 -4.54380900 2.15242400
H 2.18433200 -5.28529100 2.23315800
H 3.25407700 -4.25151000 3.17984600
H 3.86697900 -5.05300200 1.71838200

N -3.17866800 -1.37972200 0.69603200
C -2.67672400 -2.64572500 1.10602500
C -2.36761900 -3.62100200 0.13196700
C -4.47066300 -1.08561600 0.86370100
C -5.43690500 -2.13864900 1.36340400
H -4.97901000 -2.72233900 2.17659200
C -2.43362700 -2.89341700 2.47882300
C -1.63122700 -5.11429500 1.90255300
H -1.22955800 -6.08177400 2.21469300
C -2.65678500 -1.79758500 3.50376200
H -3.50680000 -1.18301800 3.16345700
C -1.83847100 -4.84596400 0.55384300
H -1.59250200 -5.60778800 -0.19059500
C -1.92652100 -4.14068000 2.85362500
H -1.74305100 -4.35115300 3.91028500
C -2.57025000 -3.32740000 -1.34111400
H -3.37942600 -2.57868400 -1.41597600
C -5.88888600 -3.08522200 0.26166400
H -6.65158700 -3.78467200 0.62996000
H -5.05351500 -3.68740000 -0.12166100
H -6.32063900 -2.53540500 -0.58748800
C -1.30655500 -2.71172600 -1.93697700
H -0.92506300 -1.83853200 -1.37661800
H -1.46672500 -2.40701000 -2.98257400
H -0.48193000 -3.44004500 -1.92617700
C -1.43757600 -0.87871000 3.56018000
H -0.56440000 -1.41561800 3.96305500
H -1.61971800 -0.00605300 4.20554600
H -1.13511000 -0.50552200 2.56598200
C -3.00871700 -2.31384600 4.89026900
H -3.86064400 -3.00914000 4.86596500
H -3.27580900 -1.48175700 5.55701600
H -2.16644000 -2.83939600 5.36497900
C -2.99424000 -4.54363800 -2.15243800
H -2.18466900 -5.28517700 -2.23318400
H -3.25435200 -4.25131500 -3.17985500
H -3.86729200 -5.05277300 -1.71838800
H -6.22489900 2.41146200 0.58001400
H -6.31129000 -1.63475900 1.79982200
H 6.31121000 -1.63508000 -1.79978600
H 6.22503600 2.41114700 -0.57998700

H_2
2
-1.170703414

H 0.00000000 0.00000000 0.37726500
H 0.00000000 0.00000000 -0.37726500

Int-1
161
-3111.614704

Mg -1.85042500 0.01642200 -0.04527200
O -0.03514300 -0.00827800 -0.09206700
N -3.19072700 1.47494800 -0.50420100
C -5.12202600 0.19114700 0.19309900
H -6.20487200 0.23947700 0.29413000
C -2.67892600 2.70296100 -1.00868000
C -2.30343800 3.72323500 -0.10604300
C -4.51013700 1.34714600 -0.32546600
C -5.39121600 2.52249700 -0.69474700
H -5.17667400 2.77531900 -1.74807100
C -2.53323700 2.88054200 -2.40276900
C -1.69636400 5.12493300 -1.99701500
H -1.32238900 6.07627200 -2.38387200
C -2.84582200 1.73858900 -3.34918400
H -3.63505700 1.12641500 -2.87948000
C -1.80980600 4.92543000 -0.62394100
H -1.51824900 5.72570900 0.06105500
C -2.04860500 4.10420500 -2.87550700
H -1.93905400 4.26070600 -3.95182000
C -2.40856600 3.49225900 1.38834100
H -3.26873500 2.82023600 1.55735000
C -6.88354500 2.39403900 -0.49270900
H -7.14678200 2.21877500 0.55988500
H -7.31348200 1.57142600 -1.08132700
H -7.39261900 3.31572700 -0.80432600
C -1.15867700 2.77978900 1.90079300

H	-0.91520200	1.85661200	1.34607100
H	-1.25138300	2.52346800	2.96739800
H	-0.27562200	3.42599000	1.78892900
C	-1.61508100	0.84632400	-3.50669000
H	-0.80262900	1.39137700	-4.01326900
H	-1.83726700	-0.05042800	-4.10496600
H	-1.19695700	0.51369700	-2.53943400
C	-3.36660700	2.18811100	-4.70557100
H	-4.22691200	2.86680300	-4.61105000
H	-3.68721700	1.32386600	-5.30452800
H	-2.59742700	2.71051500	-5.29421400
C	-2.66142200	4.76257800	2.18622300
H	-1.79090400	5.43629300	2.17444100
H	-2.85771400	4.52344000	3.24105300
H	-3.52486100	5.32742100	1.80449700
Mg	1.78017100	0.02431100	-0.14885300
N	3.16976400	1.39724900	0.43009000
C	5.04624600	-0.02149200	-0.16942400
H	6.13075500	-0.02550100	-0.20722600
C	2.71583400	2.62149100	0.99902100
C	2.36208700	3.70213500	0.15893800
C	4.48207900	1.18247600	0.30115700
C	5.42278100	2.30250200	0.69790400
H	5.08822000	3.21339800	0.17102300
C	2.60209400	2.73452100	2.40345600
C	1.82252000	5.01938300	2.12994900
H	1.48079500	5.95870500	2.57186800
C	2.89572100	1.54311200	3.29295800
H	3.64393000	0.91598000	2.77852500
C	1.91378300	4.88964200	0.74739600
H	1.64204900	5.73477000	0.11019200
C	2.16010800	3.94472000	2.94685500
H	2.07294100	4.04636800	4.03174800
C	2.46969400	3.55935700	-1.34518400
H	3.35032500	2.92340300	-1.54046900
C	6.90684700	2.10484200	0.48902700
H	7.45921500	3.00328700	0.79462900
H	7.15573500	1.91348100	-0.56447800
H	7.30368000	1.26645800	1.07882500
C	1.24624400	2.84617700	-1.91580000
H	1.00356000	1.89098600	-1.41841500
H	1.36146700	2.64670800	-2.99346000
H	0.34574200	3.46456200	-1.78850900
C	1.63244600	0.69989600	3.45221100
H	0.85286200	1.26589700	3.98666900
H	1.82946600	-0.21882400	4.02530500
H	1.19098200	0.40575400	2.48341400
C	3.47427500	1.91650300	4.64940300
H	4.35679100	2.56618700	4.55550600
H	3.77945300	1.01640200	5.20179400
H	2.74418400	2.44170900	5.28343800
C	2.69053400	4.87716400	-2.07243800
H	1.80045600	5.52367600	-2.03177000
H	2.90010700	4.69894700	-3.13679800
H	3.53533500	5.44495100	-1.65560200
N	-3.26202100	-1.32561000	0.56174000
C	-2.79297300	-2.58109300	1.03892800
C	-2.46423000	-3.59572100	0.11228300
C	-4.56293100	-1.03297000	0.61538300
C	-5.55803200	-2.06029800	1.11376300
H	-5.14225300	-2.60025100	1.97839700
C	-2.60446600	-2.77946600	2.42707000
C	-1.81516300	-5.03041300	1.96375000
H	-1.43903600	-5.99067800	2.32590200
C	-2.85324200	-1.64256500	3.39935200
H	-3.66983600	-1.02316500	2.99149700
C	-1.97043200	-4.81119900	0.59888500
H	-1.71028700	-5.60488700	-0.10625100
C	-2.12895900	-4.01803200	2.86678100
H	-1.98662600	-4.19107900	3.93654700
C	-2.61702800	-3.35172300	-1.37604800
H	-3.43489600	-2.61991300	-1.50230500
C	-5.96512800	-3.06349100	0.04524100
H	-6.76053500	-3.72716500	0.41067900
H	-5.12247400	-3.70151500	-0.25438100
H	-6.33925700	-2.55882400	-0.85768900
C	-1.34710900	-2.72942400	-1.95149300
H	-0.98885100	-1.84242800	-1.39834800
H	-1.48904700	-2.44315100	-3.00497100

H -0.51304300 -3.44603400 -1.90984600
C -1.61592400 -0.75065500 3.48606300
H -0.77560100 -1.29324400 3.94698000
H -1.80658900 0.14777100 4.09235500
H -1.25487100 -0.41761800 2.49687100
C -3.28710600 -2.09990900 4.78331300
H -4.15308700 -2.77654000 4.73944800
H -3.56731700 -1.23864300 5.40619600
H -2.48268700 -2.62672300 5.31841800
C -2.99160200 -4.59922400 -2.16347000
H -2.16741800 -5.32840400 -2.19283000
H -3.21954500 -4.34258900 -3.20790900
H -3.87115200 -5.11063200 -1.74529500
N 3.12392700 -1.43681600 -0.59700500
C 2.61571800 -2.71636400 -0.96121200
C 2.28441300 -3.64524200 0.04954900
C 4.44324200 -1.24178100 -0.54030700
C 5.34531000 -2.40949400 -0.88440600
H 5.05478700 -2.77232800 -1.88579900
C 2.40824000 -3.03146000 -2.32437000
C 1.56758400 -5.21366000 -1.66367300
H 1.16152400 -6.19074200 -1.93803200
C 2.68367900 -1.99593700 -3.39541300
H 3.51699300 -1.36813800 -3.03841000
C 1.75094800 -4.88329200 -0.32522700
H 1.47951800 -5.60554900 0.44935300
C 1.89603300 -4.29016500 -2.65213600
H 1.73752800 -4.54902800 -3.70218900
C 2.45124800 -3.28684600 1.51198200
H 3.24069100 -2.51748800 1.57862600
C 6.84193000 -2.20482800 -0.82828900
H 7.18573400 -1.91898300 0.17586900
H 7.18194300 -1.42701400 -1.52675400
H 7.36546800 -3.13163700 -1.09776600
C 1.15698300 -2.67451800 2.04125700
H 0.80846500 -1.81052700 1.44672000
H 1.26623400 -2.35130900 3.08769500
H 0.34073500 -3.41133900 2.00376100
C 1.47072200 -1.08300300 -3.56617500
H 0.61587000 -1.64623900 -3.97248700
H 1.67969500 -0.25283400 -4.25929500
H 1.12007700 -0.64683300 -2.61293900
C 3.10546100 -2.58727800 -4.73181000
H 3.95076400 -3.28275600 -4.62447000
H 3.41274400 -1.79307500 -5.42708600
H 2.28718500 -3.13555900 -5.22248700
C 2.88692200 -4.45973400 2.37867500
H 2.09809800 -5.22220800 2.46880200
H 3.11421000 -4.12242800 3.40009700
H 3.78486000 -4.95685500 1.98277500
H 3.13211700 0.97081800 -2.72895300
H 3.77077100 0.78620000 -2.35595200
H -5.01305400 3.40281200 -0.14555800
H -6.44988300 -1.53186200 1.48054400
H 5.05588700 -3.24554600 -0.22311800
H 5.22343400 2.54018600 1.75771600

Transition state
161
-3111.590684

Mg -1.88219700 -0.02880900 0.01307100
O -0.06576100 0.23014100 -0.24739900
N -3.35675900 1.27825600 -0.45026700
C -5.14566200 -0.17886200 0.26811100
H -6.22678800 -0.23713000 0.38178600
C -2.95503600 2.53271800 -0.99136800
C -2.62158000 3.59019200 -0.11630500
C -4.65473600 1.03256700 -0.25421100
C -5.64343000 2.12369800 -0.60539800
H -5.47146100 2.39705400 -1.66125200
C -2.86621300 2.69167800 -2.39231000
C -2.11455600 4.97552300 -2.04484400
H -1.78425700 5.93160000 -2.45828400
C -3.20085800 1.53680100 -3.31761200
H -3.98369200 0.93457600 -2.82362300
C -2.19593300 4.80305300 -0.66708900
H -1.92900600 5.63060600 -0.00481600
C -2.44458300 3.92608000 -2.89629900

H -2.37163200 4.06949200 -3.97728700
C -2.71284300 3.39464300 1.38422800
H -3.53138600 2.67678900 1.57104600
C -7.11379900 1.86035800 -0.37574200
H -7.33954800 1.65821100 0.68072000
H -7.47895400 1.00471800 -0.96099000
H -7.71001900 2.73323100 -0.67262100
C -1.42736800 2.77702900 1.92791800
H -1.11746100 1.86032900 1.39823100
H -1.51901900 2.53763700 2.99848300
H -0.58091700 3.46974500 1.80925000
C -1.99007000 0.62650300 -3.50642600
H -1.15278100 1.16507100 -3.97727600
H -2.23221600 -0.24190700 -4.13760300
H -1.59575100 0.23907300 -2.55320500
C -3.75392600 1.97812100 -4.66443800
H -4.59881100 2.67359100 -4.55431000
H -4.10658600 1.11149700 -5.24134800
H -2.99274600 2.47816900 -5.28181100
C -3.04674700 4.66936100 2.14477600
H -2.22030100 5.39559300 2.11373800
H -3.23150700 4.44967000 3.20592700
H -3.94211000 5.16727600 1.74455600
Mg 1.84498700 0.22870400 -0.39606400
N 3.13231500 1.54970900 0.47455900
C 5.07561900 0.12678700 0.21230300
H 6.15253100 0.11799300 0.34496700
C 2.58643800 2.78589100 0.91133400
C 2.20157700 3.75938700 -0.03578700
C 4.44944900 1.34070400 0.55454700
C 5.32113200 2.47855600 1.04878700
H 5.03377700 3.37930600 0.48005100
C 2.37593600 3.00770700 2.29542400
C 1.49363300 5.21353000 1.78139800
H 1.07972400 6.16610100 2.12210100
C 2.64376600 1.89722600 3.29309900
H 3.46971500 1.27995100 2.90216400
C 1.65441300 4.96457900 0.42419600
H 1.35761200 5.72555500 -0.30445000
C 1.84603600 4.23213900 2.70694300
H 1.68848500 4.41971900 3.77236300
C 2.37734900 3.54086400 -1.52555300
H 2.71855100 2.50299300 -1.67756100
C 6.82220100 2.30184600 1.00674800
H 7.32514100 3.21700400 1.34663900
H 7.18535100 2.08765700 -0.00852000
H 7.16604400 1.48584400 1.65802300
C 1.05873200 3.69757900 -2.27108700
H 0.26506800 3.07759000 -1.82697000
H 1.16279700 3.39498800 -3.32408800
H 0.70687400 4.74075200 -2.26191000
C 1.42021000 0.98767700 3.38432000
H 0.56458000 1.52828400 3.81931300
H 1.61720800 0.10865500 4.01698500
H 1.09827300 0.62567600 2.39353900
C 3.05876100 2.39055200 4.67049400
H 3.91219700 3.08265000 4.62002700
H 3.35008600 1.54775400 5.31344800
H 2.24111300 2.91376400 5.18904400
C 3.45445000 4.45399900 -2.10281200
H 3.18671600 5.51567600 -1.98366100
H 3.59462000 4.26993400 -3.17817900
H 4.42765100 4.30579300 -1.61238400
N -3.13968800 -1.51406900 0.60866000
C -2.56532300 -2.73422100 1.06673200
C -2.15035900 -3.70483700 0.12818800
C -4.46618500 -1.34732700 0.66553000
C -5.35728300 -2.47761100 1.13712900
H -4.88805800 -3.00945500 1.97867300
C -2.37263800 -2.93761800 2.45446800
C -1.41135900 -5.11409000 1.96675000
H -0.96962000 -6.04977800 2.31861400
C -2.72568800 -1.83695000 3.43663000
H -3.61897000 -1.31645800 3.05175700
C -1.56845200 -4.88612400 0.60374500
H -1.24001900 -5.64585600 -0.11014400
C -1.80790800 -4.14213400 2.88223100
H -1.66421300 -4.32176700 3.95047400
C -2.29569900 -3.45490400 -1.36055000

H -3.14897500 -2.76598700 -1.49622600
C -5.68355300 -3.47297400 0.03397700
H -6.40445600 -4.22484200 0.38247900
H -4.78821000 -4.01296000 -0.30304100
H -6.11968900 -2.97152300 -0.84236900
C -1.04698400 -2.76597900 -1.90505100
H -0.78909000 -1.83338800 -1.37528800
H -1.15376300 -2.52533300 -2.97356500
H -0.16777400 -3.41791000 -1.79684100
C -1.59957300 -0.80686000 3.49981600
H -0.69212600 -1.24303500 3.94456100
H -1.88115000 0.06886100 4.10348900
H -1.29786300 -0.43563000 2.50419100
C -3.06795700 -2.33948900 4.83019600
H -3.85322400 -3.10895100 4.80664300
H -3.42707500 -1.51520900 5.46225000
H -2.19539700 -2.77234300 5.34208500
C -2.58896300 -4.71477700 -2.16332400
H -1.72281300 -5.39320000 -2.19309700
H -2.82260400 -4.46072800 -3.20689800
H -3.44000400 -5.28177600 -1.75830300
N 3.23501000 -1.26107500 -0.53479600
C 2.78793100 -2.52321500 -1.01024100
C 2.43526400 -3.52898000 -0.07691700
C 4.52919000 -1.08357600 -0.25923000
C 5.46485200 -2.25989700 -0.45731200
H 5.28339500 -2.66557900 -1.46640400
C 2.66459400 -2.75623400 -2.39839500
C 1.94135800 -5.03084000 -1.92314800
H 1.62688400 -6.01499300 -2.28002600
C 2.97147400 -1.68518600 -3.42873500
H 3.16055200 -0.74337000 -2.88700800
C 2.01477000 -4.77250800 -0.55543300
H 1.74588200 -5.55925600 0.15392200
C 2.25403400 -4.02462800 -2.82881100
H 2.17517500 -4.22160000 -3.90244800
C 2.48176700 -3.23308000 1.40960200
H 3.34466600 -2.56815800 1.59182600
C 6.94665700 -2.04221800 -0.25146000
H 7.18609400 -1.73828800 0.77733400
H 7.34899900 -1.27132900 -0.92428400
H 7.50127900 -2.96879700 -0.45106000
C 1.22885100 -2.47275400 1.83460400
H 1.09851500 -1.52078800 1.29685300
H 1.24753100 -2.24385000 2.91103800
H 0.32642400 -3.06819100 1.63247400
C 1.78189900 -1.44066000 -4.35101900
H 1.54271600 -2.32807400 -4.95823000
H 1.99194800 -0.61538000 -5.04743600
H 0.88342200 -1.16467300 -3.78107500
C 4.22609200 -2.01601600 -4.23168600
H 5.11073600 -2.13455400 -3.58941900
H 4.45190400 -1.22142000 -4.95795800
H 4.10627700 -2.95293300 -4.79824400
C 2.66593300 -4.46949500 2.27571800
H 1.77630900 -5.11767100 2.25920600
H 2.82776200 -4.18473300 3.32505400
H 3.52667500 -5.07560300 1.95667500
H 0.32986400 0.48656400 -1.40422000
H 1.05055100 0.62361500 -2.13589800
H -5.33391700 3.03264100 -0.05959100
H -6.28939300 -2.04470400 1.52801800
H 5.11327700 -3.07276600 0.20304500
H 5.00624100 2.72318300 2.07809200

Int-2
161
-3111.613202

Mg 1.72564800 -0.37744100 0.14050300
O 0.08148400 0.49474600 -0.28849300
N 3.48235800 0.56419500 0.53480000
C 4.86098100 -1.34396300 0.00123500
H 5.89223600 -1.69115900 -0.02263500
C 3.40452400 1.92349900 0.95036700
C 3.44536600 2.95041800 -0.01993000
C 4.67958900 -0.01951800 0.43684400
C 5.89935000 0.78572700 0.83289600
H 5.75179300 1.10451200 1.88020200

C	3.27330900	2.21692300	2.32583500
C	3.18119200	4.57515700	1.77111900
H	3.08395800	5.61610100	2.08964300
C	3.21618100	1.08837400	3.33655300
H	3.83859700	0.26510500	2.94422300
C	3.31528400	4.27301400	0.42001900
H	3.33106800	5.08844300	-0.30583200
C	3.17019100	3.55433200	2.71520000
H	3.06491100	3.80219100	3.77443200
C	3.69427300	2.62325900	-1.48532600
H	4.54406300	1.91430700	-1.51117300
C	7.26016200	0.14642300	0.67778200
H	7.46778400	-0.13865400	-0.36323200
H	7.37011800	-0.75567700	1.29565300
H	8.04828700	0.84595100	0.98576500
C	2.52058600	1.91724300	-2.16346500
H	2.41632300	0.86838200	-1.84594700
H	2.66522800	1.87926000	-3.25338500
H	1.57129800	2.44452500	-1.98037000
C	1.79137600	0.55764100	3.45998700
H	1.12389300	1.29124100	3.93620100
H	1.74555900	-0.36854900	4.05289900
H	1.30586400	0.35739900	2.48764000
C	3.77110600	1.46039000	4.70274700
H	4.78595000	1.87865500	4.63390800
H	3.81647500	0.57792700	5.35639500
H	3.14148100	2.20100200	5.21786800
C	4.09755800	3.83687600	-2.30977000
H	3.25852000	4.53878500	-2.43358200
H	4.40787700	3.52975500	-3.31805300
H	4.93273700	4.39045900	-1.85832700
Mg	-1.68287900	0.61779000	0.71846300
N	-2.54384700	2.14572800	-0.42230100
C	-4.80292200	1.28407800	-0.36508500
H	-5.83186300	1.55421100	-0.57902100
C	-1.64855200	3.15218400	-0.85196800
C	-1.05385100	4.01147400	0.10478800
C	-3.85517600	2.28622200	-0.63162500
C	-4.34800000	3.60504200	-1.19460600
H	-3.89313800	4.40759700	-0.58857400
C	-1.27338000	3.24680000	-2.21819200
C	0.25678500	5.05652900	-1.65790700
H	0.99156500	5.80253300	-1.97240700
C	-1.84736200	2.31978700	-3.27662600
H	-2.68659000	1.76527400	-2.82564600
C	-0.10601700	4.94819200	-0.31968100
H	0.35387300	5.61100800	0.42030400
C	-0.32655900	4.20649300	-2.59305100
H	-0.03082400	4.27995200	-3.64477900
C	-1.43939500	3.95471700	1.56981200
H	-2.12228400	3.10028600	1.70802500
C	-5.83979100	3.82678900	-1.30241600
H	-6.05226500	4.83560700	-1.68133800
H	-6.34337000	3.73068700	-0.33000800
H	-6.31840300	3.11654100	-1.99149200
C	-0.22767800	3.71466000	2.45855300
H	0.35053300	2.84127200	2.12006000
H	-0.53345800	3.52133500	3.49769100
H	0.45058300	4.58188700	2.46429000
C	-0.81134000	1.28684600	-3.70887700
H	0.07260200	1.76975900	-4.15685000
H	-1.22274000	0.59367600	-4.45869000
H	-0.46660400	0.67764900	-2.86045900
C	-2.38323000	3.07556100	-4.48783300
H	-3.11398300	3.84796200	-4.20737600
H	-2.87851000	2.38966900	-5.19036600
H	-1.57904800	3.57912700	-5.04581300
C	-2.19526400	5.20966200	1.99555000
H	-1.56989000	6.11014700	1.88908800
H	-2.50265200	5.14655600	3.04973100
H	-3.10231600	5.36989500	1.39506000
N	2.59648000	-2.13648500	-0.44073900
C	1.78006200	-3.22289200	-0.87795200
C	1.06494600	-3.97079100	0.08351700
C	3.92429000	-2.31688700	-0.39750200
C	4.52524600	-3.66845800	-0.72770100
H	4.00305400	-4.13072000	-1.57768200
C	1.67039700	-3.52297100	-2.25476800
C	0.17584900	-5.35729000	-1.69834100

H -0.44518200 -6.19739200 -2.01958300
C 2.34702100 -2.64078600 -3.28489200
H 3.27585800 -2.24801100 -2.83752100
C 0.26895200 -5.03590100 -0.34766900
H -0.28557900 -5.62375500 0.38730900
C 0.86649700 -4.60005000 -2.64051900
H 0.77253100 -4.84803800 -3.70068500
C 1.12259900 -3.55792700 1.53882700
H 2.14095500 -3.17285900 1.72962800
C 4.50087600 -4.62429400 0.45637600
H 5.02743500 -5.55823600 0.21811400
H 3.47436500 -4.89440100 0.74068000
H 4.98677600 -4.18227800 1.33854100
C 0.13416700 -2.41661700 1.77576800
H 0.10231200 -1.68905600 0.94334900
H 0.31066100 -1.86807500 2.71141200
H -0.89760200 -2.79532000 1.81015200
C 1.45233700 -1.44093600 -3.58310300
H 0.52670300 -1.75753900 -4.08812100
H 1.95363700 -0.70487900 -4.22955500
H 1.14117900 -0.92314800 -2.66051900
C 2.73499200 -3.36671200 -4.56317900
H 3.34232500 -4.26076800 -4.35976000
H 3.32011600 -2.70828000 -5.22050300
H 1.85620500 -3.68845100 -5.14174000
C 0.86227600 -4.68305700 2.52634800
H -0.17451500 -5.04662700 2.46414000
H 1.01039200 -4.33337600 3.55780900
H 1.53222200 -5.53963900 2.36321300
N -3.43598100 -0.53166700 0.46635700
C -3.35345300 -1.88143200 0.89002700
C -3.14474700 -2.89484400 -0.08065500
C -4.61365600 -0.03653000 0.09292600
C -5.82560900 -0.94823600 0.15011300
H -5.84323800 -1.41942100 1.14613100
C -3.42753300 -2.20982800 2.26535400
C -3.07352000 -4.55181100 1.69747100
H -2.96954900 -5.59397100 2.01189600
C -3.67098500 -1.16877100 3.34275500
H -3.60017900 -0.17575800 2.87033300
C -3.00039300 -4.21722200 0.34659100
H -2.83865700 -5.00630300 -0.39176400
C -3.29120900 -3.55341500 2.63917400
H -3.35733500 -3.81773500 3.69909000
C -3.07561300 -2.53947500 -1.55476700
H -3.80380900 -1.72852900 -1.73192600
C -7.18357200 -0.35997800 -0.15993300
H -7.24797300 0.02642900 -1.18700300
H -7.44112700 0.46605000 0.51845700
H -7.96592400 -1.12349500 -0.05318300
C -1.70565700 -1.98494000 -1.92878400
H -1.46378500 -1.05976800 -1.38700200
H -1.65754800 -1.74836700 -3.00292600
H -0.91195400 -2.71751300 -1.71298200
C -2.60731400 -1.22006500 4.43331300
H -2.62052300 -2.17670200 4.97966600
H -2.77318900 -0.42205800 5.17194000
H -1.60465800 -1.06602900 4.01109600
C -5.06423600 -1.30981700 3.95191500
H -5.86388600 -1.21950300 3.20286800
H -5.24047100 -0.53517200 4.71281500
H -5.19033500 -2.28678300 4.44529300
C -3.44537800 -3.69300600 -2.47506300
H -2.67819300 -4.48301700 -2.46952400
H -3.52949300 -3.34405400 -3.51416800
H -4.40405100 -4.15613800 -2.19838800
H 0.12453900 1.22231300 -0.92193100
H -1.16650800 0.54642700 2.38556500
H 5.86802000 1.73820600 0.27720200
H 5.56420600 -3.51483100 -1.05264000
H -5.62943300 -1.80087400 -0.52407100
H -3.88376500 3.74494800 -2.18516900

Product - coplanar (**4b**)
161
-3111.640629

Mg -1.36090700 -0.35110100 -0.32761900
O -0.07636900 -0.38875400 1.15573500

N -2.13507300 -2.19882700 -0.85690500
C -3.09991000 -2.24265000 -1.78284200
C -3.94463400 -1.15620100 -2.07283100
H -4.71400400 -1.36560600 -2.81998100
C -4.13831900 0.04393800 -1.35287900
N -3.25028000 0.56365500 -0.50617200
C -1.61022500 -3.42513600 -0.36679200
C -2.05944200 -3.89284000 0.89480200
C -1.59350000 -5.13105900 1.34799900
H -1.93864300 -5.51876500 2.30879900
C -0.67454400 -5.87370600 0.61215400
H -0.32309500 -6.83785200 0.98770700
C -0.18009700 -5.36581800 -0.58298300
H 0.57704700 -5.92850600 -1.13592000
C -0.63539100 -4.14464600 -1.09197500
C -2.97967500 -3.04433600 1.75864500
H -2.59218200 -2.00731900 1.68766900
C -4.42670000 -3.00859600 1.27119300
H -4.52779000 -2.56687300 0.27141500
H -5.05083900 -2.41200100 1.95458300
H -4.85862700 -4.02119300 1.24128000
C -2.95220000 -3.43687600 3.22896000
H -3.41750200 -4.41974800 3.39892200
H -3.52451800 -2.71575000 3.83033800
H -1.93192400 -3.48014600 3.63645300
C -0.02828500 -3.56746800 -2.35358600
H -0.74542500 -2.84607600 -2.77721400
C 0.26749600 -4.60656100 -3.42562200
H 1.07339100 -5.29271200 -3.12445700
H 0.59907000 -4.12135400 -4.35469000
H -0.61422200 -5.21938500 -3.66526700
C 1.22960600 -2.78767000 -1.99487500
H 1.02033800 -2.02542100 -1.22659700
H 1.64221100 -2.27214200 -2.87356700
H 2.00586400 -3.45300900 -1.58932400
C -3.43290300 -3.52177600 -2.52876800
H -2.51688300 -4.08934600 -2.74147100
C -4.42083300 -4.43639700 -1.82220500
H -4.00752500 -4.83616700 -0.88531600
H -4.67609300 -5.29490500 -2.45870700
H -5.35777700 -3.91318800 -1.57939900
C -5.51870000 0.64696900 -1.51790800
H -5.52361500 1.72048900 -1.28703600
C -6.52562500 -0.06090500 -0.62319800
H -6.59743300 -1.13180400 -0.86395900
H -7.52852900 0.37659000 -0.72219300
H -6.23449800 0.01647300 0.43475100
C -3.56369800 1.79264300 0.14310200
C -3.46929100 3.00886400 -0.58530700
C -3.73024300 4.20523000 0.09067700
H -3.65760600 5.15310700 -0.44539700
C -4.09458000 4.22004400 1.43563900
H -4.29487200 5.16948900 1.93811000
C -4.20473800 3.02311800 2.12857900
H -4.49209000 3.03379300 3.18385600
C -3.94251100 1.79780300 1.50378500
C -3.13418300 3.00845800 -2.07008800
H -3.85310900 2.32557800 -2.55959600
C -1.73559200 2.46101700 -2.36693200
H -1.00770800 2.74851400 -1.59373300
H -1.35729500 2.84899500 -3.32372800
H -1.71711700 1.36646600 -2.44910900
C -3.32507900 4.36730800 -2.72721000
H -4.33275700 4.77580400 -2.56401300
H -3.17272500 4.28727100 -3.81246600
H -2.59782900 5.10488900 -2.35551800
C -4.01952800 0.52057000 2.31553700
H -4.01924900 -0.32074800 1.60196600
C -2.78225200 0.38869400 3.20093800
H -1.85318300 0.45333100 2.61627800
H -2.77501000 -0.56586300 3.74955300
H -2.74811100 1.19741300 3.94841200
C -5.28722800 0.41561700 3.15452900
H -5.32193400 1.17884900 3.94643400
H -5.34243500 -0.56135000 3.65795300
H -6.19931500 0.53168800 2.55166000
H -0.12632900 -0.79305300 2.02417400
Mg 1.43862200 0.41928700 0.18108600
N 2.16924500 2.36828200 0.29949900

C	3.26579900	2.67877500	0.98881500
C	4.12611600	1.71732600	1.56014800
H	4.95018800	2.13999800	2.12981500
C	4.20220500	0.33325500	1.34139200
N	3.28645800	-0.38361300	0.67301100
C	1.47743700	3.37789300	-0.42305800
C	1.67263500	3.46030900	-1.82029000
C	1.03026500	4.48327100	-2.52593400
H	1.18734600	4.57292300	-3.60370200
C	0.18847600	5.38278600	-1.88304800
H	-0.29992000	6.17864600	-2.45088300
C	-0.03798800	5.26035800	-0.51465200
H	-0.71215900	5.96234900	-0.01764700
C	0.59933200	4.26911800	0.23770400
C	2.59089600	2.48835400	-2.53469700
H	2.55623400	1.53724600	-1.97157000
C	4.04083600	2.96417800	-2.49598300
H	4.40678400	3.07539700	-1.46545400
H	4.70956600	2.25382500	-3.00602700
H	4.15021200	3.93947300	-2.99557300
C	2.14375500	2.18213000	-3.95577400
H	2.25366000	3.04930500	-4.62432500
H	2.75008100	1.37449200	-4.38916900
H	1.09179700	1.86310000	-3.98790700
C	0.36589700	4.13850700	1.72843800
H	1.32501300	3.84426100	2.18478100
C	-0.07325500	5.43094000	2.39737100
H	-1.08888300	5.72787800	2.09356900
H	-0.09454600	5.31082700	3.49002600
H	0.60318700	6.26768100	2.16592500
C	-0.62334100	3.01583500	2.01325800
H	-0.27204700	2.04614700	1.62941700
H	-0.79387700	2.89372700	3.09387900
H	-1.59366600	3.22784000	1.54345400
C	3.72425600	4.12040300	1.14641700
H	2.99760900	4.80401000	0.68718500
C	3.99649100	4.55864500	2.57645400
H	4.26466900	5.62361000	2.60940100
H	3.11619400	4.42411300	3.22198700
H	4.82376800	4.00526200	3.04132300
C	5.43613600	-0.38754100	1.84858000
H	5.10318500	-1.31324900	2.34492700
C	6.38697400	0.36216800	2.75394800
H	5.88418300	0.74865600	3.65211900
H	7.19422300	-0.30109300	3.09237500
H	6.86301100	1.21491300	2.25011100
C	3.59850400	-1.73925500	0.36965600
C	3.11469400	-2.78549400	1.18771900
C	3.39728700	-4.10919600	0.83044500
H	3.01465600	-4.92266300	1.45485800
C	4.16281000	-4.40751900	-0.29102200
H	4.37720200	-5.44667200	-0.55215000
C	4.65835900	-3.36989000	-1.07567400
H	5.26253000	-3.60428600	-1.95705400
C	4.39117700	-2.03313000	-0.76682000
C	2.33147000	-2.51397600	2.45683600
H	2.17217500	-1.42467300	2.51726200
C	0.96757400	-3.19072000	2.42283800
H	1.05381100	-4.28597100	2.49007400
H	0.34310800	-2.87025600	3.27315800
H	0.42495000	-2.96732700	1.49338400
C	3.10395200	-2.93832200	3.70310600
H	4.07695000	-2.43420600	3.78839500
H	2.53455300	-2.71071200	4.61650100
H	3.29780000	-4.02210700	3.70180100
C	4.93240200	-0.93375800	-1.66304600
H	4.84446100	0.02211300	-1.12025600
C	4.08897100	-0.81023800	-2.92761900
H	3.04065300	-0.56619300	-2.69718900
H	4.47722900	-0.02438900	-3.59303300
H	4.08601900	-1.75324900	-3.49732100
C	6.40359700	-1.12395500	-2.01254400
H	6.56831400	-2.00182000	-2.65541800
H	6.78837800	-0.25163900	-2.56119200
H	7.02791700	-1.25463300	-1.11664300
H	0.18349900	0.34276700	-1.22130300
H	-5.82416400	0.54913000	-2.57111700
H	-3.84480900	-3.23188900	-3.50724600
H	4.64811800	4.24264900	0.55461000

H 5.98314100 -0.76179600 0.96565800

Product - orthogonal (**4b**)
161
-3111.64315

Mg 1.37407400 -0.45603400 0.18000800
H 0.00063700 0.00767000 1.37754300
N 2.24325200 -2.15365900 -0.61469400
N 3.12607300 0.12651000 1.07576500
C 3.37534900 -2.67989300 -0.15629100
C 4.20782900 -2.02719700 0.77780200
H 5.09693700 -2.59024800 1.05390300
C 4.15618200 -0.70922200 1.26599600
C 1.50736100 -2.73335900 -1.67877600
C 1.57362900 -2.12809000 -2.95711300
C 0.75664500 -2.63016600 -3.97597800
H 0.78990300 -2.17430000 -4.96826700
C -0.10378500 -3.70119600 -3.75250600
H -0.73323300 -4.07706600 -4.56279200
C -0.15973100 -4.28714200 -2.49258900
H -0.84040600 -5.12462900 -2.31913400
C 0.62945600 -3.81457200 -1.43994900
C 2.51760400 -0.96558700 -3.20797200
H 2.50435400 -0.33924600 -2.29551700
C 2.09793000 -0.08026600 -4.37093800
H 2.15251000 -0.60726500 -5.33546300
H 2.75899100 0.79359700 -4.45614500
H 1.07205000 0.30279900 -4.25783800
C 3.95964700 -1.43329400 -3.38645800
H 4.32332500 -1.99184300 -2.51405800
H 4.63598700 -0.57662000 -3.53204000
H 4.05795500 -2.08726100 -4.26683800
C 0.50570600 -4.39279200 -0.04681800
H 1.49497600 -4.31651000 0.43132500
C -0.45351200 -3.55193500 0.78613000
H -0.16748900 -2.48666700 0.80481800
H -0.49404300 -3.89499100 1.83020900
H -1.47656500 -3.59844500 0.38223400
C 0.10131400 -5.85789300 -0.01737600
H -0.93557600 -6.00785800 -0.35619700
H 0.15485600 -6.25281600 1.00731500
H 0.75238900 -6.47865200 -0.65105600
C 3.88719100 -4.01278200 -0.67449600
H 4.80684400 -3.82129400 -1.25415000
H 3.17060400 -4.43167700 -1.39580400
C 4.19405600 -5.04274600 0.40068800
H 3.31625400 -5.25786300 1.02731900
H 4.99855500 -4.71997600 1.07551800
H 4.51311800 -5.99223700 -0.05078600
C 5.37383000 -0.18491800 2.00259600
H 5.83656300 0.58384100 1.35785900
H 5.02470000 0.39615500 2.87106400
C 6.42504600 -1.17833600 2.44402400
H 6.00158000 -1.96959200 3.07956200
H 7.20635400 -0.67388100 3.02807400
H 6.92508700 -1.66833800 1.59713200
C 3.28206600 1.48376200 1.47094300
C 3.93711600 2.38037500 0.59068300
C 4.14997600 3.69858600 1.00030300
H 4.65690400 4.39521000 0.32673000
C 3.72164000 4.14151800 2.24904300
H 3.91137200 5.17043200 2.56382600
C 3.02915700 3.27021100 3.08006100
H 2.66821600 3.62699900 4.04947100
C 2.77880700 1.94260800 2.70869100
C 4.29913500 1.95388000 -0.81867300
H 4.39752300 0.85505800 -0.83283400
C 3.14795500 2.32416000 -1.75296200
H 2.18564200 1.87399600 -1.44925700
H 3.34651900 2.00983800 -2.78835400
H 2.98833000 3.41455900 -1.76435100
C 5.61281400 2.53429100 -1.32217900
H 5.56245100 3.62500800 -1.45906100
H 5.87621100 2.10646100 -2.30039500
H 6.44478000 2.32746500 -0.63355900
C 1.99512200 1.04715000 3.65114500
H 1.76444300 0.11394200 3.11076600
C 0.66650500 1.68554200 4.04313100

H	0.80400000	2.65465600	4.54690800
H	0.10760900	1.04412400	4.73959700
H	0.02656800	1.85250100	3.16450200
C	2.80844100	0.67606500	4.88777400
H	3.74208600	0.15657300	4.62819100
H	2.23734400	0.00966900	5.55150800
H	3.08125900	1.56795300	5.47369300
Mg	-1.37571000	0.45188000	0.17712700
N	-2.23706100	2.15264600	-0.61986800
N	-3.13025400	-0.12543500	1.07036700
C	-3.37124200	2.67983600	-0.16817700
C	-4.20656700	2.03050400	0.76594300
H	-5.09556700	2.59530100	1.03859900
C	-4.15765000	0.71428600	1.25874600
C	-1.49425900	2.73103600	-1.67989300
C	-1.54980700	2.12242400	-2.95702200
C	-0.72749200	2.62433700	-3.97156700
H	-0.75292000	2.16598100	-4.96297800
C	0.12727800	3.69918500	-3.74514000
H	0.76062200	4.07554200	-4.55217600
C	0.17227900	4.28889900	-2.48648800
H	0.84804000	5.12992900	-2.31121600
C	-0.62159900	3.81591900	-1.43754500
C	-2.49109600	0.95886300	-3.21252300
H	-2.49060100	0.33811800	-2.29635900
C	-2.05784200	0.06591800	-4.36469200
H	-2.10010600	0.58669000	-5.33321200
H	-2.71833500	-0.80814600	-4.45238000
H	-1.03359800	-0.31752900	-4.23751300
C	-3.93028400	1.42725400	-3.41123900
H	-4.30205600	1.99390100	-2.54742500
H	-4.60655700	0.57050800	-3.55675400
H	-4.01785900	2.07420200	-4.29790700
C	-0.51044900	4.39922900	-0.04523200
H	-1.50562700	4.33066700	0.42176900
C	0.43432400	3.55751800	0.80328800
H	0.14051500	2.49470200	0.82769600
H	0.46667700	3.90802600	1.84526900
H	1.46159800	3.59430300	0.40948000
C	-0.09783700	5.86205700	-0.01732700
H	0.94439500	6.00388900	-0.34320000
H	-0.16220100	6.26197100	1.00478600
H	-0.73665500	6.48397300	-0.66222700
C	-3.88292600	4.00951700	-0.69489200
H	-4.79716300	3.81258300	-1.28130500
H	-3.16201900	4.42790600	-1.41217200
C	-4.20232700	5.04300800	0.37318900
H	-3.33107600	5.26272600	1.00732900
H	-5.01264400	4.72147000	1.04160300
H	-4.51916200	5.98989700	-0.08524400
C	-5.37525700	0.19618600	1.99980000
H	-5.83872200	-0.57785400	1.36205800
H	-5.02508100	-0.37766500	2.87274700
C	-6.42616400	1.19311000	2.43393500
H	-6.00271500	1.98899000	3.06362400
H	-7.20764200	0.69311300	3.02158500
H	-6.92597300	1.67684800	1.58329800
C	-3.29181000	-1.48204600	1.46539900
C	-3.95379000	-2.37479800	0.58615600
C	-4.16936200	-3.69306700	0.99398100
H	-4.68130100	-4.38659900	0.32099300
C	-3.73778200	-4.13992600	2.24023300
H	-3.92949600	-5.16890000	2.55360300
C	-3.04043200	-3.27216300	3.07080100
H	-2.67776200	-3.63169700	4.03858500
C	-2.78706000	-1.94464100	2.70111000
C	-4.32270900	-1.94387200	-0.82011100
H	-4.41514100	-0.84456300	-0.83172200
C	-3.18045300	-2.31772800	-1.76372200
H	-2.21387900	-1.87422200	-1.46428500
H	-3.38520200	-1.99721600	-2.79608300
H	-3.02769300	-3.40903700	-1.78105700
C	-5.64319900	-2.51586400	-1.31542100
H	-5.60034800	-3.60668800	-1.45403000
H	-5.91068900	-2.08504100	-2.29124300
H	-6.46918500	-2.30495900	-0.62086300
C	-1.99863600	-1.05370200	3.64392200
H	-1.77264100	-0.11672500	3.10804400
C	-0.66691000	-1.69277000	4.02441000

H -0.80051900 -2.66726800 4.51882400
H -0.10677200 -1.05710300 4.72514400
H -0.02968400 -1.84939400 3.14187900
C -2.80522400 -0.69209600 4.88776500
H -3.74289200 -0.17563300 4.63685800
H -2.23263200 -0.02654300 5.55102300
H -3.07033900 -1.58797400 5.47109800
O -0.00430200 -0.01479300 -1.12561700
H -0.00506900 -0.03099100 -2.08511700

[{(^{iPrDep}nacnac)Mg}$_2$(μ-O)] + H_2 → [{(^{iPrDep}nacnac)Mg}$_2$(μ-H))(μ-OH)]
M06-D3$_{PCM(benzene)}$/def2-TZVP//M06-L-D3/def2-SVP

[{(^{iPrDep}nacnac)Mg}$_2$(μ-O)]
147
-2953.247123

Mg 1.82607200 -0.03249900 -0.01447600
O 0.01576900 -0.04359400 0.02704500
N 3.17282500 1.33170000 0.66214900
C 5.10123300 0.08704700 -0.08650400
H 6.18576300 0.13824800 -0.14356700
C 2.59335400 2.54212300 1.11474700
C 2.43148200 3.60773900 0.19835800
C 4.48871500 1.22455500 0.47407500
C 5.40925700 2.34240000 0.95725200
H 4.83889400 3.28235500 0.88166900
C 2.10922900 2.65185800 2.43430400
C 1.47846700 4.95657900 1.98067900
H 1.06594700 5.90827700 2.32574000
C 2.10081700 1.47766600 3.37663600
H 2.83251100 0.72158500 3.04903000
C 1.88958200 4.80964600 0.65751600
H 1.77812900 5.64118300 -0.04514100
C 1.56404900 3.87328000 2.84787500
H 1.20160000 3.96689000 3.87699600
C 2.81872000 3.45593400 -1.25064800
H 3.91201600 3.32029500 -1.34305700
C 5.72439900 2.13158300 2.43651100
H 6.26232600 1.18387400 2.59335600
H 4.81204200 2.10277400 3.04797200
H 6.35667600 2.94393800 2.82315300
C 6.68212500 2.52419800 0.14451700
H 7.18097900 3.46203500 0.42789500
H 6.48297300 2.56581700 -0.93596700
H 7.41139800 1.71837400 0.31500200
C 2.11457200 2.33195700 -2.01248000
H 2.66871800 1.37914700 -1.95468900
H 2.04612500 2.56270100 -3.08502800
H 1.08239200 2.16795000 -1.65671000
C 0.71937400 0.84218300 3.48102400
H -0.02412800 1.56741300 3.84807700
H 0.71775600 -0.01102400 4.17458300
H 0.36443800 0.48528400 2.49765300
Mg -1.78071000 -0.06066600 -0.16636700
N -3.04503400 1.36390700 -0.85503300
C -5.05790300 0.12803900 -0.36596800
H -6.14345600 0.19330000 -0.41855000
C -2.38809600 2.56664400 -1.21869000
C -1.94668100 3.42900500 -0.18886400
C -4.37324800 1.25026000 -0.87157000
C -5.22378600 2.41098800 -1.37856400
H -4.58932800 2.99402400 -2.06570200
C -2.08741200 2.84020700 -2.56885800
C -0.99869700 4.89728700 -1.87932300
H -0.46887000 5.81823300 -2.13648900
C -2.44438600 1.84928500 -3.64224900
H -3.43388700 1.40675300 -3.44329600
C -1.27279000 4.60057800 -0.54606800
H -0.94359700 5.28814800 0.23618600
C -1.39019600 4.01207300 -2.87944100
H -1.15852000 4.22898300 -3.92711100
C -2.15007800 3.03600100 1.25307500
H -3.08691700 2.46227700 1.34889700
C -6.45705700 1.98300700 -2.16108000
H -7.23589300 1.54760500 -1.51790800
H -6.21743500 1.24208700 -2.93720700
H -6.91095700 2.85208100 -2.65861000

C	-5.59465300	3.33394300	-0.22078100
H	-6.19879400	4.18228200	-0.57395800
H	-4.70451300	3.74843100	0.27283400
H	-6.18222500	2.79749200	0.54041000
C	-1.41013900	0.74007600	-3.76294000
H	-0.42336200	1.14479300	-4.03177700
H	-1.68764200	-0.00497300	-4.52243900
H	-1.26849400	0.20017600	-2.81053800
C	-2.15025900	4.16629700	2.26105700
H	-1.18635100	4.69121000	2.30069300
H	-2.34934900	3.78871500	3.27362600
H	-2.92455600	4.91370300	2.02951600
N	3.24423200	-1.40567200	-0.51712700
C	2.76327500	-2.69040400	-0.88641300
C	2.40647500	-3.60767400	0.12419700
C	4.55258900	-1.13560600	-0.52044100
C	5.54630000	-2.21148800	-0.95262300
H	5.00283900	-2.90318200	-1.61543500
C	2.58469400	-3.02071500	-2.24869900
C	1.76957500	-5.21280100	-1.58904200
H	1.39288900	-6.20132600	-1.86287100
C	2.84555600	-1.99796700	-3.31989400
H	3.71686400	-1.37699200	-3.05591900
C	1.90715100	-4.86107000	-0.25017900
H	1.62680100	-5.57092400	0.53481000
C	2.10000500	-4.28889800	-2.57858400
H	1.97229700	-4.54781300	-3.63453100
C	2.49178200	-3.23774200	1.58089600
H	3.27889500	-2.48284100	1.73807500
C	6.73668300	-1.68486200	-1.74219900
H	7.45193400	-1.13233900	-1.11526700
H	6.42889000	-1.01639900	-2.55901400
H	7.29313000	-2.52160500	-2.18846200
C	6.00117700	-3.01921900	0.25993600
H	6.70065100	-3.81337000	-0.03908100
H	5.15649800	-3.50016800	0.77224700
H	6.51531400	-2.37735800	0.99196000
C	1.16367400	-2.72175800	2.11800000
H	0.78445000	-1.84899400	1.55431000
H	1.23579100	-2.43747900	3.17854400
H	0.38098000	-3.48850200	2.03022900
C	1.63792400	-1.10182600	-3.55596800
H	0.78403900	-1.67901000	-3.94012600
H	1.85408400	-0.30147800	-4.27839500
H	1.28349500	-0.62302700	-2.62519200
N	-3.27121900	-1.33914900	0.34468900
C	-2.83316300	-2.56284400	0.91232300
C	-2.52280500	-3.64539000	0.06618900
C	-4.56933500	-1.04603000	0.23943600
C	-5.61563000	-2.03002000	0.75880000
H	-5.09825000	-2.70873400	1.45614500
C	-2.62097600	-2.64855500	2.30707000
C	-1.92664300	-4.95887300	2.02044600
H	-1.58807000	-5.90154500	2.45721300
C	-2.84994900	-1.42261400	3.14987900
H	-3.89592400	-1.08545600	3.02977400
C	-2.07136800	-4.83862400	0.64252500
H	-1.83273800	-5.68395400	-0.01086800
C	-2.18666800	-3.86316900	2.84335600
H	-2.03151700	-3.95362800	3.92119400
C	-2.59265100	-3.49483100	-1.42877100
H	-3.41481000	-2.81746600	-1.70758500
C	-6.74256200	-1.36203000	1.53665100
H	-7.42448700	-0.79378900	0.88719100
H	-6.36365400	-0.66835800	2.30086600
H	-7.35269900	-2.12048400	2.04823700
C	-6.16580400	-2.88635500	-0.37763200
H	-6.90957900	-3.60361600	-0.00104500
H	-5.37624600	-3.46429400	-0.87692500
H	-6.65845600	-2.26467100	-1.14112100
C	-1.28723600	-2.97343600	-2.01239700
H	-0.96392100	-2.01999000	-1.55466000
H	-1.36183700	-2.81485200	-3.09831300
H	-0.45912800	-3.67258600	-1.82895300
C	-2.51427700	-1.55311100	4.61836800
H	-3.13686200	-2.30897500	5.11900100
H	-2.67027300	-0.60243800	5.14544600
H	-1.46368800	-1.84216900	4.77090100
H	2.80094400	-4.12245300	2.16156200

H 3.11142200 -2.50741500 -4.25958900
H 2.43265700 1.80595800 4.37595600
H 2.60784300 4.41302100 -1.75169200
H -1.34898400 2.31173400 1.51439000
H -2.53459900 2.37094400 -4.60813900
H -2.26150800 -0.59120800 2.71282600
H -2.83074900 -4.46913500 -1.88520100

H_2
2
-1.170703414

H 0.00000000 0.00000000 0.37726500
H 0.00000000 0.00000000 -0.37726500

Transition state
149
-2954.387634

Mg 1.81987500 -0.34262700 -0.09588000
O 0.00836500 -0.17384700 -0.36404900
N 2.63186100 -2.09313100 -0.69678900
C 4.90667100 -1.33969200 -0.50481800
H 5.93310600 -1.67495800 -0.64226100
C 1.64294800 -3.08360900 -0.93532500
C 1.20131100 -3.87669700 0.14155000
C 3.92926600 -2.28947600 -0.88131800
C 4.43033800 -3.60029700 -1.47626000
H 3.54946200 -4.11003800 -1.90113800
C 0.99675100 -3.13429000 -2.18913200
C -0.52483000 -4.80196500 -1.29128200
H -1.38009700 -5.46822000 -1.42844700
C 1.46528100 -2.21757300 -3.28599700
H 2.53965300 -2.39934200 -3.47441300
C 0.11354100 -4.73339800 -0.05816900
H -0.24350300 -5.34152700 0.77893000
C -0.08397000 -4.00532100 -2.34633100
H -0.60568000 -4.04759400 -3.30509100
C 1.80164300 -3.72259800 1.51314800
H 2.85576500 -3.41219200 1.44475000
C 5.42701300 -3.38412900 -2.60829300
H 6.38966800 -2.99172800 -2.24876300
H 5.04792400 -2.68012200 -3.36269700
H 5.63957600 -4.33504600 -3.11781600
C 5.00754900 -4.51290900 -0.39918700
H 5.34395500 -5.46484600 -0.83487800
H 4.26799800 -4.75106800 0.37747400
H 5.87495100 -4.05012300 0.09627000
C 1.03500400 -2.71585700 2.35618100
H 0.96064400 -1.73276600 1.85754400
H 1.49752000 -2.55564900 3.34162200
H -0.00540100 -3.03848600 2.50878400
C 0.69190400 -2.28415900 -4.58307700
H 0.72996000 -3.28629500 -5.03513700
H 1.09715600 -1.57852700 -5.32030900
H -0.36606100 -2.02627900 -4.42912500
Mg -1.86381900 0.06274600 -0.59225500
N -3.57789000 -0.65618100 0.23290300
C -4.85572800 1.34607500 -0.24034000
H -5.86431400 1.75402500 -0.19199800
C -3.47038200 -1.95754300 0.78435100
C -3.91864000 -3.09804600 0.08103400
C -4.73012800 0.02103300 0.22020000
C -5.97938600 -0.61992700 0.81827100
H -5.85294900 -1.71043000 0.73413000
C -2.79912300 -2.09526900 2.02497600
C -3.13619800 -4.49719200 1.91192500
H -3.02065800 -5.48801400 2.35800900
C -2.21336300 -0.87100100 2.68046200
H -2.92736200 -0.03399900 2.59781100
C -3.74146800 -4.35650500 0.66886500
H -4.08550800 -5.24636700 0.13354800
C -2.66238000 -3.37006000 2.58009100
H -2.17484300 -3.48635100 3.55150700
C -4.55513600 -2.98518900 -1.28120000
H -4.72136000 -1.92703000 -1.52918900
C -6.05348600 -0.29809100 2.30866700
H -6.12458300 0.78810600 2.47542300
H -5.16670500 -0.66278600 2.84720400

H	-6.93417100	-0.76780900	2.77051900
C	-7.27622100	-0.26824100	0.10503600
H	-8.09117900	-0.91636700	0.45912000
H	-7.19578100	-0.39894100	-0.98395600
H	-7.59611300	0.76755400	0.29135700
C	-1.76516900	-1.01585300	4.11880600
H	-2.59227400	-1.32858300	4.77402100
H	-1.38369100	-0.06053700	4.50658200
H	-0.95900000	-1.75414400	4.23923700
C	-5.85412600	-3.75602200	-1.44368800
H	-5.72033400	-4.83809700	-1.30365500
H	-6.27849300	-3.61328800	-2.44715600
H	-6.61328100	-3.43047200	-0.71626400
N	3.59593500	0.54061000	0.29944500
C	3.54257300	1.83332300	0.87922700
C	3.89305300	2.97105100	0.11569200
C	4.77304900	-0.07631100	0.09290300
C	6.05212300	0.56839300	0.62450600
H	5.90269400	1.65887000	0.61346100
C	3.04041000	1.97173900	2.19171700
C	3.46275200	4.35916700	2.06278000
H	3.46018600	5.34399800	2.53543300
C	2.47821700	0.76217200	2.89627200
H	3.13944500	-0.10576400	2.72707000
C	3.85808400	4.22406400	0.73345500
H	4.14062700	5.11371500	0.16526600
C	3.03526900	3.24322800	2.77625200
H	2.68741200	3.36373400	3.80500800
C	4.25436600	2.79478200	-1.33544900
H	5.09883800	2.08908600	-1.42594300
C	6.23928400	0.16682000	2.08667300
H	6.34535100	-0.92450400	2.18603300
H	5.38873400	0.48045900	2.70873500
H	7.14203600	0.63204800	2.50821700
C	7.30601700	0.28216700	-0.18774400
H	8.12697300	0.93362600	0.14388700
H	7.15407000	0.45895100	-1.26224600
H	7.66052100	-0.75224400	-0.06865100
C	2.19718400	0.90644800	4.37630700
H	3.10689600	1.17338400	4.93418900
H	1.81607500	-0.03435400	4.79599700
H	1.44454300	1.67974000	4.58608900
C	4.57317600	4.05802100	-2.10350000
H	3.72815200	4.76171700	-2.10835600
H	4.80954700	3.83019600	-3.15107500
H	5.43987600	4.58944200	-1.68358800
N	-2.58259700	1.95667700	-0.74940500
C	-1.54321700	2.91073600	-0.87447800
C	-0.84317200	3.25452600	0.30869300
C	-3.86376200	2.27690100	-0.61747600
C	-4.31618000	3.71798900	-0.82709600
H	-3.46884600	4.25244600	-1.28705700
C	-1.10898400	3.37757500	-2.13013500
C	0.69942600	4.57004800	-1.02283100
H	1.57480300	5.22267700	-1.07576800
C	-1.82168700	3.00305900	-3.40299100
H	-2.62834600	2.28931700	-3.17952400
C	0.26877500	4.09158500	0.21449000
H	0.82415100	4.36397100	1.11549700
C	0.01856100	4.20946500	-2.17909900
H	0.36606500	4.57421700	-3.15118500
C	-1.27948400	2.64730200	1.61595700
H	-2.38006400	2.66474000	1.68569400
C	-5.49540200	3.82662700	-1.78566900
H	-6.42501000	3.42840300	-1.35304000
H	-5.31243000	3.28447700	-2.72467900
H	-5.68708500	4.87934700	-2.04107200
C	-4.61231200	4.40124300	0.50351400
H	-4.91447600	5.44745000	0.34888200
H	-3.73273700	4.40892100	1.16312900
H	-5.42799700	3.89429700	1.04189700
C	-2.38190800	4.19226500	-4.16761700
H	-3.10626300	4.75891500	-3.56366900
H	-2.89598500	3.87482100	-5.08583200
H	-1.59261600	4.89842500	-4.46374900
C	-0.67983600	3.24624400	2.86828900
H	0.41453500	3.14207900	2.89513800
H	-1.07811700	2.75712300	3.76865300
H	-0.90205300	4.32096700	2.95261900

H -0.42161100 -0.66899300 -1.45884200
H -1.13840700 -0.88199600 -2.15252800
H -1.12186600 2.45109800 -4.05474900
H -3.82632400 -3.33426600 -2.03584300
H -1.34792900 -0.54571500 2.06473400
H -1.03808000 1.56580000 1.57630500
H 1.80246300 -4.69696100 2.02783200
H 1.43922400 -1.17832700 -2.90305500
H 1.52356400 0.48576800 2.39148400
H 3.41958300 2.25979400 -1.82613300

Product-coplanar
149
-2954.458195

Mg 1.36065000 -0.22880300 -0.11957700
O 0.03658300 0.09738500 1.26242400
N 2.87169800 1.06193100 -0.58236000
C 4.05878800 0.63587300 -1.02201700
C 4.47535300 -0.70805400 -0.97520300
H 5.47765400 -0.88454200 -1.36330100
C 3.89918900 -1.80966100 -0.30961800
N 2.66169100 -1.80117100 0.17875800
C 2.66232100 2.44266800 -0.33463300
C 2.95306900 2.93325300 0.95743800
C 2.78357300 4.29894100 1.20768700
H 3.01536800 4.70183700 2.19699000
C 2.32016700 5.15197600 0.21093200
H 2.19638400 6.21803100 0.41836600
C 1.98159500 4.64494400 -1.04046200
H 1.58660200 5.32108400 -1.80224200
C 2.13151100 3.28609500 -1.33264800
C 3.38664500 1.96362900 2.02647900
H 2.55058200 1.25490700 2.18937800
C 3.80046700 2.56255400 3.35197600
H 4.64454300 3.25970700 3.23956000
H 4.11756400 1.77973400 4.05480000
H 2.98143700 3.11812100 3.83254700
C 1.76661600 2.69875300 -2.66950500
H 2.62330800 2.11481100 -3.05092700
C 1.31221200 3.68590600 -3.72136900
H 0.42554900 4.25150100 -3.39723600
H 1.04801700 3.17384200 -4.65654600
H 2.09491700 4.42164800 -3.95958600
C 5.08479200 1.63833800 -1.55046500
H 4.54694000 2.57963300 -1.74586000
C 5.71028800 1.19880100 -2.86877300
H 6.29852100 2.02002300 -3.30335600
H 4.95030600 0.90596500 -3.60722100
H 6.39596100 0.34734400 -2.74826500
C 6.15408400 1.94283200 -0.50601500
H 5.72512900 2.38829400 0.40275700
H 6.89327200 2.65549900 -0.90029100
H 6.69529900 1.03058000 -0.20973800
C 4.81566000 -3.00612600 -0.06965300
H 4.17929300 -3.85778800 0.21805200
C 5.61646700 -3.42835200 -1.29405800
H 4.98346800 -3.53862000 -2.18655300
H 6.10917600 -4.39466600 -1.11385200
H 6.41185300 -2.71101000 -1.54396400
C 5.73165100 -2.71064900 1.11523000
H 6.40402800 -1.86691600 0.89532100
H 6.35550300 -3.58248000 1.36098100
H 5.15740100 -2.44875400 2.01507000
C 2.11839500 -2.90583700 0.87840600
C 1.63411800 -4.02389200 0.15864500
C 1.04919200 -5.07490000 0.87133700
H 0.68558200 -5.94428700 0.31570900
C 0.92824300 -5.03434900 2.25789300
H 0.47018600 -5.86844200 2.79530300
C 1.38817500 -3.92116100 2.95222600
H 1.28175600 -3.88241400 4.03996300
C 1.98068300 -2.84291300 2.28500600
C 1.76311600 -4.12288100 -1.34006700
H 2.83067400 -4.20150600 -1.61551100
C 1.11788000 -3.01230700 -2.15795200
H 0.14258300 -2.71846500 -1.74214300
H 0.93463900 -3.34424600 -3.18965600
H 1.74222600 -2.10879400 -2.22854400

C 2.46262900 -1.64065700 3.05470700
H 3.46680900 -1.84198700 3.47147200
C 1.54579600 -1.18529700 4.17646500
H 0.50408300 -1.08328200 3.83277500
H 1.86501300 -0.21414600 4.58277300
H 1.52504300 -1.88999800 5.01998300
H 0.08879300 0.16283000 2.21691200
Mg -1.49620500 0.03841600 0.02861700
N -3.04753700 -1.28980800 -0.28798800
C -4.29769900 -1.05817400 0.11481500
C -4.71362400 0.15667300 0.69248300
H -5.75205700 0.17847300 1.02061300
C -4.05075600 1.39593200 0.77225900
N -2.79251300 1.58555400 0.37690100
C -2.68868100 -2.53770000 -0.85818900
C -2.52848000 -2.66334600 -2.25590200
C -2.16282600 -3.91028200 -2.77980800
H -2.05602600 -4.01647600 -3.86391000
C -1.93122300 -5.00381700 -1.95548100
H -1.64851600 -5.96873600 -2.38382500
C -2.06351500 -4.85936300 -0.57604300
H -1.88524500 -5.71391700 0.08340000
C -2.44145200 -3.64149900 -0.00512600
C -2.73079200 -1.50431700 -3.19893600
H -3.05938500 -0.61641600 -2.63494000
C -1.49697700 -1.16877300 -4.02382600
H -1.15167000 -2.03201000 -4.61190600
H -1.69779100 -0.35455000 -4.73409700
H -0.66053500 -0.85339300 -3.38158700
C -2.63037100 -3.55038700 1.48823700
H -3.69751100 -3.37027700 1.71437900
C -1.78793900 -2.51945900 2.22829300
H -2.22381700 -1.50904700 2.19330200
H -1.69878600 -2.78128200 3.29254000
H -0.76516600 -2.46462800 1.82639100
C -5.38737600 -2.09915500 -0.13077400
H -4.89157500 -3.07439300 -0.25679600
C -6.38731200 -2.24603200 1.00762400
H -7.01604900 -3.13394700 0.84870900
H -5.89285000 -2.35890500 1.98341500
H -7.07078500 -1.38756300 1.08305100
C -6.09219800 -1.78320500 -1.44765400
H -5.38414300 -1.74131400 -2.28691300
H -6.84621400 -2.54764400 -1.68584600
H -6.60346400 -0.80925300 -1.39695800
C -4.87394400 2.56124800 1.31797700
H -4.18534200 3.40967700 1.45775700
C -5.47664400 2.24028900 2.68121100
H -4.72088100 1.86630400 3.38657300
H -5.92942800 3.14018700 3.12269900
H -6.26897800 1.47989400 2.61859800
C -5.94670400 3.00727400 0.33069400
H -6.66250800 2.19737400 0.12116300
H -6.51701400 3.85784800 0.73193000
H -5.51573600 3.32462900 -0.62887300
C -2.25312600 2.89236500 0.26070300
C -1.54707900 3.48460200 1.33194900
C -0.99064800 4.75382100 1.14363900
H -0.44154500 5.22428500 1.96402500
C -1.12970700 5.43350900 -0.06199900
H -0.68872500 6.42587400 -0.18469800
C -1.82012800 4.83827000 -1.11232600
H -1.93271800 5.36637900 -2.06519600
C -2.38771600 3.56781700 -0.97344300
C -1.45038800 2.79717400 2.66966500
H -1.75941600 1.74658600 2.55807100
C -0.08013400 2.86022600 3.31829800
H 0.21956300 3.88690500 3.57465900
H -0.05057000 2.27855100 4.25189000
H 0.68922000 2.46203300 2.64068600
C -3.09367000 2.93064900 -2.14113600
H -3.88173600 2.24617400 -1.79071000
C -2.15310400 2.16666400 -3.05871300
H -1.62715900 1.35728000 -2.52476000
H -2.69367700 1.70686000 -3.89825700
H -1.37738900 2.82261100 -3.47955800
H -0.14696200 -0.15957900 -1.23763800
H 0.98337600 1.93195700 -2.51153500
H 4.19331500 1.31497600 1.64193900

H 2.62866000 -0.81449700 2.34746700
H 1.32270300 -5.08586100 -1.64255400
H -2.20067200 3.23714200 3.35327300
H -3.60740200 3.71518000 -2.72051400
H -3.56575000 -1.74090500 -3.88281900
H -2.41722600 -4.54679900 1.90549600

[{(^{MeMe}nacnac)Mg}$_2$(μ-O)] + H_2 → [{(^{MeMe}nacnac)Mg}$_2$(μ-H))(μ-OH)]
M06-D3$_{PCM(benzene)}$/def2-TZVP//M06-L-D3/def2-SVP

[{(^{MeMe}nacnac)Mg}$_2$(μ-O)]
47
-1243.438694

Mg 1.81083500 -0.00000500 -0.00001600
O 0.00000000 -0.00000600 -0.00002900
N 3.17674700 1.49724100 -0.00000100
C 5.08635500 0.00000700 0.00001200
H 6.17716100 0.00001000 0.00002300
C 4.49138700 1.27967900 0.00000900
C 5.42673700 2.45533200 0.00002000
H 5.26002500 3.09728200 -0.87833600
Mg -1.81083400 0.00000000 -0.00001500
N -3.17675300 1.49724100 -0.00000700
C -5.08635500 -0.00000100 0.00001000
H -6.17716100 -0.00000200 0.00001700
C -4.49139200 1.27967400 0.00000500
C -5.42674700 2.45532300 0.00001400
H -5.26004000 3.09727000 -0.87834500
N 3.17675700 -1.49724300 -0.00000900
C 4.49139600 -1.27967000 0.00000400
C 5.42675600 -2.45531500 0.00001500
H 5.26005300 -3.09726400 -0.87834500
N -3.17675200 -1.49724300 0.00000000
C -4.49139100 -1.27967600 0.00000800
C -5.42674500 -2.45532500 0.00001800
H -5.26003100 -3.09726400 0.87838200
C -2.70026100 -2.86196900 0.00000200
H -3.02533700 -3.43740300 -0.88551400
H -1.60031900 -2.87158500 -0.00000300
H -3.02532900 -3.43739900 0.88552200
C -2.70026200 2.86196800 -0.00001100
H -3.02533300 3.43740300 0.88550500
H -1.60032000 2.87158400 -0.00001200
H -3.02533400 3.43739600 -0.88553100
C 2.70025000 2.86196500 -0.00000300
H 3.02532200 3.43740100 0.88551200
H 3.02531700 3.43739600 -0.88552300
H 1.60030800 2.87157700 -0.00000100
C 2.70027300 -2.86197200 -0.00001400
H 3.02535200 -3.43740100 -0.88553100
H 3.02534200 -3.43740400 0.88550500
H 1.60033100 -2.87159300 -0.00002000
H 6.47771100 -2.14726700 0.00002500
H 5.26003500 -3.09726200 0.87837200
H 6.47769400 2.14729300 0.00002600
H 5.26001400 3.09727400 0.87838100
H -6.47770200 -2.14728200 0.00001600
H -5.26003000 -3.09727900 -0.87833500
H -6.47770300 2.14727900 0.00002300
H -5.26002400 3.09726800 0.87837100

H_2
2
-1.170703414

H 0.00000000 0.00000000 0.37726500
H 0.00000000 0.00000000 -0.37726500

Int-1
49
-1244.611967

Mg 1.79451400 -0.02972600 -0.03046400
O -0.02258100 -0.01476100 -0.02457900
N 3.16670200 1.03739200 -1.07890100
C 5.07297900 0.05148600 0.05293900
H 6.16340000 0.07248400 0.07503100

C 4.48006000 0.91367900 -0.89418500
C 5.41767900 1.73323200 -1.73508100
H 5.26940400 1.53405000 -2.80732500
Mg -1.83400700 0.01814300 0.00974700
N -3.19593600 1.21785700 0.91353800
C -5.11101200 0.02155900 0.02308000
H -6.20181000 0.02296000 0.02768000
C -4.51149600 1.04532500 0.78713400
C -5.44379100 1.98799800 1.49402400
H -5.27282600 3.02873300 1.17944900
N 3.16156000 -1.00239400 1.11267000
C 4.47562400 -0.82845700 0.98072200
C 5.40921900 -1.60698400 1.86405500
H 5.26289200 -2.69104300 1.74248300
N -3.20658700 -1.17758300 -0.88203600
C -4.52043900 -1.00368200 -0.74604500
C -5.45951000 -1.94477800 -1.44586000
H -5.28793500 -2.98567000 -1.13207000
C -2.73195200 -2.26992200 -1.70084200
H -3.05695600 -2.19933300 -2.75456900
H -1.63201500 -2.27777800 -1.70685900
H -3.05675800 -3.26144400 -1.33708600
C -2.71666100 2.31099100 1.72942700
H -3.03494800 2.23971100 2.78508200
H -1.61709300 2.32158400 1.72924600
H -3.04584500 3.30182200 1.36794500
C 2.69228700 1.95664200 -2.08812700
H 2.98808000 3.00442600 -1.89891800
H 3.04707900 1.70907900 -3.10513900
H 1.59307100 1.93836300 -2.12081700
C 2.68252900 -1.94110000 2.10157500
H 3.03831900 -2.97358200 1.93165100
H 2.97305000 -1.67503700 3.13404200
H 1.58338500 -1.97532100 2.08044900
H 6.46029400 -1.38102500 1.65510700
H 5.22076600 -1.39511700 2.92759500
H 6.46783300 1.53545100 -1.49522300
H 5.23526100 2.81038900 -1.60096600
H -6.50944400 -1.70087100 -1.25204900
H -5.30197900 -1.92861100 -2.53507000
H -6.49543000 1.74585600 1.30748100
H -5.27884500 1.97125800 2.58211300
H 1.99436700 -1.88328900 -1.72366500
H 1.25597500 -1.73380500 -1.60413700

Int-2
49
-1244.612267

Mg 1.79220800 0.00000100 0.11848300
O -0.02317300 0.00000200 0.18021700
N 3.15512200 -1.49759600 -0.01620400
C 5.06037800 -0.00000100 -0.13435400
H 6.14890900 -0.00000200 -0.20552900
C 4.46637300 -1.28013900 -0.10200000
C 5.40027600 -2.45530100 -0.17149600
H 5.28863800 -3.10675600 0.70859200
Mg -1.83157200 0.00000100 0.07183900
N -3.19527500 -1.49723000 0.00011300
C -5.10217100 -0.00000100 -0.09860100
H -6.19150100 -0.00000200 -0.15548500
C -4.50815000 -1.27983200 -0.06841700
C -5.44277600 -2.45512600 -0.11843600
H -5.32172000 -3.09906600 0.76589900
N 3.15512400 1.49759700 -0.01620200
C 4.46637500 1.28013700 -0.10199700
C 5.40028000 2.45529800 -0.17149200
H 5.28864300 3.10675200 0.70859700
N -3.19527700 1.49723100 0.00011300
C -4.50815200 1.27983000 -0.06841700
C -5.44278000 2.45512300 -0.11843600
H -5.23193100 3.09526400 -0.98859900
C -2.71737700 2.86129300 0.02356800
H -3.08776300 3.43891400 0.88961500
H -1.61895000 2.86874300 0.08272100
H -2.99233300 3.43610700 -0.87914100
C -2.71737200 -2.86129200 0.02356800
H -2.99232800 -3.43610700 -0.87914000
H -1.61894500 -2.86874000 0.08272000

H -3.08775600 -3.43891300 0.88961500
C 2.67620800 -2.86062700 0.00919600
H 2.93749400 -3.43381800 -0.89873600
H 3.05718900 -3.44191100 0.86858600
H 1.57838200 -2.86701800 0.08279300
C 2.67621300 2.86062800 0.00920100
H 3.05719400 3.44191000 0.86859200
H 2.93750000 3.43382000 -0.89873000
H 1.57838600 2.86702100 0.08279800
H 6.44919400 2.14654900 -0.23397100
H 5.17948600 3.08803600 -1.04465500
H 6.44919000 -2.14655300 -0.23397400
H 5.17948100 -3.08803700 -1.04466000
H -6.49216100 2.14633900 -0.17159400
H -5.32172500 3.09906300 0.76589900
H -6.49215800 -2.14634300 -0.17159400
H -5.23192700 -3.09526600 -0.98859900
H 2.13938000 -0.00000100 2.57770500
H 1.38809500 0.00000000 2.44860600

Transition State
49
-1244.595239

Mg 1.76747200 -0.51602600 -0.26978200
O 0.07067600 -1.06822300 -0.70614700
N 3.62124100 -1.29155400 -0.40512900
C 4.67225000 0.68724000 0.53784500
H 5.64905700 1.09188200 0.80540700
C 4.70075600 -0.61295400 -0.00883600
Mg -1.82400500 -0.82269900 -0.76768800
N -3.27149500 -1.53362300 0.46613800
C -4.60262300 0.49381500 0.37175600
H -5.53008100 0.92881600 0.74669700
C -4.34560300 -0.82448500 0.80696400
N 2.31107000 1.24528300 0.54262400
C 3.58417300 1.54906600 0.79473300
N -2.69008400 0.99648200 -1.03412800
C -3.84966500 1.33642400 -0.47264700
H -0.45775000 -1.69065400 -1.68807100
H -1.23737600 -1.98063300 -2.26423100
C -4.41731900 2.70251800 -0.73700900
H -3.72335000 3.49295600 -0.41241700
H -4.57786600 2.86580400 -1.81356100
H -5.37230200 2.85960500 -0.22431500
C -2.02384600 1.95743400 -1.88150000
H -2.61141300 2.23378100 -2.77585200
H -1.77662600 2.90322200 -1.36483100
H -1.07316100 1.53904400 -2.24582000
C -3.13746000 -2.87721000 0.97986300
H -3.95963600 -3.54707900 0.67014000
H -2.20618800 -3.32954400 0.60846800
H -3.09289900 -2.92076800 2.08311100
C -5.37210900 -1.44213700 1.71428700
H -5.79435500 -2.35757900 1.27245400
H -4.92555400 -1.74826200 2.67256900
H -6.19958300 -0.75746100 1.92875000
C 3.80202300 -2.62004000 -0.94954700
H 2.82973900 -3.04437800 -1.23848500
H 4.43643600 -2.63723600 -1.85316900
H 4.25524900 -3.32625400 -0.23159800
C 1.28678300 2.21845200 0.85154700
H 1.39084400 3.15599000 0.27609900
H 0.29537600 1.80697900 0.61056400
H 1.26349800 2.50308100 1.91823500
C 3.91048000 2.88765400 1.39138200
H 3.54861800 3.70685500 0.75183900
H 3.41208200 3.02150700 2.36345400
H 4.98676100 3.02272700 1.54081800
C 6.05239400 -1.25333500 -0.14345600
H 6.26676400 -1.51586600 -1.19042700
H 6.85545700 -0.60015400 0.21327700
H 6.10472800 -2.19665900 0.42088300

Int-3
49
-1244.645902

Mg 0.12379100 -0.54191000 -0.88851000

O -0.47732200 -2.35827000 -1.35934300
N   2.19100100 -0.73660600 -0.87211200
C   2.41593000   1.48816900   0.04578600
H   3.10531500   2.13206800   0.59435600
C   2.91144700   0.20223600 -0.26935200
C   4.33842300 -0.08044700   0.10981300
H   4.41437500 -0.96663000   0.75947700
Mg -1.64141700 -2.35513100   0.22430500
N -0.21009800 -1.06116800   1.27170700
C -1.78185400   0.75296800   1.51635500
H -2.08194200   1.58487500   2.15465900
C -0.66486000   0.01020700   1.94339100
C   0.03072100   0.46075200   3.18991700
H   1.07707600   0.72180000   2.96874000
N   0.26789500   1.50416200 -1.08420600
C   1.23478600   2.11765800 -0.40178700
C   1.09702800   3.58002300 -0.08251100
H   0.17035400   3.77496400   0.48055300
N -2.25587100 -0.41964200 -0.52661400
C -2.55868000   0.53480400   0.36177400
C -3.75332400   1.41230600   0.14819000
H -4.67727100   0.81375200   0.15308500
C -3.12062200 -0.57620300 -1.68075600
H -3.18806400   0.33158900 -2.30756000
H -2.72473100 -1.38021300 -2.31751000
H -4.15741000 -0.84694400 -1.41127500
C   0.90757200 -1.79155300   1.84399600
H   0.66537900 -2.23744300   2.82452700
H   1.18369800 -2.61723500   1.17106600
H   1.81226700 -1.17305200   1.97723900
C   2.84773000 -1.97194100 -1.21878000
H   3.67633900 -1.83745200 -1.93766300
H   3.27708100 -2.50384100 -0.34813900
H   2.12963200 -2.66099900 -1.68314300
C -0.79705400   2.32294000 -1.60729000
H -1.36544600   2.86299400 -0.82591100
H -0.44235200   3.08942200 -2.32007000
H -1.52093500   1.70184400 -2.15430000
H   1.94062100   3.95368900   0.50777800
H   1.03012500   4.18865200 -0.99684200
H   4.80423200   0.76504900   0.62767800
H   4.94626200 -0.30748700 -0.77920700
H -3.84429300   2.18318600   0.92118600
H -3.72001400   1.90678100 -0.83392500
H -0.45312700   1.33563500   3.63691100
H   0.06348200 -0.33864200   3.94468100
H -0.07358100 -3.06904200 -1.85454500
H -2.61092800 -3.55540900   0.97946500

Product - coplanar
49
-1244.652564

Mg -1.43989000   0.00000000   0.00612700
O   0.00000400 -0.00000200   1.32796500
N -2.81136500 -1.50019300 -0.04846500
C -4.12396000 -1.27994000 -0.09917900
C -4.71772100   0.00000000 -0.12019200
H -5.80768300   0.00000100 -0.16203600
C -4.12396000   1.27994000 -0.09917400
N -2.81136500   1.50019300 -0.04846000
C -5.06157100 -2.45369600 -0.13815200
H -4.92931000 -3.10046400   0.74256100
C -5.06157200   2.45369600 -0.13814300
H -4.86519000   3.09159100 -1.01334400
H   0.00000500 -0.00000400   2.28590400
Mg   1.43989100   0.00000000   0.00612100
N   2.81136500   1.50019300 -0.04847700
C   4.12396000   1.27994000 -0.09921400
C   4.71772000   0.00000000 -0.12024400
H   5.80768100   0.00000000 -0.16210700
C   4.12395900 -1.27994000 -0.09921900
N   2.81136500 -1.50019300 -0.04848300
C   5.06157100   2.45369600 -0.13819700
H   4.86517600   3.09159200 -1.01339300
C   5.06157000 -2.45369600 -0.13820600
H   4.86517400 -3.09158900 -1.01340500
H -0.00000300   0.00000100 -1.20154100
C -2.34206300   2.86522700 -0.03615900

H	-2.63093100	3.43735500	-0.93630100
H	-2.69856300	3.44485200	0.83459600
H	-1.24281700	2.88384700	0.00620700
C	2.34206300	2.86522700	-0.03616500
H	1.24281800	2.88384600	0.00622200
H	2.69858000	3.44485000	0.83458400
H	2.63091300	3.43735700	-0.93631100
C	2.34206300	-2.86522700	-0.03617600
H	2.63091300	-3.43735300	-0.93632500
H	2.69858100	-3.44485400	0.83457000
H	1.24281800	-2.88384600	0.00621200
C	-2.34206400	-2.86522700	-0.03616900
H	-2.63093300	-3.43735200	-0.93631300
H	-1.24281900	-2.88384800	0.00619700
H	-2.69856500	-3.44485500	0.83458400
H	-6.11104900	2.14286400	-0.17515400
H	-4.92931100	3.10046100	0.74257200
H	-6.11104900	-2.14286400	-0.17516200
H	-4.86519000	-3.09158800	-1.01335500
H	6.11104700	-2.14286400	-0.17523500
H	4.92932400	-3.10046300	0.74251000
H	6.11104800	2.14286300	-0.17522600
H	4.92932600	3.10045900	0.74252200

5 References

(1) Bourne, C.; Dong, H.; McKain, K.; Mayer, L. C.; McKay, A. P.; Cordes, D. B.; Slawin, A. M. Z.; Stasch, A. Alkyl backbone variations in common β-diketiminate ligands and applications to N-heterocyclic silylene chemistry. *Dalton Trans.* **2024**, *53*, 9887−9895.

(2) Lalrempuia, R.; Stasch, A.; Jones, C. The reductive disproportionation of CO_2 using a magnesium(I) complex: analogies with low valent f-block chemistry. *Chem. Sci.* **2013**, *4*, 4383−4388.

(3) Bonyhady, S. J.; Jones, C.; Nembenna, S.; Stasch, A.; Edwards, A. J.; McIntyre, G. J. β-Diketiminate-Stabilized Magnesium(I) Dimers and Magnesium(II) Hydride Complexes: Synthesis, Characterization, Adduct Formation, and Reactivity Studies. *Chem. Eur. J.* **2010**, *16*, 938–955.

(4) Burnett, S.; Bourne, C.; Slawin, A. M. Z.; van Mourik, T.; Stasch, A. Umpolung of an Aliphatic Ketone to a Magnesium Ketone-1,2-diide Complex with Vicinal Dianionic Charge. *Angew. Chem. Int. Ed.* **2022**, *61*, e202204472.

(5) Borys, A. M. An Illustrated Guide to Schlenk Line Techniques, *Organometallics* **2023**, *42*, 182−196.

(6) Green, S. P.; Jones, C.; Stasch, A. Stable Adducts of a Dimeric Magnesium(I) Compound. *Science* **2007**, *318*, 1754–1757.

(7) Green, S. P.; Jones, C.; Stasch, A. Stable Adducts of a Dimeric Magnesium(I) Compound. *Angew. Chem. Int. Ed.* **2008**, *47*, 9079–9083.

(8) Fulmer, G. R.; Miller, A. J. M.; Sherden, N. H.; Gottlieb, H. E.; Nudelman, A.; Stoltz, B. M.; Bercaw, J. E.; Goldberg, K. I. NMR Chemical Shifts of Trace Impurities: Common Laboratory Solvents, Organics, and Gases in Deuterated Solvents Relevant to the Organometallic Chemist. *Organometallics* **2010**, *29*, 2176–2179.

(9) *CrystalClear-SM Exper*t v2.1. Rigaku Americas, *The Woodlands, Texas, USA*, and Rigaku Corporation, *Tokyo, Japan*, 2015.

(10) *CrysAlisPro* v1.171.40.14a, 41.82a, 42.49, .42.74a, 42.93a, 42.96a, 43.109a, 43.126a, Rigaku Oxford Diffraction, Rigaku Corporation, *Tokyo, Japan*, 2018−2024.

(11) Sheldrick, G. M. SHELXT – Integrated space-group and crystal structure determination. *Acta Crystallogr., Sect. A: Found. Adv*. **2015**, *71*, 3−8.

(12) Burla, M. C.; Caliandro, R.; Camalli, M.; Carrozzini, B.; Cascarano, G. L.; Giacovazzo, C.; Mallamo, M.; Mazzone, A.; Polidori, G.; Spagna, R. SIR2011: a new package for crystal structure determination and refinement. *J. Appl. Crystallogr.* **2012**, *45*, 357−361.

(13) Sheldrick, G. M. Crystal structure refinement with SHELXL. *Acta Crystallogr., Sect. C: Struct. Chem.* **2015**, *71*, 3−8.

(14) Spek, A. L. Structure validation in chemical crystallography. *Acta Crystallogr. Sect D: Biol. Crystallogr.* **2009**, *65*, 148−155.

(15) *CrystalStructure* v4.3.0. Rigaku Americas, *The Woodlands, Texas, USA*, and Rigaku Corporation, *Tokyo, Japan*, 2018.

(16) Dolomanov, O. V.; Bourhis, L. J.; Gildea, R. J.; Howard, J. A. K.; Puschmann, H. OLEX2: a complete structure solution, refinement and analysis program. *J. Appl. Crystallogr.* **2009**, *42*, 339−341.

(17) Poater, A.; Cosenza, B.; Correa, A.; Giudice, S.; Ragone, F.; Scarano, V.; Cavallo, L. Samb *V*ca: A Web Application for the Calculation of the Buried Volume of N-Heterocyclic Carbene Ligands. *Eur. J. Inorg. Chem.* **2009**, 1759–1766.

(18) Mantina, M.; Chamberlin, A. C.; Valero, R.; Cramer, C. J.; Truhlar, D. G. Consistent van der Waals Radii for the Whole Main Group. *J. Phys. Chem. A* **2009**, *113*, 5806–5812.

(19) Zhao, Y.; Truhlar, D. G. The M06 suite of density functionals for main group thermochemistry, thermochemical kinetics, noncovalent interactions, excited states, and transition elements: two new functionals and systematic testing of four M06-class functionals and 12 other functionals. *Theor. Chem. Acc.* **2008**, *120*, 215–241.

(20) Grimme, S.; Antony, J.; Ehrlich, S.; Krieg, H. A consistent and accurate *ab initio* parametrization of density functional dispersion correction (DFT-D) for the 94 elements H-Pu. *J. Chem. Phys.* **2010**, *132*, 154104.

(21) Weigend, F.; Ahlrichs, R. Balanced basis sets of split valence, triple zeta valence and quadruple zeta valence quality for H to Rn: Design and assessment of accuracy. *Phys. Chem. Chem. Phys.* **2005**, *7*, 3297–3305.

(22) Miertus, S.; Scrocco, E.; Tomasi, J. Electrostatic interaction of a solute with a continuum. A direct utilization of AB initio molecular potentials for the prevision of solvent effects. *Chem. Phys.* **1981**, *55*, 117–129.

(23) Reed, A. E.; Weinstock, R. B.; Weinhold, F. Natural population analysis. *J. Chem. Phys.* **1985**, *83*, 735–746.

(24) AIMAll (Version 19.10.12), Keith, T. A. TK Gristmill Software, Overland Park KS, USA, 2019 (aim.tkgristmill.com).

(25) Gaussian 16, Revision C.01, Frisch, M. J.; Trucks, G. W.; Schlegel, H. B.; Scuseria, G. E.; Robb, M. A.; Cheeseman, J. R.; Scalmani, G.; Barone, V.; Petersson, G. A.; Nakatsuji, H.; Li, X.; Caricato, M; Marenich, A. V.; Bloino, J.; Janesko, B.G.; Gomperts, R.; Mennucci, B.; Hratchian, H.P.; Ortiz, J.V.; Izmaylov, A.F.; Sonnenberg, J.L.; Williams-Young, D.; Ding, F.; Lipparini, F.; Egidi, F.; Goings, J.; Peng, B.; Petrone, A.; Henderson, T.; Ranasinghe, D.; Zakrzewski, V.G.; Gao, J.; Rega, N.; Zheng, G.; Liang, W.; Hada, M.; Ehara, M.; Toyota, K.; Fukuda, R.; Hasegawa, J.;

Ishida, M.; Nakajima, T.; Honda, Y.; Kitao, O.; Nakai, H.; Vreven, T.; Throssell, K.; Montgomery Jr., J.A.; Peralta, J.E.; Ogliaro, F.; Bearpark, M.J.; Heyd, J.J.; Brothers, E.N.; Kudin, K.N.; Staroverov, V.N.; Keith, T.A.; Kobayashi, R.; Normand, J.; Raghavachari, K.; Rendell, A.P.; Burant, J.C.; Iyengar, S.S.; Tomasi, J.; Cossi, M.; Millam, J.M.; Klene, M.; Adamo, C.; Cammi, R.; Ochterski, J.W.; Martin, R.L.; Morokuma, K.; Farkas, O.; Foresman, J.B.; Fox, D.J. Gaussian, Inc., Wallingford CT, 2016.

(26) Contreras-García, J.; Johnson, E. R.; Keinan, S.; Chaudret, R.; Piquemal, J. P.; Beratan, D. N.; Yang, W. NCIPLOT: a program for plotting noncovalent interaction regions. *J. Chem. Theory Comput.* **2011**, *7*, 625–632.

(27) Boys, S. F.; Bernardi, F. J. M. P. The calculation of small molecular interactions by the differences of separate total energies. Some procedures with reduced errors. *Mol. Phys.* **1970**, *19*, 553–566.

(28) Burnett, S.; Ferns, R.; Cordes, D. B.; Slawin, A. M. Z.; van Mourik, T.; Stasch, A. Low-Coordinate Magnesium Sulfide and Selenide Complexes Low-Coordinate Magnesium Sulfide and Selenide Complexes. *Inorg. Chem*. **2023**, *62*, 16443−16450.
